# Multi-omics and high-spatial-resolution omics: deciphering complexity in neurological disorders

**DOI:** 10.1093/gigascience/giaf137

**Published:** 2025-12-05

**Authors:** Xiuyun Liu, Fangfang Li, Marek Czosnyka, Zofia Czosnyka, Huijie Yu, Xiaoguang Tong, Yan Xing, Hongliang Li, Ke Pu, Keke Feng, Kuo Zhang, Meijun Pang, Dong Ming

**Affiliations:** State Key Laboratory of Advanced Medical Materials and Devices, Medical School, Tianjin University, Tianjin, 300072, China; Haihe Laboratory of Brain-Computer Interaction and Human-Machine Integration, Tianjin, 300380, China; School of Pharmaceutical Science and Technology, Tianjin University, Tianjin, 300072, China; State Key Laboratory of Advanced Medical Materials and Devices, Medical School, Tianjin University, Tianjin, 300072, China; Department of Clinical Neurosciences, Addenbrooke’s Hospital, University of Cambridge, Cambridge, CB2 0QQ, UK; Department of Clinical Neurosciences, Addenbrooke’s Hospital, University of Cambridge, Cambridge, CB2 0QQ, UK; Department of Neurosurgery, Tianjin Medical University General Hospital, Tianjin, 300052, China; Department of Neurosurgery, Tianjin Huanhu Hospital, Tianjin, 300350, China; Department of Neurology, Aviation General Hospital, Beijing, 100012, China; Department of Neurology, Aviation General Hospital, Beijing, 100012, China; Department of Neurosurgery, Tianjin Huanhu Hospital, Tianjin, 300350, China; Department of Neurosurgery, Tianjin Huanhu Hospital, Tianjin, 300350, China; State Key Laboratory of Advanced Medical Materials and Devices, Medical School, Tianjin University, Tianjin, 300072, China; Haihe Laboratory of Brain-Computer Interaction and Human-Machine Integration, Tianjin, 300380, China; State Key Laboratory of Advanced Medical Materials and Devices, Medical School, Tianjin University, Tianjin, 300072, China; Haihe Laboratory of Brain-Computer Interaction and Human-Machine Integration, Tianjin, 300380, China; State Key Laboratory of Advanced Medical Materials and Devices, Medical School, Tianjin University, Tianjin, 300072, China; Haihe Laboratory of Brain-Computer Interaction and Human-Machine Integration, Tianjin, 300380, China

**Keywords:** multi-omics, neurological diseases, single-cell omics, spatial transcriptomics

## Abstract

**Background:**

The world has witnessed a steady rise in neurological diseases, which represent a heterogeneous group of disorders characterized by complex pathogenesis involving disruptions at multiple molecular levels, including genomic, transcriptomic, proteomic, and metabolomic levels. These disorders, often caused by genetic mutations, metabolic imbalances, immune dysregulation, and environmental factors, pose significant challenges to global public health due to their high prevalence, mortality, and disability burden.

**Results:**

The advent of high-throughput technologies, such as next-generation sequencing and mass spectrometry, has provided valuable insights into the underlying mechanisms of disease, especially the development of multi- and high-spatial-resolution omics technologies, enabling the interaction of multiple levels of biology and analysis of the complex molecular networks and pathophysiological processes.

**Conclusions:**

This review provides a comprehensive analysis of the latest advancements in multi- and high-spatial-resolution omics, with a focus on their applications in precision diagnostics, biomarker discovery, and therapeutic target identification in brain diseases. The study also highlights the current challenges in the clinical implementation and discusses the future directions, with artificial intelligence being anticipated to enhance clinical translation and diagnostic accuracy significantly.

## Graphical Abstract

**Graphical Abstract:**
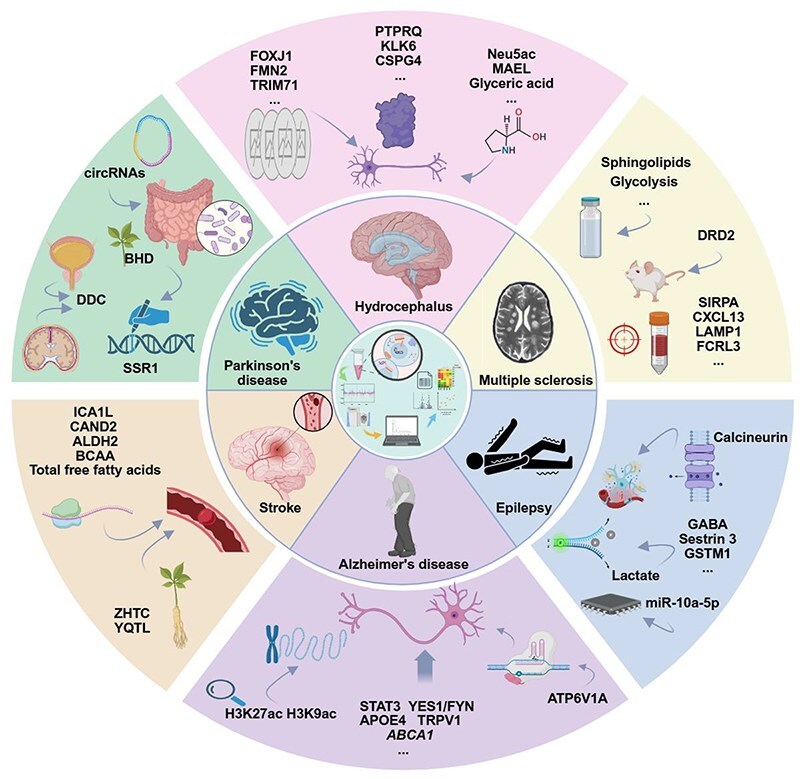
Applications of multi-omics and high-spatial-resolution omics technologies in the diagnosis and treatment of brain diseases in the field of neurology. By integrating high-throughput omics technologies, including 4 basic omics, single-cell omics, and spatial omics technologies, it is possible to dissect the complex pathogenic mechanisms of neurological disorders comprehensively. This multi-omics approach spans multiple levels, from genetic variations to metabolic changes, and reveals the interactions between these levels, providing an unprecedented perspective for in-depth disease understanding. In the study of diseases such as Alzheimer’s disease, Parkinson’s disease, stroke, epilepsy, multiple sclerosis, and hydrocephalus, the application of these cutting-edge technologies has greatly facilitated the discovery of key biomarkers and significantly deepened our understanding of the molecular mechanisms of disease pathogenesis.

## Highlights

- Forty-three percent of the global population has neurological disorders, yet diagnostic and therapeutic strategies remain limited, primarily due to the complexity involving disruptions at multiple molecular levels, including genomic, transcriptomic, proteomic, and metabolomic levels.
- Rapid advances in multi- and high-spatial-resolution omics enable the interaction of multiple levels of biology, showing great potential in the analysis of the complex molecular networks and pathophysiological processes of brain disease.
- This review provides a comprehensive analysis of the latest advancements in multi- and high-spatial-resolution omics, with a focus on their applications in precision diagnostics, biomarker discovery, and therapeutic target identification in brain diseases.

## Introduction

Neurological brain diseases encompass a wide range of brain disorders that affect the structure or function of the central and peripheral nervous systems. Globally, an estimated 43% of the world population has neurological diseases, becoming the leading cause of overall disease burden in the world [1]. New treatments that can completely resolve brain diseases have yet to be discovered, due to the blood–brain barrier (BBB), unclear disease mechanisms, and limited brain analysis tools. Conventional approaches for brain imaging, such as magnetic resonance imaging (MRI) or computed tomography (CT), provide us with abundant information on brain structure and function, but they lack interpretation at the cellular level. Developing advanced analytical technologies is essential for unraveling the complexities of the brain and detecting disease-related therapeutic targets for early diagnosis and precise medicine [2–4].

Since the concept of genomics was first proposed by Thomas H. Roderick in 1986 [5], omics technologies, such as genomics, proteomics, metabolomics, lipidomics, glycomics, and transcriptomics, have gained rapid development [6]. However, basic omics, which employs a singular approach to elucidate the isolated function of a single molecule of a biological system, provides information that is highly fragmented and limited. As the foundation for all living organisms, understanding the complex process by which genetic information is transformed into functional proteins, including transcriptional regulation, translational regulation, RNA/polymer degradation, post-translational modification, and differential transport, plays a critical role in revealing the basic mechanism of brain diseases and discovering new targets [7]. Multiple omics, which integrates basic omics technologies and combines diverse omics data to extract meaningful information, enables a systematic analysis of the mechanisms and phenotypes of complex biological processes and advances our understanding of the regulatory relationships among various molecules [6, 8–10].

In recent years, the development of high-spatial-resolution (single-cell and spatial) omics technologies has significantly advanced the study of neurological brain diseases. These technologies allow the detailed profiling of molecular changes at the single-cell and tissue-level spatial resolution, thereby uncovering the heterogeneity of disease mechanisms and revealing localized pathophysiological alterations [11]. Single-cell omics provides insights into cellular diversity and gene expression dynamics [12], while spatial omics preserves tissue architecture to map molecular events in their anatomical context [13]. The integration of multi-omics with high-spatial-resolution omics further enhances the biological relevance and depth of disease characterization, offering a more comprehensive view of neurodegenerative and inflammatory brain disorders.

This article provides a systematic review of multi-omics and high-spatial-resolution omics techniques and their application in the neurology field for brain disease diagnosis and treatment. We also discuss the current challenges and prospects, aiming to offer researchers a comprehensive and evidence-based perspective on the development, utility, and translational potential of multi- and high-spatial-resolution omics approaches in the neuroscience area.

## Omics Technology

### Basic omics technology

Different basic omics technologies, including genomics, transcriptomics, proteomics, and metabolomics, have been developed for neuroscience researchers, with distinct advantages and disadvantages shown in Table 1.

**Table 1:** Overview of the 4 omics technologies

Technology	Precision	Price range	Advantages	Disadvantages	Disease application representation
Genomics	High	High	● Conduct a comprehensive analysis of genetic sequences. ● Uncover the depth of genetic variations. ● Be suitable for gene discovery and genetic disease studies.	● High experimental costs. ● Complex techniques and large sample sizes. ● Considerable time in analysis.	● Genetic disorders. ● Cancer genomics. ● Genetic counseling.
Transcriptomics	Medium	Medium	● Reveal dynamic changes in gene expression. ● Differentiate gene regulatory networks. ● Assist in the classification of disease subtypes.	● High experimental and data analysis design. ● Limited real-time to reflect messenger RNA levels.	● Mental disorders. ● Cardiovascular diseases. ● Prognostication and efficacy assessment in cancer.
Proteomics	Low	Medium	● Reflect protein levels and modifications directly. ● Reveal protein–protein interaction networks. ● Explore changes in protein function.	● Complex data analysis with low standardization. ● Effects of PTMs.	● Development of tumor biomarkers. ● Mechanistic studies in autoimmune diseases. ● Pathology of neurodegenerative diseases.
Metabolomics	Medium	Medium	● Provide the overall metabolic profile of the organism. ● Reflect metabolic changes associated with disease. ● Assist in early diagnosis and monitoring.	● Sensitive sample handling and storage conditions. ● Challenges in the detection of metabolites.	● Monitoring of endocrine disorders. ● Prediction of cardiovascular disease risk. ● Metabolic testing in diabetes.
Single-cell omics	High	High	● Resolve cellular heterogeneity (tumor subclones). ● Identify rare cell types (<0.1% population). ● Enable multi-omics integration (ATAC + RNA).	● Spatial information loss from tissue dissociation. ● Significant technical noise (dropout rate >15%). ● Single-cell amplification bias.	● Tumor evolutionary tree construction. ● T-cell receptor clonal tracking. ● Nervous diseases neuronal subtyping.
Spatial omics	High	High	● Preserve in situ spatial topology. ● Quantify cell–cell interactions (immune synapses). ● Directly correlate pathological morphology with molecular expression.	● Resolution inversely proportional to throughput (e.g., MERFISH: ~1,000 genes). ● Optical diffraction limitations (>200 nm). ● High complexity in multidimensional data integration.	● Tumor immune exclusion zone mapping. ● Brain region–specific protein gradient atlases. ● Myocardial infarction spatial injury demarcation.

PTM: posttranslational modification.

#### Genomics

Genomics investigates the entirety of an organism’s genetic material, encompassing DNA sequences, gene repertoire, and regulatory elements. It emphasizes the holistic analysis of genome architecture, functionality, evolution, and regulation, utilizing high-throughput sequencing and bioinformatics for comparative genomic data analysis across individuals and species [8].

Genomic research leverages advanced technologies such as next-generation sequencing, CRISPR-mediated gene editing, and genome-wide association studies (GWAS) to explore genetic variations and their influence on disease susceptibility and progression. GWAS, specifically, employ statistical models to detect associations between genetic variants and phenotypic traits across large populations, providing a robust framework for elucidating the genetic underpinnings of complex neurological conditions [14]. In the realm of neurological brain disorders, genomics has been pivotal in pinpointing risk loci, clarifying disease mechanisms, and guiding the development of targeted therapies and precision medicine strategies [15]. Nevertheless, this field is still facing several challenges, including difficulty in interpretation for noncoding variations, challenges in clinical translation, and so on.

#### Transcriptomics

Transcriptomics, developed by Charles Auffray in 1999, systematically investigates RNA transcripts and their regulatory networks to unravel the molecular underpinnings of cellular function and disease [16, 17].

By utilizing high-throughput RNA sequencing (RNA-seq) and other cutting-edge technologies to profile the transcriptome in specific cell types or tissues, transcriptomics facilitates the identification of gene expression patterns, alternative splicing events, and the role of noncoding RNAs in the progression of neurological diseases. Transcriptomic studies encounter challenges, including spatial and temporal heterogeneity of RNA expression in complex tissues [18, 19], instability of RNA, and the lack of mature analytical methods.

#### Proteomics

Proteomics, introduced by Marc Wilkins in 1994, has been widely used to investigate the protein composition and its dynamic changes in cells, tissues, or organisms, serving as a crucial tool for exploring the structure and function of proteins [20]. It serves as a powerful complement to genomics and transcriptomics, encompassing protein identification, comparative proteomics, glycomics, targeted proteomics, and so on, which have been applied in new drug development and synthetic biology.

The most prevalent analysis methods for proteomics include the data-dependent acquisition (DDA) mode, also known as the “shotgun” approach; isobaric tags for relative and absolute quantification (iTRAQ) [21]; tandem mass tag (TMT) technology [22]; stable-isotope labeling by amino acids in cell culture (SILAC) [23]; label-free quantification [24]; and data-independent acquisition [25], especially the data-independent acquisition/sequential window acquisition of all-theoretical mass spectral approach (DIA/SWATH), with their advantages and disadvantages depicted in Table 2. Additionally, mass spectrometry–based sequencing is used to identify and analyze posttranslational modifications (PTMs) [26]. These technologies enable proteomics to be an indispensable tool for brain disease diagnosis and treatment, which offers crucial insights into the molecular pathophysiology underlying neurological disorders.

**Table 2:** Comparative analysis of 5 mainstream proteomics techniques.

Technology	Introduction	Advantages	Disadvantages	Labeling groups	Data volume	Cost
iTRAQ	Employ chemical labels to identify proteins.	● Simultaneous analysis of up to 8 groups. ● Processing of multiple samples. ● Enhanced throughput.	● Expensive reagents. ● Complex experimental procedures.	4 or 8	Medium	High
TMT	Utilize chemical labeling to identify proteins.	● Simultaneous analysis of up to 10 or 11 groups. ● Simultaneous processing of more samples. ● Higher sensitivity.	● Expensive reagents. ● Complex experimental operations.	10 or 11	Medium	High
SILAC	Introduce isotope-labeled amino acids into the culture media.	● Accurate quantification. ● High sensitivity.	● Requirement for cell culture. ● Unsuitability for clinical samples.	2 or 3	Low	Medium
Label-free	Detect endogenous peptides without labeling.	● No need for labeling. ● Simple sample preparation. ● Cost-effective.	● Reduced reproducibility. ● Slightly diminished sensitivity.	Unlimited	High	Low
DIA/SWATH	Obtain mass spectrometry data through a full scan.	● No labeling required. ● Simultaneous quantification of numerous proteins. ● Good reproducibility.	● Complex data analysis. ● Need for specialized software.	Unlimited	High	Medium

DIA: data-independent acquisition; iTRAQ: isobaric tags for relative and absolute quantification; SILAC: stable-isotope labeling by amino acids in cell culture; SWATH: sequential window acquisition of all theoretical mass spectral approach; TMT: tandem mass tag.

#### Metabolomics

Introduced by Nicholson et al. [27] in 1999, metabolomics investigates the metabolic responses of living organisms to exogenous stimuli, environmental changes, or genetic modifications, mapping out comprehensive dynamic profiles of metabolite alterations. As a relatively recent addition to the omics framework following genomics and proteomics, metabolomics aims to characterize small molecules with molecular weights typically ranging from 100 to 1,000 Da, either qualitatively or quantitatively, to uncover their functional roles in health and disease [28].

**Figure 1:**
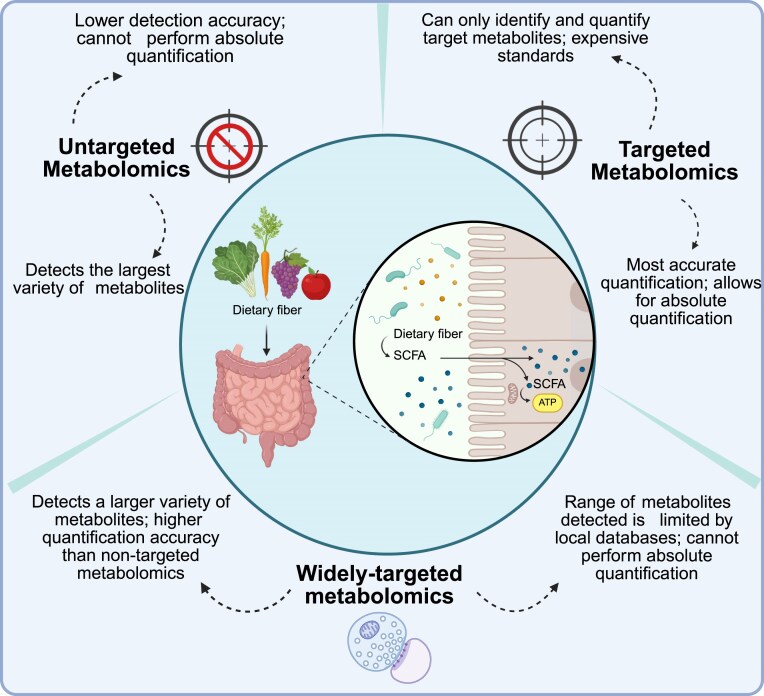
Three main types of metabolomics, including targeted metabolomics, untargeted metabolomics, and widely targeted metabolomics. This figure outlines these 3 metabolomics techniques, each with unique strengths and limitations. Careful selection among them can lead to the optimal choice to meet the specific requirements of your experimental objectives.

Metabolomic analysis employs a variety of analytical platforms, including nuclear magnetic resonance (NMR), Fourier transform– infrared (FT-IR) spectroscopy, gas chromatography–mass spectrometry (GC-MS), and liquid chromatography–mass spectrometry (LC-MS), which provide high-throughput, high spatial resolution, and sensitive detection of metabolite profiles. Currently, metabolomics can be broadly classified into 3 main approaches based on detection principles: untargeted metabolomics [29], targeted metabolomics [30], and widely targeted metabolomics [31]. Untargeted metabolomics offers broad coverage of the metabolome but at the expense of precision. In contrast, targeted metabolomics enables precise quantification of a predefined set of metabolites, although it limits the discovery of novel biomarkers. Widely targeted metabolomics, meanwhile, strikes a balance between breadth and specificity, allowing for both comprehensive detection and reliable quantification of a larger subset of known metabolites. As illustrated in Fig. 1, these methodologies collectively support the exploration of metabolic alterations in complex diseases.

In the context of neurological brain diseases, metabolomics has demonstrated significant potential in identifying disease-specific metabolite signatures that can be employed as diagnostic or prognostic biomarkers [28]. It provides a functional readout of biological processes and has been increasingly used to detect early metabolic changes, assess disease progression, and guide therapeutic interventions. However, several challenges remain. First, the comprehensive understanding of the metabolome is still in its infancy, with less than 5% of detected metabolites currently annotated. Second, the dynamic and context-dependent nature of metabolic profiles introduces complexity in data interpretation. Third, technical variability across platforms and sample types can hinder reproducibility and comparability. Despite these limitations, ongoing advancements in analytical technologies and bioinformatic tools are expected to position metabolomics as a powerful and indispensable strategy for more precise and efficient diagnosis of neurological disorders.

### Single-cell omics technology

First introduced in 2009 by Tang et al. [32] for RNA-seq at the single-cell level, single-cell omics has evolved into a high-throughput platform for analyzing genomic [33–35], transcriptomic, epigenomic [34], and proteomic profiles with cellular resolution [36]. Unlike bulk sequencing, which provides averaged signals across cell populations, single-cell approaches enable the deconvolution of individual cellular states, revealing heterogeneity with unprecedented precision. Single-cell transcriptomics remains the most mature and widely applied modality, having advanced from low-throughput methods to high-throughput platforms capable of profiling millions of cells. Representative technologies include full-length amplification (e.g., SMART-seq), high-throughput barcoding (e.g., 10x Genomics), and multi-omics compatible methods (e.g., Andeplete) [37]. Key steps in the workflow encompass cell isolation, nucleic acid amplification, sequencing, and computational analysis, with isolation and amplification being particularly critical for data quality. These technologies are increasingly integrated with spatial and epigenomic approaches to provide comprehensive insights into gene regulation, cellular function, and disease mechanisms. Despite significant progress, challenges such as amplification bias, cell dropout, and data standardization remain, necessitating continued methodological innovation.

### Spatial omics technology

**Figure 2:**
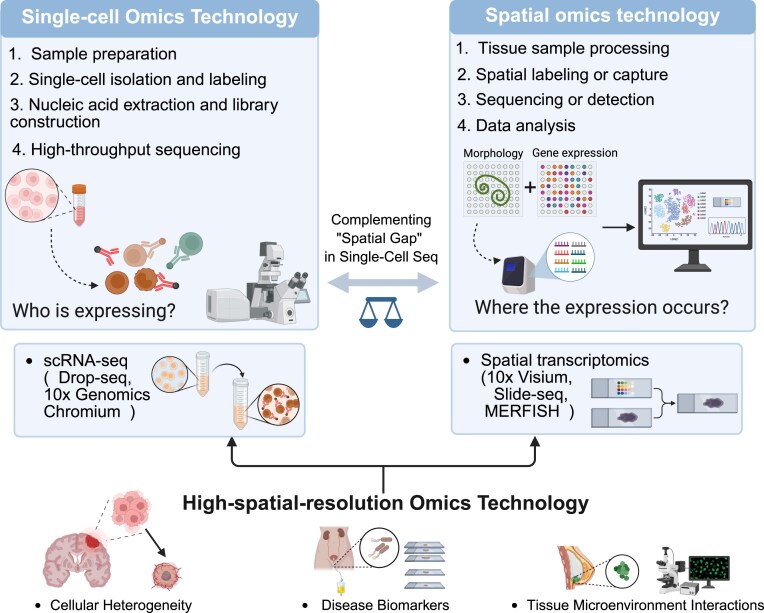
Research workflow and applications of high-spatial-resolution omics technologies: integrated development of single-cell and spatial omics. This figure illustrates the integrated research workflow of high-spatial-resolution omics technologies, combining single-cell omics technology with spatial omics approaches. Single-cell omics involves (i) sample preparation, (ii) single-cell isolation and labeling (utilizing representative technologies such as Drop-seq and 10x Genomics Chromium), (iii) nucleic acid extraction and library construction, and (4) high-throughput sequencing to resolve cellular gene expression profiles and uncover cellular heterogeneity. In parallel, spatial omics employs (i) tissue sample processing, (ii) spatial labeling/capture (via techniques like 10x Visium, Slide-seq, and MERFISH), (iii) sequencing/detection, and (iv) computational data analysis to map gene expression with spatial coordinates, thereby elucidating tissue microenvironment interactions. The synergy between these approaches addresses complementary questions—who is expressing a gene versus where expression occurs spatially—enabling breakthroughs in tumor biomarker identification, cellular subpopulation localization, and tissue developmental mechanisms, among others.

First introduced in 2016 by Ståhl et al. [38] for *in situ* RNA capture, spatial omics technology represents a significant advancement in molecular biology by enabling the profiling of transcriptomic information while preserving spatial context within intact tissue. As illustrated in Fig. 2, unlike single-cell sequencing, which provides molecular resolution but loses spatial information, spatial omics allows the mapping of gene expression, epigenetic modifications, protein localization, and metabolic profiles to precise anatomical coordinates, offering a more comprehensive view of tissue organization and function [39]. The field has developed multiple modalities, including spatial transcriptomics [40], (epi)genomics [41], proteomics [42], and metabolomics [43]. Among these, spatial transcriptomics is the most mature, utilizing either *in situ* hybridization techniques (e.g., MERFISH, seqFISH) for subcellular-resolution transcript mapping or sequencing-based methods (e.g., Visium, Slide-seq) that spatially barcode RNA through microarray capture or hybridization-based imaging [44–46]. These technologies are increasingly being applied in tumor microenvironment characterization, neural development studies, inflammatory disease modeling, and organoid/tissue engineering to uncover spatially regulated gene expression patterns and their functional implications. Despite its potential, spatial omics still faces challenges such as limited resolution, high cost (often exceeding $1,500 per sample), and computational demands in 3-dimensional (3D) spatial data integration. Future directions will focus on improving resolution, reducing cost, enhancing data analysis tools, and promoting multi-omics integration to better elucidate the complex regulatory networks governing tissue biology and disease progression.

## Application of Multi-omics Technologies in the Diagnosis or Treatment of Chronic Neurological Diseases

**Figure 3:**
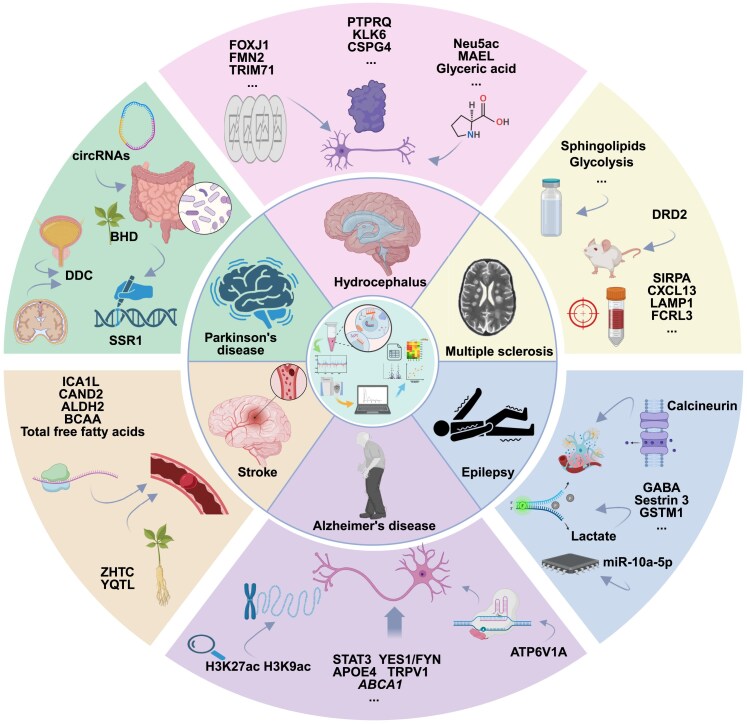
Applications of multi-omics and high-spatial-resolution omics technologies in the diagnosis and treatment of brain diseases in the field of neurology. By integrating the high-throughput omics technologies, including 4 basic omics, single-cell omics, and spatial omics technologies, it is possible to comprehensively dissect the complex pathogenic mechanisms of neurological disorders. This multi-omics approach spans multiple levels, from genetic variations to metabolic changes, and reveals the interactions between these levels, providing an unprecedented perspective for in-depth disease understanding. In the study of diseases such as AD, PD, stroke, epilepsy, MS, and hydrocephalus, the application of these cutting-edge technologies has greatly facilitated the discovery of key biomarkers and significantly deepened our understanding of the molecular mechanisms of disease pathogenesis.

Neurological diseases constitute a major global health challenge, including but not limited to Alzheimer’s disease (AD), Parkinson’s disease (PD), epilepsy, multiple sclerosis (MS), stroke, hydrocephalus, and various neurological diseases [47]. With the rapid advancement of science and technology, multi-omics approaches have become increasingly pivotal in the research of neurological diseases. As shown in Fig. 3, by integrating multilayered data, researchers can delve deeper into the molecular mechanisms underlying these diseases, thereby offering novel perspectives and methods for clinical diagnosis, treatment, and prevention. This review provides a comprehensive overview of the advancements made in the application of multi-omics technologies over the past 5 years in the study of representative neurological diseases.

### Application of multi-omics technologies in AD

AD, commonly referred to as senile dementia, is one of the most prevalent neurodegenerative disorders to date and a leading cause of disability and death among the elderly [48, 49]. The disease typically begins insidiously and progresses irreversibly, leading to severe cognitive impairment [50–52]. However, there is still no effective treatment for AD, and the underlying mechanisms contributing to the pathogenesis of AD remain incompletely elucidated, although amyloid plaques and tau neurofibrillary tangles have revealed major pathological changes in AD [51, 54]. These pathological changes may trigger synaptic dysfunction, neuroglial inflammation, and eventually neuronal loss in the cerebral cortex, subcortical regions, temporal lobe, parietal lobe, and cingulate gyrus [55–57], and they may even affect the gut microbiome [47, 58].

The field of AD intervention has long been confronted with 2 major challenges: first, the pronounced delay in early diagnosis—owing to its insidious onset, most patients are identified only at moderate to severe stages when pathological progression has become largely irreversible [59], resulting in a critical missed window for intervention; second, the incomplete understanding of its pathogenetic mechanisms—although core pathological features such as amyloid plaques and tau neurofibrillary tangles have been identified [52, 54], the molecular regulatory networks underlying subsequent neuronal injury, neuroinflammation, and gut microbiome dysbiosis remain poorly elucidated [55]. Prior to the emergence of multi-omics technologies, these challenges were compounded by methodological limitations. Conventional hypothesis-driven approaches largely focused on isolated pathways such as Aβ deposition or tau phosphorylation [54]. While valuable for elucidating specific pathological components, such strategies failed to capture system-level interactions among gene expression, protein networks, and metabolic alterations. This constrained the discovery of early specific biomarkers and hindered the development of integrated therapeutic strategies targeting multiple pathological processes.

The advent of multi-omics approaches has provided a pivotal toolkit to overcome these limitations [60]. This global perspective transcends the constraints of single-pathway investigations and marks a shift from compartmentalized pathological observation toward systematic mechanistic decoding. It provides a robust foundation for the identification of early intervention targets and the development of precision therapeutics, heralding a new era in AD research and clinical management

#### Basic omics approaches in AD

Basic omics approaches have provided valuable, unique insights into different aspects of AD pathogenesis (Supplementary Table S1). Genomic studies have identified high-risk genes and epigenetic modifications associated with AD, including *APP, PSEN1*, tau, *APOE4*, acetylation status of histone H4 at lysine 16 (*H4K16ac*), and *H3K9ac*. Among these, *APOE4* is particularly well characterized in its association with late-onset AD, as it accelerates vascular dysfunction, disrupts the BBB, and promotes neuronal degeneration. These effects position *APOE4* as a central player in AD’s vascular and neurodegenerative mechanisms, making it a key diagnostic and prognostic marker [61].

Epigenomic analyses have revealed the influence of single-nucleotide polymorphisms (SNPs) in tau and enhancer regions on chromatin structure and disease progression. In particular, *H4K16ac* is significantly altered with aging and AD-related gene expression. Compared to non-AD elderly individuals, 25,000 peaks show loss of *H4K16ac*, whereas 9,000 exhibit increased acetylation in patients with AD. These changes suggest that *H4K16ac* may serve as an epigenetic link between aging and AD pathology, as well as a potential diagnostic biomarker [62]. Similarly, *H3K9ac* and tau have been identified as epigenetic and proteomic biomarkers, with *H3K9ac* showing parallel acetylation patterns in tau-related contexts, indicating a complex interplay between tau pathology and chromatin regulation [63].

Transcriptomic profiling has enabled the identification of coexpression modules that are closely linked to AD pathology. One such module, M109, is most directly associated with cognitive decline and amyloid load. Within this module, *INPPL1* and *PLXNB1* have been identified as potential candidates for *in vitro* amyloid biology investigation. Notably, *INPPL1* and *PLXNB1* are associated with extracellular β-amyloid levels in astrocyte cultures, suggesting their relevance in early detection and mechanistic understanding of AD [64]. This transcriptomic study reveals key molecular connections between brain aging and late-onset AD, demonstrating both shared and distinct gene expression patterns. In the hippocampus and several cortical regions, both conditions show similar alterations in synaptic genes, phosphoproteins, and alternative splicing. However, late-onset AD exhibits unique molecular signatures, including glycoprotein dysregulation, upregulated inflammatory responses, and downregulated myelin sheath and lipoprotein genes. Importantly, these late-onset AD-specific changes appear to progressively accumulate in an “AD-similar” aging subgroup. These findings suggest that early molecular interventions targeting hippocampal and related cortical regions may potentially block the progression from normal aging to late-onset AD, providing novel insights into the transition mechanisms between aging and neurodegeneration at the transcriptomic level [65].

Proteomic studies have uncovered disease-specific alterations in protein expression. For instance, elevated levels of FYN, YES1, and STAT3 in AD-derived induced neurons are associated with neuroinflammation, tau phosphorylation, and increased amyloid-42 production [66]. Proteomics also found that APP/PS1 and APOE4 knock-in mice lead to changes in early hippocampal protein expression profiles and that the changes in hippocampal proteins involved in insulin signaling and the mitochondrial electron transport chain may be key biological processes in AD progression [67]. These findings suggest that proteomics can help distinguish AD subtypes and identify critical pathways in disease progression.

Phosphoproteomic analyses have further highlighted the role of PTMs in AD. Abnormal phosphorylation of GSK3β and PPP3CA in models treated with low-dose copper is linked to mitochondrial dysfunction [68]. A recent study has revealed the staged pathophysiological progression in preclinical autosomal dominant AD via cerebrospinal fluid (CSF) proteome analysis. The 6-protein model (GFAP, NPTX2, PEA15, SMOC1, SMOC2, TNFRSF1B) demonstrates high predictive accuracy (area under the curve [AUC] > 0.9), validated independently, and offers a valuable tool for ultra-early autosomal dominant AD screening, precise staging, and clinical trial enrollment [69].

Metabolomic research has identified sphingolipids as potential early biomarkers of AD, and dysregulated amino acid and tryptophan metabolism have also been observed in transgenic models [70]. Furthermore, some investigators unveiled a metabolic shift toward aerobic glycolysis in AD-derived induced neurons, triggered by pyruvate kinase M2 (PKM2)’s loss of metabolic activity, nuclear translocation, and interaction with *STAT3* and *HIF1α*, which collectively precipitate metabolic and transcriptional changes that underlie neuronal identity loss and heightened vulnerability in sporadic AD [71].

#### High-spatial-resolution omics technologies in AD

High-spatial-resolution omics technologies have transformed the understanding of AD by revealing cell-type–specific and spatially resolved molecular alterations [72]. Mathys et al. [73] pioneered the use of single-cell RNA sequencing (scRNA-seq) in AD, profiling ~80,000 prefrontal cortex cells across disease stages and identifying myelination maintenance as a critical regulatory response, especially the relevant gene *LINGO1*. The experiment also revealed gender differences that exist in AD, including transcriptional response differences and the overrepresentation of females in AD-associated cell subpopulations. Grubman et al. [74] further analyzed the entorhinal cortex, an early AD-affected brain region, and found that astrocytes exhibited distinct activation patterns compared to other areas. Expression changes of multiple AD risk genes (e.g., *APOE, TREM2*) were also validated in specific cell types. For example, *APOE* expression was elevated in microglia and astrocytes, correlating with tau pathology severity, offering cell-specific insights into genetic risk mechanisms.

Spatial transcriptomics has further advanced AD research by preserving spatial context during transcriptome profiling. In AD mouse models, this method detected early transcriptional dysregulation in 100-μm plaque microenvironments, particularly in myelination-related and oligodendrocyte gene networks. Late-stage spatial profiles revealed 57 plaque-induced genes (PIGs) enriched in complement activation, oxidative stress, lysosomal dysfunction, and neuroinflammation [75]. Integration of 10x Genomics Visium spatial transcriptomics with co-immunofluorescence mapping in human middle temporal gyrus identified 5 layer- and white matter–specific marker genes, including both established (*RORB, PCP4, MBP*) and novel (*SPARC, CALB2, DIRAS2, KRT17*) candidates with potential as diagnostic and mechanistic biomarkers [76].

Integrative omics strategies combining single-cell and spatial transcriptomics are beginning to illuminate intercellular communication in AD pathology. A recent study identified a *PTPRG*-expressing microglial subpopulation that promotes neuronal *VIRMA* expression, which enhances m^6^A modification of *PRKN* transcripts, leading to RNA destabilization, impaired mitophagy, and neuronal death. These findings highlight *PTPRG* and *VIRMA* as promising therapeutic targets for AD [77].

Using single-nucleus RNA sequencing (snRNA-seq) and spatial transcriptomic data, a key study addresses the unresolved question of how cellular alterations in AD unfold over time and how these changes can be distinguished from normal brain aging. Using dorsolateral prefrontal cortex (DLPFC) samples from 437 participants in the ROSMAP cohort, a single-cell transcriptomic atlas encompassing 95 cellular subpopulations was constructed. Integration of the BEYOND algorithm with causal modeling identified 2 distinct cellular trajectories: a progressive AD trajectory, characterized by increasing Aβ and tau burden alongside cognitive decline, and an alternative brain aging trajectory, marked by low pathological burden and relatively stable cognition. Lipid-associated microglia Mic.12 (*CPM*) were closely linked to Aβ accumulation, while Mic.13 (*PTPRG*) mediated tau pathology induced by Aβ. A stress-responsive astrocyte subpopulation, Ast.10 (*SLC38A2*), was implicated in cognitive dysfunction. These findings demonstrate that AD arises from dysregulation across coordinated multicellular communities rather than dysfunction of a single cell type, bridging a critical gap between the cellular mechanisms of AD and brain aging and providing potential targets for therapeutic intervention [78].

Recent studies utilizing multi-omics (ATAC-seq, RNA-seq, and Hi-C) approaches have advanced our understanding of how genetic factors influence chromatin accessibility and, consequently, gene expression in human microglia. In microglia associated with AD, there is noticeable activation of immune and inflammatory pathways, accompanied by changes in chromatin structure. Analyses combining transcriptomic and epigenomic data have identified *SPI1/PU.1* as a crucial regulator of gene expression in these cells, with reductions in *PU.1* binding leading to decreased chromatin accessibility. This positions *SPI1/PU.1* as a central player in the dysfunction of microglia related to AD and also points to other potential transcription factors involved. Hi-C data demonstrate that open chromatin regions physically interact with their target genes within the 3-dimensional genome. By integrating genetic variation with regulatory elements, the study identifies *KCNN4, FIBP*, and *LRRC25* as potential risk genes, and the analysis shows that alleles associated with reduced expression of all 3 are linked to increased AD risk. These findings provide insight into how genetic differences can influence gene regulation specific to glial cells, offering a deeper understanding of the mechanisms underlying AD risk [79]. Through multi-omics analyses (snRNA-seq, snATAC-seq) conducted on 92 human prefrontal cortex samples, researchers successfully constructed a cell-type–specific regulatory atlas of the human brain and an AD-associated regulatory network. This study demonstrated that AD genetic risk loci are significantly enriched in the enhancer regions of microglia, with a strong association with the binding sites of transcription factors *SPI1, ELF2*, and *RUNX1*. Concurrently, by integrating 9,628 previously identified cell-type–specific ATAC quantitative trait loci and peak-to-gene linkage data, the research further deciphered the variant regulatory pathways involved in AD. Regarding disease progression–related features, regulatory abnormalities in early-stage AD are primarily concentrated in neurons, whereas late-stage AD is characterized by prominent regulatory dysregulation in glial cells. Additionally, late-stage AD exhibits global epigenomic erosion, manifested as reduced chromatin accessibility, increased heterochromatin, impaired nuclear architecture, and decreased Lamin-B1 expression—findings that suggest widespread loss of cellular identity. This integrated research resource provides critical support for the prioritization of AD-causing variants. However, future studies should incorporate histone chromatin immunoprecipitation sequencing technology to enable more refined characterization of chromatin states [80].

A recent study leveraged single-cell epigenomics and spatial genomics to characterize cell populations in AD, thereby defining 2 distinct disease stages. The early stage is distinguished by an expansion of inflammatory microglia and reactive astrocytes, coupled with the loss of SST⁺ inhibitory neurons and the gradual pathological accumulation associated with remyelination. In contrast, the late stage is hallmarked by elevated pathological indices—specifically increased levels of Aβ and pTau—and the depletion of both excitatory neurons and Pvalb⁺/Vip⁺ inhibitory neurons. To ensure the robustness of these findings, researchers conducted rigorous intrasample validation and further corroborated the results through cross-cohort analyses across 10 publicly available snRNA-seq datasets. Beyond elucidating AD pathogenesis, this work also demonstrates that the integration of multi-omics technologies with quantitative neuropathology enables effective modeling of disease progression aligned with the severity of attention-deficit disorders, underscoring the translational value of such interdisciplinary approaches [81].

One study used snRNA-seq alongside spatial transcriptomics to investigate key molecular features of highly penetrant autosomal dominant AD. Their findings revealed that, compared to sporadic AD cases, autosomal dominant AD cases showed a significant increase in the expression of autophagy-related and chaperone genes. Spatial transcriptomic analyses further confirmed the specific activation of chaperone-mediated autophagy pathways in carriers of the PSEN1-E280A mutation. Notably, this mutation was associated with cell-type–specific activation of autophagy and chaperone pathways in astrocytes and neurons, which may reflect a compensatory mechanism to maintain protein homeostasis in the presence of the mutation. In autosomal dominant AD cases, the team also observed elevated *LRP1* expression in astrocytes, increased *FKBP1B* levels, and decreased *PSEN1* expression in neurons. Intriguingly, the study further noted that individuals homozygous for the APOE3-Christchurch variant showed analogous molecular patterns. By uncovering these genotype-specific molecular profiles, this study advances a more precise and mechanistic understanding of how distinct AD-associated genotypes shape disease-related cellular phenotypes [82].

#### Multi-omics integration in AD

Multi-omics integration has enabled the identification of disease-specific molecular modules and biomarker signatures that reflect the underlying pathophysiology of AD [83] (Supplementary Table S1). For example, combined proteomic and transcriptomic analyses have revealed 2 distinct AD-related modules: the *MAPK*/metabolic module, which is associated with the rate of cognitive decline, and the matrix body (matrixsome) module, whose expression is modulated by the *APOE ε4* allele [84]. Further integration of proteomic and transcriptomic data has identified *FBP1, FBP2, RHOH, JPH2, ERAP2*, and *SCLT1* as upregulated proteins in *APOE4* carriers compared to controls. In contrast, the myeloid basic protein encoding gene (*MBP*) is among the top-ranked candidate genes that reinforce the importance of myelination in AD pathogenesis and cognitive decline. Notably, these biomarkers show consistent expression patterns in both plasma and brain tissue [85]. A multi-omics approach integrating genomics, transcriptomics, proteomics, and metabolomics has identified *ABCA1, CPT1A*, adiponectin, and *NGAL* as key players in the regulation of acylcarnitines and amino acid metabolism. Disruptions in the homeostasis of short-chain acylcarnitines and essential amino acids are closely correlated with disease severity, suggesting that these molecules may serve as early diagnostic indicators [86]. In addition, *IVD, CYFIP1*, and *ADD2* have been identified as serum-based diagnostic biomarkers through proteomic and transcriptomic analysis. *IVD* shows significantly higher protein abundance in patients with AD, whereas *CYFIP1* and *ADD2* are downregulated. The combination of these 3 proteins improves early detection and disease discrimination [87]. From a lysosomal perspective, multi-omics integration has revealed that *CSTD, CTSB, CTSD*, and *GM2A* are significantly upregulated in patients with AD and have been validated as CSF and plasma biomarkers. These lysosomal proteins show progressive fold changes during AD progression, further supporting their diagnostic utility [88]. Moreover, *PBXIP1* has been identified as a multi-omics-linked diagnostic target through genomics, transcriptomics, and proteomics. *PBXIP1*-encoded proteins are significantly associated with all 3 neuropathological hallmarks of AD-Aβ plaques, neurofibrillary tangles, and neurodegeneration. It functions in hippocampal neurons, astrocytes, and mTOR signaling, linking it to both neuropathology and cognitive dysfunction [89]. Epigenomic and proteomic integration has further revealed *H3K27ac* as a potential diagnostic marker, particularly in the entorhinal cortex. Analysis has shown that AD risk variants are significantly enriched in *H3K27ac* peak regions, including *CR1, GPR22, KMO, PIM3, PSEN1*, and *RGCC* [90]. Additionally, integrative studies using transcriptomics, proteomics, and epigenomics have demonstrated that *H3K27ac* and *H3K9ac*, transcriptionally active post-PTMs, are genome-wide dysregulated in AD, with RNA-seq revealing increased expression of histone acetyltransferases. These modifications are enriched in Aβ and tau-related pathways, and their dysregulation contributes to transcriptional and chromatin dysfunction [91]. These epigenetic alterations may serve as early indicators of AD-related gene expression and chromatin dysregulation. Pathway-level integration of proteomics, metabolomics, and lipidomics has identified gender-dependent effects in GABA synthesis, arginine biosynthesis, and alanine/aspartate/glutamate/arginine metabolism. These findings emphasize the importance of lysophospholipid and amino acid metabolism in the AD brain and suggest the need for gender-specific diagnostic models [92]. Finally, 4 CSF proteins—14-3-3 zeta/delta, clusterin, interleukin 15, and transgelin 2—have been shown to enhance AD prediction accuracy through multi-omics integration [93].

From a therapeutic perspective, multi-omics integration has revealed potential drug targets and intervention points (Supplementary Table S1). One such target is *TRPV1*, a pharmacologically modifiable receptor that, when activated, rescues memory deficits and neuronal loss in *APOE4* mice on a high-fat diet. These results highlight the potential of *TRPV1*-based therapies in mitigating AD pathology [94]. In late-onset AD, *ATP6V1A* has been identified as a key regulator of the most dysregulated neuronal subnetwork. Targeting *ATP6V1A* has shown therapeutic potential, as *NCH-51*, a compound targeting this pathway, ameliorates neuronal damage in *Drosophila* models [95].

Several computational platforms have been developed to facilitate the integration and interpretation of multi-omics data in AD. For instance, the Alzheimer’s Disease Genome-Wide Positioning Systems (AlzGPS) platform is used for drug discovery by mining AD-related targets and clinically relevant candidate drugs [96].

Despite advancements in multi-omics technologies, AD research remains heavily reliant on classic biomarkers—Aβ and tau, which continue to dominate both mechanistic and clinical frameworks. While omics approaches have identified numerous candidate biomarkers and pathways, few have been rigorously validated in large, diverse cohorts or translated into clinically usable tools. A major persistent challenge is the fragmented integration of multi-omics data: genomic, proteomic, and metabolomic layers are often studied in isolation rather than as interconnected networks. This lack of systematic integration limits our ability to uncover actionable biological mechanisms or subtype-specific signatures, hindering the development of personalized diagnostic and therapeutic strategies.

**Figure 4:**
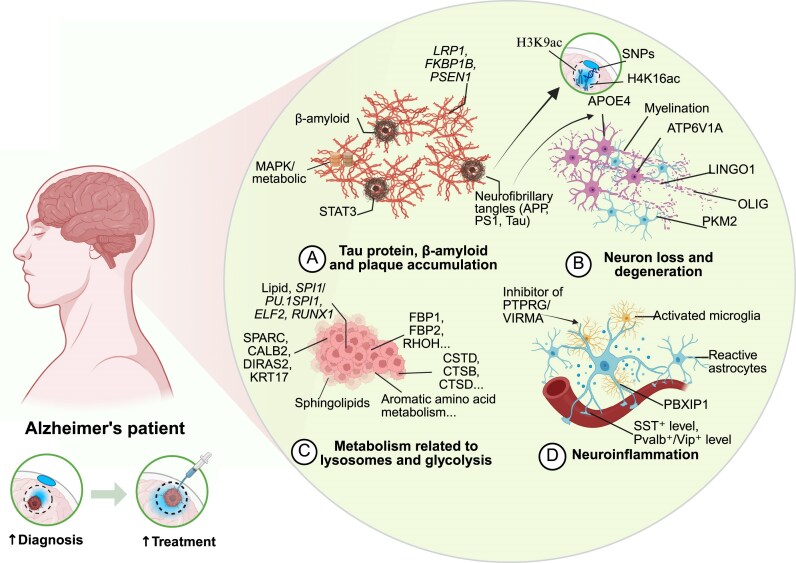
Pathogenesis of AD revealed by multi-omics and high-spatial-resolution omics technologies. This figure provides an overview of the pathogenesis of AD revealed by omics technologies, with a particular focus on several key aspects, including abnormal tau protein, deposition of Aβ protein, formation of neurofibrillary tangles, neuronal loss and degeneration, disorders of lysosomal-related metabolic pathways, and neuroinflammation. In the context of tau protein, Aβ protein, and plaque accumulation, the M7 *MAPK* module and *STAT3* gene are involved. Moreover, *LRP1, FKBP1B, PSEN1, APP, PS1*, and tau proteins constitute the main components of neurofibrillary tangles, and their abnormal alterations represent crucial pathological features of AD. The process of neuronal loss and degeneration involves changes in various key elements, including genes (*APOE4, ATP6V1A, LINGO1, OLIG*), *histone modifications (H3K9ac, H4K16ac)*, metabolite regulation (PKM2), and myelination-related alterations. These factors act in concert, leading to the impairment of neuronal functions and the decline of cognitive abilities. In the lysosome- and glycolysis-related metabolic pathways, the abnormalities of FBP1, FBP2, RHOH, lipid, SPI1/PU.1SPI1, ELF2, CSTD, SPARC, CALB2, and CTSB, *RUNX1*, as well as the metabolic pathways of sphingolipids and aromatic amino acids, reflect cellular metabolic impairments observed in AD. In terms of neuroinflammation, *PBXIP1*, along with activated microglia and astrocytes, shows abnormal hyperactivity. *PTPRG/VIRMA* inhibitors show their impacts on mitochondrial function and neuronal survival, offering potential therapies for AD. Alterations in SST⁺ and Pvalb⁺/Vip⁺ levels also show potential for the diagnosis of AD. By integrating multilevel data across the genome, transcriptome, proteome, and metabolome, multi-omics and high-spatial-resolution omics technologies provide a comprehensive view of AD pathogenesis, offering crucial insights for early diagnosis and precision therapy.

Taken together, these studies underscore the importance of integrating multi-omics data to elucidate the associations between brain functional and structural changes related to AD. The integration of multiple omics layers provides a comprehensive and mechanistically grounded view of the disease, offering a solid theoretical foundation for the development of precision diagnostics and targeted therapies. A detailed diagram illustrating the multi-omics-driven pathogenesis of AD is shown in Fig. 4.

### Application of multi-omics technologies in PD

PD is the second most common chronic neurodegenerative disorder globally, following AD, affecting approximately 1% to 2% of the population. It is characterized by resting tremor, increased muscle tone (rigidity or stiffness), bradykinesia (slowness of movement), and postural instability, with numerous patients also experiencing cognitive impairments or dementia [4, 97, 98]. The disease primarily impacts the motor system [99, 100] and often has an insidious onset, with early diagnosis lacking distinct features [47]. The BBB is disrupted in PD disease, and leukocytes and neutrophils enter the brain and release large amounts of inflammatory cytokines, such as tumor necrosis factor α (TNF-α), interleukin 1β (IL-1β), and interleukin 6 (IL-6) [101]. The accumulation of α-synuclein in Lewy bodies and Lewy neurites, predominantly in the substantia nigra, leads to the loss of dopaminergic neurons and the manifestation of prominent symptoms. Lewy bodies may also be associated with AD, complicating the determination of PD’s etiology and pathogenesis. At present, PD diagnosis depends mainly on clinical evaluation and neuroimaging, but these methods are subject to diagnostic delays and misclassification, especially in the early stages.

A central unresolved question in PD research is how to accurately define disease subtypes and identify early molecular events prior to overt neurodegeneration. Without multi-omics approaches, studies were often limited to clinical phenotypes and single molecular layers, failing to capture the systemic interactions among genetic variants, transcriptional dysregulation, protein misfolding, and metabolic dysfunction. This narrow view hindered the discovery of predictive biomarkers and subtype-specific therapeutic targets, particularly at presymptomatic stages. The application of integrated genomics, transcriptomics, proteomics, and metabolomics has transformed this paradigm. These technologies enable high-resolution mapping of endophenotypes and dynamic pathways, revealing novel biomarkers, such as *GPNMB* and DDC, and unmasking critical mechanisms like neuroinflammation, oxidative stress, and mitochondrial impairment. Multi-omics thus provides a holistic, mechanistic framework for early diagnosis, patient stratification, and targeted interventions in PD.

#### Basic omics approaches in PD

Basic omics approaches have yielded a range of candidate biomarkers and pathogenic insights in PD (Supplementary Table S2). Genomic studies have identified several high-risk loci and genes associated with PD, including leucine-rich repeat kinase 2 (*LRRK2*), *IL1R2, ZNF184, PARK16, ITPKB, HLA, MAPT, TRIM10*, and *SETD1A* [102, 103]. Allelic variations in *LRRK2* and *IL1R2* significantly increase PD risk, while *HLA* and *MAPT* loci are repeatedly implicated in disease susceptibility. Most of these genes are involved in autophagy and lysosomal function–related pathways, which are critical for the clearance of misfolded α-synuclein and other toxic proteins [103].

Transcriptomic profiling has revealed dysregulated gene expression patterns in PD. Signal sequence receptor subunit 1 (*SSR1*), a gene encoding a mitochondrial protein, is upregulated in patients with PD and negatively correlated with dopaminergic neuron survival. Notably, *SSR1* is upregulated in peripheral blood before the onset of motor symptoms, suggesting its potential as an early diagnostic marker. A machine learning (ML)–based random forest (RF) classifier incorporating *SSR1* expression achieves a high diagnostic accuracy (AUC = 0.91), reinforcing its value in PD detection [104].

Proteomic studies have identified several disease-associated proteins and inflammatory signatures in PD. OMD, CD44, VGF, PRL, and MAN2B1 show significant expression changes in patients with PD and are strongly correlated with clinical scores, including motor severity and disease progression [105]. In addition, LRRK2 carriers exhibit enhanced neuroinflammatory profiles, further supporting its pathogenic and diagnostic relevance. These proteins are considered potential biomarkers for PD diagnosis.

Metabolomic analyses have revealed widespread metabolic dysregulation in PD, particularly in lipid and energy metabolism. Alterations in carnitine shuttle, sphingolipid metabolism, arachidonic acid metabolism, and fatty acid biosynthesis are consistently observed, with carnitine shuttle activity being most significantly perturbed in unmedicated patients with PD [106].

Additionally, short-chain fatty acids, including butyric acid, are significantly reduced and correlated with cognitive decline and motor dysfunction [107]. A comprehensive urinary metabolomic profile has identified 139 differentially regulated metabolites, with proline among the most predictive [108]. Moreover, phenylacetic acid, phenylacetylglutamine, histidine, uric acid, and imidazoleacetic acid are consistently upregulated in urine and show strong diagnostic power in early-stage PD, with a 45-metabolite model achieving high accuracy [109]. Using the same technique, this team also found that 18 differential metabolites changed metabolic pathways related to branched-chain amino acid (BCAA) metabolism, glycine derivatives, steroid hormone biosynthesis, tryptophan metabolism, and phenylalanine metabolism, confirming the team’s earlier findings [110].

#### High-spatial-resolution omics technologies in PD

Smajić et al. [111] employed snRNA-seq and GWAS to profile midbrain cell contributions, identifying a PD-specific neuronal cluster overexpressing *CADPS2* and showing reduced tyrosine hydroxylase levels. Glial populations in affected regions exhibited disease-restricted proliferation, along with dysregulation in unfolded protein response and cytokine signaling pathways, while reactive astrocytes showed *CD44* upregulation and microglia displayed a proinflammatory trajectory marked by elevated *IL-1B, GPNMB*, and *HSP90AA1*. These results position *IL-1B, GPNMB*, and *HSP90AA1* as possible diagnostic biomarkers for PD [111]. A recent scRNA-seq study integrated with gene set enrichment analysis provided the first single-cell resolution gene atlas of the dorsolateral prefrontal cortex in PD, revealing transcriptomic changes across microglia, astrocytes, oligodendrocytes, and oligodendrocyte precursor cells. Key findings include mitochondrial dysfunction, immune dysregulation, and impaired protein folding in glial cells, underscoring their roles in disease progression. This experiment shows that using *HSP90* inhibitors can speed up the breakdown of inflammasomes, thus lowering inflammatory responses and easing neurodegeneration. This finding opens up novel possibilities for the selection of immunotherapeutic approaches [112].

While dopaminergic neuron loss in the substantia nigra pars compacta (SNpc) is a defining pathological hallmark of PD, the molecular basis of their selective vulnerability remains unclear. Integrating single-cell genomics and slide-seq spatial transcriptomics, Kamath et al.[113] identified a distinct *AGTR1*-expressing neuronal subtype spatially confined to the SNpc ventral tier that exhibits heightened PD susceptibility. This vulnerable subtype demonstrated significant upregulation of *TP53* and *NR2F2* target genes, implicating these transcription factors in degenerative processes, and displayed pronounced transcriptomic alterations in stress pathways regulated by *TP53/NR2F2*, directly linked to PD-associated neuronal death. Combining single-cell transcriptomic and proteomics, Zhu et al. [114] revealed an inverse correlation between α-synuclein pathology and chaperone expression in excitatory neurons, concurrent with attenuated neuron–astrocyte interactions and exacerbated neuroinflammation in PD. ScRNA-seq analysis demonstrated *SYN2* enrichment in PD brains, indicating significantly enhanced synaptic signaling at both transcriptional and proteomic levels, providing novel insights into coordinated molecular dysregulation.

#### Multi-omics integration in PD

In the diagnostic process of PD, multi-omics technologies have achieved remarkable success by precisely identifying a series of key biomarkers and pathways (Supplementary Table S2). At the protein and transcript levels, *GPNMB, CD38*, and *DGKQ* have been identified as candidate diagnostic biomarkers through integrative proteomic and transcriptomic analysis. These proteins show significant associations with PD risk, and their expression is supported by quantitative trait locus analysis and fine mapping [115]. Furthermore, DDC is consistently upregulated in the CSF, blood, and urine of patients with PD and is strongly correlated with symptom severity [116]. DDC and related proteins are thus considered viable targets for accurate PD diagnosis.

From a therapeutic perspective, the integration of transcriptomics and metabolomics has revealed that Buyang Huanwu decoction (BHD) exerts therapeutic effects in PD by modulating key metabolic pathways, including the relaxin signaling pathway, adhesion patch, and PI3K-Akt signaling pathway, and by reducing disease-related symptoms. In preclinical models, BHD treatment enhances dopaminergic neuron survival and improves motor function, indicating its potential for neuroprotection and functional recovery [117]. In addition, genomic and metabolomic studies have identified circular RNA *CircSV2b* as a potential therapeutic target in PD. *CircSV2b* is significantly deregulated in PD mouse models compared to wild-type controls, and its overexpression via the ceRNA–Akt1 axis has been shown to mitigate oxidative stress, a central pathological mechanism in PD [118].

Despite advances in multi-omics research, the field of PD continues to grapple with 3 fundamental challenges: clinical heterogeneity (e.g., differential treatment responses between tremor-dominant and postural instability/gait difficulty subtypes), poorly understood mechanisms of α-synuclein propagation (particularly via proposed gut-to-brain pathways through the vagus nerve), and a persistent lack of disease-modifying therapies. Future studies should prioritize deeply phenotyped longitudinal cohorts—incorporating real-time motor monitoring via wearable sensors and comprehensive autonomic function assessments—alongside the integration of single-cell sequencing and spatial transcriptomics to elucidate region-specific neurodegeneration in the substantia nigra. The development of advanced computational models (e.g., multimodal data fusion and Mendelian randomization) will be essential to infer causal pathways from high-dimensional data. By focusing on prodromal cohorts and currently underrepresented populations, researchers can systematically unravel neuroimmune-metabolic cross-talk, ultimately enabling the discovery of early biomarkers, patient stratification, and personalized therapeutic strategies.

**Figure 5:**
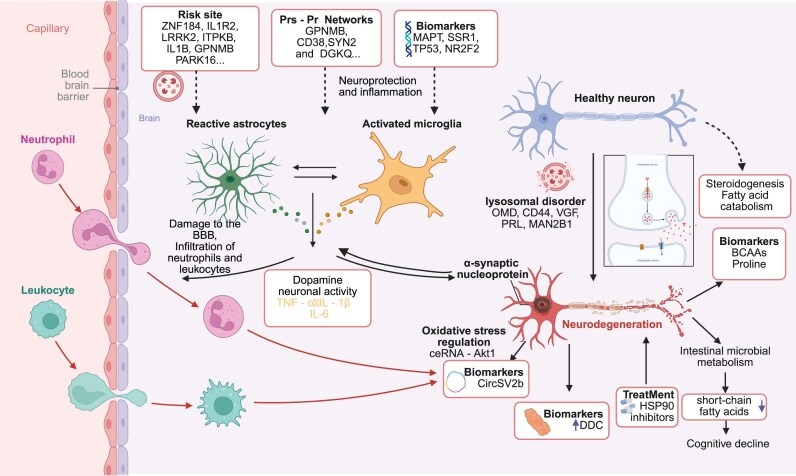
Multi-omics and high-spatial-resolution omics reveal key mechanisms, biomarkers, and risk factors in PD. PD is a complex neurodegenerative disorder influenced by genetic, environmental, and neurobiological factors. Mutations in genes such as *ZNF184, IL1R2, IL1B, GPNMB*, and *LRRK2* are central to PD risk prediction and genetic susceptibility. Aberrant expression of proteins, like *GPNMB, CD38, SYN2*, and *DGKQ*, and biomarkers, such as *MAPT, SSR1, TP53*, and *NR2F2*, is linked to PD progression and diagnostic value. These changes drive neuroinflammation and immune activation. BBB disruption facilitates the infiltration of leukocytes and neutrophils, initiating neuroinflammation. Activated microglia and astrocytes release inflammatory cytokines (e.g., TNF-α, IL-1β, IL-6), which exacerbate the inflammatory environment and damage neurons. Lysosomal dysfunction, involving OMD, CD44, VGF, PRL, and MAN2B1, and ceRNA–Akt1 axis disruption contribute to α-synuclein aggregation. Inflammatory biomarkers, including CircSV2b, DDC, Proline, BCAAs, and molecules in steroidogenesis and fatty acid catabolism, are also identified. Dysregulated short-chain fatty acid metabolism is associated with cognitive decline in PD. *HSP90* inhibitors show therapeutic potential, as suggested by scRNA-seq of neuronal heterogeneity and molecular pathways. This figure integrates multilevel omics data to illuminate PD pathogenesis, supporting early diagnosis, monitoring, and targeted treatment.

Taken together, these integrative approaches not only enhance our understanding of PD heterogeneity but also pave the way for the development of precision diagnostics and personalized therapies. A schematic overview of the multi-omics-driven pathogenesis and therapeutic mechanisms in PD is presented in Fig. 5.

### Application of multi-omics technologies in epilepsy

Epilepsy is the second most common neurological disorder, affecting approximately 3–6% of pediatric neurological cases and occurring across the entire life span [119]. It is associated with recurrent abnormal neuronal discharges in the central nervous system (CNS), leading to episodic dysfunction in motor, sensory, autonomic, and cognitive domains, as well as contributing to cognitive, psychological, and social impairments [120, 121]. The etiology and pathogenesis of epilepsy are highly heterogeneous, involving genetic, metabolic, and inflammatory factors. Over the past decade, omics technologies—including metabolomics, proteomics, and transcriptomics—have provided critical insights into the molecular underpinnings of epilepsy, although the precise mechanisms remain incompletely understood.

A fundamental challenge in epilepsy research is how to decipher the complex, multi-dimensional interactions among genetic predisposition, neuronal excitability, inflammatory processes, and metabolic dysregulation that collectively contribute to seizure generation and progression. Prior to the advent of multi-omics technologies, research was often siloed into isolated domains—electrophysiology, candidate genes, or histopathology—which provided fragmentary insights but failed to capture the systemic and dynamic nature of epileptogenesis. This limitation hindered the identification of convergent pathways and translatable biomarkers, especially across different epilepsy subtypes and developmental stages.

#### Basic-omics approaches in epilepsy

Basic omics technologies have played a crucial role in identifying key molecular alterations in epilepsy across different biological layers (Supplementary Table S3). Transcriptomic studies have identified dysregulated gene expression in various epilepsy subtypes. In generalized epilepsy, *P38MAPK, JAK STAT, PI3K*, and *MTOR* signaling pathways are consistently upregulated and show stable regulation in affected patients [122]. In temporal lobe epilepsy (TLE), hub genes such as *TLR2, LGALS3, SERPINE1*, and *STAT3* are positively correlated with seizure frequency and are associated with microglial/macrophage activation, extracellular matrix (ECM) remodeling, cell motility, and immune responses [123]. These findings suggest that transcriptomics can provide mechanistic insights into seizure onset and progression.

By integrating analyses of gray matter volume with transcriptomic data from the human brain atlas, 1 study analyzed brain morphology and possible underlying mechanisms in patients with unilateral TLE with hippocampal sclerosis, who were divided into 2 groups: divided into focal to bilateral tonic–clonic seizure (FBTCS^+^) and (FBTCS^−^). Structural MRI revealed gray matter volume atrophy in both cortical and subcortical regions in FBTCS^+^ patients, while FBTCS^−^ patients showed localized atrophy. Imaging transcriptomics was employed to link gray matter volume changes to gene expression, and both groups involved in the study exhibited anomalies related to synaptic function and MAPK signaling. FBTCS^−^ genes were involved with processes of both excitatory and inhibitory neurons, while FBTCS^+^ genes were involved with only excitatory neurons. GABAergic neuron damage may lead to excitatory/inhibitory imbalance and FBTCS in FBTCS^+^ patients. The new findings could have major implications for TLE pathogenesis and potentially help in diagnosis and treatment [124]. One study, based on a GABRA4 knockout (GABRA4⁻⁄⁻) mouse model and using methods including RNA-seq omics analysis, behavioral analysis, network analysis, and electrophysiological recording, revealed that GABRA4⁻⁄⁻ mice exhibited autism-like phenotypes while showing enhanced spatial memory and reduced seizure susceptibility, and differentially expressed genes in the hippocampus were enriched in autism spectrum disorder (ASD)– and synapse-related pathways. Network analysis further indicated that ASD-, epilepsy-, and memory-associated subnetworks converged on a regulatory module centered on the NMDA receptor (NMDAR) system, in which Grin1 served as a key upregulated node. These findings laid an experimental foundation for the development of cross-disease therapeutic strategies targeting the simultaneous improvement of symptoms related to ASD, memory impairment, and epilepsy [125]. One study on TLE, based on [¹⁸F]SynVesT-1 PET (targeting SV2A) and 2 transcriptome datasets, and using synaptic density similarity network (SDSN) topological analysis, spatial correlation investigation, gene enrichment, and genetic interaction analysis, revealed that patients with TLE had reduced SDSN strength/clustering coefficient and increased path length (temporo-limbic/frontoparietal distribution, indicating connectivity loss and reorganization), that SDSN changes correlated with TLE risk gene expression and gene dysregulation, that 183 downregulated genes enriched in synaptic pathways formed a connected network (GABAergic genes such as *SLITRK3*and *RBFOX1* as core nodes), and that these findings first linked downregulated risk gene spatial patterns to TLE synaptic density network dysfunction [126]. Proteomic investigations have revealed abnormal protein expression patterns in both diagnostic and therapeutic contexts. In TLE, glial fibrillary acidic protein (GFAP) is consistently downregulated in brain tissue with a high spike frequency and shows a strong negative correlation with seizure severity, indicating its role in reactive astrocyte function and neuroprotection [127]. In the hippocampal region of epileptic brains, 144 differentially expressed proteins, such as ADP-ribosyl cyclase (ADPRC), lysophosphatidic acid receptor 3 (LPAR3), calreticulin, ubiquitin carboxyl-terminal hydrolase L1 (UCH-L1), synaptosome-associated protein 25 (SNAP-25), and transgelin 3, have been identified, with most related to Ca²⁺ homeostasis. Notably, inhibition of calcium influx has been shown to alleviate seizures, supporting the relevance of ion channel and signaling protein targets [128]. Additionally, tutin induces epilepsy and causes significant neurological damage by activating calcineurin, a key phosphatase involved in seizure initiation and progression [129].

Metabolomic analyses have uncovered distinct metabolic signatures in epilepsy. In pediatric epilepsy, N-acetyl glycoprotein, lactate, creatine, glycine, and lipids are elevated, while citrate levels are reduced, suggesting their potential as diagnostic biomarkers [130]. In mesial temporal lobe epilepsy (MTLE), γ-aminobutyric acid (GABA) is significantly upregulated in the epileptogenic zone of KA-MTLE mice and is considered a specific metabolic marker for MTLE [131]. These findings indicate that metabolomics can reveal early-stage metabolic perturbations and pathophysiological changes in epilepsy.

#### High-spatial-resolution omics technologies in epilepsy

High-spatial-resolution omics technologies have enabled the identification of dysfunctional neuronal subtypes associated with epileptic seizure activity, particularly in the human temporal cortex. SnRNA-seq of over 110,000 neurons revealed the most significant transcriptomic alterations in principal neuron subtypes, including *L5-6_FEZF2* and *L2-3_CUX2*, as well as *GABAergic* interneurons marked by *SST* and *PVALB* gene expression. Among the most profoundly dysregulated pathways is glutamatergic signaling, particularly through the upregulation of genes encoding AMPA receptor auxiliary subunits in *SST* and *PVALB* subtypes. These results highlight the central role of *GABAergic* interneurons in early epileptogenesis and their potential as diagnostic targets [132]. Comparative scRNA-seq studies between posttraumatic epilepsy (PTE) and hereditary epilepsy revealed distinct cellular landscapes. Hereditary epilepsy samples exhibited increased abundance of oligodendrocytes and astrocytes but reduced microglia and neuronal populations compared to PTE. Within microglia and astrocytes, the IL-17 signaling pathway emerged as a potential therapeutic and biomarker candidate, pointing to immune and inflammatory mechanisms as critical contributors to neuronal dysfunction in PTE. Notably, *XIST*, a long noncoding RNA associated with inflammatory cell infiltration, fibrosis, and satellite glial cell activation, was significantly upregulated in PTE, suggesting its role in epileptogenic progression and early detection [133].

To further uncover the molecular basis of hippocampal sclerosis and hyperexcitability in TLE, a multi-omics strategy combined scRNA-seq, snRNA-seq, and Xenium spatial transcriptomics. The spatial data highlighted glia-specific gene upregulation and neuron-specific downregulation, with key dysregulated genes including *SPP1* and *TREM2* (upregulated in glia) and *TLE4* and *SIPA1L3* (downregulated in neurons), suggesting their diagnostic and mechanistic relevance in TLE [134].

#### Multi-omics integration in epilepsy

In epilepsy diagnosis, multi-omics research has identified several key targets for precise interventions to deepen our understanding of the origins of epilepsy (Supplementary Table S3).

Integrated genomics and transcriptomics have revealed the key role of *SESN3* in the pro-convulsant gene network of the human hippocampus in epilepsy. *Sestrin 3* positively regulates modules in macrophages, microglia, and neurons, and it is considered a potential diagnostic marker [135]. Integrative proteomic and transcriptomic analyses have revealed the involvement of the transforming growth factor β (*TGF-β*) signaling pathway in cardiac dysfunction associated with epilepsy. Within this pathway, *STAT3, ERBB*, and *MAPK8* emerge as key regulators of cardiac alterations induced by seizure activity [136]. Integration of proteomics and metabolomics has identified glutathione S-transferase M1 (GSTM1) and aldehyde dehydrogenase 2 (ALDH2) as protein hubs in the somatosensory cortex and thalamus, respectively [137]. Furthermore, genomic and metabolomic integration has identified lactate, creatine, phosphocreatine, and choline as metabolic markers with distinct expression patterns in epilepsy. Lactate is significantly reduced, whereas creatine, phosphocreatine, and choline are markedly elevated, reflecting metabolic reprogramming associated with seizure activity [138].

In epilepsy, although the focus of multi-omics research has largely been on pathogenesis and diagnosis, relatively few studies have explored the therapeutic potential of these approaches. Integrative proteomic and transcriptomic analyses have uncovered *miR-10a-5p, miR-21a-5p*, and *miR-142a-5p* as important microRNA transcripts associated with epileptogenesis, primarily through the *TGF-β* signaling pathway. Notably, anti-miR therapies targeting these miRNAs have shown protective effects in both acute and spontaneous seizure models, underscoring their potential for therapeutic application in modulating seizure activity and signaling networks [139].

The pathogenesis of epilepsy is highly complex, involving multilayered biological processes. Different types of epilepsy may exhibit distinct etiologies and mechanisms, complicating both research and therapeutic development. Furthermore, significant clinical heterogeneity among patients adds another layer of difficulty to studying the disease. Multi-omics studies in epilepsy generate vast amounts of data across genomic, transcriptomic, epigenomic, and other molecular levels. These insights extend beyond pathogenesis to offer novel diagnostic biomarkers—such as*miR-10a-5p* and *ALDH2*—and reveal new therapeutic targets, exemplifying how multi-omics bridges mechanistic discovery and precision medicine in epilepsy. Integrating and analyzing these datasets require high-performance computing resources as well as systematic and statistically robust analytical frameworks and mathematical models. A major ongoing challenge lies in the development of efficient computational strategies to process and interpret these complex, high-dimensional data.

In summary, basic omics and multi-omics approaches have significantly contributed to elucidating the molecular complexity of epilepsy. These technologies have identified critical genes, proteins, and metabolites that are linked to seizure activity, neuroinflammation, and metabolic dysregulation. Although most research has focused on diagnostic and mechanistic exploration, the therapeutic potential of multi-omics-based strategies is increasingly being recognized.

### Application of multi-omics technologies in MS

MS is a chronic, immune-mediated, inflammatory demyelinating disease of the CNS, marked by temporal and spatial lesion dissemination [140, 141]. While the exact etiology remains unclear, risk factors such as Epstein-Barr virus infection, low vitamin D levels, limited sunlight exposure, smoking, and high adolescent body mass index have been linked to MS development [142, 143]. Clinical symptoms are diverse, including visual impairment, paresthesia, muscle weakness, and progressive disability [144]. A major feature is the presence of oligoclonal bands (OCBs) in CSF, indicating CNS inflammation. Although OCBs are a classical biomarker for MS, their lack of specificity—as they appear in other CNS inflammatory diseases—limits their diagnostic utility [145]. A major challenge in MS research is understanding how immune, inflammatory, and degenerative processes interact across disease stages. Traditional methods offered limited insight into system-wide changes, hindering biomarker discovery and targeted therapy development. The complex and heterogeneous mechanisms of MS remain incompletely understood, impeding targeted therapeutic development. Multi-omics technologies offer a comprehensive approach to uncover molecular underpinnings and identify novel biomarkers and therapeutic targets. Multi-omics approaches have revolutionized MS research by integrating spatial, single-cell, and molecular data. These technologies reveal key cell subtypes, lesion-specific biomarkers, and critical pathways such as *APOE-TREM2* and *CXCL12-CXCR4*. These advances are paving the way for improved stratification mechanisms and precision medicine interventions in MS.

#### Basic omics approaches in MS

Basic omics studies have made substantial contributions to the diagnostic and therapeutic landscape of MS. A large-cohort study based on GWAS data from 20,831 patients with MS and 729,220 control participants, using genetic locus mapping, functional annotation, neuronal/glial cell-type enrichment analysis, and cross-ancestry replication, revealed the identification of 4 novel MS-associated genetic loci, along with the key finding that the expression of *IL7* and *STAT3*—genes previously linked to immune and inflammatory processes—was specifically altered only in inhibitory neuron subtypes; these results further highlight the critical importance of both neuronal and glial dysfunction in driving MS susceptibility, extending prior understanding of the disease’s genetic and cellular underpinnings [146]. In proteomic profiling, several proteins have been identified as potential biomarkers, including CXCL13, LTA, FCN2, ICAM3, LY9, SLAMF7, TYMP, CHI3L1, FYB1, TNFRSF1B, and NFL. Notably, lower levels of NFL in CSF show predictive potential for disease activity (AUC = 0.77) [147]. In the metabolomic domain, dopamine receptor D2 (DRD2) has been shown to exacerbate MS by promoting inflammation and reducing *Lactobacillus* abundance in the gut microbiome. Conversely, *Lactobacillus*-derived N2-acetyl-L-lysine has anti-neurodegenerative effects by inhibiting microglial activation [148]. In relapsing-remitting multiple sclerosis (RRMS), metabolomics has identified 4 dysregulated metabolic pathways, with glycolysis serving as a common upstream driver. Targeting glycolysis in experimental autoimmune encephalomyelitis ameliorated the disease pathology by impeding immune cell effector function [149].

#### High-spatial-resolution omics technologies in MS

High-spatial-resolution and single-cell omics technologies have provided unprecedented insights into cellular and molecular heterogeneity in MS, particularly in active lesions and periplaque regions involving both CNS and peripheral immune compartments. Single-cell transcriptomic analysis of CSF has revealed a compartmentalized immune landscape, with enrichment of myeloid dendritic cells and regulatory T cells. A notable finding is the cluster-independent expansion of T follicular helper (TFH) cells, which is associated with increased B-lineage cell infiltration into the CNS and worsened disease severity in MS animal models. These results underscore the local T/B-cell crosstalk as a critical driver of MS pathology [150].

Spatial transcriptomics profiling techniques, such as *in situ* sequencing (10x Xenium) and scRNA-seq, have enabled the spatial mapping of active MS lesion evolution. Astrocytes were categorized into 3 distinct functional states: homeostatic, intermediate, and disease-associated. Disease-associated astrocytes showed marked upregulation of *SERPINA3*, a gene strongly correlated with active lesion areas in MS. This expression pattern may reflect a glioprotective response aimed at resolving inflammation and preventing apoptosis during early and late lesion resolution phases, and *SERPINA3* has been proposed as a spatial biomarker for MS pathology [151].

Integration of scRNA-seq and spatial transcriptomics has identified perturbations in *KLF/SP* regulatory drivers in oligodendrocytes, including enhanced iron uptake, expression of proinflammatory molecules near axonal injury sites, and the role of *MAFB*, an inflammatory transcription factor, as a hallmark of MS lesions [152]. These studies highlight the complex interplay between complement factors, apolipoproteins, and immune cells, as well as a distinct *APOE–TREM2* axis involved in lesion repair.

Multi-omics integration—combining spatial transcriptomics (10x Visium), scRNA-seq, and spatial proteomics (imaging mass cytometry)—has uncovered the fibrogenic niche in systemic MS skin lesions, driven by a dynamic fibroblast–macrophage axis through the *ACKR3–CXCL12–CXCR4* signaling pathway. Pharmacological inhibition of *CXCR4* using AMD3100 significantly reduced dermal and pulmonary fibrosis, as well as myofibroblast accumulation, in preclinical models, validating this pathway as a potential therapeutic target. Importantly, the markedly elevated *POSTN/SCARA5* ratio in MS lesions exhibits strong potential as a predictive diagnostic biomarker for disease progression [153].

#### Multi-omics integration in MS

Multi-omics integration has enhanced the comprehensive understanding of MS pathophysiology and expanded the identification of robust biomarkers (Supplementary Table S4). Combined proteomics and transcriptomics have revealed *GPR37L1, SIRPA, FGFR3, CADM3*, and *TYRO3* as highly expressed candidate molecules in the CNS, associated with early neuronal degeneration and impaired trophic and anti-inflammatory intercellular communication, supporting their use as diagnostic tools [154]. Additionally, 24 iron death-related genes (e.g., *CHMP5, SLC38A1, PML*) have been linked to neuroinflammatory processes, where high iron death scores correlate with phagocytic activation at lesion margins and neurological dysfunction in cortical neurons. A blood-based model incorporating these genes has shown prognostic value for MS diagnosis [155]. An integrative approach combining proteomics and metabolomics has identified LAMP1, FCG2A, and heparinase (HPSE) as potential specific biomarkers for MS, with HPSE showing strong correlations with metabolites such as L-tyrosine, sphingosine 1-phosphate, and L-tryptophan [156]. Moreover, another study has also identified reduced levels of anti-inflammatory molecules and sphingolipids, as well as low equine uric acid in severe MS subgroups, pointing to their potential in biomarker development and targeted therapeutic strategies [157].

Multi-omics technologies have systematically uncovered key mechanisms in MS, including dysregulated CD4⁺ T-cell/B-cell crosstalk, impaired *TREM2-DAP12* signaling in microglia, and disrupted differentiation of oligodendrocyte precursor cells. While potential therapeutic targets such as S*EMA4D* and *CXCL13* have been identified, their functional roles in remyelination and immune infiltration require further validation through *in vivo* gene editing and organoid models. Critical challenges remain in predicting the transition from relapsing-remitting to secondary progressive MS, deciphering compartmentalized inflammation within chronic lesions, and elucidating mechanisms of resistance to B-cell–targeted therapies. Future efforts should prioritize longitudinal multi-omic profiling of well-annotated conversion-phase cohorts, integration of digital pathology with real-time wearable sensor data, and application of artificial intelligence—such as graph neural networks and multimodal learning—to uncover clinically actionable biomarkers and enable precision medicine interventions.

Currently, our understanding of the relationship between multi-omics profile alterations in patients with MS and the underlying molecular networks contributing to MS pathogenesis remains limited. One study outlines a potential integrated multi-omics mechanism that may regulate peripheral immune-inflammatory responses and the progression of MS. These findings can facilitate the development of novel auxiliary diagnostic biomarkers and therapeutic strategies for MS. The summary information is shown in Supplementary Table S4.

### Application of multi-omics technologies in stroke

Stroke, or cerebrovascular accident, is the most severe neurological disorder to date, causing approximately 160 million years of healthy life lost annually. It is mainly classified into ischemic stroke (IS, ~87%) and hemorrhagic stroke (HS), resulting from vascular injury and leading to focal or global brain damage. Common symptoms include hemiplegia, facial paralysis, and speech impairment, with severe cases progressing to sudden loss of consciousness. Stroke is characterized by high incidence, disability, and mortality, underscoring the urgent need for improved prevention and treatment strategies. High-throughput technologies now offer new opportunities to unravel its complex pathophysiological mechanisms [158, 159].

A major challenge in stroke research is grasping the spatiotemporal dynamics of molecular and cellular responses to acute injury and recovery. Before multi-omics, studies were confined to single pathways or cell types, missing system-wide interactions across key networks and hindering the discovery of reliable biomarkers and neuroprotective strategies, especially early poststroke. Multi-omics have revolutionized this by enabling high-resolution, integrated profiling of transcriptional, proteomic, and metabolic changes in a cell- and region-specific way. This has revealed key mechanisms, such as microglia–endothelial crosstalk and immune–metabolic axes (e.g., *SPP1–CD44*), and identified novel biomarkers and targets (e.g., *LILRB4, LGALS9*) for stroke treatment.

#### Basic omics approaches in stroke

Basic omics studies have identified key biomarkers and pathways associated with stroke (Supplementary Table S5). Proteomic profiling has revealed elevated levels of NSF, RhoGDI1, RabGDI, CKB, and CMPK in the circulation of IS patients, reflecting neuronal excitotoxicity and energy metabolism disruption [160, 161]. Additionally, SAHH2 increased expression of SAHH2 in neurons from the infarcted area, probably because of ischemia-triggered Ca2^+^ mobilization [162].

Transcriptomic analyses have identified differentially expressed long noncoding RNAs (lncRNAs), such as *MEG3, H19*, and *MALAT1*, and extracellular microRNAs, including *miR-32-3p, miR-106b-5p, miR-423–5p*, and *miR-4739*. These molecules are involved in apoptosis, oxidative stress, angiogenesis, and neurogenesis [163–168]. Through the use of a permanent middle cerebral artery occlusion model, the recent study performed RNA-seq on brain tissues from 3-month-old (young) and 18-month-old (aged) female mice to systematically characterize the molecular mechanisms between aging and ischemic stroke. Both groups of mice exhibited similar transcriptional profiles in the ischemic cortex; however, the responses of aged mice to ischemia were significantly greater. In particular, the aged brain demonstrated pronounced activation of the type I interferon (*IFN-I*) signaling cascade, accompanied by marked downregulation of genes associated with axonal integrity and synaptic maintenance—especially those defining PV^+^ interneurons—and enhanced infiltration of peripheral leukocytes, notably neutrophils. Single-cell analyses further identified microglia and oligodendrocytes as principal cellular sources of *IFN-I* pathway upregulation in the aged brain. The findings indicate that aging-related neuroinflammation and synaptic vulnerability act synergistically to exacerbate ischemic injury, offering mechanistic insights into stroke pathology in the aging brain and suggesting potential avenues for targeted therapeutic intervention [169].

Metabolomic studies indicate that reduced levels of BCAA are associated with cardioembolic stroke and poor neurological outcomes, highlighting their potential as diagnostic and prognostic biomarkers [170]. Additionally, total plasma free fatty acid levels are significantly elevated in cardioembolic stroke patients compared to those with non-cardioembolic stroke, further suggesting their value as diagnostic targets [171].

#### High-spatial-resolution omics technologies in stroke

High-spatial-resolution omics technologies have revealed critical molecular and cellular mechanisms underlying stroke pathophysiology and recovery, particularly in aging-related neuroinflammation and myelin repair. ScRNA-seq studies have shown that aging impairs paracrine communication between microglia/macrophages and endothelial and oligodendrocyte precursor cells, thereby hindering angiogenesis and remyelination. Transplantation of young microglia/macrophages into aged stroke models partially restores these processes and sensorimotor function, highlighting these cells as potential therapeutic targets for stroke recovery [172].

Modulating *LILRB4* signaling and its downstream effectors emerges as a promising therapeutic approach for IS, as spatial transcriptomics and scRNA-seq have identified a stroke-associated microglial cluster 3 and revealed significant upregulation of *LILRB4* expression in ischemic brain regions. Functional studies show that *LILRB4* deficiency exacerbates ischemic injury through increased CD8^+^ T-cell recruitment, while its overexpression exerts neuroprotective effects, underscoring the therapeutic potential of modulating *LILRB4* and its downstream pathways in mitigating immune-mediated damage [173]. In contrast, Li et al. [174] investigated brainstem stroke and found that oligodendrocyte loss leads to neurological deficits, followed by regenerative attempts. They identified a subcluster of Pros1^+^ oligodendrocytes, termed *OLG8*, and demonstrated that Myo1e overexpression promotes *OLG8* differentiation, reduces ischemic damage, and enhances neurological recovery, offering cell-type–specific therapeutic avenues. Gu et al. [175] further uncovered the dynamic immune landscape in poststroke brains, revealing distinct differentiation patterns between myeloid and lymphoid cell populations and spatially localized interactions. Notably, a *SPP1*-high lymphocyte subcluster was found to interact with Lyz2^+^ macrophage-associated lymphocytes, while in the choroid plexus, Lgmn^+^ macrophage/T-cell communication via the *SPP1–CD44* axis was observed during the acute phase of intracerebral hemorrhage. These findings establish *SPP1* and *Lyz2* as potential biomarkers for stroke diagnostics. Further, they identified ferroptosis as the dominant form of programmed cell death, occurring as early as 1 hour after intracerebral hemorrhage, primarily in mature oligodendrocytes. They showed that a CSF1/CSF1R-mediated interaction between Lipocalin-2^+^ microglia and oligodendrocytes drives ferroptosis and functional deterioration, suggesting that early inhibition of microglial Lipocalin-2 may protect oligodendrocytes and mitigate poststroke deficits, representing a novel neuroprotective strategy [176]. In IS models, Han et al. [177] revealed elevated galectin (*LGAL*) signaling in microglia and macrophages, and they found that *LGALS9* administration promotes oligodendrocyte remyelination and functional recovery, supporting its therapeutic utility in stroke. In subarachnoid hemorrhage (SAH), Wang et al. [178] demonstrated that meningeal lymphatic vessels (mLVs) injury is induced by SAH, with *THBS1* and *S100A6* showing marked upregulation postinjury. They identified the *THBS1–CD47* ligand–receptor axis as a key driver of meningeal lymphatic endothelial apoptosis through *STAT3/BCL2* signaling, suggesting that targeting this axis could preserve mLV integrity and improve clinical outcomes.

A study utilizing spatial transcriptomics and GWAS compared different regions along the hemodynamic direction of human carotid artery plaques and found that plaque rupture primarily occurs in the proximal and most stenotic areas, exhibiting distinct features of inflammation, matrix degradation, and thrombosis. RNA sequencing identified differentially expressed genes distinguishing vulnerable regions from distal sites, and genome-wide association analysis demonstrated that these differentially expressed genes were genetically enriched for traits related to atherosclerosis and stroke risk. Spatial transcriptomics further validated rupture-associated molecular pathways, among which matrix metalloproteinase 9 (*MMP-9*) was highly expressed within rupture zones. Mendelian randomization analysis confirmed a causal relationship between elevated circulating *MMP-9* levels and atherosclerotic risk, offering novel insights into plaque rupture mechanisms and targeted therapeutic approaches [179]. This study employed a cortical ischemic stroke model in male mice, integrating Visium spatial transcriptomics, 10x Chromium single-cell transcriptomics, and the novel spatially resolved single-cell omics platform tDISCO, to systematically examine the spatiotemporal heterogeneity of astrocytic responses following stroke. Results revealed that the acute phase (d2) was characterized predominantly by macrophage-related gene expression, whereas the subacute phase (d10) showed pronounced glial responses, and gene expression in the cortex tended to recover during the chronic phase (d21). The study identified 2 distinct astrocyte populations located proximally and distally to the lesion. Proximal cells enriched for lipid transport and metabolism-related genes (e.g., *APOE, FABP5*), suggesting potential involvement in synaptic remodeling and neuroprotection. Moreover, tDISCO further validated the molecular characteristics of astrocytes residing in different spatial locations, providing new insights for studying glial cell function and precision interventions after stroke [180].

#### Multi-omics integration in stroke

Multi-omics integration has enabled the identification of complex molecular interactions and novel therapeutic targets in stroke (Supplementary Table S5). In cardioembolic stroke (CES), combined proteomic and transcriptomic approaches have uncovered *ICA1L, CAND2*, and *ALDH2* as potential biomarkers related to excitatory synaptic dysfunction [181]. In the therapeutic context, integrated genomics and metabolomics have shown that Zhilong Huoxue Tongyu capsules (ZHTCs), a traditional Chinese medicine, can modulate gut microbiota and metabolic profiles, including arginine, lysine, and methionine, and enhance intestinal barrier integrity [182]. Furthermore, multi-omics and network pharmacology studies on Yiqitongluo granules (YQTLs) have revealed 15 active components that regulate 82 targets across 19 pathways, with *PI3K-Akt, MAPK*, and *cAMP* signaling playing central roles in neuroprotection against cerebral ischemia–reperfusion injury [183].

While multi-omics technologies hold promise for advancing precision medicine in stroke, addressing critical challenges—including pronounced disease heterogeneity, complex repair mechanisms, and difficulties in translating omic findings into clinical applications—remains essential to fully realize their potential. It is crucial to validate biomarkers and therapeutic targets across diverse stroke populations and to develop targeted delivery systems for neuroprotective agents. However, current stroke multi-omics datasets remain limited in scale and diversity, and integrating multimodal data is complicated by missing values, batch effects, and lack of standardization. Future research should prioritize longitudinal multi-omics profiling to capture dynamic disease progression, incorporate single-cell and spatial technologies to resolve cell- and region-specific responses, and leverage artificial intelligence for integrated analysis of multimodal data.

In summary, basic omics studies have provided critical insights into the biomarkers and pathophysiological mechanisms of stroke, particularly in IS and cardioembolic stroke. Proteomic, transcriptomic, and metabolomic approaches have identified key proteins, noncoding RNAs, and metabolic alterations associated with disease onset, progression, and outcomes. Meanwhile, multi-omics integration has further revealed molecular networks and therapeutic targets, demonstrating the potential of systems biology in advancing stroke diagnosis and treatment.

### Application of multi-omics technologies in hydrocephalus

Hydrocephalus is a potentially fatal neurologic disorder affecting individuals across the life span [184]. It is characterized by the abnormal accumulation of CSF due to disruption of its circulation, resulting in ventricular dilation and frequently elevated intracranial pressure (ICP) [185]. Based on CSF flow dynamics, the disease is classified into 3 main types: obstructive, communicating, and normal-pressure hydrocephalus (NPH) [186]. Despite modest progress in surgical techniques over the past 5 decades, preventive and curative strategies remain limited. Current diagnostic methods—Hakim’s triad, CT and MRI, lumbar puncture, and lumbar drainage—are subjective, invasive, and lack specificity, especially in differentiating NPH from vascular or AD-related dementias, due to incomplete understanding of its pathogenesis [184, 187]. Likewise, pharmacological therapies have not produced effective treatment options so far [188–190].

Multi-omics technologies integrate genomic, proteomic, and metabolomic data to systematically dissect molecular networks underlying hydrocephalus, enabling the discovery of novel biomarkers such as *CSPG4* to improve early diagnosis and dynamic monitoring of disease progression. These approaches further uncover central pathophysiological mechanisms—including choroid plexus inflammation and ciliary dysfunction—and identify potential therapeutic targets within dysregulated signaling pathways (e.g., TGF-β, VEGF) and metabolic perturbations, thereby providing new avenues for developing precision interventions.

In this context, multi-omics technologies are emerging as promising tools for elucidating the molecular mechanisms, identifying novel biomarkers, and developing targeted, noninvasive therapeutic strategies for hydrocephalus.

#### Basic omics approaches in hydrocephalus

Basic omics studies have identified key molecular signatures associated with the pathogenesis and clinical manifestations of hydrocephalus, particularly communicating hydrocephalus (CH) and idiopathic normal-pressure hydrocephalus (iNPH) (Supplementary Table S6). Genomics in CH has revealed *TRIM71* and *SMARCC1* as genes with genome-wide significant *de novo* mutations, potentially serving as genetic risk factors. Additionally, *PIK3CA, PTEN, MTOR, FOXJ1, FMN2, PTCH1*, and *FXYD2* have been identified as high-confidence sporadic CH-associated genes, with *TRIM71* deletion linked to reduced neural cell proliferation, making it a potential diagnostic marker [191].

Proteomic profiling has identified kallikrein 6 (KLK6) as significantly upregulated in patients with CH, implicating it in disease progression and suggesting its utility in diagnostic strategies [192]. In iNPH, 39 upregulated and 285 downregulated proteins in CSF have been observed, with elevated glutaminyl-peptide cyclotransferase (QPCT) and retinol-binding protein 4 (RBP4) levels showing prognostic and diagnostic relevance for shunt response [193]. Notably, Q-type protein tyrosine phosphatase receptor (PTPRQ) is significantly higher in iNPH compared to AD and may help distinguish iNPH from AD-related dementia [194].

Metabolomic studies in iNPH and NPH have uncovered CSF metabolic profiles that aid in differential diagnosis and treatment response prediction. In iNPH, elevated glyceric acid and N-acetylneuraminic acid (Neu5Ac), along with reduced serine and 2-hydroxybutyric acid, form a diagnostic signature that differentiates it from AD [195]. In NPH, low CSF Neu5Ac levels are associated with astrocyte activation and periventricular demyelination. Elevating brain Neu5Ac has been shown to improve neurological outcomes, indicating its therapeutic potential [196].

#### High-spatial-resolution omics technologies in hydrocephalus

A recent study utilizing snRNA-seq and spatial transcriptomics in a tumor-associated hydrocephalus (TAH) mouse model revealed the expansion of choroid plexus mast cells (*CPMCs*) in the ventricular region, including the choroid plexus and ependymal walls. These *CPMCs* contribute to TAH pathogenesis by disrupting ciliated epithelial cells through the tryptase–PAR2–FOXJ1 signaling axis, thereby enhancing CSF production. Importantly, elevated CSF tryptase levels have been associated with increased clinical severity of TAH, and administration of the brain-penetrant tryptase inhibitor *BMS-262,084* significantly attenuated TAH *in vivo* and protected against ciliary damage in human stem cell–derived choroid plexus organoids. These results identify *CPMCs* as key drivers of TAH and highlight *BMS-262,084* as a potential therapeutic candidate [197].

By integrating genomics and scRNA-seq approaches, 1 study systematically characterized the evolutionary, temporal, and spatial expression profiles of maelstrom spermatogenic transposon silencer (*MAEL*), a PIWI-interacting RNA pathway component, during human brain development, with functional validation confirming its expression in hydrocephalic human brain tissues. ScRNA-seq analyses of the cortical plate and germinal zone reveal robust *MAEL* expression in neural progenitor niches, with low homology observed in model organisms. In the later stages of brain development, *MAEL* is preferentially enriched in glial progenitor cells and excitatory neurons. A reduction in *MAEL* expression may trigger extensive genomic rearrangements, disrupting cortical development, volume, and function, consistent with previous transcriptome-wide association studies (TWAS) findings. These results suggest that decreased *MAEL* expression may contribute to the pathogenesis of hydrocephalus through multiple etiological pathways [198].

#### Multi-omics integration in hydrocephalus

Multi-omics integration has enabled a systems-level understanding of hydrocephalus and the identification of novel diagnostic and therapeutic biomarkers through cross-omics validation and functional annotation (Supplementary Table S6). In response, multi-omics joint analysis has emerged as a crucial method for revealing therapeutic targets and refining diagnostic methods by integrating and comprehensively analyzing data from different omics layers (Supplementary Table S6). Integrated genomics, proteomics, and transcriptomics in CH have highlighted the maelstrom *MAEL* as a candidate diagnostic biomarker. Reduced *MAEL* expression in multiple brain regions is significantly associated with hydrocephalus, and PrediXcan analysis confirms its pathophysiological relevance [199]. In posthemorrhagic hydrocephalus, the combined use of proteomics and metabolomics has identified chondroitin sulfate proteoglycan 4 (CSPG4) as a promising CSF biomarker. CSPG4 is positively correlated with ventricular size and the incidence of periventricular leukomalacia. Functional studies indicated that CSPG4 silencing can inhibit ferroptosis, cell adhesion, and intracellular Ca²⁺ flux, supporting its role in both diagnosis and treatment [200].

However, multi-omics studies in hydrocephalus face considerable challenges due to the relative difficulty in obtaining CSF samples, which are often limited in volume. Furthermore, the low abundance of proteins and metabolites in CSF necessitates highly sensitive detection technologies. Preanalytical variables during sample collection, processing, and storage can also significantly affect data accuracy and reproducibility. Critically, the scarcity of these precious samples has resulted in a notable paucity of high-quality omics datasets in hydrocephalus, making existing data exceptionally valuable. Overcoming these barriers will require intensified research efforts, standardized protocols, and enhanced support from funding agencies and policymakers to facilitate larger-scale sample acquisition and multi-institutional collaboration.

Although multi-omics approaches have unveiled a plethora of candidate targets and pathways for hydrocephalus therapy, definitive clinical breakthroughs in therapeutic development remain elusive. These findings not only enhance our comprehension of the pathological processes of hydrocephalus but also lay a theoretical foundation for the future development of targeted therapies directed at specific pathways or molecules.

## Current Challenges and Future Trends

Although multi-omics technologies have advanced our understanding of neurological disease pathophysiology, their full clinical translation is hindered by significant technical, analytical, and translational challenges. These are particularly evident in high-spatial-resolution omics approaches, such as scRNA-seq and spatial transcriptomics, which provide cell-type–specific and spatially resolved insights but also introduce additional layers of complexity in data generation, integration, and interpretation.

### Data complexity in multi-omics and high-spatial-resolution omics integration

Omics data are inherently high-dimensional, heterogeneous, and multimodal, posing significant challenges in standardization, integration, and biological interpretation. The lack of universally accepted data formats and protocols across institutions leads to inconsistent data quality and poor interoperability, especially when integrating genomics, transcriptomics, and proteomics, which vary in dynamic ranges and measurement scales [201]. Technical variability in sample preparation and instrument performance further introduces noise, missing values, and batch effects, necessitating robust preprocessing strategies, such as missing value imputation and outlier detection, to ensure data reliability [202–204].

High-spatial-resolution omics technologies, such as scRNA-seq and spatial transcriptomics, exacerbate these challenges by capturing cell-type–specific and spatially resolved molecular profiles. These approaches generate ultra-high-spatial-resolution datasets with substantial cellular and spatial heterogeneity, requiring advanced computational pipelines for clustering, trajectory inference, and cell-type annotation. Spatial transcriptomics, in particular, introduces spatial coordinates, thereby demanding novel algorithms that can integrate molecular and spatial information simultaneously [205]. The absence of standardized annotation systems and centralized data repositories for high-spatial-resolution data remains a major bottleneck, especially in complex brain regions such as the cortex, hippocampus, and choroid plexus, where cellular diversity and spatial architecture are particularly pronounced [206].

Given these challenges, the emergence of artificial intelligence (AI), particularly ML and deep learning, has opened new avenues for multi-omics data integration. ML algorithms can extract key features and identify underlying patterns from high-dimensional and heterogeneous datasets, offering critical support for biomarker discovery and mechanistic elucidation [207]. For instance, DL-based approaches have demonstrated remarkable success in early AD diagnosis by analyzing integrated multi-omics and network data, significantly improving diagnostic accuracy [208]. Moreover, AI-driven multi-omics analysis not only enhances the understanding of neurodegenerative disease mechanisms but also accelerates the identification of novel druggable targets and supports the development of disease-specific biomarkers, ultimately improving treatment outcomes [209].

### Challenges in biomarker validation and clinical translation

The validation of neurological disease biomarkers remains a critical translational hurdle, particularly in the context of multi-omics and high-spatial-resolution omics. Traditional bulk omics methods often average out cellular and interindividual heterogeneity, thereby obscuring biologically relevant signals that are cell-type or region specific. In contrast, scRNA-seq and spatial transcriptomics provide detailed molecular profiles at the cellular and spatial levels, yet their technical complexity and data variability pose significant challenges for robust clinical validation. A major limitation is the lack of standardized, reproducible, and scalable validation frameworks that can retain the resolution and biological context of high-dimensional and multimodal data. Most existing protocols are optimized for bulk-level analysis, which fails to preserve the cellular and spatial information essential for identifying cell-type– or region-specific biomarkers. As a result, many high-spatial-resolution biomarkers may not be detectable or reproducible in standard clinical assays, where resolution is lower and biological noise is higher. For instance, S100B, a protein detectable in stroke patients, lacks sufficient specificity due to its expression in healthy individuals and in other neurological conditions [210]. This underscores the need for more rigorous validation strategies that account for interindividual variability driven by genetic, epigenetic, and environmental factors. Such variability can mask or distort molecular signatures, requiring large-scale, well-phenotyped, and multi-omics-annotated cohorts for robust biomarker discovery and cross-population validation. Furthermore, the clinical translation of multi-omics findings is hindered by limited clinician engagement and inadequate integration with clinical workflows.

To overcome these barriers, a comprehensive and standardized validation framework is essential. This framework should incorporate high-spatial-resolution experimental validation, multi-omics data harmonization, and clinically annotated reference datasets to ensure reproducibility and generalizability across diverse populations and clinical settings. Moreover, collaboration across disciplines—including computational biology, neurology, and bioinformatics—is necessary to align research findings with clinical needs and to develop practical, scalable solutions for the translation of high-spatial-resolution omics biomarkers into routine clinical diagnostics and personalized treatment strategies.

### Multi-modal integration of multi-omics with clinical imaging and real-time sensing

A key future direction in neurological disease research lies in the integration of multi-omics and high-spatial-resolution omics data with clinical imaging and real-time sensing technologies. This multimodal strategy enables the bridging of molecular insights with anatomical and functional information, thereby enhancing mechanistic understanding at multiple biological scales. For example, in the research of AD, by utilizing MRI to obtain brain structural images and integrating metabolomics data, it becomes possible to identify abnormal metabolite changes in specific brain regions [211]. Such integrative frameworks are critical for connecting molecular heterogeneity with clinical phenotypes, improving diagnostic accuracy, and enabling personalized therapeutic interventions.

Despite its promise, this approach faces several technical and methodological challenges. First, the heterogeneous nature of omics, imaging, and real-time sensor data complicates data alignment and integration. Second, standardized protocols for multimodal validation are still in early development, which limits reproducibility and clinical translation. Third, real-time data from wearable biosensors, while offering continuous monitoring and high temporal resolution, pose new computational demands when fused with static omics data, requiring novel methods for dynamic profiling and predictive modeling.

Moving forward, the development of unified platforms that support seamless integration of multi-omics, imaging, and real-time sensing will be essential for advancing precision neurology. These platforms should incorporate AI-driven analysis, adaptive data fusion strategies, and clinically validated biomarker pipelines to facilitate early detection, individualized treatment planning, and longitudinal disease tracking.

## Conclusion

Neurological diseases, characterized by their high incidence, high disability rate, and severe impact on patients’ quality of life, have emerged as a significant global health challenge that urgently demands solutions. While traditional methods have laid the groundwork for understanding disease mechanisms, the integration of multi-omics and high-spatial-resolution omics technologies now provides comprehensive and spatially resolved molecular insights. Studies on major neurological disorders, including AD, PD, MS, stroke, and hydrocephalus, have identified numerous disease-associated genes, proteins, metabolites, and pathways. These discoveries deepen our understanding of pathogenesis, highlight key drivers of disease progression, and offer potential biomarkers and therapeutic targets for early detection, precision treatment, and prognosis.

Despite these advances, the application of multi-omics and high-spatial-resolution omics in this field remains challenging, particularly in data integration, standardization of validation protocols, and translation to clinical settings. Addressing these issues is critical for harnessing the full potential of these technologies in neurological disease research. Looking ahead, the continued development of open-source platforms will enhance the adoption and utility of multi-omics and high-spatial-resolution omics approaches. This progress is expected to accelerate mechanistic discovery, improve diagnostic accuracy, and support individualized therapies, ultimately advancing precision neurology and translational medicine.

## Supplementary Material

giaf137_Supplemental_File

giaf137_Authors_Response_To_Reviewer_Comments_Original_Submission

giaf137_Authors_Response_To_Reviewer_Comments_Revision_1

giaf137_GIGA-D-25-00354_Original_Submission

giaf137_GIGA-D-25-00354_Revision_1

giaf137_GIGA-D-25-00354_Revision_2

giaf137_Reviewer_1_Report_Original_Submission Chuan Xu -- 9/15/2025

giaf137_Reviewer_1_Report_Revision_1 Chuan Xu -- 9/24/2025

giaf137_Reviewer_2_Report_Original_Submission Lu Zeng -- 9/15/2025

giaf137_Reviewer_2_Report_Revision_1 Lu Zeng -- 10/6/2025

giaf137_Reviewer_2_Report_Revision_2 Lu Zeng -- 10/21/2025

## Data Availability

Not applicable.

## Additional Files

**Supplementary Table S1**. Application of multi-omics and high-spatial-resolution omics technologies in AD.

**Supplementary Table S2**. Application of multi-omics and high-spatial-resolution omics technologies in PD.

**Supplementary Table S3**. Application of multi-omics and high-spatial-resolution omics technologies in epilepsy.

**Supplementary Table S4**. Application of multi-omics and high-spatial-resolution omics technologies in MS.

**Supplementary Table S5**. Application of multi-omics and high-spatial-resolution omics technologies in stroke.

**Supplementary Table S6**. Application of multi-omics and high-spatial-resolution omics technologies in hydrocephalus.

## Abbreviations

AD: Alzheimer’s disease; ADPRC: ADP-ribosyl cyclase; AI: artificial intelligence; ALDH2: aldehyde dehydrogenase 2; AlzGPS: Alzheimer’s Disease Genome-Wide Positioning Systems; ASD: autism spectrum disorder; ATAC-seq: assay for transposase-accessible chromatin using sequencing; AUC: area under the curve; BBB: blood–brain barrier; BCAA: branched-chain amino acid; BHD: Buyang huanwu decoction; CH: communicating hydrocephalus; CNS: central nervous system; *CPMC*: choroid plexus mast cell; CSF: cerebrospinal fluid; CSPG4: chondroitin sulfate proteoglycan 4; CT: computed tomography; DDA: data-dependent acquisition; DIA/SWATH: data-independent acquisition/sequential window acquisition of all-theoretical mass spectral approach; DLPFC: dorsolateral prefrontal cortex; DRD2: domain, dopamine receptor D2; FBTCS: focal to bilateral tonic–clonic seizures; FT-IR: Fourier transform–infrared; GABA: γ-aminobutyric acid; GC-MS: gas chromatography–mass spectrometry; GFAP: glial fibrillary acidic protein; GSTM1: glutathione S-transferase M1; GWAS: genome-wide association studies; *H4K16ac*: acetylation status of histone H4 at lysine 16; HPSE: heparinase; HS: hemorrhagic stroke; ICP: intracranial pressure; *IFN-I*: type I interferon; IL-1β: interleukin 1β; IL-6: interleukin 6; iNPH: idiopathic normal-pressure hydrocephalus; IS: ischemic stroke; iTRAQ: isobaric tags for relative and absolute quantification; KLK6: kallikrein 6; LC-MS: liquid chromatography–mass spectrometry; lncRNA: long noncoding RNA; LPAR3: lysophosphatidic acid receptor 3; LRRK2: leucine-rich repeat kinase 2; MAEL: maelstrom spermatogenic transposon silencer; TWAS: transcriptome-wide association studies; *MBP*: myeloid basic protein encoding gene; ML: machine learning; MMP-9: matrix metalloproteinase 9; CES: cardioembolic stroke; MRI: magnetic resonance imaging; MS: multiple sclerosis; MTLE: mesial temporal lobe epilepsy; Neu5Ac: N-acetylneuraminic acid; NMDA: N-methyl-D-aspartate; NMDAR: NMDA receptor; NMR: nuclear magnetic resonance; NPH: normal-pressure hydrocephalus; OCB: oligoclonal band; PD: Parkinson’s disease; PIG: plaque-induced gene; PTE: posttraumatic epilepsy; PTM: posttranslational modification; PTPRQ: Q-type protein tyrosine phosphatase receptor; QPCT: glutaminyl-peptide cyclotransferase; RBP4: retinol-binding protein 4; RF: random forest; RNA-seq: RNA sequencing; RRMS: relapsing-remitting multiple sclerosis; SAH: subarachnoid hemorrhage; mLVs: meningeal lymphatic vessels; scRNA-seq: single-cell RNA sequencing; SDSN: synaptic density similarity network; SILAC: stable-isotope labeling by amino acids in cell culture; SNAP-25: synaptosome-associated protein 25; SNP: single-nucleotide polymorphism; SNpc: substantia nigra pars compacta; snRNA-seq: single-nucleus RNA sequencing; *SSR1*: signal sequence receptor subunit 1; TAH: tumor-associated hydrocephalus; TFH: T follicular helper; *TGF-β*: transforming growth factor β; TLE: temporal lobe epilepsy; ECM: extracellular matrix; TMT: tandem mass tag; *TNF-α*: tumor necrosis factor α; UCH-L1: calreticulin, ubiquitin carboxyl-terminal hydrolase L1; YQTL: Yiqitongluo granule; ZHTC: Zhilong Huoxue Tongyu capsule.
