# Supplementary material for: Multi-omics and high-spatial-resolution omics: deciphering complexity in neurological disorders
Source: Gigascience. 2025 Dec 5;14:giaf137. doi: 10.1093/gigascience/giaf137 (PMC12723665; doi:10.1093/gigascience/giaf137)
Supplement: giaf137_Supplemental_File [file giaf137_supplemental_file.docx]

**Table 1: Overview of the Four Omics Technologies.**

| Technology | Precision | Price Range | Advantages | Disadvantages | Disease Application Representation |
| --- | --- | --- | --- | --- | --- |
| Genomics | High | High | - Conduct a comprehensive analysis of genetic sequences. - Uncover the depth of genetic variations. - Be suitable for gene discovery and genetic disease studies. | - High experimental costs. - Complex techniques and large sample sizes. - Considerable time in analysis. | - Genetic disorders. - Cancer genomics. - Genetic counseling. |
| Transcriptomics | Medium | Medium | - Reveal dynamic changes in gene expression. - Differentiate gene regulatory networks. - Assist in the classification of disease subtypes. | - High experimental and data analysis design. - Limited real-time to reflect mRNA levels. | - Mental disorders. - Cardiovascular diseases. - Prognostication and efficacy assessment in cancer. |
| Proteomics | Low | Medium | - Reflect protein levels and modifications directly. - Reveal protein-protein interaction networks. - Explore changes in protein function. | - Complex data analysis with low standardization. - Effects of PTMs. | - Development of tumor biomarkers. - Mechanistic studies in autoimmune diseases. - Pathology of neurodegenerative diseases. |
| Metabolomics | Medium | Medium | - Provide the overall metabolic profile of the organism. - Reflect metabolic changes associated with disease. - Assist in early diagnosis and monitoring. | - Sensitive sample handling and storage conditions. - Challenges in the detection of metabolites. | - Monitoring of endocrine disorders. - Prediction of cardiovascular disease risk. - Metabolic testing in diabetes. |
| Single-cell Omics | High | High | - Resolve cellular heterogeneity (tumor subclones). - Identify rare cell types (<0.1% population). - Enable multi-omics integration (ATAC+RNA). | - Spatial information loss from tissue dissociation - Significant technical noise (dropout rate >15%) - Single-cell amplification bias | - Tumor evolutionary tree construction. - T-cell receptor clonal tracking. - Nervous diseases neuronal subtyping. |
| Spatial Omics | High | High | - Preserve in-situ spatial topology. - Quantify cell-cell interactions (immune synapses). - Directly correlate pathological morphology with molecular expression. | - Resolution inversely proportional to throughput (e.g., MERFISH: ~1,000 genes). - Optical diffraction limitations (>200nm). - High complexity in multidimensional data integration. | - Tumor immune exclusion zone mapping. - Brain region-specific protein gradient atlases. - Myocardial infarction spatial injury demarcation. |

PTMs: post-translational modifications.

**Table 2: Comparative Analysis of Five Mainstream Proteomics Techniques.**

| Technology | Introduction | Advantages | Disadvantages | Labeling  Groups | Data  Volume | Cost |
| --- | --- | --- | --- | --- | --- | --- |
| iTRAQ | Employ chemical labels to identify proteins. | - Simultaneous analysis of up to 8 groups. - Processing of multiple samples. - Enhanced throughput. | - Expensive reagents. - Complex experimental procedures. | 4 or 8 | Medium | High |
| TMT | Utilize chemical labeling to identify proteins. | - Simultaneous analysis of up to 10 or 11 groups. - Simultaneous processing of more samples. - Higher sensitivity. | - Expensive reagents. - Complex experimental operations. | 10 or 11 | Medium | High |
| SILAC | Introduce isotope-labeled amino acids into the culture media. | - Accurate quantification. - High sensitivity. | - Requirement for cell culture. - Unsuitability for clinical samples. | 2 or 3 | Low | Medium |
| Label-free | Detect endogenous peptides without labeling. | - No need for labeling. - Simple sample preparation. - Cost-effective. | - Reduced reproducibility. - Slightly diminished sensitivity. | Unlimited | High | Low |
| DIA/SWATH | Obtain mass spectrometry data through a full scan. | - No labeling required. - Simultaneous quantification of numerous proteins. - Good reproducibility. | - Complex data analysis. - Need for specialized software. | Unlimited | High | Medium |

iTRAQ: isobaric tags for relative and absolute quantification; TMT: tandem mass tag technology; SILAC: stable-isotope labeling by amino acids in cell culture; DIA: data-independent acquisition; SWATH: sequential window acquisition of all theoretical mass spectral approach.

**Supplementary Table S1:** Application of Multi-omics and High-spatial-resolution Omics Technologies in AD.

| Disease | Omics Type | Purpose | Biomarker | Relevance to AD Pathogenesis | Experiment Validation | Reference |
| --- | --- | --- | --- | --- | --- | --- |
| AD | Genomics | Diagnosis | *APOE4* | - *APOE4* accelerates vascular dysfunction, BBB rupture, and neuronal degeneration. - *APOE4* is pivotal in AD's vascular and neurodegenerative pathogenesis and serves as a marker. | N | Montagne A et al.[61] |
| AD | Genomics | Diagnosis | *H4K16ac* | - Compared to non-AD elderly participants, 25,000 peaks showed *H4K16ac* loss, while 9,000 showed increased *H4K16ac* in AD individuals. - *H4K16ac* decreases with aging or AD-related gene sites. - *H4K16ac* set the stage for an epigenetic link between aging and AD. - *H4K16ac* can be a diagnostic marker for AD disease. | N | Nativio R et al.[62] |
| AD | Genomics | Diagnosis | *H3K9ac*,  TAU protein | - TAU, whereas non-amyloid β pathology has a broad impact on histone acetylation in AD brain. - *H3K9ac* structural domain shows similar gain or loss of TAU-related histone acetylation. - Complex interactions between TAU and chromatin structure. - *H3K9ac* and TAU are biomarkers for AD. | Y (Three Independent Datasets) | Klein HU et al.[63] |
| AD | Transcriptomics | Diagnosis | *INPPL1, PLXNB1* | - The M109 module is the one most directly associated with cognitive decline and amyloid load. - *INPPL1* and *PLXNB1* are associated with extracellular β-amyloid levels in astrocyte cultures. - *INPPL1* and *PLXNB1* are interesting candidates for AD. | Y (Two Independent Datasets) | Mostafavi S et al.[64] |
| AD | Transcriptomics | Diagnosis | Myelination | - Neuroinflammation pathways are significantly upregulated in AD. - Genes for myelination and lipid metabolism are downregulated in AD. - Novel treatments for AD can focus on promoting myelin repair. | Y (Three Independent Datasets) | [Shouneng Peng](https://pubmed.ncbi.nlm.nih.gov/?term=Peng+S&cauthor_id=34924992) et al.[65] |
| AD | Proteomics | Treatment | STAT3*,* YES1 and FYN | - STAT3*,* YES1*,* and FYN reduce neuroinflammation, TAU phosphorylation, and endogenous production of amyloid-42. - Drugs targeting the cytokine transducer STAT3 and the Src family tyrosine kinases, YES1 and FYN, rescued molecular phenotypes relevant to AD pathogenesis. - STAT3, YES1, and FYN can be used as drug targets for the treatment. | Y (Three Independent Datasets) | Jackson A et al.[66] |
| AD | Proteomics | Treatment | Insulin signaling and mitochondrial electron transport chain | - Changes in hippocampal protein expression profiles in APP/PS1 and E4 knockout mice. - Different expression proteins in both mouse models, participate in insulin signaling and the mitochondrial electron transport chain. - Preserving mitochondrial function and boosting insulin signaling could aid in improving cognitive function for AD patients. | N | He K et al.[67] |
| AD | Proteomics | Diagnosis /Treatment | The phosphorylation levels of GSK3β and Ppp3ca*,* GSK3β, Ppp3ca | - The phosphorylation levels of GSK3β and Ppp3ca are closely associated with mitochondrial biogenesis. - Low-dose oral copper treatment changes the phosphorylation of key hippocampal proteins involved in mitochondrial, synaptic and axonal integrity. - The phosphorylation levels of GSK3β and Ppp3ca are potential diagnostic and therapeutic targets. | N | Chen C et al.[68] |
| AD  (autosomal dominant) | Proteomics | Diagnosis | GFAP*,* NPTX2, PEA15, SMOC1, SMOC2, TNFRSF1B | - Six-protein prediction model (GFAP, NPTX2, PEA15, SMOC1, SMOC2, TNFRSF1B) with excellent predictive performance (AUC>0.9) - Six early biomarkers far exceed the warning time window of traditional markers | Y (Three Independent Datasets) | Shen Y et al.[69] |
| AD | Metabolomics | Diagnosis | Sphingolipids | - Identified sphingolipids map to AD-related pathways (TAU phosphorylation, amyloid metabolism, calcium homeostasis, acetylcholine biosynthesis, apoptosis AD is associated with dysregulation of transmethylation and polyamine pathways. - Sphingolipids as early AD biomarkers. | Y (Independent Cohort) | Varma VR et al.[70] |
| AD | Metabolomics | Diagnosis | PKM2 | - PKM is an important glycolytic enzyme associated with AD pathology. - Induced neurons iNs from AD patients express cancer-associated PKM2. - PKM2 promotes Warburg effect-like glycolytic reprogramming in old neurons. - PKM2 specifically interacts with and enhances the transcription factors *STAT3* and *HIF1α*, promoting AD-induced neurons’ neuronal fate loss. - PKM2 is a potential diagnostic target for AD. | N | Traxler L et al.[71] |
| AD | ScRNA-seq | Diagnosis | Myelination-related gene (*LINGO1*) | - Myelination plays a pivotal role in the pathophysiology of AD. - The myelination-related gene *LINGO1* is perturbed in neurons and glial cells in patients with AD. | Y (Two Independent Datasets | Mathys H et al.[73] |
| AD | SnRNA-seq | Diagnosis | *APOE* | - The transcription factor EB serves as a principal regulator of lysosomal function, modulating multiple disease-associated genes in specific astrocyte subpopulations in AD. - The risk gene *APOE* exhibits upregulated expression in specific microglia and astrocytes in AD, correlating with the severity of TAU pathology. - *APOE* as a risk gene for AD has been confirmed. | N | Grubman A et al.[74] |
| AD | Spatial transcriptomics | Diagnosis | *OLIG* | - Early plaque-proximal dysregulation of *OLIG*/myelination gene co-expression networks in AD. - Multicellular 57-PIG networks emerge, enriched for complement activation, oxidative stress, lysosomal dysfunction, and neuroinflammatory pathways. - *OLIG* can be used as a marker for future AD diagnosis to lay the foundation for AD diagnosis. | Y (Mouse Model) | Wei-Ting Chen et al.[75] |
| AD | Spatial transcriptomics | Diagnosis | *SPARC, CALB2, DIRAS2,* and *KRT17* | - 10× Visium + co-immunofluorescence of AD markers delineated gene expression architecture in human middle temporal gyrus. - Cortex-specific layer-enriched DEGs, including novel candidates *SPARC, CALB2, DIRAS2,* and *KRT17,* exhibiting pronounced alterations. - These genes demonstrate significant potential as diagnostic targets for AD. | Y (Independent cohort) | Shuo Chen et al.[76] |
| AD | ScRNA-seq, Spatial transcriptomics | Treatment | Inhibitor of *PTPRG* or *VIRMA* | - *PTPRG+* microglia subpopulation induces neuronal *VIRMA* via intercellular signaling. - Neuronal *PTPRG* binding to *VIRMA* enhances RNA stability; upregulated *VIRMA* increases PRKN m6A, reduces its RNA stability, causing mitophagy-driven neuronal death and AD progression. - *PTPRG/VIRMA* inhibitors show their impacts on mitochondrial function and neuronal survival, offering potential therapies for AD. | Y (Mouse Model) | Donghua Zou et al.[77] |
| AD | SnRNA-seq, Spatial transcriptomics | Treatment | Lipid | - Specific microglial and astrocytic subtypes linked to Aβ, TAU, and lipid pathways were identified, revealing new therapeutic targets for AD. - Lipid-associated microglia drive Aβ-TAU interplay, while reactive astrocytes exacerbate TAU-mediated inflammation and cognitive decline. - Altered glial communities and divergent aging trajectories directly promote AD through multicellular dysregulation, enabling early intervention strategies. | N | Green, G S et al.[78] |
| AD | Spatial transcriptomics,  Epigenomes | Diagnosis | *SPI1/PU.1* | - *PU.1* contributes to inter-individual differences in microglial regulatory networks. - Reduced *PU.1* binding disrupts chromatin stability, supporting its functional role in AD. - *SPI1/PU.1* identified as key regulator of microglial gene expression and AD risk. | N | Kosoy, R et al.[79] |
| AD | Spatial transcriptomics,  Epigenomes | Diagnosis | *SPI1, ELF2, RUNX1* | - AD risk loci are highly enriched in microglial enhancers and TF-binding sites (*SPI1, ELF2, RUNX1*). - Glial cells show accessible regulatory changes in late AD, while neurons exhibit alterations in early stages. - Suggests epigenomic erosion as a hallmark of late AD and a potential indicator of disease progression. | N | Xiong, X et al.[80] |
| AD | Spatial transcriptomics,  Epigenomes | Diagnosis | SST⁺ level, Pvalb⁺/Vip⁺ level | - An early stage characterized by slow pathological accumulation, inflammatory microglia, reactive astrocytes, loss of *SST⁺* inhibitory neurons, and remyelination by oligodendrocyte precursor cells. - A late stage marked by exponential pathological increase and loss of both excitatory neurons and inhibitory (*Pvalb⁺/Vip⁺*) neuronal subtypes. | N | Gabitto, M I et al.[81] |
| AD  (autosomal dominant) | SnRNA-seq, Transcriptomics | Diagnosis | *LRP1, FKBP1B, PSEN1* | - In autosomal dominant AD, autophagy and chaperone genes show marked upregulation, with spatially resolved transcriptomics confirming specific activation of chaperone-mediated autophagy. - In autosomal dominant AD cases, astrocytic *LRP1* and *FKBP1B* upregulation alongside neuronal *PSEN1* downregulation may collectively represent an intrinsic protective mechanism. | Y (Independent Cohort) | Almeida, M C et al.[82] |
| AD | Proteomics, Transcriptomics | Diagnosis | *MAPK*/metabolic module, matrix body module | - AD-related modules include *MAPK* signaling/metabolism and matrixsome modules. - Matrixsome module is affected by *APOE ε4* allele. - *MAPK*/metabolism module links to cognitive decline rate. - Disease modules are potential AD targets/biomarkers. | N | ECB et al.[84] |
| AD | Proteomics, Transcriptomics | Diagnosis | *FBP1, FBP2, RHOH, JPH2, ERAP2, SCLT1,* and *MBP* | - *FBP1, FBP2, RHOH, JPH2, ERAp2,* and *SCLT1* are upregulated in *APOE4* cases compared to average expression in the normal brain. - *MBP* is one of the top candidate genes enhancing the relevance of myelination in AD. - Biomarkers show consistent protein profiles in plasma and brain. | Y (Two Independent Datasets) | Madrid L et al.[85] |
| AD | Genomics, Transcriptomics, Proteomics, Metabolomics | Diagnosis | *ABCA1, CPT1A, Adiponectin* and *NGAL* | - Short-chain acylcarnitines/amino acids and medium/long-chain acylcarnitines are closely correlated with the severity of AD. - Two genes (*ABCA1* and *CPT1A*) and two proteins (Adiponectin and *NGAL*) participate in the regulation of acylcarnitines and amino acids in AD. - *ABCA1, CPT1A*, Adiponectin, and *NGAL* may be AD diagnostic biomarkers. | Y (Two Independent Datasets) | Horgusluoglu E et al.[86] |
| AD | Proteomics, Transcriptomics | Diagnosis | *IVD, CYFIP1* and *ADD2* | - Significantly higher *IVD* protein abundance in AD patients. - *CYFIP1* and *ADD2* are significantly downregulated in AD patients. - *IVD, CYF0IP1,* and *ADD2* combine to diagnose AD. | N | San Segundo et al.[87] |
| AD | Genomics, Metabolomics | Diagnosis | *CSTD, CTSB, CTSD,* and *GM2A* | - *CSTD* has been validated as a marker in previous CSF and plasma samples. - AD progression is clearly accompanied by increased fold changes in these lysosomal proteins. - Lysosomal proteins *CTSB, CTSD,* and *GM2A* are significantly increased as markers in CSF samples from AD patients. | N | Wang H et al.[88] |
| AD | Genomics, Proteomics | Diagnosis | *PBXIP1* | - *PBXIP1*-encoded protein shows significant association with all three AD neuropathological features. - *PBXIP1* is associated with AD through its role in astrocytes and hippocampal neurons and the mTOR pathway. - *PBXIP1* is associated with neuropathology and cognitive function. | Y (Two Independent Datasets) | Jingyun Zhang et al.[89] |
| AD | Genomics, Transcriptomics, Proteomics | Diagnosis | *H3K27ac* | - Differentially acetylated peaks are enriched in disease-related biological pathways, including those associated with Aβ and TAU pathology progression. - Highly significant enrichment of AD risk variants in the *H3K27ac* peak region of the inner olfactory cortex, including *CR1, GPR22, KMO, PIM3, PSEN1,* and *RGCC*. - *H3K27ac* can serve as a diagnostic target for AD. | N | Marzi SJ et al.[90] |
| AD | Transcriptomics, Proteomics, Epigenomics | Diagnosis | *H3K27ac, H3K9ac* | - RNA-seq analysis reveals upregulation of histone acetyltransferases in *H3K27ac* and *H3K9ac*. - Genome-wide increases in *H3K27ac* and *H3K9ac* exacerbate Aβ42-driven neurodegeneration. - Proteomic screening singles out *H3K27ac* and *H3K9ac* as major AD-specific enrichments. - *H3K27ac* and *H3K9ac* affect disease pathways through dysregulated transcription and chromatin gene feedback loops. | Y (Two Independent Datasets, Drosophila Model) | Raffaella Nativio et al.[91] |
| AD | Proteomics, Metabolomics, Lipidomics | Diagnosis | Protein 14-3-3 zeta/delta, clusterin, interleukin-15, and transgelin-2 | - Enrichment pathway analysis reveals overexpression of hemostatic, immune response, and extracellular matrix signaling pathways associated with AD. - Protein 14-3-3 zeta/delta, clusterin, interleukin-15, and transgelin-2 improve AD prediction. | N | Clark C et al.[92] |
| AD | Proteomics, Metabolomics, Lipidomics | Diagnosis | GABA synthesis, arginine biosynthesis, and alanine, aspartate, glutamate, and arginine pathways | - Gender-dependent effects are seen on the pathways of significant enrichment, including those of GABA synthesis, arginine biosynthesis, and alanine, aspartate, glutamate, and arginine metabolism. - Lysophospholipid and amino acid metabolism are involved in the AD brain. | Y (Mouse Model) | Abigail Strefeler et al.[93] |
| AD | Genomics, Transcriptomics | Treatment | *TRPV1* | - *TRPV1* activation rescues memory deficits and neuronal loss in *APOE4* high-fat diet-fed mice. - Neuronal loss increases in *APOE4* high-fat diet mice, rescued by *TRPV1* activation in the capsaicin group. - *TRPV1* is a treatment option for AD disease. | Y (Mouse Model) | Chenfei Wang et al.[94] |
| AD  (late-onset) | Genomics, Transcriptomics | Treatment | *ATP6V1A* | - *ATP6V1A* has been identified as a key regulator of the top neuronal subnetwork, which is the most dysregulated in late-onset AD. - *ATP6V1A* can be used as a therapeutic target. - NCH-51 ameliorates neuronal damage caused by *ATP6V1A* deficiency in a Drosophila model. | Y (Drosophila Model) | Wang M et al.[95] |

AlzGPS: Alzheimer's disease genome-wide positioning systems platform; CSF: cerebrospinal fluid; PKM2: pyruvate kinase M2; *MBP*: Myeloid basic protein encoding gene.

**Supplementary Table S2:** Application of Multi-omics and High-spatial-resolution Omics Technologies in PD.

| Disease | Omics Type | Purpose | Biomarker | Relevance to PD Pathogenesis | Experiment Validation | Reference |
| --- | --- | --- | --- | --- | --- | --- |
| PD | Genomics | Diagnosis | *ZNF184, IL1R2, LRRK2, ITPKB,* and *PARK16* | - Alleles of *LRRK2* and *IL1R2* confer a higher risk of developing PD. - The genotype models of *ZNF184,* *PARK16,* and *ITPKB* are significantly associated with PD. - Most of these genes are involved in autophagy and lysosomal function-related pathways. | N | Gao T et al.[102] |
| PD | Genomics | Diagnosis | *HLA, LRRK2, MAPT, TRIM10,* and *SETD1A* | - *HLA, LRRK2, MAPT, TRIM10,* and *SETD1A* are high-risk genes associated with PD. - Significantly associated loci linked to PD are found in the *HLA* and *MAPT* gene loci. | N | Witoelar A et al.[103] |
| PD | Transcriptomics | Diagnosis | *SSR1* | - *SSR1* is found to be upregulated in PD patients. - *SSR1* expression is negatively correlated with dopaminergic neuron survival. - The upregulation of *SSR1* expression in peripheral blood precedes the abnormal behavior of the animals. - The *SSR1*-based RF classifier has an AUC value of 0.91 and can be used as a diagnostic marker. | N | Zhang W et al.[104] |
| PD | Proteomics | Diagnosis | OMD*,* CD44*、*VGF*,* PRL*,* MAN2B1*,* and LRRK2 | - ML identifies that OMD, CD44, VGF, PRL*,* and MAN2B1 show significant changes in PD patients and are significantly correlated with PD clinical scores. - The enhanced neuroinflammatory characteristics in LRRK2 gene carriers are strongly correlated with PD. - OMD, CD44, VGF, PRL, MAN2B1*,* and LRRK2 can be used as biomarkers for PD. | N | Karayel, MM et al.[105] |
| PD | Metabolomics | Diagnosis | Lipid metabolism related to carnitine shuttle, sphingolipid metabolism, and arachidonic acid metabolism | - Alterations in lipid metabolism related to carnitine shuttle, sphingolipid metabolism, arachidonic acid metabolism, and fatty acid biosynthesis are detected. - Carnitine shuttling is the most important pathway associated with unmedicated PD patients by sebum. | N | Sinclair E et al.[106] |
| PD | Metabolomics | Diagnosis | Short-chain fatty acids, butyric acid | - Low levels of short-chain fatty acids are significantly associated with cognitive decline in PD patients. - Decreased butyric acid levels are associated with poorer posture and gait disorder scores. - Short-chain fatty acids and butyric acid serve as a potential diagnostic target. | N | Tan AH et al.[107] |
| PD | Metabolomics | Diagnosis | Proline | - Energy and lipid metabolism are overexpressed in PD. - 139 metabolites, including proline, have notable changes in carnitine shuttle, vitamin E metabolism, lipid-related, glycerol phospholipids, sphingolipids, and fatty acids pathways. - Proline and 139 other metabolites are considered particularly predictive of PD status. | Y (Independent Cohort) | Pereira, P.A.B et al.[108] |
| PD | Metabolomics | Diagnosis | Phenylacetic acid, phenylacetylglutamine, histidine, uric acid, and imidazoleacetic acid | - 45 metabolic markers in PD patients show high diagnostic power in early stages (AUC=0.92). - Metabolites like phenylacetic acid, phenylacetylglutamine, histidine, uric acid, and imidazoleacetic acid show upregulated urine levels in PD, linked to neuro disorders. | N | Cai Z et al.[109] |
| PD | Metabolomics | Diagnosis | BCAA metabolism, glycine derivatives, steroid hormone biosynthesis, tryptophan, and phenylalanine metabolism | - 18 differential metabolites in urine have been identified as biomarkers for PD. - Differential metabolites alter metabolic pathways associated with BCAA metabolism, glycine derivatives, steroid hormone biosynthesis, tryptophan metabolism, and phenylalanine metabolism. | N | Cai Z et al.[110] |
| PD | SnRNA-seq | Diagnosis | *IL1B, GPNMB,* and *HSP90AA1* | - A neuron cluster characterized by *CADPS2* overexpression and low tyrosine hydroxylase levels is identified in PD. - Astrocytes and microglia in PD show specific proliferation and gene dysregulation linked to unfolded protein response and cytokine signaling. - Microglia show a pro-inflammatory state with high *IL1B,* *GPNMB,* and *HSP90AA1,* suggesting their diagnostic potential. | N | Semra Smajić et al.[111] |
| PD | ScRNA-seq | Treatment | *HSP90* inhibitors | - Neurons and glial cells in PD exhibit dysfunction, immune dysregulation, and impaired protein folding. - Administration of *HSP90* inhibitors accelerates the degradation of inflammasomes, reducing inflammatory responses and alleviating neurodegeneration. | Y (Mouse Model) | [Gabriel](https://www.nature.com/articles/s41392-024-02086-7#auth-Gabriel-Gonzalez_Escamilla-Aff1) GE et al.[112] |
| PD | Single-cell genomics,  Spatial transcriptomics | Diagnosis | *TP53, NR2F2* | - The AGTR1-marked SNpc ventral subtype is highly PD-susceptible, showing *TP53/NR2F2* target gene upregulation. - *TP53/NR2F2*-regulated pathways are key to PD-related neuronal death. - *TP53/NR2F2* target gene upregulation indicates diagnostic biomarker potential. | Y (Macaque Model) | Tushar Kamath et al.[113] |
| PD | ScRNA-seq, Proteomics | Diagnosis | *SYN2* | - Negative correlation between α-synuclein pathology and chaperone protein expression in excitatory neurons in PD, along with weakened neuron-astrocyte interaction and aggravated neuroinflammation. - *SYN2* enrichment in PD brain regions suggests significant increase in synaptic signaling at both RNA and protein levels. - *SYN2* as a potential diagnostic biomarker for PD | Y (Independent Cohort) | Biqing Zhu et al.[114] |
| PD | Proteomics, Transcriptomics | Diagnosis | *GPNMB, CD38,* and *DGKQ* | - *GPNMB* and *CD38* show significant causal effects in PD, with evidence from quantitative trait locus analysis and fine mapping. - *GPNMB, CD38,* and *DGKQ* proteins are associated with PD risk. | Y (Three Independent Datasets) | Guxiaojing et al.[115] |
| PD | Three Proteomics | Diagnosis | DDC | - DDC*,* SUMF1, DPP7, ENPEP, WFDC2*,* and hundreds of proteins are upregulated in the CSF, blood, or urine of PD patients. - DDC levels are linked to symptom severity in PD patients. - DDC can serve as a target for accurate PD diagnosis. | Y (Seven Independent Datasets) | Rutledge J et al.[116] |
| PD | Transcriptomics, Metabolomics | Treatment | The relaxin signaling pathway, adhesion patch, and PI3K-Akt signaling pathway | - BHD reduces PD symptoms, impacting metabolic pathways, including the relaxin signaling pathway, adhesion patch, and *PI3K-Akt* signaling pathway. - BHD promotes the survival of dopaminergic neurons in PD mice, leading to improved motor performance. | Y (Mouse Model) | Hujun et  al.[117] |
| PD | Genomics, Metabolomics | Diagnosis/Treatment | *CircSV2b* | - Detect 33 deregulated circular RNAs in the PD mouse model vs wild-type controls. - *CircSV2b* overexpression via the ceRNA-Akt1 axis mitigates oxidative stress in PD. - *CircSV2b* is a potential Parkinson's diagnostic and curative biomarker. | Y (Mouse Model) | Cheng Qc et al.[118] |

BCAA: branched chain amino acid; SNpc: substantia nigra pars compacta; DDC: Dopamine decarboxylase; ML: machine learning; *LRRK2*: leucine-rich repeat kinase 2; SUMF1: sulfatase-modifying factor 1; DPP7: dipeptidyl peptidase 2/7; *SSR1*: signal sequence receptor subunit 1; ENPEP: glutamyl aminopeptidase; WFDC2: WAP four-disulfide core domain 2; BHD: Buyang Huanwu Decoction.

**Supplementary Table S3:** Application of Multi-omics and High-spatial-resolution Omics Technologies in Epilepsy.

| Disease | Omics Type | Purpose | Biomarker | Relevance to Epilepsy Pathogenesis | Experiment Validation | Reference |  |  |
| --- | --- | --- | --- | --- | --- | --- | --- | --- |
| Epilepsy | Transcriptomics | Diagnosis | *P38MAPK, JAK-STAT, PI3K,* and mTOR signal pathway | - The*P38MAPK, JAK-STAT,* and *PI3K* consistently exhibit high expression and along with stable regulation of mTOR signaling pathways in epilepsy patients. - Differential genes engage in signal cascades, ECM remodeling, cell motility, apoptosis, and immune responses linked to seizures. | N | Oswaldo K Okamoto et al.[122] |  |  |
| Epilepsy  (temporal lobe) | Transcriptomics | Diagnosis | *Tlr2, Lgals3, Serpine 1* and *Stat3* et al. | - Several hub genes identified in TLE, such as *Tlr2, Lgals3, Serpine1,* and *Stat3,* et al., positively correlate with seizure frequency. - Activation and phagocytic activity of microglia/macrophages have changed during the epileptic occurrence process of TLE. - *Tlr2, Lgals3, Serpine 1,* and *Stat3* can serve as markers for TLE. | N | QingLan Chen et al.[123] |  |  |
| Epilepsy | Transcriptomics | Diagnosis | *GABAergic* | - FBTCS+ patients exhibit more widespread bilateral cortical and subcortical morphological alterations compared to FBTCS− patients. - Excitatory and inhibitory neurons are affected in FBTCS−, while only excitatory neurons are significantly altered in FBTCS+. - Excitatory/inhibitory imbalance and *GABAergic* dysfunction may underlie FBTCS susceptibility. | N | Lin, Q et al.[124] |  |  |
| Epilepsy | Transcriptomics | Diagnosis | *NMDAR* | - Analyses identified upregulation of the *NMDAR* signaling pathway as a key mechanism underlying both the autism-like behaviors and the observed anti-epileptic phenotypes. - Impaired *GABAergic* function and enhanced *NMDAR* activity disrupt excitatory-inhibitory balance, promoting autism-epilepsy comorbidity. - Dysfunctional *GABAergic* signaling and elevated *NMDA* activity contribute to co-occurrence of autism and epilepsy. | Y (Mouse Model) | Fan, C et al.[125] |  |  |
| Epilepsy  (temporal lobe) | Transcriptomics | Diagnosis | *RBFOX1* | - TLE shows synaptic network reorganization, reduced connectivity, lower clustering, longer pathways, mainly in temporolimbic and frontoparietal regions. - Identified 183 downregulated synaptic genes: *RBFOX1* and other *GABAergic* genes are central. - Coordinated downregulation of risk genes may drive synaptic dysfunction and epilepsy in TLE, suggesting new treatment targets. | Y (Two Independent Datasets) | Li, R et al.[126] |  |  |
| Epilepsy | Proteomics | Diagnosis | GFAP | - GFAP is consistently downregulated in brain tissue with high spike frequencies and exhibits a strong negative correlation with spike frequency. - Reactive astrocytes, such as GFAP, protect the neocortex from epileptic discharges rather than induce them. - Epilepsy severity is closely linked to decreased GFAP (astrocyte marker) levels. | N | Gal Keren-Aviram et al.[127] |  |  |
| Epilepsy | | Proteomics | Diagnosis/Treatment | ADPRC, LPAR3, calreticulin, UCH-L1, SNAP-25, and transgelin-3 | - A total of 144 differentially expressed proteins, such as ADPRC, LPAR3, calreticulin, UCH-L1, SNAP-25, and transgelin-3, are identified in the epileptic hippocampal regions. - Most differentially expressed proteins are associated with Ca2+ homeostasis. - Inhibiting calcium influx alleviates seizures triggered by excessive brain Ca²+ rise in epilepsy. | N | Leila Sadeghi et al.[128] | |
| Epilepsy | | Proteomics | Diagnosis | Calcineurin | - Tutin induces epilepsy by activating calcium-modulating phosphatase and produces significant neurological damage. - Calcineurin is a target of tutin, and that tutin activates Calcineurin, leading to seizures. | Y (Mouse Model) | Shi-Shan Yu et al.[129] | |
| Epilepsy | Metabolomics | Diagnosis | N-acetyl glycoprotein, lactate, creatine, glycine, lipid, and citrate | - Serum N-acetyl glycoprotein, lactate, creatine, glycine, and lipid levels are elevated decreased levels of citrate in epileptic children, while the level of citrate is reduced. - The aforementioned metabolic substances are potential diagnostic targets for epilepsy. | N | Łukasz Boguszewicz et al.[130] | |  |
| Epilepsy  (mesial temporal lobe) | Metabolomics | Diagnosis | GABA | - GABA is significantly increased in the epileptogenic zone of KA-MTLE mice. - GABA is a specific biomarker of the epileptogenic zone in MTLE. | Y (Mouse Model) | Hamelin, S et al.[131] | |  |
| Epilepsy | SnRNA-seq | Diagnosis | *Sst* and *Pvalb* | - Major transcriptomic alterations occur in principal neurons (*L5-6_Fezf2, L2-3_Cux2*) and *GABAergic* interneurons (*Sst, Pvalb*). - Profound dysregulation in glutamate signaling, characterized by robust upregulation of glutamate receptor genes, notably within *Sst/Pvalb* subtypes. - *Sst/Pvalb* interneurons represent potential diagnostic targets and are fundamental to early epileptogenesis. | N | Ulrich Pfisterer et al.[132] | |  |
| Epilepsy  (post-traumatic) | ScRNA-seq | Diagnosis | *XIST* | - Hereditary epilepsy shows higher oligodendrocyte/astrocyte counts, lower microglia/neuron counts vs PTE. - *IL-17* signaling in microglia/astrocytes can be a PTE target/biomarker. - *XIST*, upregulated in PTE, drives inflammation/fibrosis, useful for diagnosis and mechanism study. | N | Fang Wen et al.[133] |  |  |
| Epilepsy  (temporal lobe) | ScRNA-seq, SnRNA-seq, Spatial transcriptomics | Diagnosis | *SPP1, Trem2, Tle4 and Sipa1l3* | - The differentially up-regulated genes in TLE patients are predominantly expressed in glial cells, while the down-regulated genes are mainly expressed in neurons. - *SPP1* and *Trem2* are up-regulated in glial cells, whereas *Tle4* and *Sipa1l3* are down-regulated in these cells. | N | Quanlei Liu et al.[134] |  |  |
| Epilepsy | Genomics, Transcriptomics | Diagnosis | *Sestrin 3* | - *Sestrin 3* is a key regulator in the pro-convulsant gene network in the hippocampus of human epilepsy. - *Sestrin 3* positively regulates modules in macrophages, microglia, and neurons. - *Sestrin 3* holds potential as a diagnostic means for epilepsy. | Y (Mouse Model) | Johnson, M.R. et al.[135] |  |  |
| Epilepsy | Proteomics, Transcriptomics | Diagnosis | *STAT3, ErbB,* and *MAPK8* | - The TGF-β pathway is associated with cardiac function in the hearts of epileptic animals. - *STAT3, ErbB,* and *MAPK8* are key regulators of cardiac alterations in epilepsy that contribute to seizure-mediated cardiac damage. | N | Sharma, S et al.[136] |  |  |
| Epilepsy | Proteomics, Metabolomics | Diagnosis | GSTM1, ALDH2 | - Within the somatosensory cortex module, GSTM1 is identified as a protein hub and elevated expression levels. - In the thalamus module, ALDH2 is pinpointed as a protein hub. - The metabolic pathway enriched by the differences is lysine degradation. - GSTM1 and ALDH2 are identified as markers for seizure-related modules in epilepsy. | N | Harutyunyan, A et al.[137] |  |  |
| Epilepsy | Genomics, Metabolomics | Diagnosis | Lactate, creatine, phosphocreatine, and choline | - Lactate is significantly reduced, while creatine, phosphocreatine, and choline are significantly increased. - Lactate is involved in G protein-coupled receptor signaling and angiogenic pathways, and shows upregulation of ubiquitination-related genes. | N | Wu, H.C. et al.[138] |  |  |
| Epilepsy | Proteomics, Transcriptomics | Treatment | *miR-10a-5p, miR-21a-5p* and *miR-142a-5p* | - *miR-10a-5p, miR-21a-5p,* and *miR-142a-5p* are identified as key transcripts. - These microRNA transcripts are primarily associated with the TGF-β pathway signaling. - The combination of anti-miR (*miR-10a-5p, miR-21a-5p, miR-142a-5p*) exhibits protective effects against acute and spontaneous seizures. | Y (Mouse Model) | Venø, M.T. et al.[139] |  |  |

PTE: post-traumatic epilepsy; TLE: temporal lobe epilepsy; MTLE: mesial temporal lobe epilepsy; NMDA: N-methyl-D-aspartate; GABA: γ-aminobutyric acid; *GFAP*: glial fibrillary acidic protein; KA-MTLE: kainic acid into mesiotemporal lobe epilepsy mice; TGF-β, transforming growth factor β; ADPRC: ADP-ribosyl cyclase; FBTCS: focal to bilateral tonic–clonic seizures; LRRK2: leucine-rich repeat kinase 2; LPAR3: lysophosphatidic acid receptor 3; UCH-L1: ubiquitin carboxyl-terminal hydrolase L1; GSTM1: glutathione s-transferase M1; SNAP-25: synaptosome-associated protein 25.

**Supplementary Table S4:** Application of Multi-omics and High-spatial-resolution Omics Technologies in MS.

| Disease | Omics Type | Purpose | | Biomarker | Relevance to MS Pathogenesis | Experiment Validation | Reference |
| --- | --- | --- | --- | --- | --- | --- | --- |
| MS | Genomics | Diagnosis | *STAT3, IL7* | | - Inhibitory neurons in the CNS are key contributors to MS susceptibility. - Genes such as *STAT3* and *IL7* disrupt immune pathways specifically in inhibitory neurons. - Targeted interventions for CNS pathways—such as neuronal and glial functions, including *STAT3* and *IL7*. | Y (Three Independent Datasets) | [Philip De Jager](https://pubmed.ncbi.nlm.nih.gov/?term=De+Jager+P&cauthor_id=39866869) et al.[146] |
| MS | Proteomics | Diagnosis | CXCL13, LTA, FCN2, ICAM3, LY9, SLAMF7, TYMP, CHI3L1, FYB1, TNFRSF1B, and NFL | | - Lower levels of *NFL* in CSF show predictive potential for disease activity (AUC=0.77). - An 11-protein panel in CSF has a high AUC for prediction, including CXCL13, LTA, FCN2, ICAM3, LY9, SLAMF7, TYMP, CHI3L1, FYB1, TNFRSF1B, and NFL (AUC=0.9). - All the above proteins can be markers for MS. | N | Mika Gustafsson et al.[147] |
| MS | Metabolomics | Diagnosis | DRD2 | | - DRD2 exacerbates the disease by promoting inflammation and reducing the abundance of Lactobacillus species in the microbiome. - Lactobacillus-derived N2-acetyl-L-lysine inhibits microglial activation, combating neurodegeneration. - Intestinal epithelial DRD2, serving as a biomarker, can modulate the gut microbiome in MS. | N | Hairong Peng et al.[148] |
| MS  (relapsing-remitting) | Metabolomics | Diagnosis/Treatment | Glycolysis | | - Identified four perturbed metabolic pathways, including structural/signaling lipids and energy, in the serum of patients with MS. - Glycolysis is the common upstream feeding of these altered metabolic pathways. - Targeting glycolysis in experimental autoimmune encephalomyelitis ameliorated the disease pathology by impeding immune cell effector function. | Y (Mouse Model) | Insha Zahoor et al.[149] |
| MS | ScRNA-seq | Diagnosis | *TFH* | | - Myeloid dendritic cells and regulatory T cells are enriched in the CSF of patients with MS. - The independent increase in clusters of TFH cells drives the known expansion of B-lineage cells in the CSF in MS. - TFH cells promote the infiltration of B cells into the central nervous system, exacerbating MS disease. | Y (Mouse Model) | David Schafflick et al.[150] |
| MS | ScRNA-seq, Spatial transcriptomics | Diagnosis | *SERPINA3* | | - Astrocytes can be classified into three types: homeostatic, intermediate, and disease-associated types. - In patients with DA-Astro, the expression level of *SERPINA3* is significantly elevated. - *SERPINA3* expression may constitute a glial cell survival response to resolve inflammation and prevent apoptosis during both initial and late resolution phases. | N | Petra Kukanja et al.[151] |
| MS | ScRNA-seq, Spatial transcriptomics | Diagnosis | *MAFB* | | - The expression of pro-inflammatory molecules in oligodendrocytes near axonal damage is elevated in MS patients. - *MAFB* mediates intercellular communication via complement factors and apolipoproteins. - The inflammatory transcription factor *MAFB* serves as a biomarker for MS lesions. | N | Maria L Elkjaer et al.[152] |
| MS  (systemic) | ScRNA-seq,  Spatial transcriptomics, Spatial proteomics | Diagnosis/Treatment | *POSTN/SCARA5,*  *CXCR4* | | - A dynamic spatial interaction network is established between fibroblasts and macrophages via the *ACKR3-CXCL12-CXCR4* signaling axis, playing a central role in driving fibrosis progression. - Treatment with the *CXCR4* inhibitor AMD3100 significantly alleviates fibrosis in skin and lung tissues. - The significantly elevated *POSTN/SCARA5* ratio in MS can serve as a predictive diagnostic biomarker. | Y (Mouse Model) | Zhijian Li et al.[153] |
| MS | Proteomics, Transcriptomics | Diagnosis | *GPR37L1, SIRPA, FGFR3, CADM3,* and *TYRO3* | | - Neurological candidate molecules, including *GPR37L1, SIRPA, FGFR3, CADM3*, and TYRO3, are highly expressed in the CNS of MS. - These genes are associated with early neuronal degeneration and dysfunctional trophic/anti-inflammatory intercellular communication. - *GPR37L1, SIRPA, FGFR3, CADM3,* and *TYRO3* can be used as a diagnostic method for MS. | N | Max Kaufmann et al.[154] |
| MS | Proteomics, Transcriptomics | Diagnosis | 24 iron death-related genes (*CHMP5, SLC38A1, PML*, etc.) | | - High iron death scores at the margins of active lesions correlate with phagocytic activation. - Elevated iron death scores in cortical neurons are associated with neurological diseases. - A blood-based model of 24 iron death-related genes is a prognostic marker for diagnosing MS, including C*HMP5, SLC38A1, PML,* etc. | N | Tao Wu et al[155] |
| MS | Proteomics,  Metabolomics | Diagnosis | LAMP1, FCG2A, and HPSE | | - *HPSE* is positively correlated with many MS-related metabolites, including L-tyrosine, sphingosine 1-phosphate, sphingosine 1-phosphate, and L-tryptophan. - The proteins LAMP1, FCG2A, and HPSE exhibit potential utility as specific biomarkers for MS. | N | Fan Yang et al.[156] |
| MS | Proteomics, Metabolomics | Diagnosis | Equine uric acid, sphingolipids | | - Anti-inflammatory molecules and sphingolipids are reduced by metabolomics in MS patients. - Low levels of equine uric acid in a severe subgroup of MS. - Sphingolipids and equine uric acid facilitate the future development of biomarkers and targeted therapeutic interventions for MS. | N | Qinming Zhou et al.[157] |

NFL: neurofilament light chain; DRD2: Dopamine Receptor D2; CNS: central nervous system; HPSE: heparinase; TFH: T follicular helper.

**Supplementary Table S5:** Application of Multi-omics and High-spatial-resolution Omics Technologies in Stroke.

| Disease | Omics Type | Purpose | Biomarker | Relevance to Stroke Pathogenesis | Experiment Validation | Reference |
| --- | --- | --- | --- | --- | --- | --- |
| Stroke  (ischemic) | Proteomics | Diagnosis | NSF, RhoGDI1, and RabGDI | - Circulating NSF, RhoGDI1, and RabGDI are upregulated in patients with IS. - These proteins trigger neuronal depolarization and calcium surge, activating death pathways in stroke. | Y (Independent Cohort) | Eloy Cuadrado et al.[160] |
| Stroke  (ischemic) | Proteomics | Diagnosis | CMPK, CKB | - Circulating levels of CKB and CMPK are higher in patients with ischemic stroke than in controls during the acute phase. - CKB plays a crucial role in energy transduction and homeostasis. - CMPK is released in large amounts and participates in mechanisms that counteract cell disruption and neuronal cell death. | N | Alba Simats et al.[161] |
| Stroke  (ischemic) | Proteomics | Diagnosis | SAHH2 | - SAHH2 plays a significant role in the coordinated inhibition of Ca²+ ion transporters. - Increased expression of SAHH2 in neurons from the infarcted area is probably because of ischemia-triggered Ca2+ mobilization. | N | Teresa García-Berrocoso et al.[162] |
| Stroke | Transcriptomics | Diagnosis | LncRNA (*MEG3, H19,* and *MALAT1*) | - LncRNAs, such as *MEG3, H19,* and *MALAT1,* in blood cells between patients with stroke and healthy controls show differences. - Differential genes modulate neuronal survival/apoptosis targets, impacting p53-mediated apoptosis in stroke. | N | Cheryl Dykstra Aiello et al.[163-167] |
| Stroke | Transcriptomics | Diagnosis | Extracellular microRNA | - Decreased levels of extracellular *miR-32-3p, miR-106b-5p, miR-423-5p, miR-451a, miR-1246, miR-1299, miR-3149* and *miR-4739,* and increased levels of extracellular *miR-224-3p, miR-377-5p, miR-518b, miR-532-5p* and *miR-1913* associate with stroke. - These genes affect multiple pathways such as apoptosis, oxidation, angiogenesis, and neurogenesis in IS. | N | Ceren Eyileten et al.[168] |
| Stroke | Transcriptomics | Diagnosis | *IFN-I* | - Aged brains show marked upregulation of *IFN-I* signaling following ischemic injury. - Aging leads to downregulation of genetic programs essential for axonal and synaptic integrity after stroke. - *IFN-I* may serve as a diagnostic biomarker for stroke. | N | Androvic, P et al.[169] |
| Stroke (cardioembolic) | Metabolomics | Diagnosis | Valine, Leucine, Isoleucine | - The expression levels of BCAA, including valine, leucine, and isoleucine, are decreased in patients with cardioembolic stroke. - Lower BCAA levels are also associated with poor neurological outcomes. | N | W Taylor Kimberly et al.[170] |
| Stroke | Metabolomics | Diagnosis | Total free fatty acid | - Plasma concentration of total free fatty acids is higher in patients with cardioembolic stroke than in patients with non-cardioembolic stroke. - Elevated free fatty acid levels are significantly associated with cardioembolic stroke, suggesting their potential as a diagnostic target. | N | Jeong Yoon Choi et al.[171] |
| Stroke | ScRNA-seq | Treatment | Microglia and macrophages | - Aging jeopardizes the repair and regeneration of the cerebrovascular system and proteins after stroke. - After stroke, microglia and macrophages may affect angiogenesis and oligodendrogenesis via paracrine mechanisms, impeding stroke recovery. - Transplanting microglia and macrophages from the brains of young mice into the cerebral cortex of aged stroke-affected mice partially restores angiogenesis and oligodendrogenesis. - Microglia and macrophages serve as effective targets for promoting stroke recovery | N | Chenghao Jin et al.[172] |
| Stroke  (ischemic) | ScRNA-seq,  Spatial transcriptomics | Treatment | *LILRB4* | - Stroke brains have up-regulated *LILRB4* and ischemia-linked microglial cluster 3. - *LILRB4* knockout worsens ischemic brain injury via CD8+ T cell recruitment; overexpression offers neuroprotection. - Targeting *LILRB4* and its downstream pathways represents an effective therapeutic strategy for ischemic stroke. | N | Yilin Ma et al.[173] |
| Stroke (brainstem) | SnRNA-seq, ScRNA-seq | Treatment | Myo1e | - Oligodendrocyte loss leads to neurological deficits following brainstem stroke. - *OLG8* has an innate neuroprotective effect in brainstem stroke. - Myo1e aids *OLG8* migration to the peri-infarct area in brainstem stroke. - Myo1e overexpression in *OLG8* oligodendrocytes boosts brainstem stroke recovery. | N | Shaojun Li et al.[174] |
| Stroke  (intracerebral hemorrhage) | ScRNA-seq, Spatial transcriptomics | Diagnosis | *SPP1, Lyz2* | - *SPP1/Lyz2* show high expression levels, and lymphocytes with high expression interact with myeloid cells in the late stage of stroke. - During the acute phase of intracerebral hemorrhage, Lgmn+Macro-T cells and microglia interact via the *SPP1-cd44* pathway. - *SPP1* and *Lyz2* are potential diagnostic targets for the acute phase of intracerebral hemorrhage. | N | Lingui Gu et al.[175] |
| Stroke | ScRNA-seq, Spatial transcriptomics | Treatment | Lipocalin-2 | - Ferroptosis is the primary programmed cell death process post-hemorrhagic stroke, mainly affecting mature oligodendrocytes. - A specific interaction between lipocalin-2-positive microglia and oligodendrocytes, mediated by the CSF1 receptor pathway, induces ferroptosis in oligodendrocytes and subsequent neurological deficits. - Early therapeutic intervention by inhibiting *LCN2* expression may alleviate ferroptosis-induced oligodendrocyte damage and related neurological deficits. | N | Lingui Gu et al[176] |
| Stroke | ScRNA-seq, Spatial transcriptomics | Treatment | *LGALS9* | - Galectin (*LGAL*) signaling is enhanced in microglia and macrophages of ischemic mice. - *LGALS9* treatment promotes oligodendrocyte remyelination and improves stroke recovery in mice. - *LGALS9* can serve as a therapeutic approach to ameliorate stroke. | N | Bing Han et al.[177] |
| Stroke  (subarachnoid hemorrhage) | ScRNA-seq, Spatial transcriptomics | Diagnosis | *THBS1, S100A6* | - *THBS1* and *S100A6* are closely associated with the prognosis of SAH, with their expression significantly increasing following the hemorrhage. - The *THBS1-CD47* pair regulates cell apoptosis, and blocking their interaction may represent a new therapeutic approach for SAH. - *THBS1* and *S100A6* serve as diagnostic biomarkers for stroke. | N | Xiaoyu Wang et al.[178] |
| Stroke | Genomics, Spatial transcriptomics | Diagnosis | *MMP-9* | - Plaque rupture occurs predominantly in proximal and most stenotic areas. - Identified *MMP-9* as a key gene causally linked to rupture risk. - Supports targeted intervention against *MMP-9* for precise stroke treatment. | N | Sun, J et al.[179] |
| Stroke | ScRNA-seq, Spatial transcriptomics | Diagnosis | *APOE*, *FABP5* | - Distinct astrocyte states were identified post-stroke, influenced by both time and proximity to the ischemic lesion. - Proximal astrocytes exhibited functional divergence in lipid transport, characterized by elevated expression of *APOE* and *FABP5* after cortical ischemic stroke. - *APOE* and *FABP5* hold promise as diagnostic biomarkers for stroke. | N | Scott, EY et al.[180] |
| Stroke (cardioembolic) | Proteomics, Transcriptomics | Diagnosis | *ICA1L, CAND2,* and *ALDH2* | - Reduced *ICA1L, CAND2,* and *ALDH2* may impair excitatory synaptic signaling, contributing to cardioembolic stroke pathogenesis. - *ICA1L, CAND2,* and *ALDH2* are potential biomarkers for lacunar stroke. | Y (Three Independent Datasets) | Zhang C et al.[181] |
| Stroke  (ischemic) | Genomics, Metabolomics | Treatment | Gut flora and metabolic disturbances | - ZHTC modulates the abundance of specific bacterial groups and 23 metabolic differences for IS, including arginine, L-lysine, and L-methionine. - ZHTC improves intestinal barrier integrity by increasing the expression levels of tight junction proteins - ZHTC meliorates IS by modulating gut flora and metabolic disturbances. | Y (Rat Model) | Wang R et al.[182] |
| Stroke  (ischemic) | Proteomics, Transcriptomics | Treatment | *PI3K-Akt, MAPK*, and cAMP signaling pathways | - *YQTL* reduces infarct volume percentage and improves neurological function in cerebral ischemia-reperfusion injury mice. - Network pharmacology and multi-omics studies reveal 15 components that regulate 82 targets and 19 pathways. - *YQTL* protects against cerebral ischemia-reperfusion injury through *PI3K-Akt, MAPK,* and cAMP signaling pathways. | Y (Mouse Model) | Yuan Y et al.[183] |

lncRNAs: long non-coding RNAs; IS: Ischemic Stroke; BCAA: branched-chain amino acid; *LGAL*: Galectin; *CSF1*: colony-stimulating factor 1; *SAH*: subarachnoid hemorrhage; ZHTC: Zhilong Huoxue Tongyu capsule; YQTL: Yiqi Tongluo granule; *ALDH2*: aldehyde dehydrogenase 2.

**Supplementary Table S6:** Application of Multi-omics and High-spatial-resolution Omics Technologies in Hydrocephalus.

| Disease | Omics Type | Purpose | Biomarker | Relevance to Hydrocephalus Pathogenesis | Experiment Validation | Reference |
| --- | --- | --- | --- | --- | --- | --- |
| Hydrocephalus  (communicating) | Genomics | Diagnosis | *TRIM71, SMARCC1, PIK3CA, PTEN, MTOR, FOXJ1, FMN2, PTCH1,* and *FXYD2* | - *TRIM71* and *SMARCC1* exhibit genome-wide significant enrichment of de novo mutations, which may be genuine risk factors for CH. - *PIK3CA, PTEN, MTOR, FOXJ1, FMN2, PTCH1,* and *FXYD2* are newly identified high-confidence sporadic CH genes. - *TRIM71* and other genes, reducing neural cell proliferation to cause hydrocephalus, can be a diagnostic marker. | N | Sheng Chih Jin et al.[191] |
| Hydrocephalus  (communicating) | Proteomics | Diagnosis | KLK6 | - Expression of KLK6 is significantly up-regulated in CH patients. - KLK6 is involved in CH development and may provide a new target for CH diagnosis. | Y (Rat Model) | Lei Yuan et al.[192] |
| Hydrocephalus  (idiopathic normal pressure) | Proteomics | Diagnosis | QPCT, RBP4 | - 39 proteins exhibit a significant increase, while 285 proteins show a significant decrease in CSF of iNPH. - Elevated proteins mainly relate to myeloid leukocyte migration and extracellular matrix organization; reduced ones are linked to axon and synaptic development. - QPCT and RBP4 have been identified as potential protein biomarkers in iNPH for predicting shunt outcomes. | N | Yuqi Ying et al.[193] |
| Hydrocephalus  (idiopathic normal pressure) | Proteomics | Diagnosis | PTPRQ | - PTPRQ concentrations in CSF are significantly higher in iNPH patients than in AD patients. - PTPRQ concentration in the CSF of non-responders to shunt operation tended to be relatively lower compared with that in the responders. - PTPRQ is a candidate biomarker to distinguish iNPH from AD. | N | Yuki Nagata et al.[194] |
| Hydrocephalus  (idiopathic normal pressure) | Metabolomics | Diagnosis | Glyceric acid, N-acetyl neuraminic acid, serine, and 2-hydroxybutyric acid | - Elevated glyceric acid and N-acetyl neuraminic acid, and reduced serine and 2-hydroxybutyric acid in AD CSF distinguish it from iNPH. - Serine, glyceric acid, Neu5Ac, and 2-hydroxybutyrate combine as a diagnostic iNPH biomarker. | N | Yuki Nagata et al.[195] |
| Hydrocephalus  (normal pressure) | Metabolomics | Treatment | Neu5Ac | - CSF Neu5Ac levels are low in NPH patients. - Boosting brain Neu5Ac inhibits astrocyte activation. - Brain Neu5Ac elevation reduces periventricular demyelination and improves hydrocephalus. - Enhanced brain Neu5Ac improves neurological outcomes in NPH, suggesting a potential treatment. | Y (Mouse Model) | Zhangyang Wang et al.[196] |
| Hydrocephalus  (tumor-associated) | SnRNA-seq, Spatial transcriptomics | Diagnosis/Treatment | *CPMCs* | - Ventricular cell atlas reveals *CPMC* expansion in TAH mice. - *CPMCs* compromise ependymal ciliary integrity via tryptase-PAR2-FOXJ1 signaling, triggering pathological CSF hypersecretion that underlies hydrocephalus pathogenesis. - Brain barrier-penetrating trypsin-like inhibitor BMS-262084 effectively inhibits TAH progression in vivo and attenuates mast cell-induced epithelial cilia damage. | N | Yiye Li et al.[197] |
| Hydrocephalus | Genomics,  scRNA-seq | Diagnosis | *MAEL* | - scRNA-seq data from the cortical plate and germinal matrix revealed robust *MAEL* expression within neurogenic niches. - Reduced *MAEL* levels may induce genomic structural alterations, thereby impairing cortical development, volume, and function. - Decreased *MAEL* expression is associated with the pathogenesis of hydrocephalus. | Y (Independent Cohort) | Hale, AT et al.[198] |
| Hydrocephalus  (communicating) | Genomics,  Proteomics, Transcriptomics | Diagnosis | *MAEL* | - PrediXcan analysis in 10 neuro tissues and whole blood shows a correlation between reduced *MAEL* gene expression in the brain and hydrocephalus (p < 0.05). - Reduced *MAEL* expression increases susceptibility to hydrocephalus. - *MAEL* is a diagnostic biomarker for hydrocephalus. | Y (Mouse Model) | Andrew T Hale et al.[199] |
| Hydrocephalus  (post-hemorrhagic) | Proteomics, Metabolomics | Diagnosis/Treatment | CSPG4 | - CSPG4 positively correlates with ventricular size and the incidence of periventricular leukomalacia. - Silencing of CSPG4 can inhibit ferroptosis, cell adhesion functions, and intracellular Ca2+ flux. - CSPG4 has been identified as a CSF biomarker and effective therapeutic target. | Y (Mouse Model) | Juncao Chen et al.[200] |

NPH: normal pressure hydrocephalus; iNPH: idiopathic normal pressure hydrocephalus; CH: communicating hydrocephalus; *Neu5Ac*: N-acetylneuraminic acid; TAH: tumor-associated hydrocephalus; *CPMCs*: choroid plexus mast cells; MAEL: maelstrom spermatogenic transposon silencer; KLK6, kallikrein-6; *QPCT*: glutaminyl-peptide cyclotransferase; RBP4: retinol-binding protein 4; PTPRQ: Q-type protein tyrosine phosphatase receptor; CSPG4: chondroitin sulfate proteoglycan 4.
