# Supplementary material for: Multi-omics and high-spatial-resolution omics: deciphering complexity in neurological disorders
Source: Gigascience. 2025 Dec 5;14:giaf137. doi: 10.1093/gigascience/giaf137 (PMC12723665; doi:10.1093/gigascience/giaf137)
Supplement: giaf137_GIGA-D-25-00354_Original_Submission [file giaf137_giga-d-25-00354_original_submission.pdf]

GigaScience

Multi-Omics and High-Spatial-Resolution Omics: Deciphering Complexity in Neurological Disorders

--Manuscript Draft--

**Manuscript Number:**	GIGA-D-25-00354
**Full Title:**	Multi-Omics and High-Spatial-Resolution Omics: Deciphering Complexity in Neurological Disorders
**Article Type:**	Review
**Funding Information:**	Scientific Research Innovation Capability Support Project for Young Faculty (ZYGXQNJSKYCXNLZCXM-H15) — PhD Xiuyun Liu<br>National Science Fund for Excellent Overseas Scholars (0401260011) — PhD Xiuyun Liu<br>National Natural Science Foundation of China (82472098) — PhD Xiuyun Liu<br>National Natural Science Foundation of China (32300704) — PhD Xiuyun Liu<br>Tianjin Natural Science Foundation-Outstanding Youth Project (24JCJQJC00250) — PhD Xiuyun Liu<br>Major Science and Technology Special Projects and Engineering-Major Project of National Key Laboratories (24ZXZSSS00510) — PhD Xiuyun Liu<br>Key Technologies Research and Development Program (2021YFF1200602) — PhD Xiuyun Liu<br>the Non-profit Central Research Institute Fund of Chinese Academy of Medical Sciences (2024-JKCS-16) — PhD Xiuyun Liu
**Abstract:**	The world has witnessed a steady rise in neurological diseases, which represent a heterogeneous group of disorders characterized by complex pathogenesis involving disruptions at multiple molecular levels, including genomic, transcriptomic, proteomic, and metabolomic levels. These disorders, often caused by genetic mutations, metabolic imbalances, immune dysregulation, and environmental factors, pose significant challenges to global public health due to their high prevalence, mortality, and disability burden. The advent of high-throughput technologies, such as next-generation sequencing and mass spectrometry, has provided valuable insights into the underlying mechanisms of disease, especially the development of multi- and high-spatial-resolution omics technologies, enabling the interaction of multiple levels of biology and analysis of the complex molecular networks and pathophysiological processes. This review provides a comprehensive analysis of the latest advancements in multi- and high-spatial-resolution omics, with a focus on their applications in precision diagnostics, biomarker discovery, and therapeutic target identification in brain diseases. The study also highlights the current challenges in the clinical implementation and discusses the future directions, with artificial intelligence being anticipated to enhance clinical translation and diagnostic accuracy significantly.
**Corresponding Author:**	Xiuyun Liu, Ph.D<br>Tianjin University<br>Tianjin, Tianjin CHINA
**Corresponding Author Secondary Information:**	
**Corresponding Author's Institution:**	Tianjin University
**Corresponding Author's Secondary**	

Powered by Editorial Manager® and ProduXion Manager® from Aries Systems Corporation

Institution:	
First Author:	Xiuyun Liu, PhD
First Author Secondary Information:	
Order of Authors:	Xiuyun Liu, PhD
	Fangfang Li, PhD
	Marek Czosnyka, PhD
	Zofia Czosnyka, PhD
	Huijie Yu, PhD
	Xiaoguang Tong, PhD
	Yan Xing, PhD
	Hongliang Li, PhD
	Ke Pu, PhD
	Keke Feng, PhD
	Kuo Zhang, PhD
	Meijun Pang, PhD
	Dong Ming, PhD
Order of Authors Secondary Information:	
Additional Information:	
Question	Response
Are you submitting this manuscript to a special series or article collection?	No
**Experimental design and statistics**<br><br>Full details of the experimental design and statistical methods used should be given in the Methods section, as detailed in our Minimum Standards Reporting Checklist. Information essential to interpreting the data presented should be made available in the figure legends.<br><br>Have you included all the information requested in your manuscript?	No
If not, please give reasons for any omissions below.<br><br>as follow-up to "**Experimental design and statistics**<br><br>Full details of the experimental design and	This is a review paper

statistical methods used should be given in the Methods section, as detailed in our Minimum Standards Reporting Checklist. Information essential to interpreting the data presented should be made available in the figure legends.<br><br>Have you included all the information requested in your manuscript?<br><br>"	
**Resources**<br><br>A description of all resources used, including antibodies, cell lines, animals and software tools, with enough information to allow them to be uniquely identified, should be included in the Methods section. Authors are strongly encouraged to cite Research Resource Identifiers (RRIDs) for antibodies, model organisms and tools, where possible.<br><br>Have you included the information requested as detailed in our Minimum Standards Reporting Checklist?	No
If not, please give reasons for any omissions below.<br><br>as follow-up to "**Resources**<br><br>A description of all resources used, including antibodies, cell lines, animals and software tools, with enough information to allow them to be uniquely identified, should be included in the Methods section. Authors are strongly encouraged to cite Research Resource Identifiers (RRIDs) for antibodies, model organisms and tools, where possible.<br><br>Have you included the information requested as detailed in our Minimum Standards Reporting Checklist?	This is a review paper, summarizing previous publication results

"	
**Availability of data and materials**<br><br>All datasets and code on which the conclusions of the paper rely must be either included in your submission or deposited in publicly available repositories (where available and ethically appropriate), referencing such data using a unique identifier in the references and in the "Availability of Data and Materials" section of your manuscript.<br><br>Have you have met the above requirement as detailed in our Minimum Standards Reporting Checklist?	Yes
GigaScience has policies and guidelines in place for the use of generative AI-writing tools such as ChatGPT. If you have used such writing tools to assist with writing the manuscript this must be declared and cited in the text. Authors should not list AI-writing tools and other AI-assisted technologies as an author or co-author and should acknowledge that they are fully responsible for text generated or refined by AI-writing tools.<p><br><br>A summary of use (particularly in the introduction or among methods) needs to be included at the end of the paper, and the outputs should also be included as a supplementary file hosted in GigaDB or other open repositories. Please read our guidelines for more information. <p><br><br>By submitting to GigaScience, you are aware of the journal's AI-writing tools policy, and if you have declared use of such tools below, you have acknowledged this where appropriate in your manuscript and have made a summary of use and outputs available. <p><br><b>AI-assisted writing tools have been used in the preparation of this manuscript?	No

Manuscript Click here to access/download;Manuscript;Manuscript .docx

Multi-Omics and High-Spatial-Resolution Omics: Deciphering Complexity in Neurological Disorders

Xiuyun Liu, PhD [1,2,3#], Fangfang Li, MD [1#], Marek Czosnyka, PhD [4], Zofia Czosnyka, PhD [4], Huijie Yu, PhD [5], Xiaoguang Tong, PhD [6], Yan Xing, PhD [7], Hongliang Li, PhD [7], Ke Pu, PhD [6], Keke Feng, PhD [6], Kuo Zhang, PhD[1,2], Meijun Pang, PhD [1,2*], and Dong Ming, PhD [1,2*]

[1] State Key Laboratory of Advanced Medical Materials and Devices, Medical School, Tianjin University, Tianjin, 300072, China.

[2] Haihe Laboratory of Brain-Computer Interaction and Human-Machine Integration, Tianjin, 300380, China.

[3] School of Pharmaceutical Science and Technology, Tianjin University, Tianjin, 300072, China.

[4] Department of Clinical Neurosciences, Addenbrooke's Hospital, University of Cambridge, Cambridge, CB2 0QQ, UK.

[5] Department of Neurosurgery, Tianjin Medical University General Hospital, Tianjin, 300052, China.

[6] Department of Neurosurgery, Tianjin Huanhu Hospital, Tianjin, 300350, China.

[7] Department of Neurology, Aviation General Hospital, 100012, Beijing, China.

[#] These authors contributed equally to this work.

* Corresponding authors:

Meijun Pang, State Key Laboratory of Advanced Medical Materials and Devices, Medical School, Tianjin University, 92 Weijin Road, Nankai District, Tianjin, 300072, China, Email: meijun.pang@tju.edu.cn

Dong Ming, State Key Laboratory of Advanced Medical Materials and Devices, Medical School, Tianjin University, 92 Weijin Road, Nankai District, Tianjin, 300072, China, Email: richardming@tju.edu.cn

Multi-omics technologies integration

Abstract

Background

The world has witnessed a steady rise in neurological diseases, which represent a heterogeneous group of disorders characterized by complex pathogenesis involving disruptions at multiple molecular levels, including genomic, transcriptomic, proteomic, and metabolomic levels. These disorders, often caused by genetic mutations, metabolic imbalances, immune dysregulation, and environmental factors, pose significant challenges to global public health due to their high prevalence, mortality, and disability burden.

Results

The advent of high-throughput technologies, such as next-generation sequencing and mass spectrometry, has provided valuable insights into the underlying mechanisms of disease, especially the development of multi- and high-spatial-resolution omics technologies, enabling the interaction of multiple levels of biology and analysis of the complex molecular networks and pathophysiological processes.

Conclusions

This review provides a comprehensive analysis of the latest advancements in multi- and high-spatial-resolution omics, with a focus on their applications in precision diagnostics, biomarker discovery, and therapeutic target identification in brain diseases. The study also highlights the current challenges in the clinical implementation and discusses the future directions, with artificial intelligence being anticipated to enhance clinical translation and diagnostic accuracy significantly.

Keywords: Multi-Omics, Neurological Diseases, Single-Cell Omics, Spatial Transcriptomics

Highlights

- 43% of the global population is suffering from neurological disorders, yet diagnostic and therapeutic strategies remain limited, primarily due to the complexity involving disruptions at multiple molecular levels, including genomic, transcriptomic, proteomic, and metabolomic levels.
- Rapid advances in multi- and high-spatial-resolution omics enable the interaction of multiple levels of biology, showing great potential in the analysis of the complex molecular networks and pathophysiological processes of brain disease.
- This review provides a comprehensive analysis of the latest advancements in multi- and high-spatial-resolution omics, with a focus on their applications in precision diagnostics, biomarker discovery, and therapeutic target identification in brain diseases.

Introduction

Neurological brain diseases encompass a wide range of brain disorders that affect the structure or function of the central and peripheral nervous systems. Globally, an estimated 43% of the world population suffers from neurological diseases, becoming the leading cause of overall disease burden in the world[1]. New treatments that can completely resolve brain diseases have yet to be discovered, due to the blood-brain-barrier (BBB), unclear disease mechanisms, and limited brain analysis tools. Conventional approaches for brain imaging, such as MRI or CT, provide us with abundant information on brain structure and function, but they lack interpretation at the cellular level. Developing advanced analytical technologies is essential for unraveling the complexities of the brain and detecting disease-related therapeutic targets for early diagnosis and precise medicine[2-4].

Since the concept of genomics was first proposed by Thomas H. Roderick in 1986[5], omics technologies, such as genomics, proteomics, metabolomics, lipidomics, glycomics, and transcriptomics, have gained rapid development[6]. However, basic-omics, which employs a singular approach to elucidate the isolated function of a single molecule of a biological system, provides information that is highly fragmented and limited. As the foundation for all living organisms, understanding the complex process by which genetic information is transformed into functional proteins, including transcriptional regulation, translational regulation, RNA/polymer degradation, post-translational modification, and differential transport, plays a critical role in revealing the basic mechanism of brain diseases and discovering new targets[7]. Multiple omics, which integrates basic-omics technologies and combines diverse omics data to extract meaningful information, enables a systematic analysis of the mechanisms and phenotypes of complex biological

processes and advances our understanding of the regulatory relationships among various molecules[6,8-10].

In recent years, the development of high-spatial-resolution (single-cell and spatial) omics technologies has significantly advanced the study of neurological brain diseases. These technologies allow the detailed profiling of molecular changes at the single-cell and tissue-level spatial resolution, thereby uncovering the heterogeneity of disease mechanisms and revealing localized pathophysiological alterations[11]. Single-cell omics provides insights into cellular diversity and gene expression dynamics[12], while spatial omics preserves tissue architecture to map molecular events in their anatomical context[13]. The integration of multi-omics with high-spatial-resolution omics further enhances the biological relevance and depth of disease characterization, offering a more comprehensive view of neurodegenerative and inflammatory brain disorders.

This article provides a systematic review of multi-omics and high-spatial-resolution omics techniques and their application in the neurology field for brain disease diagnosis and treatment. We also discussed the current challenges and prospects, aiming to offer researchers a comprehensive and evidence-based perspective on the development, utility, and translational potential of multi- and high-spatial-resolution omics approaches in the neuroscience area.

2. Omics Technology

2.1 Basic Omics Technology

Different basic omics technologies, including genomics, transcriptomics, proteomics, and metabolomics, have been developed for neuroscience researchers, with distinct advantages and disadvantages shown in Table 1.

Multi-omics technologies integration

2.1.1 Genomics

Genomics investigates the entirety of an organism's genetic material, encompassing DNA sequences, gene repertoire, and regulatory elements. It emphasizes the holistic analysis of genome architecture, functionality, evolution, and regulation, utilizing high-throughput sequencing and bioinformatics for comparative genomic data analysis across individuals and species[8].

Genomic research leverages advanced technologies such as next-generation sequencing, CRISPR-mediated gene editing, and genome-wide association studies (GWAS) to explore genetic variations and their influence on disease susceptibility and progression. GWAS, specifically, employ statistical models to detect associations between genetic variants and phenotypic traits across large populations, providing a robust framework for elucidating the genetic underpinnings of complex neurological conditions[14]. In the realm of neurological brain disorders, genomics has been pivotal in pinpointing risk loci, clarifying disease mechanisms, and guiding the development of targeted therapies and precision medicine strategies[15]. Nevertheless, this field is still facing several challenges, including difficulty in interpretation for non-coding variations, challenges in clinical translation, etc.

2.1.2 Transcriptomics

Transcriptomics, developed by Charles Auffray in 1999, systematically investigates RNA transcripts and their regulatory networks to unravel the molecular underpinnings of cellular function and disease [16,17].

By utilizing high-throughput RNA sequencing (RNA-seq) and other cutting-edge technologies to profile the transcriptome in specific cell types or tissues, transcriptomics facilitates the identification of gene expression patterns, alternative splicing events, and the role of non-coding

RNAs in the progression of neurological diseases. Transcriptomic studies encounter challenges including spatial and temporal heterogeneity of RNA expression in complex tissues[18,19], instability of RNA, and the lack of mature analytical methods.

2.1.3 Proteomics

Proteomics, introduced by Marc Wilkins in 1994, has been widely used to investigate the protein composition and its dynamic changes in cells, tissues, or organisms, serving as a crucial tool for exploring the structure and function of proteins[20]. It serves as a powerful complement to genomics and transcriptomics, encompassing protein identification, comparative proteomics, glycomics, targeted proteomics, etc., which have been applied in new drug development and synthetic biology.

The most prevalent analysis methods for proteomics include the data-dependent acquisition (DDA) mode, also known as the "shotgun" approach; isobaric tags for relative and absolute quantification (iTRAQ)[21]; tandem mass tag (TMT) technology[22]; stable-isotope labeling by amino acids in cell culture (SILAC)[23]; label-free quantification[24]; and data-independent acquisition[25] especially the data independent acquisition-sequential window acquisition of all theoretical mass spectral approach (DIA/SWATH), with their advantages and disadvantages depicted in Table 2. Additionally, mass spectrometry-based sequencing is used to identify and analyze post-translational modifications (PTMs)[26]. These technologies enable proteomics to be an indispensable tool for brain disease diagnosis and treatment, which offers crucial insights into the molecular pathophysiology underlying neurological disorders.

2.1.4 Metabolomics

Introduced by Nicholson et al. in 1999, metabolomics investigates the metabolic responses of

living organisms to exogenous stimuli, environmental changes, or genetic modifications, mapping out comprehensive dynamic profiles of metabolite alterations[27]. As a relatively recent addition to the omics framework following genomics and proteomics, metabolomics aims to characterize small molecules with molecular weights typically ranging from 100 to 1000 Da, either qualitatively or quantitatively, to uncover their functional roles in health and disease[28].

Metabolomic analysis employs a variety of analytical platforms, including nuclear magnetic resonance (NMR), Fourier-transform infrared (FT-IR) spectroscopy, gas chromatography-mass – mass spectrometry (GC–MS), and liquid chromatography-mass spectrometry (LC-MS), which provide high-throughput, high-spatial-resolution, and sensitive detection of metabolite profiles. Currently, metabolomics can be broadly classified into three main approaches based on detection principles: untargeted metabolomics[29], targeted metabolomics[30], and widely targeted metabolomics[31]. Untargeted metabolomics offers broad coverage of the metabolome but at the expense of precision. In contrast, targeted metabolomics enables precise quantification of a predefined set of metabolites, though it limits the discovery of novel biomarkers. Widely targeted metabolomics, meanwhile, strikes a balance between breadth and specificity, allowing for both comprehensive detection and reliable quantification of a larger subset of known metabolites. As illustrated in Figure 1, these methodologies collectively support the exploration of metabolic alterations in complex diseases.

In the context of neurological brain diseases, metabolomics has demonstrated significant potential in identifying disease-specific metabolite signatures that can be employed as diagnostic or prognostic biomarkers[28]. It provides a functional readout of biological processes and has been increasingly used to detect early metabolic changes, assess disease progression, and guide

therapeutic interventions. However, several challenges remain. First, the comprehensive understanding of the metabolome is still in its infancy, with less than 5% of detected metabolites currently annotated. Second, the dynamic and context-dependent nature of metabolic profiles introduces complexity in data interpretation. Third, technical variability across platforms and sample types can hinder reproducibility and comparability. Despite these limitations, ongoing advancements in analytical technologies and bioinformatic tools are expected to position metabolomics as a powerful and indispensable strategy for more precise and efficient diagnosis of neurological disorders.

2.2 Single-Cell Omics Technology

First introduced in 2009 by Tang et al. for RNA-seq at the single-cell level[32], single-cell omics has evolved into a high-throughput platform for analyzing genomic[33-35], transcriptomic, epigenomic[34], and proteomic profiles with cellular resolution[36]. Unlike bulk sequencing, which provides averaged signals across cell populations, single-cell approaches enable the deconvolution of individual cellular states, revealing heterogeneity with unprecedented precision. Single-cell transcriptomics remains the most mature and widely applied modality, having advanced from low-throughput methods to high-throughput platforms capable of profiling millions of cells. Representative technologies include full-length amplification (e.g., SMART-seq), high-throughput barcoding (e.g., 10× Genomics), and multi-omics compatible methods (e.g., Andeplete)[37]. Key steps in the workflow encompass cell isolation, nucleic acid amplification, sequencing, and computational analysis, with isolation and amplification being particularly critical for data quality. These technologies are increasingly integrated with spatial and epigenomic approaches to provide comprehensive insights into gene regulation, cellular function, and disease mechanisms. Despite

significant progress, challenges such as amplification bias, cell dropout, and data standardization remain, necessitating continued methodological innovation.

2.3 Spatial Omics Technology

First introduced in 2016 by Joakim et al. for in situ RNA capture, it represents a significant advancement in molecular biology by enabling the profiling of transcriptomic information while preserving spatial context within intact tissue[38]. As illustrated in Figure 2, unlike single-cell sequencing, which provides molecular resolution but loses spatial information, spatial omics allows the mapping of gene expression, epigenetic modifications, protein localization, and metabolic profiles to precise anatomical coordinates, offering a more comprehensive view of tissue organization and function[39]. The field has developed multiple modalities, including spatial transcriptomics[40], (epi) genomics[41], proteomics[42], and metabolomics[43]. Among these, spatial transcriptomics is the most mature, utilizing either in situ hybridization techniques (e.g., MERFISH, seqFISH) for subcellular-resolution transcript mapping or sequencing-based methods (e.g., Visium, Slide-seq) that spatially barcode RNA through microarray capture or hybridization-based imaging[44-46]. These technologies are increasingly being applied in tumor microenvironment characterization, neural development studies, inflammatory disease modeling, and organoid/tissue engineering to uncover spatially regulated gene expression patterns and their functional implications. Despite its potential, spatial omics still faces challenges such as limited resolution, high cost (often exceeding $1,500 per sample), and computational demands in 3D spatial data integration. Future directions will focus on improving resolution, reducing cost, enhancing data analysis tools, and promoting multi-omics integration to better elucidate the complex regulatory networks governing tissue biology and disease progression.

3. Application of Multi-Omics Technologies in the Diagnosis or Treatment of Chronic Neurological Diseases

Neurological diseases constitute a major global health challenge, including but not limited to Alzheimer's disease (AD), Parkinson’s disease (PD), epilepsy, multiple sclerosis (MS), stroke, hydrocephalus, and various neurological diseases[47]. With the rapid advancement of science and technology, multi-omics approaches have become increasingly pivotal in the research of neurological diseases. As shown in Figure 3, by integrating multi-layered data, researchers can delve deeper into the molecular mechanisms underlying these diseases, thereby offering novel perspectives and methods for clinical diagnosis, treatment, and prevention. This review provides a comprehensive overview of the advancements made in the application of multi-omics technologies over the past five years in the study of representative neurological diseases.

3.1 Application of Multi-Omics Technologies in AD

AD, commonly referred to as senile dementia, is one of the most prevalent neurodegenerative disorders to date and a leading cause of disability and death among the elderly[48,49]. The disease typically begins insidiously and progresses irreversibly, leading to severe cognitive impairment[50-52]. However, there is still no effective treatment for AD, and the underlying mechanisms contributing to the pathogenesis of AD remain incompletely elucidated, although amyloid plaques and tau neurofibrillary tangles have revealed major pathological changes in AD[53,54]. These pathological changes may trigger synaptic dysfunction, neuroglial inflammation, and eventually neuronal loss in the cerebral cortex, subcortical regions, temporal lobe, parietal lobe, and cingulate gyrus[55-57], and may even affect the gut microbiome[47,58]. The most critical challenge in AD prevention and treatment is the difficulty in diagnosing the disease at its early stage. By the time

clinical symptoms become apparent, various patients are already in a moderate-to-severe phase, at which point the disease is largely irreversible[59]. Therefore, the identification of preclinical molecular and biochemical changes is essential for transforming AD into a preventable and treatable condition[60].

3.1.1 Basic Omics Approaches in AD

Basic-omics approaches have provided valuable, unique insights into different aspects of AD pathogenesis (Supplementary Table S1). Genomic studies have identified high-risk genes and epigenetic modifications associated with AD, including APP, PSEN1, Tau, APOE4, acetylation status of histone H4 at lysine 16 (H4K16ac), and H3K9ac. Among these, APOE4 is particularly well-characterized in its association with late-onset AD, as it accelerates vascular dysfunction, disrupts the BBB, and promotes neuronal degeneration. These effects position APOE4 as a central player in AD's vascular and neurodegenerative mechanisms, making it a key diagnostic and prognostic marker[61].

Epigenomic analyses have revealed the influence of single-nucleotide polymorphisms (SNPs) in Tau and enhancer regions on chromatin structure and disease progression. In particular, the H4K16ac is significantly altered with aging and AD-related gene expression. Compared to non-AD elderly individuals, 25,000 peaks show loss of H4K16ac, whereas 9,000 exhibit increased acetylation in AD patients. These changes suggest that H4K16ac may serve as an epigenetic link between aging and AD pathology, and as a potential diagnostic biomarker[62]. Similarly, H3K9ac and Tau have been identified as epigenetic and proteomic biomarkers, with H3K9ac showing parallel acetylation patterns in Tau-related contexts, indicating a complex interplay between Tau pathology and chromatin regulation[63].

Multi-omics technologies integration

Transcriptomic profiling has enabled the identification of co-expression modules that are closely linked to AD pathology. One such module, M109, is most directly associated with cognitive decline and amyloid load. Within this module, INPPL1 and PLXNB1 have been identified as potential candidates for in vitro amyloid biology investigation. Notably, INPPL1 and PLXNB1 are associated with extracellular β-amyloid levels in astrocyte cultures, suggesting their relevance in early detection and mechanistic understanding of AD[64].

Proteomic studies have uncovered disease-specific alterations in protein expression. For instance, elevated levels of FYN, YES1, and STAT3 in AD-derived induced neurons are associated with neuroinflammation, tau phosphorylation, and increased amyloid-42 production[65]. Proteomics also found that APP/PS1 and ApoE4 knock-in mice lead to changes in early hippocampal protein expression profiles, and that the changes in hippocampal proteins involved in insulin signaling and the mitochondrial electron transport chain may be key biological processes in AD progression[66]. These findings suggest that proteomics can help distinguish AD subtypes and identify critical pathways in disease progression.

Phosphoproteomic analyses have further highlighted the role of PTMs in AD. Abnormal phosphorylation of GSK3β and PPP3CA in models treated with low-dose copper is linked to mitochondrial dysfunction[67]. A recent study has revealed the staged pathophysiological progression in preclinical autosomal dominant AD via cerebrospinal fluid (CSF) proteome analysis. The six-protein model (GFAP, NPTX2, PEA15, SMOC1, SMOC2, TNFRSF1B) demonstrates high predictive accuracy (AUC > 0.9), validated independently, and offers a valuable tool for ultra-early autosomal dominant AD screening, precise staging, and clinical trial enrollment[68].

Metabolomic research has identified sphingolipids as potential early biomarkers of AD, and

dysregulated amino acid and tryptophan metabolism have also been observed in transgenic models[69]. Furthermore, some investigators unveiled a metabolic shift towards aerobic glycolysis in AD-derived induced neurons, triggered by pyruvate kinase M2 (PKM2)'s loss of metabolic activity, nuclear translocation, and interaction with STAT3 and HIF1α, which collectively precipitate metabolic and transcriptional changes that underlie neuronal identity loss and heightened vulnerability in sporadic AD[70].

3.1.2 High-spatial-resolution Omics Technologies in AD

High-spatial-resolution omics technologies have transformed the understanding of AD by revealing cell-type-specific and spatially resolved molecular alterations[71]. Mathys et al. pioneered the use of single-cell RNA sequencing (scRNA-seq) in AD, profiling ~80,000 prefrontal cortex cells across disease stages and identifying myelination maintenance as a critical regulatory response, especially the relevant gene LINGO1[72]. Grubman et al. further analyzed the entorhinal cortex, an early AD-affected brain region, and found that astrocytes exhibited distinct activation patterns compared to other areas. Expression changes of multiple AD risk genes (e.g., APOE, TREM2) were also validated in specific cell types. For example, APOE expression was elevated in microglia and astrocytes, correlating with tau pathology severity, offering cell-specific insights into genetic risk mechanisms[73].

Spatial transcriptomics has further advanced AD research by preserving spatial context during transcriptome profiling. In AD mouse models, this method detected early transcriptional dysregulation in 100-μm plaque microenvironments, particularly in myelination-related and oligodendrocyte gene networks. Late-stage spatial profiles revealed 57 plaque-induced genes (PIGs) enriched in complement activation, oxidative stress, lysosomal dysfunction, and

neuroinflammation[74]. Integration of 10× Genomics Visium spatial transcriptomics with co-immunofluorescence mapping in human middle temporal gyrus identified five layer- and white matter-specific marker genes, including both established (RORB, PCP4, MBP) and novel (SPARC, CALB2, DIRAS2, KRT17) candidates with potential as diagnostic and mechanistic biomarkers[75].

Integrative omics strategies combining single-cell and spatial transcriptomics are beginning to illuminate intercellular communication in AD pathology. A recent study identified a PTPRG-expressing microglial subpopulation that promotes neuronal VIRMA expression, which enhances m^6A modification of PRKN transcripts, leading to RNA destabilization, impaired mitophagy, and neuronal death. These findings highlight PTPRG and VIRMA as promising therapeutic targets for AD[76].

3.1.3 Multi-Omics Integration in AD

Multi-omics integration has enabled the identification of disease-specific molecular modules and biomarker signatures that reflect the underlying pathophysiology of AD[77] (Supplementary Table S1). For example, combined proteomic and transcriptomic analyses have revealed two distinct AD-related modules: the MAPK/metabolic module, which is associated with the rate of cognitive decline, and the matrix body (matrixsome) module, whose expression is modulated by the APOE ε4 allele[78]. Further integration of proteomic and transcriptomic data has identified FBP1, FBP2, RHOH, JPH2, ERAP2, and SCLT1 as upregulated proteins in APOE4 carriers compared to controls. In contrast, the Myeloid basic protein encoding gene (MBP) is among the top-ranked candidate genes that reinforce the importance of myelination in AD pathogenesis and cognitive decline. Notably, these biomarkers show consistent expression patterns in both plasma and brain tissue[79]. A multi-omics approach integrating genomics, transcriptomics, proteomics, and metabolomics has

identified ABCA1, CPT1A, adiponectin, and NGAL as key players in the regulation of acylcarnitines and amino acid metabolism. Disruptions in the homeostasis of short-chain acylcarnitines and essential amino acids are closely correlated with disease severity, suggesting that these molecules may serve as early diagnostic indicators[80]. In addition, IVD, CYFIP1, and ADD2 have been identified as serum-based diagnostic biomarkers through proteomic and transcriptomic analysis. IVD shows significantly higher protein abundance in AD patients, whereas CYFIP1 and ADD2 are downregulated. The combination of these three proteins improves early detection and disease discrimination[81]. From a lysosomal perspective, multi-omics integration has revealed that CSTD, CTSB, CTSD, and GM2A are significantly upregulated in AD patients and have been validated as CSF and plasma biomarkers. These lysosomal proteins show progressive fold changes during AD progression, further supporting their diagnostic utility[82]. Moreover, PBXIP1 has been identified as a multi-omics-linked diagnostic target through genomics, transcriptomics, and proteomics. PBXIP1-encoded proteins are significantly associated with all three neuropathological hallmarks of AD-amyloid-β plaques, neurofibrillary tangles, and neurodegeneration. It functions in hippocampal neurons, astrocytes, and mTOR signaling, linking it to both neuropathology and cognitive dysfunction[83]. Epigenomic and proteomic integration has further revealed H3K27ac as a potential diagnostic marker, particularly in the entorhinal cortex. Analysis has shown that AD risk variants are significantly enriched in H3K27ac peak regions, including CR1, GPR22, KMO, PIM3, PSEN1, and RGCC[84]. Additionally, integrative studies using transcriptomics, proteomics, and epigenomics have demonstrated that H3K27ac and H3K9ac, transcriptionally active post-PTMs, are genome-wide dysregulated in AD, with RNA-seq revealing increased expression of histone acetyltransferases. These modifications are enriched in amyloid-β and tau-related pathways, and

their dysregulation contributes to transcriptional and chromatin dysfunction[85]. These epigenetic alterations may serve as early indicators of AD-related gene expression and chromatin dysregulation. Pathway-level integration of proteomics, metabolomics, and lipidomics has identified gender-dependent effects in GABA synthesis, arginine biosynthesis, and alanine/aspartate/glutamate/arginine metabolism. These findings emphasize the importance of lysophospholipid and amino acid metabolism in the AD brain and suggest the need for gender-specific diagnostic models[86]. Finally, four CSF proteins-14-3-3 zeta/delta, clusterin, interleukin-15, and transgelin- 2 -have been shown to enhance AD prediction accuracy through multi-omics integration[87].

From a therapeutic perspective, multi-omics integration has revealed potential drug targets and intervention points (Supplementary Table S1). One such target is TRPV1, a pharmacologically modifiable receptor that, when activated, rescues memory deficits and neuronal loss in ApoE4 mice on a high-fat diet. These results highlight the potential of TRPV1-based therapies in mitigating AD pathology[88]. In late-onset AD, ATP6V1A has been identified as a key regulator of the most dysregulated neuronal subnetwork. Targeting ATP6V1A has shown therapeutic potential, as NCH-51, a compound targeting this pathway, ameliorates neuronal damage in Drosophila models[89].

Several computational platforms have been developed to facilitate the integration and interpretation of multi-omics data in AD. For instance, the Alzheimer's Disease Genome-Wide Positioning Systems platform (AlzGPS) is used for drug discovery by mining AD-related targets and clinically relevant candidate drugs[90].

Taken together, these studies underscore the importance of integrating multi-omics data to elucidate the associations between brain functional and structural changes related to AD. The integration of multiple omics layers provides a comprehensive and mechanistically grounded view

of the disease, offering a solid theoretical foundation for the development of precision diagnostics and targeted therapies. A detailed diagram illustrating the multi-omics-driven pathogenesis of AD is shown in Figure 4.

3.2 Application of Multi-Omics Technologies in PD

PD is the second most common chronic neurodegenerative disorder globally, following AD, affecting approximately 1% to 2% of the population. It is characterized by resting tremor, increased muscle tone (rigidity or stiffness), bradykinesia (slowness of movement), and postural instability, with numerous patients also experiencing cognitive impairments or dementia[4,91,92]. The disease primarily impacts the motor system[93,94] and often has an insidious onset with early diagnosis lacking distinct features[47]. The BBB is disrupted in PD disease, and leukocytes and neutrophils enter the brain and release large amounts of inflammatory cytokines, such as tumor necrosis factor-α (TNF-α), interleukin-1β (IL-1β), and interleukin-6 (IL-6)[95]. The accumulation of α-synuclein in Lewy bodies and Lewy neurites, predominantly in the substantia nigra, leads to the loss of dopaminergic neurons and the manifestation of prominent symptoms. Lewy bodies may also be associated with AD, complicating the determination of PD's etiology and pathogenesis. At present, PD diagnosis depends mainly on clinical evaluation and neuroimaging, but these methods are subject to diagnostic delays and misclassification, especially in the early stages. Given the clinical and pathological heterogeneity in PD, spanning genetic basis, biomarker profiles, progression patterns, and treatment responses, there is a growing need for more accurate, early, and mechanistically grounded diagnostic tools, such as those derived from multi-omics approaches.

3.2.1 Basic-Omics Approaches in PD

Basic-omics approaches have yielded a range of candidate biomarkers and pathogenic insights

in PD (Supplementary Table S2). Genomic studies have identified several high-risk loci and genes associated with PD, including leucine-rich repeat kinase 2 (LRRK2), IL1R2, ZNF184, PARK16, ITPKB, HLA, MAPT, TRIM10, and SETD1A[96,97]. Allelic variations in LRRK2 and IL1R2 significantly increase PD risk, while HLA and MAPT loci are repeatedly implicated in disease susceptibility. Most of these genes are involved in autophagy and lysosomal function-related pathways, which are critical for the clearance of misfolded α-synuclein and other toxic proteins[97].

Transcriptomic profiling has revealed dysregulated gene expression patterns in PD. Signal sequence receptor subunit 1 (SSR1), a gene encoding a mitochondrial protein, is upregulated in PD patients and negatively correlated with dopaminergic neuron survival. Notably, SSR1 is upregulated in peripheral blood before the onset of motor symptoms, suggesting its potential as an early diagnostic marker. A machine learning (ML)-based random forest (RF) classifier incorporating SSR1 expression achieves a high diagnostic accuracy (AUC = 0.91), reinforcing its value in PD detection[98].

Proteomic studies have identified several disease-associated proteins and inflammatory signatures in PD. OMD, CD44, VGF, PRL, and MAN2B1 show significant expression changes in PD patients and are strongly correlated with clinical scores, including motor severity and disease progression[99]. In addition, LRRK2 carriers exhibit enhanced neuroinflammatory profiles, further supporting its pathogenic and diagnostic relevance. These proteins are considered potential biomarkers for PD diagnosis.

Metabolomic analyses have revealed widespread metabolic dysregulation in PD, particularly in lipid and energy metabolism. Alterations in carnitine shuttle, sphingolipid metabolism, arachidonic acid metabolism, and fatty acid biosynthesis are consistently observed, with carnitine

shuttle activity being most significantly perturbed in unmedicated PD patients[100].

Additionally, short-chain fatty acids, including butyric acid, are significantly reduced and correlated with cognitive decline and motor dysfunction[101]. A comprehensive urinary metabolomic profile has identified 139 differentially regulated metabolites, with proline among the most predictive[102]. Moreover, phenylacetic acid, phenylacetylglutamine, histidine, uric acid, and imidazoleacetic acid are consistently upregulated in urine and show strong diagnostic power in early-stage PD, with a 45-metabolite model achieving high accuracy[103]. Using the same technique, this team also found that 18 differential metabolites changed metabolic pathways related to branched-chain amino acid (BCAA) metabolism, glycine derivatives, steroid hormone biosynthesis, tryptophan metabolism, and phenylalanine metabolism, confirming the team's earlier findings[104].

3.2.2 High-spatial-resolution Omics Technologies in PD

Semra et al. employed single-nucleus RNA sequencing (snRNA-seq) and GWAS to profile midbrain cell contributions, identifying a PD-specific neuronal cluster overexpressing CADPS2 and showing reduced tyrosine hydroxylase levels. Glial populations in affected regions exhibited disease-restricted proliferation, along with dysregulation in unfolded protein response and cytokine signaling pathways, while reactive astrocytes showed CD44 upregulation and microglia displayed a pro-inflammatory trajectory marked by elevated IL-1B, GPNMB, and HSP90AA1. These results position IL-1B, GPNMB, and HSP90AA1 as possible diagnostic biomarkers for PD (105). A recent scRNA-seq study integrated with gene set enrichment analysis provided the first single-cell resolution gene atlas of the dorsolateral prefrontal cortex in PD, revealing transcriptomic changes across microglia, astrocytes, oligodendrocytes, and oligodendrocyte precursor cells. Key findings

include mitochondrial dysfunction, immune dysregulation, and impaired protein folding in glial cells, underscoring their roles in disease progression. This experiment shows that using HSP90 inhibitors can speed up the breakdown of inflammasomes, thus lowering inflammatory responses and easing neurodegeneration. This finding opens up novel possibilities for the selection of immunotherapeutic approaches[105].

While dopaminergic neuron loss in the substantia nigra pars compacta (SNpc) is a defining pathological hallmark of PD, the molecular basis of their selective vulnerability remains unclear. Integrating single-cell genomics and slide-seq spatial transcriptomics, Tushar et al. identified a distinct AGTR1-expressing neuronal subtype spatially confined to the SNpc ventral tier that exhibits heightened PD susceptibility. This vulnerable subtype demonstrated significant upregulation of TP53 and NR2F2 target genes, implicating these transcription factors in degenerative processes, and displayed pronounced transcriptomic alterations in stress pathways regulated by TP53/NR2F2, directly linked to PD-associated neuronal death[106]. Combining single-cell transcriptomic and proteomics, Biqing et al. revealed an inverse correlation between α-synuclein pathology and chaperone expression in excitatory neurons, concurrent with attenuated neuron-astrocyte interactions and exacerbated neuroinflammation in PD. ScRNA-seq analysis demonstrated SYN2 enrichment in PD brains, indicating significantly enhanced synaptic signaling at both transcriptional and proteomic levels, providing novel insights into coordinated molecular dysregulation[107].

3.2.3 Multi-Omics Integration in PD

In the diagnostic process of PD, multi-omics technologies have achieved remarkable success by precisely identifying a series of key biomarkers and pathways (Supplementary Table S2). At the protein and transcript level, GPNMB, CD38, and DGKQ have been identified as candidate

diagnostic biomarkers through integrative proteomic and transcriptomic analysis. These proteins show significant associations with PD risk, and their expression is supported by quantitative trait locus analysis and fine mapping[108]. Furthermore, DDC is consistently upregulated in CSF, blood, and urine of PD patients and is strongly correlated with symptom severity[109]. DDC and related proteins are thus considered viable targets for accurate PD diagnosis.

From a therapeutic perspective, the integration of transcriptomics and metabolomics has revealed that Buyang Huanwu Decoction (BHD) exerts therapeutic effects in PD by modulating key metabolic pathways, including the relaxin signaling pathway, adhesion patch, and PI3K-Akt signaling pathway, and by reducing disease-related symptoms. In preclinical models, BHD treatment enhances dopaminergic neuron survival and improves motor function, indicating its potential for neuroprotection and functional recovery[110]. In addition, genomic and metabolomic studies have identified circular RNA CircSV2b as a potential therapeutic target in PD. CircSV2b is significantly deregulated in PD mouse models compared to wild-type controls, and its overexpression via the ceRNA-Akt1 axis has been shown to mitigate oxidative stress, a central pathological mechanism in PD[111].

Taken together, these integrative approaches not only enhance our understanding of PD heterogeneity but also pave the way for the development of precision diagnostics and personalized therapies. A schematic overview of the multi-omics-driven pathogenesis and therapeutic mechanisms in PD is presented in Figure 5.

3.3 Application of Multi-Omics Technologies in Epilepsy

Epilepsy is the second most common neurological disorder, affecting approximately 3–6% of pediatric neurological cases and occurring across the entire lifespan[112]. It is associated with

recurrent abnormal neuronal discharges in the central nervous system (CNS), leading to episodic dysfunction in motor, sensory, autonomic, and cognitive domains, and contributing to cognitive, psychological, and social impairments[113,114]. The etiology and pathogenesis of epilepsy are highly heterogeneous, involving genetic, metabolic, and inflammatory factors. Over the past decade, omics technologies-including metabolomics, proteomics, and transcriptomics-have provided critical insights into the molecular underpinnings of epilepsy, although the precise mechanisms remain incompletely understood.

3.3.1 Basic-Omics Approaches in Epilepsy

Basic-omics technologies have played a crucial role in identifying key molecular alterations in epilepsy across different biological layers (Supplementary Table S3). Transcriptomic studies have identified dysregulated gene expression in various epilepsy subtypes. In generalized epilepsy, p38MAPK, Jak-STAT, PI3K, and mTOR signaling pathways are consistently upregulated and show stable regulation in affected patients[115]. In temporal lobe epilepsy (TLE), hub genes such as Tlr2, Lgals3, Serpine1, and Stat3 are positively correlated with seizure frequency and are associated with microglial/macrophage activation, ECM remodeling, cell motility, and immune responses[116]. These findings suggest that transcriptomics can provide mechanistic insights into seizure onset and progression.

Proteomic investigations have revealed abnormal protein expression patterns in both diagnostic and therapeutic contexts. In TLE, glial fibrillary acidic protein (GFAP) is consistently downregulated in brain tissue with high spike frequency and shows a strong negative correlation with seizure severity, indicating its role in reactive astrocyte function and neuroprotection[117]. In the hippocampal region of epileptic brains, 144 differentially expressed proteins, such as ADP-

ribosyl cyclase (ADPRC), lysophosphatidic acid receptor 3 (LPAR3), calreticulin, ubiquitin carboxyl-terminal hydrolase L1 (UCH-L1), synaptosome-associated protein 25 (SNAP-25), and transgelin-3, have been identified, with most related to Ca^{2+} homeostasis. Notably, inhibition of calcium influx has been shown to alleviate seizures, supporting the relevance of ion channel and signaling protein targets[118]. Additionally, tutin induces epilepsy and causes significant neurological damage by activating calcineurin, a key phosphatase involved in seizure initiation and progression[119].

Metabolomic analyses have uncovered distinct metabolic signatures in epilepsy. In pediatric epilepsy, N-acetyl glycoprotein, lactate, creatine, glycine, and lipids are elevated, while citrate levels are reduced, suggesting their potential as diagnostic biomarkers[120]. In mesial temporal lobe epilepsy (MTLE), γ-aminobutyric acid (GABA) is significantly upregulated in the epileptogenic zone of KA-MTLE mice and is considered a specific metabolic marker for MTLE[121]. These findings indicate that metabolomics can reveal early-stage metabolic perturbations and pathophysiological changes in epilepsy.

3.3.2 High-spatial-resolution Omics Technologies in Epilepsy

High-spatial-resolution omics technologies have enabled the identification of dysfunctional neuronal subtypes associated with epileptic seizure activity, particularly in the human temporal cortex. SnRNA-seq of over 110,000 neurons revealed the most significant transcriptomic alterations in principal neuron subtypes, including L5-6_Fezf2 and L2-3_Cux2, as well as GABAergic interneurons marked by Sst and Pvalb gene expression. Among the most profoundly dysregulated pathways is glutamatergic signaling, particularly through the upregulation of genes encoding AMPA receptor auxiliary subunits in Sst and Pvalb subtypes. These results highlight the central role of

GABAergic interneurons in early epileptogenesis and their potential as diagnostic targets[122]. Comparative scRNA-seq studies between post-traumatic epilepsy (PTE) and hereditary epilepsy revealed distinct cellular landscapes. Hereditary epilepsy samples exhibited increased abundance of oligodendrocytes and astrocytes, but reduced microglia and neuronal populations compared to PTE. Within microglia and astrocytes, the IL-17 signaling pathway emerged as a potential therapeutic and biomarker candidate, pointing to immune and inflammatory mechanisms as critical contributors to neuronal dysfunction in PTE. Notably, XIST, a long non-coding RNA associated with inflammatory cell infiltration, fibrosis, and satellite glial cell activation, was significantly upregulated in PTE, suggesting its role in epileptogenic progression and early detection[123].

To further uncover the molecular basis of hippocampal sclerosis and hyperexcitability in TLE, a multi-omics strategy combined scRNA-seq, snRNA-seq, and Xenium spatial transcriptomics. The spatial data highlighted glia-specific gene upregulation and neuron-specific downregulation, with key dysregulated genes including Spp1 and Trem2 (upregulated in glia) and Tle4 and Sipa1l3 (downregulated in neurons), suggesting their diagnostic and mechanistic relevance in TLE[124].

3.3.3 Multi-Omics Integration in Epilepsy

In epilepsy diagnosis, multi-omics research has identified several key targets for precise interventions to deepen our understanding of the origins of epilepsy (Supplementary Table S3).

Integrated genomics and transcriptomics have revealed the key role of Sestrin 3 in the pro-convulsant gene network of the human hippocampus in epilepsy. Sestrin 3 positively regulates modules in macrophages, microglia, and neurons, and is considered a potential diagnostic marker[125]. Integrative proteomic and transcriptomic analyses have revealed the involvement of the transforming growth factor β (TGF-β) signaling pathway in cardiac dysfunction associated with

epilepsy. Within this pathway, STAT3, ErbB, and Mapk8 emerge as key regulators of cardiac alterations induced by seizure activity[126]. Integration of proteomics and metabolomics has identified glutathione S-transferase M1 (GSTM1) and aldehyde dehydrogenase 2 (ALDH2) as protein hubs in the somatosensory cortex and thalamus, respectively[127]. Furthermore, genomic and metabolomic integration has identified lactate, creatine, phosphocreatine, and choline as metabolic markers with distinct expression patterns in epilepsy. Lactate is significantly reduced, whereas creatine, phosphocreatine, and choline are markedly elevated, reflecting metabolic reprogramming associated with seizure activity[128].

In epilepsy, although the focus of multi-omics research has largely been on pathogenesis and diagnosis, relatively few studies have explored the therapeutic potential of these approaches. Integrative proteomic and transcriptomic analyses have uncovered miR-10a-5p, miR-21a-5p, and miR-142a-5p as important microRNA transcripts associated with epileptogenesis, primarily through the TGF-β signaling pathway. Notably, anti-miR therapies targeting these miRNAs have shown protective effects in both acute and spontaneous seizure models, underscoring their potential for therapeutic application in modulating seizure activity and signaling networks[129].

In summary, basic-omics and multi-omics approaches have significantly contributed to elucidating the molecular complexity of epilepsy. These technologies have identified critical genes, proteins, and metabolites that are linked to seizure activity, neuroinflammation, and metabolic dysregulation. Although most research has focused on diagnostic and mechanistic exploration, the therapeutic potential of multi-omics-based strategies is increasingly being recognized.

3.4 Application of Multi-Omics Technologies in MS

MS is a chronic, immune-mediated, inflammatory demyelinating disease of the CNS, marked

by temporal and spatial lesion dissemination[130,131]. While the exact etiology remains unclear, risk factors such as Epstein-Barr virus infection, low vitamin D levels, limited sunlight exposure, smoking, and high adolescent BMI have been linked to MS development[132,133]. Clinical symptoms are diverse, including visual impairment, paresthesia, muscle weakness, and progressive disability[134]. A major feature is the presence of oligoclonal bands (OCBs) in CSF, indicating CNS inflammation. Although OCBs are a classical biomarker for MS, their lack of specificity-as they appear in other CNS inflammatory diseases-limits their diagnostic utility[135]. The complex and heterogeneous mechanisms of MS remain incompletely understood, impeding targeted therapeutic development. Multi-omics technologies offer a comprehensive approach to uncover molecular underpinnings and identify novel biomarkers and therapeutic targets.

3.4.1 Basic-Omics Approaches in MS

Basic-omics studies have made substantial contributions to the diagnostic and therapeutic landscape of MS. In proteomic profiling, several proteins have been identified as potential biomarkers, including CXCL13, LTA, FCN2, ICAM3, LY9, SLAMF7, TYMP, CHI3L1, FYB1, TNFRSF1B, and NFL. Notably, lower levels of NFL in CSF show predictive potential for disease activity (AUC = 0.77)[136]. In the metabolomic domain, dopamine receptor D2 (DRD2) has been shown to exacerbate MS by promoting inflammation and reducing Lactobacillus abundance in the gut microbiome. Conversely, Lactobacillus-derived N2-acetyl-L-lysine has anti-neurodegenerative effects by inhibiting microglial activation[137]. In relapsing-remitting multiple sclerosis (RRMS), metabolomics has identified four dysregulated metabolic pathways, with glycolysis serving as a common upstream driver. Targeting glycolysis in experimental autoimmune encephalomyelitis ameliorated the disease pathology by impeding immune cell effector function[138].

Multi-omics technologies integration

3.4.2 High-spatial-resolution Omics Technologies in MS

High-spatial-resolution and single-cell omics technologies have provided unprecedented insights into cellular and molecular heterogeneity in MS, particularly in active lesions and periplaque regions involving both CNS and peripheral immune compartments. Single-cell transcriptomic analysis of CSF has revealed a compartmentalized immune landscape, with enrichment of myeloid dendritic cells and regulatory T cells. A notable finding is the cluster-independent expansion of T follicular helper (TFH) cells, which is associated with increased B-lineage cell infiltration into the CNS and worsened disease severity in MS animal models. These results underscore the local T/B cell crosstalk as a critical driver of MS pathology[139].

Spatial transcriptomics profiling techniques, such as in situ sequencing (10x Xenium) and scRNA-seq, have enabled the spatial mapping of active MS lesion evolution. Astrocytes were categorized into three distinct functional states: homeostatic, intermediate, and disease-associated. Disease-associated astrocytes showed marked upregulation of SERPINA3, a gene strongly correlated with active lesion areas in MS. This expression pattern may reflect a glioprotective response aimed at resolving inflammation and preventing apoptosis during early and late lesion resolution phases, and SERPINA3 has been proposed as a spatial biomarker for MS pathology[140].

Integration of scRNA-seq and spatial transcriptomics has identified perturbations in KLF/SP regulatory drivers in oligodendrocytes, including enhanced iron uptake, expression of pro-inflammatory molecules near axonal injury sites, and the role of MAFB, an inflammatory transcription factor, as a hallmark of MS lesions[141]. These studies highlight the complex interplay between complement factors, apolipoproteins, and immune cells, as well as a distinct APOE-TREM2 axis involved in lesion repair.

Multi-omics technologies integration

Multi-omics integration—combining spatial transcriptomics (10x Visium), scRNA-seq, and spatial proteomics (imaging mass cytometry, IMC)—has uncovered the fibrogenic niche in systemic MS skin lesions, driven by a dynamic fibroblast-macrophage axis through the ACKR3-CXCL12-CXCR4 signaling pathway. Pharmacological inhibition of CXCR4 using AMD3100 significantly reduced dermal and pulmonary fibrosis, as well as myofibroblast accumulation, in preclinical models, validating this pathway as a potential therapeutic target. Importantly, the markedly elevated POSTN/SCARA5 ratio in MS lesions exhibits strong potential as a predictive diagnostic biomarker for disease progression[142].

3.4.3 Multi-Omics Integration in MS

Multi-omics integration has enhanced the comprehensive understanding of MS pathophysiology and expanded the identification of robust biomarkers (Supplementary Table S4). Combined proteomics and transcriptomics have revealed GPR37L1, SIRPA, FGFR3, CADM3, and TYRO3 as highly expressed candidate molecules in the CNS, associated with early neuronal degeneration and impaired trophic and anti-inflammatory intercellular communication, supporting their use as diagnostic tools[143]. Additionally, 24 iron death-related genes (e.g., CHMP5, SLC38A1, PML) have been linked to neuroinflammatory processes, where high iron death scores correlate with phagocytic activation at lesion margins and neurological dysfunction in cortical neurons. A blood-based model incorporating these genes has shown prognostic value for MS diagnosis[144]. An integrative approach combining proteomics and metabolomics has identified LAMP1, FCG2A, and heparinase (HPSE) as potential specific biomarkers for MS, with HPSE showing strong correlations with metabolites such as L-tyrosine, sphingosine 1-phosphate, and L-tryptophan[145]. Moreover, another study has also identified reduced levels of anti-inflammatory

molecules and sphingolipids, as well as low equine uric acid in severe MS subgroups, pointing to their potential in biomarker development and targeted therapeutic strategies[146].

Currently, our understanding of the relationship between multi-omics profile alterations in MS patients and the underlying molecular networks contributing to MS pathogenesis remains limited. This study outlines a potential integrated multi-omics mechanism that may regulate peripheral immune-inflammatory responses and the progression of MS. These findings can facilitate the development of novel auxiliary diagnostic biomarkers and therapeutic strategies for MS. The summary information is shown in Supplementary Table S4.

3.5 Application of Multi-Omics Technologies in Stroke

Stroke, or cerebrovascular accident, is the most severe neurological disorder to date, causing approximately 160 million years of healthy life lost annually. It is mainly classified into ischemic stroke (IS, ~87%) and hemorrhagic stroke (HS), resulting from vascular injury and leading to focal or global brain damage. Common symptoms include hemiplegia, facial paralysis, and speech impairment, with severe cases progressing to sudden loss of consciousness. Stroke is characterized by high incidence, disability, and mortality, underscoring the urgent need for improved prevention and treatment strategies. High-throughput technologies now offer new opportunities to unravel its complex pathophysiological mechanisms[147,148].

3.5.1 Basic-Omics Approaches in Stroke

Basic-omics studies have identified key biomarkers and pathways associated with stroke (Supplementary Table S5). Proteomic profiling has revealed elevated levels of NSF, RhoGDI1, RabGDI, CKB, and CMPK in the circulation of IS patients, reflecting neuronal excitotoxicity and energy metabolism disruption[149,150]. Additionally, SAHH2 increased expression of SAHH2 in

neurons from the infarcted area, probably because of ischemia-triggered Ca2+ mobilization[151].

Transcriptomic analyses have identified differentially expressed long non-coding RNAs (lncRNAs), such as MEG3, H19, and MALAT1, and extracellular microRNAs including miR-32-3p, miR-106b-5p, miR-423-5p, and miR-4739. These molecules are involved in apoptosis, oxidative stress, angiogenesis, and neurogenesis[152-157].

Metabolomic studies indicate that reduced levels of BCAA are associated with cardioembolic stroke and poor neurological outcomes, highlighting their potential as diagnostic and prognostic biomarkers[158]. Additionally, total plasma free fatty acid levels are significantly elevated in cardioembolic stroke patients compared to those with non-cardioembolic stroke, further suggesting their value as diagnostic targets[159].

3.5.2 High-spatial-resolution Omics Technologies in Stroke

High-spatial-resolution omics technologies have revealed critical molecular and cellular mechanisms underlying stroke pathophysiology and recovery, particularly in aging-related neuroinflammation and myelin repair. scRNA-seq studies have shown that aging impairs paracrine communication between microglia/macrophages and endothelial and oligodendrocyte precursor cells, thereby hindering angiogenesis and remyelination. Transplantation of young microglia/macrophages into aged stroke models partially restores these processes and sensorimotor function, highlighting these cells as potential therapeutic targets for stroke recovery[160].

Modulating LILRB4 signaling and its downstream effectors emerges as a promising therapeutic approach for IS, as spatial transcriptomics and scRNA-seq have identified a stroke-associated microglial cluster 3 and revealed significant upregulation of LILRB4 expression in ischemic brain regions. Functional studies show that LILRB4 deficiency exacerbates ischemic

injury through increased CD8+ T cell recruitment, while its overexpression exerts neuroprotective effects, underscoring the therapeutic potential of modulating LILRB4 and its downstream pathways in mitigating immune-mediated damage[161]. In contrast, Li et al. investigated brainstem stroke and found that oligodendrocyte loss leads to neurological deficits, followed by regenerative attempts. They identified a sub-cluster of Pros1+ oligodendrocytes, termed OLG8, and demonstrated that Myo1e overexpression promotes OLG8 differentiation, reduces ischemic damage, and enhances neurological recovery, offering cell-type-specific therapeutic avenues[162]. Gu et al. further uncovered the dynamic immune landscape in post-stroke brains, revealing distinct differentiation patterns between myeloid and lymphoid cell populations and spatially localized interactions. Notably, a Spp1-high lymphocyte sub-cluster was found to interact with Lyz2+ macrophage-associated lymphocytes, while in the choroid plexus, Lgmn+ macrophage-T cell communication via the Spp1-CD44 axis was observed during the acute phase of intracerebral hemorrhage. These findings establish Spp1 and Lyz2 as potential biomarkers for stroke diagnostics[163]. Further, they identified ferroptosis as the dominant form of programmed cell death occurring as early as one hour post-intracerebral hemorrhage, primarily in mature oligodendrocytes. They showed that a CSF1/CSF1R-mediated interaction between Lipocalin-2+ microglia and oligodendrocytes drives ferroptosis and functional deterioration, suggesting that early inhibition of microglial Lipocalin-2 may protect oligodendrocytes and mitigate post-stroke deficits, representing a novel neuroprotective strategy[164]. In IS models, Han et al. revealed elevated galectin (LGAL) signaling in microglia and macrophages, and found that LGALS9 administration promotes oligodendrocyte remyelination and functional recovery, supporting its therapeutic utility in stroke[165]. In subarachnoid hemorrhage (SAH), Wang et al. demonstrated that mLV injury is induced by SAH, with THBS1 and

S100A6 showing marked upregulation post-injury. They identified the THBS1-CD47 ligand-receptor axis as a key driver of meningeal lymphatic endothelial apoptosis through STAT3/Bcl-2 signaling, suggesting that targeting this axis could preserve mLV integrity and improve clinical outcomes[166].

3.5.3 Multi-Omics Integration in Stroke

Multi-omics integration has enabled the identification of complex molecular interactions and novel therapeutic targets in stroke (Supplementary Table S5). In CES, combined proteomic and transcriptomic approaches have uncovered ICA1L, CAND2, and ALDH2 as potential biomarkers related to excitatory synaptic dysfunction[167]. In the therapeutic context, integrated genomics and metabolomics have shown that Zhilong Huoxue Tongyu Capsules (ZHTC), a traditional Chinese medicin, can modulate gut microbiota and metabolic profiles, including arginine, lysine, and methionine, and enhance intestinal barrier integrity[168]. Furthermore, multi-omics and network pharmacology studies on Yiqitongluo Granule (YQTL) have revealed 15 active components that regulate 82 targets across 19 pathways, with PI3K-Akt, MAPK, and cAMP signaling playing central roles in neuroprotection against cerebral ischemia-reperfusion injury[169].

In summary, basic-omics studies have provided critical insights into the biomarkers and pathophysiological mechanisms of stroke, particularly in IS and cardioembolic stroke. Proteomic, transcriptomic, and metabolomic approaches have identified key proteins, non-coding RNAs, and metabolic alterations associated with disease onset, progression, and outcomes. Meanwhile, multi-omics integration has further revealed molecular networks and therapeutic targets, demonstrating the potential of systems biology in advancing stroke diagnosis and treatment.

3.6 Application of Multi-Omics Technologies in Hydrocephalus

Hydrocephalus is a potentially fatal neurologic disorder affecting individuals across the lifespan[170]. It is characterized by the abnormal accumulation of CSF due to disruption of its circulation, resulting in ventricular dilation and frequently elevated intracranial pressure (ICP)[171]. Based on CSF flow dynamics, the disease is classified into three main types: obstructive, communicating, and normal pressure hydrocephalus (NPH)[172]. Despite modest progress in surgical techniques over the past five decades, preventive and curative strategies remain limited. Current diagnostic methods-Hakim's triad, computed tomography(CT) and magnetic resonance imaging(MRI), lumbar puncture, and lumbar drainage-are subjective, invasive, and lack specificity, especially in differentiating NPH from vascular or AD-related dementias, due to incomplete understanding of its pathogenesis[170,173]. Likewise, pharmacological therapies have not produced effective treatment options so far[174-176].

In this context, multi-omics technologies are emerging as promising tools for elucidating the molecular mechanisms, identifying novel biomarkers, and developing targeted, non-invasive therapeutic strategies for hydrocephalus.

3.6.1 Basic-Omics Approaches in Hydrocephalus

Basic-omics studies have identified key molecular signatures associated with the pathogenesis and clinical manifestations of hydrocephalus, particularly communicating hydrocephalus (CH) and idiopathic normal pressure hydrocephalus (iNPH) (Supplementary Table S6). Genomics in CH has revealed TRIM71 and SMARCC1 as genes with genome-wide significant de novo mutations, potentially serving as genetic risk factors. Additionally, PIK3CA, PTEN, MTOR, FOXJ1, FMN2, PTCH1, and FXYD2 have been identified as high-confidence sporadic CH-associated genes, with

TRIM71 deletion linked to reduced neural cell proliferation, making it a potential diagnostic marker[177].

Proteomic profiling has identified kallikrein-6 (KLK6) as significantly upregulated in CH patients, implicating it in disease progression and suggesting its utility in diagnostic strategies[178]. In iNPH, 39 upregulated and 285 downregulated proteins in CSF have been observed, with elevated glutaminyl-peptide cyclotransferase (QPCT) and retinol-binding protein 4 (RBP4) levels showing prognostic and diagnostic relevance for shunt response[179]. Notably, Q-type protein tyrosine phosphatase receptor (PTPRQ) is significantly higher in iNPH compared to AD and may help distinguish iNPH from AD-related dementia[180].

Metabolomic studies in iNPH and NPH have uncovered CSF metabolic profiles that aid in differential diagnosis and treatment response prediction. In iNPH, elevated glyceric acid and N-acetylneuraminic acid (Neu5Ac), along with reduced serine and 2-hydroxybutyric acid, form a diagnostic signature that differentiates it from AD[181]. In NPH, low CSF Neu5Ac levels are associated with astrocyte activation and periventricular demyelination. Elevating brain Neu5Ac has been shown to improve neurological outcomes, indicating its therapeutic potential[182].

3.6.2 High-spatial-resolution Omics Technologies in Hydrocephalus

A recent study utilizing snRNA-seq and spatial transcriptomics in a tumor-associated hydrocephalus (TAH) mouse model revealed the expansion of choroid plexus mast cells (CPMCs) in the ventricular region, including the choroid plexus and ependymal walls. These CPMCs contribute to TAH pathogenesis by disrupting ciliated epithelial cells through the tryptase-PAR2-FoxJ1 signaling axis, thereby enhancing CSF production. Importantly, elevated CSF tryptase levels have been associated with increased clinical severity of TAH, and administration of the brain-

penetrant tryptase inhibitor BMS-262084 significantly attenuated TAH in vivo and protected against ciliary damage in human stem cell-derived choroid plexus organoids. These results identify CPMCs as key drivers of TAH and highlight BMS-262084 as a potential therapeutic candidate[183].

3.6.3 Multi-Omics Integration in Hydrocephalus

Multi-omics integration has enabled a systems-level understanding of hydrocephalus and the identification of novel diagnostic and therapeutic biomarkers through cross-omics validation and functional annotation (Supplementary Table S6). In response, multi-omics joint analysis has emerged as a crucial method for revealing therapeutic targets and refining diagnostic methods by integrating and comprehensively analyzing data from different omics layers (Supplementary Table S6). Integrated genomics, proteomics, and transcriptomics in CH have highlighted the maelstrom spermatogenic transposon silencer (MAEL) as a candidate diagnostic biomarker. Reduced MAEL expression in multiple brain regions is significantly associated with hydrocephalus, and PrediXcan analysis confirms its pathophysiological relevance[184]. In post-hemorrhagic hydrocephalus, the combined use of proteomics and metabolomics has identified chondroitin sulfate proteoglycan 4 (CSPG4) as a promising CSF biomarker. CSPG4 is positively correlated with ventricular size and the incidence of periventricular leukomalacia. Functional studies indicated that CSPG4 silencing can inhibit ferroptosis, cell adhesion, and intracellular Ca^{2+} flux, supporting its role in both diagnosis and treatment[185].

Although multi-omics approaches have unveiled a plethora of candidate targets and pathways for hydrocephalus therapy, definitive clinical breakthroughs in therapeutic development remain elusive. These findings not only enhance our comprehension of the pathological processes of hydrocephalus but also lay a theoretical foundation for the future development of targeted therapies

directed at specific pathways or molecules.

4. Current Challenges and Future Trends

Although multi-omics technologies have advanced our understanding of neurological disease pathophysiology, their full clinical translation is hindered by significant technical, analytical, and translational challenges. These are particularly evident in high-spatial-resolution omics approaches, such as scRNA-seq and spatial transcriptomics, which provide cell-type-specific and spatially resolved insights but also introduce additional layers of complexity in data generation, integration, and interpretation.

4.1 Data Complexity in Multi-omics and High-Spatial-Resolution Omics Integration

Omics data is inherently high-dimensional, heterogeneous, and multi-modal, posing significant challenges in standardization, integration, and biological interpretation. The lack of universally accepted data formats and protocols across institutions leads to inconsistent data quality and poor interoperability, especially when integrating genomics, transcriptomics, and proteomics, which vary in dynamic ranges and measurement scales[186]. Technical variability in sample preparation and instrument performance further introduces noise, missing values, and batch effects, necessitating robust preprocessing strategies, such as missing value imputation and outlier detection, to ensure data reliability[187-189].

High-spatial-resolution omics technologies, such as scRNA-seq and spatial transcriptomics, exacerbate these challenges by capturing cell-type-specific and spatially resolved molecular profiles. These approaches generate ultra-high-spatial-resolution datasets with substantial cellular and spatial heterogeneity, requiring advanced computational pipelines for clustering, trajectory inference, and

cell-type annotation. Spatial transcriptomics, in particular, introduces spatial coordinates, thereby demanding novel algorithms that can integrate molecular and spatial information simultaneously[190]. The absence of standardized annotation systems and centralized data repositories for high-spatial-resolution data remains a major bottleneck, especially in complex brain regions such as the cortex, hippocampus, and choroid plexus, where cellular diversity and spatial architecture are particularly pronounced[191].

Given these challenges, the emergence of artificial intelligence (AI), particularly ML and deep learning, has opened new avenues for multi-omics data integration. ML algorithms can extract key features and identifying underlying patterns from high-dimensional and heterogeneous datasets, offering critical support for biomarker discovery and mechanistic elucidation[192]. For instance, DL-based approaches have demonstrated remarkable success in early AD diagnosis by analyzing integrated multi-omics and network data, significantly improving diagnostic accuracy[193]. Moreover, AI-driven multi-omics analysis not only enhances the understanding of neurodegenerative disease mechanisms but also accelerates the identification of novel druggable targets and supports the development of disease-specific biomarkers, ultimately improving treatment outcomes[194].

4.2 Challenges in Biomarker Validation and Clinical Translation

The validation of neurological disease biomarkers remains a critical translational hurdle, particularly in the context of multi-omics and high-spatial-resolution omics. Traditional bulk omics methods often average out cellular and inter-individual heterogeneity, thereby obscuring biologically relevant signals that are cell-type- or region-specific. In contrast, scRNA-seq and spatial transcriptomics provide detailed molecular profiles at the cellular and spatial level, yet their

technical complexity and data variability pose significant challenges for robust clinical validation. A major limitation is the lack of standardized, reproducible, and scalable validation frameworks that can retain the resolution and biological context of high-dimensional and multi-modal data. Most existing protocols are optimized for bulk-level analysis, which fails to preserve the cellular and spatial information essential for identifying cell-type- or region-specific biomarkers. As a result, many high-spatial-resolution biomarkers may not be detectable or reproducible in standard clinical assays, where resolution is lower and biological noise is higher. For instance, S100B, a protein detectable in stroke patients, lacks sufficient specificity due to its expression in healthy individuals and in other neurological conditions[195]. This underscores the need for more rigorous validation strategies that account for inter-individual variability driven by genetic, epigenetic, and environmental factors. Such variability can mask or distort molecular signatures, requiring large-scale, well-phenotyped, and multi-omics-annotated cohorts for robust biomarker discovery and cross-population validation. Furthermore, the clinical translation of multi-omics findings is hindered by limited clinician engagement and inadequate integration with clinical workflows.

To overcome these barriers, a comprehensive and standardized validation framework is essential. This framework should incorporate high-spatial-resolution experimental validation, multi-omics data harmonization, and clinically annotated reference datasets to ensure reproducibility and generalizability across diverse populations and clinical settings. Moreover, collaboration across disciplines—including computational biology, neurology, and bioinformatics—is necessary to align research findings with clinical needs and to develop practical, scalable solutions for the translation of high-spatial-resolution omics biomarkers into routine clinical diagnostics and personalized treatment strategies.

4.3 Multi-modal Integration of Multi-omics with Clinical Imaging and Real-Time Sensing

A key future direction in neurological disease research lies in the integration of multi-omics and high-spatial-resolution omics data with clinical imaging and real-time sensing technologies. This multi-modal strategy enables the bridging of molecular insights with anatomical and functional information, thereby enhancing mechanistic understanding at multiple biological scales. For example, in the research of AD, by utilizing MRI to obtain brain structural images and integrating metabolomics data, it becomes possible to identify abnormal metabolite changes in specific brain regions[196]. Such integrative frameworks are critical for connecting molecular heterogeneity with clinical phenotypes, improving diagnostic accuracy, and enabling personalized therapeutic interventions.

Despite its promise, this approach faces several technical and methodological challenges. First, the heterogeneous nature of omics, imaging, and real-time sensor data complicates data alignment and integration. Second, standardized protocols for multi-modal validation are still in early development, which limits reproducibility and clinical translation. Third, real-time data from wearable biosensors, while offering continuous monitoring and high temporal resolution, pose new computational demands when fused with static omics data, requiring novel methods for dynamic profiling and predictive modeling.

Moving forward, the development of unified platforms that support seamless integration of multi-omics, imaging, and real-time sensing will be essential for advancing precision neurology. These platforms should incorporate AI-driven analysis, adaptive data fusion strategies, and clinically validated biomarker pipelines to facilitate early detection, individualized treatment

planning, and longitudinal disease tracking.

5. Conclusion

Neurological diseases, characterized by their high incidence, high disability rate, and severe impact on patients' quality of life, have emerged as a significant global health challenge that urgently demands solutions. While traditional methods have laid the groundwork for understanding disease mechanisms, the integration of multi-omics and high-spatial-resolution omics technologies now provides comprehensive and spatially resolved molecular insights. Studies on major neurological disorders, including AD, PD, MS, stroke, and hydrocephalus, have identified numerous disease-associated genes, proteins, metabolites, and pathways. These discoveries deepen our understanding of pathogenesis, highlight key drivers of disease progression, and offer potential biomarkers and therapeutic targets for early detection, precision treatment, and prognosis.

Despite these advances, the application of multi- and high-spatial-resolution omics in this field remains challenging, particularly in data integration, standardization of validation protocols, and translation to clinical settings. Addressing these issues is critical for harnessing the full potential of these technologies in neurological disease research. Looking ahead, the continued development of open-source platforms will enhance the adoption and utility of multi- and high-spatial-resolution omics approaches. This progress is expected to accelerate mechanistic discovery, improve diagnostic accuracy, and support individualized therapies, ultimately advancing precision neurology and translational medicine.

Additional Files

Supplementary Table S1. Application of Multi-omics and High-spatial-resolution Omics Technologies in AD.

Multi-omics technologies integration

Supplementary Table S2. Application of Multi-omics and High-spatial-resolution Omics Technologies in PD.

Supplementary Table S3. Application of Multi-omics and High-spatial-resolution Omics Technologies in Epilepsy.

Supplementary Table S4. Application of Multi-omics and High-spatial-resolution Omics Technologies in MS.

Supplementary Table S5. Application of Multi-omics and High-spatial-resolution Omics Technologies in Stroke.

Supplementary Table S6. Application of Multi-omics and High-spatial-resolution Omics Technologies in Hydrocephalus.

Abbreviations

AI: artificial intelligence; AD: Alzheimer's disease; ADPRC: ADP-ribosyl cyclase; ALDH2: aldehyde dehydrogenase 2; AlzGPS: Alzheimer’s disease genome-wide positioning systems platform; BBB: blood-brain-barrier; BCAA: branched-chain amino acid; BHD: Buyang huanwu decoction; CH: communicating hydrocephalus; CNS: central nervous system; CPMCs: choroid plexus mast cells; CSF: cerebrospinal fluid; CSPG4: chondroitin sulfate proteoglycan 4; CT: computed tomography; DDA: data-dependent acquisition; DIA/SWATH: data independent acquisition-sequential window acquisition of all theoretical mass spectral approach; DRD2: domain, dopamine receptor D2; FT-IR: fourier-transform infrared; GFAP: glial fibrillary acidic protein; GC-MS: gas chromatography–mass spectrometry; GABA: γ-aminobutyric acid; GWAS: genome-wide association studies; GSTM1: glutathione S-transferase M1; HS: hemorrhagic stroke; HPSE: heparinase; H4K16ac: acetylation status of histone H4 at lysine 16; iNPH: idiopathic normal

pressure hydrocephalus; ICP: intracranial pressure; IL-1β: interleukin-1β; IL-6: interleukin-6; IS: ischemic stroke; iTRAQ: isobaric tags for relative and absolute quantification; KLK6: kallikrein-6; LC-MS: liquid chromatography–mass spectrometry; lncRNAs: long non-coding RNAs; LPAR3: lysophosphatidic acid receptor 3; LRRK2: leucine-rich repeat kinase 2; MAEL: maelstrom spermatogenic transposon silencer; MBP: Myeloid basic protein encoding gene; ML: machine learning; MS: multiple sclerosis; MTLE: mesial temporal lobe epilepsy; MRI: magnetic resonance imaging; Neu5Ac: N-acetylneuraminic acid; NMR: nuclear magnetic resonance; NPH: normal pressure hydrocephalus; OCBs: oligoclonal bands; PIGs: plaque-induced genes; PD: Parkinson's disease; PKM2: pyruvate kinase M2; PTE: post-traumatic epilepsy; PTPRQ: Q-type protein tyrosine phosphatase receptor; QPCT: glutaminyl-peptide cyclotransferase; RBP4: retinol-binding protein 4; RF: random forest; RNA-seq: RNA sequencing; RRMS: relapsing-remitting multiple sclerosis; SAH: subarachnoid hemorrhag; scRNA-seq: single-cell RNA sequencing; SILAC: stable-isotope labeling by amino acids in cell culture; SNPs: single-nucleotide polymorphisms; SNAP-25: synaptosome-associated protein 25; SNpc: substantia nigra pars compacta; snRNA-seq: single-nucleus RNA sequencing; SSR1: signal sequence receptor subunit 1; TAH: tumor-associated hydrocephalus; TGF-β: transforming growth factor β; TLE: temporal lobe epilepsy; TNF-α: tumor necrosis factor-α; TMT: tandem mass tag; TFH: T follicular helper; UCH-L1: calreticulin, ubiquitin carboxyl-terminal hydrolase L1; YQTL: Yiqitongluo granule; ZHTC: Zhilong huoxue ttongyu capsules.

Declarations

Ethics Approval and Consent to Participate (Not Applicable)

Consent for Publication (Not Applicable)

Data Availability

The datasets in the current study are available by reasonable request to the first author. (xiuyun_liu@tju.edu.cn).

Competing Interests

The authors declare no competing financial interest.

Funding

This study was funded by Scientific Research Innovation Capability Support Project for Young Faculty (ZYGXQNJSKYCXNLZCXM-H15), National Science Fund for Excellent Overseas Scholars (0401260011), National Natural Science Foundation of China (82472098, 32300704), Tianjin Natural Science Foundation-Outstanding Youth Project (24JCJQJC00250), Major Science and Technology Special Projects and Engineering-Major Project of National Key Laboratories (24ZXZSSS00510) and National Key Technologies Research and Development Program (2021YFF1200602), the Non-profit Central Research Institute Fund of Chinese Academy of Medical Sciences (2024-JKCS-16). Part of the figures were created by BioRender (www.biorender.com).

Author’s Contributions

Xiuyun Liu: conceptualization; Xiuyun Liu and Fangfang Li: writing-review & editing; Fangfang Li: writing – original draft; Xiuyun Liu, Fangfang Li, Meijun Pang, Marek Czosnyka, and Zofia Czosnyka: methodology; Meijun Pang, Huijie Yu, Xiaoguang Tong, Yan Xing, Hongliang Li,

Multi-omics technologies integration

Ke Pu, Keke Feng, and Kuo Zhang: investigation; Meijun Pang: supervision; Dong Ming: project administration.

Acknowledgements

The authors sincerely acknowledge the financial support for this study from the Scientific Research Innovation Capability Support Project for Young Faculty (ZYGXQNJSKYCXNLZCXM-H15), National Science Fund for Excellent Overseas Scholars (0401260011), National Natural Science Foundation of China (82472098, 32300704), Tianjin Natural Science Foundation-Outstanding Youth Project (24JCJQJC00250), Major Science and Technology Special Projects and Engineering-Major Project of National Key Laboratories (24ZXZSSS00510), National Key Technologies Research and Development Program (2021YFF1200602), and Non-profit Central Research Institute Fund of Chinese Academy of Medical Sciences (2024-JKCS-16). Special thanks are due to BioRender (www.biorender.com) for supporting the creation of part of the study's figures. The authors also appreciate all team members who contributed to study methodology, investigation, supervision, project administration, and manuscript preparation—their efforts were essential to the completion of this research.

Authors' information

Xiuyun Liu, Chair Professor of Tianjin University, Deputy Director of the Medical Department, and Dean of the School of Pharmacy. The research primarily focuses on neurocritical care medicine and critical care brain-computer interface, with a specific emphasis on developing key diagnostic and therapeutic technologies for brain disorders such as reversible dementia (hydrocephalus), dementia, traumatic brain injury, epilepsy, stroke, and postoperative delirium. The overarching goal is to address the current engineering bottlenecks in neurocritical care, providing physicians with

precise treatment methods and strategies. Tianjin University, 92 Weijin Road, Nankai District, Tianjin, 300072, China, Email: xiuyun_liu@tju.edu.cn.

References

1. Steinmetz, J D, Seeher, Katrin Maria et al Global, regional, and national burden of disorders affecting the nervous system, 1990–2021: a systematic analysis for the Global Burden of Disease Study 2021. Lancet Neurol. 2024;23:344-381. https://doi.org/10.1016/s1474-4422(24)00038-3
2. Leinenga, G, Langton, C, Nisbet, R, and Götz, J Ultrasound treatment of neurological diseases — current and emerging applications. Nat Rev Neurol. 2016;12:161-174. https://doi.org/10.1038/nrneurol.2016.13
3. Milligan, T A Diagnosis in neurologic disease. Med Clin North Am. 2019;103:173-190. https://doi.org/10.1016/j.mcna.2018.10.011
4. Jankovic, J Parkinson's disease: clinical features and diagnosis. J Neurol Neurosurg Psychiatry. 2008;79:368-376. https://doi.org/10.1136/jnnp.2007.131045
5. Kuska, B Beer, Bethesda, and biology: how "genomics" came into being. J Natl Cancer Inst. 1998;90:93. https://doi.org/10.1093/jnci/90.2.93
6. Manzoni, C, Kia, D A, Vandrovcova, J, Hardy, J, Wood, N W, Lewis, P A, et al. Genome, transcriptome and proteome: the rise of omics data and their integration in biomedical sciences. Brief Bioinform. 2018;19:286-302. https://doi.org/10.1093/bib/bbw114
7. Karczewski, K J, and Snyder, M P Integrative omics for health and disease. Nat Rev Genet. 2018;19:299-310. https://doi.org/10.1038/nrg.2018.4
8. Hasin, Y, Seldin, M, and Lusis, A Multi-omics approaches to disease. Genome Biol. 2017;18:83. https://doi.org/10.1186/s13059-017-1215-1
9. Bujak, R, Struck-Lewicka, W, Markuszewski, M J, and Kaliszan, R Metabolomics for laboratory diagnostics. J Pharm Biomed Anal. 2015;113:108-120. https://doi.org/10.1016/j.jpba.2014.12.017
10. C., F R. Epigenetics: The ultimate mystery of inheritance. W. W. Norton & Company; 2011.
11. Qian, Z, Qin, J, Lai, Y, Zhang, C, and Zhang, X Large-Scale Integration of Single-Cell RNA-Seq Data Reveals Astrocyte Diversity and Transcriptomic Modules across Six Central Nervous System Disorders. Biomolecules. 2023;13. https://doi.org/10.3390/biom13040692
12. Shapiro, E, Biezuner, T, and Linnarsson, S Single-cell sequencing-based technologies will revolutionize whole-organism science. Nat Rev Genet. 2013;14:618-30. https://doi.org/10.1038/nrg3542
13. Close, J L, Long, B R, and Zeng, H Spatially resolved transcriptomics in neuroscience. Nature methods. 2021;18:23-25. https://doi.org/10.1038/s41592-020-01040-z
14. Uffelmann, E, Huang, Q Q, Munung, N S, de Vries, J, Okada, Y, Martin, A R, et al. Genome-wide association studies. Nat Rev Methods Primers. 2021;1:59. https://doi.org/10.1038/s43586-021-00056-9
15. Madden, E B, Hindorff, L A, Bonham, V L, Akintobi, T H, Burchard, E G, Baker, K E, et al. Advancing genomics to improve health equity. Nat Genet. 2024;56:752-757. https://doi.org/10.1038/s41588-024-01711-z
16. iétu G, M-S R, Fayein NA, Matingou C, Eveno E, Houlgatte R, Decraene C, Vandenbrouck Y, Tahi F, Devignes MD, Wirkner U, Ansorge W, Cox D, Nagase T, Nomura N, Auffray C The Genexpress

IMAGE knowledge base of the human brain transcriptome prototype integrated resource for functional and computational genomics. Genome Res. 1999;9:195 – 209. https://doi.org/10.1101/gr.9.2.195
17. Lowe, R, Shirley, N, Bleackley, M, Dolan, S, and Shafee, T Transcriptomics technologies. Plos Comput Biol. 2017;13. https://doi.org/10.1371/journal.pcbi.1005457
18. Lebrigand, K, Bergenstråhle, J, Thrane, K, Mollbrink, A, Meletis, K, Barbry, P, et al. The spatial landscape of gene expression isoforms in tissue sections. Nucleic Acids Res. 2023;51:e47-e47. https://doi.org/10.1093/nar/gkad169
19. Kukurba, K R, and Montgomery, S B RNA Sequencing and Analysis. Cold Spring Harbor Protocols. 2015;2015:951–969. https://doi.org/10.1101/pdb.top084970
20. Godovac-Zimmermann, J 8th siena meeting. From genome to proteome: Integration and proteome completion. Expert Rev Proteomic. 2014;5:769-773. https://doi.org/10.1586/14789450.5.6.769
21. Aggarwal, K, Choe Lh Fau - Lee, K H, and Lee, K H Shotgun proteomics using the iTRAQ isobaric tags. Brief Funct Genomic Proteomic. 2006;5(2):112-120. https://doi.org/10.1093/bfgp/ell018
22. Sharma, K B, Aggarwal, S, Yadav, A K, Vrati, S, and Kalia, M Studying autophagy using a TMT-based quantitative proteomics approach. Methods Mol Biol. 2022;2445:183 – 203. https://doi.org/10.1007/978-1-0716-2071-7_12
23. Chen, X, Wei, S, Ji, Y, Guo, X, and Yang, F Quantitative proteomics using SILAC: Principles, applications, and developments. Proteomics. 2015;15:3175-3192. https://doi.org/10.1002/pmic.201500108
24. Souza, G, Guest, P C, and Martins-de-Souza, D LC-MS(E), multiplex MS/MS, ion mobility, and label-free quantitation in clinical proteomics. Methods Mol Biol. 2017;1546:57 – 73. https://doi.org/10.1007/978-1-4939-6730-8_4
25. Aebersold, R, Bensimon, A, Collins, B C, Ludwig, C, and Sabido, E Applications and developments in targeted proteomics: from SRM to DIA/SWATH. Proteomics. 2016;16:2065-2067. https://doi.org/10.1002/pmic.201600203
26. Aebersold, R, and Mann, M Mass-spectrometric exploration of proteome structure and function. Nature. 2016;537:347-355. https://doi.org/10.1038/nature19949
27. Nicholson JK, Lindon JC, and E, H Metabonomics understanding the metabolic responses of living systems to pathophysiological stimuli via multivariate statistical analysis of biological NMR spectroscopic data. Xenobiotica. 1999;29:1181–1189. https://doi.org/10.1080/004982599238047
28. Wu, X, Ao, H, Gao, H, and Zhu, Z Metabolite biomarker discovery for human gastric cancer using dried blood spot mass spectrometry metabolomic approach. Sci Rep-uk. 2022;12:14632. https://doi.org/10.1038/s41598-022-19061-3
29. Schrimpe-Rutledge, A C, Codreanu, S G, Sherrod, S D, and McLean, J A Untargeted metabolomics strategies—challenges and emerging directions. J Am Soc Mass Spectr. 2016;27:1897-1905. https://doi.org/10.1007/s13361-016-1469-y
30. Carrillo, A J, Halilovic, L, Hur, M, Kirkwood, J S, and Borkovich, K A Targeted metabolomics using LC-MS in neurospora crassa. Curr Protoc. 2022;2:e454. https://doi.org/10.1002/cpz1.454
31. Chen, W, Gong, L, Guo, Z, Wang, W, Zhang, H, Liu, X, et al. A novel integrated method for large-scale detection, identification, and quantification of widely targeted metabolites: application in the study of rice metabolomics. Mol Plant. 2013;6:1769-1780. https://doi.org/10.1093/mp/sst080
32. Tang, F, Barbacioru, C, Wang, Y, Nordman, E, Lee, C, Xu, N, et al. mRNA-Seq whole-transcriptome analysis of a single cell. Nature methods. 2009;6:377-82.

https://doi.org/10.1038/nmeth.1315
33. Chen, C, Xing, D, Tan, L, Li, H, Zhou, G, Huang, L, et al. Single-cell whole-genome analyses by Linear Amplification via Transposon Insertion (LIANTI). Science. 2017;356:189-194. https://doi.org/10.1126/science.aak9787
34. Luo, C, Fernie, A R, and Yan, J Single-Cell Genomics and Epigenomics: Technologies and Applications in Plants. Trends Plant Sci. 2020;25:1030-1040. https://doi.org/10.1016/j.tplants.2020.04.016
35. Wang, J, Ye, F, Chai, H, Jiang, Y, Wang, T, Ran, X, et al. Advances and applications in single-cell and spatial genomics. Sci China Life Sci. 2025;68:1226-1282. https://doi.org/10.1007/s11427-024-2770-x
36. Irish, J M, Hovland, R, Krutzik, P O, Perez, O D, Bruserud, Ø, Gjertsen, B T, et al. Single cell profiling of potentiated phospho-protein networks in cancer cells. Cell. 2004;118:217-28. https://doi.org/10.1016/j.cell.2004.06.028
37. Aldridge, S, and Teichmann, S A Single cell transcriptomics comes of age. Nat Commun. 2020;11:4307. https://doi.org/10.1038/s41467-020-18158-5
38. Ståhl, P L, Salmén, F, Vickovic, S, Lundmark, A, Navarro, J F, Magnusson, J, et al. Visualization and analysis of gene expression in tissue sections by spatial transcriptomics. Science. 2016;353:78-82. https://doi.org/10.1126/science.aaf2403
39. Eisenstein, M Seven technologies to watch in 2022. Nature. 2022;601:658-661. https://doi.org/10.1038/d41586-022-00163-x
40. Moffitt, J R, Lundberg, E, and Heyn, H The emerging landscape of spatial profiling technologies. Nat Rev Genet. 2022;23:741-759. https://doi.org/10.1038/s41576-022-00515-3
41. Bouwman, B A M, Crosetto, N, and Bienko, M The era of 3D and spatial genomics. Trends Genet. 2022;38:1062-1075. https://doi.org/10.1016/j.tig.2022.05.010
42. Lundberg, E, and Borner, G H H Spatial proteomics: a powerful discovery tool for cell biology. Nat Rev Mol Cell Biol. 2019;20:285-302. https://doi.org/10.1038/s41580-018-0094-y
43. Taylor, M J, Lukowski, J K, and Anderton, C R Spatially Resolved Mass Spectrometry at the Single Cell: Recent Innovations in Proteomics and Metabolomics. J Am Soc Mass Spectrom. 2021;32:872-894. https://doi.org/10.1021/jasms.0c00439
44. Vandereyken, K, Sifrim, A, Thienpont, B, and Voet, T Methods and applications for single-cell and spatial multi-omics. Nat Rev Genet. 2023;24:494-515. https://doi.org/10.1038/s41576-023-00580-2
45. Xia, C, Fan, J, Emanuel, G, Hao, J, and Zhuang, X Spatial transcriptome profiling by MERFISH reveals subcellular RNA compartmentalization and cell cycle-dependent gene expression. Proc Natl Acad Sci U S A. 2019;116:19490-19499. https://doi.org/10.1073/pnas.1912459116
46. Takei, Y, Yun, J, Zheng, S, Ollikainen, N, Pierson, N, White, J, et al. Integrated spatial genomics reveals global architecture of single nuclei. Nature. 2021;590:344-350. https://doi.org/10.1038/s41586-020-03126-2
47. Ruffini, N, Klingenberg, S, Schweiger, S, and Gerber, S Common factors in neurodegeneration: A meta-study revealing shared patterns on a multi-omics scale. Cells. 2020;9:2642. https://doi.org/10.3390/cells9122642
48. International, A s D World Alzheimer Report 2019. Alzheimer's Dis. 2019.
49. Association, A s 2019 Alzheimer's disease facts and figures. AlzheimersDement. 2019;15:321-387. https://doi.org/10.1016/j.jalz.2019.01.010

50. Joe E, R J Cognitive symptoms of Alzheimer's disease: clinical management and prevention. BMJ. 2019;367:l6217. https://doi.org/10.1136/bmj.l6217
51. Scheltens, P, Blennow, K, Breteler, M M B, de Strooper, B, Frisoni, G B, Salloway, S, et al. Alzheimer's disease. The Lancet. 2021;397:1577 – 1590. https://doi.org/10.1016/s0140-6736(15)01124-1
52. Ballard, C, Gauthier, S, Corbett, A, Brayne, C, Aarsland, D, and Jones, E Alzheimer's disease. The Lancet. 2011;377:1019-1031. https://doi.org/10.1016/s0140-6736(10)61349-9
53. Scheltens, P, De Strooper, B, Kivipelto, M, Holstege, H, Chételat, G, Teunissen, C E, et al. Alzheimer's disease. The Lancet. 2021;397:1577-1590. https://doi.org/10.1016/s0140-6736(20)32205-4
54. Chen, C, Wang, J, Pan, D, Wang, X, Xu, Y, Yan, J, et al. Applications of multi-omics analysis in human diseases. MedComm. 2023;4:e315. https://doi.org/10.1002/mco2.315
55. Braak, H B, E. Neuropathological stageing of Alzheimer-related changes. Acta Neuropathologica 1991;82:239–259. https://doi.org/10.1007/BF00308809
56. Eva-Maria Mandelkow, E M Tau in Alzheimer's disease. Trends Cell Biol. 1998;8:425-427. https://doi.org/10.1016/S0962-8924(98)01368-3
57. Iqbal, K, and Grundke-Iqbal, I Neurofibrillary pathology leads to synaptic loss and not the other way around in Alzheimer disease. J Alzheimer's Dis. 2002;4:235-238. https://doi.org/10.3233/JAD-2002-4313
58. dos Santos Guilherme, M, Todorov, H, Osterhof, C, Möllerke, A, Cub, K, Hankeln, T, et al. Impact of acute and chronic amyloid-β peptide exposure on gut microbial commensals in the mouse. Front Microbiol. 2020;11:1008. https://doi.org/10.3389/fmicb.2020.01008
59. Peña-Bautista, C, Baquero, M, Vento, M, and Cháfer-Pericás, C Omics-based biomarkers for the early Alzheimer disease diagnosis and reliable therapeutic targets development. Curr Neuropharmacol. 2019;17:630-647. https://doi.org/10.2174/1570159x16666180926123722
60. Hampel, H, Nisticò, R, Seyfried, N T, Levey, A I, Modeste, E, Lemercier, P, et al. Omics sciences for systems biology in Alzheimer's disease: State-of-the-art of the evidence. Ageing Res Rev. 2021;69:101346. https://doi.org/10.1016/j.arr.2021.101346
61. Montagne, A, Nikolakopoulou, A M, Huuskonen, M T, Sagare, A P, Lawson, E J, Lazic, D, et al. APOE4 accelerates advanced-stage vascular and neurodegenerative disorder in old Alzheimer's mice via cyclophilin A independently of amyloid-β. Nat Aging. 2021;1:506-520. https://doi.org/10.1038/s43587-021-00073-z
62. Nativio, R, Donahue, G, Berson, A, Lan, Y, Amlie-Wolf, A, Tuzer, F, et al. Dysregulation of the epigenetic landscape of normal aging in Alzheimer's disease. Nat Neurosci. 2018;21:497-505. https://doi.org/10.1038/s41593-018-0101-9
63. Klein, H-U, McCabe, C, Gjoneska, E, Sullivan, S E, Kaskow, B J, Tang, A, et al. Epigenome-wide study uncovers large-scale changes in histone acetylation driven by tau pathology in aging and Alzheimer's human brains. Nat Neurosci. 2018;22:37-46. https://doi.org/10.1038/s41593-018-0291-1
64. Mostafavi, S, Gaiteri, C, Sullivan, S E, White, C C, Tasaki, S, Xu, J, et al. A molecular network of the aging human brain provides insights into the pathology and cognitive decline of Alzheimer's disease. Nat Neurosci. 2018;21:811-819. https://doi.org/10.1038/s41593-018-0154-9
65. Roberts JA, Varma VR, An Y, Varma S, Candia J, Fantoni G, et al. A brain proteomic signature of incipient Alzheimer's disease in young APOE ε4 carriers identifies novel drug targets. Sci Adv.

2021;7:eabi8178. https://doi.org/10.1126/sciadv.abi817

66. He, K, Nie, L, Zhou, Q, Rahman, S U, Liu, J, Yang, X, et al. Proteomic profiles of the early mitochondrial changes in APP/PS1 and ApoE4 transgenic mice models of Alzheimer's disease. J Proteome Res. 2019;18:2632-2642. https://doi.org/10.1021/acs.jproteome.9b00136
67. Chen, C, Jiang, X, Li, Y, Yu, H, Li, S, Zhang, Z, et al. Low-dose oral copper treatment changes the hippocampal phosphoproteomic profile and perturbs mitochondrial function in a mouse model of Alzheimer's disease. Free Radical Bio Med. 2019;135:144-156. https://doi.org/10.1016/j.freeradbiomed.2019.03.002
68. Shen, Y, Timsina, J, Heo, G, Beric, A, Ali, M, Wang, C, et al. CSF proteomics identifies early changes in autosomal dominant Alzheimer's disease. Cell. 2024;187:6309-6326.e15. https://doi.org/10.1016/j.cell.2024.08.049
69. Brayne, C, Varma, V R, Oommen, A M, Varma, S, Casanova, R, An, Y, et al. Brain and blood metabolite signatures of pathology and progression in Alzheimer disease: A targeted metabolomics study. Plos Med. 2018;15:e1002482. https://doi.org/10.1371/journal.pmed.1002482
70. Traxler, L, Herdy, J R, Stefanoni, D, Eichhorner, S, Pelucchi, S, Szücs, A, et al. Warburg-like metabolic transformation underlies neuronal degeneration in sporadic Alzheimer's disease. Cell Metab. 2022;34:1248-1263. https://doi.org/10.1016/j.cmet.2022.07.014
71. He, Y, Lu, W, Zhou, X, Mu, J, and Shen, W Unraveling Alzheimer's disease: insights from single-cell sequencing and spatial transcriptomic. Front Neurol. 2024;15:1515981. https://doi.org/10.3389/fneur.2024.1515981
72. Mathys, H, Davila-Velderrain, J, Peng, Z, Gao, F, Mohammadi, S, Young, J Z, et al. Single-cell transcriptomic analysis of Alzheimer's disease. Nature. 2019;570:332-337. https://doi.org/10.1038/s41586-019-1195-2
73. Grubman, A, Chew, G, Ouyang, J F, Sun, G, Choo, X Y, McLean, C, et al. A single-cell atlas of entorhinal cortex from individuals with Alzheimer's disease reveals cell-type-specific gene expression regulation. Nat Neurosci. 2019;22:2087-2097. https://doi.org/10.1038/s41593-019-0539-4
74. Chen, W T, Lu, A, Craessaerts, K, Pavie, B, Sala Frigerio, C, Corthout, N, et al. Spatial Transcriptomics and In Situ Sequencing to Study Alzheimer's Disease. Cell. 2020;182:976-991.e19. https://doi.org/10.1016/j.cell.2020.06.038
75. Chen, S, Chang, Y, Li, L, Acosta, D, Li, Y, Guo, Q, et al. Spatially resolved transcriptomics reveals genes associated with the vulnerability of middle temporal gyrus in Alzheimer's disease. Acta Neuropathol Commun. 2022;10:188. https://doi.org/10.1186/s40478-022-01494-6
76. Zou, D, Huang, X, Lan, Y, Pan, M, Xie, J, Huang, Q, et al. Single-cell and spatial transcriptomics reveals that PTPRG activates the m(6)A methyltransferase VIRMA to block mitophagy-mediated neuronal death in Alzheimer's disease. Pharmacol Res. 2024;201:107098. https://doi.org/10.1016/j.phrs.2024.107098
77. Zupanic, A, Bernstein, H C, and Heiland, I Systems biology: current status and challenges. Cell Mol Life Sci. 2020;77:379-380. https://doi.org/10.1007/s00018-019-03410-z
78. Johnson, E C B, Carter, E K, Dammer, E B, Duong, D M, Gerasimov, E S, Liu, Y, et al. Large-scale deep multi-layer analysis of Alzheimer's disease brain reveals strong proteomic disease-related changes not observed at the RNA level. Nat Neurosci. 2022;25:213-225. https://doi.org/10.1038/s41593-021-00999-y
79. Madrid L, Moreno-Grau S, Ahmad S, G-P A, de Rojas I, X R, Martino Adami PV, García-Gonzá

lez P, et al. Multiomics integrative analysis identifies APOE allele-specific blood biomarkers associated to Alzheimers disease etiopathogenesis. Aging. 2021;13:9277 – 9329. https://doi.org/10.18632/aging.202950
80. Horgusluoglu, E, Neff, R, Song, W M, Wang, M, Wang, Q, Arnold, M, et al. Integrative metabolomics-genomics approach reveals key metabolic pathways and regulators of Alzheimer's disease. AlzheimersDement. 2021;18:1260-1278. https://doi.org/10.1002/alz.12468
81. San Segundo-Acosta, P, Montero-Calle, A, Jernbom-Falk, A, Alonso-Navarro, M, Pin, E, Andersson, E, et al. Multiomics profiling of Alzheimer's disease serum for the identification of autoantibody biomarkers. J Proteome Res. 2021;20:5115-5130. https://doi.org/10.1021/acs.jproteome.1c00630
82. Wang, H, Robinson, J L, Kocabas, P, Gustafsson, J, Anton, M, Cholley, P-E, et al. Genome-scale metabolic network reconstruction of model animals as a platform for translational research. P Natl A Sci. 2021;118:e2102344118. https://doi.org/10.1073/pnas.2102344118
83. Zhang, J, Sun, X, Jia, X, Sun, B, Xu, S, Zhang, W, et al. Integrative multi-omics analysis reveals the critical role of the PBXIP1 gene in Alzheimer's disease. Aging Cell. 2023;23:e14044. https://doi.org/10.1111/acel.14044
84. Marzi, S J, Leung, S K, Ribarska, T, Hannon, E, Smith, A R, Pishva, E, et al. A histone acetylome-wide association study of Alzheimer's disease identifies disease-associated H3K27ac differences in the entorhinal cortex. Nat Neurosci. 2018;21:1618-1627. https://doi.org/10.1038/s41593-018-0253-7
85. Nativio, R, Lan, Y, Donahue, G, Sidoli, S, Berson, A, Srinivasan, A R, et al. An integrated multi-omics approach identifies epigenetic alterations associated with Alzheimer's disease. Nat Genet. 2020;52:1024-1035. https://doi.org/10.1038/s41588-020-0696-0
86. Clark, C, Dayon, L, Masoodi, M, Bowman, G L, and Popp, J An integrative multi-omics approach reveals new central nervous system pathway alterations in Alzheimer's disease. Alzheimer's Res Ther. 2021;13:71. https://doi.org/10.1186/s13195-021-00814-7
87. Strefeler, A, Jan, M, Quadroni, M, Teav, T, Rosenberg, N, Chatton, J-Y, et al. Molecular insights into sex-specific metabolic alterations in Alzheimer's mouse brain using multi-omics approach. Alzheimer's Res Ther. 2023;15:8. https://doi.org/10.1186/s13195-023-01162-4
88. Wang, C, Lu, J, Sha, X, Qiu, Y, Chen, H, and Yu, Z TRPV1 regulates ApoE4-disrupted intracellular lipid homeostasis and decreases synaptic phagocytosis by microglia. Experimental & Molecular Medicine. 2023;55:347-363. https://doi.org/10.1038/s12276-023-00935-z
89. Wang, M, Li, A, Sekiya, M, Beckmann, N D, Quan, X, Schrode, N, et al. Transformative network modeling of multi-omics data reveals detailed circuits, key regulators, and potential therapeutics for Alzheimer ' s disease. Neuron. **2021**;109:257-272.e14. https://doi.org/10.1016/j.neuron.2020.11.002
90. Zhou, Y, Fang, J, Bekris, L M, Kim, Y H, Pieper, A A, Leverenz, J B, et al. AlzGPS: a genome-wide positioning systems platform to catalyze multi-omics for Alzheimer's drug discovery. Alzheimer's Res Ther. 2021;13:24. https://doi.org/10.1186/s13195-020-00760-w
91. Alexander, G E Biology of Parkinson's disease: Pathogenesis and pathophysiology of a multisystem neurodegenerative disorder. Dialogues Clin Neurosci. 2004;6(3):259 – 280. https://doi.org/10.31887/DCNS.2004.6.3/galexander
92. A. Berardelli, J C R, P. D. Thompson and M. Hallett Pathophysiology of bradykinesia in Parkinson's disease. Brain. 2001;124:2131–2146. https://doi.org/10.1093/brain/124.11.2131

93. William Dauer, a S P Parkinson's disease mechanisms and models. Neuron. 2003;39:889–909. https://doi.org/10.1016/s0896-6273(03)00568-3
94. Wright Willis, A, Evanoff, B A, Lian, M, Criswell, S R, and Racette, B A Geographic and ethnic variation in Parkinson disease: A population-based study of US medicare beneficiaries. Neuroepidemiology. 2010;34:143-151. https://doi.org/10.1159/000275491
95. Kempuraj D, Thangavel R, Natteru PA, Selvakumar GP, Saeed D, Zahoor H, et al. Neuroinflammation induces neurodegeneration. J Neurol Neurosurg Spine. 2016;1:1003.
96. Gao, T, Zheng, R, Ruan, Y, Fang, Y, Jin, C, Cao, J, et al. Association of ZNF184, IL1R2, LRRK2, ITPKB, and PARK16 with sporadic Parkinson's disease in Eastern China. Neurosci Lett. 2020;735:135261. https://doi.org/10.1016/j.neulet.2020.135261
97. Witoelar, A, Jansen, I E, Wang, Y, Desikan, R S, Gibbs, J R, Blauwendraat, C, et al. Genome-wide pleiotropy between Parkinson disease and autoimmune diseases. JAMA Neurol. 2017;74:780–792. https://doi.org/10.1001/jamaneurol.2017.0469
98. Zhang, W, Shen, J, Wang, Y, Cai, K, Zhang, Q, and Cao, M Blood SSR1: A possible biomarker for early prediction of Parkinson's disease. Front Mol Neurosci. 2022;15:762544. https://doi.org/10.3389/fnmol.2022.762544
99. Karayel, O, Virreira Winter, S, Padmanabhan, S, Kuras, Y I, Vu, D T, Tuncali, I, et al. Proteome profiling of cerebrospinal fluid reveals biomarker candidates for Parkinson's disease. Cell Rep Med. 2022;3:100661. https://doi.org/10.1016/j.xcrm.2022.100661
100. Sinclair, E, Trivedi, D K, Sarkar, D, Walton-Doyle, C, Milne, J, Kunath, T, et al. Metabolomics of sebum reveals lipid dysregulation in Parkinson's disease. Nat Commun. 2021;12:1592. https://doi.org/10.1038/s41467-021-21669-4
101. Tan, A H, Chong, C W, Lim, S-Y, Yap, I K S, Teh, C S J, Loke, M F, et al. Gut microbial ecosystem in Parkinson disease: new clinicobiological insights from multi-omics. Ann Neurol. 2020;89:546–559. https://doi.org/10.1002/ana.25982
102. Pereira, P A B, Trivedi, D K, Silverman, J, Duru, I C, Paulin, L, Auvinen, P, et al. Multiomics implicate gut microbiota in altered lipid and energy metabolism in Parkinson's disease. npj Parkinson's Dis. 2022;8:39. https://doi.org/10.1038/s41531-022-00300-3
103. Luan, H, Liu, L-F, Meng, N, Tang, Z, Chua, K-K, Chen, L-L, et al. LC–MS-Based urinary metabolite signatures in idiopathic Parkinson's disease. J Proteome Res. 2014;14:467-478. https://doi.org/10.1021/pr500807t
104. Luan, H, Liu, L-F, Tang, Z, Zhang, M, Chua, K-K, Song, J-X, et al. Comprehensive urinary metabolomic profiling and identification of potential noninvasive marker for idiopathic Parkinson's disease. Sci Rep-uk. 2015;5:13888. https://doi.org/10.1038/srep13888
105. Mirzac, D, Bange, M, Kunz, S, de Jager, P L, Groppa, S, and Gonzalez-Escamilla, G Targeting pathological brain activity-related to neuroinflammation through scRNA-seq for new personalized therapies in Parkinson's disease. Signal Transduct Target Ther. 2025;10:10. https://doi.org/10.1038/s41392-024-02086-7
106. Kamath, T, Abdulraouf, A, Burris, S J, Langlieb, J, Gazestani, V, Nadaf, N M, et al. Single-cell genomic profiling of human dopamine neurons identifies a population that selectively degenerates in Parkinson's disease. Nat Neurosci. 2022;25:588-595. https://doi.org/10.1038/s41593-022-01061-1
107. Zhu, B, Park, J M, Coffey, S R, Russo, A, Hsu, I U, Wang, J, et al. Single-cell transcriptomic and proteomic analysis of Parkinson's disease brains. Sci Transl Med. 2024;16:eabo1997.

https://doi.org/10.1126/scitranslmed.abo1997
108. Gu, X-J, Su, W-M, Dou, M, Jiang, Z, Duan, Q-Q, Yin, K-F, et al. Expanding causal genes for Parkinson ' s disease via multi-omics analysis. npj Parkinson's Dis. 2023;9:146. https://doi.org/10.1038/s41531-023-00591-0
109. Rutledge, J, Lehallier, B, Zarifkar, P, Losada, P M, Shahid-Besanti, M, Western, D, et al. Comprehensive proteomics of CSF, plasma, and urine identify DDC and other biomarkers of early Parkinson's disease. Acta Neuropathol. 2024;147:52. https://doi.org/10.1007/s00401-024-02706-0
110. Hu, J, Li, P, Han, H, Ji, P, Zhao, X, and Li, Z Integrated analysis of metabolomic and transcriptomic profiling reveals the effect of Buyang Huanwu decoction on Parkinson's disease in mice. Phytomedicine. 2023;114:154755. https://doi.org/10.1016/j.phymed.2023.154755
111. Cheng, Q, Wang, J, Li, M, Fang, J, Ding, H, Meng, J, et al. CircSV2b participates in oxidative stress regulation through miR-5107-5p-Foxk1-Akt1 axis in Parkinson's disease. Redox Biol. 2022;56:102430. https://doi.org/10.1016/j.redox.2022.102430
112. Organization, W H Epilepsy: A Public Health Imperative. Epilepsy. 2019;1:171.
113. Kwon, C A-O, Wagner, R A-O, Carpio, A A-O, Jetté, N A-O, Newton, C A-O, and Thurman, D A-O The worldwide epilepsy treatment gap: A systematic review and recommendations for revised definitions - A report from the ILAE Epidemiology Commission. Epilepsia. 63:551 – 564. https://doi.org/10.1111/epi.17112
114. Scheffer, I E, Berkovic, S, Capovilla, G, Connolly, M B, French, J, Guilhoto, L, et al. ILAE classification of the epilepsies: Position paper of the ILAE commission for classification and terminology. Epilepsia. 2017;58:512-521. https://doi.org/10.1111/epi.13709
115. Okamoto, O K, Janjoppi, L, Bonone, F M, Pansani, A P, da Silva, A V, Scorza, F A, et al. Whole transcriptome analysis of the hippocampus: toward a molecular portrait of epileptogenesis. BMC Genomics. 2010;11:230. https://doi.org/10.1186/1471-2164-11-230
116. Chen, Q L, Xia, L, Zhong, S P, Wang, Q, Ding, J, and Wang, X Bioinformatic analysis identifies key transcriptome signatures in temporal lobe epilepsy. CNS Neurosci Ther. 2020;26:1266-1277. https://doi.org/10.1111/cns.13470
117. Biagini, G, Keren-Aviram, G, Dachet, F, Bagla, S, Balan, K, Loeb, J A, et al. Proteomic analysis of human epileptic neocortex predicts vascular and glial changes in epileptic regions. Plos One. 2018;13:e0195639. https://doi.org/10.1371/journal.pone.0195639
118. Sadeghi, L, Rizvanov, A A, Dabirmanesh, B, Salafutdinov, I I, Sayyah, M, Shojaei, A, et al. Proteomic profiling of the rat hippocampus from the kindling and pilocarpine models of epilepsy: potential targets in calcium regulatory network. Sci Rep-uk. 2021;11:8252. https://doi.org/10.1038/s41598-021-87555-7
119. Han, Q-T, Yang, W-Q, Zang, C, Zhou, L, Zhang, C-J, Bao, X, et al. The toxic natural product tutin causes epileptic seizures in mice by activating calcineurin. Signal Transduction Targeted Ther. 2023;8:101. https://doi.org/10.1038/s41392-023-01312-y
120. Boguszewicz, Ł, Jamroz, E, Ciszek, M, Emich-Widera, E, Kijonka, M, Banasik, T, et al. NMR-based metabolomics in pediatric drug resistant epilepsy – preliminary results. Sci Rep-uk. 2019;9:15035. https://doi.org/10.1038/s41598-019-51337-z
121. Hamelin, S, Stupar, V, Mazière, L, Guo, J, Labriji, W, Liu, C, et al. In vivo γ-aminobutyric acid increase as a biomarker of the epileptogenic zone: An unbiased metabolomics approach. Epilepsia. 2020;62:163-175. https://doi.org/10.1111/epi.16768
122. Pfisterer, U, Petukhov, V, Demharter, S, Meichsner, J, Thompson, J J, Batiuk, M Y, et al.

Identification of epilepsy-associated neuronal subtypes and gene expression underlying epileptogenesis. Nat Commun. 2020;11:5038. https://doi.org/10.1038/s41467-020-18752-7

123. Wen, F, Tan, Z, Huang, D, and Xiang, J Molecular mechanism analyses of post-traumatic epilepsy and hereditary epilepsy based on 10× single-cell transcriptome sequencing technology. CNS Neurosci Ther. 2024;30:e14702. https://doi.org/10.1111/cns.14702
124. Liu, Q, Shen, C, Dai, Y, Tang, T, Hou, C, Yang, H, et al. Single-cell, single-nucleus and xenium-based spatial transcriptomics analyses reveal inflammatory activation and altered cell interactions in the hippocampus in mice with temporal lobe epilepsy. Biomark Res. 2024;12:103. https://doi.org/10.1186/s40364-024-00636-3
125. Johnson, M R, Behmoaras, J, Bottolo, L, Krishnan, M L, Pernhorst, K, Santoscoy, P L M, et al. Systems genetics identifies Sestrin 3 as a regulator of a proconvulsant gene network in human epileptic hippocampus. Nat Commun. 2015;6:6031. https://doi.org/10.1038/ncomms7031
126. Sharma, S, Sharma, M, Rana, A K, Joshi, R, Swarnkar, M K, Acharya, V, et al. Deciphering key regulators involved in epilepsy‐induced cardiac damage through whole transcriptome and proteome analysis in a rat model. Epilepsia. 2020;62:504-516. https://doi.org/10.1111/epi.16794
127. Harutyunyan, A, Chong, D, Li, R, Shah, A D, Ali, Z, Huang, C, et al. An integrated multi-omic network analysis identifies seizure-associated dysregulated pathways in the GAERS model of absence epilepsy. Int J Mol Sci. 2022;23:6063. https://doi.org/10.3390/ijms23116063
128. Wu, H C, Dachet, F, Ghoddoussi, F, Bagla, S, Fuerst, D, Stanley, J A, et al. Altered metabolomic–genomic signature: A potential noninvasive biomarker of epilepsy. Epilepsia. 2017;58:1626-1636. https://doi.org/10.1111/epi.13848
129. Venø, M T, Reschke, C R, Morris, G, Connolly, N M C, Su, J, Yan, Y, et al. A systems approach delivers a functional microRNA catalog and expanded targets for seizure suppression in temporal lobe epilepsy. P Natl A Sci. 2020;117:15977-15988. https://doi.org/10.1073/pnas.1919313117
130. Tian, D-C, Zhang, C, Yuan, M, Yang, X, Gu, H, Li, Z, et al. Incidence of multiple sclerosis in China: A nationwide hospital-based study. The Lancet Regional Health Western Pacific. 2020;1:100010. https://doi.org/10.1016/j.lanwpc.2020.100010
131. Zhou, Q, Zhang, T, Meng, H, Shen, D, Li, Y, He, L, et al. Characteristics of cerebral blood flow in an Eastern sample of multiple sclerosis patients: A potential quantitative imaging marker associated with disease severity. Front Immunol. 2022;13:1025908. https://doi.org/10.3389/fimmu.2022.1025908
132. Weinshenker BG, B B, Rice GP, Noseworthy J, Carriere W, Baskerville J, Ebers GC The natural history of multiple sclerosis: a geographically based study. 2. Predictive value of the early clinical course. Brain. 1989;112:1419–1428. https://doi.org/10.1093/brain/112.6.1419
133. Waubant, E, Lucas, R, Mowry, E, Graves, J, Olsson, T, Alfredsson, L, et al. Environmental and genetic risk factors for MS: an integrated review. Ann Clin Transl Neurol. 2019;6:1905-1922. https://doi.org/10.1002/acn3.50862
134. Galea, I, Ward-Abel, N, and Heesen, C Relapse in multiple sclerosis. BMJ: British Medical Journal. 2015;350:h1765. https://doi.org/10.1136/bmj.h1765
135. Arneth, B, and Kraus, J Laboratory biomarkers of multiple sclerosis (MS). Clin Biochem. 2022;99:1-8. https://doi.org/10.1016/j.clinbiochem.2021.10.004
136. Åkesson, J, Hojjati, S, Hellberg, S, Raffetseder, J, Khademi, M, Rynkowski, R, et al. Proteomics reveal biomarkers for diagnosis, disease activity and long-term disability outcomes in multiple sclerosis. Nat Commun. 2023;14(1):6903. https://doi.org/10.1038/s41467-023-42682-9

137. Peng, H-r, Qiu, J-Q, Zhou, Q-m, Zhang, Y-k, Chen, Q-y, Yin, Y-q, et al. Intestinal epithelial dopamine receptor signaling drives sex-specific disease exacerbation in a mouse model of multiple sclerosis. Immun. 2023;56:2773-2789. https://doi.org/10.1016/j.immuni.2023.10.016
138. Insha Zahoora, H S, 1, Indrani Dattab, Mohammad Ejaz Ahmeda, Laila M. Poissonb, Jeffrey Watersa, Faraz Rashida, Rui Bina, Jaspreet Singha,Mirela Cerghet, Ashok Kumarc, Md Nasrul Hodaa, Ramandeep Rattand, Ashutosh K. Mangalamand Shailendra Giri Blood-based untargeted metabolomics in relapsing-remitting multiple sclerosis revealed the testable therapeutic target. P Natl A Sci. 119:e2123265119. https://doi.org/10.1073/pnas
139. Schafflick, D, Xu, C A, Hartlehnert, M, Cole, M, Schulte-Mecklenbeck, A, Lautwein, T, et al. Integrated single cell analysis of blood and cerebrospinal fluid leukocytes in multiple sclerosis. Nat Commun. 2020;11:247. https://doi.org/10.1038/s41467-019-14118-w
140. Kukanja, P, Langseth, C M, Rubio Rodríguez-Kirby, L A, Agirre, E, Zheng, C, Raman, A, et al. Cellular architecture of evolving neuroinflammatory lesions and multiple sclerosis pathology. Cell. 2024;187:1990-2009.e19. https://doi.org/https://doi.org/10.1016/j.cell.2024.02.030
141. Elkjaer, M L, Hartebrodt, A, Oubounyt, M, Weber, A, Vitved, L, Reynolds, R, et al. Single-Cell Multi-Omics Map of Cell Type-Specific Mechanistic Drivers of Multiple Sclerosis Lesions. Neurology(R) neuroimmunology & neuroinflammation. 2024;11:e200213. https://doi.org/10.1212/nxi.0000000000200213
142. Li, Z, Rius Rigau, A, Xie, W, Huang, L, Ye, W, Li, Y N, et al. Spatial multiomics decipher fibroblast-macrophage dynamics in systemic sclerosis. Ann Rheum Dis. 2025;84:1231-1245. https://doi.org/10.1016/j.ard.2025.04.025
143. Kaufmann, M, Schaupp, A-L, Sun, R, Coscia, F, Dendrou, C A, Cortes, A, et al. Identification of early neurodegenerative pathways in progressive multiple sclerosis. Nat Neurosci. 2022;25:944-955. https://doi.org/10.1038/s41593-022-01097-3
144. Wu, T, Ning, S, Zhang, H, Cao, Y, Li, X, Hao, J, et al. Role of ferroptosis in neuroimmunity and neurodegeneration in multiple sclerosis revealed by multi‐omics data. J Cell Mol Med. 2024;28:e18396. https://doi.org/10.1111/jcmm.18396
145. Yang, F, Zhao, L-Y, Yang, W-Q, Chao, S, Ling, Z-X, Sun, B-Y, et al. Quantitative proteomics and multi-omics analysis identifies potential biomarkers and the underlying pathological molecular networks in Chinese patients with multiple sclerosis. BMC Neurol. 2024;24:423. https://doi.org/10.1186/s12883-024-03926-3
146. Zhou, Q, Xie, Z, He, L, Sun, G, Meng, H, Luo, Z, et al. Multi-omics profiling reveals peripheral blood biomarkers of multiple sclerosis: implications for diagnosis and stratification. Front Pharmacol. 2024;15:1458046. https://doi.org/10.3389/fphar.2024.1458046
147. Benjamin, E J, Muntner, P, Alonso, A, Bittencourt, M S, Callaway, C W, Carson, A P, et al. Heart disease and stroke statistics—2019 update: A report from the american heart association. Circulation. 2019;139:e56–e528. https://doi.org/10.1161/cir.0000000000000659
148. Tu, W-J, Zhao, Z, Yin, P, Cao, L, Zeng, J, Chen, H, et al. Estimated burden of stroke in china in 2020. JAMA Netw Open. 2023;6:e231455. https://doi.org/10.1001/jamanetworkopen.2023.1455
149. Eloy Cuadrado, Anna Rosell, Nuria Colomé, Mar Hernández-Guillamon, Teresa García-Berrocoso, Marc Ribo, et al. The proteome of human brain after ischemic stroke. J Neuropath Exp Neur. 2010;69:1105–1115. https://doi.org/10.1097/NEN.0b013e3181f8c539
150. Simats, A, García-Berrocoso, T, Ramiro, L, Giralt, D, Gill, N, Penalba, A, et al. Characterization of the rat cerebrospinal fluid proteome following acute cerebral ischemia using an aptamer-based

proteomic technology. Sci Rep-uk. 2018;8:7899. https://doi.org/10.1038/s41598-018-26237-3
151. García-Berrocoso, T, Llombart, V, Colàs-Campàs, L, Hainard, A, Licker, V, Penalba, A, et al. Single cell immuno-laser microdissection coupled to label-free proteomics to reveal the proteotypes of human brain cells after ischemia. Mol Cell Proteomics. 2018;17:175-189. https://doi.org/10.1074/mcp.RA117.000419
152. Dykstra-Aiello, C, Jickling, G C, Ander, B P, Shroff, N, Zhan, X, Liu, D, et al. Altered expression of long noncoding RNAs in blood after ischemic stroke and proximity to putative stroke risk loci. Stroke. 2016;47:2896-2903. https://doi.org/10.1161/strokeaha.116.013869
153. Deng, Q-W, Li, S, Wang, H, Sun, H-L, Zuo, L, Gu, Z-T, et al. Differential long noncoding RNA expressions in peripheral blood mononuclear cells for detection of acute ischemic stroke. Clin Sci. 2018;132:1597-1614. https://doi.org/10.1042/cs20180411
154. Wang, W, Gao, F, Zhao, Z, Wang, H, Zhang, L, Zhang, D, et al. Integrated analysis of LncRNA-mRNA co-expression profiles in patients with moyamoya disease. Sci Rep-uk. 2017;7:42421. https://doi.org/10.1038/srep42421
155. Holdt, L M, and Teupser, D Long noncoding RNA ANRIL: Lnc-ing genetic variation at the chromosome 9p21 locus to molecular mechanisms of atherosclerosis. Front Cardiovasc Med. 2018;5:145. https://doi.org/10.3389/fcvm.2018.00145
156. Wang, J, Ruan, J, Zhu, M, Yang, J, Du, S, Xu, P, et al. Predictive value of long noncoding RNA ZFAS1 in patients with ischemic stroke. Clin Exp Hypertens. **2018**;41:615-621. https://doi.org/10.1080/10641963.2018.1529774
157. Li, P, Teng, F, Gao, F, Zhang, M, Wu, J, and Zhang, C Identification of circulating microRNAs as potential biomarkers for detecting acute ischemic stroke. Cell Mol Neurobiol. 2015;35:433-447. https://doi.org/10.1007/s10571-014-0139-5
158. Kimberly, W T, Wang, Y, Pham, L, Furie, K L, and Gerszten, R E Metabolite profiling identifies a branched chain amino acid signature in acute cardioembolic stroke. Stroke. 2013;44:1389-1395. https://doi.org/10.1161/strokeaha.111.000397
159. Choi JY, Kim JS, Kim JH, Oh K, Koh SB, and WK., S High free fatty acid level is associated with recurrent stroke in cardioembolic stroke patients. Neurology. 2014;82:1142 – 1148. https://doi.org/10.1212/WNL.0000000000000264
160. Jin, C, Shi, Y, Shi, L, Leak, R K, Zhang, W, Chen, K, et al. Leveraging single-cell RNA sequencing to unravel the impact of aging on stroke recovery mechanisms in mice. Proc Natl Acad Sci U S A. 2023;120:e2300012120. https://doi.org/10.1073/pnas.2300012120
161. Ma, Y, Zheng, K, Zhao, C, Chen, J, Chen, L, Zhang, Y, et al. Microglia LILRB4 upregulation reduces brain damage after acute ischemic stroke by limiting CD8(+) T cell recruitment. J Neuroinflammation. 2024;21:214. https://doi.org/10.1186/s12974-024-03206-4
162. Li, S, Zeng, G, Pang, C, Li, J, Wu, L, Luo, M, et al. Single-cell and spatial transcriptomics analysis reveals that Pros1(+) oligodendrocytes are involved in endogenous neuroprotection after brainstem stroke. Neurobiol Dis. 2025;208:106855. https://doi.org/10.1016/j.nbd.2025.106855
163. Gu, L, Chen, H, Sun, M, Chen, Y, Shi, Q, Chang, J, et al. Unraveling dynamic immunological landscapes in intracerebral hemorrhage: insights from single-cell and spatial transcriptomic profiling. MedComm. 2024;5:e635. https://doi.org/10.1002/mco2.635
164. Gu, L, Chen, H, Geng, R, Sun, M, Shi, Q, Chen, Y, et al. Single-cell and Spatial Transcriptomics Reveals Ferroptosis as The Most Enriched Programmed Cell Death Process in Hemorrhage Stroke-induced Oligodendrocyte-mediated White Matter Injury. Int J Biol Sci. 2024;20:3842-3862.

https://doi.org/10.7150/ijbs.96262
165. Han, B, Zhou, S, Zhang, Y, Chen, S, Xi, W, Liu, C, et al. Integrating spatial and single-cell transcriptomics to characterize the molecular and cellular architecture of the ischemic mouse brain. Sci Transl Med. 2024;16:eadg1323. https://doi.org/10.1126/scitranslmed.adg1323
166. Wang, X, Zhang, A, Yu, Q, Wang, Z, Wang, J, Xu, P, et al. Single-Cell RNA Sequencing and Spatial Transcriptomics Reveal Pathogenesis of Meningeal Lymphatic Dysfunction after Experimental Subarachnoid Hemorrhage. Adv Sci (Weinh). 2023;10:e2301428. https://doi.org/10.1002/advs.202301428
167. Zhang, C, Qin, F, Li, X, Du, X, and Li, T Identification of novel proteins for lacunar stroke by integrating genome-wide association data and human brain proteomes. BMC Med. 2022;20:211. https://doi.org/10.1186/s12916-022-02408-y
168. Wang, R, Liu, M, Ren, G, Luo, G, Wang, Z, Ge, Z, et al. Zhilong Huoxue Tongyu capsules' effects on ischemic stroke: An assessment using fecal 16S rRNA gene sequencing and untargeted serum metabolomics. Front Pharmacol. 2022;13:1052110. https://doi.org/10.3389/fphar.2022.1052110
169. Yuan, Y, Sheng, P, Ma, B, Xue, B, Shen, M, Zhang, L, et al. Elucidation of the mechanism of Yiqi Tongluo Granule against cerebral ischemia/reperfusion injury based on a combined strategy of network pharmacology, multi-omics and molecular biology. Phytomedicine. 2023;118:154934. https://doi.org/10.1016/j.phymed.2023.154934
170. Hochstetler, A, Raskin, J, and Blazer-Yost, B L Hydrocephalus: historical analysis and considerations for treatment. Eur J Med Res. 2022;27:168. https://doi.org/10.1186/s40001-022-00798-6
171. Rekate, H L A contemporary Ddefinition and classification of hydrocephalus. Semin Pediatr Neurol. 2009;16:9-15. https://doi.org/10.1016/j.spen.2009.01.002
172. Reith, W Hydrozephalus. Der Radiologe. 2012;52:805-806. https://doi.org/10.1007/s00117-012-2329-9
173. Whitelaw, A, Brion, L P, Kennedy, C R, and Odd, D Diuretic therapy for newborn infants with posthemorrhagic ventricular dilatation. Cochrane Db Syst Rev. 2001;2001:CD002270. https://doi.org/10.1002/14651858.Cd002270
174. Kahle, K T, Kulkarni, A V, Limbrick, D D, and Warf, B C Hydrocephalus in children. The Lancet. 2016;387:788-799. https://doi.org/10.1016/s0140-6736(15)60694-8
175. Anwar, F, Zhang, K, Sun, C, Pang, M, Zhou, W, Li, H, et al. Hydrocephalus: An update on latest progress in pathophysiological and therapeutic research. Biomed Pharmacother. 2024;181:117702. https://doi.org/10.1016/j.biopha.2024.117702
176. Xiuyun Liu , H Z, Marek Czosnyka , Chiara Robba , Zofia Czosnyka , Jennifer Lee Summers , Huijie Yu , Xiaoguang Tong , Guoyi Gao , Gelei Xiao , Kai Yu , Yan Xing , Renling Mao , Shaoya Yin , Yangong Chao , Hongliang Li , Ke Pu , Keke Feng , Meijun Pang , Dong Ming. Advancing hydrocephalus management: pathogenesis insights, therapeutic innovations, and emerging challenges. Aging Dis. 2025:10.14336/AD.2024.1434. https://doi.org/10.14336/AD.2024.1434
177. Jin, S C, Dong, W, Kundishora, A J, Panchagnula, S, Moreno-De-Luca, A, Furey, C G, et al. Exome sequencing implicates genetic disruption of prenatal neuro-gliogenesis in sporadic congenital hydrocephalus. Nat Med. 2020;26:1754-1765. https://doi.org/10.1038/s41591-020-1090-2
178. Yuan, L, Zou, D, Yang, X, Chen, X, Lu, Y, Zhang, A, et al. Proteomics and functional study reveal kallikrein-6 enhances communicating hydrocephalus. Clin Proteomics. 2021;18:30. https://doi.org/10.1186/s12014-021-09335-9

Multi-omics technologies integration

179. Ying, Y, Lin, J, Gao, W, Yue, L, Zeng, Q, Bartas, K, et al. Proteomic profiling in cerebrospinal fluid reveal biomarkers for shunt outcome in idiopathic normal-pressure hydrocephalus. J Adv Res. 2025;S2090-12329250:00287-5. https://doi.org/https://doi.org/10.1016/j.jare.2025.04.043
180. Nagata, Y, Bundo, M, Sugiura, S, Kamita, M, Ono, M, Hattori, K, et al. PTPRQ as a potential biomarker for idiopathic normal pressure hydrocephalus. Mol Med Rep. 2017;16:3034-3040. https://doi.org/10.3892/mmr.2017.7015
181. Nagata, Y, Hirayama, A, Ikeda, S, Shirahata, A, Shoji, F, Maruyama, M, et al. Comparative analysis of cerebrospinal fluid metabolites in Alzheimer's disease and idiopathic normal pressure hydrocephalus in a Japanese cohort. Biomark Res. 2018;6:5. https://doi.org/10.1186/s40364-018-0119-x
182. Wang, Z, Nie, X, Gao, F, Tang, Y, Ma, Y, Zhang, Y, et al. Increasing brain N-acetylneuraminic acid alleviates hydrocephalus-induced neurological deficits. CNS Neurosci Ther. 2023;29:3183-3198. https://doi.org/10.1111/cns.14253
183. Li, Y, Di, C, Song, S, Zhang, Y, Lu, Y, Liao, J, et al. Choroid plexus mast cells drive tumor-associated hydrocephalus. Cell. 2023;186:5719-5738.e28. https://doi.org/https://doi.org/10.1016/j.cell.2023.11.001
184. Hale, A T, Bastarache, L, Morales, D M, Wellons, J C, Limbrick, D D, and Gamazon, E R Multi-omic analysis elucidates the genetic basis of hydrocephalus. Cell Rep. 2021;35:109085. https://doi.org/10.1016/j.celrep.2021.109085
185. Chen, J, Wang, L, Peng, X, Cheng, T, Yang, Y, Su, J, et al. Identification of CSPG4 as a biomarker and therapeutic target for infantile post-hemorrhagic hydrocephalus via multi-omics analysis. Adv Sci. 2024;12:e2410056. https://doi.org/10.1002/advs.202410056
186. Tarazona, S, Balzano-Nogueira, L, Gómez-Cabrero, D, Schmidt, A, Imhof, A, Hankemeier, T, et al. Harmonization of quality metrics and power calculation in multi-omic studies. Nat Commun. 2020;11:3092. https://doi.org/10.1038/s41467-020-16937-8
187. Antonelli, J, Claggett, B L, Henglin, M, Kim, A, Ovsak, G, Kim, N, et al. Statistical workflow for feature selection in human metabolomics Data. Metabolites. 2019;9:143. https://doi.org/10.3390/metabo9070143
188. Liew, A W C, Law, N F, and Yan, H Missing value imputation for gene expression data: computational techniques to recover missing data from available information. Briefings Bioinf. 2010;12:498-513. https://doi.org/10.1093/bib/bbq080
189. Vivian J, Eizenga JM, Beale HC, Vaske OM, and B., P Bayesian framework for detecting gene expression outliers in individual samples. JCO Clin Cancer Inform. 2020;4:160 – 170. https://doi.org/10.1200/CCI.19.00095
190. Liu, L, Chen, A, Li, Y, Mulder, J, Heyn, H, and Xu, X Spatiotemporal omics for biology and medicine. Cell. 2024;187:4488-4519. https://doi.org/10.1016/j.cell.2024.07.040
191. Lim, J, Park, C, Kim, M, Kim, H, Kim, J, and Lee, D-S Advances in single-cell omics and multiomics for high-resolution molecular profiling. Experimental & Molecular Medicine. 2024;56:515-526. https://doi.org/10.1038/s12276-024-01186-2
192. Park, H J, Park, B, and Lee, S S Radiomics and deep learning: hepatic applications. Korean J Radiol. 2020;21:387-401. https://doi.org/10.3348/kjr.2019.0752
193. Xu, J L, Mao, C S, Hou, Y, Luo, Y, Binder, J L, Zhou, Y D, et al. Interpretable deep learning translation of GWAS and multi-omics findings to identify pathobiology and drug repurposing in Alzheimer?s disease. Cell Rep. 2022;41:26. https://doi.org/10.1016/j.celrep.2022.111717

Multi-omics technologies integration

194. He, X J, Liu, X W, Zuo, F L, Shi, H B, and Jing, J Artificial intelligence-based multi-omics analysis fuels cancer precision medicine. Semin Cancer Biol. 2023;88:187-200. https://doi.org/10.1016/j.semcancer.2022.12.009

195. Michetti, F, Clementi, M E, Di Liddo, R, Valeriani, F, Ria, F, Rende, M, et al. The S100B protein: A multifaceted pathogenic factor more than a biomarker. Int J Mol Sci. 2023;24. https://doi.org/10.3390/ijms24119605

196. Vignoli, A, Paciotti, S, Tenori, L, Eusebi, P, Biscetti, L, Chiasserini, D, et al. Fingerprinting Alzheimer's disease by 1H nuclear magnetic resonance spectroscopy of cerebrospinal fluid. J Proteome Res. 2020;19:1696-1705. https://doi.org/10.1021/acs.jproteome.9b00850

197. Smajić, S, Prada-Medina, C A, Landoulsi, Z, Ghelfi, J, Delcambre, S, Dietrich, C, et al. Single-cell sequencing of human midbrain reveals glial activation and a Parkinson-specific neuronal state. Brain. 2022;145:964-978. https://doi.org/10.1093/brain/awab446

Multi-omics technologies integration

Table 1: Overview of the Four Omics Technologies.

Technology	Precision	Price Range	Advantages	Disadvantages	Disease Application Representation
**Genomics**	High	High	· Conduct a comprehensive analysis of genetic sequences.<br>· Uncover the depth of genetic variations.<br>· Be suitable for gene discovery and genetic disease studies.	· High experimental costs.<br>· Complex techniques and large sample sizes.<br>· Considerable time in analysis.	· Genetic disorders.<br>· Cancer genomics.<br>· Genetic counseling.
**Transcriptomics**	Medium	Medium	· Reveal dynamic changes in gene expression.<br>· Differentiate gene regulatory networks.<br>· Assist in the classification of disease subtypes.	· High experimental and data analysis design.<br>· Limited real-time to reflect mRNA levels.	· Mental disorders.<br>· Cardiovascular diseases.<br>· Prognostication and efficacy assessment in cancer.
**Proteomics**	Low	Medium	· Reflect protein levels and modifications directly.<br>· Reveal protein-protein interaction networks.<br>· Explore changes in protein function.	· Complex data analysis with low standardization.<br>· Effects of PTMs.	· Development of tumor biomarkers.<br>· Mechanistic studies in autoimmune diseases.<br>· Pathology of neurodegenerative diseases.
**Metabolomics**	Medium	Medium	· Provide the overall metabolic profile of the organism.<br>· Reflect metabolic changes associated with disease.<br>· Assist in early diagnosis and monitoring.	· Sensitive sample handling and storage conditions.<br>· Challenges in the detection of metabolites.	· Monitoring of endocrine disorders.<br>· Prediction of cardiovascular disease risk.<br>· Metabolic testing in diabetes.

Multi-omics technologies integration

**Single-cell Omics**	High	High	· Resolve cellular heterogeneity (tumor subclones).<br>· Identify rare cell types (<0.1% population).<br>· Enable multi-omics integration (ATAC+RNA).	· Spatial information loss from tissue dissociation<br>· Significant technical noise (dropout rate >15%)<br>· Single-cell amplification bias	· Tumor evolutionary tree construction.<br>· T-cell receptor clonal tracking.<br>· Nervous diseases neuronal subtyping.
**Spatial Omics**	High	High	· Preserve in-situ spatial topology.<br>· Quantify cell-cell interactions (immune synapses).<br>· Directly correlate pathological morphology with molecular expression.	· Resolution inversely proportional to throughput (e.g., MERFISH: ~1,000 genes).<br>· Optical diffraction limitations (>200nm).<br>· High complexity in multidimensional data integration.	· Tumor immune exclusion zone mapping.<br>· Brain region-specific protein gradient atlases.<br>· Myocardial infarction spatial injury demarcation.

PTMs: post-translational modifications.

Table 2: Comparative Analysis of Five Mainstream Proteomics Techniques.

Technology	Introduction	Advantages	Disadvantages	Labeling Groups	Data Volume	Cost
**iTRAQ**	Employ chemical labels to identify proteins.	· Simultaneous analysis of up to 8 groups.<br>· Processing of multiple samples.<br>· Enhanced throughput.	· Expensive reagents.<br>· Complex experimental procedures.	4 or 8	Medium	High
**TMT**	Utilize chemical labeling to identify proteins.	· Simultaneous analysis of up to 10 or 11 groups.<br>· Simultaneous processing of more samples.<br>· Higher sensitivity.	· Expensive reagents.<br>· Complex experimental operations.	10 or 11	Medium	High
**SILAC**	Introduce isotope-labeled amino acids into the culture media.	· Accurate quantification.<br>· High sensitivity.	· Requirement for cell culture.<br>· Unsuitability for clinical samples.	2 or 3	Low	Medium
**Label-free**	Detect endogenous peptides without labeling.	· No need for labeling.<br>· Simple sample preparation.<br>· Cost-effective.	· Reduced reproducibility.<br>· Slightly diminished sensitivity.	Unlimited	High	Low
**DIA/SWATH**	Obtain mass spectrometry data through a full scan.	· No labeling required.<br>· Simultaneous quantification of numerous proteins.<br>· Good reproducibility.	· Complex data analysis.<br>· Need for specialized software.	Unlimited	High	Medium

iTRAQ: isobaric tags for relative and absolute quantification; TMT: tandem mass tag technology; SILAC: stable-isotope labeling by amino acids in cell culture; DIA: data-independent acquisition; SWATH: sequential window acquisition of all theoretical mass spectral approach.

Multi-omics technologies integration

Supplementary Table S1: Application of Multi-omics and High-spatial-resolution Omics Technologies in AD.

Disease	Omics Type	Purpose	Biomarker	Relevance to AD Pathogenesis	Reference
**AD**	Genomics	Diagnosis	APOE4	· APOE4 accelerates vascular dysfunction, BBB rupture, and neuronal degeneration.<br>· APOE4 is pivotal in AD's vascular and neurodegenerative pathogenesis and serves as a marker.	Montagne A et al.[61]
**AD**	Genomics	Diagnosis	H4K16ac	· Compared to non-AD elderly participants, 25,000 peaks showed H4K16ac loss, while 9,000 showed increased H4K16ac in AD individuals.<br>· H4K16ac decreases with aging or AD-related gene sites.<br>· H4K16ac set the stage for an epigenetic link between aging and AD.<br>· H4K16ac can be a diagnostic marker for AD disease.	Nativio R et al.[62]
**AD**	Genomics	Diagnosis	H3K9ac,<br>Tau protein	· Tau, whereas non-amyloid β pathology has a broad impact on histone acetylation in AD brain.<br>· H3K9ac structural domain shows similar gain or loss of tau-related histone acetylation.<br>· Complex interactions between tau and chromatin structure.<br>· H3K9ac and Tau are biomarkers for AD.	Klein HU et al.[63]
**AD**	Transcriptomics	Diagnosis	INPPL1, PLXNB1	· The M109 module is the one most directly associated with cognitive decline and amyloid load.<br>· INPPL1 and PLXNB1 are associated with extracellular β-amyloid levels in astrocyte cultures.<br>· INPPL1 and PLXNB1 are interesting candidates for AD patients.	Mostafavi S et al.[64]

Multi-omics technologies integration

**AD**	Proteomics	Treatment	STAT3, YES1 and FYN	· STAT3, YES1, and FYN reduce neuroinflammation, tau phosphorylation, and endogenous production of amyloid-42.<br>· Drugs targeting the cytokine transducer STAT3 and the Src family tyrosine kinases, YES1 and FYN, rescued molecular phenotypes relevant to AD pathogenesis.<br>· STAT3, YES1, and FYN can be used as drug targets for the treatment.	Jackson A et al.[65]
**AD**	Proteomics	Treatment	Insulin signaling and mitochondrial electron transport chain	· Changes in hippocampal protein expression profiles in APP/PS1 and E4 knockout mice.<br>· Different expression proteins in both mouse models, participate in insulin signaling and the mitochondrial electron transport chain.<br>· Preserving mitochondrial function and boosting insulin signaling could aid in improving cognitive function for AD patients.	He K et al.[66]
**AD**	Proteomics	Diagnosis /Treatment	The phosphorylation levels of GSK3β and Ppp3caGSK3β, Ppp3ca	· The phosphorylation levels of GSK3β and Ppp3ca are closely associated with mitochondrial biogenesis.<br>· Low-dose oral copper treatment changes the phosphorylation of key hippocampal proteins involved in mitochondrial, synaptic and axonal integrity.<br>· The phosphorylation levels of GSK3β and Ppp3ca are potential diagnostic and therapeutic targets.	Chen C et al.[67]
**AD** (autosomal dominant)	Proteomics	Diagnosis	GFAP, NPTX2, PEA15, SMOC1, SMOC2, TNFRSF1B	· Six-protein prediction model (GFAP, NPTX2, PEA15, SMOC1, SMOC2, TNFRSF1B) with excellent predictive performance (AUC>0.9)<br>· Six early biomarkers far exceed the warning time window of traditional markers	Shen Y et al.[68]

**AD**	Metabolomics	Diagnosis	Sphingolipids	· Identified sphingolipids map to AD-related pathways (tau phosphorylation, amyloid metabolism, calcium homeostasis, acetylcholine biosynthesis, apoptosis AD is associated with dysregulation of transmethylation and polyamine pathways.<br>· Sphingolipids as early AD biomarkers.	Varma VR et al.[69]
**AD**	Metabolomics	Diagnosis	PKM2	· PKM is an important glycolytic enzyme associated with AD pathology.<br>· Induced neurons iNs from AD patients express cancer-associated PKM2.<br>· PKM2 promotes Warburg effect-like glycolytic reprogramming in old neurons.<br>· PKM2 specifically interacts with and enhances the transcription factors STAT3 and HIF1α, promoting AD-induced neurons' neuronal fate loss.<br>· PKM2 is a potential diagnostic target for AD.	Traxler L et al.[70]
**AD**	ScRNA-seq	Diagnosis	Myelination-related gene (LINGO1)	· Myelination plays a pivotal role in the pathophysiology of AD.<br>· The myelination-related gene LINGO1 is perturbed in neurons and glial cells in patients with AD.	Mathys H et al.[72]
**AD**	SnRNA-seq	Diagnosis	APOE	· The transcription factor EB serves as a principal regulator of lysosomal function, modulating multiple disease-associated genes in specific astrocyte subpopulations in AD.<br>· The risk gene APOE exhibits upregulated expression in specific microglia and astrocytes in AD, correlating with the severity of Tau pathology.<br>· APOE as a risk gene for AD has been confirmed.	Grubman A et al.[73]

Multi-omics technologies integration

**AD**	Spatial transcriptomics	Diagnosis	OLIG	· Early plaque-proximal dysregulation of OLIG/myelination gene co-expression networks in AD.<br>· Multicellular 57-PIG networks emerge, enriched for complement activation, oxidative stress, lysosomal dysfunction, and neuroinflammatory pathways.<br>· OLIG can be used as a marker for future AD diagnosis to lay the foundation for AD diagnosis.	Wei-Ting Chen et al.[74]
**AD**	Spatial transcriptomics	Diagnosis	SPARC, CALB2, DIRAS2, and KRT17	· 10× Visium + co-immunofluorescence of AD markers delineated gene expression architecture in human middle temporal gyrus.<br>· Cortex-specific layer-enriched DEGs, including novel candidates SPARC, CALB2, DIRAS2, and KRT17, exhibiting pronounced alterations.<br>· These genes demonstrate significant potential as diagnostic targets for AD.	Shuo Chen et al.[75]
**AD**	ScRNA-seq, Spatial transcriptomics	Treatment	Inhibitor of PTPRG or VIRMA	· PTPRG+ microglia subpopulation induces neuronal VIRMA via intercellular signaling.<br>· Neuronal PTPRG binding to VIRMA enhances RNA stability; upregulated VIRMA increases PRKN m6A, reduces its RNA stability, causing mitophagy-driven neuronal death and AD progression.<br>· PTPRG/VIRMA inhibitors show their impacts on mitochondrial function and neuronal survival, offering potential therapies for AD.	Donghua Zou et al.[76]
**AD**	Proteomics, Transcriptomics	Diagnosis	MAPK/metabolic module, matrix body module	· AD-related modules include MAPK signaling/metabolism and matrixsome modules.<br>· Matrixsome module is affected by APOE ε4 allele.<br>· MAPK/metabolism module links to cognitive decline rate.<br>· Disease modules are potential AD targets/biomarkers.	ECB et al.[78]

Multi-omics technologies integration

**AD**	Proteomics, Transcriptomics	Diagnosis	FBP1, FBP2, RHOH, JPH2, ERAp2, and SCLT1, MBP	· FBP1, FBP2, RHOH, JPH2, ERAp2, and SCLT1 are upregulated in APOE4 cases compared to average expression in the normal brain.<br>· MBP is one of the top candidate genes enhancing the relevance of myelination in AD.<br>· Biomarkers show consistent protein profiles in plasma and brain.	Madrid L et al.[79]
**AD**	Genomics, Transcriptomics, Proteomics, Metabolomics	Diagnosis	ABCA1, CPT1A, Adiponectin and NGAL	· Short-chain acylcarnitines/amino acids and medium/long-chain acylcarnitines are closely correlated with the severity of AD.<br>· Two genes (ABCA1 and CPT1A) and two proteins (Adiponectin and NGAL) participate in the regulation of acylcarnitines and amino acids in AD.<br>· ABCA1, CPT1A, Adiponectin, and NGAL may be AD diagnostic biomarkers.	Horgusluoglu E et al.[80]
**AD**	Proteomics, Transcriptomics	Diagnosis	IVD, CYFIP1 and ADD2	· Significantly higher IVD protein abundance in AD patients.<br>· CYFIP1 and ADD2 are significantly downregulated in AD patients.<br>· IVD, CYF0IP1, and ADD2 combine to diagnose AD.	San Segundo et al.[81]
**AD**	Genomics, Metabolomics	Diagnosis	CSTD, CTSB, CTSD, and GM2A	· CSTD has been validated as a marker in previous CSF and plasma samples.<br>· AD progression is clearly accompanied by increased fold changes in these lysosomal proteins.<br>· Lysosomal proteins CTSB, CTSD, and GM2A are significantly increased as markers in CSF samples from AD patients.	Wang H et al.[82]

Multi-omics technologies integration

**AD**	Genomics, Proteomics	Diagnosis	PBXIP1	· PBXIP1-encoded protein shows significant association with all three AD neuropathological features.<br>· PBXIP1 is associated with AD through its role in astrocytes and hippocampal neurons and the mTOR pathway.<br>· PBXIP1 is associated with neuropathology and cognitive function.	Jingyun Zhang et al.[83]
**AD**	Genomics, Transcriptomics, Proteomics	Diagnosis	H3K27ac	· Differentially acetylated peaks are enriched in disease-related biological pathways, including those associated with amyloid-β and tau pathology progression.<br>· Highly significant enrichment of AD risk variants in the H3K27ac peak region of the inner olfactory cortex, including CR1, GPR22, KMO, PIM3, PSEN1, and RGCC.<br>· H3K27ac can serve as a diagnostic target for AD.	Marzi SJ et al.[84]
**AD**	Transcriptomics, Proteomics, Epigenomics	Diagnosis	H3K27ac, H3K9ac	· RNA-seq analysis reveals upregulation of histone acetyltransferases in H3K27ac and H3K9ac.<br>· Genome-wide increases in H3K27ac and H3K9ac exacerbate amyloid-β42-driven neurodegeneration.<br>· Proteomic screening singles out H3K27ac and H3K9ac as major AD-specific enrichments.<br>· H3K27ac and H3K9ac affect disease pathways through dysregulated transcription and chromatin gene feedback loops.	Raffaella Nativio et al.[85]
**AD**	Proteomics, Metabolomics, Lipidomics	Diagnosis	Protein 14-3-3 zeta/delta, clusterin, interleukin-15, and transgelin-2	· Enrichment pathway analysis reveals overexpression of hemostatic, immune response, and extracellular matrix signaling pathways associated with AD.<br>· Protein 14-3-3 zeta/delta, clusterin, interleukin-15, and transgelin-2 improve AD prediction.	Clark C et al.[86]

Multi-omics technologies integration

**AD**	Proteomics, Metabolomics, Lipidomics	Diagnosis	GABA synthesis, arginine biosynthesis, and alanine, aspartate, glutamate, and arginine pathways	· Gender-dependent effects are seen on the pathways of significant enrichment, including those of GABA synthesis, arginine biosynthesis, and alanine, aspartate, glutamate, and arginine metabolism.<br>· Lysophospholipid and amino acid metabolism are involved in the AD brain.	Abigail Strefeler et al.[87]
**AD**	Genomics, Transcriptomics	Treatment	TRPV1	· TRPV1 activation rescues memory deficits and neuronal loss in ApoE4 high-fat diet-fed mice.<br>· Neuronal loss increases in ApoE4 high-fat diet mice, rescued by TRPV1 activation in the capsaicin group.<br>· TRPV1 is a treatment option for AD disease.	Chenfei Wang et al.[88]
**AD** (late-onset)	Genomics, Transcriptomics	Treatment	ATP6V1A	· ATP6V1A has been identified as a key regulator of the top neuronal subnetwork, which is the most dysregulated in late-onset AD.<br>· ATP6V1A can be used as a therapeutic target.<br>· NCH-51 ameliorates neuronal damage caused by ATP6V1A deficiency in a drosophila model.	Wang M et al.[89]

AlzGPS: Alzheimer's disease genome-wide positioning systems platform; CSF: cerebrospinal fluid; PKM2: pyruvate kinase M2; MBP: Myeloid basic protein encoding gene.

Multi-omics technologies integration

Supplementary Table S2: Application of Multi-omics and High-spatial-resolution Omics Technologies in PD.

Disease	Omics Type	Purpose	Biomarker	Relevance to PD Pathogenesis	Reference
**PD**	Genomics	Diagnosis	ZNF184, IL1R2、LRRK2, ITPKB, and PARK16	· Alleles of LRRK2 and IL1R2 confer a higher risk of developing PD.<br>· The genotype models of ZNF184, PARK16, and ITPKB are significantly associated with PD.<br>· Most of these genes are involved in autophagy and lysosomal function-related pathways.	Gao T et al.[96]
**PD**	Genomics	Diagnosis	HLA, LRRK2, MAPT, TRIM10, and SETD1A	· HLA, LRRK2, MAPT, TRIM10, and SETD1A are high-risk genes associated with PD.<br>· Significantly associated loci linked to PD are found in the HLA and MAPT gene loci.	Witoelar A et al.[97]
**PD**	Transcriptomics	Diagnosis	SSR1	· SSR1 is found to be upregulated in PD patients.<br>· SSR1 expression is negatively correlated with dopaminergic neuron survival.<br>· The upregulation of SSR1 expression in peripheral blood precedes the abnormal behavior of the animals.<br>· The SSR1-based RF classifier has an AUC value of 0.91 and can be used as a diagnostic marker.	Zhang W et al.[98]
**PD**	Proteomics	Diagnosis	OMD, CD44、VGF, PRL, MAN2B1, and LRRK2	· ML identifies that OMD, CD44, VGF, PRL, and MAN2B1 show significant changes in PD patients and are significantly correlated with PD clinical scores.<br>· The enhanced neuroinflammatory characteristics in LRRK2 gene carriers are strongly correlated with PD.<br>· OMD, CD44, VGF, PRL, MAN2B1, and LRRK2 can be used as biomarkers for PD.	Karayel, Matthias Man et al.[99]

Multi-omics technologies integration

**PD**	Metabolomics	Diagnosis	Lipid metabolism related to carnitine shuttle, sphingolipid metabolism, and arachidonic acid metabolism	· Alterations in lipid metabolism related to carnitine shuttle, sphingolipid metabolism, arachidonic acid metabolism, and fatty acid biosynthesis are detected.<br>· Carnitine shuttling is the most important pathway associated with unmedicated PD patients by sebum.	Sinclair E et al.[100]
**PD**	Metabolomics	Diagnosis	Short-chain fatty acids, butyric acid	· Low levels of short-chain fatty acids are significantly associated with cognitive decline in PD patients.<br>· Decreased butyric acid levels are associated with poorer posture and gait disorder scores.<br>· Short-chain fatty acids and butyric acid serve as a potential diagnostic target.	Tan AH et al.[101]
**PD**	Metabolomics	Diagnosis	Proline	· Energy and lipid metabolism are overexpressed in PD.<br>· 139 metabolites, including proline, have notable changes in carnitine shuttle, vitamin E metabolism, lipid-related, glycerol phospholipids, sphingolipids, and fatty acids pathways.<br>· Proline and 139 other metabolites are considered particularly predictive of PD status.	Pereira, P.A.B et al.[102]
**PD**	Metabolomics	Diagnosis	Phenylacetic acid, phenylacetylglutamine, histidine, uric acid, and imidazoleacetic acid	· 45 metabolic markers in PD patients show high diagnostic power in early stages (AUC=0.92).<br>· Metabolites like phenylacetic acid, phenylacetylglutamine, histidine, uric acid, and imidazoleacetic acid show upregulated urine levels in PD, linked to neuro disorders.	Cai Z et al.[103]

**PD**	Metabolomics	Diagnosis	BCAA metabolism, glycine derivatives, steroid hormone biosynthesis, tryptophan, and phenylalanine metabolism	· 18 differential metabolites in urine have been identified as biomarkers for PD.<br>· Differential metabolites alter metabolic pathways associated with BCAA metabolism, glycine derivatives, steroid hormone biosynthesis, tryptophan metabolism, and phenylalanine metabolism.	Cai Z et al.[104]
**PD**	SnRNA-seq	Diagnosis	IL1B, GPNMB, and HSP90AA1	· A neuron cluster characterized by CADPS2 overexpression and low tyrosine hydroxylase levels is identified in PD.<br>· Astrocytes and microglia in PD show specific proliferation and gene dysregulation linked to unfolded protein response and cytokine signaling.<br>· Microglia show a pro-inflammatory state with high IL1B, GPNMB, and HSP90AA1, suggesting their diagnostic potential.	Semra Smajić et al.[197]
**PD**	ScRNA-seq	Treatment	HSP90 inhibitors	· Neurons and glial cells in PD exhibit dysfunction, immune dysregulation, and impaired protein folding.<br>· Administration of HSP90 inhibitors accelerates the degradation of inflammasomes, reducing inflammatory responses and alleviating neurodegeneration.	Gabriel GE et al.[105]

Multi-omics technologies integration

**PD**	Single-cell genomics, Spatial transcriptomics	Diagnosis	TP53, NR2F2	· The AGTR1-marked SNpc ventral subtype is highly PD-susceptible, showing TP53/NR2F2 target gene upregulation.<br>· TP53/NR2F2-regulated pathways are key to PD-related neuronal death.<br>· TP53/NR2F2 target gene upregulation indicates diagnostic biomarker potential.	Tushar Kamath et al.[106]
**PD**	ScRNA-seq, Proteomics	Diagnosis	SYN2	· Negative correlation between α-synuclein pathology and chaperone protein expression in excitatory neurons in PD, along with weakened neuron-astrocyte interaction and aggravated neuroinflammation.<br>· SYN2 enrichment in PD brain regions suggests significant increase in synaptic signaling at both RNA and protein levels.<br>· SYN2 as a potential diagnostic biomarker for PD	Biqing Zhu et al.[107]
**PD**	Proteomics, Transcriptomics	Diagnosis	GPNMB, CD38, and DGKQ	· GPNMB and CD38 show significant causal effects in PD, with evidence from quantitative trait locus analysis and fine mapping.<br>· GPNMB, CD38, and DGKQ proteins are associated with PD risk.	Guxiaojing et al.[108]
**PD**	Three Proteomics	Diagnosis	DDC	· DDC, SUMF1, DPP7, ENPEP, WFDC2, and hundreds of proteins are upregulated in the CSF, blood, or urine of PD patients.<br>· DDC levels are linked to symptom severity in PD patients.<br>· DDC can serve as a target for accurate PD diagnosis.	Rutledge J et al.[109]
**PD**	Transcriptomics, Metabolomics	Treatment	The relaxin signaling pathway, adhesion patch, and PI3K-Akt signaling pathway	· BHD reduces PD symptoms, impacting metabolic pathways, including the relaxin signaling pathway, adhesion patch, and PI3K-Akt signaling pathway.<br>· BHD promotes the survival of dopaminergic neurons in PD mice, leading to improved motor performance.	Hujun et al.[110]

Multi-omics technologies integration

**PD**	Genomics, Metabolomics	Diagnosis/ Treatment	CircSV2b	· Detect 33 deregulated circular RNAs in the PD mouse model vs wild-type controls.<br>· CircSV2b overexpression via the ceRNA-Akt1 axis mitigates oxidative stress in PD.<br>· CircSV2b is a potential Parkinson's diagnostic and curative biomarker.	Cheng Qi et al.[111]

BCAA: branched chain amino acid; SNpc: substantia nigra pars compacta; DDC: Dopamine decarboxylase; ML: machine learning; LRRK2: leucine-rich repeat kinase 2; SUMF1: sulfatase-modifying factor 1; DPP7: dipeptidyl peptidase 2/7; SSR1: signal sequence receptor subunit 1; ENPEP: glutamyl aminopeptidase; WFDC2: WAP four-disulfide core domain 2; BHD: Buyang Huanwu Decoction.

Multi-omics technologies integration

Supplementary Table S3: Application of Multi-omics and High-spatial-resolution Omics Technologies in Epilepsy.

Disease	Omics Type	Purpose	Biomarker	Relevance to Epilepsy Pathogenesis	Reference
**Epilepsy**	Transcriptomics	Diagnosis	p38MAPK, Jak-STAT, PI3K, and mTOR signal pathway	· The p38MAPK, Jak-STAT, and PI3K consistently exhibit high expression and along with stable regulation of mTOR signaling pathways in epilepsy patients.<br>· Differential genes engage in signal cascades, ECM remodeling, cell motility, apoptosis, and immune responses linked to seizures.	Oswaldo K Okamoto et al.[115]
**Epilepsy** (temporal lobe)	Transcriptomics	Diagnosis	Tlr2, Lgals3, Serpine 1 and Stat3 et al.	· Several hub genes identified in TLE, such as Tlr2, Lgals3, Serpine1, and Stat3, et al., positively correlate with seizure frequency.<br>· Activation and phagocytic activity of microglia/macrophages have changed during the epileptic occurrence process of TLE.<br>· Tlr2, Lgals3, Serpine 1, and Stat3 can serve as markers for TLE.	QingLan Chen et al.[116]
**Epilepsy**	Proteomics	Diagnosis	GFAP	· GFAP is consistently downregulated in brain tissue with high spike frequencies and exhibits a strong negative correlation with spike frequency.<br>· Reactive astrocytes, such as GFAP, protect the neocortex from epileptic discharges rather than induce them.<br>· Epilepsy severity is closely linked to decreased GFAP (astrocyte marker) levels.	Gal Keren-Aviram et al.[117]

Multi-omics technologies integration

**Epilepsy**	Proteomics	Diagnosis/ Treatment	ADPRC, LPAR3, calreticulin, UCH-L1, SNAP-25, and transgelin-3	· A total of 144 differentially expressed proteins, such as ADPRC, LPAR3, calreticulin, UCH-L1, SNAP-25, and transgelin-3, are identified in the epileptic hippocampal regions.<br>· Most differentially expressed proteins are associated with Ca2+ homeostasis.<br>· Inhibiting calcium influx alleviates seizures triggered by excessive brain Ca^2+ rise in epilepsy.	Leila Sadeghi et al.[118]
**Epilepsy**	Proteomics	Diagnosis	Calcineurin	· Tutin induces epilepsy by activating calcium-modulating phosphatase and produces significant neurological damage.<br>· Calcineurin is a target of tutin, and that tutin activates Calcineurin, leading to seizures.	Shi-Shan Yu et al.[119]
**Epilepsy**	Metabolomics	Diagnosis	N-acetyl glycoprotein, lactate, creatine, glycine, lipid, and citrate	· Serum N-acetyl glycoprotein, lactate, creatine, glycine, and lipid levels are elevated decreased levels of citrate in epileptic children, while the level of citrate is reduced.<br>· The aforementioned metabolic substances are potential diagnostic targets for epilepsy.	Łukasz Boguszewicz et al.[120]
**Epilepsy** (mesial temporal lobe)	Metabolomics	Diagnosis	GABA	· GABA is significantly increased in the epileptogenic zone of KA-MTLE mice.<br>· GABA is a specific biomarker of the epileptogenic zone in MTLE.	Hamelin, S et al.[121]
**Epilepsy**	SnRNA-seq	Diagnosis	Sst and Pvalb	· Major transcriptomic alterations occur in principal neurons (L5-6_Fezf2, L2-3_Cux2) and GABAergic interneurons (Sst, Pvalb).<br>· Profound dysregulation in glutamate signaling, characterized by robust upregulation of glutamate receptor genes, notably within Sst/Pvalb subtypes.<br>· Sst/Pvalb interneurons represent potential diagnostic targets and are fundamental to early epileptogenesis.	Ulrich Pfisterer et al.[122]

Multi-omics technologies integration

**Epilepsy** (post-traumatic)	ScRNA-seq	Diagnosis	XIST	· Hereditary epilepsy shows higher oligodendrocyte/astrocyte counts, lower microglia/neuron counts vs PTE.<br>· IL-17 signaling in microglia/astrocytes can be a PTE target/biomarker.<br>· XIST, upregulated in PTE, drives inflammation/fibrosis, useful for diagnosis and mechanism study.	Fang Wen et al.[123]
**Epilepsy** (temporal lobe)	ScRNA-seq, SnRNA-seq, Spatial transcriptomics	Diagnosis	Spp1, Trem2, Tle4 and Sipa1l3	· The differentially up-regulated genes in TLE patients are predominantly expressed in glial cells, while the down-regulated genes are mainly expressed in neurons.<br>· Spp1 and Trem2 are up-regulated in glial cells, whereas Tle4 and Sipa1l3 are down-regulated in these cells.	Quanlei Liu et al.[124]
**Epilepsy**	Genomics, Transcriptomics	Diagnosis	Sestrin 3	· Sestrin 3 is a key regulator in the pro-convulsant gene network in the hippocampus of human epilepsy.<br>· Sestrin 3 positively regulates modules in macrophages, microglia, and neurons.<br>· Sestrin 3 holds potential as a diagnostic means for epilepsy.	Johnson, M.R. et al.[125]
**Epilepsy**	Proteomics, Transcriptomics	Diagnosis	STAT3, ErbB, and Mapk8	· The TGF-β pathway is associated with cardiac function in the hearts of epileptic animals.<br>· STAT3, ErbB, and Mapk8 are key regulators of cardiac alterations in epilepsy that contribute to seizure-mediated cardiac damage.	Sharma, S et al.[126]
**Epilepsy**	Proteomics, Metabolomics	Diagnosis	GSTM1, ALDH2	· Within the somatosensory cortex module, GSTM1 is identified as a protein hub and elevated expression levels.<br>· In the thalamus module, ALDH2 is pinpointed as a protein hub.<br>· The metabolic pathway enriched by the differences is lysine degradation.<br>· GSTM1 and ALDH2 are identified as markers for seizure-related modules in epilepsy.	Harutyunyan, A et al.[127]

Multi-omics technologies integration

**Epilepsy**	Genomics, Metabolomics	Diagnosis	Lactate, creatine, phosphocreatine, and choline	· Lactate is significantly reduced, while creatine, phosphocreatine, and choline are significantly increased.<br>· Lactate is involved in G protein-coupled receptor signaling and angiogenic pathways, and shows upregulation of ubiquitination-related genes.	Wu, H.C. et al.[128]
**Epilepsy**	Proteomics, Transcriptomics	Treatment	miR-10a-5p, miR-21a-5p and miR-142a-5p	· miR-10a-5p, miR-21a-5p, and miR-142a-5p are identified as key transcripts.<br>· These microRNA transcripts are primarily associated with the TGF-β pathway signaling.<br>· The combination of anti-miR (miR-10a-5p, miR-21a-5p, miR-142a-5p) exhibits protective effects against acute and spontaneous seizures.	Venø, M.T et al.[129]

PTE: post-traumatic epilepsy; TLE: temporal lobe epilepsy; MTLE: mesial temporal lobe epilepsy; NMDA: N-methyl-D-aspartate; GABA: γ-aminobutyric acid; GFAP: glial fibrillary acidic protein; KA-MTLE: kainic acid into mesiotemporal lobe epilepsy mice; TGF-β, transforming growth factor β; ADPRC: ADP-ribosyl cyclase; LRRK2: leucine-rich repeat kinase 2; LPAR3: lysophosphatidic acid receptor 3; UCH-L1: ubiquitin carboxyl-terminal hydrolase L1; GSTM1: glutathione s-transferase M1; SNAP-25: synaptosome-associated protein 25.

Multi-omics technologies integration

Supplementary Table S4: Application of Multi-omics and High-spatial-resolution Omics Technologies in MS.

Disease	Omics Type	Purpose	Biomarker	Relevance to MS Pathogenesis	Reference
**MS**	Proteomics	Diagnosis	CXCL13, LTA, FCN2, ICAM3, LY9, SLAMF7, TYMP, CHI3L1, FYB1, TNFRSF1B, and NFL	· Lower levels of NFL in CSF show predictive potential for disease activity (AUC=0.77).<br>· An 11-protein panel in CSF has a high AUC for prediction, including CXCL13, LTA, FCN2, ICAM3, LY9, SLAMF7, TYMP, CHI3L1, FYB1, TNFRSF1B, and NFL (AUC=0.9).<br>· All the above proteins can be markers for MS.	Mika Gustafsson et al.[136]
**MS**	Metabolomics	Diagnosis	DRD2	· DRD2 exacerbates the disease by promoting inflammation and reducing the abundance of Lactobacillus species in the microbiome.<br>· Lactobacillus-derived N2-acetyl-L-lysine inhibits microglial activation, combating neurodegeneration.<br>· Intestinal epithelial DRD2, serving as a biomarker, can modulate the gut microbiome in MS.	Hairong Peng et al.[137]
**MS** (relapsing-remitting)	Metabolomics	Diagnosis/ Treatment	Glycolysis	· Identified four perturbed metabolic pathways, including structural/signaling lipids and energy, in the serum of patients with MS.<br>· Glycolysis is the common upstream feeding of these altered metabolic pathways.<br>· Targeting glycolysis in experimental autoimmune encephalomyelitis ameliorated the disease pathology by impeding immune cell effector function.	Insha Zahoor et al.[138]

Multi-omics technologies integration

**MS**	ScRNA-seq	Diagnosis	TFH	· Myeloid dendritic cells and regulatory T cells are enriched in the CSF of patients with MS.<br>· The independent increase in clusters of TFH cells drives the known expansion of B-lineage cells in the CSF in MS.<br>· TFH cells promote the infiltration of B cells into the central nervous system, exacerbating MS disease.	David Schafflick et al.[139]
**MS**	ScRNA-seq, Spatial transcriptomics	Diagnosis	SERPINA3	· Astrocytes can be classified into three types: homeostatic, intermediate, and disease-associated types.<br>· In patients with DA-Astro, the expression level of SERPINA3 is significantly elevated.<br>· SERPINA3 expression may constitute a glial cell survival response to resolve inflammation and prevent apoptosis during both initial and late resolution phases.	Petra Kukanja et al.[140]
**MS**	ScRNA-seq, Spatial transcriptomics	Diagnosis	MAFB	· The expression of pro-inflammatory molecules in oligodendrocytes near axonal damage is elevated in MS patients.<br>· MAFB mediates intercellular communication via complement factors and apolipoproteins.<br>· The inflammatory transcription factor MAFB serves as a biomarker for MS lesions.	Maria L Elkjaer et al.[141]
**MS (systemic)**	ScRNA-seq, Spatial transcriptomics, Spatial proteomics	Diagnosis/ Treatment	POSTN/SCARA5, CXCR4	· A dynamic spatial interaction network is established between fibroblasts and macrophages via the ACKR3-CXCL12-CXCR4 signaling axis, playing a central role in driving fibrosis progression.<br>· Treatment with the CXCR4 inhibitor AMD3100 significantly alleviates fibrosis in skin and lung tissues.<br>· The significantly elevated POSTN/SCARA5 ratio in MS can serve as a predictive diagnostic biomarker.	Zhijian Li et al.[142]

Multi-omics technologies integration

**MS**	Proteomics, Transcriptomics	Diagnosis	GPR37L1, SIRPA, FGFR3, CADM3, and TYRO3	· Neurological candidate molecules, including GPR37L1, SIRPA, FGFR3, CADM3, and TYRO3, are highly expressed in the CNS of MS.<br>· These genes are associated with early neuronal degeneration and dysfunctional trophic/anti-inflammatory intercellular communication.<br>· GPR37L1, SIRPA, FGFR3, CADM3, and TYRO3 can be used as a diagnostic method for MS.	Max Kaufmann et al.[143]
**MS**	Proteomics, Transcriptomics	Diagnosis	24 iron death-related genes (CHMP5, SLC38A1, PML, etc.)	· High iron death scores at the margins of active lesions correlate with phagocytic activation.<br>· Elevated iron death scores in cortical neurons are associated with neurological diseases.<br>· A blood-based model of 24 iron death-related genes is a prognostic marker for diagnosing MS, including CHMP5, SLC38A1, PML, etc.	Tao Wu et al[144]
**MS**	Proteomics, Metabolomics	Diagnosis	LAMP1, FCG2A, and HPSE	· HPSE is positively correlated with many MS-related metabolites, including L-tyrosine, sphingosine 1-phosphate, sphingosine 1-phosphate, and L-tryptophan.<br>· The proteins LAMP1, FCG2A, and HPSE exhibit potential utility as specific biomarkers for MS.	Fan Yang et al.[145]
**MS**	Proteomics, Metabolomics	Diagnosis	Equine uric acid, sphingolipids	· Anti-inflammatory molecules and sphingolipids are reduced by metabolomics in MS patients.<br>· Low levels of equine uric acid in a severe subgroup of MS.<br>· Sphingolipids and equine uric acid facilitate the future development of biomarkers and targeted therapeutic interventions for MS.	Qinming Zhou et al.[146]

NFL: neurofilament light chain; DRD2: Dopamine Receptor D2; CNS: central nervous system; HPSE: heparinase; TFH: T follicular helper.

Multi-omics technologies integration

Supplementary Table S5: Application of Multi-omics and High-spatial-resolution Omics Technologies in Stroke.

Disease	Omics Type	Purpose	Biomarker	Relevance to Stroke Pathogenesis	Reference
**Stroke** (ischemic)	Proteomics	Diagnosis	NSF, RhoGDI1, and RabGDI	· Circulating NSF, RhoGDI1, and RabGDI are upregulated in patients with IS.<br>· These proteins trigger neuronal depolarization and calcium surge, activating death pathways in stroke.	Eloy Cuadrado et al.[149]
**Stroke** (ischemic)	Proteomics	Diagnosis	CMPK, CKB	· Circulating levels of CKB and CMPK are higher in patients with ischemic stroke than in controls during the acute phase.<br>· CKB plays a crucial role in energy transduction and homeostasis.<br>· CMPK is released in large amounts and participates in mechanisms that counteract cell disruption and neuronal cell death.	Alba Simats et al.[150]
**Stroke** (ischemic)	Proteomics	Diagnosis	SAHH2	· SAHH2 plays a significant role in the coordinated inhibition of Ca^{2+} ion transporters.<br>· Increased expression of SAHH2 in neurons from the infarcted area is probably because of ischemia-triggered Ca2+ mobilization.	Teresa García -Berrocoso et al.[151]
**Stroke**	Transcriptomics	Diagnosis	LncRNA (MEG3, H19, and MALAT1)	· LncRNAs, such as MEG3, H19, and MALAT1, in blood cells between patients with stroke and healthy controls show differences.<br>· Differential genes modulate neuronal survival/apoptosis targets, impacting p53-mediated apoptosis in stroke.	Cheryl Dykstra Aiello et al.[152-156]

**Stroke**	Transcriptomics	Diagnosis	Extracellular microRNA	· Decreased levels of extracellular miR-32-3p, miR-106b-5p, miR-423-5p, miR-451a, miR-1246, miR-1299, miR-3149 and miR-4739, and increased levels of extracellular miR-224-3p, miR-377-5p, miR-518b, miR-532-5p and miR-1913 associate with stroke.<br>· These genes affect multiple pathways such as apoptosis, oxidation, angiogenesis, and neurogenesis in IS.	Ceren Eyileten et al.[157]
**Stroke** (cardioembolic)	Metabolomics	Diagnosis	Valine, Leucine, Isoleucine	· The expression levels of BCAA, including valine, leucine, and isoleucine, are decreased in patients with cardioembolic stroke.<br>· Lower BCAA levels are also associated with poor neurological outcomes.	W Taylor Kimberly et al.[158]
**Stroke**	Metabolomics	Diagnosis	Total free fatty acid	· Plasma concentration of total free fatty acids is higher in patients with cardioembolic stroke than in patients with non-cardioembolic stroke.<br>· Elevated free fatty acid levels are significantly associated with cardioembolic stroke, suggesting their potential as a diagnostic target.	Jeong Yoon Choi et al.[159]
**Stroke**	ScRNA-seq	Treatment	Microglia and macrophages	· Aging jeopardizes the repair and regeneration of the cerebrovascular system and proteins after stroke.<br>· After stroke, microglia and macrophages may affect angiogenesis and oligodendrogenesis via paracrine mechanisms, impeding stroke recovery.<br>· Transplanting microglia and macrophages from the brains of young mice into the cerebral cortex of aged stroke-affected mice partially restores angiogenesis and oligodendrogenesis.<br>· Microglia and macrophages serve as effective targets for promoting stroke recovery	Chenghao Jin et al.[160]

Multi-omics technologies integration

**Stroke** (ischemic)	ScRNA-seq, Spatial transcriptomics	Treatment	LILRB4	· Stroke brains have up-regulated LILRB4 and ischemia-linked microglial cluster 3.<br>· LILRB4 knockout worsens ischemic brain injury via CD8+ T cell recruitment; overexpression offers neuroprotection.<br>· Targeting LILRB4 and its downstream pathways represents an effective therapeutic strategy for ischemic stroke.	Yilin Ma et al.[161]
**Stroke** (brainstem)	SnRNA-seq, ScRNA-seq	Treatment	Myo1e	· Oligodendrocyte loss leads to neurological deficits following brainstem stroke.<br>· OLG8 has an innate neuroprotective effect in brainstem stroke.<br>· Myo1e aids OLG8 migration to the peri-infarct area in brainstem stroke.<br>· Myo1e overexpression in OLG8 oligodendrocytes boosts brainstem stroke recovery.	Shaojun Li et al.[162]
**Stroke** (intracerebral hemorrhage)	ScRNA-seq, Spatial transcriptomics	Diagnosis	Spp1, Lyz2	· Spp1/Lyz2 show high expression levels, and lymphocytes with high expression interact with myeloid cells in the late stage of stroke.<br>· During the acute phase of intracerebral hemorrhage, Lgmn+Macro-T cells and microglia interact via the Spp1-cd44 pathway.<br>· Spp1 and Lyz2 are potential diagnostic targets for the acute phase of intracerebral hemorrhage.	Lingui Gu et al.[163]
**Stroke**	ScRNA-seq, Spatial transcriptomics	Treatment	Lipocalin-2	· Ferroptosis is the primary programmed cell death process post-hemorrhagic stroke, mainly affecting mature oligodendrocytes.<br>· A specific interaction between lipocalin-2-positive microglia and oligodendrocytes, mediated by the CSF1 receptor pathway, induces ferroptosis in oligodendrocytes and subsequent neurological deficits.<br>· Early therapeutic intervention by inhibiting LCN2 expression may alleviate ferroptosis-induced oligodendrocyte damage and related neurological deficits.	Lingui Gu et al[164]

Multi-omics technologies integration

**Stroke**	ScRNA-seq, Spatial transcriptomics	Treatment	LGALS9	· Galectin (LGAL) signaling is enhanced in microglia and macrophages of ischemic mice.<br>· LGALS9 treatment promotes oligodendrocyte remyelination and improves stroke recovery in mice.<br>· LGALS9 can serve as a therapeutic approach to ameliorate stroke.	Bing Han et al.[165]
**Stroke (subarachnoid hemorrhage)**	ScRNA-seq, Spatial transcriptomics	Diagnosis	THBS1, S100A6	· THBS1 and S100A6 are closely associated with the prognosis of SAH, with their expression significantly increasing following the hemorrhage.<br>· The THBS1-CD47 pair regulates cell apoptosis, and blocking their interaction may represent a new therapeutic approach for SAH.<br>· THBS1 and S100A6 serve as diagnostic biomarkers for stroke.	Xiaoyu Wang et al.[166]
**Stroke** (cardioembolic)	Proteomics, Transcriptomics	Diagnosis	ICA1L, CAND2, and ALDH2	· Reduced ICA1L, CAND2, and ALDH2 may impair excitatory synaptic signaling, contributing to cardioembolic stroke pathogenesis.<br>· ICA1L, CAND2, and ALDH2 are potential biomarkers for lacunar stroke.	Zhang C et al.[167]
**Stroke** (ischemic)	Genomics, Metabolomics	Treatment	Gut flora and metabolic disturbances	· ZHTC modulates the abundance of specific bacterial groups and 23 metabolic differences for IS, including arginine, L-lysine, and L-methionine.<br>· ZHTC improves intestinal barrier integrity by increasing the expression levels of tight junction proteins<br>· ZHTC meliorates IS by modulating gut flora and metabolic disturbances.	Wang R et al.[168]

Multi-omics technologies integration

**Stroke** (ischemic)	Proteomics, Transcriptomics	Treatment	PI3K-Akt, MAPK, and cAMP signaling pathways	· YQTL reduces infarct volume percentage and improves neurological function in cerebral ischemia-reperfusion injury mice.<br>· Network pharmacology and multi-omics studies reveal 15 components that regulate 82 targets and 19 pathways.<br>· YQTL protects against cerebral ischemia-reperfusion injury through PI3K-Akt, MAPK, and cAMP signaling pathways.	Yuan Y et al et al.[169]

lncRNAs: long non-coding RNAs; IS: Ischemic Stroke; BCAA: branched-chain amino acid; LGAL: Galectin; CSF1: colony-stimulating factor 1; SAH: subarachnoid hemorrhage; ZHTC: Zhilong Huoxue Tongyu capsule; YQTL: Yiqi Tongluo granule; ALDH2: aldehyde dehydrogenase 2.

Multi-omics technologies integration

Supplementary Table S6: Application of Multi-omics and High-spatial-resolution Omics Technologies in Hydrocephalus.

Disease	Omics Type	Purpose	Biomarker	Relevance to Hydrocephalus Pathogenesis	Reference
**Hydrocephalus** (communicating)	Genomics	Diagnosis	TRIM71, SMARCC1, PIK3CA, PTEN, MTOR, FOXJ1, FMN2, PTCH1, and FXYD2	· TRIM71 and SMARCC1 exhibit genome-wide significant enrichment of de novo mutations, which may be genuine risk factors for CH.<br>· PIK3CA, PTEN, MTOR, FOXJ1, FMN2, PTCH1, and FXYD2 are newly identified high-confidence sporadic CH genes.<br>· TRIM71 and other genes, reducing neural cell proliferation to cause hydrocephalus, can be a diagnostic marker.	Sheng Chih Jin et al.[177]
**Hydrocephalus** (communicating)	Proteomics	Diagnosis	KLK6	· Expression of KLK6 is significantly up-regulated in CH patients.<br>· KLK6 is involved in CH development and may provide a new target for CH diagnosis.	Lei Yuan et al.[178]
**Hydrocephalus** (idiopathic normal pressure)	Proteomics	Diagnosis	QPCT, RBP4	· 39 proteins exhibit a significant increase, while 285 proteins show a significant decrease in CSF of iNPH.<br>· Elevated proteins mainly relate to myeloid leukocyte migration and extracellular matrix organization; reduced ones are linked to axon and synaptic development.<br>· QPCT and RBP4 have been identified as potential protein biomarkers in iNPH for predicting shunt outcomes.	Yuqi Ying et al.[179]

Multi-omics technologies integration

**Hydrocephalus** (idiopathic normal pressure)	Proteomics	Diagnosis	PTPRQ	· PTPRQ concentrations in CSF are significantly higher in iNPH patients than in AD patients.<br>· PTPRQ concentration in the CSF of non-responders to shunt operation tended to be relatively lower compared with that in the responders.<br>· PTPRQ is a candidate biomarker to distinguish iNPH from AD.	Yuki Nagata et al.[180]
**Hydrocephalus** (idiopathic normal pressure**)**	Metabolomics	Diagnosis	Glyceric acid, N-acetyl neuraminic acid, serine, and 2-hydroxybutyric acid	· Elevated glyceric acid and N-acetyl neuraminic acid, and reduced serine and 2-hydroxybutyric acid in AD CSF distinguish it from iNPH.<br>· Serine, glyceric acid, Neu5Ac, and 2-hydroxybutyrate combine as a diagnostic iNPH biomarker.	Yuki Nagata et al.[181]
**Hydrocephalus** (normal pressure)	Metabolomics	Treatment	Neu5Ac	· CSF Neu5Ac levels are low in NPH patients.<br>· Boosting brain Neu5Ac inhibits astrocyte activation.<br>· Brain Neu5Ac elevation reduces periventricular demyelination and improves hydrocephalus.<br>· Enhanced brain Neu5Ac improves neurological outcomes in NPH, suggesting a potential treatment.	Zhangyang Wang et al.[182]
**Hydrocephalus** (tumor-associated)	SnRNA-seq, Spatial transcriptomics	Diagnosis/ Treatment	CPMCs,	· Ventricular cell atlas reveals CPMC expansion in TAH mice.<br>· CPMCs compromise ependymal ciliary integrity via tryptase-PAR2-FoxJ1 signaling, triggering pathological CSF hypersecretion that underlies hydrocephalus pathogenesis.<br>· Brain barrier-penetrating trypsin-like inhibitor BMS-262084 effectively inhibits TAH progression in vivo and attenuates mast cell-induced epithelial cilia damage.	Yiye Li et al.[183]

Multi-omics technologies integration

**Hydrocephalus** (communicating)	Genomics, Proteomics, Transcriptomics	Diagnosis	MAEL	· PrediXcan analysis in 10 neuro tissues and whole blood shows a correlation between reduced MAEL gene expression in the brain and hydrocephalus ($p < 0.05$).<br>· Reduced MAEL expression increases susceptibility to hydrocephalus.<br>· MAEL is a diagnostic biomarker for hydrocephalus.	Andrew T Hale et al.[184]
**Hydrocephalus** (post-hemorrhagic)	Proteomics, Metabolomics	Diagnosis/ Treatment	CSPG4	· CSPG4 positively correlates with ventricular size and the incidence of periventricular leukomalacia.<br>· Silencing of CSPG4 can inhibit ferroptosis, cell adhesion functions, and intracellular Ca2+ flux.<br>· CSPG4 has been identified as a CSF biomarker and effective therapeutic target.	Juncao Chen et al.[185]

NPH: normal pressure hydrocephalus; iNPH: idiopathic normal pressure hydrocephalus; CH: communicating hydrocephalus; Neu5Ac: N-acetylneuraminic acid; TAH: tumor-associated hydrocephalus; CPMCs: choroid plexus mast cells; MAEL: maelstrom spermatogenic transposon silencer; KLK6, kallikrein-6; QPCT: glutaminyl-peptide cyclotransferase; RBP4: retinol-binding protein 4; PTPRQ: Q-type protein tyrosine phosphatase receptor; CSPG4: chondroitin sulfate proteoglycan 4.

Multi-omics technologies integration

Figure Legends

Figure 1: Three main types of metabolomics, including targeted metabolomics, untargeted metabolomics, and widely-targeted metabolomics. This figure outlines these three metabolomics techniques, each with unique strengths and limitations. Careful selection among them can lead to the optimal choice to meet the specific requirements of your experimental objectives.

Figure 2: Research workflow and applications of high-spatial-resolution omics technologies: integrated development of single-cell and spatial omics. This figure illustrates the integrated research workflow of high-spatial-resolution omics technologies, combining single-cell omics technology with spatial omics approaches. Single-cell omics involves: (1) sample preparation; (2) single-cell isolation and labeling (utilizing representative technologies such as Drop-seq and 10x Genomics Chromium); (3) nucleic acid extraction and library construction; and (4) high-throughput sequencing to resolve cellular gene expression profiles and uncover cellular heterogeneity. In parallel, spatial omics employs: (1) tissue sample processing; (2) spatial labeling/capture (via techniques like 10x Visium, Slide-seq, and MERFISH); (3) sequencing/detection; and (4) computational data analysis to map gene expression with spatial coordinates, thereby elucidating tissue microenvironment interactions. The synergy between these approaches addresses complementary questions—who is expressing a gene versus & where expression occurs spatially—enabling breakthroughs in tumor biomarker identification, cellular subpopulation localization, and tissue developmental mechanisms, among others.

Figure 3: Applications of multi-omics and high-spatial-resolution omics technologies in the diagnosis and treatment of brain diseases in the field of neurology. By integrating the high-throughput omics technologies, including four basic omics, single-cell, and spatial omics technologies, it is possible to comprehensively dissect the complex pathogenic mechanisms of neurological disorders. This multi-omics approach spans multiple levels, from genetic variations to metabolic changes, and reveals the interactions between these levels, providing an unprecedented perspective for in-depth disease understanding. In the study of diseases such as AD, PD, stroke, epilepsy, MS, and hydrocephalus, the application of these cutting-edge technologies has greatly facilitated the

discovery of key biomarkers and significantly deepened our understanding of the molecular mechanisms of disease pathogenesis.

Figure 4: Pathogenesis of AD revealed by multi-omics and high-spatial-resolution omics technologies. This figure provides an overview of the pathogenesis of AD revealed by omics technologies, with a particular focus on several key aspects, including abnormal Tau protein, deposition of β-amyloid protein and formation of neurofibrillary tangles, neuronal loss and degeneration, disorders of lysosomal-related metabolic pathways, and neuroinflammation. In the context of Tau protein, β-amyloid protein, and plaque accumulation, the M7 MAPK module and STAT3 gene are involved. Moreover, APP, PS1, and Tau proteins constitute the main components of neurofibrillary tangles, and their abnormal alterations represent crucial pathological features of AD. The process of neuronal loss and degeneration involves APOE4, ATP6V1A, PKM2, LINGO1, OLIG, as well as histone modifications H3K9ac and H4K16ac, and SNPs. These factors act in concert, leading to the impairment of neuronal functions and the decline of cognitive abilities. In the lysosome and glycolysis-related metabolic pathways, the abnormalities of FBP1, FBP2, RHOH, CSTD, SPARC, CALB2, and CTSB, as well as the metabolic pathways of sphingolipids and aromatic amino acids, reflect cellular metabolic impairments observed in AD. In terms of neuroinflammation, PBXIP1, along with activated microglia and astrocytes, shows abnormal hyperactivity. PTPRG/VIRMA inhibitors show their impacts on mitochondrial function and neuronal survival, offering potential therapies for AD. By integrating multi-level data across the genome, transcriptome, proteome, and metabolome, multi-omics and high-spatial-resolution omics technologies provide a comprehensive view of AD pathogenesis, offering crucial insights for early diagnosis and precision therapy.

Figure 5: Multi-omics and high-spatial-resolution omics reveal key mechanisms, biomarkers, and risk factors in PD. PD is a complex neurodegenerative disorder influenced by genetic, environmental, and neurobiological factors. Mutations in genes such as ZNF184, IL1R2, IL1B, GPNMB, and LRRK2 are central to PD risk prediction and genetic susceptibility. Aberrant expression of proteins like GPNMB, CD38, SYN2, DGKQ, and biomarkers such as MAPT, SSR1, TP53, and NR2F2 is linked to PD progression and diagnostic value. These changes drive neuroinflammation and immune activation. BBB disruption facilitates the infiltration

of leukocytes and neutrophils, initiating neuroinflammation. Activated microglia and astrocytes release inflammatory cytokines (e.g., TNF-α, IL-1β, IL-6), which exacerbate the inflammatory environment and damage neurons. Lysosomal dysfunction, involving OMD, CD44, VGF, PRL, and MAN2B1, and ceRNA-Akt1 axis disruption, contribute to α-synuclein aggregation. Inflammatory biomarkers, including CircSV2b, DDC, Proline, BCAAs, and molecules in steroidogenesis and fatty acid catabolism, are also identified. Dysregulated short-chain fatty acid metabolism is associated with cognitive decline in PD. HSP90 inhibitors show therapeutic potential, as suggested by scRNA-seq of neuronal heterogeneity and molecular pathways. This figure integrates multi-level omics data to illuminate PD pathogenesis, supporting early diagnosis, monitoring, and targeted treatment.

 Click here to access/download;Table;Table.docx

Table 1: Overview of the Four Omics Technologies.

Technology	Precision	Price Range	Advantages	Disadvantages	Disease Application Representation
**Genomics**	High	High	· Conduct a comprehensive analysis of genetic sequences.<br>· Uncover the depth of genetic variations.<br>· Be suitable for gene discovery and genetic disease studies.	· High experimental costs.<br>· Complex techniques and large sample sizes.<br>· Considerable time in analysis.	· Genetic disorders.<br>· Cancer genomics.<br>· Genetic counseling.
**Transcriptomics**	Medium	Medium	· Reveal dynamic changes in gene expression.<br>· Differentiate gene regulatory networks.<br>· Assist in the classification of disease subtypes.	· High experimental and data analysis design.<br>· Limited real-time to reflect mRNA levels.	· Mental disorders.<br>· Cardiovascular diseases.<br>· Prognostication and efficacy assessment in cancer.
**Proteomics**	Low	Medium	· Reflect protein levels and modifications directly.<br>· Reveal protein-protein interaction networks.<br>· Explore changes in protein function.	· Complex data analysis with low standardization.<br>· Effects of PTMs.	· Development of tumor biomarkers.<br>· Mechanistic studies in autoimmune diseases.<br>· Pathology of neurodegenerative diseases.
**Metabolomics**	Medium	Medium	· Provide the overall metabolic profile of the organism.<br>· Reflect metabolic changes associated with disease.<br>· Assist in early diagnosis and monitoring.	· Sensitive sample handling and storage conditions.<br>· Challenges in the detection of metabolites.	· Monitoring of endocrine disorders.<br>· Prediction of cardiovascular disease risk.<br>· Metabolic testing in diabetes.

**Single-cell Omics**	High	High	· Resolve cellular heterogeneity (tumor subclones).<br>· Identify rare cell types (<0.1% population).<br>· Enable multi-omics integration (ATAC+RNA).	· Spatial information loss from tissue dissociation<br>· Significant technical noise (dropout rate >15%)<br>· Single-cell amplification bias	· Tumor evolutionary tree construction.<br>· T-cell receptor clonal tracking.<br>· Nervous diseases neuronal subtyping.
**Spatial Omics**	High	High	· Preserve in-situ spatial topology.<br>· Quantify cell-cell interactions (immune synapses).<br>· Directly correlate pathological morphology with molecular expression.	· Resolution inversely proportional to throughput (e.g., MERFISH: ~1,000 genes).<br>· Optical diffraction limitations (>200nm).<br>· High complexity in multidimensional data integration.	· Tumor immune exclusion zone mapping.<br>· Brain region-specific protein gradient atlases.<br>· Myocardial infarction spatial injury demarcation.

PTMs: post-translational modifications.

Table 2: Comparative Analysis of Five Mainstream Proteomics Techniques.

Technology	Introduction	Advantages	Disadvantages	Labeling Groups	Data Volume	Cost
**iTRAQ**	Employ chemical labels to identify proteins.	· Simultaneous analysis of up to 8 groups.<br>· Processing of multiple samples.<br>· Enhanced throughput.	· Expensive reagents.<br>· Complex experimental procedures.	4 or 8	Medium	High
**TMT**	Utilize chemical labeling to identify proteins.	· Simultaneous analysis of up to 10 or 11 groups.<br>· Simultaneous processing of more samples.<br>· Higher sensitivity.	· Expensive reagents.<br>· Complex experimental operations.	10 or 11	Medium	High
**SILAC**	Introduce isotope-labeled amino acids into the culture media.	· Accurate quantification.<br>· High sensitivity.	· Requirement for cell culture.<br>· Unsuitability for clinical samples.	2 or 3	Low	Medium
**Label-free**	Detect endogenous peptides without labeling.	· No need for labeling.<br>· Simple sample preparation.<br>· Cost-effective.	· Reduced reproducibility.<br>· Slightly diminished sensitivity.	Unlimited	High	Low
**DIA/SWATH**	Obtain mass spectrometry data through a full scan.	· No labeling required.<br>· Simultaneous quantification of numerous proteins.<br>· Good reproducibility.	· Complex data analysis.<br>· Need for specialized software.	Unlimited	High	Medium

iTRAQ: isobaric tags for relative and absolute quantification; TMT: tandem mass tag technology; SILAC: stable-isotope labeling by amino acids in cell culture; DIA: data-independent acquisition; SWATH: sequential window acquisition of all theoretical mass spectral approach.

Supplementary Table S1: Application of Multi-omics and High-spatial-resolution Omics Technologies in AD.

Disease	Omics Type	Purpose	Biomarker	Relevance to AD Pathogenesis	Reference
**AD**	Genomics	Diagnosis	APOE4	· APOE4 accelerates vascular dysfunction, BBB rupture, and neuronal degeneration.<br>· APOE4 is pivotal in AD's vascular and neurodegenerative pathogenesis and serves as a marker.	Montagne A et al. (61)
**AD**	Genomics	Diagnosis	H4K16ac	· Compared to non-AD elderly participants, 25,000 peaks showed H4K16ac loss, while 9,000 showed increased H4K16ac in AD individuals.<br>· H4K16ac decreases with aging or AD-related gene sites.<br>· H4K16ac set the stage for an epigenetic link between aging and AD.<br>· H4K16ac can be a diagnostic marker for AD disease.	Nativio R et al. (62)
**AD**	Genomics	Diagnosis	H3K9ac,<br>Tau protein	· Tau, whereas non-amyloid β pathology has a broad impact on histone acetylation in AD brain.<br>· H3K9ac structural domain shows similar gain or loss of tau-related histone acetylation.<br>· Complex interactions between tau and chromatin structure.<br>· H3K9ac and Tau are biomarkers for AD.	Klein HU et al. (63)
**AD**	Transcriptomics	Diagnosis	INPPL1, PLXNB1	· The M109 module is the one most directly associated with cognitive decline and amyloid load.<br>· INPPL1 and PLXNB1 are associated with extracellular β-amyloid levels in astrocyte cultures.<br>· INPPL1 and PLXNB1 are interesting candidates for AD patients.	Mostafavi S et al. (64)

**AD**	Proteomics	Treatment	STAT3, YES1 and FYN	· STAT3, YES1, and FYN reduce neuroinflammation, tau phosphorylation, and endogenous production of amyloid-42.<br>· Drugs targeting the cytokine transducer STAT3 and the Src family tyrosine kinases, YES1 and FYN, rescued molecular phenotypes relevant to AD pathogenesis.<br>· STAT3, YES1, and FYN can be used as drug targets for the treatment.	Jackson A et al. (65)
**AD**	Proteomics	Treatment	Insulin signaling and mitochondrial electron transport chain	· Changes in hippocampal protein expression profiles in APP/PS1 and E4 knockout mice.<br>· Different expression proteins in both mouse models, participate in insulin signaling and the mitochondrial electron transport chain.<br>· Preserving mitochondrial function and boosting insulin signaling could aid in improving cognitive function for AD patients.	He K et al. (66)
**AD**	Proteomics	Diagnosis /Treatment	The phosphorylation levels of GSK3β and Ppp3caGSK3β, Ppp3ca	· The phosphorylation levels of GSK3β and Ppp3ca are closely associated with mitochondrial biogenesis.<br>· Low-dose oral copper treatment changes the phosphorylation of key hippocampal proteins involved in mitochondrial, synaptic and axonal integrity.<br>· The phosphorylation levels of GSK3β and Ppp3ca are potential diagnostic and therapeutic targets.	Chen C et al. (67)
**AD** (autosomal dominant)	Proteomics	Diagnosis	GFAP, NPTX2, PEA15, SMOC1, SMOC2, TNFRSF1B	· Six-protein prediction model (GFAP, NPTX2, PEA15, SMOC1, SMOC2, TNFRSF1B) with excellent predictive performance (AUC>0.9)<br>· Six early biomarkers far exceed the warning time window of traditional markers	Shen Y et al. (68)

**AD**	Metabolomics	Diagnosis	Sphingolipids	· Identified sphingolipids map to AD-related pathways (tau phosphorylation, amyloid metabolism, calcium homeostasis, acetylcholine biosynthesis, apoptosis AD is associated with dysregulation of transmethylation and polyamine pathways.<br>· Sphingolipids as early AD biomarkers.	Varma VR et al. (69)
**AD**	Metabolomics	Diagnosis	PKM2	· PKM is an important glycolytic enzyme associated with AD pathology.<br>· Induced neurons iNs from AD patients express cancer-associated PKM2.<br>· PKM2 promotes Warburg effect-like glycolytic reprogramming in old neurons.<br>· PKM2 specifically interacts with and enhances the transcription factors STAT3 and HIF1α, promoting AD-induced neurons' neuronal fate loss.<br>· PKM2 is a potential diagnostic target for AD.	Traxler L et al. (70)
**AD**	ScRNA-seq	Diagnosis	Myelination-related gene (LINGO1)	· Myelination plays a pivotal role in the pathophysiology of AD.<br>· The myelination-related gene LINGO1 is perturbed in neurons and glial cells in patients with AD.	Mathys H et al. (72)
**AD**	SnRNA-seq	Diagnosis	APOE	· The transcription factor EB serves as a principal regulator of lysosomal function, modulating multiple disease-associated genes in specific astrocyte subpopulations in AD.<br>· The risk gene APOE exhibits upregulated expression in specific microglia and astrocytes in AD, correlating with the severity of Tau pathology.<br>· APOE as a risk gene for AD has been confirmed.	Grubman A et al. (73)

**AD**	Spatial transcriptomics	Diagnosis	OLIG	· Early plaque-proximal dysregulation of OLIG/myelination gene co-expression networks in AD.<br>· Multicellular 57-PIG networks emerge, enriched for complement activation, oxidative stress, lysosomal dysfunction, and neuroinflammatory pathways.<br>· OLIG can be used as a marker for future AD diagnosis to lay the foundation for AD diagnosis.	Wei-Ting Chen et al. (74)
**AD**	Spatial transcriptomics	Diagnosis	SPARC, CALB2, DIRAS2, and KRT17	· 10× Visium + co-immunofluorescence of AD markers delineated gene expression architecture in human middle temporal gyrus.<br>· Cortex-specific layer-enriched DEGs, including novel candidates SPARC, CALB2, DIRAS2, and KRT17, exhibiting pronounced alterations.<br>· These genes demonstrate significant potential as diagnostic targets for AD.	Shuo Chen et al. (75)
**AD**	ScRNA-seq, Spatial transcriptomics	Treatment	Inhibitor of PTPRG or VIRMA	· PTPRG+ microglia subpopulation induces neuronal VIRMA via intercellular signaling.<br>· Neuronal PTPRG binding to VIRMA enhances RNA stability; upregulated VIRMA increases PRKN m6A, reduces its RNA stability, causing mitophagy-driven neuronal death and AD progression.<br>· PTPRG/VIRMA inhibitors show their impacts on mitochondrial function and neuronal survival, offering potential therapies for AD.	Donghua Zou et al. (76)
**AD**	Proteomics, Transcriptomics	Diagnosis	MAPK/metabolic module, matrix body module	· AD-related modules include MAPK signaling/metabolism and matrixsome modules.<br>· Matrixsome module is affected by APOE ε4 allele.<br>· MAPK/metabolism module links to cognitive decline rate.<br>· Disease modules are potential AD targets/biomarkers.	ECB et al. (78)

**AD**	Proteomics, Transcriptomics	Diagnosis	FBP1, FBP2, RHOH, JPH2, ERAp2, and SCLT1, MBP	· FBP1, FBP2, RHOH, JPH2, ERAp2, and SCLT1 are upregulated in APOE4 cases compared to average expression in the normal brain.<br>· MBP is one of the top candidate genes enhancing the relevance of myelination in AD.<br>· Biomarkers show consistent protein profiles in plasma and brain.	Madrid L et al. (79)
**AD**	Genomics, Transcriptomics, Proteomics, Metabolomics	Diagnosis	ABCA1, CPT1A, Adiponectin and NGAL	· Short-chain acylcarnitines/amino acids and medium/long-chain acylcarnitines are closely correlated with the severity of AD.<br>· Two genes (ABCA1 and CPT1A) and two proteins (Adiponectin and NGAL) participate in the regulation of acylcarnitines and amino acids in AD.<br>· ABCA1, CPT1A, Adiponectin, and NGAL may be AD diagnostic biomarkers.	Horgusluoglu E et al. (80)
**AD**	Proteomics, Transcriptomics	Diagnosis	IVD, CYFIP1 and ADD2	· Significantly higher IVD protein abundance in AD patients.<br>· CYFIP1 and ADD2 are significantly downregulated in AD patients.<br>· IVD, CYF0IP1, and ADD2 combine to diagnose AD.	San Segundo et al. (81)
**AD**	Genomics, Metabolomics	Diagnosis	CSTD, CTSB, CTSD, and GM2A	· CSTD has been validated as a marker in previous CSF and plasma samples.<br>· AD progression is clearly accompanied by increased fold changes in these lysosomal proteins.<br>· Lysosomal proteins CTSB, CTSD, and GM2A are significantly increased as markers in CSF samples from AD patients.	Wang H et al. (82)

**AD**	Genomics, Proteomics	Diagnosis	PBXIP1	· PBXIP1-encoded protein shows significant association with all three AD neuropathological features.<br>· PBXIP1 is associated with AD through its role in astrocytes and hippocampal neurons and the mTOR pathway.<br>· PBXIP1 is associated with neuropathology and cognitive function.	Jingyun Zhang et al. (83)
**AD**	Genomics, Transcriptomics, Proteomics	Diagnosis	H3K27ac	· Differentially acetylated peaks are enriched in disease-related biological pathways, including those associated with amyloid-β and tau pathology progression.<br>· Highly significant enrichment of AD risk variants in the H3K27ac peak region of the inner olfactory cortex, including CR1, GPR22, KMO, PIM3, PSEN1, and RGCC.<br>· H3K27ac can serve as a diagnostic target for AD.	Marzi SJ et al. (84)
**AD**	Transcriptomics, Proteomics, Epigenomics	Diagnosis	H3K27ac, H3K9ac	· RNA-seq analysis reveals upregulation of histone acetyltransferases in H3K27ac and H3K9ac.<br>· Genome-wide increases in H3K27ac and H3K9ac exacerbate amyloid-β42-driven neurodegeneration.<br>· Proteomic screening singles out H3K27ac and H3K9ac as major AD-specific enrichments.<br>· H3K27ac and H3K9ac affect disease pathways through dysregulated transcription and chromatin gene feedback loops.	Raffaella Nativio et al. (85)
**AD**	Proteomics, Metabolomics, Lipidomics	Diagnosis	Protein 14-3-3 zeta/delta, clusterin, interleukin-15, and transgelin-2	· Enrichment pathway analysis reveals overexpression of hemostatic, immune response, and extracellular matrix signaling pathways associated with AD.<br>· Protein 14-3-3 zeta/delta, clusterin, interleukin-15, and transgelin-2 improve AD prediction.	Clark C et al. (86)

**AD**	Proteomics, Metabolomics, Lipidomics	Diagnosis	GABA synthesis, arginine biosynthesis, and alanine, aspartate, glutamate, and arginine pathways	· Gender-dependent effects are seen on the pathways of significant enrichment, including those of GABA synthesis, arginine biosynthesis, and alanine, aspartate, glutamate, and arginine metabolism.<br>· Lysophospholipid and amino acid metabolism are involved in the AD brain.	Abigail Strefeler et al. (87)
**AD**	Genomics, Transcriptomics	Treatment	TRPV1	· TRPV1 activation rescues memory deficits and neuronal loss in ApoE4 high-fat diet-fed mice.<br>· Neuronal loss increases in ApoE4 high-fat diet mice, rescued by TRPV1 activation in the capsaicin group.<br>· TRPV1 is a treatment option for AD disease.	Chenfei Wang et al. (88)
**AD** (late-onset)	Genomics, Transcriptomics	Treatment	ATP6V1A	· ATP6V1A has been identified as a key regulator of the top neuronal subnetwork, which is the most dysregulated in late-onset AD.<br>· ATP6V1A can be used as a therapeutic target.<br>· NCH-51 ameliorates neuronal damage caused by ATP6V1A deficiency in a drosophila model.	Wang M et al. (89)

AlzGPS: Alzheimer's disease genome-wide positioning systems platform; CSF: cerebrospinal fluid; PKM2: pyruvate kinase M2; MBP: Myeloid basic protein encoding gene.

Supplementary Table S2: Application of Multi-omics and High-spatial-resolution Omics Technologies in PD.

Disease	Omics Type	Purpose	Biomarker	Conclusion	Reference
**PD**	Genomics	Diagnosis	ZNF184, IL1R2、LRRK2, ITPKB, and PARK16	· Alleles of LRRK2 and IL1R2 confer a higher risk of developing PD.<br>· The genotype models of ZNF184, PARK16, and ITPKB are significantly associated with PD.<br>· Most of these genes are involved in autophagy and lysosomal function-related pathways.	Gao T et al. (96)
**PD**	Genomics	Diagnosis	HLA, LRRK2, MAPT, TRIM10, and SETD1A	· HLA, LRRK2, MAPT, TRIM10, and SETD1A are high-risk genes associated with PD.<br>· Significantly associated loci linked to PD are found in the HLA and MAPT gene loci.	Witoelar A et al. (97)
**PD**	Transcriptomics	Diagnosis	SSR1	· SSR1 is found to be upregulated in PD patients.<br>· SSR1 expression is negatively correlated with dopaminergic neuron survival.<br>· The upregulation of SSR1 expression in peripheral blood precedes the abnormal behavior of the animals.<br>· The SSR1-based RF classifier has an AUC value of 0.91 and can be used as a diagnostic marker.	Zhang W et al. (98)
**PD**	Proteomics	Diagnosis	OMD, CD44、VGF, PRL, MAN2B1, and LRRK2	· ML identifies that OMD, CD44, VGF, PRL, and MAN2B1 show significant changes in PD patients and are significantly correlated with PD clinical scores.<br>· The enhanced neuroinflammatory characteristics in LRRK2 gene carriers are strongly correlated with PD.<br>· OMD, CD44, VGF, PRL, MAN2B1, and LRRK2 can be used as biomarkers for PD.	Karayel, Matthias Man et al. (99)

**PD**	Metabolomics	Diagnosis	Lipid metabolism related to carnitine shuttle, sphingolipid metabolism, and arachidonic acid metabolism	· Alterations in lipid metabolism related to carnitine shuttle, sphingolipid metabolism, arachidonic acid metabolism, and fatty acid biosynthesis are detected.<br>· Carnitine shuttling is the most important pathway associated with unmedicated PD patients by sebum.	Sinclair E et al. (100)
**PD**	Metabolomics	Diagnosis	Short-chain fatty acids, butyric acid	· Low levels of short-chain fatty acids are significantly associated with cognitive decline in PD patients.<br>· Decreased butyric acid levels are associated with poorer posture and gait disorder scores.<br>· Short-chain fatty acids and butyric acid serve as a potential diagnostic target.	Tan AH et al. (101)
**PD**	Metabolomics	Diagnosis	Proline	· Energy and lipid metabolism are overexpressed in PD.<br>· 139 metabolites, including proline, have notable changes in carnitine shuttle, vitamin E metabolism, lipid-related, glycerol phospholipids, sphingolipids, and fatty acids pathways.<br>· Proline and 139 other metabolites are considered particularly predictive of PD status.	Pereira, P.A.B et al. (102)
**PD**	Metabolomics	Diagnosis	Phenylacetic acid, phenylacetylglutamine, histidine, uric acid, and imidazoleacetic acid	· 45 metabolic markers in PD patients show high diagnostic power in early stages (AUC=0.92).<br>· Metabolites like phenylacetic acid, phenylacetylglutamine, histidine, uric acid, and imidazoleacetic acid show upregulated urine levels in PD, linked to neuro disorders.	Cai Z et al. (103)

**PD**	Metabolomics	Diagnosis	BCAA metabolism, glycine derivatives, steroid hormone biosynthesis, tryptophan, and phenylalanine metabolism	· 18 differential metabolites in urine have been identified as biomarkers for PD.<br>· Differential metabolites alter metabolic pathways associated with BCAA metabolism, glycine derivatives, steroid hormone biosynthesis, tryptophan metabolism, and phenylalanine metabolism.	Cai Z et al. (104)
**PD**	SnRNA-seq	Diagnosis	IL1B, GPNMB, and HSP90AA1	· A neuron cluster characterized by CADPS2 overexpression and low tyrosine hydroxylase levels is identified in PD.<br>· Astrocytes and microglia in PD show specific proliferation and gene dysregulation linked to unfolded protein response and cytokine signaling.<br>· Microglia show a pro-inflammatory state with high IL1B, GPNMB, and HSP90AA1, suggesting their diagnostic potential.	Semra Smajić et al. (197)
**PD**	ScRNA-seq	Treatment	HSP90 inhibitors	· Neurons and glial cells in PD exhibit dysfunction, immune dysregulation, and impaired protein folding.<br>· Administration of HSP90 inhibitors accelerates the degradation of inflammasomes, reducing inflammatory responses and alleviating neurodegeneration.	Gabriel GE et al. (105)

**PD**	Single-cell genomics, Spatial transcriptomics	Diagnosis	TP53, NR2F2	· The AGTR1-marked SNpc ventral subtype is highly PD-susceptible, showing TP53/NR2F2 target gene upregulation.<br>· TP53/NR2F2-regulated pathways are key to PD-related neuronal death.<br>· TP53/NR2F2 target gene upregulation indicates diagnostic biomarker potential.	Tushar Kamath et al. (106)
**PD**	ScRNA-seq, Proteomics	Diagnosis	SYN2	· Negative correlation between α-synuclein pathology and chaperone protein expression in excitatory neurons in PD, along with weakened neuron-astrocyte interaction and aggravated neuroinflammation.<br>· SYN2 enrichment in PD brain regions suggests significant increase in synaptic signaling at both RNA and protein levels.<br>· SYN2 as a potential diagnostic biomarker for PD	Biqing Zhu et al. (107)
**PD**	Proteomics, Transcriptomics	Diagnosis	GPNMB, CD38, and DGKQ	· GPNMB and CD38 show significant causal effects in PD, with evidence from quantitative trait locus analysis and fine mapping.<br>· GPNMB, CD38, and DGKQ proteins are associated with PD risk.	Guxiaojing et al. (108)
**PD**	Three Proteomics	Diagnosis	DDC	· DDC, SUMF1, DPP7, ENPEP, WFDC2, and hundreds of proteins are upregulated in the CSF, blood, or urine of PD patients.<br>· DDC levels are linked to symptom severity in PD patients.<br>· DDC can serve as a target for accurate PD diagnosis.	Rutledge J et al. (109)
**PD**	Transcriptomics, Metabolomics	Treatment	The relaxin signaling pathway, adhesion patch, and PI3K-Akt signaling pathway	· BHD reduces PD symptoms, impacting metabolic pathways, including the relaxin signaling pathway, adhesion patch, and PI3K-Akt signaling pathway.<br>· BHD promotes the survival of dopaminergic neurons in PD mice, leading to improved motor performance.	Hujun et al. (110)

**PD**	Genomics, Metabolomics	Diagnosis/ Treatment	CircSV2b	· Detect 33 deregulated circular RNAs in the PD mouse model vs wild-type controls.<br>· CircSV2b overexpression via the ceRNA-Akt1 axis mitigates oxidative stress in PD.<br>· CircSV2b is a potential Parkinson's diagnostic and curative biomarker.	Cheng Qi et al. (111)

BCAA: branched chain amino acid; SNpc: substantia nigra pars compacta; DDC: Dopamine decarboxylase; ML: machine learning; LRRK2: leucine-rich repeat kinase 2; SUMF1: sulfatase-modifying factor 1; DPP7: dipeptidyl peptidase 2/7; SSR1: signal sequence receptor subunit 1; ENPEP: glutamyl aminopeptidase; WFDC2: WAP four-disulfide core domain 2; BHD: Buyang Huanwu Decoction.

Supplementary Table S3: Application of Multi-omics and High-spatial-resolution Omics Technologies in Epilepsy.

Disease	Omics Type	Purpose	Biomarker	Conclusion	Reference
**Epilepsy**	Transcriptomics	Diagnosis	p38MAPK, Jak-STAT, PI3K, and mTOR signal pathway	· The p38MAPK, Jak-STAT, and PI3K consistently exhibit high expression and along with stable regulation of mTOR signaling pathways in epilepsy patients.<br>· Differential genes engage in signal cascades, ECM remodeling, cell motility, apoptosis, and immune responses linked to seizures.	Oswaldo K Okamoto et al. (115)
**Epilepsy** (temporal lobe)	Transcriptomics	Diagnosis	Tlr2, Lgals3, Serpine 1 and Stat3 et al.	· Several hub genes identified in TLE, such as Tlr2, Lgals3, Serpine1, and Stat3, et al., positively correlate with seizure frequency.<br>· Activation and phagocytic activity of microglia/macrophages have changed during the epileptic occurrence process of TLE.<br>· Tlr2, Lgals3, Serpine 1, and Stat3 can serve as markers for TLE.	QingLan Chen et al. (116)
**Epilepsy**	Proteomics	Diagnosis	GFAP	· GFAP is consistently downregulated in brain tissue with high spike frequencies and exhibits a strong negative correlation with spike frequency.<br>· Reactive astrocytes, such as GFAP, protect the neocortex from epileptic discharges rather than induce them.<br>· Epilepsy severity is closely linked to decreased GFAP (astrocyte marker) levels.	Gal Keren-Aviram et al. (117)

**Epilepsy**	Proteomics	Diagnosis/ Treatment	ADPRC, LPAR3, calreticulin, UCH-L1, SNAP-25, and transgelin-3	· A total of 144 differentially expressed proteins, such as ADPRC, LPAR3, calreticulin, UCH-L1, SNAP-25, and transgelin-3, are identified in the epileptic hippocampal regions.<br>· Most differentially expressed proteins are associated with Ca2+ homeostasis.<br>· Inhibiting calcium influx alleviates seizures triggered by excessive brain Ca^{2}+ rise in epilepsy.	Leila Sadeghi et al. (118)
**Epilepsy**	Proteomics	Diagnosis	Calcineurin	· Tutin induces epilepsy by activating calcium-modulating phosphatase and produces significant neurological damage.<br>· Calcineurin is a target of tutin, and that tutin activates Calcineurin, leading to seizures.	Shi-Shan Yu et al. (119)
**Epilepsy**	Metabolomics	Diagnosis	N-acetyl glycoprotein, lactate, creatine, glycine, lipid, and citrate	· Serum N-acetyl glycoprotein, lactate, creatine, glycine, and lipid levels are elevated decreased levels of citrate in epileptic children, while the level of citrate is reduced.<br>· The aforementioned metabolic substances are potential diagnostic targets for epilepsy.	Łukasz Boguszewicz et al. (120)
**Epilepsy** (mesial temporal lobe)	Metabolomics	Diagnosis	GABA	· GABA is significantly increased in the epileptogenic zone of KA-MTLE mice.<br>· GABA is a specific biomarker of the epileptogenic zone in MTLE.	Hamelin, S et al. (121)
**Epilepsy**	SnRNA-seq	Diagnosis	Sst and Pvalb	· Major transcriptomic alterations occur in principal neurons (L5-6_Fezf2, L2-3_Cux2) and GABAergic interneurons (Sst, Pvalb).<br>· Profound dysregulation in glutamate signaling, characterized by robust upregulation of glutamate receptor genes, notably within Sst/Pvalb subtypes.<br>· Sst/Pvalb interneurons represent potential diagnostic targets and are fundamental to early epileptogenesis.	Ulrich Pfisterer et al. (122)

**Epilepsy** (post-traumatic)	ScRNA-seq	Diagnosis	XIST	・Hereditary epilepsy shows higher oligodendrocyte/astrocyte counts, lower microglia/neuron counts vs PTE.<br>・IL-17 signaling in microglia/astrocytes can be a PTE target/biomarker.<br>・XIST, upregulated in PTE, drives inflammation/fibrosis, useful for diagnosis and mechanism study.	Fang Wen et al. (123)
**Epilepsy** (temporal lobe)	ScRNA-seq, SnRNA-seq, Spatial transcriptomics	Diagnosis	Spp1, Trem2, Tle4 and Sipa1l3	・The differentially up-regulated genes in TLE patients are predominantly expressed in glial cells, while the down-regulated genes are mainly expressed in neurons.<br>・Spp1 and Trem2 are up-regulated in glial cells, whereas Tle4 and Sipa1l3 are down-regulated in these cells.	Quanlei Liu et al. (124)
**Epilepsy**	Genomics, Transcriptomics	Diagnosis	Sestrin 3	・Sestrin 3 is a key regulator in the pro-convulsant gene network in the hippocampus of human epilepsy.<br>・Sestrin 3 positively regulates modules in macrophages, microglia, and neurons.<br>・Sestrin 3 holds potential as a diagnostic means for epilepsy.	Johnson, M.R. et al. (125)
**Epilepsy**	Proteomics, Transcriptomics	Diagnosis	STAT3, ErbB, and Mapk8	・The TGF-β pathway is associated with cardiac function in the hearts of epileptic animals.<br>・STAT3, ErbB, and Mapk8 are key regulators of cardiac alterations in epilepsy that contribute to seizure-mediated cardiac damage.	Sharma, S et al. (126)
**Epilepsy**	Proteomics, Metabolomics	Diagnosis	GSTM1, ALDH2	・Within the somatosensory cortex module, GSTM1 is identified as a protein hub and elevated expression levels.<br>・In the thalamus module, ALDH2 is pinpointed as a protein hub.<br>・The metabolic pathway enriched by the differences is lysine degradation.<br>・GSTM1 and ALDH2 are identified as markers for seizure-related modules in epilepsy.	Harutyunyan, A et al. (127)

**Epilepsy**	Genomics, Metabolomics	Diagnosis	Lactate, creatine, phosphocreatine, and choline	· Lactate is significantly reduced, while creatine, phosphocreatine, and choline are significantly increased.<br>· Lactate is involved in G protein-coupled receptor signaling and angiogenic pathways, and shows upregulation of ubiquitination-related genes.	Wu, H.C. et al. (128)
**Epilepsy**	Proteomics, Transcriptomics	Treatment	miR-10a-5p, miR-21a-5p and miR-142a-5p	· miR-10a-5p, miR-21a-5p, and miR-142a-5p are identified as key transcripts.<br>· These microRNA transcripts are primarily associated with the TGF-β pathway signaling.<br>· The combination of anti-miR (miR-10a-5p, miR-21a-5p, miR-142a-5p) exhibits protective effects against acute and spontaneous seizures.	Venø, M.T et al. (129)

PTE: post-traumatic epilepsy; TLE: temporal lobe epilepsy; MTLE: mesial temporal lobe epilepsy; NMDA: N-methyl-D-aspartate; GABA: γ-aminobutyric acid; GFAP: glial fibrillary acidic protein; KA-MTLE: kainic acid into mesiotemporal lobe epilepsy mice; TGF-β, transforming growth factor β; ADPRC: ADP-ribosyl cyclase; LRRK2: leucine-rich repeat kinase 2; LPAR3: lysophosphatidic acid receptor 3; UCH-L1: ubiquitin carboxyl-terminal hydrolase L1; GSTM1: glutathione s-transferase M1; SNAP-25: synaptosome-associated protein 25.

Supplementary Table S4: Application of Multi-omics and High-spatial-resolution Omics Technologies in MS.

Disease	Omics Type	Purpose	Biomarker	Conclusion	Reference
**MS**	Proteomics	Diagnosis	CXCL13, LTA, FCN2, ICAM3, LY9, SLAMF7, TYMP, CHI3L1, FYB1, TNFRSF1B, and NFL	· Lower levels of NFL in CSF show predictive potential for disease activity (AUC=0.77).<br>· An 11-protein panel in CSF has a high AUC for prediction, including CXCL13, LTA, FCN2, ICAM3, LY9, SLAMF7, TYMP, CHI3L1, FYB1, TNFRSF1B, and NFL (AUC=0.9).<br>· All the above proteins can be markers for MS.	Mika Gustafsson et al. (136)
**MS**	Metabolomics	Diagnosis	DRD2	· DRD2 exacerbates the disease by promoting inflammation and reducing the abundance of Lactobacillus species in the microbiome.<br>· Lactobacillus-derived N2-acetyl-L-lysine inhibits microglial activation, combating neurodegeneration.<br>· Intestinal epithelial DRD2, serving as a biomarker, can modulate the gut microbiome in MS.	Hairong Peng et al. (137)
**MS** (relapsing-remitting)	Metabolomics	Diagnosis/ Treatment	Glycolysis	· Identified four perturbed metabolic pathways, including structural/signaling lipids and energy, in the serum of patients with MS.<br>· Glycolysis is the common upstream feeding of these altered metabolic pathways.<br>· Targeting glycolysis in experimental autoimmune encephalomyelitis ameliorated the disease pathology by impeding immune cell effector function.	Insha Zahoor et al. (138)

**MS**	ScRNA-seq	Diagnosis	TFH	· Myeloid dendritic cells and regulatory T cells are enriched in the CSF of patients with MS.<br>· The independent increase in clusters of TFH cells drives the known expansion of B-lineage cells in the CSF in MS.<br>· TFH cells promote the infiltration of B cells into the central nervous system, exacerbating MS disease.	David Schafflick et al. (139)
**MS**	ScRNA-seq, Spatial transcriptomics	Diagnosis	SERPINA3	· Astrocytes can be classified into three types: homeostatic, intermediate, and disease-associated types.<br>· In patients with DA-Astro, the expression level of SERPINA3 is significantly elevated.<br>· SERPINA3 expression may constitute a glial cell survival response to resolve inflammation and prevent apoptosis during both initial and late resolution phases.	Petra Kukanja et al. (140)
**MS**	ScRNA-seq, Spatial transcriptomics	Diagnosis	MAFB	· The expression of pro-inflammatory molecules in oligodendrocytes near axonal damage is elevated in MS patients.<br>· MAFB mediates intercellular communication via complement factors and apolipoproteins.<br>· The inflammatory transcription factor MAFB serves as a biomarker for MS lesions.	Maria L Elkjaer et al. (141)
**MS (systemic)**	ScRNA-seq, Spatial transcriptomics, Spatial proteomics	Diagnosis/ Treatment	POSTN/SCARA5, CXCR4	· A dynamic spatial interaction network is established between fibroblasts and macrophages via the ACKR3-CXCL12-CXCR4 signaling axis, playing a central role in driving fibrosis progression.<br>· Treatment with the CXCR4 inhibitor AMD3100 significantly alleviates fibrosis in skin and lung tissues.<br>· The significantly elevated POSTN/SCARA5 ratio in MS can serve as a predictive diagnostic biomarker.	Zhijian Li et al. (142)

**MS**	Proteomics, Transcriptomics	Diagnosis	GPR37L1, SIRPA, FGFR3, CADM3, and TYRO3	· Neurological candidate molecules, including GPR37L1, SIRPA, FGFR3, CADM3, and TYRO3, are highly expressed in the CNS of MS.<br>· These genes are associated with early neuronal degeneration and dysfunctional trophic/anti-inflammatory intercellular communication.<br>· GPR37L1, SIRPA, FGFR3, CADM3, and TYRO3 can be used as a diagnostic method for MS.	Max Kaufmann et al. (143)
**MS**	Proteomics, Transcriptomics	Diagnosis	24 iron death-related genes (CHMP5, SLC38A1, PML, etc.)	· High iron death scores at the margins of active lesions correlate with phagocytic activation.<br>· Elevated iron death scores in cortical neurons are associated with neurological diseases.<br>· A blood-based model of 24 iron death-related genes is a prognostic marker for diagnosing MS, including CHMP5, SLC38A1, PML, etc.	Tao Wu et al (144)
**MS**	Proteomics, Metabolomics	Diagnosis	LAMP1, FCG2A, and HPSE	· HPSE is positively correlated with many MS-related metabolites, including L-tyrosine, sphingosine 1-phosphate, sphingosine 1-phosphate, and L-tryptophan.<br>· The proteins LAMP1, FCG2A, and HPSE exhibit potential utility as specific biomarkers for MS.	Fan Yang et al. (145)
**MS**	Proteomics, Metabolomics	Diagnosis	Equine uric acid, sphingolipids	· Anti-inflammatory molecules and sphingolipids are reduced by metabolomics in MS patients.<br>· Low levels of equine uric acid in a severe subgroup of MS.<br>· Sphingolipids and equine uric acid facilitate the future development of biomarkers and targeted therapeutic interventions for MS.	Qinming Zhou et al. (146)

NFL: neurofilament light chain; DRD2: Dopamine Receptor D2; CNS: central nervous system; HPSE: heparinase; TFH: T follicular helper.

Supplementary Table S5: Application of Multi-omics and High-spatial-resolution Omics Technologies in Stroke.

Disease	Omics Type	Purpose	Biomarker	Conclusion	Reference
**Stroke** (ischemic)	Proteomics	Diagnosis	NSF, RhoGDI1, and RabGDI	· Circulating NSF, RhoGDI1, and RabGDI are upregulated in patients with IS.<br>· These proteins trigger neuronal depolarization and calcium surge, activating death pathways in stroke.	Eloy Cuadrado et al. (149)
**Stroke** (ischemic)	Proteomics	Diagnosis	CMPK, CKB	· Circulating levels of CKB and CMPK are higher in patients with ischemic stroke than in controls during the acute phase.<br>· CKB plays a crucial role in energy transduction and homeostasis.<br>· CMPK is released in large amounts and participates in mechanisms that counteract cell disruption and neuronal cell death.	Alba Simats et al. (150)
**Stroke** (ischemic)	Proteomics	Diagnosis	SAHH2	· SAHH2 plays a significant role in the coordinated inhibition of Ca^{2+} ion transporters.<br>· Increased expression of SAHH2 in neurons from the infarcted area is probably because of ischemia-triggered Ca2+ mobilization.	Teresa García -Berrocoso et al. (151)
**Stroke**	Transcriptomics	Diagnosis	LncRNA (MEG3, H19, and MALAT1)	· LncRNAs, such as MEG3, H19, and MALAT1, in blood cells between patients with stroke and healthy controls show differences.<br>· Differential genes modulate neuronal survival/apoptosis targets, impacting p53-mediated apoptosis in stroke.	Cheryl Dykstra Aiello et al. (152-156)

**Stroke**	Transcriptomics	Diagnosis	Extracellular microRNA	· Decreased levels of extracellular miR-32-3p, miR-106b-5p, miR-423-5p, miR-451a, miR-1246, miR-1299, miR-3149 and miR-4739, and increased levels of extracellular miR-224-3p, miR-377-5p, miR-518b, miR-532-5p and miR-1913 associate with stroke.<br>· These genes affect multiple pathways such as apoptosis, oxidation, angiogenesis, and neurogenesis in IS.	Ceren Eyileten et al. (157)
**Stroke** (cardioembolic)	Metabolomics	Diagnosis	Valine, Leucine, Isoleucine	· The expression levels of BCAA, including valine, leucine, and isoleucine, are decreased in patients with cardioembolic stroke.<br>· Lower BCAA levels are also associated with poor neurological outcomes.	W Taylor Kimberly et al. (158)
**Stroke**	Metabolomics	Diagnosis	Total free fatty acid	· Plasma concentration of total free fatty acids is higher in patients with cardioembolic stroke than in patients with non-cardioembolic stroke.<br>· Elevated free fatty acid levels are significantly associated with cardioembolic stroke, suggesting their potential as a diagnostic target.	Jeong Yoon Choi et al. (159)
**Stroke**	ScRNA-seq	Treatment	Microglia and macrophages	· Aging jeopardizes the repair and regeneration of the cerebrovascular system and proteins after stroke.<br>· After stroke, microglia and macrophages may affect angiogenesis and oligodendrogenesis via paracrine mechanisms, impeding stroke recovery.<br>· Transplanting microglia and macrophages from the brains of young mice into the cerebral cortex of aged stroke-affected mice partially restores angiogenesis and oligodendrogenesis.<br>· Microglia and macrophages serve as effective targets for promoting stroke recovery	Chenghao Jin et al. (160)

**Stroke** (ischemic)	ScRNA-seq, Spatial transcriptomics	Treatment	LILRB4	· Stroke brains have up-regulated LILRB4 and ischemia-linked microglial cluster 3.<br>· LILRB4 knockout worsens ischemic brain injury via CD8+ T cell recruitment; overexpression offers neuroprotection.<br>· Targeting LILRB4 and its downstream pathways represents an effective therapeutic strategy for ischemic stroke.	Yilin Ma et al. (161)
**Stroke** (brainstem)	SnRNA-seq, ScRNA-seq	Treatment	Myo1e	· Oligodendrocyte loss leads to neurological deficits following brainstem stroke.<br>· OLG8 has an innate neuroprotective effect in brainstem stroke.<br>· Myo1e aids OLG8 migration to the peri-infarct area in brainstem stroke.<br>· Myo1e overexpression in OLG8 oligodendrocytes boosts brainstem stroke recovery.	Shaojun Li et al. (162)
**Stroke** (intracerebral hemorrhage)	ScRNA-seq, Spatial transcriptomics	Diagnosis	Spp1, Lyz2	· Spp1/Lyz2 show high expression levels, and lymphocytes with high expression interact with myeloid cells in the late stage of stroke.<br>· During the acute phase of intracerebral hemorrhage, Lgmn+Macro-T cells and microglia interact via the Spp1-cd44 pathway.<br>· Spp1 and Lyz2 are potential diagnostic targets for the acute phase of intracerebral hemorrhage.	Lingui Gu et al. (163)
**Stroke**	ScRNA-seq, Spatial transcriptomics	Treatment	Lipocalin-2	· Ferroptosis is the primary programmed cell death process post-hemorrhagic stroke, mainly affecting mature oligodendrocytes.<br>· A specific interaction between lipocalin-2-positive microglia and oligodendrocytes, mediated by the CSF1 receptor pathway, induces ferroptosis in oligodendrocytes and subsequent neurological deficits.<br>· Early therapeutic intervention by inhibiting LCN2 expression may alleviate ferroptosis-induced oligodendrocyte damage and related neurological deficits.	Lingui Gu et al (164)

**Stroke**	ScRNA-seq, Spatial transcriptomics	Treatment	LGALS9	· Galectin (LGAL) signaling is enhanced in microglia and macrophages of ischemic mice.<br>· LGALS9 treatment promotes oligodendrocyte remyelination and improves stroke recovery in mice.<br>· LGALS9 can serve as a therapeutic approach to ameliorate stroke.	Bing Han et al. (165)
**Stroke (subarachnoid hemorrhage)**	ScRNA-seq, Spatial transcriptomics	Diagnosis	THBS1, S100A6	· THBS1 and S100A6 are closely associated with the prognosis of SAH, with their expression significantly increasing following the hemorrhage.<br>· The THBS1-CD47 pair regulates cell apoptosis, and blocking their interaction may represent a new therapeutic approach for SAH.<br>· THBS1 and S100A6 serve as diagnostic biomarkers for stroke.	Xiaoyu Wang et al. (166)
**Stroke** (cardioembolic)	Proteomics, Transcriptomics	Diagnosis	ICA1L, CAND2, and ALDH2	· Reduced ICA1L, CAND2, and ALDH2 may impair excitatory synaptic signaling, contributing to cardioembolic stroke pathogenesis.<br>· ICA1L, CAND2, and ALDH2 are potential biomarkers for lacunar stroke.	Zhang C et al. (167)
**Stroke** (ischemic)	Genomics, Metabolomics	Treatment	Gut flora and metabolic disturbances	· ZHTC modulates the abundance of specific bacterial groups and 23 metabolic differences for IS, including arginine, L-lysine, and L-methionine.<br>· ZHTC improves intestinal barrier integrity by increasing the expression levels of tight junction proteins<br>· ZHTC meliorates IS by modulating gut flora and metabolic disturbances.	Wang R et al. (168)

**Stroke** (ischemic)	Proteomics, Transcriptomics	Treatment	PI3K-Akt, MAPK, and cAMP signaling pathways	· YQTL reduces infarct volume percentage and improves neurological function in cerebral ischemia-reperfusion injury mice.<br>· Network pharmacology and multi-omics studies reveal 15 components that regulate 82 targets and 19 pathways.<br>· YQTL protects against cerebral ischemia-reperfusion injury through PI3K-Akt, MAPK, and cAMP signaling pathways.	Yuan Y et al et al. (169)

lncRNAs: long non-coding RNAs; IS: Ischemic Stroke; BCAA: branched-chain amino acid; LGAL: Galectin; CSF1: colony-stimulating factor 1; SAH: subarachnoid hemorrhage; ZHTC: Zhilong Huoxue Tongyu capsule; YQTL: Yiqi Tongluo granule; ALDH2: aldehyde dehydrogenase 2.

Supplementary Table S6: Application of Multi-omics and High-spatial-resolution Omics Technologies in Hydrocephalus.

Disease	Omics Type	Purpose	Biomarker	Conclusion	Reference
**Hydrocephalus** (communicating)	Genomics	Diagnosis	TRIM71, SMARCC1, PIK3CA, PTEN, MTOR, FOXJ1, FMN2, PTCH1, and FXYD2	· TRIM71 and SMARCC1 exhibit genome-wide significant enrichment of de novo mutations, which may be genuine risk factors for CH.<br>· PIK3CA, PTEN, MTOR, FOXJ1, FMN2, PTCH1, and FXYD2 are newly identified high-confidence sporadic CH genes.<br>· TRIM71 and other genes, reducing neural cell proliferation to cause hydrocephalus, can be a diagnostic marker.	Sheng Chih Jin et al. (177)
**Hydrocephalus** (communicating)	Proteomics	Diagnosis	KLK6	· Expression of KLK6 is significantly up-regulated in CH patients.<br>· KLK6 is involved in CH development and may provide a new target for CH diagnosis.	Lei Yuan et al. (178)
**Hydrocephalus** (idiopathic normal pressure)	Proteomics	Diagnosis	QPCT, RBP4	· 39 proteins exhibit a significant increase, while 285 proteins show a significant decrease in CSF of iNPH.<br>· Elevated proteins mainly relate to myeloid leukocyte migration and extracellular matrix organization; reduced ones are linked to axon and synaptic development.<br>· QPCT and RBP4 have been identified as potential protein biomarkers in iNPH for predicting shunt outcomes.	Yuqi Ying et al. (179)

**Hydrocephalus** (idiopathic normal pressure)	Proteomics	Diagnosis	PTPRQ	· PTPRQ concentrations in CSF are significantly higher in iNPH patients than in AD patients.<br>· PTPRQ concentration in the CSF of non-responders to shunt operation tended to be relatively lower compared with that in the responders.<br>· PTPRQ is a candidate biomarker to distinguish iNPH from AD.	Yuki Nagata et al. (180)
**Hydrocephalus** (idiopathic normal pressure**)**	Metabolomics	Diagnosis	Glyceric acid, N-acetyl neuraminic acid, serine, and 2-hydroxybutyric acid	· Elevated glyceric acid and N-acetyl neuraminic acid, and reduced serine and 2-hydroxybutyric acid in AD CSF distinguish it from iNPH.<br>· Serine, glyceric acid, Neu5Ac, and 2-hydroxybutyrate combine as a diagnostic iNPH biomarker.	Yuki Nagata et al. (181)
**Hydrocephalus** (normal pressure)	Metabolomics	Treatment	Neu5Ac	· CSF Neu5Ac levels are low in NPH patients.<br>· Boosting brain Neu5Ac inhibits astrocyte activation.<br>· Brain Neu5Ac elevation reduces periventricular demyelination and improves hydrocephalus.<br>· Enhanced brain Neu5Ac improves neurological outcomes in NPH, suggesting a potential treatment.	Zhangyang Wang et al. (182)
**Hydrocephalus** (tumor-associated)	SnRNA-seq, Spatial transcriptomics	Diagnosis/ Treatment	CPMCs,	· Ventricular cell atlas reveals CPMC expansion in TAH mice.<br>· CPMCs compromise ependymal ciliary integrity via tryptase-PAR2-FoxJ1 signaling, triggering pathological CSF hypersecretion that underlies hydrocephalus pathogenesis.<br>· Brain barrier-penetrating trypsin-like inhibitor BMS-262084 effectively inhibits TAH progression in vivo and attenuates mast cell-induced epithelial cilia damage.	Yiye Li et al. (183)

**Hydrocephalus** (communicating)	Genomics, Proteomics, Transcriptomics	Diagnosis	MAEL	· PrediXcan analysis in 10 neuro tissues and whole blood shows a correlation between reduced MAEL gene expression in the brain and hydrocephalus ($p < 0.05$).<br>· Reduced MAEL expression increases susceptibility to hydrocephalus.<br>· MAEL is a diagnostic biomarker for hydrocephalus.	Andrew T Hale et al. (184)
**Hydrocephalus** (post-hemorrhagic)	Proteomics, Metabolomics	Diagnosis/ Treatment	CSPG4	· CSPG4 positively correlates with ventricular size and the incidence of periventricular leukomalacia.<br>· Silencing of CSPG4 can inhibit ferroptosis, cell adhesion functions, and intracellular Ca2+ flux.<br>· CSPG4 has been identified as a CSF biomarker and effective therapeutic target.	Juncao Chen et al. (185)

NPH: normal pressure hydrocephalus; iNPH: idiopathic normal pressure hydrocephalus; CH: communicating hydrocephalus; Neu5Ac: N-acetylneuraminic acid; TAH: tumor-associated hydrocephalus; CPMCs: choroid plexus mast cells; MAEL: maelstrom spermatogenic transposon silencer; KLK6, kallikrein-6; QPCT: glutaminyl-peptide cyclotransferase; RBP4: retinol-binding protein 4; PTPRQ: Q-type protein tyrosine phosphatase receptor; CSPG4: chondroitin sulfate proteoglycan 4.

Figure1

Click here to access/download;Figure;Figure1-Metabolomics_type.pdf

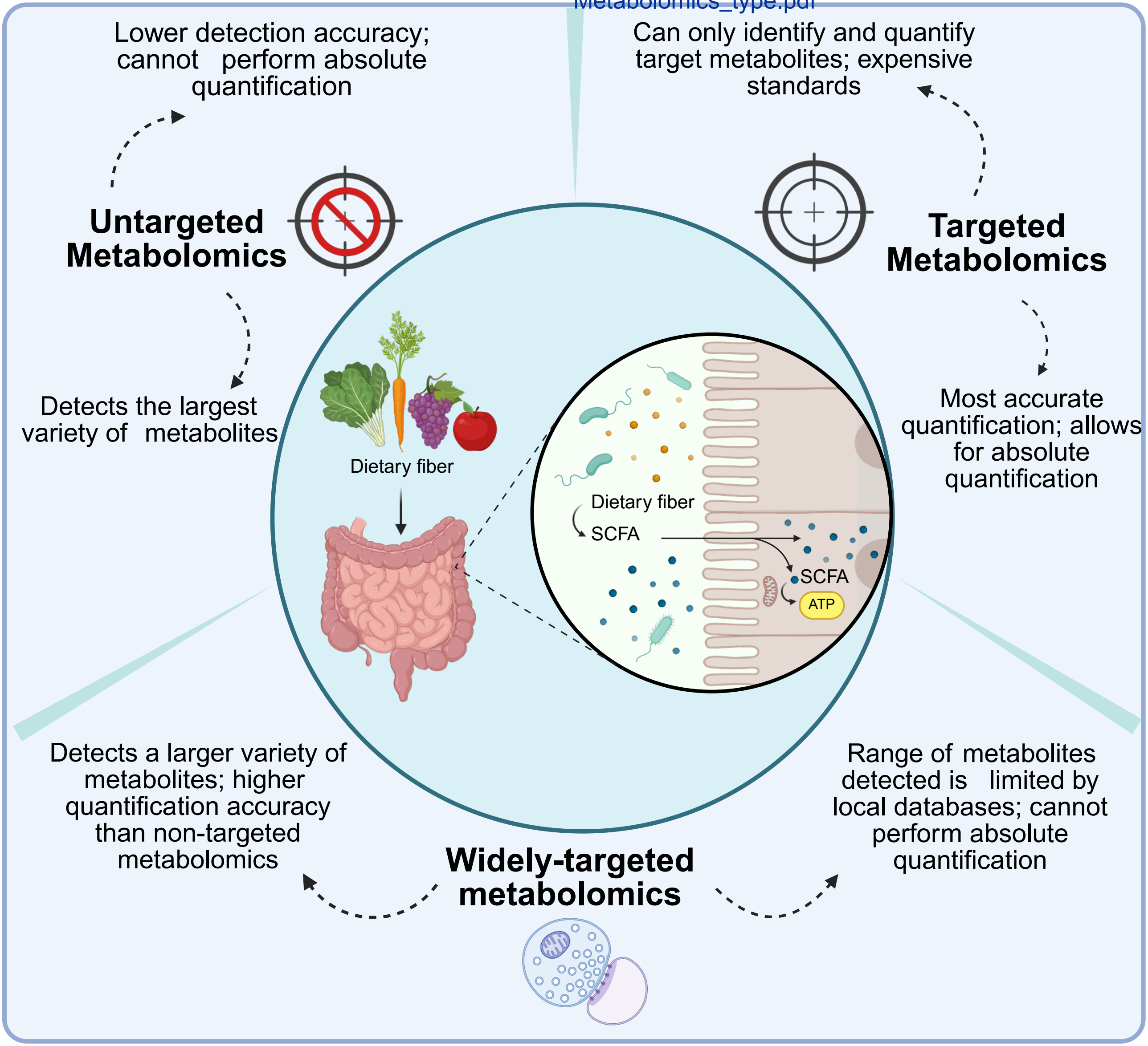

Figure2

Click here to access/download;Figure;Figure2-High-resolution_omics.pdf

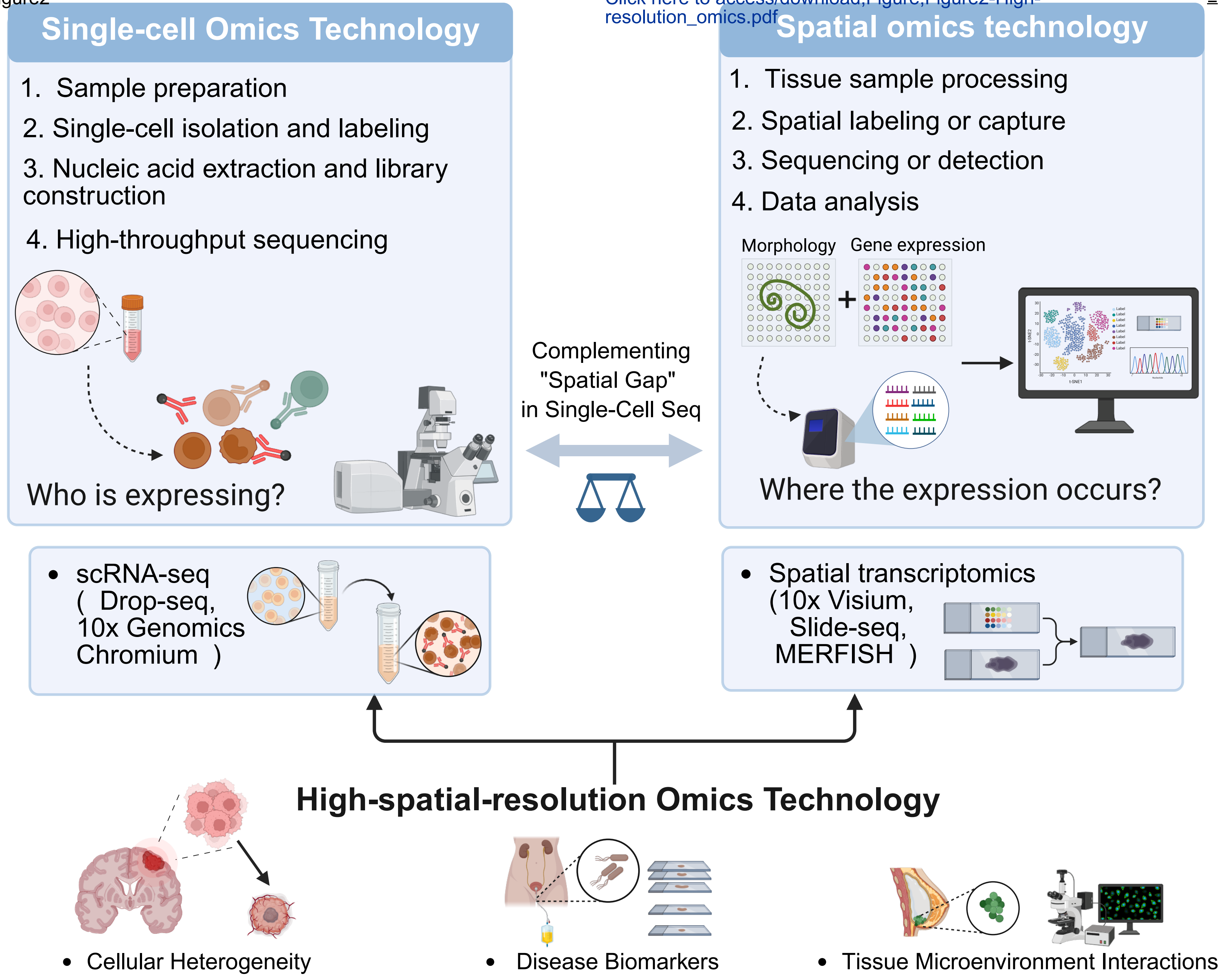

Figure3

Click here to access/download;Figure;Figure3-Muti_omics_in_nervous_system_diseases.pdf

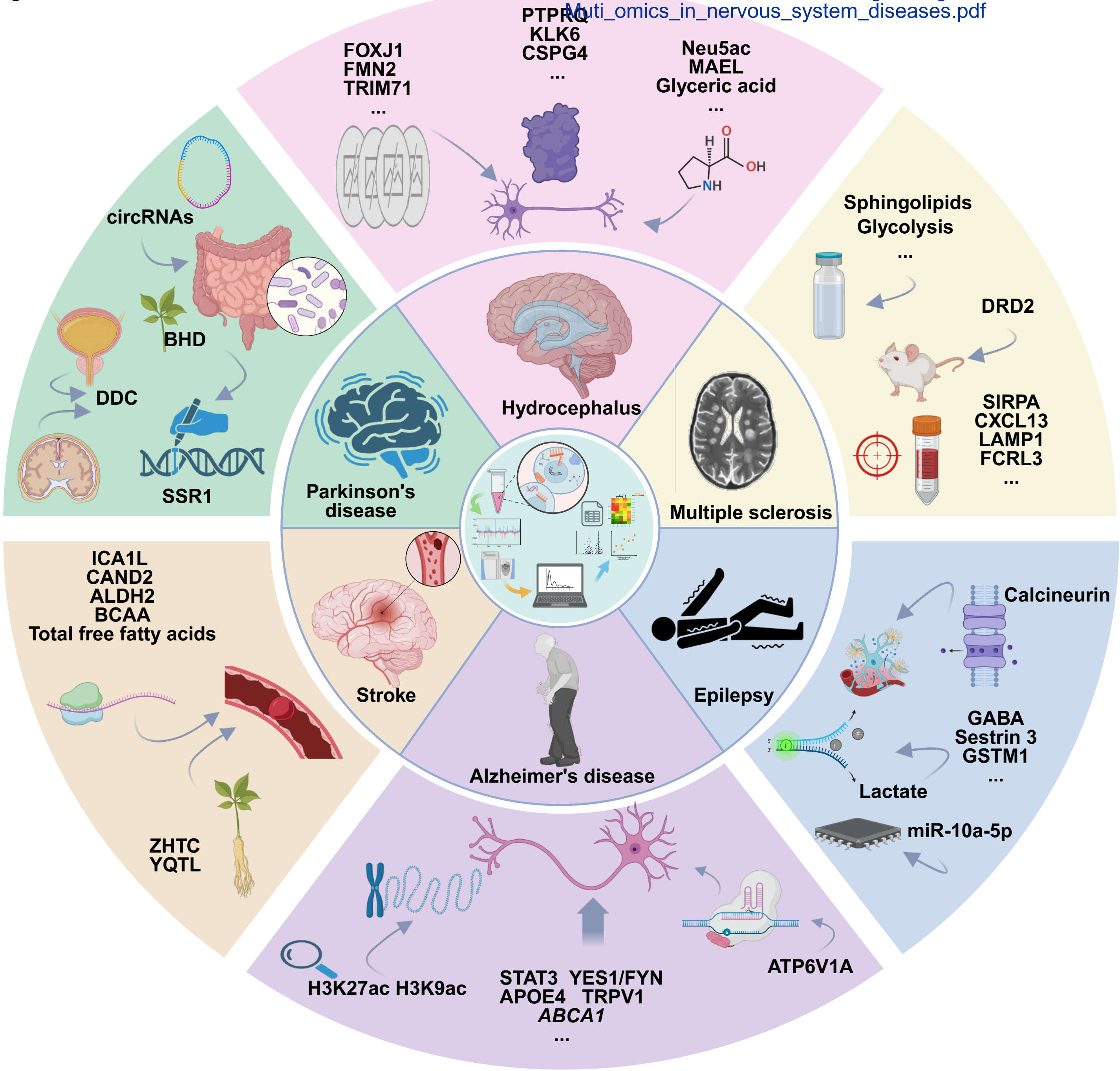

Figure4

Click here to access/download;Figure;Figure4-AD_mechanism.pdf

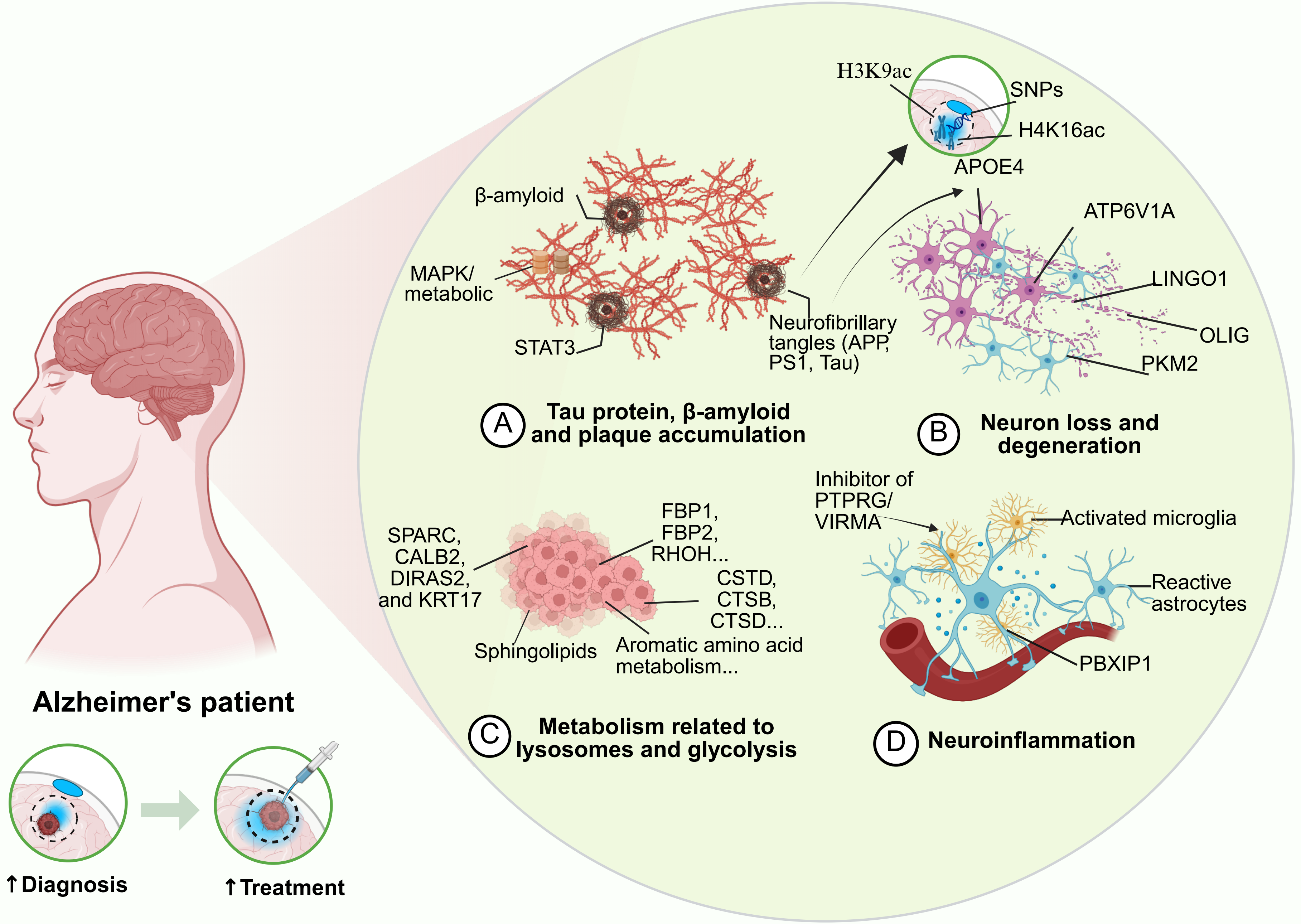

Figure5

Click here to access/download;Figure;Figure5-PD_mechanism.pdf

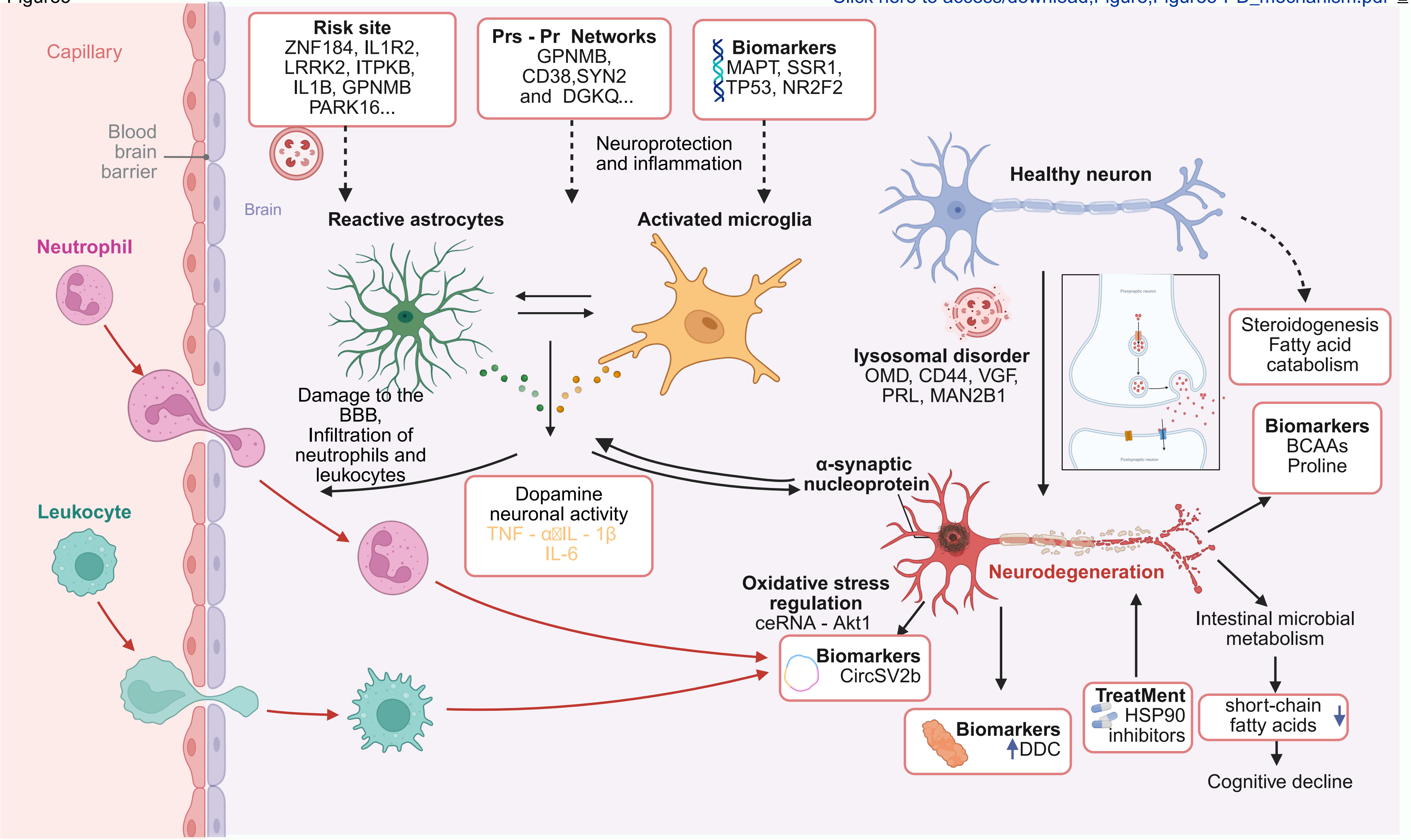

Personal Cover

Click here to access/download;Personal Cover;Cover_Letter.docx

Xiuyun Liu, Professor
Dean of School of Pharmaceutical Sciences and Technology
Tianjin University
Tianjin, China, 300072
Email: xiuyun_liu@tju.edu.cn
Tel: +86 13820594672

August 30th, 2025

Dear Editor-in-Chief,

We wish to submit an original review article entitled “Multi-Omics and High-Spatial-Resolution Omics: Deciphering Complexity in Neurological Disorders” for consideration in GigaScience.

Neurological diseases, which have become an increasingly significant global health challenge, are characterized by structural and functional abnormalities in the central and peripheral nervous systems, triggered by multiple factors such as genetic mutations, metabolic disturbances, and immune dysfunctions, and exhibit highly complex pathogenesis involving disruptions at various molecular levels, ranging from genetic variations and transcriptional alterations to protein dysfunctions and metabolic imbalances. The advent of high-throughput technologies, such as next-generation sequencing and mass spectrometry, has accelerated the development of several single-omics disciplines. These include genomics, which reveals the genetic basis of diseases by analyzing gene variations; transcriptomics, which explores dynamic gene regulatory networks through RNA expression profiling; proteomics, which systematically identifies protein composition and post-translational modifications using mass spectrometry; and metabolomics, which investigates metabolic responses under both physiological and pathological conditions through comprehensive metabolite profiling. However, single-omics technologies provide only fragmented insights, each focusing on a specific molecular dimension, and fail to capture the intricate and dynamic interactions between molecular layers, which are critical for understanding the complexity of diseases. In contrast, basic-omics and high-spatial-resolution (single-cell and spatial) integration technologies, which combine data from genomics, transcriptomics, proteomics, metabolomics, single-cell, and spatial omics, offer a more comprehensive framework for decoding the complexities of neurological diseases by enabling a deeper understanding of the molecular networks and pathophysiological mechanisms underlying neurodegenerative disorders and other neurological conditions. This review deeply examines the core principles of single-omics technologies and their applications in neurological disease research, highlighting multi-omics and high-spatial-resolution integration's technological advancements, biological significance, and clinical translation potential while addressing challenges like data integration complexities, standardization issues, and high computational demands in multi-omics approaches. Studies suggest multi-omics technologies hold substantial potential for transforming precision diagnostics, biomarker discovery, therapeutic target identification, and patient stratification in neurological diseases, while also offering innovative opportunities for cross-disciplinary fields like synthetic biology beyond advancing personalized treatment strategies.

We believe that this work aligns with the scope of the prestigious journal, GigaScience, given the journal's focus on the intersection of multiple omics technologies and integrative biological approaches. We confirm that this work is original and has not been published elsewhere, nor is currently under consideration for publication elsewhere. We have no conflicts of interest to disclose.

Thank you very much for considering our submission.

Sincerely,
Xiuyun

Xiuyun Liu, Professor
Dean of School of Pharmaceutical Sciences and Technology
Tianjin University
Tianjin, China, 300072
Email: xiuyun_liu@tju.edu.cn
Tel: +86 1382059467

Graphical Abstract

Click here to access/download;Graphical Abstract;Graphical_Abstract.docx

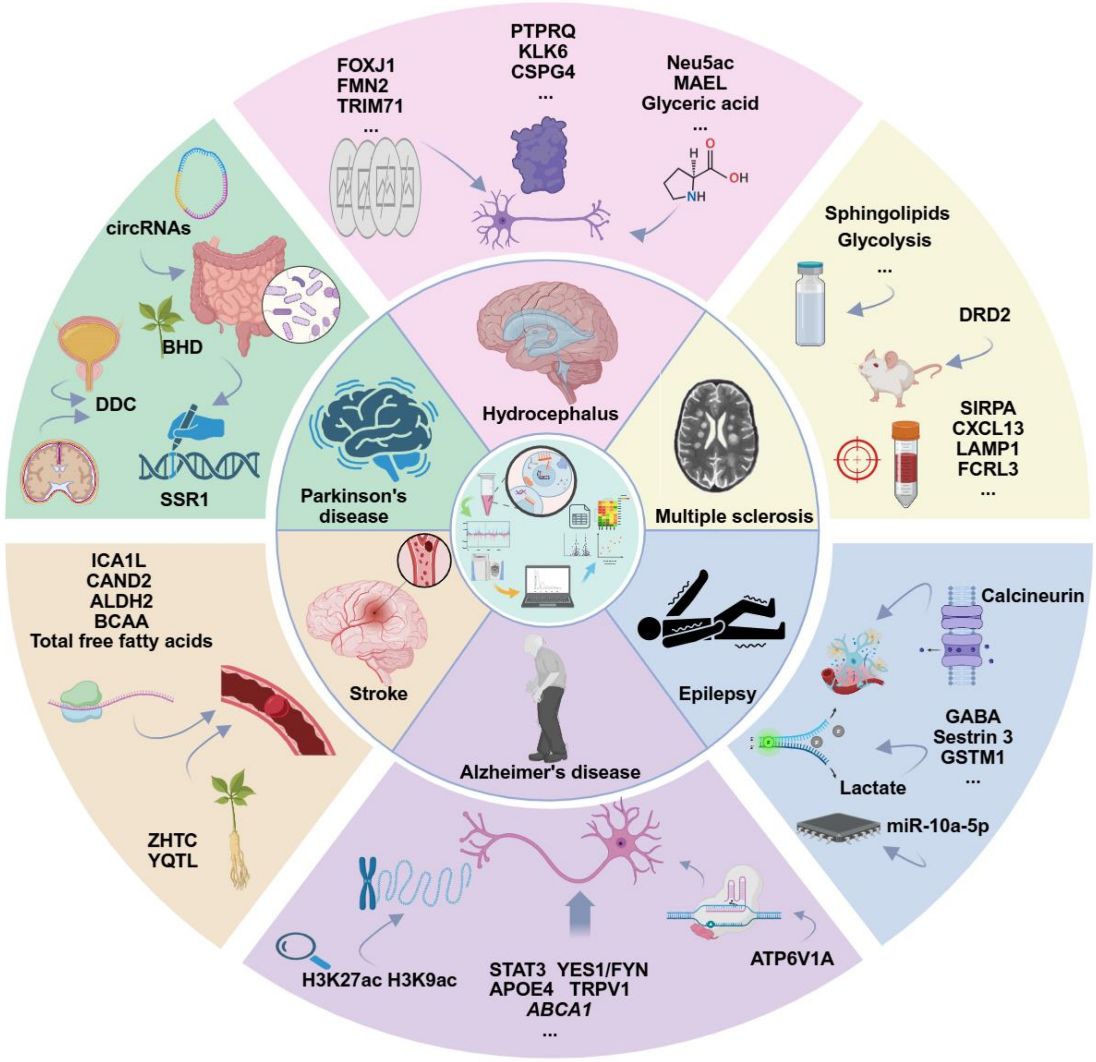

Applications of multi-omics and high-spatial-resolution omics technologies in the diagnosis and treatment of brain diseases in the field of neurology. By integrating high-throughput omics technologies, including four basic omics, single-cell, and spatial omics technologies, it is possible to dissect the complex pathogenic mechanisms of neurological disorders comprehensively. This multi-omics approach spans multiple levels, from genetic variations to metabolic changes, and reveals the interactions between these levels, providing an unprecedented perspective for in-depth disease understanding. In the study of diseases such as AD, PD, stroke, epilepsy, MS, and hydrocephalus, the application of these cutting-edge technologies has greatly facilitated the discovery of key biomarkers and significantly deepened our understanding of the molecular mechanisms of disease pathogenesis.
