# Supplementary material for: Multi-omics and high-spatial-resolution omics: deciphering complexity in neurological disorders
Source: Gigascience. 2025 Dec 5;14:giaf137. doi: 10.1093/gigascience/giaf137 (PMC12723665; doi:10.1093/gigascience/giaf137)
Supplement: giaf137_GIGA-D-25-00354_Revision_1 [file giaf137_giga-d-25-00354_revision_1.pdf]

GigaScience

Multi-Omics and High-Spatial-Resolution Omics: Deciphering Complexity in Neurological Disorders

--Manuscript Draft--

**Manuscript Number:**	GIGA-D-25-00354R1
**Full Title:**	Multi-Omics and High-Spatial-Resolution Omics: Deciphering Complexity in Neurological Disorders
**Article Type:**	Review
**Funding Information:**	Scientific Research Innovation Capability Support Project for Young Faculty (ZYGXQNJSKYCXNLZCXM-H15) — PhD Xiuyun Liu<br>National Science Fund for Excellent Overseas Scholars (0401260011) — PhD Xiuyun Liu<br>National Natural Science Foundation of China (82472098) — PhD Xiuyun Liu<br>National Natural Science Foundation of China (32300704) — PhD Xiuyun Liu<br>Tianjin Natural Science Foundation-Outstanding Youth Project (24JCJQJC00250) — PhD Xiuyun Liu<br>Major Science and Technology Special Projects and Engineering-Major Project of National Key Laboratories (24ZXZSSS00510) — PhD Xiuyun Liu<br>Key Technologies Research and Development Program (2021YFF1200602) — PhD Xiuyun Liu<br>the Non-profit Central Research Institute Fund of Chinese Academy of Medical Sciences (2024-JKCS-16) — PhD Xiuyun Liu
**Abstract:**	The world has witnessed a steady rise in neurological diseases, which represent a heterogeneous group of disorders characterized by complex pathogenesis involving disruptions at multiple molecular levels, including genomic, transcriptomic, proteomic, and metabolomic levels. These disorders, often caused by genetic mutations, metabolic imbalances, immune dysregulation, and environmental factors, pose significant challenges to global public health due to their high prevalence, mortality, and disability burden. The advent of high-throughput technologies, such as next-generation sequencing and mass spectrometry, has provided valuable insights into the underlying mechanisms of disease, especially the development of multi- and high-spatial-resolution omics technologies, enabling the interaction of multiple levels of biology and analysis of the complex molecular networks and pathophysiological processes. This review provides a comprehensive analysis of the latest advancements in multi- and high-spatial-resolution omics, with a focus on their applications in precision diagnostics, biomarker discovery, and therapeutic target identification in brain diseases. The study also highlights the current challenges in the clinical implementation and discusses the future directions, with artificial intelligence being anticipated to enhance clinical translation and diagnostic accuracy significantly.
**Corresponding Author:**	Xiuyun Liu, Ph.D<br>Tianjin University<br>Tianjin, Tianjin CHINA
**Corresponding Author Secondary Information:**	
**Corresponding Author's Institution:**	Tianjin University
**Corresponding Author's Secondary**	

Powered by Editorial Manager® and ProduXion Manager® from Aries Systems Corporation

**Institution:**	
**First Author:**	Xiuyun Liu, PhD
**First Author Secondary Information:**	
**Order of Authors:**	Xiuyun Liu, PhD
	Fangfang Li, PhD
	Marek Czosnyka, PhD
	Zofia Czosnyka, PhD
	Huijie Yu, PhD
	Xiaoguang Tong, PhD
	Yan Xing, PhD
	Hongliang Li, PhD
	Ke Pu, PhD
	Keke Feng, PhD
	Kuo Zhang, PhD
	Meijun Pang, PhD
	Dong Ming, PhD
**Order of Authors Secondary Information:**	
**Response to Reviewers:**	Dear editors and all the reviewers,<br>Thank you very much for your comments and feedback. We do appreciate your valuable time reviewing this manuscript, as well as your consideration of our work. We have carefully read through the comments on our manuscript and would like to express our sincere gratitude for the insightful and helpful comments. We have thoroughly revised our manuscript and we hope the revised version meets the requirements of the journal for publication.<br>In summary, we have revised the opening section of each disease to enhance reader engagement, structuring the content around the core challenges of the disease and how multi-omics technologies address these issues and drive advancements. We have also supplemented the conclusion section of each disease with specific descriptions of current challenges and future research directions—through a review of each disease, we discuss the limitations of existing technologies and the key priorities for overcoming disease-related hurdles. Additionally, we have labeled the experimental verifiability of each disease in all tables throughout the manuscript, specifying the validation type (e.g., human-based, animal model-based, or validated using multiple independent datasets). Furthermore, we have integrated the latest multi-omics dataset findings for each disease in a bid to render the review more comprehensive and up-to-date. Please find our detailed responses to your comments below. Finally, we have also conducted a comprehensive review of all gene and protein names throughout the manuscript to ensure their compliance with relevant nomenclature standards.<br>Once again, thank you for your time and consideration. We look forward to your feedback on the revised manuscript.<br><br>Sincerely,<br>Xiuyun Liu<br><br>Authors' Responses to Reviewers:<br>Reviewer #1:<br>Minor concerns:<br>1. It would be helpful to clearly distinguish between findings that remain at the omics-data level and those that have been experimentally validated. Omics analyses often reveal correlations rather than causality. Among the cases cited, which rely solely on data-driven evidence and which are supported by experimental validation should be explicitly specified.<br><br>Response 1. Thank you for your suggestion. We fully agree with your point that clearly

distinguishing between omics-data-level findings and experimentally validated results is crucial for enhancing the rigor and clarity of our research. Following the reviewer's suggestion, an extra column has been added to the summary table for each disease, detailing whether the experiment was validated and specifying the validation type, including independent cohort-based validation, animal model-based validation, and validation using multiple independent datasets. Please refer to Table for Supplementary Table S1-S6, Page 71-106.

2. The current structure places heavy emphasis on uniform technical descriptions, with each section organized primarily around methodological perspectives. This makes the review somewhat monotonous. A more engaging approach would be to start each section by posing a key scientific question in the context of brain disorders, discuss the challenges in addressing it without omics, and then explain how omics technologies have advanced understanding of the problem. Such a structure would improve the narrative and reader engagement.

Response 2. We totally agree with the review and thank you for your suggestion. Furthermore, the framework you proposed, which involves opening each section with key scientific questions in the context of brain disorders, discussing the challenges inherent in addressing such questions without omics technologies, and subsequently elaborating on how omics approaches have advanced our understanding of these issues, provides a clear and invaluable direction for optimizing the narrative logic of our work and enhancing reader engagement. We have added and modified the corresponding content before describing the histological techniques for each disease section.
Please refer to Line 175-193, Paragraph 2-3, Page 12.
The field of AD intervention has long been confronted with two major challenges: first, the pronounced delay in early diagnosis—owing to its insidious onset, most patients are identified only at moderate to severe stages when pathological progression has become largely irreversible[59], resulting in a critical missed window for intervention; second, the incomplete understanding of its pathogenetic mechanisms—although core pathological features such as amyloid plaques and tau neurofibrillary tangles have been identified[52,54], the molecular regulatory networks underlying subsequent neuronal injury, neuroinflammation, and gut microbiome dysbiosis remain poorly elucidated[55]. Prior to the emergence of multi-omics technologies, these challenges were compounded by methodological limitations. Conventional hypothesis-driven approaches largely focused on isolated pathways such as Aβ deposition or tau phosphorylation[54]. While valuable for elucidating specific pathological components, such strategies failed to capture system-level interactions among gene expression, protein networks, and metabolic alterations. This constrained the discovery of early specific biomarkers and hindered the development of integrated therapeutic strategies targeting multiple pathological processes.
The advent of multi-omics approaches has provided a pivotal toolkit to overcome these limitations[60]. This global perspective transcends the constraints of single-pathway investigations and marks a shift from compartmentalized pathological observation towards systematic mechanistic decoding. It provides a robust foundation for the identification of early intervention targets and the development of precision therapeutics, heralding a new era in AD research and clinical management.

Please refer to Line 397-408, Paragraph 3, Page 21-22.
A central unresolved question in PD research is how to accurately define disease subtypes and identify early molecular events prior to overt neurodegeneration. Without multi-omics approaches, studies were often limited to clinical phenotypes and single molecular layers, failing to capture the systemic interactions among genetic variants, transcriptional dysregulation, protein misfolding, and metabolic dysfunction. This narrow view hindered the discovery of predictive biomarkers and subtype-specific therapeutic targets, particularly at pre-symptomatic stages. The application of integrated genomics, transcriptomics, proteomics, and metabolomics has transformed this paradigm. These technologies enable high-resolution mapping of endophenotypes and dynamic pathways, revealing novel biomarkers such as GPNMB and DDC, and unmasking critical mechanisms like neuroinflammation, oxidative stress, and mitochondrial impairment. Multi-omics thus provides a holistic, mechanistic framework for early diagnosis, patient stratification, and targeted interventions in PD.

Powered by Editorial Manager® and ProduXion Manager® from Aries Systems Corporation

Please refer to Line 519-526, Paragraph 2, Page 27.
A fundamental challenge in epilepsy research is how to decipher the complex, multi-dimensional interactions among genetic predisposition, neuronal excitability, inflammatory processes, and metabolic dysregulation that collectively contribute to seizure generation and progression. Prior to the advent of multi-omics technologies, research was often siloed into isolated domains—electrophysiology, candidate genes, or histopathology—which provided fragmentary insights but failed to capture the systemic and dynamic nature of epileptogenesis. This limitation hindered the identification of convergent pathways and translatable biomarkers, especially across different epilepsy subtypes and developmental stages.

Please refer to Line 652-662, Paragraph 1, Page 33.
A major challenge in MS research is understanding how immune, inflammatory, and degenerative processes interact across disease stages. Traditional methods offered limited insight into system-wide changes, hindering biomarker discovery and targeted therapy development. The complex and heterogeneous mechanisms of MS remain incompletely understood, impeding targeted therapeutic development. Multi-omics technologies offer a comprehensive approach to uncover molecular underpinnings and identify novel biomarkers and therapeutic targets.Multi-omics approaches have revolutionized MS research by integrating spatial, single-cell, and molecular data. These technologies reveal key cell subtypes, lesion-specific biomarkers, and critical pathways such as APOE-TREM2 and CXCL12-CXCR4. These advances are paving the way for improved stratification mechanisms and precision medicine interventions in MS.

Please refer to Line 756-763, Paragraph 2, Page 38.
A major challenge in stroke research is grasping the spatiotemporal dynamics of molecular and cellular responses to acute injury and recovery. Before multi-omics, studies were confined to single pathways or cell types, missing system-wide interactions across key networks and hindering the discovery of reliable biomarkers and neuroprotective strategies, especially early post-stroke. Multi-omics have revolutionized this by enabling high-resolution, integrated profiling of transcriptional, proteomic, and metabolic changes in a cell- and region-specific way. This has revealed key mechanisms like microglia-endothelial crosstalk and immune-metabolic axes (e.g., SPP1-CD44), and identified novel biomarkers and targets (e.g., LILRB4, LGALS9) for stroke treatment.

Please refer to Line 878-884, Paragraph 2, Page 43.
Multi-omics technologies integrate genomic, proteomic, and metabolomic data to systematically dissect molecular networks underlying hydrocephalus, enabling the discovery of novel biomarkers such as CSPG4 to improve early diagnosis and dynamic monitoring of disease progression. These approaches further uncover central pathophysiological mechanisms—including choroid plexus inflammation and ciliary dysfunction—and identify potential therapeutic targets within dysregulated signaling pathways (e.g., TGF-β, VEGF) and metabolic perturbations, thereby providing new avenues for developing precision interventions.

3. While the review provides a good summary of past progress, it pays relatively little attention to open questions in the field and potential avenues for future solutions. Beyond summarizing existing achievements, a strong review should also highlight unresolved challenges (both in neurological disease research and in the development and application of omics technologies) and discuss how emerging omics strategies might address them. This aspect should be strengthened.

Response 3. We are deeply grateful for your perceptive comment. In the revised manuscript, we have added a dedicated section at the end of each disease chapter—including stroke, epilepsy, AD, and others—outlining the major unresolved challenges and future directions for omics research. In addition, we describe in detail the common set of challenges facing neurological disorders and the future direction of multi-omics technology in the Discussion section.
Please refer to Line 368-376, Paragraph 4, Page 20.
Despite advancements in multi-omics technologies, AD research remains heavily reliant on classic biomarkers—Aβ and tau, which continue to dominate both mechanistic and clinical frameworks. While omics approaches have identified

numerous candidate biomarkers and pathways, few have been rigorously validated in large, diverse cohorts or translated into clinically usable tools. A major persistent challenge is the fragmented integration of multi-omics data: genomic, proteomic, and metabolomic layers are often studied in isolation rather than as interconnected networks. This lack of systematic integration limits our ability to uncover actionable biological mechanisms or subtype-specific signatures, hindering the development of personalized diagnostic and therapeutic strategies.

Please refer to Line 492-504, Paragraph 2, Page 26.
Despite advances in multi-omics research, the field of PD continues to grapple with three fundamental challenges: clinical heterogeneity (such as differential treatment responses between tremor-dominant and postural instability/gait difficulty subtypes), poorly understood mechanisms of α-synuclein propagation (particularly via proposed gut-to-brain pathways through the vagus nerve), and a persistent lack of disease-modifying therapies. Future studies should prioritize deeply phenotyped longitudinal cohorts—incorporating real-time motor monitoring via wearable sensors and comprehensive autonomic function assessments—alongside the integration of single-cell sequencing and spatial transcriptomics to elucidate region-specific neurodegeneration in the substantia nigra. The development of advanced computational models (e.g., multimodal data fusion and Mendelian randomization) will be essential to infer causal pathways from high-dimensional data. By focusing on prodromal cohorts and currently underrepresented populations, researchers can systematically unravel neuroimmune-metabolic cross-talk, ultimately enabling the discovery of early biomarkers, patient stratification, and personalized therapeutic strategies.

Please refer to Line 628-638, Paragraph 2, Page 32.
The pathogenesis of epilepsy is highly complex, involving multilayered biological processes. Different types of epilepsy may exhibit distinct etiologies and mechanisms, complicating both research and therapeutic development. Furthermore, significant clinical heterogeneity among patients adds another layer of difficulty to studying the disease. Multi-omics studies in epilepsy generate vast amounts of data across genomic, transcriptomic, epigenomic, and other molecular levels. These insights extend beyond pathogenesis to offer novel diagnostic biomarkers—like miR-10a-5p and ALDH2—and reveal new therapeutic targets, exemplifying how multi-omics bridges mechanistic discovery and precision medicine in epilepsy. Integrating and analyzing these datasets require high-performance computing resources as well as systematic and statistically robust analytical frameworks and mathematical models. A major ongoing challenge lies in the development of efficient computational strategies to process and interpret these complex, high-dimensional data.

Please refer to Line 729-740 Paragraph 3, Page 36-37.
Multi-omics technologies have systematically uncovered key mechanisms in MS, including dysregulated $CD4^+$ T cell–B cell crosstalk, impaired TREM2-DAP12 signaling in microglia, and disrupted differentiation of oligodendrocyte precursor cells. While potential therapeutic targets such as SEMA4D and CXCL13 have been identified, their functional roles in remyelination and immune infiltration require further validation through in vivo gene editing and organoid models. Critical challenges remain in predicting the transition from relapsing-remitting to secondary progressive MS, deciphering compartmentalized inflammation within chronic lesions, and elucidating mechanisms of resistance to B cell-targeted therapies. Future efforts should prioritize longitudinal multi-omic profiling of well-annotated conversion-phase cohorts, integration of digital pathology with real-time wearable sensor data, and application of artificial intelligence—such as graph neural networks and multimodal learning—to uncover clinically actionable biomarkers and enable precision medicine interventions.

Please refer to Line 849-859, Paragraph 2, Page 42.
While multi-omics technologies hold promise for advancing precision medicine in stroke, addressing critical challenges—including pronounced disease heterogeneity, complex repair mechanisms, and difficulties in translating omic findings into clinical applications—remains essential to fully realize their potential. It is crucial to validate biomarkers and therapeutic targets across diverse stroke populations and to develop targeted delivery systems for neuroprotective agents. However, current stroke multi-omics datasets remain limited in scale and diversity, and integrating multimodal data is

complicated by missing values, batch effects, and lack of standardization. Future research should prioritize longitudinal multi-omics profiling to capture dynamic disease progression, incorporate single-cell and spatial technologies to resolve cell- and region-specific responses, and leverage artificial intelligence for integrated analysis of multimodal data.

Please refer to Line 943-951, Paragraph 2, Page 46.
However, multi-omics studies in hydrocephalus face considerable challenges due to the relative difficulty in obtaining CSF samples, which are often limited in volume. Furthermore, the low abundance of proteins and metabolites in CSF necessitates highly sensitive detection technologies. Preanalytical variables during sample collection, processing, and storage can also significantly affect data accuracy and reproducibility. Critically, the scarcity of these precious samples has resulted in a notable paucity of high-quality omics datasets in hydrocephalus, making existing data exceptionally valuable. Overcoming these barriers will require intensified research efforts, standardized protocols, and enhanced support from funding agencies and policymakers to facilitate larger-scale sample acquisition and multi-institutional collaboration.

Reviewer #2
Major concerns:
1. Overall, this manuscript is thorough. However, to enhance its impact and ensure the review remains current and informative, the authors are encouraged to incorporate more up-to-date and comprehensive datasets in their analysis. Several recent published AD snucRNAseq datasets, e.g., PMIDs: 31042697 and 39198642, and the PsychAD project should be reviewed and appropriately cited.

Response 1. We sincerely thank the reviewer for this helpful suggestion. We have now incorporated and discussed these recommended snucRNA-seq datasets, including the PsychAD project, to enhance the timeliness and comprehensiveness of our review.
Please refer to Line 252-254, Paragraph 2, Page 15.
The experiment also revealed gender differences that exist in AD, including transcriptional response differences and the overrepresentation of females in AD-associated cell subpopulations.

Please refer to Line 275-282, Paragraph 3, Page 16.
Using single-nucleus RNA sequencing (snRNA-seq) and spatial transcriptomic data, one study constructed a comprehensive brain cell atlas comprising 1.65 million single-nucleus transcriptomes from 437 aged individuals. It identified glial and neuronal subpopulations associated with AD, and employed causal modeling to highlight lipid-associated microglial subsets—which drive Aβ pathology or mediate Aβ-TAU crosstalk—alongside TAU-responsive astrocytic populations. These findings provide novel cellular insights into AD pathogenesis and suggest promising therapeutic targets. Furthermore, the identification of distinct brain aging trajectories may facilitate early diagnosis and personalized treatment strategies[78].

2. In addition, for epigenomic datasets, including recent ATAC-seq datasets (e.g., PMIDs 35931864 and 37774680), should also be considered, as they offer important insights into chromatin accessibility in the AD brain.

Response 2. We greatly appreciate this insightful comment. Following the reviewer's recommendation, we have included discussion of recent ATAC-seq datasets reflecting chromatin accessibility changes in AD.
Please refer to Line 283-296, Paragraph 4, Page 16-17.
Recent multi-omics studies on human primary microglia have elucidated how genetic regulation of chromatin accessibility influences transcriptional programs. Integrative transcriptomic and epigenomic analyses nominated SPI1/PU.1 as a key transcriptional regulator of microglial gene expression and AD risk, with reduced PU.1 binding destabilizing chromatin accessibility. These findings reinforce the central role of SPI1/PU.1 in microglial dysfunction in AD, while also identifying additional contributing transcription factors[79]. Through single-cell epigenomics and transcriptomics analysis of the prefrontal cortex, the study by Chen et al. constructed a cell-type-resolved regulome atlas of the human brain, revealing significant enrichment of AD risk loci in

microglial enhancers and binding sites of transcription factors including SPI1, ELF2, and RUNX1. Previous research identified 9,628 cell-type-specific ATAC-QTLs and integrated these with peak-to-gene linkages to decipher variant-regulatory circuits in AD. Furthermore, the experiment uncovered differential accessibility of regulatory modules in glial cells during late-stage AD and in neurons at early stages, with advanced AD exhibiting global epigenomic dysregulation, suggesting widespread epigenomic erosion and loss of cellular identity[80].

3. Multiome datasets that integrate transcriptomic and epigenomic layers (e.g., PMID 39402379IF: 20.0 Q1) are particularly relevant and would strengthen the manuscript's discussion of integrative analyses.

Response 3. We thank the reviewer for highlighting the importance of multiomic integration. Accordingly, we have added content related to multiome datasets (such as the cited study) that simultaneously profile transcriptomic and epigenomic layers, strengthening our discussion of integrated omics analyses.
Please refer to Line 297-303, Paragraph 2, Page 17.
One recent study employed single-cell epigenomics and spatial genomics to characterize AD cell populations, defining two disease stages: an early phase featuring slow pathological buildup with inflammatory microglia, reactive astrocytes, loss of SST^+ inhibitory neurons, and remyelination; and a late phase marked by exponential pathology increase and loss of excitatory and inhibitory ($Pvalb^+/Vip^+$) neurons. These findings were validated in independent AD cohorts, and the systematic application of multi-omics technologies coupled with quantitative neuropathology enables effective modeling of disease progression across the spectrum of AD severity[81].

4. This suggestion is not limited to AD; for each neurological disorder reviewed, the authors should aim to incorporate the most recent omics datasets and studies to reflect the current state of the field.
Response 4. Thank you for your suggestion regarding the dataset. We have systematically updated the omics datasets and incorporated the most recent literature across all neurological disorders covered in this review, including AD, PD, stroke, MS, epilepsy, and hydrocephalus. These additions ensure a more comprehensive and up-to-date representation of the latest research advances. We have also supplemented the relevant figures and tables accordingly to provide a thorough and systematic overview of the most current progress in each disease area.
Please refer to Line 304-310, Paragraph 3, Page 17.
Through snRNA-seq combined with spatially resolved transcriptomics, Maria et al. demonstrated that compared to sporadic AD, autosomal dominant AD cases exhibit pronounced upregulation of autophagy-related and chaperone genes. Spatial transcriptomic analysis further confirmed the specific activation of chaperone-mediated autophagy pathways in PSEN1-E280A carriers. In autosomal dominant cases, they observed astrocytic overexpression of LRP1, elevated FKBP1B expression, and reduced neuronal PSEN1 levels, which may collectively underlie a protective mechanism against AD pathology[82].

Please refer to Line 533-556, Paragraph 2, Page 28-29.
By integrating analyses of gray matter volume with transcriptomic data from the human brain atlas, one study revealed that patients with focal to bilateral tonic–clonic seizures (FBTCS+) exhibit more extensive bilateral cortical and subcortical morphological alterations compared to the non-FBTCS group (FBTCS−). Both groups were involved in neurodevelopmental and synaptic-related pathways. Cell-type mapping demonstrated that both excitatory and inhibitory neurons were affected in the FBTCS− group, whereas only excitatory neurons showed significant changes in the FBTCS+ group, suggesting that excitatory/inhibitory imbalance and abnormalities in the GABAergic system may constitute a key mechanism underlying FBTCS[124]. Through the establishment of a GABRA4 knockout ($GABRA4^{-/-}$) mouse model, the study found that the mice exhibit core behavioral phenotypes resembling high-functioning autism, including social deficits, enhanced spatial memory, and reduced susceptibility to seizures. Subsequent hippocampal transcriptomic and interactome analyses revealed that the NMDAR signaling pathway plays a central regulatory role in mediating these autism-like behaviors and anti-epileptic phenotypes, indicating a functional convergence between the GABAergic system and NMDA receptor pathways in the pathogenesis of autism. Gabra4 knockout mice exhibited significantly decreased

Powered by Editorial Manager® and ProduXion Manager® from Aries Systems Corporation

susceptibility to pentylenetetrazol-induced seizures, indicating a protective effect against epileptic activity[125]. By integrating [18F]SynVesT-1 PET synaptic imaging and transcriptomic data, research revealed reorganization in the synaptic density similarity network in TLE, characterized by reduced connectivity, decreased clustering, and increased path length, particularly within temporolimbic and frontoparietal circuits. These network alterations correlated spatially with TLE risk gene expression and identified 183 downregulated synaptic transmission-related genes, including core GABAergic genes such as RBFOX1. The findings suggest that coordinated downregulation of risk genes may contribute to synaptic network dysfunction and epileptogenesis in TLE, offering new directions for molecular and genetic network-targeted therapies[126].

Please refer to Line 657-664, Paragraph 2, Page 33-34.
A study involving over 20,000 MS patients has definitively established cells of the central nervous system—particularly inhibitory neurons—as pivotal cellular contributors to MS susceptibility. Specific risk variants, such as those influencing the STAT3 and IL7 genes, were found to affect gene expression exclusively within inhibitory neurons, revealing that cell-intrinsic dysregulation of immune-related pathways within neurons directly contributes to MS pathogenesis. These findings imply that future preventive and therapeutic strategies must incorporate interventions targeting CNS-specific pathways, including neuronal and glial functions, moving beyond solely immunosuppressive approaches[146].

Please refer to Line 765-773, Paragraph 3, Page 38-39.
Comprehensive RNA-sequencing analysis revealed that transcriptional responses to ischemic stroke occur in a similar direction but with markedly different magnitude in aged compared to young mouse brains. Specifically, aged mice exhibited downregulation of genetic programs associated with axonal and synaptic integrity, coupled with significant upregulation of the type I interferon (IFN-I) signaling pathway. This enhanced IFN-I response was primarily driven by microglia and oligodendrocytes. The increased vulnerability to stroke in aged brains was largely attributable to excessive activation of pro-inflammatory pathways rather than a failure of neurooprotective mechanisms[169].

Please refer to Line 816-827, Paragraph 2, Page 441.
A study utilizing spatial transcriptomics and GWAS compared different regions along the hemodynamic direction of human carotid artery plaques and found that plaque rupture primarily occurs in the proximal and most stenotic areas. Key genes such as MMP-9 were identified to have a causal relationship with rupture risk. The findings provide a theoretical basis for precise interventions targeting MMP-9 and other relevant targets in stroke patients[179]. A recent study integrating high-resolution omics technologies—including single-cell transcriptomics, proteomics, and spatial transcriptomics—revealed that proximal astrocytes exhibit divergence in lipid handling, with enriched expression of APOE and FABP5 following cortical ischemic stroke. The data reported here demonstrate the acquisition of distinct astrocyte states post-stroke, governed by both temporal and spatial proximity to the lesion, while demonstrating the utility of a high-content single-cell tool termed tDISCO, which identified both the spatial boundaries and molecular profiles defining these distinct populations[180].

Please refer to Line 910-917, Paragraph 2, Page 45.
By integrating genomic and scRNA-seq approaches, a study systematically characterizes the evolutionary, temporal, and spatial expression profiles of maelstrom spermatogenic transposon silencer (MAEL) in the developing human brain, and functionally validates its expression in human hydrocephalic brain tissue. scRNA-seq analyses of the cortical plate and germinal matrix regions identified robust MAEL expression within neurogenic niches, wherein reduced MAEL expression may induce large-scale genomic rearrangements that disrupt cortical development, volume, and function. These findings suggest that diminished MAEL expression may contribute to the pathogenesis of hydrocephalus via multiple etiological pathways[198].

Minor comments
1. Gene names should be italicized throughout the manuscript to adhere to standard formatting conventions.

	Response 1. Thank you for pointing out the issue related to genetic nomenclature. We have carefully revised the formatting of all gene and protein symbols in the manuscript to meet the standard nomenclature requirements, including the proper italicization of gene symbols. All corresponding changes are clearly marked in the original document. Please refer to the highlighted parts throughout the manuscript for specific revisions.
Additional Information:	
Question	Response
Are you submitting this manuscript to a special series or article collection?	No
**Experimental design and statistics**<br><br>Full details of the experimental design and statistical methods used should be given in the Methods section, as detailed in our Minimum Standards Reporting Checklist. Information essential to interpreting the data presented should be made available in the figure legends.<br><br>Have you included all the information requested in your manuscript?	No
If not, please give reasons for any omissions below.<br><br>as follow-up to "**Experimental design and statistics**<br><br>Full details of the experimental design and statistical methods used should be given in the Methods section, as detailed in our Minimum Standards Reporting Checklist. Information essential to interpreting the data presented should be made available in the figure legends.<br><br>Have you included all the information requested in your manuscript?<br><br>"	This is a review paper
**Resources**<br><br>A description of all resources used, including antibodies, cell lines, animals and software tools, with enough	No

information to allow them to be uniquely identified, should be included in the Methods section. Authors are strongly encouraged to cite Research Resource Identifiers (RRIDs) for antibodies, model organisms and tools, where possible.<br><br>Have you included the information requested as detailed in our Minimum Standards Reporting Checklist?	
If not, please give reasons for any omissions below.<br><br>as follow-up to "**Resources**<br><br>A description of all resources used, including antibodies, cell lines, animals and software tools, with enough information to allow them to be uniquely identified, should be included in the Methods section. Authors are strongly encouraged to cite Research Resource Identifiers (RRIDs) for antibodies, model organisms and tools, where possible.<br><br>Have you included the information requested as detailed in our Minimum Standards Reporting Checklist?<br><br>"	This is a review paper, summarizing previous publication results
**Availability of data and materials**<br><br>All datasets and code on which the conclusions of the paper rely must be either included in your submission or deposited in publicly available repositories (where available and ethically appropriate), referencing such data using a unique identifier in the references and in the "Availability of Data and Materials" section of your manuscript.<br><br>Have you have met the above requirement as detailed in our Minimum	Yes

Standards Reporting Checklist?	
GigaScience has policies and guidelines in place for the use of generative AI-writing tools such as ChatGPT. If you have used such writing tools to assist with writing the manuscript this must be declared and cited in the text. Authors should not list AI-writing tools and other AI-assisted technologies as an author or co-author and should acknowledge that they are fully responsible for text generated or refined by AI-writing tools.<p><br><br>A summary of use (particularly in the introduction or among methods) needs to be included at the end of the paper, and the outputs should also be included as a supplementary file hosted in GigaDB or other open repositories. Please read our guidelines for more information. <p><br><br>By submitting to GigaScience, you are aware of the journal's AI-writing tools policy, and if you have declared use of such tools below, you have acknowledged this where appropriate in your manuscript and have made a summary of use and outputs available. <p><br><b>AI-assisted writing tools have been used in the preparation of this manuscript?	No

Manuscript Click here to access/download;Manuscript;Manuscript.docx

Multi-Omics and High-Spatial-Resolution Omics: Deciphering Complexity in Neurological Disorders

Xiuyun Liu, PhD [1,2,3#], Fangfang Li, MD [1#], Marek Czosnyka, PhD [4], Zofia Czosnyka, PhD [4], Huijie Yu, PhD [5], Xiaoguang Tong, PhD [6], Yan Xing, PhD [7], Hongliang Li, PhD [7], Ke Pu, PhD [6], Keke Feng, PhD [6], Kuo Zhang, PhD[1,2], Meijun Pang, PhD [1,2*], and Dong Ming, PhD [1,2*]

[1] State Key Laboratory of Advanced Medical Materials and Devices, Medical School, Tianjin University, Tianjin, 300072, China.

[2] Haihe Laboratory of Brain-Computer Interaction and Human-Machine Integration, Tianjin, 300380, China.

[3] School of Pharmaceutical Science and Technology, Tianjin University, Tianjin, 300072, China.

[4] Department of Clinical Neurosciences, Addenbrooke's Hospital, University of Cambridge, Cambridge, CB2 0QQ, UK.

[5] Department of Neurosurgery, Tianjin Medical University General Hospital, Tianjin, 300052, China.

[6] Department of Neurosurgery, Tianjin Huanhu Hospital, Tianjin, 300350, China.

[7] Department of Neurology, Aviation General Hospital, 100012, Beijing, China.

[#] These authors contributed equally to this work.

* Corresponding authors:

Meijun Pang, State Key Laboratory of Advanced Medical Materials and Devices, Medical School, Tianjin University, 92 Weijin Road, Nankai District, Tianjin, 300072, China, Email: meijun.pang@tju.edu.cn

Dong Ming, State Key Laboratory of Advanced Medical Materials and Devices, Medical School, Tianjin University, 92 Weijin Road, Nankai District, Tianjin, 300072, China, Email: richardming@tju.edu.cn

Multi-omics technologies integration

Abstract

Background

The world has witnessed a steady rise in neurological diseases, which represent a heterogeneous group of disorders characterized by complex pathogenesis involving disruptions at multiple molecular levels, including genomic, transcriptomic, proteomic, and metabolomic levels. These disorders, often caused by genetic mutations, metabolic imbalances, immune dysregulation, and environmental factors, pose significant challenges to global public health due to their high prevalence, mortality, and disability burden.

Results

The advent of high-throughput technologies, such as next-generation sequencing and mass spectrometry, has provided valuable insights into the underlying mechanisms of disease, especially the development of multi- and high-spatial-resolution omics technologies, enabling the interaction of multiple levels of biology and analysis of the complex molecular networks and pathophysiological processes.

Conclusions

This review provides a comprehensive analysis of the latest advancements in multi- and high-spatial-resolution omics, with a focus on their applications in precision diagnostics, biomarker discovery, and therapeutic target identification in brain diseases. The study also highlights the current challenges in the clinical implementation and discusses the future directions, with artificial intelligence being anticipated to enhance clinical translation and diagnostic accuracy significantly.

Keywords: Multi-Omics, Neurological Diseases, Single-Cell Omics, Spatial Transcriptomics

Multi-omics technologies integration

Highlights

- 43% of the global population is suffering from neurological disorders, yet diagnostic and therapeutic strategies remain limited, primarily due to the complexity involving disruptions at multiple molecular levels, including genomic, transcriptomic, proteomic, and metabolomic levels.
- Rapid advances in multi- and high-spatial-resolution omics enable the interaction of multiple levels of biology, showing great potential in the analysis of the complex molecular networks and pathophysiological processes of brain disease.
- This review provides a comprehensive analysis of the latest advancements in multi- and high-spatial-resolution omics, with a focus on their applications in precision diagnostics, biomarker discovery, and therapeutic target identification in brain diseases.

Introduction

Neurological brain diseases encompass a wide range of brain disorders that affect the structure or function of the central and peripheral nervous systems. Globally, an estimated 43% of the world population suffers from neurological diseases, becoming the leading cause of overall disease burden in the world[1]. New treatments that can completely resolve brain diseases have yet to be discovered, due to the blood-brain-barrier (BBB), unclear disease mechanisms, and limited brain analysis tools. Conventional approaches for brain imaging, such as MRI or CT, provide us with abundant information on brain structure and function, but they lack interpretation at the cellular level. Developing advanced analytical technologies is essential for unraveling the complexities of the brain and detecting disease-related therapeutic targets for early diagnosis and precise medicine[2-4].

Since the concept of genomics was first proposed by Thomas H. Roderick in 1986[5], omics technologies, such as genomics, proteomics, metabolomics, lipidomics, glycomics, and transcriptomics, have gained rapid development[6]. However, basic-omics, which employs a singular approach to elucidate the isolated function of a single molecule of a biological system, provides information that is highly fragmented and limited. As the foundation for all living organisms, understanding the complex process by which genetic information is transformed into functional proteins, including transcriptional regulation, translational regulation, RNA/polymer degradation, post-translational modification, and differential transport, plays a critical role in revealing the basic mechanism of brain diseases and discovering new targets[7]. Multiple omics, which integrates basic-omics technologies and combines diverse omics data to extract meaningful information, enables a systematic analysis of the mechanisms and phenotypes of complex biological

processes and advances our understanding of the regulatory relationships among various molecules[6,8-10].

In recent years, the development of high-spatial-resolution (single-cell and spatial) omics technologies has significantly advanced the study of neurological brain diseases. These technologies allow the detailed profiling of molecular changes at the single-cell and tissue-level spatial resolution, thereby uncovering the heterogeneity of disease mechanisms and revealing localized pathophysiological alterations[11]. Single-cell omics provides insights into cellular diversity and gene expression dynamics[12], while spatial omics preserves tissue architecture to map molecular events in their anatomical context[13]. The integration of multi-omics with high-spatial-resolution omics further enhances the biological relevance and depth of disease characterization, offering a more comprehensive view of neurodegenerative and inflammatory brain disorders.

This article provides a systematic review of multi-omics and high-spatial-resolution omics techniques and their application in the neurology field for brain disease diagnosis and treatment. We also discussed the current challenges and prospects, aiming to offer researchers a comprehensive and evidence-based perspective on the development, utility, and translational potential of multi- and high-spatial-resolution omics approaches in the neuroscience area.

2. Omics Technology

2.1 Basic Omics Technology

Different basic omics technologies, including genomics, transcriptomics, proteomics, and metabolomics, have been developed for neuroscience researchers, with distinct advantages and disadvantages shown in Table 1.

Multi-omics technologies integration

2.1.1 Genomics

Genomics investigates the entirety of an organism's genetic material, encompassing DNA sequences, gene repertoire, and regulatory elements. It emphasizes the holistic analysis of genome architecture, functionality, evolution, and regulation, utilizing high-throughput sequencing and bioinformatics for comparative genomic data analysis across individuals and species[8].

Genomic research leverages advanced technologies such as next-generation sequencing, CRISPR-mediated gene editing, and genome-wide association studies (GWAS) to explore genetic variations and their influence on disease susceptibility and progression. GWAS, specifically, employ statistical models to detect associations between genetic variants and phenotypic traits across large populations, providing a robust framework for elucidating the genetic underpinnings of complex neurological conditions[14]. In the realm of neurological brain disorders, genomics has been pivotal in pinpointing risk loci, clarifying disease mechanisms, and guiding the development of targeted therapies and precision medicine strategies[15]. Nevertheless, this field is still facing several challenges, including difficulty in interpretation for non-coding variations, challenges in clinical translation, etc.

2.1.2 Transcriptomics

Transcriptomics, developed by Charles Auffray in 1999, systematically investigates RNA transcripts and their regulatory networks to unravel the molecular underpinnings of cellular function and disease [16,17].

By utilizing high-throughput RNA sequencing (RNA-seq) and other cutting-edge technologies to profile the transcriptome in specific cell types or tissues, transcriptomics facilitates the identification of gene expression patterns, alternative splicing events, and the role of non-coding

RNAs in the progression of neurological diseases. Transcriptomic studies encounter challenges including spatial and temporal heterogeneity of RNA expression in complex tissues[18,19], instability of RNA, and the lack of mature analytical methods.

2.1.3 Proteomics

Proteomics, introduced by Marc Wilkins in 1994, has been widely used to investigate the protein composition and its dynamic changes in cells, tissues, or organisms, serving as a crucial tool for exploring the structure and function of proteins[20]. It serves as a powerful complement to genomics and transcriptomics, encompassing protein identification, comparative proteomics, glycomics, targeted proteomics, etc., which have been applied in new drug development and synthetic biology.

The most prevalent analysis methods for proteomics include the data-dependent acquisition (DDA) mode, also known as the "shotgun" approach; isobaric tags for relative and absolute quantification (iTRAQ)[21]; tandem mass tag (TMT) technology[22]; stable-isotope labeling by amino acids in cell culture (SILAC)[23]; label-free quantification[24]; and data-independent acquisition[25] especially the data independent acquisition-sequential window acquisition of all theoretical mass spectral approach (DIA/SWATH), with their advantages and disadvantages depicted in Table 2. Additionally, mass spectrometry-based sequencing is used to identify and analyze post-translational modifications (PTMs)[26]. These technologies enable proteomics to be an indispensable tool for brain disease diagnosis and treatment, which offers crucial insights into the molecular pathophysiology underlying neurological disorders.

2.1.4 Metabolomics

Introduced by Nicholson et al. in 1999, metabolomics investigates the metabolic responses of

living organisms to exogenous stimuli, environmental changes, or genetic modifications, mapping out comprehensive dynamic profiles of metabolite alterations[27]. As a relatively recent addition to the omics framework following genomics and proteomics, metabolomics aims to characterize small molecules with molecular weights typically ranging from 100 to 1000 Da, either qualitatively or quantitatively, to uncover their functional roles in health and disease[28].

Metabolomic analysis employs a variety of analytical platforms, including nuclear magnetic resonance (NMR), Fourier-transform infrared (FT-IR) spectroscopy, gas chromatography-mass – mass spectrometry (GC–MS), and liquid chromatography-mass spectrometry (LC-MS), which provide high-throughput, high-spatial-resolution, and sensitive detection of metabolite profiles. Currently, metabolomics can be broadly classified into three main approaches based on detection principles: untargeted metabolomics[29], targeted metabolomics[30], and widely targeted metabolomics[31]. Untargeted metabolomics offers broad coverage of the metabolome but at the expense of precision. In contrast, targeted metabolomics enables precise quantification of a predefined set of metabolites, though it limits the discovery of novel biomarkers. Widely targeted metabolomics, meanwhile, strikes a balance between breadth and specificity, allowing for both comprehensive detection and reliable quantification of a larger subset of known metabolites. As illustrated in Figure 1, these methodologies collectively support the exploration of metabolic alterations in complex diseases.

In the context of neurological brain diseases, metabolomics has demonstrated significant potential in identifying disease-specific metabolite signatures that can be employed as diagnostic or prognostic biomarkers[28]. It provides a functional readout of biological processes and has been increasingly used to detect early metabolic changes, assess disease progression, and guide

therapeutic interventions. However, several challenges remain. First, the comprehensive understanding of the metabolome is still in its infancy, with less than 5% of detected metabolites currently annotated. Second, the dynamic and context-dependent nature of metabolic profiles introduces complexity in data interpretation. Third, technical variability across platforms and sample types can hinder reproducibility and comparability. Despite these limitations, ongoing advancements in analytical technologies and bioinformatic tools are expected to position metabolomics as a powerful and indispensable strategy for more precise and efficient diagnosis of neurological disorders.

2.2 Single-Cell Omics Technology

First introduced in 2009 by Tang et al. for RNA-seq at the single-cell level[32], single-cell omics has evolved into a high-throughput platform for analyzing genomic[33-35], transcriptomic, epigenomic[34], and proteomic profiles with cellular resolution[36]. Unlike bulk sequencing, which provides averaged signals across cell populations, single-cell approaches enable the deconvolution of individual cellular states, revealing heterogeneity with unprecedented precision. Single-cell transcriptomics remains the most mature and widely applied modality, having advanced from low-throughput methods to high-throughput platforms capable of profiling millions of cells. Representative technologies include full-length amplification (e.g., SMART-seq), high-throughput barcoding (e.g., 10× Genomics), and multi-omics compatible methods (e.g., Andeplete)[37]. Key steps in the workflow encompass cell isolation, nucleic acid amplification, sequencing, and computational analysis, with isolation and amplification being particularly critical for data quality. These technologies are increasingly integrated with spatial and epigenomic approaches to provide comprehensive insights into gene regulation, cellular function, and disease mechanisms. Despite

significant progress, challenges such as amplification bias, cell dropout, and data standardization remain, necessitating continued methodological innovation.

2.3 Spatial Omics Technology

First introduced in 2016 by Joakim et al. for in situ RNA capture, it represents a significant advancement in molecular biology by enabling the profiling of transcriptomic information while preserving spatial context within intact tissue[38]. As illustrated in Figure 2, unlike single-cell sequencing, which provides molecular resolution but loses spatial information, spatial omics allows the mapping of gene expression, epigenetic modifications, protein localization, and metabolic profiles to precise anatomical coordinates, offering a more comprehensive view of tissue organization and function[39]. The field has developed multiple modalities, including spatial transcriptomics[40], (epi) genomics[41], proteomics[42], and metabolomics[43]. Among these, spatial transcriptomics is the most mature, utilizing either in situ hybridization techniques (e.g., MERFISH, seqFISH) for subcellular-resolution transcript mapping or sequencing-based methods (e.g., Visium, Slide-seq) that spatially barcode RNA through microarray capture or hybridization-based imaging[44-46]. These technologies are increasingly being applied in tumor microenvironment characterization, neural development studies, inflammatory disease modeling, and organoid/tissue engineering to uncover spatially regulated gene expression patterns and their functional implications. Despite its potential, spatial omics still faces challenges such as limited resolution, high cost (often exceeding $1,500 per sample), and computational demands in 3D spatial data integration. Future directions will focus on improving resolution, reducing cost, enhancing data analysis tools, and promoting multi-omics integration to better elucidate the complex regulatory networks governing tissue biology and disease progression.

3. Application of Multi-Omics Technologies in the Diagnosis or Treatment of Chronic Neurological Diseases

Neurological diseases constitute a major global health challenge, including but not limited to Alzheimer's disease (AD), Parkinson’s disease (PD), epilepsy, multiple sclerosis (MS), stroke, hydrocephalus, and various neurological diseases[47]. With the rapid advancement of science and technology, multi-omics approaches have become increasingly pivotal in the research of neurological diseases. As shown in Figure 3, by integrating multi-layered data, researchers can delve deeper into the molecular mechanisms underlying these diseases, thereby offering novel perspectives and methods for clinical diagnosis, treatment, and prevention. This review provides a comprehensive overview of the advancements made in the application of multi-omics technologies over the past five years in the study of representative neurological diseases.

3.1 Application of Multi-Omics Technologies in AD

AD, commonly referred to as senile dementia, is one of the most prevalent neurodegenerative disorders to date and a leading cause of disability and death among the elderly[48,49]. The disease typically begins insidiously and progresses irreversibly, leading to severe cognitive impairment[50-52]. However, there is still no effective treatment for AD, and the underlying mechanisms contributing to the pathogenesis of AD remain incompletely elucidated, although amyloid plaques and TAU neurofibrillary tangles have revealed major pathological changes in AD[53,54]. These pathological changes may trigger synaptic dysfunction, neuroglial inflammation, and eventually neuronal loss in the cerebral cortex, subcortical regions, temporal lobe, parietal lobe, and cingulate gyrus[55-57], and may even affect the gut microbiome[47,58].

The field of AD intervention has long been confronted with two major challenges: first, the

pronounced delay in early diagnosis—owing to its insidious onset, most patients are identified only at moderate to severe stages when pathological progression has become largely irreversible[59], resulting in a critical missed window for intervention; second, the incomplete understanding of its pathogenetic mechanisms—although core pathological features such as amyloid plaques and TAU neurofibrillary tangles have been identified[52,54], the molecular regulatory networks underlying subsequent neuronal injury, neuroinflammation, and gut microbiome dysbiosis remain poorly elucidated[55]. Prior to the emergence of multi-omics technologies, these challenges were compounded by methodological limitations. Conventional hypothesis-driven approaches largely focused on isolated pathways such as Aβ deposition or TAU phosphorylation[54]. While valuable for elucidating specific pathological components, such strategies failed to capture system-level interactions among gene expression, protein networks, and metabolic alterations. This constrained the discovery of early specific biomarkers and hindered the development of integrated therapeutic strategies targeting multiple pathological processes.

The advent of multi-omics approaches has provided a pivotal toolkit to overcome these limitations[60]. This global perspective transcends the constraints of single-pathway investigations and marks a shift from compartmentalized pathological observation towards systematic mechanistic decoding. It provides a robust foundation for the identification of early intervention targets and the development of precision therapeutics, heralding a new era in AD research and clinical management

3.1.1 Basic Omics Approaches in AD

Basic-omics approaches have provided valuable, unique insights into different aspects of AD pathogenesis (Supplementary Table S1). Genomic studies have identified high-risk genes and epigenetic modifications associated with AD, including *APP, PSEN1, TAU, APOE4*, acetylation

status of histone H4 at lysine 16 (*H4K16ac*), and *H3K9ac*. Among these, *APOE4* is particularly well-characterized in its association with late-onset AD, as it accelerates vascular dysfunction, disrupts the BBB, and promotes neuronal degeneration. These effects position *APOE4* as a central player in AD's vascular and neurodegenerative mechanisms, making it a key diagnostic and prognostic marker[61].

Epigenomic analyses have revealed the influence of single-nucleotide polymorphisms (SNPs) in *TAU* and enhancer regions on chromatin structure and disease progression. In particular, the *H4K16ac* is significantly altered with aging and AD-related gene expression. Compared to non-AD elderly individuals, 25,000 peaks show loss of *H4K16ac*, whereas 9,000 exhibit increased acetylation in AD patients. These changes suggest that *H4K16ac* may serve as an epigenetic link between aging and AD pathology, and as a potential diagnostic biomarker[62]. Similarly, *H3K9ac* and TAU have been identified as epigenetic and proteomic biomarkers, with *H3K9ac* showing parallel acetylation patterns in TAU-related contexts, indicating a complex interplay between TAU pathology and chromatin regulation[63].

Transcriptomic profiling has enabled the identification of co-expression modules that are closely linked to AD pathology. One such module, M109, is most directly associated with cognitive decline and amyloid load. Within this module, *INPPL1* and *PLXNB1* have been identified as potential candidates for in vitro amyloid biology investigation. Notably, *INPPL1* and *PLXNB1* are associated with extracellular β-amyloid levels in astrocyte cultures, suggesting their relevance in early detection and mechanistic understanding of AD[64]. Through transcriptomic analyses, Shouneng Peng and colleagues elucidated that aging and AD share common alterations in genes associated with phosphorylation, alternative splicing, and the cytoskeleton. Both conditions exhibit

concomitant downregulation of synaptic function, leading to early impairment in neuronal communication. AD-specific changes are predominantly characterized by upregulation of neuroinflammatory pathways and downregulation of genes involved in myelination and lipid metabolism. Based on these findings, promoting myelin repair and stabilizing cerebral lipid homeostasis emerge as promising novel therapeutic strategies[65].

Proteomic studies have uncovered disease-specific alterations in protein expression. For instance, elevated levels of FYN, YES1, and STAT3 in AD-derived induced neurons are associated with neuroinflammation, TAU phosphorylation, and increased amyloid-42 production[66]. Proteomics also found that APP/PS1 and APOE4 knock-in mice lead to changes in early hippocampal protein expression profiles, and that the changes in hippocampal proteins involved in insulin signaling and the mitochondrial electron transport chain may be key biological processes in AD progression[67]. These findings suggest that proteomics can help distinguish AD subtypes and identify critical pathways in disease progression.

Phosphoproteomic analyses have further highlighted the role of PTMs in AD. Abnormal phosphorylation of GSK3β and PPP3CA in models treated with low-dose copper is linked to mitochondrial dysfunction[68]. A recent study has revealed the staged pathophysiological progression in preclinical autosomal dominant AD via cerebrospinal fluid (CSF) proteome analysis. The six-protein model (GFAP, NPTX2, PEA15, SMOC1, SMOC2, TNFRSF1B) demonstrates high predictive accuracy ($AUC > 0.9$), validated independently, and offers a valuable tool for ultra-early autosomal dominant AD screening, precise staging, and clinical trial enrollment[69].

Metabolomic research has identified sphingolipids as potential early biomarkers of AD, and dysregulated amino acid and tryptophan metabolism have also been observed in transgenic

models[70]. Furthermore, some investigators unveiled a metabolic shift towards aerobic glycolysis in AD-derived induced neurons, triggered by pyruvate kinase M2 (PKM2)'s loss of metabolic activity, nuclear translocation, and interaction with *STAT3* and *HIF1α*, which collectively precipitate metabolic and transcriptional changes that underlie neuronal identity loss and heightened vulnerability in sporadic AD[71].

3.1.2 High-spatial-resolution Omics Technologies in AD

High-spatial-resolution omics technologies have transformed the understanding of AD by revealing cell-type-specific and spatially resolved molecular alterations[72]. Mathys et al. pioneered the use of single-cell RNA sequencing (scRNA-seq) in AD, profiling ~80,000 prefrontal cortex cells across disease stages and identifying myelination maintenance as a critical regulatory response, especially the relevant gene *LINGO1*[73]. The experiment also revealed gender differences that exist in AD, including transcriptional response differences and the overrepresentation of females in AD-associated cell subpopulations. Grubman et al. further analyzed the entorhinal cortex, an early AD-affected brain region, and found that astrocytes exhibited distinct activation patterns compared to other areas. Expression changes of multiple AD risk genes (e.g., *APOE*, *TREM2*) were also validated in specific cell types. For example, *APOE* expression was elevated in microglia and astrocytes, correlating with TAU pathology severity, offering cell-specific insights into genetic risk mechanisms[74].

Spatial transcriptomics has further advanced AD research by preserving spatial context during transcriptome profiling. In AD mouse models, this method detected early transcriptional dysregulation in 100-μm plaque microenvironments, particularly in myelination-related and oligodendrocyte gene networks. Late-stage spatial profiles revealed 57 plaque-induced genes (*PIGs*)

enriched in complement activation, oxidative stress, lysosomal dysfunction, and neuroinflammation[75]. Integration of 10× Genomics Visium spatial transcriptomics with co-immunofluorescence mapping in human middle temporal gyrus identified five layer- and white matter-specific marker genes, including both established (*RORB, PCP4, MBP*) and novel (*SPARC, CALB2, DIRAS2, KRT17*) candidates with potential as diagnostic and mechanistic biomarkers[76].

Integrative omics strategies combining single-cell and spatial transcriptomics are beginning to illuminate intercellular communication in AD pathology. A recent study identified a *PTPRG*-expressing microglial subpopulation that promotes neuronal *VIRMA* expression, which enhances m^6A modification of *PRKN* transcripts, leading to RNA destabilization, impaired mitophagy, and neuronal death. These findings highlight *PTPRG* and *VIRMA* as promising therapeutic targets for AD[77].

Using single-nucleus RNA sequencing (snRNA-seq) and spatial transcriptomic data, one study constructed a comprehensive brain cell atlas comprising 1.65 million single-nucleus transcriptomes from 437 aged individuals. It identified glial and neuronal subpopulations associated with AD, and employed causal modeling to highlight lipid-associated microglial subsets—which drive Aβ pathology or mediate Aβ-TAU crosstalk—alongside TAU-responsive astrocytic populations. These findings provide novel cellular insights into AD pathogenesis and suggest promising therapeutic targets. Furthermore, the identification of distinct brain aging trajectories may facilitate early diagnosis and personalized treatment strategies[78].

Recent multi-omics studies on human primary microglia have elucidated how genetic regulation of chromatin accessibility influences transcriptional programs. Integrative transcriptomic and epigenomic analyses nominated *SPI1/PU.1* as a key transcriptional regulator of microglial gene

expression and AD risk, with reduced *PU.1* binding destabilizing chromatin accessibility. These findings reinforce the central role of *SPI1/PU.1* in microglial dysfunction in AD, while also identifying additional contributing transcription factors[79]. Through single-cell epigenomics and transcriptomics analysis of the prefrontal cortex, the study by Chen et al. constructed a cell-type-resolved regulome atlas of the human brain, revealing significant enrichment of AD risk loci in microglial enhancers and binding sites of transcription factors including *SPI1, ELF2, and RUNX1*. Previous research identified 9,628 cell-type-specific ATAC-QTLs and integrated these with peak-to-gene linkages to decipher variant-regulatory circuits in AD. Furthermore, the experiment uncovered differential accessibility of regulatory modules in glial cells during late-stage AD and in neurons at early stages, with advanced AD exhibiting global epigenomic dysregulation, suggesting widespread epigenomic erosion and loss of cellular identity[80].

One recent study employed single-cell epigenomics and spatial genomics to characterize AD cell populations, defining two disease stages: an early phase featuring slow pathological buildup with inflammatory microglia, reactive astrocytes, loss of SST$^+$ inhibitory neurons, and remyelination; and a late phase marked by exponential pathology increase and loss of excitatory and inhibitory (Pvalb$^+$/Vip$^+$) neurons. These findings were validated in independent AD cohorts, and the systematic application of multi-omics technologies coupled with quantitative neuropathology enables effective modeling of disease progression across the spectrum of AD severity[81].

Through snRNA-seq combined with spatially resolved transcriptomics, Maria et al. demonstrated that compared to sporadic AD, autosomal dominant AD cases exhibit pronounced upregulation of autophagy-related and chaperone genes. Spatial transcriptomic analysis further confirmed the specific activation of chaperone-mediated autophagy pathways in *PSEN1-E280A*

carriers. In autosomal dominant cases, they observed astrocytic overexpression of *LRP1*, elevated *FKBP1B* expression, and reduced neuronal *PSEN1* levels, which may collectively underlie a protective mechanism against AD pathology[82].

3.1.3 Multi-Omics Integration in AD

Multi-omics integration has enabled the identification of disease-specific molecular modules and biomarker signatures that reflect the underlying pathophysiology of AD[83] (Supplementary Table S1). For example, combined proteomic and transcriptomic analyses have revealed two distinct AD-related modules: the *MAPK*/metabolic module, which is associated with the rate of cognitive decline, and the matrix body (matrixsome) module, whose expression is modulated by the *APOE ε4* allele[84]. Further integration of proteomic and transcriptomic data has identified *FBP1*, *FBP2*, *RHOH*, *JPH2*, *ERAP2*, and *SCLT1* as upregulated proteins in *APOE4* carriers compared to controls. In contrast, the Myeloid basic protein encoding gene (*MBP*) is among the top-ranked candidate genes that reinforce the importance of myelination in AD pathogenesis and cognitive decline. Notably, these biomarkers show consistent expression patterns in both plasma and brain tissue[85]. A multi-omics approach integrating genomics, transcriptomics, proteomics, and metabolomics has identified *ABCA1*, *CPT1A*, adiponectin, and *NGAL* as key players in the regulation of acylcarnitines and amino acid metabolism. Disruptions in the homeostasis of short-chain acylcarnitines and essential amino acids are closely correlated with disease severity, suggesting that these molecules may serve as early diagnostic indicators[86]. In addition, *IVD*, *CYFIP1*, and *ADD2* have been identified as serum-based diagnostic biomarkers through proteomic and transcriptomic analysis. *IVD* shows significantly higher protein abundance in AD patients, whereas *CYFIP1* and *ADD2* are downregulated. The combination of these three proteins improves early detection and disease

discrimination[87]. From a lysosomal perspective, multi-omics integration has revealed that *CSTD*, *CTSB*, *CTSD*, and *GM2A* are significantly upregulated in AD patients and have been validated as CSF and plasma biomarkers. These lysosomal proteins show progressive fold changes during AD progression, further supporting their diagnostic utility[88]. Moreover, *PBXIP1* has been identified as a multi-omics-linked diagnostic target through genomics, transcriptomics, and proteomics. *PBXIP1*-encoded proteins are significantly associated with all three neuropathological hallmarks of AD-Aβ plaques, neurofibrillary tangles, and neurodegeneration. It functions in hippocampal neurons, astrocytes, and mTOR signaling, linking it to both neuropathology and cognitive dysfunction[89]. Epigenomic and proteomic integration has further revealed *H3K27ac* as a potential diagnostic marker, particularly in the entorhinal cortex. Analysis has shown that AD risk variants are significantly enriched in *H3K27ac* peak regions, including *CR1, GPR22, KMO, PIM3, PSEN1,* and *RGCC*[90]. Additionally, integrative studies using transcriptomics, proteomics, and epigenomics have demonstrated that *H3K27ac* and *H3K9ac*, transcriptionally active post-PTMs, are genome-wide dysregulated in AD, with RNA-seq revealing increased expression of histone acetyltransferases. These modifications are enriched in Aβ and TAU-related pathways, and their dysregulation contributes to transcriptional and chromatin dysfunction[91]. These epigenetic alterations may serve as early indicators of AD-related gene expression and chromatin dysregulation. Pathway-level integration of proteomics, metabolomics, and lipidomics has identified gender-dependent effects in GABA synthesis, arginine biosynthesis, and alanine/aspartate/glutamate/ arginine metabolism. These findings emphasize the importance of lysophospholipid and amino acid metabolism in the AD brain and suggest the need for gender-specific diagnostic models[92]. Finally, four CSF proteins-14-3-3 zeta/delta, clusterin, interleukin-15, and transgelin- 2 -have been shown

to enhance AD prediction accuracy through multi-omics integration[93].

From a therapeutic perspective, multi-omics integration has revealed potential drug targets and intervention points (Supplementary Table S1). One such target is *TRPV1*, a pharmacologically modifiable receptor that, when activated, rescues memory deficits and neuronal loss in *APOE4* mice on a high-fat diet. These results highlight the potential of *TRPV1*-based therapies in mitigating AD pathology[94]. In late-onset AD, *ATP6V1A* has been identified as a key regulator of the most dysregulated neuronal subnetwork. Targeting *ATP6V1A* has shown therapeutic potential, as *NCH-51*, a compound targeting this pathway, ameliorates neuronal damage in drosophila models[95].

Several computational platforms have been developed to facilitate the integration and interpretation of multi-omics data in AD. For instance, the Alzheimer's Disease Genome-Wide Positioning Systems platform (AlzGPS) is used for drug discovery by mining AD-related targets and clinically relevant candidate drugs[96].

Despite advancements in multi-omics technologies, AD research remains heavily reliant on classic biomarkers—Aβ and TAU, which continue to dominate both mechanistic and clinical frameworks. While omics approaches have identified numerous candidate biomarkers and pathways, few have been rigorously validated in large, diverse cohorts or translated into clinically usable tools. A major persistent challenge is the fragmented integration of multi-omics data: genomic, proteomic, and metabolomic layers are often studied in isolation rather than as interconnected networks. This lack of systematic integration limits our ability to uncover actionable biological mechanisms or subtype-specific signatures, hindering the development of personalized diagnostic and therapeutic strategies.

Taken together, these studies underscore the importance of integrating multi-omics data to

elucidate the associations between brain functional and structural changes related to AD. The integration of multiple omics layers provides a comprehensive and mechanistically grounded view of the disease, offering a solid theoretical foundation for the development of precision diagnostics and targeted therapies. A detailed diagram illustrating the multi-omics-driven pathogenesis of AD is shown in Figure 4.

3.2 Application of Multi-Omics Technologies in PD

PD is the second most common chronic neurodegenerative disorder globally, following AD, affecting approximately 1% to 2% of the population. It is characterized by resting tremor, increased muscle tone (rigidity or stiffness), bradykinesia (slowness of movement), and postural instability, with numerous patients also experiencing cognitive impairments or dementia[4,97,98]. The disease primarily impacts the motor system[99,100] and often has an insidious onset with early diagnosis lacking distinct features[47]. The BBB is disrupted in PD disease, and leukocytes and neutrophils enter the brain and release large amounts of inflammatory cytokines, such as tumor necrosis factor-α (TNF-α), interleukin-1β (IL-1β), and interleukin-6 (IL-6)[101]. The accumulation of α-synuclein in Lewy bodies and Lewy neurites, predominantly in the substantia nigra, leads to the loss of dopaminergic neurons and the manifestation of prominent symptoms. Lewy bodies may also be associated with AD, complicating the determination of PD's etiology and pathogenesis. At present, PD diagnosis depends mainly on clinical evaluation and neuroimaging, but these methods are subject to diagnostic delays and misclassification, especially in the early stages.

A central unresolved question in PD research is how to accurately define disease subtypes and identify early molecular events prior to overt neurodegeneration. Without multi-omics approaches, studies were often limited to clinical phenotypes and single molecular layers, failing to capture the

Multi-omics technologies integration

systemic interactions among genetic variants, transcriptional dysregulation, protein misfolding, and metabolic dysfunction. This narrow view hindered the discovery of predictive biomarkers and subtype-specific therapeutic targets, particularly at pre-symptomatic stages. The application of integrated genomics, transcriptomics, proteomics, and metabolomics has transformed this paradigm. These technologies enable high-resolution mapping of endophenotypes and dynamic pathways, revealing novel biomarkers such as *GPNMB* and DDC, and unmasking critical mechanisms like neuroinflammation, oxidative stress, and mitochondrial impairment. Multi-omics thus provides a holistic, mechanistic framework for early diagnosis, patient stratification, and targeted interventions in PD.

3.2.1 Basic-Omics Approaches in PD

Basic-omics approaches have yielded a range of candidate biomarkers and pathogenic insights in PD (Supplementary Table S2). Genomic studies have identified several high-risk loci and genes associated with PD, including leucine-rich repeat kinase 2 (*LRRK2*), *IL1R2, ZNF184, PARK16, ITPKB, HLA, MAPT, TRIM10,* and *SETD1A*[102,103]. Allelic variations in *LRRK2* and *IL1R2* significantly increase PD risk, while *HLA* and *MAPT* loci are repeatedly implicated in disease susceptibility. Most of these genes are involved in autophagy and lysosomal function-related pathways, which are critical for the clearance of misfolded α-synuclein and other toxic proteins[103].

Transcriptomic profiling has revealed dysregulated gene expression patterns in PD. Signal sequence receptor subunit 1 (*SSR1*), a gene encoding a mitochondrial protein, is upregulated in PD patients and negatively correlated with dopaminergic neuron survival. Notably, *SSR1* is upregulated in peripheral blood before the onset of motor symptoms, suggesting its potential as an early diagnostic marker. A machine learning (ML)-based random forest (RF) classifier incorporating

Multi-omics technologies integration

SSR1 expression achieves a high diagnostic accuracy (AUC = 0.91), reinforcing its value in PD detection[104].

Proteomic studies have identified several disease-associated proteins and inflammatory signatures in PD. OMD, CD44, VGF, PRL, and MAN2B1 show significant expression changes in PD patients and are strongly correlated with clinical scores, including motor severity and disease progression[105]. In addition, LRRK2 carriers exhibit enhanced neuroinflammatory profiles, further supporting its pathogenic and diagnostic relevance. These proteins are considered potential biomarkers for PD diagnosis.

Metabolomic analyses have revealed widespread metabolic dysregulation in PD, particularly in lipid and energy metabolism. Alterations in carnitine shuttle, sphingolipid metabolism, arachidonic acid metabolism, and fatty acid biosynthesis are consistently observed, with carnitine shuttle activity being most significantly perturbed in unmedicated PD patients[106].

Additionally, short-chain fatty acids, including butyric acid, are significantly reduced and correlated with cognitive decline and motor dysfunction[107]. A comprehensive urinary metabolomic profile has identified 139 differentially regulated metabolites, with proline among the most predictive[108]. Moreover, phenylacetic acid, phenylacetylglutamine, histidine, uric acid, and imidazoleacetic acid are consistently upregulated in urine and show strong diagnostic power in early-stage PD, with a 45-metabolite model achieving high accuracy[109]. Using the same technique, this team also found that 18 differential metabolites changed metabolic pathways related to branched-chain amino acid (BCAA) metabolism, glycine derivatives, steroid hormone biosynthesis, tryptophan metabolism, and phenylalanine metabolism, confirming the team's earlier findings[110].

3.2.2 High-spatial-resolution Omics Technologies in PD

Semra et al. employed snRNA-seq and GWAS to profile midbrain cell contributions, identifying a PD-specific neuronal cluster overexpressing *CADPS2* and showing reduced tyrosine hydroxylase levels[111]. Glial populations in affected regions exhibited disease-restricted proliferation, along with dysregulation in unfolded protein response and cytokine signaling pathways, while reactive astrocytes showed *CD44* upregulation and microglia displayed a pro-inflammatory trajectory marked by elevated *IL-1B, GPNMB,* and *HSP90AA1*. These results position *IL-1B, GPNMB,* and *HSP90AA1* as possible diagnostic biomarkers for PD[111]. A recent scRNA-seq study integrated with gene set enrichment analysis provided the first single-cell resolution gene atlas of the dorsolateral prefrontal cortex in PD, revealing transcriptomic changes across microglia, astrocytes, oligodendrocytes, and oligodendrocyte precursor cells. Key findings include mitochondrial dysfunction, immune dysregulation, and impaired protein folding in glial cells, underscoring their roles in disease progression. This experiment shows that using *HSP90* inhibitors can speed up the breakdown of inflammasomes, thus lowering inflammatory responses and easing neurodegeneration. This finding opens up novel possibilities for the selection of immunotherapeutic approaches[112].

While dopaminergic neuron loss in the substantia nigra pars compacta (SNpc) is a defining pathological hallmark of PD, the molecular basis of their selective vulnerability remains unclear. Integrating single-cell genomics and slide-seq spatial transcriptomics, Tushar et al. identified a distinct *AGTR1*-expressing neuronal subtype spatially confined to the SNpc ventral tier that exhibits heightened PD susceptibility. This vulnerable subtype demonstrated significant upregulation of *TP53* and *NR2F2* target genes, implicating these transcription factors in degenerative processes, and

displayed pronounced transcriptomic alterations in stress pathways regulated by *TP53/NR2F2*, directly linked to PD-associated neuronal death[113]. Combining single-cell transcriptomic and proteomics, Biqing et al. revealed an inverse correlation between α-synuclein pathology and chaperone expression in excitatory neurons, concurrent with attenuated neuron-astrocyte interactions and exacerbated neuroinflammation in PD. ScRNA-seq analysis demonstrated *SYN2* enrichment in PD brains, indicating significantly enhanced synaptic signaling at both transcriptional and proteomic levels, providing novel insights into coordinated molecular dysregulation[114].

3.2.3 Multi-Omics Integration in PD

In the diagnostic process of PD, multi-omics technologies have achieved remarkable success by precisely identifying a series of key biomarkers and pathways (Supplementary Table S2). At the protein and transcript level, *GPNMB*, *CD38*, and *DGKQ* have been identified as candidate diagnostic biomarkers through integrative proteomic and transcriptomic analysis. These proteins show significant associations with PD risk, and their expression is supported by quantitative trait locus analysis and fine mapping[115]. Furthermore, DDC is consistently upregulated in CSF, blood, and urine of PD patients and is strongly correlated with symptom severity[116]. DDC and related proteins are thus considered viable targets for accurate PD diagnosis.

From a therapeutic perspective, the integration of transcriptomics and metabolomics has revealed that Buyang Huanwu Decoction (BHD) exerts therapeutic effects in PD by modulating key metabolic pathways, including the relaxin signaling pathway, adhesion patch, and PI3K-Akt signaling pathway, and by reducing disease-related symptoms. In preclinical models, BHD treatment enhances dopaminergic neuron survival and improves motor function, indicating its potential for neuroprotection and functional recovery[117]. In addition, genomic and metabolomic

studies have identified circular RNA *CircSV2b* as a potential therapeutic target in PD. *CircSV2b* is significantly deregulated in PD mouse models compared to wild-type controls, and its overexpression via the ceRNA-Akt1 axis has been shown to mitigate oxidative stress, a central pathological mechanism in PD[118].

Despite advances in multi-omics research, the field of PD continues to grapple with three fundamental challenges: clinical heterogeneity (such as differential treatment responses between tremor-dominant and postural instability/gait difficulty subtypes), poorly understood mechanisms of α-synuclein propagation (particularly via proposed gut-to-brain pathways through the vagus nerve), and a persistent lack of disease-modifying therapies. Future studies should prioritize deeply phenotyped longitudinal cohorts—incorporating real-time motor monitoring via wearable sensors and comprehensive autonomic function assessments—alongside the integration of single-cell sequencing and spatial transcriptomics to elucidate region-specific neurodegeneration in the substantia nigra. The development of advanced computational models (e.g., multimodal data fusion and Mendelian randomization) will be essential to infer causal pathways from high-dimensional data. By focusing on prodromal cohorts and currently underrepresented populations, researchers can systematically unravel neuroimmune-metabolic cross-talk, ultimately enabling the discovery of early biomarkers, patient stratification, and personalized therapeutic strategies.

Taken together, these integrative approaches not only enhance our understanding of PD heterogeneity but also pave the way for the development of precision diagnostics and personalized therapies. A schematic overview of the multi-omics-driven pathogenesis and therapeutic mechanisms in PD is presented in Figure 5.

3.3 Application of Multi-Omics Technologies in Epilepsy

Epilepsy is the second most common neurological disorder, affecting approximately 3–6% of pediatric neurological cases and occurring across the entire lifespan[119]. It is associated with recurrent abnormal neuronal discharges in the central nervous system (CNS), leading to episodic dysfunction in motor, sensory, autonomic, and cognitive domains, and contributing to cognitive, psychological, and social impairments[120,121]. The etiology and pathogenesis of epilepsy are highly heterogeneous, involving genetic, metabolic, and inflammatory factors. Over the past decade, omics technologies-including metabolomics, proteomics, and transcriptomics-have provided critical insights into the molecular underpinnings of epilepsy, although the precise mechanisms remain incompletely understood.

A fundamental challenge in epilepsy research is how to decipher the complex, multi-dimensional interactions among genetic predisposition, neuronal excitability, inflammatory processes, and metabolic dysregulation that collectively contribute to seizure generation and progression. Prior to the advent of multi-omics technologies, research was often siloed into isolated domains—electrophysiology, candidate genes, or histopathology—which provided fragmentary insights but failed to capture the systemic and dynamic nature of epileptogenesis. This limitation hindered the identification of convergent pathways and translatable biomarkers, especially across different epilepsy subtypes and developmental stages.

3.3.1 Basic-Omics Approaches in Epilepsy

Basic-omics technologies have played a crucial role in identifying key molecular alterations in epilepsy across different biological layers (Supplementary Table S3). Transcriptomic studies have identified dysregulated gene expression in various epilepsy subtypes. In generalized epilepsy,

Multi-omics technologies integration

P38MAPK, JAK STAT, PI3K, and *MTOR* signaling pathways are consistently upregulated and show stable regulation in affected patients[122]. In temporal lobe epilepsy (TLE), hub genes such as *TLR2*, *LGALS3*, *SERPINE1*, and *STAT3* are positively correlated with seizure frequency and are associated with microglial/macrophage activation, ECM remodeling, cell motility, and immune responses[123]. These findings suggest that transcriptomics can provide mechanistic insights into seizure onset and progression.

By integrating analyses of gray matter volume with transcriptomic data from the human brain atlas, one study revealed that patients with focal to bilateral tonic–clonic seizures (FBTCS+) exhibit more extensive bilateral cortical and subcortical morphological alterations compared to the non-FBTCS group (FBTCS−). Both groups were involved in neurodevelopmental and synaptic-related pathways. Cell-type mapping demonstrated that both excitatory and inhibitory neurons were affected in the FBTCS− group, whereas only excitatory neurons showed significant changes in the FBTCS+ group, suggesting that excitatory/inhibitory imbalance and abnormalities in the *GABAergic* system may constitute a key mechanism underlying FBTCS[124]. Through the establishment of a GABRA4 knockout (GABRA4$^{-/-}$) mouse model, the study found that the mice exhibit core behavioral phenotypes resembling high-functioning autism, including social deficits, enhanced spatial memory, and reduced susceptibility to seizures. Subsequent hippocampal transcriptomic and interactome analyses revealed that the *NMDAR* signaling pathway plays a central regulatory role in mediating these autism-like behaviors and anti-epileptic phenotypes, indicating a functional convergence between the *GABAergic* system and *NMDA* receptor pathways in the pathogenesis of autism. *Gabra4* knockout mice exhibited significantly decreased susceptibility to pentylenetetrazol-induced seizures, indicating a protective effect against epileptic activity[125]. By

integrating [^{18}F]SynVesT-1 PET synaptic imaging and transcriptomic data, research revealed reorganization in the synaptic density similarity network in TLE, characterized by reduced connectivity, decreased clustering, and increased path length, particularly within temporolimbic and frontoparietal circuits. These network alterations correlated spatially with TLE risk gene expression and identified 183 downregulated synaptic transmission-related genes, including core *GABAergic* genes such as *RBFOX1*. The findings suggest that coordinated downregulation of risk genes may contribute to synaptic network dysfunction and epileptogenesis in TLE, offering new directions for molecular and genetic network-targeted therapies[126]. Proteomic investigations have revealed abnormal protein expression patterns in both diagnostic and therapeutic contexts. In TLE, glial fibrillary acidic protein (GFAP) is consistently downregulated in brain tissue with high spike frequency and shows a strong negative correlation with seizure severity, indicating its role in reactive astrocyte function and neuroprotection[127]. In the hippocampal region of epileptic brains, 144 differentially expressed proteins, such as ADP-ribosyl cyclase (ADPRC), lysophosphatidic acid receptor 3 (LPAR3), calreticulin, ubiquitin carboxyl-terminal hydrolase L1 (UCH-L1), synaptosome-associated protein 25 (SNAP-25), and transgelin-3, have been identified, with most related to Ca^{2+} homeostasis. Notably, inhibition of calcium influx has been shown to alleviate seizures, supporting the relevance of ion channel and signaling protein targets[128]. Additionally, tutin induces epilepsy and causes significant neurological damage by activating calcineurin, a key phosphatase involved in seizure initiation and progression[129].

Metabolomic analyses have uncovered distinct metabolic signatures in epilepsy. In pediatric epilepsy, N-acetyl glycoprotein, lactate, creatine, glycine, and lipids are elevated, while citrate levels are reduced, suggesting their potential as diagnostic biomarkers[130]. In mesial temporal lobe

epilepsy (MTLE), γ-aminobutyric acid (GABA) is significantly upregulated in the epileptogenic zone of KA-MTLE mice and is considered a specific metabolic marker for MTLE[131]. These findings indicate that metabolomics can reveal early-stage metabolic perturbations and pathophysiological changes in epilepsy.

3.3.2 High-spatial-resolution Omics Technologies in Epilepsy

High-spatial-resolution omics technologies have enabled the identification of dysfunctional neuronal subtypes associated with epileptic seizure activity, particularly in the human temporal cortex. SnRNA-seq of over 110,000 neurons revealed the most significant transcriptomic alterations in principal neuron subtypes, including *L5-6_FEZF2* and *L2-3_CUX2*, as well as *GABAergic* interneurons marked by *SST* and *PVALB* gene expression. Among the most profoundly dysregulated pathways is glutamatergic signaling, particularly through the upregulation of genes encoding AMPA receptor auxiliary subunits in *SST* and *PVALB* subtypes. These results highlight the central role of *GABAergic* interneurons in early epileptogenesis and their potential as diagnostic targets[132]. Comparative scRNA-seq studies between post-traumatic epilepsy (PTE) and hereditary epilepsy revealed distinct cellular landscapes. Hereditary epilepsy samples exhibited increased abundance of oligodendrocytes and astrocytes, but reduced microglia and neuronal populations compared to PTE. Within microglia and astrocytes, the IL-17 signaling pathway emerged as a potential therapeutic and biomarker candidate, pointing to immune and inflammatory mechanisms as critical contributors to neuronal dysfunction in PTE. Notably, *XIST*, a long non-coding RNA associated with inflammatory cell infiltration, fibrosis, and satellite glial cell activation, was significantly upregulated in PTE, suggesting its role in epileptogenic progression and early detection[133].

To further uncover the molecular basis of hippocampal sclerosis and hyperexcitability in TLE,

a multi-omics strategy combined scRNA-seq, snRNA-seq, and Xenium spatial transcriptomics. The spatial data highlighted glia-specific gene upregulation and neuron-specific downregulation, with key dysregulated genes including *SPP1* and *TREM2* (upregulated in glia) and *TLE4* and *SIPA1L3* (downregulated in neurons), suggesting their diagnostic and mechanistic relevance in TLE[134].

3.3.3 Multi-Omics Integration in Epilepsy

In epilepsy diagnosis, multi-omics research has identified several key targets for precise interventions to deepen our understanding of the origins of epilepsy (Supplementary Table S3).

Integrated genomics and transcriptomics have revealed the key role of *SESN3* in the pro-convulsant gene network of the human hippocampus in epilepsy. *Sestrin 3* positively regulates modules in macrophages, microglia, and neurons, and is considered a potential diagnostic marker[135]. Integrative proteomic and transcriptomic analyses have revealed the involvement of the transforming growth factor β (*TGF-β*) signaling pathway in cardiac dysfunction associated with epilepsy. Within this pathway, *STAT3*, *ERBB*, and *MAPK8* emerge as key regulators of cardiac alterations induced by seizure activity[136]. Integration of proteomics and metabolomics has identified glutathione S-transferase M1 (GSTM1) and aldehyde dehydrogenase 2 (ALDH2) as protein hubs in the somatosensory cortex and thalamus, respectively[137]. Furthermore, genomic and metabolomic integration has identified lactate, creatine, phosphocreatine, and choline as metabolic markers with distinct expression patterns in epilepsy. Lactate is significantly reduced, whereas creatine, phosphocreatine, and choline are markedly elevated, reflecting metabolic reprogramming associated with seizure activity[138].

In epilepsy, although the focus of multi-omics research has largely been on pathogenesis and diagnosis, relatively few studies have explored the therapeutic potential of these approaches.

Multi-omics technologies integration

Integrative proteomic and transcriptomic analyses have uncovered *miR-10a-5p*, *miR-21a-5p*, and *miR-142a-5p* as important microRNA transcripts associated with epileptogenesis, primarily through the *TGF-β* signaling pathway. Notably, anti-miR therapies targeting these miRNAs have shown protective effects in both acute and spontaneous seizure models, underscoring their potential for therapeutic application in modulating seizure activity and signaling networks[139].

The pathogenesis of epilepsy is highly complex, involving multilayered biological processes. Different types of epilepsy may exhibit distinct etiologies and mechanisms, complicating both research and therapeutic development. Furthermore, significant clinical heterogeneity among patients adds another layer of difficulty to studying the disease. Multi-omics studies in epilepsy generate vast amounts of data across genomic, transcriptomic, epigenomic, and other molecular levels. These insights extend beyond pathogenesis to offer novel diagnostic biomarkers—like *miR-10a-5p* and *ALDH2*—and reveal new therapeutic targets, exemplifying how multi-omics bridges mechanistic discovery and precision medicine in epilepsy. Integrating and analyzing these datasets require high-performance computing resources as well as systematic and statistically robust analytical frameworks and mathematical models. A major ongoing challenge lies in the development of efficient computational strategies to process and interpret these complex, high-dimensional data.

In summary, basic-omics and multi-omics approaches have significantly contributed to elucidating the molecular complexity of epilepsy. These technologies have identified critical genes, proteins, and metabolites that are linked to seizure activity, neuroinflammation, and metabolic dysregulation. Although most research has focused on diagnostic and mechanistic exploration, the therapeutic potential of multi-omics-based strategies is increasingly being recognized.

3.4 Application of Multi-Omics Technologies in MS

MS is a chronic, immune-mediated, inflammatory demyelinating disease of the CNS, marked by temporal and spatial lesion dissemination[140,141]. While the exact etiology remains unclear, risk factors such as Epstein-Barr virus infection, low vitamin D levels, limited sunlight exposure, smoking, and high adolescent BMI have been linked to MS development[142,143]. Clinical symptoms are diverse, including visual impairment, paresthesia, muscle weakness, and progressive disability[144]. A major feature is the presence of oligoclonal bands (OCBs) in CSF, indicating CNS inflammation. Although OCBs are a classical biomarker for MS, their lack of specificity-as they appear in other CNS inflammatory diseases-limits their diagnostic utility[145]. A major challenge in MS research is understanding how immune, inflammatory, and degenerative processes interact across disease stages. Traditional methods offered limited insight into system-wide changes, hindering biomarker discovery and targeted therapy development. The complex and heterogeneous mechanisms of MS remain incompletely understood, impeding targeted therapeutic development. Multi-omics technologies offer a comprehensive approach to uncover molecular underpinnings and identify novel biomarkers and therapeutic targets.Multi-omics approaches have revolutionized MS research by integrating spatial, single-cell, and molecular data. These technologies reveal key cell subtypes, lesion-specific biomarkers, and critical pathways such as *APOE-TREM2* and *CXCL12-CXCR4*. These advances are paving the way for improved stratification mechanisms and precision medicine interventions in MS.

3.4.1 Basic-Omics Approaches in MS

Basic-omics studies have made substantial contributions to the diagnostic and therapeutic landscape of MS. A study involving over 20,000 MS patients has definitively established cells of

the central nervous system—particularly inhibitory neurons—as pivotal cellular contributors to MS susceptibility. Specific risk variants, such as those influencing the *STAT3* and *IL7* genes, were found to affect gene expression exclusively within inhibitory neurons, revealing that cell-intrinsic dysregulation of immune-related pathways within neurons directly contributes to MS pathogenesis. These findings imply that future preventive and therapeutic strategies must incorporate interventions targeting CNS-specific pathways, including neuronal and glial functions, moving beyond solely immunosuppressive approaches[146]. In proteomic profiling, several proteins have been identified as potential biomarkers, including CXCL13, LTA, FCN2, ICAM3, LY9, SLAMF7, TYMP, CHI3L1, FYB1, TNFRSF1B, and NFL. Notably, lower levels of NFL in CSF show predictive potential for disease activity (AUC = 0.77)[147]. In the metabolomic domain, dopamine receptor D2 (DRD2) has been shown to exacerbate MS by promoting inflammation and reducing Lactobacillus abundance in the gut microbiome. Conversely, Lactobacillus-derived N2-acetyl-L-lysine has anti-neurodegenerative effects by inhibiting microglial activation[148]. In relapsing-remitting multiple sclerosis (RRMS), metabolomics has identified four dysregulated metabolic pathways, with glycolysis serving as a common upstream driver. Targeting glycolysis in experimental autoimmune encephalomyelitis ameliorated the disease pathology by impeding immune cell effector function[149].

3.4.2 High-spatial-resolution Omics Technologies in MS

High-spatial-resolution and single-cell omics technologies have provided unprecedented insights into cellular and molecular heterogeneity in MS, particularly in active lesions and periplaque regions involving both CNS and peripheral immune compartments. Single-cell transcriptomic analysis of CSF has revealed a compartmentalized immune landscape, with

enrichment of myeloid dendritic cells and regulatory T cells. A notable finding is the cluster-independent expansion of T follicular helper (TFH) cells, which is associated with increased B-lineage cell infiltration into the CNS and worsened disease severity in MS animal models. These results underscore the local T/B cell crosstalk as a critical driver of MS pathology[150].

Spatial transcriptomics profiling techniques, such as in situ sequencing (10x Xenium) and scRNA-seq, have enabled the spatial mapping of active MS lesion evolution. Astrocytes were categorized into three distinct functional states: homeostatic, intermediate, and disease-associated. Disease-associated astrocytes showed marked upregulation of *SERPINA3,* a gene strongly correlated with active lesion areas in MS. This expression pattern may reflect a glioprotective response aimed at resolving inflammation and preventing apoptosis during early and late lesion resolution phases, and *SERPINA3* has been proposed as a spatial biomarker for MS pathology[151].

Integration of scRNA-seq and spatial transcriptomics has identified perturbations in *KLF/SP* regulatory drivers in oligodendrocytes, including enhanced iron uptake, expression of pro-inflammatory molecules near axonal injury sites, and the role of *MAFB*, an inflammatory transcription factor, as a hallmark of MS lesions[152]. These studies highlight the complex interplay between complement factors, apolipoproteins, and immune cells, as well as a distinct *APOE-TREM2* axis involved in lesion repair.

Multi-omics integration—combining spatial transcriptomics (10x Visium), scRNA-seq, and spatial proteomics (imaging mass cytometry, IMC)—has uncovered the fibrogenic niche in systemic MS skin lesions, driven by a dynamic fibroblast-macrophage axis through the *ACKR3-CXCL12-CXCR4* signaling pathway. Pharmacological inhibition of *CXCR4* using AMD3100 significantly reduced dermal and pulmonary fibrosis, as well as myofibroblast accumulation, in preclinical

models, validating this pathway as a potential therapeutic target. Importantly, the markedly elevated *POSTN/SCARA5* ratio in MS lesions exhibits strong potential as a predictive diagnostic biomarker for disease progression[153].

3.4.3 Multi-Omics Integration in MS

Multi-omics integration has enhanced the comprehensive understanding of MS pathophysiology and expanded the identification of robust biomarkers (Supplementary Table S4). Combined proteomics and transcriptomics have revealed *GPR37L1*, *SIRPA*, *FGFR3*, *CADM3*, and *TYRO3* as highly expressed candidate molecules in the CNS, associated with early neuronal degeneration and impaired trophic and anti-inflammatory intercellular communication, supporting their use as diagnostic tools[154]. Additionally, 24 iron death-related genes (e.g., *CHMP5, SLC38A1, PML*) have been linked to neuroinflammatory processes, where high iron death scores correlate with phagocytic activation at lesion margins and neurological dysfunction in cortical neurons. A blood-based model incorporating these genes has shown prognostic value for MS diagnosis[155]. An integrative approach combining proteomics and metabolomics has identified LAMP1, FCG2A, and heparinase (HPSE) as potential specific biomarkers for MS, with HPSE showing strong correlations with metabolites such as L-tyrosine, sphingosine 1-phosphate, and L-tryptophan[156]. Moreover, another study has also identified reduced levels of anti-inflammatory molecules and sphingolipids, as well as low equine uric acid in severe MS subgroups, pointing to their potential in biomarker development and targeted therapeutic strategies[157].

Multi-omics technologies have systematically uncovered key mechanisms in MS, including dysregulated $CD4^{+}$ T cell–B cell crosstalk, impaired *TREM2-DAP12* signaling in microglia, and disrupted differentiation of oligodendrocyte precursor cells. While potential therapeutic targets such

as S*EMA4D* and *CXCL13* have been identified, their functional roles in remyelination and immune infiltration require further validation through in vivo gene editing and organoid models. Critical challenges remain in predicting the transition from relapsing-remitting to secondary progressive MS, deciphering compartmentalized inflammation within chronic lesions, and elucidating mechanisms of resistance to B cell-targeted therapies. Future efforts should prioritize longitudinal multi-omic profiling of well-annotated conversion-phase cohorts, integration of digital pathology with real-time wearable sensor data, and application of artificial intelligence—such as graph neural networks and multimodal learning—to uncover clinically actionable biomarkers and enable precision medicine interventions.

Currently, our understanding of the relationship between multi-omics profile alterations in MS patients and the underlying molecular networks contributing to MS pathogenesis remains limited. One study outlines a potential integrated multi-omics mechanism that may regulate peripheral immune-inflammatory responses and the progression of MS. These findings can facilitate the development of novel auxiliary diagnostic biomarkers and therapeutic strategies for MS. The summary information is shown in Supplementary Table S4.

3.5 Application of Multi-Omics Technologies in Stroke

Stroke, or cerebrovascular accident, is the most severe neurological disorder to date, causing approximately 160 million years of healthy life lost annually. It is mainly classified into ischemic stroke (IS, ~87%) and hemorrhagic stroke (HS), resulting from vascular injury and leading to focal or global brain damage. Common symptoms include hemiplegia, facial paralysis, and speech impairment, with severe cases progressing to sudden loss of consciousness. Stroke is characterized by high incidence, disability, and mortality, underscoring the urgent need for improved prevention

and treatment strategies. High-throughput technologies now offer new opportunities to unravel its complex pathophysiological mechanisms[158,159].

A major challenge in stroke research is grasping the spatiotemporal dynamics of molecular and cellular responses to acute injury and recovery. Before multi-omics, studies were confined to single pathways or cell types, missing system-wide interactions across key networks and hindering the discovery of reliable biomarkers and neuroprotective strategies, especially early post-stroke. Multi-omics have revolutionized this by enabling high-resolution, integrated profiling of transcriptional, proteomic, and metabolic changes in a cell- and region-specific way. This has revealed key mechanisms like microglia-endothelial crosstalk and immune-metabolic axes (e.g., *SPP1-CD44*), and identified novel biomarkers and targets (e.g., *LILRB4, LGALS9*) for stroke treatment.

3.5.1 Basic-Omics Approaches in Stroke

Basic-omics studies have identified key biomarkers and pathways associated with stroke (Supplementary Table S5). Proteomic profiling has revealed elevated levels of NSF, RhoGDI1, RabGDI, CKB, and CMPK in the circulation of IS patients, reflecting neuronal excitotoxicity and energy metabolism disruption[160,161]. Additionally, SAHH2 increased expression of SAHH2 in neurons from the infarcted area, probably because of ischemia-triggered Ca2+ mobilization[162].

Transcriptomic analyses have identified differentially expressed long non-coding RNAs (lncRNAs), such as *MEG3, H19,* and *MALAT1*, and extracellular microRNAs including *miR-32-3p*, *miR-106b-5p, miR-423-5p,* and *miR-4739*. These molecules are involved in apoptosis, oxidative stress, angiogenesis, and neurogenesis[163-168]. Comprehensive RNA-sequencing analysis revealed that transcriptional responses to ischemic stroke occur in a similar direction but with markedly different magnitude in aged compared to young mouse brains.

Specifically, aged mice exhibited downregulation of genetic programs associated with axonal and synaptic integrity, coupled with significant upregulation of the type I interferon (*IFN-I*) signaling pathway. This enhanced *IFN-I* response was primarily driven by microglia and oligodendrocytes. The increased vulnerability to stroke in aged brains was largely attributable to excessive activation of pro-inflammatory pathways rather than a failure of neurooprotective mechanisms[169].

Metabolomic studies indicate that reduced levels of BCAA are associated with cardioembolic stroke and poor neurological outcomes, highlighting their potential as diagnostic and prognostic biomarkers[170]. Additionally, total plasma free fatty acid levels are significantly elevated in cardioembolic stroke patients compared to those with non-cardioembolic stroke, further suggesting their value as diagnostic targets[171].

3.5.2 High-spatial-resolution Omics Technologies in Stroke

High-spatial-resolution omics technologies have revealed critical molecular and cellular mechanisms underlying stroke pathophysiology and recovery, particularly in aging-related neuroinflammation and myelin repair. scRNA-seq studies have shown that aging impairs paracrine communication between microglia/macrophages and endothelial and oligodendrocyte precursor cells, thereby hindering angiogenesis and remyelination. Transplantation of young microglia/macrophages into aged stroke models partially restores these processes and sensorimotor function, highlighting these cells as potential therapeutic targets for stroke recovery[172].

Modulating *LILRB4* signaling and its downstream effectors emerges as a promising therapeutic approach for IS, as spatial transcriptomics and scRNA-seq have identified a stroke-associated microglial cluster 3 and revealed significant upregulation of *LILRB4* expression in ischemic brain

regions. Functional studies show that *LILRB4* deficiency exacerbates ischemic injury through increased CD8+ T cell recruitment, while its overexpression exerts neuroprotective effects, underscoring the therapeutic potential of modulating *LILRB4* and its downstream pathways in mitigating immune-mediated damage[173]. In contrast, Li et al. investigated brainstem stroke and found that oligodendrocyte loss leads to neurological deficits, followed by regenerative attempts. They identified a sub-cluster of Pros1+ oligodendrocytes, termed *OLG8*, and demonstrated that Myo1e overexpression promotes *OLG8* differentiation, reduces ischemic damage, and enhances neurological recovery, offering cell-type-specific therapeutic avenues[174]. Gu et al. further uncovered the dynamic immune landscape in post-stroke brains, revealing distinct differentiation patterns between myeloid and lymphoid cell populations and spatially localized interactions. Notably, a *SPP1*-high lymphocyte sub-cluster was found to interact with Lyz2+ macrophage-associated lymphocytes, while in the choroid plexus, Lgmn+ macrophage-T cell communication via the *SPP1-CD44* axis was observed during the acute phase of intracerebral hemorrhage. These findings establish *SPP1* and *Lyz2* as potential biomarkers for stroke diagnostics[175]. Further, they identified ferroptosis as the dominant form of programmed cell death occurring as early as one hour post-intracerebral hemorrhage, primarily in mature oligodendrocytes. They showed that a CSF1/CSF1R-mediated interaction between Lipocalin-2+ microglia and oligodendrocytes drives ferroptosis and functional deterioration, suggesting that early inhibition of microglial Lipocalin-2 may protect oligodendrocytes and mitigate post-stroke deficits, representing a novel neuroprotective strategy[176]. In IS models, Han et al. revealed elevated galectin (*LGAL*) signaling in microglia and macrophages, and found that *LGALS9* administration promotes oligodendrocyte remyelination and functional recovery, supporting its therapeutic utility in stroke[177]. In subarachnoid hemorrhage

(SAH), Wang et al. demonstrated that mLV injury is induced by SAH, with *THBS1* and *S100A6* showing marked upregulation post-injury. They identified the *THBS1-CD47* ligand-receptor axis as a key driver of meningeal lymphatic endothelial apoptosis through *STAT3/BCL2* signaling, suggesting that targeting this axis could preserve mLV integrity and improve clinical outcomes[178].

A study utilizing spatial transcriptomics and GWAS compared different regions along the hemodynamic direction of human carotid artery plaques and found that plaque rupture primarily occurs in the proximal and most stenotic areas. Key genes such as *MMP-9* were identified to have a causal relationship with rupture risk. The findings provide a theoretical basis for precise interventions targeting *MMP-9* and other relevant targets in stroke patients[179]. A recent study integrating high-resolution omics technologies—including single-cell transcriptomics, proteomics, and spatial transcriptomics—revealed that proximal astrocytes exhibit divergence in lipid handling, with enriched expression of *APOE* and *FABP5* following cortical ischemic stroke. The data reported here demonstrate the acquisition of distinct astrocyte states post-stroke, governed by both temporal and spatial proximity to the lesion, while demonstrating the utility of a high-content single-cell tool termed tDISCO, which identified both the spatial boundaries and molecular profiles defining these distinct populations[180].

3.5.3 Multi-Omics Integration in Stroke

Multi-omics integration has enabled the identification of complex molecular interactions and novel therapeutic targets in stroke (Supplementary Table S5). In CES, combined proteomic and transcriptomic approaches have uncovered *ICA1L*, *CAND2*, and *ALDH2* as potential biomarkers related to excitatory synaptic dysfunction[181]. In the therapeutic context, integrated genomics and metabolomics have shown that Zhilong Huoxue Tongyu Capsules (ZHTC), a traditional Chinese

medicin, can modulate gut microbiota and metabolic profiles, including arginine, lysine, and methionine, and enhance intestinal barrier integrity[182]. Furthermore, multi-omics and network pharmacology studies on Yiqitongluo Granule (YQTL) have revealed 15 active components that regulate 82 targets across 19 pathways, with *PI3K-Akt*, *MAPK*, and *cAMP* signaling playing central roles in neuroprotection against cerebral ischemia-reperfusion injury[183].

While multi-omics technologies hold promise for advancing precision medicine in stroke, addressing critical challenges—including pronounced disease heterogeneity, complex repair mechanisms, and difficulties in translating omic findings into clinical applications—remains essential to fully realize their potential. It is crucial to validate biomarkers and therapeutic targets across diverse stroke populations and to develop targeted delivery systems for neuroprotective agents. However, current stroke multi-omics datasets remain limited in scale and diversity, and integrating multimodal data is complicated by missing values, batch effects, and lack of standardization. Future research should prioritize longitudinal multi-omics profiling to capture dynamic disease progression, incorporate single-cell and spatial technologies to resolve cell- and region-specific responses, and leverage artificial intelligence for integrated analysis of multimodal data.

In summary, basic-omics studies have provided critical insights into the biomarkers and pathophysiological mechanisms of stroke, particularly in IS and cardioembolic stroke. Proteomic, transcriptomic, and metabolomic approaches have identified key proteins, non-coding RNAs, and metabolic alterations associated with disease onset, progression, and outcomes. Meanwhile, multi-omics integration has further revealed molecular networks and therapeutic targets, demonstrating the potential of systems biology in advancing stroke diagnosis and treatment.

3.6 Application of Multi-Omics Technologies in Hydrocephalus

Hydrocephalus is a potentially fatal neurologic disorder affecting individuals across the lifespan[184]. It is characterized by the abnormal accumulation of CSF due to disruption of its circulation, resulting in ventricular dilation and frequently elevated intracranial pressure (ICP)[185]. Based on CSF flow dynamics, the disease is classified into three main types: obstructive, communicating, and normal pressure hydrocephalus (NPH)[186]. Despite modest progress in surgical techniques over the past five decades, preventive and curative strategies remain limited. Current diagnostic methods-Hakim's triad, computed tomography(CT) and magnetic resonance imaging(MRI), lumbar puncture, and lumbar drainage-are subjective, invasive, and lack specificity, especially in differentiating NPH from vascular or AD-related dementias, due to incomplete understanding of its pathogenesis[184,187]. Likewise, pharmacological therapies have not produced effective treatment options so far[188-190].

Multi-omics technologies integrate genomic, proteomic, and metabolomic data to systematically dissect molecular networks underlying hydrocephalus, enabling the discovery of novel biomarkers such as *CSPG4* to improve early diagnosis and dynamic monitoring of disease progression. These approaches further uncover central pathophysiological mechanisms—including choroid plexus inflammation and ciliary dysfunction—and identify potential therapeutic targets within dysregulated signaling pathways (e.g., TGF-β, VEGF) and metabolic perturbations, thereby providing new avenues for developing precision interventions.

In this context, multi-omics technologies are emerging as promising tools for elucidating the molecular mechanisms, identifying novel biomarkers, and developing targeted, non-invasive therapeutic strategies for hydrocephalus.

Multi-omics technologies integration

3.6.1 Basic-Omics Approaches in Hydrocephalus

Basic-omics studies have identified key molecular signatures associated with the pathogenesis and clinical manifestations of hydrocephalus, particularly communicating hydrocephalus (CH) and idiopathic normal pressure hydrocephalus (iNPH) (Supplementary Table S6). Genomics in CH has revealed *TRIM71* and *SMARCC1* as genes with genome-wide significant de novo mutations, potentially serving as genetic risk factors. Additionally, *PIK3CA, PTEN, MTOR, FOXJ1, FMN2, PTCH1,* and *FXYD2* have been identified as high-confidence sporadic CH-associated genes, with *TRIM71* deletion linked to reduced neural cell proliferation, making it a potential diagnostic marker[191].

Proteomic profiling has identified kallikrein-6 (KLK6) as significantly upregulated in CH patients, implicating it in disease progression and suggesting its utility in diagnostic strategies[192]. In iNPH, 39 upregulated and 285 downregulated proteins in CSF have been observed, with elevated glutaminyl-peptide cyclotransferase (QPCT) and retinol-binding protein 4 (RBP4) levels showing prognostic and diagnostic relevance for shunt response[193]. Notably, Q-type protein tyrosine phosphatase receptor (PTPRQ) is significantly higher in iNPH compared to AD and may help distinguish iNPH from AD-related dementia[194].

Metabolomic studies in iNPH and NPH have uncovered CSF metabolic profiles that aid in differential diagnosis and treatment response prediction. In iNPH, elevated glyceric acid and N-acetylneuraminic acid (Neu5Ac), along with reduced serine and 2-hydroxybutyric acid, form a diagnostic signature that differentiates it from AD[195]. In NPH, low CSF Neu5Ac levels are associated with astrocyte activation and periventricular demyelination. Elevating brain Neu5Ac has been shown to improve neurological outcomes, indicating its therapeutic potential[196].

3.6.2 High-spatial-resolution Omics Technologies in Hydrocephalus

A recent study utilizing snRNA-seq and spatial transcriptomics in a tumor-associated hydrocephalus (TAH) mouse model revealed the expansion of choroid plexus mast cells (*CPMCs*) in the ventricular region, including the choroid plexus and ependymal walls. These *CPMCs* contribute to TAH pathogenesis by disrupting ciliated epithelial cells through the tryptase-PAR2-FOXJ1 signaling axis, thereby enhancing CSF production. Importantly, elevated CSF tryptase levels have been associated with increased clinical severity of TAH, and administration of the brain-penetrant tryptase inhibitor *BMS-262084* significantly attenuated TAH in vivo and protected against ciliary damage in human stem cell-derived choroid plexus organoids. These results identify *CPMCs* as key drivers of TAH and highlight *BMS-262084* as a potential therapeutic candidate[197].

By integrating genomic and scRNA-seq approaches, a study systematically characterizes the evolutionary, temporal, and spatial expression profiles of maelstrom spermatogenic transposon silencer (*MAEL*) in the developing human brain, and functionally validates its expression in human hydrocephalic brain tissue. scRNA-seq analyses of the cortical plate and germinal matrix regions identified robust *MAEL* expression within neurogenic niches, wherein reduced *MAEL* expression may induce large-scale genomic rearrangements that disrupt cortical development, volume, and function. These findings suggest that diminished *MAEL* expression may contribute to the pathogenesis of hydrocephalus via multiple etiological pathways[198].

3.6.3 Multi-Omics Integration in Hydrocephalus

Multi-omics integration has enabled a systems-level understanding of hydrocephalus and the identification of novel diagnostic and therapeutic biomarkers through cross-omics validation and functional annotation (Supplementary Table S6). In response, multi-omics joint analysis has

Multi-omics technologies integration

emerged as a crucial method for revealing therapeutic targets and refining diagnostic methods by integrating and comprehensively analyzing data from different omics layers (Supplementary Table S6). Integrated genomics, proteomics, and transcriptomics in CH have highlighted the maelstrom *MAEL* as a candidate diagnostic biomarker. Reduced *MAEL* expression in multiple brain regions is significantly associated with hydrocephalus, and PrediXcan analysis confirms its pathophysiological relevance[199]. In post-hemorrhagic hydrocephalus, the combined use of proteomics and metabolomics has identified chondroitin sulfate proteoglycan 4 (CSPG4) as a promising CSF biomarker. CSPG4 is positively correlated with ventricular size and the incidence of periventricular leukomalacia. Functional studies indicated that CSPG4 silencing can inhibit ferroptosis, cell adhesion, and intracellular Ca^{2+} flux, supporting its role in both diagnosis and treatment[200].

However, multi-omics studies in hydrocephalus face considerable challenges due to the relative difficulty in obtaining CSF samples, which are often limited in volume. Furthermore, the low abundance of proteins and metabolites in CSF necessitates highly sensitive detection technologies. Preanalytical variables during sample collection, processing, and storage can also significantly affect data accuracy and reproducibility. Critically, the scarcity of these precious samples has resulted in a notable paucity of high-quality omics datasets in hydrocephalus, making existing data exceptionally valuable. Overcoming these barriers will require intensified research efforts, standardized protocols, and enhanced support from funding agencies and policymakers to facilitate larger-scale sample acquisition and multi-institutional collaboration.

Although multi-omics approaches have unveiled a plethora of candidate targets and pathways for hydrocephalus therapy, definitive clinical breakthroughs in therapeutic development remain

elusive. These findings not only enhance our comprehension of the pathological processes of hydrocephalus but also lay a theoretical foundation for the future development of targeted therapies directed at specific pathways or molecules.

4. Current Challenges and Future Trends

Although multi-omics technologies have advanced our understanding of neurological disease pathophysiology, their full clinical translation is hindered by significant technical, analytical, and translational challenges. These are particularly evident in high-spatial-resolution omics approaches, such as scRNA-seq and spatial transcriptomics, which provide cell-type-specific and spatially resolved insights but also introduce additional layers of complexity in data generation, integration, and interpretation.

4.1 Data Complexity in Multi-omics and High-Spatial-Resolution Omics Integration

Omics data is inherently high-dimensional, heterogeneous, and multi-modal, posing significant challenges in standardization, integration, and biological interpretation. The lack of universally accepted data formats and protocols across institutions leads to inconsistent data quality and poor interoperability, especially when integrating genomics, transcriptomics, and proteomics, which vary in dynamic ranges and measurement scales[201]. Technical variability in sample preparation and instrument performance further introduces noise, missing values, and batch effects, necessitating robust preprocessing strategies, such as missing value imputation and outlier detection, to ensure data reliability[202-204].

High-spatial-resolution omics technologies, such as scRNA-seq and spatial transcriptomics, exacerbate these challenges by capturing cell-type-specific and spatially resolved molecular profiles.

Multi-omics technologies integration

These approaches generate ultra-high-spatial-resolution datasets with substantial cellular and spatial heterogeneity, requiring advanced computational pipelines for clustering, trajectory inference, and cell-type annotation. Spatial transcriptomics, in particular, introduces spatial coordinates, thereby demanding novel algorithms that can integrate molecular and spatial information simultaneously[205]. The absence of standardized annotation systems and centralized data repositories for high-spatial-resolution data remains a major bottleneck, especially in complex brain regions such as the cortex, hippocampus, and choroid plexus, where cellular diversity and spatial architecture are particularly pronounced[206].

Given these challenges, the emergence of artificial intelligence (AI), particularly ML and deep learning, has opened new avenues for multi-omics data integration. ML algorithms can extract key features and identifying underlying patterns from high-dimensional and heterogeneous datasets, offering critical support for biomarker discovery and mechanistic elucidation[207]. For instance, DL-based approaches have demonstrated remarkable success in early AD diagnosis by analyzing integrated multi-omics and network data, significantly improving diagnostic accuracy[208]. Moreover, AI-driven multi-omics analysis not only enhances the understanding of neurodegenerative disease mechanisms but also accelerates the identification of novel druggable targets and supports the development of disease-specific biomarkers, ultimately improving treatment outcomes[209].

4.2 Challenges in Biomarker Validation and Clinical Translation

The validation of neurological disease biomarkers remains a critical translational hurdle, particularly in the context of multi-omics and high-spatial-resolution omics. Traditional bulk omics methods often average out cellular and inter-individual heterogeneity, thereby obscuring

biologically relevant signals that are cell-type- or region-specific. In contrast, scRNA-seq and spatial transcriptomics provide detailed molecular profiles at the cellular and spatial level, yet their technical complexity and data variability pose significant challenges for robust clinical validation. A major limitation is the lack of standardized, reproducible, and scalable validation frameworks that can retain the resolution and biological context of high-dimensional and multi-modal data. Most existing protocols are optimized for bulk-level analysis, which fails to preserve the cellular and spatial information essential for identifying cell-type- or region-specific biomarkers. As a result, many high-spatial-resolution biomarkers may not be detectable or reproducible in standard clinical assays, where resolution is lower and biological noise is higher. For instance, S100B, a protein detectable in stroke patients, lacks sufficient specificity due to its expression in healthy individuals and in other neurological conditions[210]. This underscores the need for more rigorous validation strategies that account for inter-individual variability driven by genetic, epigenetic, and environmental factors. Such variability can mask or distort molecular signatures, requiring large-scale, well-phenotyped, and multi-omics-annotated cohorts for robust biomarker discovery and cross-population validation. Furthermore, the clinical translation of multi-omics findings is hindered by limited clinician engagement and inadequate integration with clinical workflows.

To overcome these barriers, a comprehensive and standardized validation framework is essential. This framework should incorporate high-spatial-resolution experimental validation, multi-omics data harmonization, and clinically annotated reference datasets to ensure reproducibility and generalizability across diverse populations and clinical settings. Moreover, collaboration across disciplines—including computational biology, neurology, and bioinformatics—is necessary to align research findings with clinical needs and to develop practical, scalable solutions for the translation

of high-spatial-resolution omics biomarkers into routine clinical diagnostics and personalized treatment strategies.

4.3 Multi-modal Integration of Multi-omics with Clinical Imaging and Real-Time Sensing

A key future direction in neurological disease research lies in the integration of multi-omics and high-spatial-resolution omics data with clinical imaging and real-time sensing technologies. This multi-modal strategy enables the bridging of molecular insights with anatomical and functional information, thereby enhancing mechanistic understanding at multiple biological scales. For example, in the research of AD, by utilizing MRI to obtain brain structural images and integrating metabolomics data, it becomes possible to identify abnormal metabolite changes in specific brain regions[211]. Such integrative frameworks are critical for connecting molecular heterogeneity with clinical phenotypes, improving diagnostic accuracy, and enabling personalized therapeutic interventions.

Despite its promise, this approach faces several technical and methodological challenges. First, the heterogeneous nature of omics, imaging, and real-time sensor data complicates data alignment and integration. Second, standardized protocols for multi-modal validation are still in early development, which limits reproducibility and clinical translation. Third, real-time data from wearable biosensors, while offering continuous monitoring and high temporal resolution, pose new computational demands when fused with static omics data, requiring novel methods for dynamic profiling and predictive modeling.

Moving forward, the development of unified platforms that support seamless integration of multi-omics, imaging, and real-time sensing will be essential for advancing precision neurology.

Multi-omics technologies integration

These platforms should incorporate AI-driven analysis, adaptive data fusion strategies, and clinically validated biomarker pipelines to facilitate early detection, individualized treatment planning, and longitudinal disease tracking.

5. Conclusion

Neurological diseases, characterized by their high incidence, high disability rate, and severe impact on patients' quality of life, have emerged as a significant global health challenge that urgently demands solutions. While traditional methods have laid the groundwork for understanding disease mechanisms, the integration of multi-omics and high-spatial-resolution omics technologies now provides comprehensive and spatially resolved molecular insights. Studies on major neurological disorders, including AD, PD, MS, stroke, and hydrocephalus, have identified numerous disease-associated genes, proteins, metabolites, and pathways. These discoveries deepen our understanding of pathogenesis, highlight key drivers of disease progression, and offer potential biomarkers and therapeutic targets for early detection, precision treatment, and prognosis.

Despite these advances, the application of multi- and high-spatial-resolution omics in this field remains challenging, particularly in data integration, standardization of validation protocols, and translation to clinical settings. Addressing these issues is critical for harnessing the full potential of these technologies in neurological disease research. Looking ahead, the continued development of open-source platforms will enhance the adoption and utility of multi- and high-spatial-resolution omics approaches. This progress is expected to accelerate mechanistic discovery, improve diagnostic accuracy, and support individualized therapies, ultimately advancing precision neurology and translational medicine.

Multi-omics technologies integration

Additional Files

Supplementary Table S1. Application of Multi-omics and High-spatial-resolution Omics Technologies in AD.

Supplementary Table S2. Application of Multi-omics and High-spatial-resolution Omics Technologies in PD.

Supplementary Table S3. Application of Multi-omics and High-spatial-resolution Omics Technologies in Epilepsy.

Supplementary Table S4. Application of Multi-omics and High-spatial-resolution Omics Technologies in MS.

Supplementary Table S5. Application of Multi-omics and High-spatial-resolution Omics Technologies in Stroke.

Supplementary Table S6. Application of Multi-omics and High-spatial-resolution Omics Technologies in Hydrocephalus.

Abbreviations

AI: artificial intelligence; AD: Alzheimer's disease; ADPRC: ADP-ribosyl cyclase; ALDH2: aldehyde dehydrogenase 2; AlzGPS: Alzheimer's disease genome-wide positioning systems platform; BBB: blood-brain-barrier; BCAA: branched-chain amino acid; BHD: Buyang huanwu decoction; CH: communicating hydrocephalus; CNS: central nervous system; *CPMCs*: choroid plexus mast cells; CSF: cerebrospinal fluid; CSPG4: chondroitin sulfate proteoglycan 4; CT: computed tomography; DDA: data-dependent acquisition; DIA/SWATH: data independent acquisition-sequential window acquisition of all theoretical mass spectral approach; DRD2: domain, dopamine receptor D2; FT-IR: fourier-transform infrared; GFAP: glial fibrillary acidic protein; GC-

MS: gas chromatography–mass spectrometry; GABA: γ-aminobutyric acid; GWAS: genome-wide association studies; GSTM1: glutathione S-transferase M1; HS: hemorrhagic stroke; HPSE: heparinase; *H4K16ac*: acetylation status of histone H4 at lysine 16; iNPH: idiopathic normal pressure hydrocephalus; ICP: intracranial pressure; IL-1β: interleukin-1β; IL-6: interleukin-6; IS: ischemic stroke; iTRAQ: isobaric tags for relative and absolute quantification; KLK6: kallikrein-6; LC-MS: liquid chromatography–mass spectrometry; FBTCS: focal to bilateral tonic–clonic seizures; lncRNAs: long non-coding RNAs; LPAR3: lysophosphatidic acid receptor 3; LRRK2: leucine-rich repeat kinase 2; MAEL: maelstrom spermatogenic transposon silencer; *MBP*: Myeloid basic protein encoding gene; ML: machine learning; MS: multiple sclerosis; MTLE: mesial temporal lobe epilepsy; MRI: magnetic resonance imaging; Neu5Ac: N-acetylneuraminic acid; NMR: nuclear magnetic resonance; NPH: normal pressure hydrocephalus; OCBs: oligoclonal bands; PIGs: plaque-induced genes; PD: Parkinson's disease; PKM2: pyruvate kinase M2; PTE: post-traumatic epilepsy; PTPRQ: Q-type protein tyrosine phosphatase receptor; QPCT: glutaminyl-peptide cyclotransferase; RBP4: retinol-binding protein 4; RF: random forest; RNA-seq: RNA sequencing; RRMS: relapsing-remitting multiple sclerosis; SAH: subarachnoid hemorrhag; scRNA-seq: single-cell RNA sequencing; SILAC: stable-isotope labeling by amino acids in cell culture; SNPs: single-nucleotide polymorphisms; SNAP-25: synaptosome-associated protein 25; SNpc: substantia nigra pars compacta; snRNA-seq: single-nucleus RNA sequencing; *SSR1*: signal sequence receptor subunit 1; TAH: tumor-associated hydrocephalus; *TGF-β*: transforming growth factor β; TLE: temporal lobe epilepsy; *TNF-α*: tumor necrosis factor-α; TMT: tandem mass tag; TFH: T follicular helper; UCH-L1: calreticulin, ubiquitin carboxyl-terminal hydrolase L1; YQTL: Yiqitongluo granule; ZHTC: Zhilong huoxue ttongyu capsules.

Declarations

Ethics Approval and Consent to Participate (Not Applicable)

Consent for Publication (Not Applicable)

Data Availability

The datasets in the current study are available by reasonable request to the first author. (xiuyun_liu@tju.edu.cn).

Competing Interests

The authors declare no competing financial interest.

Funding

The study was funded by Scientific Research Innovation Capability Support Project for Young Faculty (ZYGXQNJSKYCXNLZCXM-H15), National Science Fund for Excellent Overseas Scholars (0401260011), National Natural Science Foundation of China (82472098, 32300704), Tianjin Natural Science Foundation-Outstanding Youth Project (24JCJQJC00250), Major Science and Technology Special Projects and Engineering-Major Project of National Key Laboratories (24ZXZSSS00510) and National Key Technologies Research and Development Program (2021YFF1200602), the Non-profit Central Research Institute Fund of Chinese Academy of Medical Sciences (2024-JKCS-16). Part of the figures were created by BioRender (www.biorender.com).

Author's Contributions

Xiuyun Liu: conceptualization; Xiuyun Liu and Fangfang Li: writing-review & editing; Fangfang Li: writing – original draft; Xiuyun Liu, Fangfang Li, Meijun Pang, Marek Czosnyka, and Zofia Czosnyka: methodology; Meijun Pang, Huijie Yu, Xiaoguang Tong, Yan Xing, Hongliang Li,

Ke Pu, Keke Feng, and Kuo Zhang: investigation; Meijun Pang: supervision; Dong Ming: project administration.

Acknowledgements

The authors sincerely acknowledge the financial support for this study from the Scientific Research Innovation Capability Support Project for Young Faculty (ZYGXQNJSKYCXNLZCXM-H15), National Science Fund for Excellent Overseas Scholars (0401260011), National Natural Science Foundation of China (82472098, 32300704), Tianjin Natural Science Foundation-Outstanding Youth Project (24JCJQJC00250), Major Science and Technology Special Projects and Engineering-Major Project of National Key Laboratories (24ZXZSSS00510), National Key Technologies Research and Development Program (2021YFF1200602), and Non-profit Central Research Institute Fund of Chinese Academy of Medical Sciences (2024-JKCS-16). Special thanks are due to BioRender (www.biorender.com) for supporting the creation of part of the study's figures. The authors also appreciate all team members who contributed to study methodology, investigation, supervision, project administration, and manuscript preparation—their efforts were essential to the completion of this research.

Authors' information

Xiuyun Liu, Chair Professor of Tianjin University, Deputy Director of the Medical Department, and Dean of the School of Pharmacy. The research primarily focuses on neurocritical care medicine and critical care brain-computer interface, with a specific emphasis on developing key diagnostic and therapeutic technologies for brain disorders such as reversible dementia (hydrocephalus), dementia, traumatic brain injury, epilepsy, stroke, and postoperative delirium. The overarching goal is to address the current engineering bottlenecks in neurocritical care, providing physicians with

precise treatment methods and strategies. Tianjin University, 92 Weijin Road, Nankai District, Tianjin, 300072, China, Email: xiuyun_liu@tju.edu.cn.

References

1. Steinmetz J D, Seeher, Katrin Maria et al. Global, regional, and national burden of disorders affecting the nervous system, 1990–2021: a systematic analysis for the Global Burden of Disease Study 2021. Lancet Neurol. 2024;23:344-381. https://doi.org/10.1016/s1474-4422(24)00038-3
2. Leinenga G, Langton C, Nisbet R, and Götz J. Ultrasound treatment of neurological diseases — current and emerging applications. Nat Rev Neurol. 2016;12:161-174. https://doi.org/10.1038/nrneurol.2016.13
3. Milligan T A. Diagnosis in neurologic disease. Med Clin North Am. 2019;103:173-190. https://doi.org/10.1016/j.mcna.2018.10.011
4. Jankovic J. Parkinson's disease: clinical features and diagnosis. J Neurol Neurosurg Psychiatry. 2008;79:368-376. https://doi.org/10.1136/jnnp.2007.131045
5. Kuska B. Beer, Bethesda, and biology: how "genomics" came into being. J Natl Cancer Inst. 1998;90:93. https://doi.org/10.1093/jnci/90.2.93
6. Manzoni C, Kia D A, Vandrovcova J, Hardy J, Wood N W, Lewis P A, et al. Genome, transcriptome and proteome: the rise of omics data and their integration in biomedical sciences. Brief Bioinform. 2018;19:286-302. https://doi.org/10.1093/bib/bbw114
7. Karczewski K J, and Snyder M P. Integrative omics for health and disease. Nat Rev Genet. 2018;19:299-310. https://doi.org/10.1038/nrg.2018.4
8. Hasin Y, Seldin M, and Lusis A. Multi-omics approaches to disease. Genome Biol. 2017;18:83. https://doi.org/10.1186/s13059-017-1215-1
9. Bujak R, Struck-Lewicka W, Markuszewski M J, and Kaliszan R. Metabolomics for laboratory diagnostics. J Pharm Biomed Anal. 2015;113:108-120. https://doi.org/10.1016/j.jpba.2014.12.017
10. C. F R. Epigenetics: The ultimate mystery of inheritance. W. W. Norton & Company; 2011.
11. Qian Z, Qin J, Lai Y, Zhang C, and Zhang X. Large-Scale Integration of Single-Cell RNA-Seq Data Reveals Astrocyte Diversity and Transcriptomic Modules across Six Central Nervous System Disorders. Biomolecules. 2023;13. https://doi.org/10.3390/biom13040692
12. Shapiro E, Biezuner T, and Linnarsson S. Single-cell sequencing-based technologies will revolutionize whole-organism science. Nat Rev Genet. 2013;14:618-30. https://doi.org/10.1038/nrg3542
13. Close J L, Long B R, and Zeng H. Spatially resolved transcriptomics in neuroscience. Nature methods. 2021;18:23-25. https://doi.org/10.1038/s41592-020-01040-z
14. Uffelmann E, Huang Q Q, Munung N S, de Vries J, Okada Y, Martin A R, et al. Genome-wide association studies. Nat Rev Methods Primers. 2021;1:59. https://doi.org/10.1038/s43586-021-00056-9
15. Madden E B, Hindorff L A, Bonham V L, Akintobi T H, Burchard E G, Baker K E, et al. Advancing genomics to improve health equity. Nat Genet. 2024;56:752-757. https://doi.org/10.1038/s41588-024-01711-z
16. iétu G M-S R, Fayein NA, Matingou C, Eveno E, Houlgatte R, Decraene C, Vandenbrouck Y, Tahi F, Devignes MD, Wirkner U, Ansorge W, Cox D, Nagase T, Nomura N, Auffray C. The Genexpress

IMAGE knowledge base of the human brain transcriptome prototype integrated resource for functional and computational genomics. Genome Res. 1999;9:195 – 209. https://doi.org/10.1101/gr.9.2.195

17. Lowe R, Shirley N, Bleackley M, Dolan S, and Shafee T. Transcriptomics technologies. Plos Comput Biol. 2017;13. https://doi.org/10.1371/journal.pcbi.1005457
18. Lebrigand K, Bergenstråhle J, Thrane K, Mollbrink A, Meletis K, Barbry P, et al. The spatial landscape of gene expression isoforms in tissue sections. Nucleic Acids Res. 2023;51:e47-e47. https://doi.org/10.1093/nar/gkad169
19. Kukurba K R, and Montgomery S B. RNA Sequencing and Analysis. Cold Spring Harb Protoc. 2015;2015:951–969. https://doi.org/10.1101/pdb.top084970
20. Godovac-Zimmermann J. 8th siena meeting. From genome to proteome: Integration and proteome completion. Expert Rev Proteomic. 2014;5:769-773. https://doi.org/10.1586/14789450.5.6.769
21. Aggarwal K, Choe Lh Fau - Lee K H, and Lee K H. Shotgun proteomics using the iTRAQ isobaric tags. Brief Funct Genomic Proteomic. 2006;5(2):112-120. https://doi.org/10.1093/bfgp/ell018
22. Sharma K B, Aggarwal S, Yadav A K, Vrati S, and Kalia M. Studying autophagy using a TMT-based quantitative proteomics approach. Methods Mol Biol. 2022;2445:183 – 203. https://doi.org/10.1007/978-1-0716-2071-7_12
23. Chen X, Wei S, Ji Y, Guo X, and Yang F. Quantitative proteomics using SILAC: Principles, applications, and developments. Proteomics. 2015;15:3175-3192. https://doi.org/10.1002/pmic.201500108
24. Souza G, Guest P C, and Martins-de-Souza D. LC-MS(E), multiplex MS/MS, ion mobility, and label-free quantitation in clinical proteomics. Methods Mol Biol. 2017;1546:57 – 73. https://doi.org/10.1007/978-1-4939-6730-8_4
25. Aebersold R, Bensimon A, Collins B C, Ludwig C, and Sabido E. Applications and developments in targeted proteomics: from SRM to DIA/SWATH. Proteomics. 2016;16:2065-2067. https://doi.org/10.1002/pmic.201600203
26. Aebersold R, and Mann M. Mass-spectrometric exploration of proteome structure and function. Nature. 2016;537:347-355. https://doi.org/10.1038/nature19949
27. Nicholson JK, Lindon JC, and E H. Metabonomics understanding the metabolic responses of living systems to pathophysiological stimuli via multivariate statistical analysis of biological NMR spectroscopic data. Xenobiotica. 1999;29:1181–1189. https://doi.org/10.1080/004982599238047
28. Wu X, Ao H, Gao H, and Zhu Z. Metabolite biomarker discovery for human gastric cancer using dried blood spot mass spectrometry metabolomic approach. Sci Rep-uk. 2022;12:14632. https://doi.org/10.1038/s41598-022-19061-3
29. Schrimpe-Rutledge A C, Codreanu S G, Sherrod S D, and McLean J A. Untargeted metabolomics strategies—challenges and emerging directions. J Am Soc Mass Spectr. 2016;27:1897-1905. https://doi.org/10.1007/s13361-016-1469-y
30. Carrillo A J, Halilovic L, Hur M, Kirkwood J S, and Borkovich K A. Targeted metabolomics using LC-MS in neurospora crassa. Curr Protoc. 2022;2:e454. https://doi.org/10.1002/cpz1.454
31. Chen W, Gong L, Guo Z, Wang W, Zhang H, Liu X, et al. A novel integrated method for large-scale detection, identification, and quantification of widely targeted metabolites: application in the study of rice metabolomics. Mol Plant. 2013;6:1769-1780. https://doi.org/10.1093/mp/sst080
32. Tang F, Barbacioru C, Wang Y, Nordman E, Lee C, Xu N, et al. mRNA-Seq whole-transcriptome analysis of a single cell. Nature methods. 2009;6:377-82. https://doi.org/10.1038/nmeth.1315

33. Chen C, Xing D, Tan L, Li H, Zhou G, Huang L, et al. Single-cell whole-genome analyses by Linear Amplification via Transposon Insertion (LIANTI). Science. 2017;356:189-194. https://doi.org/10.1126/science.aak9787
34. Luo C, Fernie A R, and Yan J. Single-Cell Genomics and Epigenomics: Technologies and Applications in Plants. Trends Plant Sci. 2020;25:1030-1040. https://doi.org/10.1016/j.tplants.2020.04.016
35. Wang J, Ye F, Chai H, Jiang Y, Wang T, Ran X, et al. Advances and applications in single-cell and spatial genomics. Sci China Life Sci. 2025;68:1226-1282. https://doi.org/10.1007/s11427-024-2770-x
36. Irish J M, Hovland R, Krutzik P O, Perez O D, Bruserud Ø, Gjertsen B T, et al. Single cell profiling of potentiated phospho-protein networks in cancer cells. Cell. 2004;118:217-28. https://doi.org/10.1016/j.cell.2004.06.028
37. Aldridge S, and Teichmann S A. Single cell transcriptomics comes of age. Nat Commun. 2020;11:4307. https://doi.org/10.1038/s41467-020-18158-5
38. Ståhl P L, Salmén F, Vickovic S, Lundmark A, Navarro J F, Magnusson J, et al. Visualization and analysis of gene expression in tissue sections by spatial transcriptomics. Science. 2016;353:78-82. https://doi.org/10.1126/science.aaf2403
39. Eisenstein M. Seven technologies to watch in 2022. Nature. 2022;601:658-661. https://doi.org/10.1038/d41586-022-00163-x
40. Moffitt J R, Lundberg E, and Heyn H. The emerging landscape of spatial profiling technologies. Nat Rev Genet. 2022;23:741-759. https://doi.org/10.1038/s41576-022-00515-3
41. Bouwman B A M, Crosetto N, and Bienko M. The era of 3D and spatial genomics. Trends Genet. 2022;38:1062-1075. https://doi.org/10.1016/j.tig.2022.05.010
42. Lundberg E, and Borner G H H. Spatial proteomics: a powerful discovery tool for cell biology. Nat Rev Mol Cell Biol. 2019;20:285-302. https://doi.org/10.1038/s41580-018-0094-y
43. Taylor M J, Lukowski J K, and Anderton C R. Spatially Resolved Mass Spectrometry at the Single Cell: Recent Innovations in Proteomics and Metabolomics. J Am Soc Mass Spectrom. 2021;32:872-894. https://doi.org/10.1021/jasms.0c00439
44. Vandereyken K, Sifrim A, Thienpont B, and Voet T. Methods and applications for single-cell and spatial multi-omics. Nat Rev Genet. 2023;24:494-515. https://doi.org/10.1038/s41576-023-00580-2
45. Xia C, Fan J, Emanuel G, Hao J, and Zhuang X. Spatial transcriptome profiling by MERFISH reveals subcellular RNA compartmentalization and cell cycle-dependent gene expression. Proc Natl Acad Sci U S A. 2019;116:19490-19499. https://doi.org/10.1073/pnas.1912459116
46. Takei Y, Yun J, Zheng S, Ollikainen N, Pierson N, White J, et al. Integrated spatial genomics reveals global architecture of single nuclei. Nature. 2021;590:344-350. https://doi.org/10.1038/s41586-020-03126-2
47. Ruffini N, Klingenberg S, Schweiger S, and Gerber S. Common factors in neurodegeneration: A meta-study revealing shared patterns on a multi-omics scale. Cells. 2020;9:2642. https://doi.org/10.3390/cells9122642
48. International A s D. World Alzheimer Report 2019. Alzheimer's Dis. 2019.
49. Association A s. 2019 Alzheimer's disease facts and figures. AlzheimersDement. 2019;15:321-387. https://doi.org/10.1016/j.jalz.2019.01.010
50. Joe E R J. Cognitive symptoms of Alzheimer's disease: clinical management and prevention. BMJ.

2019;367:l6217. https://doi.org/10.1136/bmj.l6217

51. Scheltens P, Blennow K, Breteler M M B, de Strooper B, Frisoni G B, Salloway S, et al. Alzheimer's disease. The Lancet. 2021;397:1577–1590. https://doi.org/10.1016/s0140-6736(15)01124-1
52. Ballard C, Gauthier S, Corbett A, Brayne C, Aarsland D, and Jones E. Alzheimer's disease. The Lancet. 2011;377:1019-1031. https://doi.org/10.1016/s0140-6736(10)61349-9
53. Scheltens P, De Strooper B, Kivipelto M, Holstege H, Chételat G, Teunissen C E, et al. Alzheimer's disease. The Lancet. 2021;397:1577-1590. https://doi.org/10.1016/s0140-6736(20)32205-4
54. Chen C, Wang J, Pan D, Wang X, Xu Y, Yan J, et al. Applications of multi-omics analysis in human diseases. MedComm. 2023;4:e315. https://doi.org/10.1002/mco2.315
55. Braak H B, E. Neuropathological stageing of Alzheimer-related changes. Acta Neuropathologica 1991;82:239–259. https://doi.org/10.1007/BF00308809
56. Eva-Maria Mandelkow E M. Tau in Alzheimer's disease. Trends Cell Biol. 1998;8:425-427. https://doi.org/10.1016/S0962-8924(98)01368-3
57. Iqbal K, and Grundke-Iqbal I. Neurofibrillary pathology leads to synaptic loss and not the other way around in Alzheimer disease. J Alzheimer's Dis. 2002;4:235-238. https://doi.org/10.3233/JAD-2002-4313
58. dos Santos Guilherme M, Todorov H, Osterhof C, Möllerke A, Cub K, Hankeln T, et al. Impact of acute and chronic amyloid-β peptide exposure on gut microbial commensals in the mouse. Front Microbiol. 2020;11:1008. https://doi.org/10.3389/fmicb.2020.01008
59. Peña-Bautista C, Baquero M, Vento M, and Cháfer-Pericás C. Omics-based biomarkers for the early Alzheimer disease diagnosis and reliable therapeutic targets development. Curr Neuropharmacol. 2019;17:630-647. https://doi.org/10.2174/1570159x16666180926123722
60. Hampel H, Nisticò R, Seyfried N T, Levey A I, Modeste E, Lemercier P, et al. Omics sciences for systems biology in Alzheimer's disease: State-of-the-art of the evidence. Ageing Res Rev. 2021;69:101346. https://doi.org/10.1016/j.arr.2021.101346
61. Montagne A, Nikolakopoulou A M, Huuskonen M T, Sagare A P, Lawson E J, Lazic D, et al. APOE4 accelerates advanced-stage vascular and neurodegenerative disorder in old Alzheimer's mice via cyclophilin A independently of amyloid- β . Nat Aging. 2021;1:506-520. https://doi.org/10.1038/s43587-021-00073-z
62. Nativio R, Donahue G, Berson A, Lan Y, Amlie-Wolf A, Tuzer F, et al. Dysregulation of the epigenetic landscape of normal aging in Alzheimer's disease. Nat Neurosci. 2018;21:497-505. https://doi.org/10.1038/s41593-018-0101-9
63. Klein H-U, McCabe C, Gjoneska E, Sullivan S E, Kaskow B J, Tang A, et al. Epigenome-wide study uncovers large-scale changes in histone acetylation driven by tau pathology in aging and Alzheimer's human brains. Nat Neurosci. 2018;22:37-46. https://doi.org/10.1038/s41593-018-0291-1
64. Mostafavi S, Gaiteri C, Sullivan S E, White C C, Tasaki S, Xu J, et al. A molecular network of the aging human brain provides insights into the pathology and cognitive decline of Alzheimer's disease. Nat Neurosci. 2018;21:811-819. https://doi.org/10.1038/s41593-018-0154-9
65. Peng S, Zeng L, Haure-Mirande J V, Wang M, Huffman D M, Haroutunian V, et al. Transcriptomic Changes Highly Similar to Alzheimer's Disease Are Observed in a Subpopulation of Individuals During Normal Brain Aging. Front Aging Neurosci. 2021;13:711524. https://doi.org/10.3389/fnagi.2021.711524
66. Roberts JA, Varma VR, An Y, Varma S, Candia J, Fantoni G, et al. A brain proteomic signature of

incipient Alzheimer's disease in young APOE ε4 carriers identifies novel drug targets. Sci Adv. 2021;7:eabi8178. https://doi.org/10.1126/sciadv.abi817

67. He K, Nie L, Zhou Q, Rahman S U, Liu J, Yang X, et al. Proteomic profiles of the early mitochondrial changes in APP/PS1 and ApoE4 transgenic mice models of Alzheimer's disease. J Proteome Res. 2019;18:2632-2642. https://doi.org/10.1021/acs.jproteome.9b00136
68. Chen C, Jiang X, Li Y, Yu H, Li S, Zhang Z, et al. Low-dose oral copper treatment changes the hippocampal phosphoproteomic profile and perturbs mitochondrial function in a mouse model of Alzheimer's disease. Free Radical Bio Med. 2019;135:144-156. https://doi.org/10.1016/j.freeradbiomed.2019.03.002
69. Shen Y, Timsina J, Heo G, Beric A, Ali M, Wang C, et al. CSF proteomics identifies early changes in autosomal dominant Alzheimer's disease. Cell. 2024;187:6309-6326.e15. https://doi.org/10.1016/j.cell.2024.08.049
70. Brayne C, Varma V R, Oommen A M, Varma S, Casanova R, An Y, et al. Brain and blood metabolite signatures of pathology and progression in Alzheimer disease: A targeted metabolomics study. Plos Med. 2018;15:e1002482. https://doi.org/10.1371/journal.pmed.1002482
71. Traxler L, Herdy J R, Stefanoni D, Eichhorner S, Pelucchi S, Szücs A, et al. Warburg-like metabolic transformation underlies neuronal degeneration in sporadic Alzheimer's disease. Cell Metab. 2022;34:1248-1263. https://doi.org/10.1016/j.cmet.2022.07.014
72. He Y, Lu W, Zhou X, Mu J, and Shen W. Unraveling Alzheimer's disease: insights from single-cell sequencing and spatial transcriptomic. Front Neurol. 2024;15:1515981. https://doi.org/10.3389/fneur.2024.1515981
73. Mathys H, Davila-Velderrain J, Peng Z, Gao F, Mohammadi S, Young J Z, et al. Single-cell transcriptomic analysis of Alzheimer's disease. Nature. 2019;570:332-337. https://doi.org/10.1038/s41586-019-1195-2
74. Grubman A, Chew G, Ouyang J F, Sun G, Choo X Y, McLean C, et al. A single-cell atlas of entorhinal cortex from individuals with Alzheimer's disease reveals cell-type-specific gene expression regulation. Nat Neurosci. 2019;22:2087-2097. https://doi.org/10.1038/s41593-019-0539-4
75. Chen W T, Lu A, Craessaerts K, Pavie B, Sala Frigerio C, Corthout N, et al. Spatial Transcriptomics and In Situ Sequencing to Study Alzheimer's Disease. Cell. 2020;182:976-991.e19. https://doi.org/10.1016/j.cell.2020.06.038
76. Chen S, Chang Y, Li L, Acosta D, Li Y, Guo Q, et al. Spatially resolved transcriptomics reveals genes associated with the vulnerability of middle temporal gyrus in Alzheimer's disease. Acta Neuropathol Commun. 2022;10:188. https://doi.org/10.1186/s40478-022-01494-6
77. Zou D, Huang X, Lan Y, Pan M, Xie J, Huang Q, et al. Single-cell and spatial transcriptomics reveals that PTPRG activates the m(6)A methyltransferase VIRMA to block mitophagy-mediated neuronal death in Alzheimer's disease. Pharmacol Res. 2024;201:107098. https://doi.org/10.1016/j.phrs.2024.107098
78. Green G S, Fujita M, Yang H S, Taga M, Cain A, McCabe C, et al. Cellular communities reveal trajectories of brain ageing and Alzheimer's disease. Nature. 2024;633:634-645. https://doi.org/10.1038/s41586-024-07871-6
79. Kosoy R, Fullard J F, Zeng B, Bendl J, Dong P, Rahman S, et al. Genetics of the human microglia regulome refines Alzheimer's disease risk loci. Nat Genet. 2022;54:1145-1154. https://doi.org/10.1038/s41588-022-01149-1

Multi-omics technologies integration

80. Xiong X, James B T, Boix C A, Park Y P, Galani K, Victor M B, et al. Epigenomic dissection of Alzheimer's disease pinpoints causal variants and reveals epigenome erosion. Cell. 2023;186:4422-4437.e21. https://doi.org/10.1016/j.cell.2023.08.040
81. Gabitto M I, Travaglini K J, Rachleff V M, Kaplan E S, Long B, Ariza J, et al. Integrated multimodal cell atlas of Alzheimer's disease. Nat Neurosci. 2024;27:2366-2383. https://doi.org/10.1038/s41593-024-01774-5
82. Almeida M C, Eger S J, He C, Audouard M, Nikitina A, Glasauer S M K, et al. Single-nucleus RNA sequencing demonstrates an autosomal dominant Alzheimer's disease profile and possible mechanisms of disease protection. Neuron. 2024;112:1778-1794.e7. https://doi.org/10.1016/j.neuron.2024.02.009
83. Zupanic A, Bernstein H C, and Heiland I. Systems biology: current status and challenges. Cell Mol Life Sci. 2020;77:379-380. https://doi.org/10.1007/s00018-019-03410-z
84. Johnson E C B, Carter E K, Dammer E B, Duong D M, Gerasimov E S, Liu Y, et al. Large-scale deep multi-layer analysis of Alzheimer's disease brain reveals strong proteomic disease-related changes not observed at the RNA level. Nat Neurosci. 2022;25:213-225. https://doi.org/10.1038/s41593-021-00999-y
85. Madrid L, Moreno-Grau S, Ahmad S G-P A, de Rojas I X R, Martino Adami PV, García-González P, et al. Multiomics integrative analysis identifies APOE allele‐specific blood biomarkers associated to Alzheimers disease etiopathogenesis. Aging. 2021;13:9277 – 9329. https://doi.org/10.18632/aging.202950
86. Horgusluoglu E, Neff R, Song W M, Wang M, Wang Q, Arnold M, et al. Integrative metabolomics‐genomics approach reveals key metabolic pathways and regulators of Alzheimer's disease. AlzheimersDement. 2021;18:1260-1278. https://doi.org/10.1002/alz.12468
87. San Segundo-Acosta P, Montero-Calle A, Jernbom-Falk A, Alonso-Navarro M, Pin E, Andersson E, et al. Multiomics profiling of Alzheimer's disease serum for the identification of autoantibody biomarkers. J Proteome Res. 2021;20:5115-5130. https://doi.org/10.1021/acs.jproteome.1c00630
88. Wang H, Robinson J L, Kocabas P, Gustafsson J, Anton M, Cholley P-E, et al. Genome-scale metabolic network reconstruction of model animals as a platform for translational research. P Natl A Sci. 2021;118:e2102344118. https://doi.org/10.1073/pnas.2102344118
89. Zhang J, Sun X, Jia X, Sun B, Xu S, Zhang W, et al. Integrative multi‐omics analysis reveals the critical role of the PBXIP1 gene in Alzheimer's disease. Aging Cell. 2023;23:e14044. https://doi.org/10.1111/acel.14044
90. Marzi S J, Leung S K, Ribarska T, Hannon E, Smith A R, Pishva E, et al. A histone acetylome-wide association study of Alzheimer's disease identifies disease-associated H3K27ac differences in the entorhinal cortex. Nat Neurosci. 2018;21:1618-1627. https://doi.org/10.1038/s41593-018-0253-7
91. Nativio R, Lan Y, Donahue G, Sidoli S, Berson A, Srinivasan A R, et al. An integrated multi-omics approach identifies epigenetic alterations associated with Alzheimer's disease. Nat Genet. 2020;52:1024-1035. https://doi.org/10.1038/s41588-020-0696-0
92. Clark C, Dayon L, Masoodi M, Bowman G L, and Popp J. An integrative multi-omics approach reveals new central nervous system pathway alterations in Alzheimer's disease. Alzheimer's Res Ther. 2021;13:71. https://doi.org/10.1186/s13195-021-00814-7
93. Strefeler A, Jan M, Quadroni M, Teav T, Rosenberg N, Chatton J-Y, et al. Molecular insights into sex-specific metabolic alterations in Alzheimer's mouse brain using multi-omics approach. Alzheimer's Res Ther. 2023;15:8. https://doi.org/10.1186/s13195-023-01162-4

94. Wang C, Lu J, Sha X, Qiu Y, Chen H, and Yu Z. TRPV1 regulates ApoE4-disrupted intracellular lipid homeostasis and decreases synaptic phagocytosis by microglia. Experimental & Molecular Medicine. 2023;55:347-363. https://doi.org/10.1038/s12276-023-00935-z
95. Wang M, Li A, Sekiya M, Beckmann N D, Quan X, Schrode N, et al. Transformative network modeling of multi-omics data reveals detailed circuits, key regulators, and potential therapeutics for Alzheimer ' s disease. Neuron. **2021**;109:257-272.e14. https://doi.org/10.1016/j.neuron.2020.11.002
96. Zhou Y, Fang J, Bekris L M, Kim Y H, Pieper A A, Leverenz J B, et al. AlzGPS: a genome-wide positioning systems platform to catalyze multi-omics for Alzheimer's drug discovery. Alzheimer's Res Ther. 2021;13:24. https://doi.org/10.1186/s13195-020-00760-w
97. Alexander G E. Biology of Parkinson's disease: Pathogenesis and pathophysiology of a multisystem neurodegenerative disorder. Dialogues Clin Neurosci. 2004;6(3):259 – 280. https://doi.org/10.31887/DCNS.2004.6.3/galexander
98. A. Berardelli, J. C. Rothwell, P. D. Thompson, and M. Hallett. Pathophysiology of bradykinesia in Parkinson's disease. Brain. 2001;124:2131–2146. https://doi.org/10.1093/brain/124.11.2131
99. William Dauer a S P. Parkinson's disease mechanisms and models. Neuron. 2003;39:889–909. https://doi.org/10.1016/s0896-6273(03)00568-3
100. Wright Willis A, Evanoff B A, Lian M, Criswell S R, and Racette B A. Geographic and ethnic variation in Parkinson disease: A population-based study of US medicare beneficiaries. Neuroepidemiology. 2010;34:143-151. https://doi.org/10.1159/000275491
101. Kempuraj D, Thangavel R, Natteru PA, Selvakumar GP, Saeed D, Zahoor H, et al. Neuroinflammation induces neurodegeneration. J Neurol Neurosurg Spine. 2016;1:1003.
102. Gao T, Zheng R, Ruan Y, Fang Y, Jin C, Cao J, et al. Association of ZNF184, IL1R2, LRRK2, ITPKB, and PARK16 with sporadic Parkinson's disease in Eastern China. Neurosci Lett. 2020;735:135261. https://doi.org/10.1016/j.neulet.2020.135261
103. Witoelar A, Jansen I E, Wang Y, Desikan R S, Gibbs J R, Blauwendraat C, et al. Genome-wide pleiotropy between Parkinson disease and autoimmune diseases. JAMA Neurol. 2017;74:780–792. https://doi.org/10.1001/jamaneurol.2017.0469
104. Zhang W, Shen J, Wang Y, Cai K, Zhang Q, and Cao M. Blood SSR1: A possible biomarker for early prediction of Parkinson ' s disease. Front Mol Neurosci. 2022;15:762544. https://doi.org/10.3389/fnmol.2022.762544
105. Karayel O, Virreira Winter S, Padmanabhan S, Kuras Y I, Vu D T, Tuncali I, et al. Proteome profiling of cerebrospinal fluid reveals biomarker candidates for Parkinson's disease. Cell Rep Med. 2022;3:100661. https://doi.org/10.1016/j.xcrm.2022.100661
106. Sinclair E, Trivedi D K, Sarkar D, Walton-Doyle C, Milne J, Kunath T, et al. Metabolomics of sebum reveals lipid dysregulation in Parkinson ' s disease. Nat Commun. 2021;12:1592. https://doi.org/10.1038/s41467-021-21669-4
107. Tan A H, Chong C W, Lim S-Y, Yap I K S, Teh C S J, Loke M F, et al. Gut microbial ecosystem in Parkinson disease: new clinicobiological insights from multi-omics. Ann Neurol. 2020;89:546–559. https://doi.org/10.1002/ana.25982
108. Pereira P A B, Trivedi D K, Silverman J, Duru I C, Paulin L, Auvinen P, et al. Multiomics implicate gut microbiota in altered lipid and energy metabolism in Parkinson's disease. npj Parkinson's Dis. 2022;8:39. https://doi.org/10.1038/s41531-022-00300-3
109. Luan H, Liu L-F, Meng N, Tang Z, Chua K-K, Chen L-L, et al. LC–MS-Based urinary metabolite

signatures in idiopathic Parkinson ' s disease. J Proteome Res. 2014;14:467-478. https://doi.org/10.1021/pr500807t
110. Luan H, Liu L-F, Tang Z, Zhang M, Chua K-K, Song J-X, et al. Comprehensive urinary metabolomic profiling and identification of potential noninvasive marker for idiopathic Parkinson' s disease. Sci Rep-uk. 2015;5:13888. https://doi.org/10.1038/srep13888
111. Smajić S, Prada-Medina C A, Landoulsi Z, Ghelfi J, Delcambre S, Dietrich C, et al. Single-cell sequencing of human midbrain reveals glial activation and a Parkinson-specific neuronal state. Brain. 2022;145:964-978. https://doi.org/10.1093/brain/awab446
112. Mirzac D, Bange M, Kunz S, de Jager P L, Groppa S, and Gonzalez-Escamilla G. Targeting pathological brain activity-related to neuroinflammation through scRNA-seq for new personalized therapies in Parkinson's disease. Signal Transduct Target Ther. 2025;10:10. https://doi.org/10.1038/s41392-024-02086-7
113. Kamath T, Abdulraouf A, Burris S J, Langlieb J, Gazestani V, Nadaf N M, et al. Single-cell genomic profiling of human dopamine neurons identifies a population that selectively degenerates in Parkinson's disease. Nat Neurosci. 2022;25:588-595. https://doi.org/10.1038/s41593-022-01061-1
114. Zhu B, Park J M, Coffey S R, Russo A, Hsu I U, Wang J, et al. Single-cell transcriptomic and proteomic analysis of Parkinson's disease brains. Sci Transl Med. 2024;16:eabo1997. https://doi.org/10.1126/scitranslmed.abo1997
115. Gu X-J, Su W-M, Dou M, Jiang Z, Duan Q-Q, Yin K-F, et al. Expanding causal genes for Parkinson' s disease via multi-omics analysis. npj Parkinson's Dis. 2023;9:146. https://doi.org/10.1038/s41531-023-00591-0
116. Rutledge J, Lehallier B, Zarifkar P, Losada P M, Shahid-Besanti M, Western D, et al. Comprehensive proteomics of CSF, plasma, and urine identify DDC and other biomarkers of early Parkinson's disease. Acta Neuropathol. 2024;147:52. https://doi.org/10.1007/s00401-024-02706-0
117. Hu J, Li P, Han H, Ji P, Zhao X, and Li Z. Integrated analysis of metabolomic and transcriptomic profiling reveals the effect of Buyang Huanwu decoction on Parkinson's disease in mice. Phytomedicine. 2023;114:154755. https://doi.org/10.1016/j.phymed.2023.154755
118. Cheng Q, Wang J, Li M, Fang J, Ding H, Meng J, et al. CircSV2b participates in oxidative stress regulation through miR-5107-5p-Foxk1-Akt1 axis in Parkinson's disease. Redox Biol. 2022;56:102430. https://doi.org/10.1016/j.redox.2022.102430
119. Organization W H. Epilepsy: A Public Health Imperative. Epilepsy. 2019;1:171.
120. Kwon C A-O, Wagner R A-O, Carpio A A-O, Jetté N A-O, Newton C A-O, and Thurman D A-O. The worldwide epilepsy treatment gap: A systematic review and recommendations for revised definitions - A report from the ILAE Epidemiology Commission. Epilepsia. 63:551 – 564. https://doi.org/10.1111/epi.17112
121. Scheffer I E, Berkovic S, Capovilla G, Connolly M B, French J, Guilhoto L, et al. ILAE classification of the epilepsies: Position paper of the ILAE commission for classification and terminology. Epilepsia. 2017;58:512-521. https://doi.org/10.1111/epi.13709
122. Okamoto O K, Janjoppi L, Bonone F M, Pansani A P, da Silva A V, Scorza F A, et al. Whole transcriptome analysis of the hippocampus: toward a molecular portrait of epileptogenesis. BMC Genomics. 2010;11:230. https://doi.org/10.1186/1471-2164-11-230
123. Chen Q L, Xia L, Zhong S P, Wang Q, Ding J, and Wang X. Bioinformatic analysis identifies key transcriptome signatures in temporal lobe epilepsy. CNS Neurosci Ther. 2020;26:1266-1277. https://doi.org/10.1111/cns.13470

Multi-omics technologies integration

124. Lin Q, Li W, Zhang Y, Li Y, Liu P, Huang X, et al. Brain Morphometric Alterations in Focal to Bilateral Tonic-Clonic Seizures in Epilepsy Associated With Excitatory/Inhibitory Imbalance. CNS Neurosci Ther. 2024;30:e70129. https://doi.org/10.1111/cns.70129
125. Fan C, Gao Y, Liang G, Huang L, Wang J, Yang X, et al. Transcriptomics of Gabra4 knockout mice reveals common NMDAR pathways underlying autism, memory, and epilepsy. Mol Autism. 2020;11:13. https://doi.org/10.1186/s13229-020-0318-9
126. Li R, Xiao L, Han H, Long H, Liao W, Yang Z, et al. Transcriptionally downregulated GABAergic genes associated with synaptic density network dysfunction in temporal lobe epilepsy. Eur J Nucl Med Mol I. 2025;52:1970-1988. https://doi.org/10.1007/s00259-024-07054-5
127. Biagini G, Keren-Aviram G, Dachet F, Bagla S, Balan K, Loeb J A, et al. Proteomic analysis of human epileptic neocortex predicts vascular and glial changes in epileptic regions. Plos One. 2018;13:e0195639. https://doi.org/10.1371/journal.pone.0195639
128. Sadeghi L, Rizvanov A A, Dabirmanesh B, Salafutdinov I I, Sayyah M, Shojaei A, et al. Proteomic profiling of the rat hippocampus from the kindling and pilocarpine models of epilepsy: potential targets in calcium regulatory network. Sci Rep-uk. 2021;11:8252. https://doi.org/10.1038/s41598-021-87555-7
129. Han Q-T, Yang W-Q, Zang C, Zhou L, Zhang C-J, Bao X, et al. The toxic natural product tutin causes epileptic seizures in mice by activating calcineurin. Signal Transduction Targeted Ther. 2023;8:101. https://doi.org/10.1038/s41392-023-01312-y
130. Boguszewicz Ł, Jamroz E, Ciszek M, Emich-Widera E, Kijonka M, Banasik T, et al. NMR-based metabolomics in pediatric drug resistant epilepsy – preliminary results. Sci Rep-uk. 2019;9:15035. https://doi.org/10.1038/s41598-019-51337-z
131. Hamelin S, Stupar V, Mazière L, Guo J, Labriji W, Liu C, et al. In vivo γ-aminobutyric acid increase as a biomarker of the epileptogenic zone: An unbiased metabolomics approach. Epilepsia. 2020;62:163-175. https://doi.org/10.1111/epi.16768
132. Pfisterer U, Petukhov V, Demharter S, Meichsner J, Thompson J J, Batiuk M Y, et al. Identification of epilepsy-associated neuronal subtypes and gene expression underlying epileptogenesis. Nat Commun. 2020;11:5038. https://doi.org/10.1038/s41467-020-18752-7
133. Wen F, Tan Z, Huang D, and Xiang J. Molecular mechanism analyses of post-traumatic epilepsy and hereditary epilepsy based on 10× single-cell transcriptome sequencing technology. CNS Neurosci Ther. 2024;30:e14702. https://doi.org/10.1111/cns.14702
134. Liu Q, Shen C, Dai Y, Tang T, Hou C, Yang H, et al. Single-cell, single-nucleus and xenium-based spatial transcriptomics analyses reveal inflammatory activation and altered cell interactions in the hippocampus in mice with temporal lobe epilepsy. Biomark Res. 2024;12:103. https://doi.org/10.1186/s40364-024-00636-3
135. Johnson M R, Behmoaras J, Bottolo L, Krishnan M L, Pernhorst K, Santoscoy P L M, et al. Systems genetics identifies Sestrin 3 as a regulator of a proconvulsant gene network in human epileptic hippocampus. Nat Commun. 2015;6:6031. https://doi.org/10.1038/ncomms7031
136. Sharma S, Sharma M, Rana A K, Joshi R, Swarnkar M K, Acharya V, et al. Deciphering key regulators involved in epilepsy-induced cardiac damage through whole transcriptome and proteome analysis in a rat model. Epilepsia. 2020;62:504-516. https://doi.org/10.1111/epi.16794
137. Harutyunyan A, Chong D, Li R, Shah A D, Ali Z, Huang C, et al. An integrated multi-omic network analysis identifies seizure-associated dysregulated pathways in the GAERS model of absence epilepsy. Int J Mol Sci. 2022;23:6063. https://doi.org/10.3390/ijms23116063

Multi-omics technologies integration

138. Wu H C, Dachet F, Ghoddoussi F, Bagla S, Fuerst D, Stanley J A, et al. Altered metabolomic–genomic signature: A potential noninvasive biomarker of epilepsy. Epilepsia. 2017;58:1626-1636. https://doi.org/10.1111/epi.13848
139. Venø M T, Reschke C R, Morris G, Connolly N M C, Su J, Yan Y, et al. A systems approach delivers a functional microRNA catalog and expanded targets for seizure suppression in temporal lobe epilepsy. P Natl A Sci. 2020;117:15977-15988. https://doi.org/10.1073/pnas.1919313117
140. Tian D-C, Zhang C, Yuan M, Yang X, Gu H, Li Z, et al. Incidence of multiple sclerosis in China: A nationwide hospital-based study. The Lancet Regional Health Western Pacific. 2020;1:100010. https://doi.org/10.1016/j.lanwpc.2020.100010
141. Zhou Q, Zhang T, Meng H, Shen D, Li Y, He L, et al. Characteristics of cerebral blood flow in an Eastern sample of multiple sclerosis patients: A potential quantitative imaging marker associated with disease severity. Front Immunol. 2022;13:1025908. https://doi.org/10.3389/fimmu.2022.1025908
142. Weinshenker BG B B, Rice GP, Noseworthy J, Carriere W, Baskerville J, Ebers GC. The natural history of multiple sclerosis: a geographically based study. 2. Predictive value of the early clinical course. Brain. 1989;112:1419–1428. https://doi.org/10.1093/brain/112.6.1419
143. Waubant E, Lucas R, Mowry E, Graves J, Olsson T, Alfredsson L, et al. Environmental and genetic risk factors for MS: an integrated review. Ann Clin Transl Neurol. 2019;6:1905-1922. https://doi.org/10.1002/acn3.50862
144. Galea I, Ward-Abel N, and Heesen C. Relapse in multiple sclerosis. BMJ: British Medical Journal. 2015;350:h1765. https://doi.org/10.1136/bmj.h1765
145. Arneth B, and Kraus J. Laboratory biomarkers of multiple sclerosis (MS). Clin Biochem. 2022;99:1-8. https://doi.org/10.1016/j.clinbiochem.2021.10.004
146. De Jager P, Zeng L, Khan A, Lama T, Chitnis T, Weiner H, et al. GWAS highlights the neuronal contribution to multiple sclerosis susceptibility. medRxiv. 2025. https://doi.org/10.21203/rs.3.rs-5644532/v1
147. Åkesson J, Hojjati S, Hellberg S, Raffetseder J, Khademi M, Rynkowski R, et al. Proteomics reveal biomarkers for diagnosis, disease activity and long-term disability outcomes in multiple sclerosis. Nat Commun. 2023;14(1):6903. https://doi.org/10.1038/s41467-023-42682-9
148. Peng H-r, Qiu J-Q, Zhou Q-m, Zhang Y-k, Chen Q-y, Yin Y-q, et al. Intestinal epithelial dopamine receptor signaling drives sex-specific disease exacerbation in a mouse model of multiple sclerosis. Immun. 2023;56:2773-2789. https://doi.org/10.1016/j.immuni.2023.10.016
149. Insha Zahoora H S, 1, Indrani Dattab, Mohammad Ejaz Ahmeda, Laila M. Poissonb, Jeffrey Watersa, Faraz Rashida, Rui Bina, Jaspreet Singha,Mirela Cerghet， Ashok Kumarc, Md Nasrul Hodaa, Ramandeep Rattand, Ashutosh K. Mangalamand Shailendra Giri. Blood-based untargeted metabolomics in relapsing-remitting multiple sclerosis revealed the testable therapeutic target. P Natl A Sci. 119:e2123265119. https://doi.org/10.1073/pnas
150. Schafflick D, Xu C A, Hartlehnert M, Cole M, Schulte-Mecklenbeck A, Lautwein T, et al. Integrated single cell analysis of blood and cerebrospinal fluid leukocytes in multiple sclerosis. Nat Commun. 2020;11:247. https://doi.org/10.1038/s41467-019-14118-w
151. Kukanja P, Langseth C M, Rubio Rodríguez-Kirby L A, Agirre E, Zheng C, Raman A, et al. Cellular architecture of evolving neuroinflammatory lesions and multiple sclerosis pathology. Cell. 2024;187:1990-2009.e19. https://doi.org/https://doi.org/10.1016/j.cell.2024.02.030
152. Elkjaer M L, Hartebrodt A, Oubounyt M, Weber A, Vitved L, Reynolds R, et al. Single-Cell Multi-

Omics Map of Cell Type-Specific Mechanistic Drivers of Multiple Sclerosis Lesions. Neurology(R) neuroimmunology & neuroinflammation. 2024;11:e200213. https://doi.org/10.1212/nxi.0000000000200213
153. Li Z, Rius Rigau A, Xie W, Huang L, Ye W, Li Y N, et al. Spatial multiomics decipher fibroblast-macrophage dynamics in systemic sclerosis. Ann Rheum Dis. 2025;84:1231-1245. https://doi.org/10.1016/j.ard.2025.04.025
154. Kaufmann M, Schaupp A-L, Sun R, Coscia F, Dendrou C A, Cortes A, et al. Identification of early neurodegenerative pathways in progressive multiple sclerosis. Nat Neurosci. 2022;25:944-955. https://doi.org/10.1038/s41593-022-01097-3
155. Wu T, Ning S, Zhang H, Cao Y, Li X, Hao J, et al. Role of ferroptosis in neuroimmunity and neurodegeneration in multiple sclerosis revealed by multi - omics data. J Cell Mol Med. 2024;28:e18396. https://doi.org/10.1111/jcmm.18396
156. Yang F, Zhao L-Y, Yang W-Q, Chao S, Ling Z-X, Sun B-Y, et al. Quantitative proteomics and multi-omics analysis identifies potential biomarkers and the underlying pathological molecular networks in Chinese patients with multiple sclerosis. BMC Neurol. 2024;24:423. https://doi.org/10.1186/s12883-024-03926-3
157. Zhou Q, Xie Z, He L, Sun G, Meng H, Luo Z, et al. Multi-omics profiling reveals peripheral blood biomarkers of multiple sclerosis: implications for diagnosis and stratification. Front Pharmacol. 2024;15:1458046. https://doi.org/10.3389/fphar.2024.1458046
158. Benjamin E J, Muntner P, Alonso A, Bittencourt M S, Callaway C W, Carson A P, et al. Heart disease and stroke statistics—2019 update: A report from the american heart association. Circulation. 2019;139:e56–e528. https://doi.org/10.1161/cir.0000000000000659
159. Tu W-J, Zhao Z, Yin P, Cao L, Zeng J, Chen H, et al. Estimated burden of stroke in china in 2020. JAMA Netw Open. 2023;6:e231455. https://doi.org/10.1001/jamanetworkopen.2023.1455
160. Eloy Cuadrado, Anna Rosell, Nuria Colomé, Mar Hernández-Guillamon, Teresa García-Berrocoso, Marc Ribo, et al. The proteome of human brain after ischemic stroke. J Neuropath Exp Neur. 2010;69:1105–1115. https://doi.org/10.1097/NEN.0b013e3181f8c539
161. Simats A, García-Berrocoso T, Ramiro L, Giralt D, Gill N, Penalba A, et al. Characterization of the rat cerebrospinal fluid proteome following acute cerebral ischemia using an aptamer-based proteomic technology. Sci Rep-uk. 2018;8:7899. https://doi.org/10.1038/s41598-018-26237-3
162. García-Berrocoso T, Llombart V, Colàs-Campàs L, Hainard A, Licker V, Penalba A, et al. Single cell immuno-laser microdissection coupled to label-free proteomics to reveal the proteotypes of human brain cells after ischemia. Mol Cell Proteomics. 2018;17:175-189. https://doi.org/10.1074/mcp.RA117.000419
163. Dykstra-Aiello C, Jickling G C, Ander B P, Shroff N, Zhan X, Liu D, et al. Altered expression of long noncoding RNAs in blood after ischemic stroke and proximity to putative stroke risk loci. Stroke. 2016;47:2896-2903. https://doi.org/10.1161/strokeaha.116.013869
164. Deng Q-W, Li S, Wang H, Sun H-L, Zuo L, Gu Z-T, et al. Differential long noncoding RNA expressions in peripheral blood mononuclear cells for detection of acute ischemic stroke. Clin Sci. 2018;132:1597-1614. https://doi.org/10.1042/cs20180411
165. Wang W, Gao F, Zhao Z, Wang H, Zhang L, Zhang D, et al. Integrated analysis of LncRNA-mRNA co-expression profiles in patients with moyamoya disease. Sci Rep-uk. 2017;7:42421. https://doi.org/10.1038/srep42421
166. Holdt L M, and Teupser D. Long noncoding RNA ANRIL: Lnc-ing genetic variation at the

chromosome 9p21 locus to molecular mechanisms of atherosclerosis. Front Cardiovasc Med. 2018;5:145. https://doi.org/10.3389/fcvm.2018.00145

167. Wang J, Ruan J, Zhu M, Yang J, Du S, Xu P, et al. Predictive value of long noncoding RNA ZFAS1 in patients with ischemic stroke. Clin Exp Hypertens. **2018**;41:615-621. https://doi.org/10.1080/10641963.2018.1529774
168. Li P, Teng F, Gao F, Zhang M, Wu J, and Zhang C. Identification of circulating microRNAs as potential biomarkers for detecting acute ischemic stroke. Cell Mol Neurobiol. 2015;35:433-447. https://doi.org/10.1007/s10571-014-0139-5
169. Androvic P, Kirdajova D, Tureckova J, Zucha D, Rohlova E, Abaffy P, et al. Decoding the Transcriptional Response to Ischemic Stroke in Young and Aged Mouse Brain. Cell Rep. 2020;31:107777. https://doi.org/10.1016/j.celrep.2020.107777
170. Kimberly W T, Wang Y, Pham L, Furie K L, and Gerszten R E. Metabolite profiling identifies a branched chain amino acid signature in acute cardioembolic stroke. Stroke. 2013;44:1389-1395. https://doi.org/10.1161/strokeaha.111.000397
171. Choi JY, Kim JS, Kim JH, Oh K, Koh SB, and WK. S. High free fatty acid level is associated with recurrent stroke in cardioembolic stroke patients. Neurology. 2014;82:1142 – 1148. https://doi.org/10.1212/WNL.0000000000000264
172. Jin C, Shi Y, Shi L, Leak R K, Zhang W, Chen K, et al. Leveraging single-cell RNA sequencing to unravel the impact of aging on stroke recovery mechanisms in mice. Proc Natl Acad Sci U S A. 2023;120:e2300012120. https://doi.org/10.1073/pnas.2300012120
173. Ma Y, Zheng K, Zhao C, Chen J, Chen L, Zhang Y, et al. Microglia LILRB4 upregulation reduces brain damage after acute ischemic stroke by limiting CD8(+) T cell recruitment. J Neuroinflammation. 2024;21:214. https://doi.org/10.1186/s12974-024-03206-4
174. Li S, Zeng G, Pang C, Li J, Wu L, Luo M, et al. Single-cell and spatial transcriptomics analysis reveals that Pros1(+) oligodendrocytes are involved in endogenous neuroprotection after brainstem stroke. Neurobiol Dis. 2025;208:106855. https://doi.org/10.1016/j.nbd.2025.106855
175. Gu L, Chen H, Sun M, Chen Y, Shi Q, Chang J, et al. Unraveling dynamic immunological landscapes in intracerebral hemorrhage: insights from single-cell and spatial transcriptomic profiling. MedComm. 2024;5:e635. https://doi.org/10.1002/mco2.635
176. Gu L, Chen H, Geng R, Sun M, Shi Q, Chen Y, et al. Single-cell and Spatial Transcriptomics Reveals Ferroptosis as The Most Enriched Programmed Cell Death Process in Hemorrhage Stroke-induced Oligodendrocyte-mediated White Matter Injury. Int J Biol Sci. 2024;20:3842-3862. https://doi.org/10.7150/ijbs.96262
177. Han B, Zhou S, Zhang Y, Chen S, Xi W, Liu C, et al. Integrating spatial and single-cell transcriptomics to characterize the molecular and cellular architecture of the ischemic mouse brain. Sci Transl Med. 2024;16:eadg1323. https://doi.org/10.1126/scitranslmed.adg1323
178. Wang X, Zhang A, Yu Q, Wang Z, Wang J, Xu P, et al. Single-Cell RNA Sequencing and Spatial Transcriptomics Reveal Pathogenesis of Meningeal Lymphatic Dysfunction after Experimental Subarachnoid Hemorrhage. Adv Sci (Weinh). 2023;10:e2301428. https://doi.org/10.1002/advs.202301428
179. Sun J, Singh P, Shami A, Kluza E, Pan M, Djordjevic D, et al. Spatial Transcriptional Mapping Reveals Site-Specific Pathways Underlying Human Atherosclerotic Plaque Rupture. J Am Coll Cardiol. 2023;81:2213-2227. https://doi.org/10.1016/j.jacc.2023.04.008
180. Scott E Y, Safarian N, Casasbuenas D L, Dryden M, Tockovska T, Ali S, et al. Integrating single-

cell and spatially resolved transcriptomic strategies to survey the astrocyte response to stroke in male mice. Nat Commun. 2024;15:1584. https://doi.org/10.1038/s41467-024-45821-y

181. Zhang C, Qin F, Li X, Du X, and Li T. Identification of novel proteins for lacunar stroke by integrating genome-wide association data and human brain proteomes. BMC Med. 2022;20:211. https://doi.org/10.1186/s12916-022-02408-y
182. Wang R, Liu M, Ren G, Luo G, Wang Z, Ge Z, et al. Zhilong Huoxue Tongyu capsules' effects on ischemic stroke: An assessment using fecal 16S rRNA gene sequencing and untargeted serum metabolomics. Front Pharmacol. 2022;13:1052110. https://doi.org/10.3389/fphar.2022.1052110
183. Yuan Y, Sheng P, Ma B, Xue B, Shen M, Zhang L, et al. Elucidation of the mechanism of Yiqi Tongluo Granule against cerebral ischemia/reperfusion injury based on a combined strategy of network pharmacology, multi-omics and molecular biology. Phytomedicine. 2023;118:154934. https://doi.org/10.1016/j.phymed.2023.154934
184. Hochstetler A, Raskin J, and Blazer-Yost B L. Hydrocephalus: historical analysis and considerations for treatment. Eur J Med Res. 2022;27:168. https://doi.org/10.1186/s40001-022-00798-6
185. Rekate H L. A contemporary Ddefinition and classification of hydrocephalus. Semin Pediatr Neurol. 2009;16:9-15. https://doi.org/10.1016/j.spen.2009.01.002
186. Reith W. Hydrozephalus. Der Radiologe. 2012;52:805-806. https://doi.org/10.1007/s00117-012-2329-9
187. Whitelaw A, Brion L P, Kennedy C R, and Odd D. Diuretic therapy for newborn infants with posthemorrhagic ventricular dilatation. Cochrane Db Syst Rev. 2001;2001:CD002270. https://doi.org/10.1002/14651858.Cd002270
188. Kahle K T, Kulkarni A V, Limbrick D D, and Warf B C. Hydrocephalus in children. The Lancet. 2016;387:788-799. https://doi.org/10.1016/s0140-6736(15)60694-8
189. Anwar F, Zhang K, Sun C, Pang M, Zhou W, Li H, et al. Hydrocephalus: An update on latest progress in pathophysiological and therapeutic research. Biomed Pharmacother. 2024;181:117702. https://doi.org/10.1016/j.biopha.2024.117702
190. Xiuyun Liu H Z, Marek Czosnyka , Chiara Robba , Zofia Czosnyka , Jennifer Lee Summers , Huijie Yu , Xiaoguang Tong , Guoyi Gao , Gelei Xiao , Kai Yu , Yan Xing , Renling Mao , Shaoya Yin , Yangong Chao , Hongliang Li , Ke Pu , Keke Feng , Meijun Pang , Dong Ming. . Advancing hydrocephalus management: pathogenesis insights, therapeutic innovations, and emerging challenges. Aging Dis. 2025:10.14336/AD.2024.1434. https://doi.org/10.14336/AD.2024.1434
191. Jin S C, Dong W, Kundishora A J, Panchagnula S, Moreno-De-Luca A, Furey C G, et al. Exome sequencing implicates genetic disruption of prenatal neuro-gliogenesis in sporadic congenital hydrocephalus. Nat Med. 2020;26:1754-1765. https://doi.org/10.1038/s41591-020-1090-2
192. Yuan L, Zou D, Yang X, Chen X, Lu Y, Zhang A, et al. Proteomics and functional study reveal kallikrein-6 enhances communicating hydrocephalus. Clin Proteomics. 2021;18:30. https://doi.org/10.1186/s12014-021-09335-9
193. Ying Y, Lin J, Gao W, Yue L, Zeng Q, Bartas K, et al. Proteomic profiling in cerebrospinal fluid reveal biomarkers for shunt outcome in idiopathic normal-pressure hydrocephalus. J Adv Res. 2025;S2090-12329250:00287-5. https://doi.org/https://doi.org/10.1016/j.jare.2025.04.043
194. Nagata Y, Bundo M, Sugiura S, Kamita M, Ono M, Hattori K, et al. PTPRQ as a potential biomarker for idiopathic normal pressure hydrocephalus. Mol Med Rep. 2017;16:3034-3040. https://doi.org/10.3892/mmr.2017.7015

Multi-omics technologies integration

195. Nagata Y, Hirayama A, Ikeda S, Shirahata A, Shoji F, Maruyama M, et al. Comparative analysis of cerebrospinal fluid metabolites in Alzheimer ' s disease and idiopathic normal pressure hydrocephalus in a Japanese cohort. Biomark Res. 2018;6:5. https://doi.org/10.1186/s40364-018-0119-x
196. Wang Z, Nie X, Gao F, Tang Y, Ma Y, Zhang Y, et al. Increasing brain N-acetylneuraminic acid alleviates hydrocephalus-induced neurological deficits. CNS Neurosci Ther. 2023;29:3183-3198. https://doi.org/10.1111/cns.14253
197. Li Y, Di C, Song S, Zhang Y, Lu Y, Liao J, et al. Choroid plexus mast cells drive tumor-associated hydrocephalus. Cell. 2023;186:5719-5738.e28. https://doi.org/https://doi.org/10.1016/j.cell.2023.11.001
198. Hale A T, Song Y, Davies C, Liu S, Gaskin R, Arynchyna-Smith A, et al. Integrative genomics elucidates the evolutionary, temporal, and developmental origins of a hydrocephalus risk gene. medRxiv. 2025. https://doi.org/10.1101/2025.09.01.25334358
199. Hale A T, Bastarache L, Morales D M, Wellons J C, Limbrick D D, and Gamazon E R. Multi-omic analysis elucidates the genetic basis of hydrocephalus. Cell Rep. 2021;35:109085. https://doi.org/10.1016/j.celrep.2021.109085
200. Chen J, Wang L, Peng X, Cheng T, Yang Y, Su J, et al. Identification of CSPG4 as a biomarker and therapeutic target for infantile post-hemorrhagic hydrocephalus via multi-omics analysis. Adv Sci. 2024;12:e2410056. https://doi.org/10.1002/advs.202410056
201. Tarazona S, Balzano-Nogueira L, Gómez-Cabrero D, Schmidt A, Imhof A, Hankemeier T, et al. Harmonization of quality metrics and power calculation in multi-omic studies. Nat Commun. 2020;11:3092. https://doi.org/10.1038/s41467-020-16937-8
202. Antonelli J, Claggett B L, Henglin M, Kim A, Ovsak G, Kim N, et al. Statistical workflow for feature selection in human metabolomics Data. Metabolites. 2019;9:143. https://doi.org/10.3390/metabo9070143
203. Liew A W C, Law N F, and Yan H. Missing value imputation for gene expression data: computational techniques to recover missing data from available information. Briefings Bioinf. 2010;12:498-513. https://doi.org/10.1093/bib/bbq080
204. Vivian J, Eizenga JM, Beale HC, Vaske OM, and B. P. Bayesian framework for detecting gene expression outliers in individual samples. JCO Clin Cancer Inform. 2020;4:160 – 170. https://doi.org/10.1200/CCI.19.00095
205. Liu L, Chen A, Li Y, Mulder J, Heyn H, and Xu X. Spatiotemporal omics for biology and medicine. Cell. 2024;187:4488-4519. https://doi.org/10.1016/j.cell.2024.07.040
206. Lim J, Park C, Kim M, Kim H, Kim J, and Lee D-S. Advances in single-cell omics and multiomics for high-resolution molecular profiling. Experimental & Molecular Medicine. 2024;56:515-526. https://doi.org/10.1038/s12276-024-01186-2
207. Park H J, Park B, and Lee S S. Radiomics and deep learning: hepatic applications. Korean J Radiol. 2020;21:387-401. https://doi.org/10.3348/kjr.2019.0752
208. Xu J L, Mao C S, Hou Y, Luo Y, Binder J L, Zhou Y D, et al. Interpretable deep learning translation of GWAS and multi-omics findings to identify pathobiology and drug repurposing in Alzheimer?s disease. Cell Rep. 2022;41:26. https://doi.org/10.1016/j.celrep.2022.111717
209. He X J, Liu X W, Zuo F L, Shi H B, and Jing J. Artificial intelligence-based multi-omics analysis fuels cancer precision medicine. Semin Cancer Biol. 2023;88:187-200. https://doi.org/10.1016/j.semcancer.2022.12.009

Multi-omics technologies integration

210. Michetti F, Clementi M E, Di Liddo R, Valeriani F, Ria F, Rende M, et al. The S100B protein: A multifaceted pathogenic factor more than a biomarker. Int J Mol Sci. 2023;24. https://doi.org/10.3390/ijms24119605

211. Vignoli A, Paciotti S, Tenori L, Eusebi P, Biscetti L, Chiasserini D, et al. Fingerprinting Alzheimer's disease by 1H nuclear magnetic resonance spectroscopy of cerebrospinal fluid. J Proteome Res. 2020;19:1696-1705. https://doi.org/10.1021/acs.jproteome.9b00850

Multi-omics technologies integration

Table 1: Overview of the Four Omics Technologies.

Technology	Precision	Price Range	Advantages	Disadvantages	Disease Application Representation
**Genomics**	High	High	· Conduct a comprehensive analysis of genetic sequences.<br>· Uncover the depth of genetic variations.<br>· Be suitable for gene discovery and genetic disease studies.	· High experimental costs.<br>· Complex techniques and large sample sizes.<br>· Considerable time in analysis.	· Genetic disorders.<br>· Cancer genomics.<br>· Genetic counseling.
**Transcriptomics**	Medium	Medium	· Reveal dynamic changes in gene expression.<br>· Differentiate gene regulatory networks.<br>· Assist in the classification of disease subtypes.	· High experimental and data analysis design.<br>· Limited real-time to reflect mRNA levels.	· Mental disorders.<br>· Cardiovascular diseases.<br>· Prognostication and efficacy assessment in cancer.
**Proteomics**	Low	Medium	· Reflect protein levels and modifications directly.<br>· Reveal protein-protein interaction networks.<br>· Explore changes in protein function.	· Complex data analysis with low standardization.<br>· Effects of PTMs.	· Development of tumor biomarkers.<br>· Mechanistic studies in autoimmune diseases.<br>· Pathology of neurodegenerative diseases.
**Metabolomics**	Medium	Medium	· Provide the overall metabolic profile of the organism.<br>· Reflect metabolic changes associated with disease.<br>· Assist in early diagnosis and monitoring.	· Sensitive sample handling and storage conditions.<br>· Challenges in the detection of metabolites.	· Monitoring of endocrine disorders.<br>· Prediction of cardiovascular disease risk.<br>· Metabolic testing in diabetes.

Multi-omics technologies integration

**Single-cell Omics**	High	High	· Resolve cellular heterogeneity (tumor subclones).<br>· Identify rare cell types (<0.1% population).<br>· Enable multi-omics integration (ATAC+RNA).	· Spatial information loss from tissue dissociation<br>· Significant technical noise (dropout rate >15%)<br>· Single-cell amplification bias	· Tumor evolutionary tree construction.<br>· T-cell receptor clonal tracking.<br>· Nervous diseases neuronal subtyping.
**Spatial Omics**	High	High	· Preserve in-situ spatial topology.<br>· Quantify cell-cell interactions (immune synapses).<br>· Directly correlate pathological morphology with molecular expression.	· Resolution inversely proportional to throughput (e.g., MERFISH: ~1,000 genes).<br>· Optical diffraction limitations (>200nm).<br>· High complexity in multidimensional data integration.	· Tumor immune exclusion zone mapping.<br>· Brain region-specific protein gradient atlases.<br>· Myocardial infarction spatial injury demarcation.

PTMs: post-translational modifications.

Table 2: Comparative Analysis of Five Mainstream Proteomics Techniques.

Technology	Introduction	Advantages	Disadvantages	Labeling Groups	Data Volume	Cost
**iTRAQ**	Employ chemical labels to identify proteins.	· Simultaneous analysis of up to 8 groups.<br>· Processing of multiple samples.<br>· Enhanced throughput.	· Expensive reagents.<br>· Complex experimental procedures.	4 or 8	Medium	High
**TMT**	Utilize chemical labeling to identify proteins.	· Simultaneous analysis of up to 10 or 11 groups.<br>· Simultaneous processing of more samples.<br>· Higher sensitivity.	· Expensive reagents.<br>· Complex experimental operations.	10 or 11	Medium	High
**SILAC**	Introduce isotope-labeled amino acids into the culture media.	· Accurate quantification.<br>· High sensitivity.	· Requirement for cell culture.<br>· Unsuitability for clinical samples.	2 or 3	Low	Medium
**Label-free**	Detect endogenous peptides without labeling.	· No need for labeling.<br>· Simple sample preparation.<br>· Cost-effective.	· Reduced reproducibility.<br>· Slightly diminished sensitivity.	Unlimited	High	Low
**DIA/SWATH**	Obtain mass spectrometry data through a full scan.	· No labeling required.<br>· Simultaneous quantification of numerous proteins.<br>· Good reproducibility.	· Complex data analysis.<br>· Need for specialized software.	Unlimited	High	Medium

iTRAQ: isobaric tags for relative and absolute quantification; TMT: tandem mass tag technology; SILAC: stable-isotope labeling by amino acids in cell culture; DIA: data-independent acquisition; SWATH: sequential window acquisition of all theoretical mass spectral approach.

Multi-omics technologies integration

Supplementary Table S1: Application of Multi-omics and High-spatial-resolution Omics Technologies in AD.

Disease	Omics Type	Purpose	Biomarker	Relevance to AD Pathogenesis	Experiment Validation	Reference
**AD**	Genomics	Diagnosis	*APOE4*	· *APOE4* accelerates vascular dysfunction, BBB rupture, and neuronal degeneration.<br>· *APOE4* is pivotal in AD's vascular and neurodegenerative pathogenesis and serves as a marker.	N	Montagne A et al.[61]
**AD**	Genomics	Diagnosis	*H4K16ac*	· Compared to non-AD elderly participants, 25,000 peaks showed *H4K16ac* loss, while 9,000 showed increased *H4K16ac* in AD individuals.<br>· *H4K16ac* decreases with aging or AD-related gene sites.<br>· *H4K16ac* set the stage for an epigenetic link between aging and AD.<br>· *H4K16ac* can be a diagnostic marker for AD disease.	N	Nativio R et al.[62]
**AD**	Genomics	Diagnosis	*H3K9ac*, TAU protein	· TAU, whereas non-amyloid β pathology has a broad impact on histone acetylation in AD brain.<br>· *H3K9ac* structural domain shows similar gain or loss of TAU-related histone acetylation.<br>· Complex interactions between TAU and chromatin structure.<br>· *H3K9ac* and TAU are biomarkers for AD.	Y (Three Independent Datasets)	Klein HU et al.[63]
**AD**	Transcriptomics	Diagnosis	*INPPL1, PLXNB1*	· The M109 module is the one most directly associated with cognitive decline and amyloid load.<br>· *INPPL1* and *PLXNB1* are associated with extracellular β-amyloid levels in astrocyte cultures.<br>· *INPPL1* and *PLXNB1* are interesting candidates for AD.	Y (Two Independent Datasets)	Mostafavi S et al.[64]

Multi-omics technologies integration

**AD**	Transcriptomics	Diagnosis	Myelination	· Neuroinflammation pathways are significantly upregulated in AD.<br>· Genes for myelination and lipid metabolism are downregulated in AD.<br>· Novel treatments for AD can focus on promoting myelin repair.	Y (Three Independent Datasets)	Shouneng Peng et al.[65]
**AD**	Proteomics	Treatment	STAT3, YES1 and FYN	· STAT3, YES1, and FYN reduce neuroinflammation, TAU phosphorylation, and endogenous production of amyloid-42.<br>· Drugs targeting the cytokine transducer STAT3 and the Src family tyrosine kinases, YES1 and FYN, rescued molecular phenotypes relevant to AD pathogenesis.<br>· STAT3, YES1, and FYN can be used as drug targets for the treatment.	Y(Three Independent Datasets)	Jackson A et al.[66]
**AD**	Proteomics	Treatment	Insulin signaling and mitochondrial electron transport chain	· Changes in hippocampal protein expression profiles in APP/PS1 and E4 knockout mice.<br>· Different expression proteins in both mouse models, participate in insulin signaling and the mitochondrial electron transport chain.<br>· Preserving mitochondrial function and boosting insulin signaling could aid in improving cognitive function for AD patients.	N	He K et al.[67]
**AD**	Proteomics	Diagnosis /Treatment	The phosphorylation levels of GSK3β and Ppp3ca, GSK3β, Ppp3ca	· The phosphorylation levels of GSK3β and Ppp3ca are closely associated with mitochondrial biogenesis.<br>· Low-dose oral copper treatment changes the phosphorylation of key hippocampal proteins involved in mitochondrial, synaptic and axonal integrity.<br>· The phosphorylation levels of GSK3β and Ppp3ca are potential diagnostic and therapeutic targets.	N	Chen C et al.[68]

Multi-omics technologies integration

**AD** (autosomal dominant)	Proteomics	Diagnosis	GFAP, NPTX2, PEA15, SMOC1, SMOC2, TNFRSF1B	· Six-protein prediction model (GFAP, NPTX2, PEA15, SMOC1, SMOC2, TNFRSF1B) with excellent predictive performance (AUC>0.9)<br>· Six early biomarkers far exceed the warning time window of traditional markers	Y (Three Independent Datasets)	Shen Y et al.[69]
**AD**	Metabolomics	Diagnosis	Sphingolipids	· Identified sphingolipids map to AD-related pathways (TAU phosphorylation, amyloid metabolism, calcium homeostasis, acetylcholine biosynthesis, apoptosis AD is associated with dysregulation of transmethylation and polyamine pathways.<br>· Sphingolipids as early AD biomarkers.	Y (Independent Cohort)	Varma VR et al.[70]
**AD**	Metabolomics	Diagnosis	PKM2	· PKM is an important glycolytic enzyme associated with AD pathology.<br>· Induced neurons iNs from AD patients express cancer-associated PKM2.<br>· PKM2 promotes Warburg effect-like glycolytic reprogramming in old neurons.<br>· PKM2 specifically interacts with and enhances the transcription factors *STAT3* and *HIF1α*, promoting AD-induced neurons' neuronal fate loss.<br>· PKM2 is a potential diagnostic target for AD.	N	Traxler L et al.[71]
**AD**	ScRNA-seq	Diagnosis	Myelination-related gene (*LINGO1*)	· Myelination plays a pivotal role in the pathophysiology of AD.<br>· The myelination-related gene *LINGO1* is perturbed in neurons and glial cells in patients with AD.	Y (Two Independent Datasets	Mathys H et al.[73]

Multi-omics technologies integration

**AD**	SnRNA-seq	Diagnosis	*APOE*	· The transcription factor EB serves as a principal regulator of lysosomal function, modulating multiple disease-associated genes in specific astrocyte subpopulations in AD.<br>· The risk gene *APOE* exhibits upregulated expression in specific microglia and astrocytes in AD, correlating with the severity of TAU pathology.<br>· *APOE* as a risk gene for AD has been confirmed.	N	Grubman A et al.[74]
**AD**	Spatial transcriptomics	Diagnosis	*OLIG*	· Early plaque-proximal dysregulation of *OLIG*/myelination gene co-expression networks in AD.<br>· Multicellular 57-PIG networks emerge, enriched for complement activation, oxidative stress, lysosomal dysfunction, and neuroinflammatory pathways.<br>· *OLIG* can be used as a marker for future AD diagnosis to lay the foundation for AD diagnosis.	Y (Mouse Model)	Wei-Ting Chen et al.[75]
**AD**	Spatial transcriptomics	Diagnosis	*SPARC, CALB2, DIRAS2,* and *KRT17*	· 10× Visium + co-immunofluorescence of AD markers delineated gene expression architecture in human middle temporal gyrus.<br>· Cortex-specific layer-enriched DEGs, including novel candidates *SPARC, CALB2, DIRAS2,* and *KRT17*, exhibiting pronounced alterations.<br>· These genes demonstrate significant potential as diagnostic targets for AD.	Y (Independent cohort)	Shuo Chen et al.[76]

Multi-omics technologies integration

**AD**	ScRNA-seq, Spatial transcriptomics	Treatment	Inhibitor of *PTPRG* or *VIRMA*	· *PTPRG+* microglia subpopulation induces neuronal *VIRMA* via intercellular signaling.<br>· Neuronal *PTPRG* binding to *VIRMA* enhances RNA stability; upregulated *VIRMA* increases PRKN m6A, reduces its RNA stability, causing mitophagy-driven neuronal death and AD progression.<br>· *PTPRG/VIRMA* inhibitors show their impacts on mitochondrial function and neuronal survival, offering potential therapies for AD.	Y (Mouse Model)	Donghua Zou et al.[77]
**AD**	SnRNA-seq, Spatial transcriptomics	Treatment	Lipid	· Specific microglial and astrocytic subtypes linked to Aβ, TAU, and lipid pathways were identified, revealing new therapeutic targets for AD.<br>· Lipid-associated microglia drive Aβ-TAU interplay, while reactive astrocytes exacerbate TAU-mediated inflammation and cognitive decline.<br>· Altered glial communities and divergent aging trajectories directly promote AD through multicellular dysregulation, enabling early intervention strategies.	N	Green, G S et al.[78]
**AD**	Spatial transcriptomics, Epigenomes	Diagnosis	*SPI1/PU.1*	· *PU.1* contributes to inter-individual differences in microglial regulatory networks.<br>· Reduced *PU.1* binding disrupts chromatin stability, supporting its functional role in AD.<br>· *SPI1/PU.1* identified as key regulator of microglial gene expression and AD risk.	N	Kosoy, R et al.[79]

Multi-omics technologies integration

**AD**	Spatial transcriptomics, Epigenomes	Diagnosis	*SPI1, ELF2, RUNX1*	· AD risk loci are highly enriched in microglial enhancers and TF-binding sites (*SPI1, ELF2, RUNX1*).<br>· Glial cells show accessible regulatory changes in late AD, while neurons exhibit alterations in early stages.<br>· Suggests epigenomic erosion as a hallmark of late AD and a potential indicator of disease progression.	N	Xiong, X et al.[80]
**AD**	Spatial transcriptomics, Epigenomes	Diagnosis	SST^+ level, $Pvalb^+/Vip^+$ level	· An early stage characterized by slow pathological accumulation, inflammatory microglia, reactive astrocytes, loss of SST^+ inhibitory neurons, and remyelination by oligodendrocyte precursor cells.<br>· A late stage marked by exponential pathological increase and loss of both excitatory neurons and inhibitory ($Pvalb^+/Vip^+$) neuronal subtypes.	N	Gabitto, M I et al.[81]
**AD** (autosomal dominant)	SnRNA-seq, Transcriptomics	Diagnosis	*LRP1, FKBP1B, PSEN1*	· In autosomal dominant AD, autophagy and chaperone genes show marked upregulation, with spatially resolved transcriptomics confirming specific activation of chaperone-mediated autophagy.<br>· In autosomal dominant AD cases, astrocytic *LRP1* and *FKBP1B* upregulation alongside neuronal *PSEN1* downregulation may collectively represent an intrinsic protective mechanism.	Y (Independent Cohort)	Almeida, M C et al.[82]
**AD**	Proteomics, Transcriptomics	Diagnosis	*MAPK*/metabolic module, matrix body module	· AD-related modules include *MAPK* signaling/metabolism and matrixsome modules.<br>· Matrixsome module is affected by *APOE ε4* allele.<br>· *MAPK*/metabolism module links to cognitive decline rate.<br>· Disease modules are potential AD targets/biomarkers.	N	ECB et al.[84]

Multi-omics technologies integration

**AD**	Proteomics, Transcriptomics	Diagnosis	*FBP1, FBP2, RHOH, JPH2, ERAP2, SCLT1*, and *MBP*	· *FBP1, FBP2, RHOH, JPH2, ERAp2,* and *SCLT1* are upregulated in *APOE4* cases compared to average expression in the normal brain.<br>· *MBP* is one of the top candidate genes enhancing the relevance of myelination in AD.<br>· Biomarkers show consistent protein profiles in plasma and brain.	Y (Two Independent Datasets)	Madrid L et al.[85]
**AD**	Genomics, Transcriptomics, Proteomics, Metabolomics	Diagnosis	*ABCA1, CPT1A, Adiponectin* and *NGAL*	· Short-chain acylcarnitines/amino acids and medium/long-chain acylcarnitines are closely correlated with the severity of AD.<br>· Two genes (*ABCA1* and *CPT1A*) and two proteins (Adiponectin and *NGAL*) participate in the regulation of acylcarnitines and amino acids in AD.<br>· *ABCA1, CPT1A*, Adiponectin, and *NGAL* may be AD diagnostic biomarkers.	Y (Two Independent Datasets)	Horgusluoglu E et al.[86]
**AD**	Proteomics, Transcriptomics	Diagnosis	*IVD, CYFIP1* and *ADD2*	· Significantly higher *IVD* protein abundance in AD patients.<br>· *CYFIP1* and *ADD2* are significantly downregulated in AD patients.<br>· *IVD, CYF0IP1,* and *ADD2* combine to diagnose AD.	N	San Segundo et al.[87]
**AD**	Genomics, Metabolomics	Diagnosis	*CSTD, CTSB, CTSD,* and *GM2A*	· *CSTD* has been validated as a marker in previous CSF and plasma samples.<br>· AD progression is clearly accompanied by increased fold changes in these lysosomal proteins.<br>· Lysosomal proteins *CTSB, CTSD,* and *GM2A* are significantly increased as markers in CSF samples from AD patients.	N	Wang H et al.[88]

Multi-omics technologies integration

**AD**	Genomics, Proteomics	Diagnosis	*PBXIP1*	· *PBXIP1*-encoded protein shows significant association with all three AD neuropathological features.<br>· *PBXIP1* is associated with AD through its role in astrocytes and hippocampal neurons and the mTOR pathway.<br>· *PBXIP1* is associated with neuropathology and cognitive function.	Y (Two Independent Datasets)	Jingyun Zhang et al.[89]
**AD**	Genomics, Transcriptomics, Proteomics	Diagnosis	*H3K27ac*	· Differentially acetylated peaks are enriched in disease-related biological pathways, including those associated with Aβ and TAU pathology progression.<br>· Highly significant enrichment of AD risk variants in the *H3K27ac* peak region of the inner olfactory cortex, including *CR1, GPR22, KMO, PIM3, PSEN1,* and *RGCC*.<br>· *H3K27ac* can serve as a diagnostic target for AD.	N	Marzi SJ et al.[90]
**AD**	Transcriptomics, Proteomics, Epigenomics	Diagnosis	*H3K27ac, H3K9ac*	· RNA-seq analysis reveals upregulation of histone acetyltransferases in *H3K27ac* and *H3K9ac*.<br>· Genome-wide increases in *H3K27ac* and *H3K9ac* exacerbate Aβ42-driven neurodegeneration.<br>· Proteomic screening singles out *H3K27ac* and *H3K9ac* as major AD-specific enrichments.<br>· *H3K27ac* and *H3K9ac* affect disease pathways through dysregulated transcription and chromatin gene feedback loops.	Y (Two Independent Datasets, Drosophila Model)	Raffaella Nativio et al.[91]

Multi-omics technologies integration

**AD**	Proteomics, Metabolomics, Lipidomics	Diagnosis	Protein 14-3-3 zeta/delta, clusterin, interleukin-15, and transgelin-2	· Enrichment pathway analysis reveals overexpression of hemostatic, immune response, and extracellular matrix signaling pathways associated with AD.<br>· Protein 14-3-3 zeta/delta, clusterin, interleukin-15, and transgelin-2 improve AD prediction.	N	Clark C et al.[92]
**AD**	Proteomics, Metabolomics, Lipidomics	Diagnosis	GABA synthesis, arginine biosynthesis, and alanine, aspartate, glutamate, and arginine pathways	· Gender-dependent effects are seen on the pathways of significant enrichment, including those of GABA synthesis, arginine biosynthesis, and alanine, aspartate, glutamate, and arginine metabolism.<br>· Lysophospholipid and amino acid metabolism are involved in the AD brain.	Y (Mouse Model)	Abigail Strefeler et al.[93]
**AD**	Genomics, Transcriptomics	Treatment	*TRPV1*	· *TRPV1* activation rescues memory deficits and neuronal loss in *APOE4* high-fat diet-fed mice.<br>· Neuronal loss increases in *APOE4* high-fat diet mice, rescued by *TRPV1* activation in the capsaicin group.<br>· *TRPV1* is a treatment option for AD disease.	Y (Mouse Model)	Chenfei Wang et al.[94]
**AD** (late-onset)	Genomics, Transcriptomics	Treatment	*ATP6V1A*	· *ATP6V1A* has been identified as a key regulator of the top neuronal subnetwork, which is the most dysregulated in late-onset AD.<br>· *ATP6V1A* can be used as a therapeutic target.<br>· NCH-51 ameliorates neuronal damage caused by *ATP6V1A* deficiency in a Drosophila model.	Y (Drosophila Model)	Wang M et al.[95]

AlzGPS: Alzheimer's disease genome-wide positioning systems platform; CSF: cerebrospinal fluid; PKM2: pyruvate kinase M2; *MBP*: Myeloid basic protein encoding gene.

Multi-omics technologies integration

Supplementary Table S2: Application of Multi-omics and High-spatial-resolution Omics Technologies in PD.

Disease	Omics Type	Purpose	Biomarker	Relevance to PD Pathogenesis	Experiment Validation	Reference
**PD**	Genomics	Diagnosis	*ZNF184, IL1R2, LRRK2, ITPKB,* and *PARK16*	· Alleles of *LRRK2* and *IL1R2* confer a higher risk of developing PD.<br>· The genotype models of *ZNF184, PARK16,* and *ITPKB* are significantly associated with PD.<br>· Most of these genes are involved in autophagy and lysosomal function-related pathways.	N	Gao T et al.[102]
**PD**	Genomics	Diagnosis	*HLA, LRRK2, MAPT, TRIM10,* and *SETD1A*	· *HLA, LRRK2, MAPT, TRIM10,* and *SETD1A* are high-risk genes associated with PD.<br>· Significantly associated loci linked to PD are found in the *HLA* and *MAPT* gene loci.	N	Witoelar A et al.[103]
**PD**	Transcriptomics	Diagnosis	*SSR1*	· *SSR1* is found to be upregulated in PD patients.<br>· *SSR1* expression is negatively correlated with dopaminergic neuron survival.<br>· The upregulation of *SSR1* expression in peripheral blood precedes the abnormal behavior of the animals.<br>· The *SSR1*-based RF classifier has an AUC value of 0.91 and can be used as a diagnostic marker.	N	Zhang W et al.[104]

**PD**	Proteomics	Diagnosis	OMD, CD44、VGF, PRL, MAN2B1, and LRRK2	· ML identifies that OMD, CD44, VGF, PRL, and MAN2B1 show significant changes in PD patients and are significantly correlated with PD clinical scores.<br>· The enhanced neuroinflammatory characteristics in LRRK2 gene carriers are strongly correlated with PD.<br>· OMD, CD44, VGF, PRL, MAN2B1, and LRRK2 can be used as biomarkers for PD.	N	Karayel, MM et al.[105]
**PD**	Metabolomics	Diagnosis	Lipid metabolism related to carnitine shuttle, sphingolipid metabolism, and arachidonic acid metabolism	· Alterations in lipid metabolism related to carnitine shuttle, sphingolipid metabolism, arachidonic acid metabolism, and fatty acid biosynthesis are detected.<br>· Carnitine shuttling is the most important pathway associated with unmedicated PD patients by sebum.	N	Sinclair E et al.[106]
**PD**	Metabolomics	Diagnosis	Short-chain fatty acids, butyric acid	· Low levels of short-chain fatty acids are significantly associated with cognitive decline in PD patients.<br>· Decreased butyric acid levels are associated with poorer posture and gait disorder scores.<br>· Short-chain fatty acids and butyric acid serve as a potential diagnostic target.	N	Tan AH et al.[107]
**PD**	Metabolomics	Diagnosis	Proline	· Energy and lipid metabolism are overexpressed in PD.<br>· 139 metabolites, including proline, have notable changes in carnitine shuttle, vitamin E metabolism, lipid-related, glycerol phospholipids, sphingolipids, and fatty acids pathways.<br>· Proline and 139 other metabolites are considered particularly predictive of PD status.	Y (Independent Cohort)	Pereira, P.A.B et al.[108]

Multi-omics technologies integration

**PD**	Metabolomics	Diagnosis	Phenylacetic acid, phenylacetylglutamine, histidine, uric acid, and imidazoleacetic acid	· 45 metabolic markers in PD patients show high diagnostic power in early stages (AUC=0.92).<br>· Metabolites like phenylacetic acid, phenylacetylglutamine, histidine, uric acid, and imidazoleacetic acid show upregulated urine levels in PD, linked to neuro disorders.	N	Cai Z et al.[109]
**PD**	Metabolomics	Diagnosis	BCAA metabolism, glycine derivatives, steroid hormone biosynthesis, tryptophan, and phenylalanine metabolism	· 18 differential metabolites in urine have been identified as biomarkers for PD.<br>· Differential metabolites alter metabolic pathways associated with BCAA metabolism, glycine derivatives, steroid hormone biosynthesis, tryptophan metabolism, and phenylalanine metabolism.	N	Cai Z et al.[110]
**PD**	SnRNA-seq	Diagnosis	*IL1B, GPNMB,* and *HSP90AA1*	· A neuron cluster characterized by *CADPS2* overexpression and low tyrosine hydroxylase levels is identified in PD.<br>· Astrocytes and microglia in PD show specific proliferation and gene dysregulation linked to unfolded protein response and cytokine signaling.<br>· Microglia show a pro-inflammatory state with high *IL1B, GPNMB,* and *HSP90AA1*, suggesting their diagnostic potential.	N	Semra Smajić et al.[111]
**PD**	ScRNA-seq	Treatment	*HSP90* inhibitors	· Neurons and glial cells in PD exhibit dysfunction, immune dysregulation, and impaired protein folding.<br>· Administration of *HSP90* inhibitors accelerates the degradation of inflammasomes, reducing inflammatory responses and alleviating neurodegeneration.	Y (Mouse Model)	Gabriel GE et al.[112]

Multi-omics technologies integration

**PD**	Single-cell genomics, Spatial transcriptomics	Diagnosis	*TP53, NR2F2*	· The AGTR1-marked SNpc ventral subtype is highly PD-susceptible, showing *TP53/NR2F2* target gene upregulation.<br>· *TP53/NR2F2*-regulated pathways are key to PD-related neuronal death.<br>· *TP53/NR2F2* target gene upregulation indicates diagnostic biomarker potential.	Y (Macaque Model)	Tushar Kamath et al.[113]
**PD**	ScRNA-seq, Proteomics	Diagnosis	*SYN2*	· Negative correlation between α-synuclein pathology and chaperone protein expression in excitatory neurons in PD, along with weakened neuron-astrocyte interaction and aggravated neuroinflammation.<br>· *SYN2* enrichment in PD brain regions suggests significant increase in synaptic signaling at both RNA and protein levels.<br>· *SYN2* as a potential diagnostic biomarker for PD	Y (Independent Cohort)	Biqing Zhu et al.[114]
**PD**	Proteomics, Transcriptomics	Diagnosis	*GPNMB, CD38,* and *DGKQ*	· *GPNMB* and *CD38* show significant causal effects in PD, with evidence from quantitative trait locus analysis and fine mapping.<br>· *GPNMB, CD38,* and *DGKQ* proteins are associated with PD risk.	Y (Three Independent Datasets)	Guxiaojing et al.[115]
**PD**	Three Proteomics	Diagnosis	DDC	· DDC, SUMF1, DPP7, ENPEP, WFDC2, and hundreds of proteins are upregulated in the CSF, blood, or urine of PD patients.<br>· DDC levels are linked to symptom severity in PD patients.<br>· DDC can serve as a target for accurate PD diagnosis.	Y (Seven Independent Datasets)	Rutledge J et al.[116]

Multi-omics technologies integration

**PD**	Transcriptomics, Metabolomics	Treatment	The relaxin signaling pathway, adhesion patch, and PI3K-Akt signaling pathway	· BHD reduces PD symptoms, impacting metabolic pathways, including the relaxin signaling pathway, adhesion patch, and *PI3K-Akt* signaling pathway.<br>· BHD promotes the survival of dopaminergic neurons in PD mice, leading to improved motor performance.	Y (Mouse Model)	Hujun et al.[117]
**PD**	Genomics, Metabolomics	Diagnosis/ Treatment	*CircSV2b*	· Detect 33 deregulated circular RNAs in the PD mouse model vs wild-type controls.<br>· *CircSV2b* overexpression via the ceRNA-Akt1 axis mitigates oxidative stress in PD.<br>· *CircSV2b* is a potential Parkinson's diagnostic and curative biomarker.	Y (Mouse Model)	Cheng Qc et al.[118]

BCAA: branched chain amino acid; SNpc: substantia nigra pars compacta; DDC: Dopamine decarboxylase; ML: machine learning; *LRRK2*: leucine-rich repeat kinase 2; SUMF1: sulfatase-modifying factor 1; DPP7: dipeptidyl peptidase 2/7; *SSR1*: signal sequence receptor subunit 1; ENPEP: glutamyl aminopeptidase; WFDC2: WAP four-disulfide core domain 2; BHD: Buyang Huanwu Decoction.

Multi-omics technologies integration

Supplementary Table S3: Application of Multi-omics and High-spatial-resolution Omics Technologies in Epilepsy.

Disease	Omics Type	Purpose	Biomarker	Relevance to Epilepsy Pathogenesis	Experiment Validation	Reference
**Epilepsy**	Transcriptomics	Diagnosis	*P38MAPK, JAK-STAT, PI3K*, and mTOR signal pathway	· The*P38MAPK, JAK-STAT,* and *PI3K* consistently exhibit high expression and along with stable regulation of mTOR signaling pathways in epilepsy patients.<br>· Differential genes engage in signal cascades, ECM remodeling, cell motility, apoptosis, and immune responses linked to seizures.	N	Oswaldo K Okamoto et al.[122]
**Epilepsy** (temporal lobe)	Transcriptomics	Diagnosis	*Tlr2, Lgals3, Serpine 1* and *Stat3* et al.	· Several hub genes identified in TLE, such as *Tlr2, Lgals3, Serpine1,* and *Stat3*, et al., positively correlate with seizure frequency.<br>· Activation and phagocytic activity of microglia/macrophages have changed during the epileptic occurrence process of TLE.<br>· *Tlr2, Lgals3, Serpine 1,* and *Stat3* can serve as markers for TLE.	N	QingLan Chen et al.[123]
**Epilepsy**	Transcriptomics	Diagnosis	*GABAergic*	· FBTCS+ patients exhibit more widespread bilateral cortical and subcortical morphological alterations compared to FBTCS− patients.<br>· Excitatory and inhibitory neurons are affected in FBTCS−, while only excitatory neurons are significantly altered in FBTCS+.<br>· Excitatory/inhibitory imbalance and *GABAergic* dysfunction may underlie FBTCS susceptibility.	N	Lin, Q et al.[124]

**Epilepsy**	Transcriptomics	Diagnosis	*NMDAR*	· Analyses identified upregulation of the *NMDAR* signaling pathway as a key mechanism underlying both the autism-like behaviors and the observed anti-epileptic phenotypes.<br>· Impaired *GABAergic* function and enhanced *NMDAR* activity disrupt excitatory-inhibitory balance, promoting autism-epilepsy comorbidity.<br>· Dysfunctional *GABAergic* signaling and elevated *NMDA* activity contribute to co-occurrence of autism and epilepsy.	Y (Mouse Model)	Fan, C et al.[125]
**Epilepsy** (temporal lobe)	Transcriptomics	Diagnosis	*RBFOX1*	· TLE shows synaptic network reorganization, reduced connectivity, lower clustering, longer pathways, mainly in temporolimbic and frontoparietal regions.<br>· Identified 183 downregulated synaptic genes: *RBFOX1* and other *GABAergic* genes are central.<br>· Coordinated downregulation of risk genes may drive synaptic dysfunction and epilepsy in TLE, suggesting new treatment targets.	Y (Two Independent Datasets)	Li, R et al.[126]
**Epilepsy**	Proteomics	Diagnosis	GFAP	· GFAP is consistently downregulated in brain tissue with high spike frequencies and exhibits a strong negative correlation with spike frequency.<br>· Reactive astrocytes, such as GFAP, protect the neocortex from epileptic discharges rather than induce them.<br>· Epilepsy severity is closely linked to decreased GFAP (astrocyte marker) levels.	N	Gal Keren-Aviram et al.[127]

Multi-omics technologies integration

**Epilepsy**	Proteomics	Diagnosis/ Treatment	ADPRC, LPAR3, calreticulin, UCH-L1, SNAP-25, and transgelin-3	· A total of 144 differentially expressed proteins, such as ADPRC, LPAR3, calreticulin, UCH-L1, SNAP-25, and transgelin-3, are identified in the epileptic hippocampal regions.<br>· Most differentially expressed proteins are associated with Ca2+ homeostasis.<br>· Inhibiting calcium influx alleviates seizures triggered by excessive brain Ca^{2+} rise in epilepsy.	N	Leila Sadeghi et al.[128]
**Epilepsy**	Proteomics	Diagnosis	Calcineurin	· Tutin induces epilepsy by activating calcium-modulating phosphatase and produces significant neurological damage.<br>· Calcineurin is a target of tutin, and that tutin activates Calcineurin, leading to seizures.	Y (Mouse Model)	Shi-Shan Yu et al.[129]
**Epilepsy**	Metabolomics	Diagnosis	N-acetyl glycoprotein, lactate, creatine, glycine, lipid, and citrate	· Serum N-acetyl glycoprotein, lactate, creatine, glycine, and lipid levels are elevated decreased levels of citrate in epileptic children, while the level of citrate is reduced.<br>· The aforementioned metabolic substances are potential diagnostic targets for epilepsy.	N	Łukasz Boguszewicz et al.[130]
**Epilepsy (mesial temporal lobe)**	Metabolomics	Diagnosis	GABA	· GABA is significantly increased in the epileptogenic zone of KA-MTLE mice.<br>· GABA is a specific biomarker of the epileptogenic zone in MTLE.	Y (Mouse Model)	Hamelin, S et al.[131]
**Epilepsy**	SnRNA-seq	Diagnosis	*Sst* and *Pvalb*	· Major transcriptomic alterations occur in principal neurons (*L5-6_Fezf2, L2-3_Cux2*) and *GABAergic* interneurons (*Sst, Pvalb*).<br>· Profound dysregulation in glutamate signaling, characterized by robust upregulation of glutamate receptor genes, notably within *Sst/Pvalb* subtypes.<br>· *Sst/Pvalb* interneurons represent potential diagnostic targets and are fundamental to early epileptogenesis.	N	Ulrich Pfisterer et al.[132]

Multi-omics technologies integration

**Epilepsy** (post-traumatic)	ScRNA-seq	Diagnosis	*XIST*	· Hereditary epilepsy shows higher oligodendrocyte/astrocyte counts, lower microglia/neuron counts vs PTE.<br>· *IL-17* signaling in microglia/astrocytes can be a PTE target/biomarker.<br>· *XIST*, upregulated in PTE, drives inflammation/fibrosis, useful for diagnosis and mechanism study.	N	Fang Wen et al.[133]
**Epilepsy** (temporal lobe)	ScRNA-seq, SnRNA-seq, Spatial transcriptomics	Diagnosis	*SPP1, Trem2, Tle4 and Sipa1l3*	· The differentially up-regulated genes in TLE patients are predominantly expressed in glial cells, while the down-regulated genes are mainly expressed in neurons.<br>· *SPP1* and *Trem2* are up-regulated in glial cells, whereas *Tle4* and *Sipa1l3* are down-regulated in these cells.	N	Quanlei Liu et al.[134]
**Epilepsy**	Genomics, Transcriptomics	Diagnosis	*Sestrin 3*	· *Sestrin 3* is a key regulator in the pro-convulsant gene network in the hippocampus of human epilepsy.<br>· *Sestrin 3* positively regulates modules in macrophages, microglia, and neurons.<br>· *Sestrin 3* holds potential as a diagnostic means for epilepsy.	Y (Mouse Model)	Johnson, M.R. et al.[135]
**Epilepsy**	Proteomics, Transcriptomics	Diagnosis	*STAT3, ErbB,* and *MAPK8*	· The TGF-β pathway is associated with cardiac function in the hearts of epileptic animals.<br>· *STAT3, ErbB,* and *MAPK8* are key regulators of cardiac alterations in epilepsy that contribute to seizure-mediated cardiac damage.	N	Sharma, S et al.[136]

Multi-omics technologies integration

**Epilepsy**	Proteomics, Metabolomics	Diagnosis	GSTM1, ALDH2	· Within the somatosensory cortex module, GSTM1 is identified as a protein hub and elevated expression levels.<br>· In the thalamus module, ALDH2 is pinpointed as a protein hub.<br>· The metabolic pathway enriched by the differences is lysine degradation.<br>· GSTM1 and ALDH2 are identified as markers for seizure-related modules in epilepsy.	N	Harutyunyan, A et al.[137]
**Epilepsy**	Genomics, Metabolomics	Diagnosis	Lactate, creatine, phosphocreatine, and choline	· Lactate is significantly reduced, while creatine, phosphocreatine, and choline are significantly increased.<br>· Lactate is involved in G protein-coupled receptor signaling and angiogenic pathways, and shows upregulation of ubiquitination-related genes.	N	Wu, H.C. et al.[138]
**Epilepsy**	Proteomics, Transcriptomics	Treatment	*miR-10a-5p, miR-21a-5p* and *miR-142a-5p*	· *miR-10a-5p, miR-21a-5p,* and *miR-142a-5p* are identified as key transcripts.<br>· These microRNA transcripts are primarily associated with the TGF-β pathway signaling.<br>· The combination of anti-miR (*miR-10a-5p, miR-21a-5p, miR-142a-5p*) exhibits protective effects against acute and spontaneous seizures.	Y (Mouse Model)	Venø, M.T. et al.[139]

PTE: post-traumatic epilepsy; TLE: temporal lobe epilepsy; MTLE: mesial temporal lobe epilepsy; NMDA: N-methyl-D-aspartate; GABA: γ-aminobutyric acid; *GFAP*: glial fibrillary acidic protein; KA-MTLE: kainic acid into mesiotemporal lobe epilepsy mice; TGF-β, transforming growth factor β; ADPRC: ADP-ribosyl cyclase; FBTCS: focal to bilateral tonic–clonic seizures; LRRK2: leucine-rich repeat kinase 2; LPAR3: lysophosphatidic acid receptor 3; UCH-L1: ubiquitin carboxyl-terminal hydrolase L1; GSTM1: glutathione s-transferase M1; SNAP-25: synaptosome-associated protein 25.

Multi-omics technologies integration

Supplementary Table S4: Application of Multi-omics and High-spatial-resolution Omics Technologies in MS.

Disease	Omics Type	Purpose	Biomarker	Relevance to MS Pathogenesis	Experiment Validation	Reference
**MS**	Genomics	Diagnosis	*STAT3, IL7*	· Inhibitory neurons in the CNS are key contributors to MS susceptibility.<br>· Genes such as *STAT3* and *IL7* disrupt immune pathways specifically in inhibitory neurons.<br>· Targeted interventions for CNS pathways—such as neuronal and glial functions, including *STAT3* and *IL7*.	Y (Three Independent Datasets)	Philip De Jager et al.[146]
**MS**	Proteomics	Diagnosis	CXCL13, LTA, FCN2, ICAM3, LY9, SLAMF7, TYMP, CHI3L1, FYB1, TNFRSF1B, and NFL	· Lower levels of *NFL* in CSF show predictive potential for disease activity (AUC=0.77).<br>· An 11-protein panel in CSF has a high AUC for prediction, including CXCL13, LTA, FCN2, ICAM3, LY9, SLAMF7, TYMP, CHI3L1, FYB1, TNFRSF1B, and NFL (AUC=0.9).<br>· All the above proteins can be markers for MS.	N	Mika Gustafsson et al.[147]
**MS**	Metabolomics	Diagnosis	DRD2	· DRD2 exacerbates the disease by promoting inflammation and reducing the abundance of Lactobacillus species in the microbiome.<br>· Lactobacillus-derived N2-acetyl-L-lysine inhibits microglial activation, combating neurodegeneration.<br>· Intestinal epithelial DRD2, serving as a biomarker, can modulate the gut microbiome in MS.	N	Hairong Peng et al.[148]

Multi-omics technologies integration

**MS** (relapsing-remitting)	Metabolomics	Diagnosis/ Treatment	Glycolysis	· Identified four perturbed metabolic pathways, including structural/signaling lipids and energy, in the serum of patients with MS.<br>· Glycolysis is the common upstream feeding of these altered metabolic pathways.<br>· Targeting glycolysis in experimental autoimmune encephalomyelitis ameliorated the disease pathology by impeding immune cell effector function.	Y (Mouse Model)	Insha Zahoor et al.[149]
**MS**	ScRNA-seq	Diagnosis	*TFH*	· Myeloid dendritic cells and regulatory T cells are enriched in the CSF of patients with MS.<br>· The independent increase in clusters of TFH cells drives the known expansion of B-lineage cells in the CSF in MS.<br>· TFH cells promote the infiltration of B cells into the central nervous system, exacerbating MS disease.	Y (Mouse Model)	David Schafflick et al.[150]
**MS**	ScRNA-seq, Spatial transcriptomics	Diagnosis	*SERPINA3*	· Astrocytes can be classified into three types: homeostatic, intermediate, and disease-associated types.<br>· In patients with DA-Astro, the expression level of *SERPINA3* is significantly elevated.<br>· *SERPINA3* expression may constitute a glial cell survival response to resolve inflammation and prevent apoptosis during both initial and late resolution phases.	N	Petra Kukanja et al.[151]

Multi-omics technologies integration

**MS**	ScRNA-seq, Spatial transcriptomics	Diagnosis	*MAFB*	· The expression of pro-inflammatory molecules in oligodendrocytes near axonal damage is elevated in MS patients.<br>· *MAFB* mediates intercellular communication via complement factors and apolipoproteins.<br>· The inflammatory transcription factor *MAFB* serves as a biomarker for MS lesions.	N	Maria L Elkjaer et al.[152]
**MS (systemic)**	ScRNA-seq, Spatial transcriptomics, Spatial proteomics	Diagnosis/ Treatment	*POSTN/SCARA5, CXCR4*	· A dynamic spatial interaction network is established between fibroblasts and macrophages via the *ACKR3-CXCL12-CXCR4* signaling axis, playing a central role in driving fibrosis progression.<br>· Treatment with the *CXCR4* inhibitor AMD3100 significantly alleviates fibrosis in skin and lung tissues.<br>· The significantly elevated *POSTN/SCARA5* ratio in MS can serve as a predictive diagnostic biomarker.	Y (Mouse Model)	Zhijian Li et al.[153]
**MS**	Proteomics, Transcriptomics	Diagnosis	*GPR37L1, SIRPA, FGFR3, CADM3,* and *TYRO3*	· Neurological candidate molecules, including *GPR37L1, SIRPA, FGFR3, CADM3*, and TYRO3, are highly expressed in the CNS of MS.<br>· These genes are associated with early neuronal degeneration and dysfunctional trophic/anti-inflammatory intercellular communication.<br>· *GPR37L1, SIRPA, FGFR3, CADM3,* and *TYRO3* can be used as a diagnostic method for MS.	N	Max Kaufmann et al.[154]

Multi-omics technologies integration

**MS**	Proteomics, Transcriptomics	Diagnosis	24 iron death-related genes (*CHMP5, SLC38A1, PML*, etc.)	· High iron death scores at the margins of active lesions correlate with phagocytic activation.<br>· Elevated iron death scores in cortical neurons are associated with neurological diseases.<br>· A blood-based model of 24 iron death-related genes is a prognostic marker for diagnosing MS, including *CHMP5, SLC38A1, PML*, etc.	N	Tao Wu et al[155]
**MS**	Proteomics, Metabolomics	Diagnosis	LAMP1, FCG2A, and HPSE	· *HPSE* is positively correlated with many MS-related metabolites, including L-tyrosine, sphingosine 1-phosphate, sphingosine 1-phosphate, and L-tryptophan.<br>· The proteins LAMP1, FCG2A, and HPSE exhibit potential utility as specific biomarkers for MS.	N	Fan Yang et al.[156]
**MS**	Proteomics, Metabolomics	Diagnosis	Equine uric acid, sphingolipids	· Anti-inflammatory molecules and sphingolipids are reduced by metabolomics in MS patients.<br>· Low levels of equine uric acid in a severe subgroup of MS.<br>· Sphingolipids and equine uric acid facilitate the future development of biomarkers and targeted therapeutic interventions for MS.	N	Qinming Zhou et al.[157]

NFL: neurofilament light chain; DRD2: Dopamine Receptor D2; CNS: central nervous system; HPSE: heparinase; TFH: T follicular helper.

Multi-omics technologies integration

Supplementary Table S5: Application of Multi-omics and High-spatial-resolution Omics Technologies in Stroke.

Disease	Omics Type	Purpose	Biomarker	Relevance to Stroke Pathogenesis	Experiment Validation	Reference
**Stroke** (ischemic)	Proteomics	Diagnosis	NSF, RhoGDI1, and RabGDI	· Circulating NSF, RhoGDI1, and RabGDI are upregulated in patients with IS.<br>· These proteins trigger neuronal depolarization and calcium surge, activating death pathways in stroke.	Y (Independent Cohort)	Eloy Cuadrado et al.[160]
**Stroke** (ischemic)	Proteomics	Diagnosis	CMPK, CKB	· Circulating levels of CKB and CMPK are higher in patients with ischemic stroke than in controls during the acute phase.<br>· CKB plays a crucial role in energy transduction and homeostasis.<br>· CMPK is released in large amounts and participates in mechanisms that counteract cell disruption and neuronal cell death.	N	Alba Simats et al.[161]
**Stroke** (ischemic)	Proteomics	Diagnosis	SAHH2	· SAHH2 plays a significant role in the coordinated inhibition of $Ca^{2}+$ ion transporters.<br>· Increased expression of SAHH2 in neurons from the infarcted area is probably because of ischemia-triggered Ca2+ mobilization.	N	Teresa García-Berrocoso et al.[162]
**Stroke**	Transcriptomics	Diagnosis	LncRNA (*MEG3, H19,* and *MALAT1*)	· LncRNAs, such as *MEG3, H19,* and *MALAT1*, in blood cells between patients with stroke and healthy controls show differences.<br>· Differential genes modulate neuronal survival/apoptosis targets, impacting p53-mediated apoptosis in stroke.	N	Cheryl Dykstra Aiello et al.[163-167]

Multi-omics technologies integration

**Stroke**	Transcriptomics	Diagnosis	Extracellular microRNA	· Decreased levels of extracellular *miR-32-3p, miR-106b-5p, miR-423-5p, miR-451a, miR-1246, miR-1299, miR-3149* and *miR-4739*, and increased levels of extracellular *miR-224-3p, miR-377-5p, miR-518b, miR-532-5p* and *miR-1913* associate with stroke.<br>· These genes affect multiple pathways such as apoptosis, oxidation, angiogenesis, and neurogenesis in IS.	N	Ceren Eyileten et al.[168]
**Stroke**	Transcriptomics	Diagnosis	*IFN-I*	· Aged brains show marked upregulation of *IFN-I* signaling following ischemic injury.<br>· Aging leads to downregulation of genetic programs essential for axonal and synaptic integrity after stroke.<br>· *IFN-I* may serve as a diagnostic biomarker for stroke.	N	Androvic, P et al.[169]
**Stroke** (cardioembolic)	Metabolomics	Diagnosis	Valine, Leucine, Isoleucine	· The expression levels of BCAA, including valine, leucine, and isoleucine, are decreased in patients with cardioembolic stroke.<br>· Lower BCAA levels are also associated with poor neurological outcomes.	N	W Taylor Kimberly et al.[170]
**Stroke**	Metabolomics	Diagnosis	Total free fatty acid	· Plasma concentration of total free fatty acids is higher in patients with cardioembolic stroke than in patients with non-cardioembolic stroke.<br>· Elevated free fatty acid levels are significantly associated with cardioembolic stroke, suggesting their potential as a diagnostic target.	N	Jeong Yoon Choi et al.[171]

Multi-omics technologies integration

**Stroke**	ScRNA-seq	Treatment	Microglia and macrophages	· Aging jeopardizes the repair and regeneration of the cerebrovascular system and proteins after stroke.<br>· After stroke, microglia and macrophages may affect angiogenesis and oligodendrogenesis via paracrine mechanisms, impeding stroke recovery.<br>· Transplanting microglia and macrophages from the brains of young mice into the cerebral cortex of aged stroke-affected mice partially restores angiogenesis and oligodendrogenesis.<br>· Microglia and macrophages serve as effective targets for promoting stroke recovery	N	Chenghao Jin et al.[172]
**Stroke** (ischemic)	ScRNA-seq, Spatial transcriptomics	Treatment	*LILRB4*	· Stroke brains have up-regulated *LILRB4* and ischemia-linked microglial cluster 3.<br>· *LILRB4* knockout worsens ischemic brain injury via CD8+ T cell recruitment; overexpression offers neuroprotection.<br>· Targeting *LILRB4* and its downstream pathways represents an effective therapeutic strategy for ischemic stroke.	N	Yilin Ma et al.[173]
**Stroke** (brainstem)	SnRNA-seq, ScRNA-seq	Treatment	Myo1e	· Oligodendrocyte loss leads to neurological deficits following brainstem stroke.<br>· *OLG8* has an innate neuroprotective effect in brainstem stroke.<br>· Myo1e aids *OLG8* migration to the peri-infarct area in brainstem stroke.<br>· Myo1e overexpression in *OLG8* oligodendrocytes boosts brainstem stroke recovery.	N	Shaojun Li et al.[174]

Multi-omics technologies integration

**Stroke** (intracerebral hemorrhage)	ScRNA-seq, Spatial transcriptomics	Diagnosis	*SPP1, Lyz2*	· *SPP1/Lyz2* show high expression levels, and lymphocytes with high expression interact with myeloid cells in the late stage of stroke.<br>· During the acute phase of intracerebral hemorrhage, Lgmn+Macro-T cells and microglia interact via the *SPP1-cd44* pathway.<br>· *SPP1* and *Lyz2* are potential diagnostic targets for the acute phase of intracerebral hemorrhage.	N	Lingui Gu et al.[175]
**Stroke**	ScRNA-seq, Spatial transcriptomics	Treatment	Lipocalin-2	· Ferroptosis is the primary programmed cell death process post-hemorrhagic stroke, mainly affecting mature oligodendrocytes.<br>· A specific interaction between lipocalin-2-positive microglia and oligodendrocytes, mediated by the CSF1 receptor pathway, induces ferroptosis in oligodendrocytes and subsequent neurological deficits.<br>· Early therapeutic intervention by inhibiting *LCN2* expression may alleviate ferroptosis-induced oligodendrocyte damage and related neurological deficits.	N	Lingui Gu et al[176]
**Stroke**	ScRNA-seq, Spatial transcriptomics	Treatment	*LGALS9*	· Galectin (*LGAL*) signaling is enhanced in microglia and macrophages of ischemic mice.<br>· *LGALS9* treatment promotes oligodendrocyte remyelination and improves stroke recovery in mice.<br>· *LGALS9* can serve as a therapeutic approach to ameliorate stroke.	N	Bing Han et al.[177]

Multi-omics technologies integration

**Stroke (subarachnoid hemorrhage)**	ScRNA-seq, Spatial transcriptomics	Diagnosis	*THBS1, S100A6*	· *THBS1* and *S100A6* are closely associated with the prognosis of SAH, with their expression significantly increasing following the hemorrhage.<br>· The *THBS1-CD47* pair regulates cell apoptosis, and blocking their interaction may represent a new therapeutic approach for SAH.<br>· *THBS1* and *S100A6* serve as diagnostic biomarkers for stroke.	N	Xiaoyu Wang et al.[178]
**Stroke**	Genomics, Spatial transcriptomics	Diagnosis	*MMP-9*	· Plaque rupture occurs predominantly in proximal and most stenotic areas.<br>· Identified *MMP-9* as a key gene causally linked to rupture risk.<br>· Supports targeted intervention against *MMP-9* for precise stroke treatment.	N	Sun, J et al.[179]
**Stroke**	ScRNA-seq, Spatial transcriptomics	Diagnosis	*APOE*, *FABP5*	· Distinct astrocyte states were identified post-stroke, influenced by both time and proximity to the ischemic lesion.<br>· Proximal astrocytes exhibited functional divergence in lipid transport, characterized by elevated expression of *APOE* and *FABP5* after cortical ischemic stroke.<br>· *APOE* and *FABP5* hold promise as diagnostic biomarkers for stroke.	N	Scott, EY et al.[180]
**Stroke** (cardioembolic)	Proteomics, Transcriptomics	Diagnosis	*ICA1L, CAND2,* and *ALDH2*	· Reduced *ICA1L, CAND2,* and *ALDH2* may impair excitatory synaptic signaling, contributing to cardioembolic stroke pathogenesis.<br>· *ICA1L, CAND2,* and *ALDH2* are potential biomarkers for lacunar stroke.	Y (Three Independent Datasets)	Zhang C et al.[181]

Multi-omics technologies integration

**Stroke** (ischemic)	Genomics, Metabolomics	Treatment	Gut flora and metabolic disturbances	· ZHTC modulates the abundance of specific bacterial groups and 23 metabolic differences for IS, including arginine, L-lysine, and L-methionine.<br>· ZHTC improves intestinal barrier integrity by increasing the expression levels of tight junction proteins<br>· ZHTC meliorates IS by modulating gut flora and metabolic disturbances.	Y (Rat Model)	Wang R et al.[182]
**Stroke** (ischemic)	Proteomics, Transcriptomics	Treatment	*PI3K-Akt, MAPK*, and cAMP signaling pathways	· *YQTL* reduces infarct volume percentage and improves neurological function in cerebral ischemia-reperfusion injury mice.<br>· Network pharmacology and multi-omics studies reveal 15 components that regulate 82 targets and 19 pathways.<br>· *YQTL* protects against cerebral ischemia-reperfusion injury through *PI3K-Akt, MAPK*, and cAMP signaling pathways.	Y (Mouse Model)	Yuan Y et al.[183]

lncRNAs: long non-coding RNAs; IS: Ischemic Stroke; BCAA: branched-chain amino acid; *LGAL*: Galectin; *CSF1*: colony-stimulating factor 1; *SAH*: subarachnoid hemorrhage; ZHTC: Zhilong Huoxue Tongyu capsule; YQTL: Yiqi Tongluo granule; *ALDH2*: aldehyde dehydrogenase 2.

Multi-omics technologies integration

Supplementary Table S6: Application of Multi-omics and High-spatial-resolution Omics Technologies in Hydrocephalus.

Disease	Omics Type	Purpose	Biomarker	Relevance to Hydrocephalus Pathogenesis	Experiment Validation	Reference
**Hydrocephalus** (communicating)	Genomics	Diagnosis	*TRIM71, SMARCC1, PIK3CA, PTEN, MTOR, FOXJ1, FMN2, PTCH1,* and *FXYD2*	· *TRIM71* and *SMARCC1* exhibit genome-wide significant enrichment of de novo mutations, which may be genuine risk factors for CH.<br>· *PIK3CA, PTEN, MTOR, FOXJ1, FMN2, PTCH1,* and *FXYD2* are newly identified high-confidence sporadic CH genes.<br>· *TRIM71* and other genes, reducing neural cell proliferation to cause hydrocephalus, can be a diagnostic marker.	N	Sheng Chih Jin et al.[191]
**Hydrocephalus** (communicating)	Proteomics	Diagnosis	KLK6	· Expression of KLK6 is significantly up-regulated in CH patients.<br>· KLK6 is involved in CH development and may provide a new target for CH diagnosis.	Y (Rat Model)	Lei Yuan et al.[192]
**Hydrocephalus** (idiopathic normal pressure)	Proteomics	Diagnosis	QPCT, RBP4	· 39 proteins exhibit a significant increase, while 285 proteins show a significant decrease in CSF of iNPH.<br>· Elevated proteins mainly relate to myeloid leukocyte migration and extracellular matrix organization; reduced ones are linked to axon and synaptic development.<br>· QPCT and RBP4 have been identified as potential protein biomarkers in iNPH for predicting shunt outcomes.	N	Yuqi Ying et al.[193]

**Hydrocephalus** (idiopathic normal pressure)	Proteomics	Diagnosis	PTPRQ	· PTPRQ concentrations in CSF are significantly higher in iNPH patients than in AD patients.<br>· PTPRQ concentration in the CSF of non-responders to shunt operation tended to be relatively lower compared with that in the responders.<br>· PTPRQ is a candidate biomarker to distinguish iNPH from AD.	N	Yuki Nagata et al.[194]
**Hydrocephalus** (idiopathic normal pressure**)**	Metabolomics	Diagnosis	Glyceric acid, N-acetyl neuraminic acid, serine, and 2-hydroxybutyric acid	· Elevated glyceric acid and N-acetyl neuraminic acid, and reduced serine and 2-hydroxybutyric acid in AD CSF distinguish it from iNPH.<br>· Serine, glyceric acid, Neu5Ac, and 2-hydroxybutyrate combine as a diagnostic iNPH biomarker.	N	Yuki Nagata et al.[195]
**Hydrocephalus** (normal pressure)	Metabolomics	Treatment	Neu5Ac	· CSF Neu5Ac levels are low in NPH patients.<br>· Boosting brain Neu5Ac inhibits astrocyte activation.<br>· Brain Neu5Ac elevation reduces periventricular demyelination and improves hydrocephalus.<br>· Enhanced brain Neu5Ac improves neurological outcomes in NPH, suggesting a potential treatment.	Y (Mouse Model)	Zhangyang Wang et al.[196]
**Hydrocephalus** (tumor-associated)	SnRNA-seq, Spatial transcriptomics	Diagnosis/Treatment	*CPMCs*	· Ventricular cell atlas reveals *CPMC* expansion in TAH mice.<br>· *CPMCs* compromise ependymal ciliary integrity via tryptase-PAR2-FOXJ1 signaling, triggering pathological CSF hypersecretion that underlies hydrocephalus pathogenesis.<br>· Brain barrier-penetrating trypsin-like inhibitor BMS-262084 effectively inhibits TAH progression in vivo and attenuates mast cell-induced epithelial cilia damage.	N	Yiye Li et al.[197]

Multi-omics technologies integration

**Hydrocephalus**	Genomics, scRNA-seq	Diagnosis	*MAEL*	· scRNA-seq data from the cortical plate and germinal matrix revealed robust *MAEL* expression within neurogenic niches.<br>· Reduced *MAEL* levels may induce genomic structural alterations, thereby impairing cortical development, volume, and function.<br>· Decreased *MAEL* expression is associated with the pathogenesis of hydrocephalus.	Y (Independent Cohort)	Hale, AT et al.[198]
**Hydrocephalus** (communicating)	Genomics, Proteomics, Transcriptomics	Diagnosis	*MAEL*	· PrediXcan analysis in 10 neuro tissues and whole blood shows a correlation between reduced *MAEL* gene expression in the brain and hydrocephalus ($p < 0.05$).<br>· Reduced *MAEL* expression increases susceptibility to hydrocephalus.<br>· *MAEL* is a diagnostic biomarker for hydrocephalus.	Y (Mouse Model)	Andrew T Hale et al.[199]
**Hydrocephalus** (post-hemorrhagic)	Proteomics, Metabolomics	Diagnosis/Treatment	CSPG4	· CSPG4 positively correlates with ventricular size and the incidence of periventricular leukomalacia.<br>· Silencing of CSPG4 can inhibit ferroptosis, cell adhesion functions, and intracellular Ca^{2+} flux.<br>· CSPG4 has been identified as a CSF biomarker and effective therapeutic target.	Y (Mouse Model)	Juncao Chen et al.[200]

NPH: normal pressure hydrocephalus; iNPH: idiopathic normal pressure hydrocephalus; CH: communicating hydrocephalus; *Neu5Ac*: N-acetylneuraminic acid; TAH: tumor-associated hydrocephalus; *CPMCs*: choroid plexus mast cells; MAEL: maelstrom spermatogenic transposon silencer; KLK6, kallikrein-6; *QPCT*: glutaminyl-peptide cyclotransferase; RBP4: retinol-binding protein 4; PTPRQ: Q-type protein tyrosine phosphatase receptor; CSPG4: chondroitin sulfate proteoglycan 4.

Figure Legends

Figure 1: Three main types of metabolomics, including targeted metabolomics, untargeted metabolomics, and widely-targeted metabolomics. This figure outlines these three metabolomics techniques, each with unique strengths and limitations. Careful selection among them can lead to the optimal choice to meet the specific requirements of your experimental objectives.

Figure 2: Research workflow and applications of high-spatial-resolution omics technologies: integrated development of single-cell and spatial omics. This figure illustrates the integrated research workflow of high-spatial-resolution omics technologies, combining single-cell omics technology with spatial omics approaches. Single-cell omics involves: (1) sample preparation; (2) single-cell isolation and labeling (utilizing representative technologies such as Drop-seq and 10x Genomics Chromium); (3) nucleic acid extraction and library construction; and (4) high-throughput sequencing to resolve cellular gene expression profiles and uncover cellular heterogeneity. In parallel, spatial omics employs: (1) tissue sample processing; (2) spatial labeling/capture (via techniques like 10x Visium, Slide-seq, and MERFISH); (3) sequencing/detection; and (4) computational data analysis to map gene expression with spatial coordinates, thereby elucidating tissue microenvironment interactions. The synergy between these approaches addresses complementary questions—who is expressing a gene versus & where expression occurs spatially—enabling breakthroughs in tumor biomarker identification, cellular subpopulation localization, and tissue developmental mechanisms, among others.

Figure 3: Applications of multi-omics and high-spatial-resolution omics technologies in the diagnosis and treatment of brain diseases in the field of neurology. By integrating the high-throughput omics technologies, including four basic omics, single-cell, and spatial omics technologies, it is possible to comprehensively dissect the complex pathogenic mechanisms of neurological disorders. This multi-omics approach spans multiple levels, from genetic variations to metabolic changes, and reveals the interactions between these levels, providing an unprecedented perspective for in-depth disease understanding. In the study of diseases such as AD, PD, stroke, epilepsy, MS, and hydrocephalus, the application of these cutting-edge technologies has greatly facilitated the

discovery of key biomarkers and significantly deepened our understanding of the molecular mechanisms of disease pathogenesis.

Figure 4: Pathogenesis of AD revealed by multi-omics and high-spatial-resolution omics technologies. This figure provides an overview of the pathogenesis of AD revealed by omics technologies, with a particular focus on several key aspects, including abnormal TAU protein, deposition of Aβ protein, and formation of neurofibrillary tangles, neuronal loss and degeneration, disorders of lysosomal-related metabolic pathways, and neuroinflammation. In the context of TAU protein, Aβ protein, and plaque accumulation, the M7 *MAPK* module and *STAT3* gene are involved. Moreover, *LRP1, FKBP1B, PSEN1, APP, PS1*, and TAU proteins constitute the main components of neurofibrillary tangles, and their abnormal alterations represent crucial pathological features of AD. The process of neuronal loss and degeneration involves *APOE4, ATP6V1A,* PKM2*, LINGO1, OLIG*, as well as histone modifications *H3K9ac, H4K16ac*, Myelination, and *SNPs*. These factors act in concert, leading to the impairment of neuronal functions and the decline of cognitive abilities. In the lysosome and glycolysis-related metabolic pathways, the abnormalities of FBP1, FBP2, RHOH, Lipid, SPI1/ PU.1SPI1, ELF2, RUNX1, CSTD, SPARC, CALB2*,* and CTSB*,* as well as the metabolic pathways of sphingolipids and aromatic amino acids, reflect cellular metabolic impairments observed in AD. In terms of neuroinflammation, *PBXIP1*, along with activated microglia and astrocytes, shows abnormal hyperactivity. *PTPRG/VIRMA* inhibitors show their impacts on mitochondrial function and neuronal survival, offering potential therapies for AD. Alterations in SST^+ and $Pvalb^+/Vip^+$ levels also show potential for the diagnosis of AD. By integrating multi-level data across the genome, transcriptome, proteome, and metabolome, multi-omics and high-spatial-resolution omics technologies provide a comprehensive view of AD pathogenesis, offering crucial insights for early diagnosis and precision therapy.

Figure 5: Multi-omics and high-spatial-resolution omics reveal key mechanisms, biomarkers, and risk factors in PD. PD is a complex neurodegenerative disorder influenced by genetic, environmental, and neurobiological factors. Mutations in genes such as *ZNF184, IL1R2, IL1B, GPNMB,* and *LRRK2* are central to PD risk prediction and genetic susceptibility. Aberrant expression of proteins like *GPNMB, CD38, SYN2, DGKQ*,

Multi-omics technologies integration

and biomarkers such as *MAPT, SSR1, TP53,* and *NR2F2* is linked to PD progression and diagnostic value. These changes drive neuroinflammation and immune activation. BBB disruption facilitates the infiltration of leukocytes and neutrophils, initiating neuroinflammation. Activated microglia and astrocytes release inflammatory cytokines (e.g., TNF-α, IL-1β, IL-6), which exacerbate the inflammatory environment and damage neurons. Lysosomal dysfunction, involving OMD, CD44, VGF, PRL, and MAN2B1, and ceRNA-Akt1 axis disruption, contribute to α-synuclein aggregation. Inflammatory biomarkers, including CircSV2b, DDC, Proline, BCAAs, and molecules in steroidogenesis and fatty acid catabolism, are also identified. Dysregulated short-chain fatty acid metabolism is associated with cognitive decline in PD. *HSP90* inhibitors show therapeutic potential, as suggested by scRNA-seq of neuronal heterogeneity and molecular pathways. This figure integrates multi-level omics data to illuminate PD pathogenesis, supporting early diagnosis, monitoring, and targeted treatment.

 Click here to access/download;Table;Table.docx

Table 1: Overview of the Four Omics Technologies.

Technology	Precision	Price Range	Advantages	Disadvantages	Disease Application Representation
**Genomics**	High	High	· Conduct a comprehensive analysis of genetic sequences.<br>· Uncover the depth of genetic variations.<br>· Be suitable for gene discovery and genetic disease studies.	· High experimental costs.<br>· Complex techniques and large sample sizes.<br>· Considerable time in analysis.	· Genetic disorders.<br>· Cancer genomics.<br>· Genetic counseling.
**Transcriptomics**	Medium	Medium	· Reveal dynamic changes in gene expression.<br>· Differentiate gene regulatory networks.<br>· Assist in the classification of disease subtypes.	· High experimental and data analysis design.<br>· Limited real-time to reflect mRNA levels.	· Mental disorders.<br>· Cardiovascular diseases.<br>· Prognostication and efficacy assessment in cancer.
**Proteomics**	Low	Medium	· Reflect protein levels and modifications directly.<br>· Reveal protein-protein interaction networks.<br>· Explore changes in protein function.	· Complex data analysis with low standardization.<br>· Effects of PTMs.	· Development of tumor biomarkers.<br>· Mechanistic studies in autoimmune diseases.<br>· Pathology of neurodegenerative diseases.
**Metabolomics**	Medium	Medium	· Provide the overall metabolic profile of the organism.<br>· Reflect metabolic changes associated with disease.<br>· Assist in early diagnosis and monitoring.	· Sensitive sample handling and storage conditions.<br>· Challenges in the detection of metabolites.	· Monitoring of endocrine disorders.<br>· Prediction of cardiovascular disease risk.<br>· Metabolic testing in diabetes.

**Single-cell Omics**	High	High	· Resolve cellular heterogeneity (tumor subclones).<br>· Identify rare cell types (<0.1% population).<br>· Enable multi-omics integration (ATAC+RNA).	· Spatial information loss from tissue dissociation<br>· Significant technical noise (dropout rate >15%)<br>· Single-cell amplification bias	· Tumor evolutionary tree construction.<br>· T-cell receptor clonal tracking.<br>· Nervous diseases neuronal subtyping.
**Spatial Omics**	High	High	· Preserve in-situ spatial topology.<br>· Quantify cell-cell interactions (immune synapses).<br>· Directly correlate pathological morphology with molecular expression.	· Resolution inversely proportional to throughput (e.g., MERFISH: ~1,000 genes).<br>· Optical diffraction limitations (>200nm).<br>· High complexity in multidimensional data integration.	· Tumor immune exclusion zone mapping.<br>· Brain region-specific protein gradient atlases.<br>· Myocardial infarction spatial injury demarcation.

PTMs: post-translational modifications.

Table 2: Comparative Analysis of Five Mainstream Proteomics Techniques.

Technology	Introduction	Advantages	Disadvantages	Labeling Groups	Data Volume	Cost
**iTRAQ**	Employ chemical labels to identify proteins.	· Simultaneous analysis of up to 8 groups.<br>· Processing of multiple samples.<br>· Enhanced throughput.	· Expensive reagents.<br>· Complex experimental procedures.	4 or 8	Medium	High
**TMT**	Utilize chemical labeling to identify proteins.	· Simultaneous analysis of up to 10 or 11 groups.<br>· Simultaneous processing of more samples.<br>· Higher sensitivity.	· Expensive reagents.<br>· Complex experimental operations.	10 or 11	Medium	High
**SILAC**	Introduce isotope-labeled amino acids into the culture media.	· Accurate quantification.<br>· High sensitivity.	· Requirement for cell culture.<br>· Unsuitability for clinical samples.	2 or 3	Low	Medium
**Label-free**	Detect endogenous peptides without labeling.	· No need for labeling.<br>· Simple sample preparation.<br>· Cost-effective.	· Reduced reproducibility.<br>· Slightly diminished sensitivity.	Unlimited	High	Low
**DIA/SWATH**	Obtain mass spectrometry data through a full scan.	· No labeling required.<br>· Simultaneous quantification of numerous proteins.<br>· Good reproducibility.	· Complex data analysis.<br>· Need for specialized software.	Unlimited	High	Medium

iTRAQ: isobaric tags for relative and absolute quantification; TMT: tandem mass tag technology; SILAC: stable-isotope labeling by amino acids in cell culture; DIA: data-independent acquisition; SWATH: sequential window acquisition of all theoretical mass spectral approach.

Supplementary Table S1: Application of Multi-omics and High-spatial-resolution Omics Technologies in AD.

Disease	Omics Type	Purpose	Biomarker	Relevance to AD Pathogenesis	Experiment Validation	Reference
**AD**	Genomics	Diagnosis	*APOE4*	· *APOE4* accelerates vascular dysfunction, BBB rupture, and neuronal degeneration.<br>· *APOE4* is pivotal in AD's vascular and neurodegenerative pathogenesis and serves as a marker.	N	Montagne A et al.[61]
**AD**	Genomics	Diagnosis	*H4K16ac*	· Compared to non-AD elderly participants, 25,000 peaks showed *H4K16ac* loss, while 9,000 showed increased *H4K16ac* in AD individuals.<br>· *H4K16ac* decreases with aging or AD-related gene sites.<br>· *H4K16ac* set the stage for an epigenetic link between aging and AD.<br>· *H4K16ac* can be a diagnostic marker for AD disease.	N	Nativio R et al.[62]
**AD**	Genomics	Diagnosis	*H3K9ac*, TAU protein	· TAU, whereas non-amyloid β pathology has a broad impact on histone acetylation in AD brain.<br>· *H3K9ac* structural domain shows similar gain or loss of TAU-related histone acetylation.<br>· Complex interactions between TAU and chromatin structure.<br>· *H3K9ac* and TAU are biomarkers for AD.	Y (Three Independent Datasets)	Klein HU et al.[63]
**AD**	Transcriptomics	Diagnosis	*INPPL1, PLXNB1*	· The M109 module is the one most directly associated with cognitive decline and amyloid load.<br>· *INPPL1 and PLXNB1* are associated with extracellular β-amyloid levels in astrocyte cultures.<br>· *INPPL1 and PLXNB1* are interesting candidates for AD.	Y (Two Independent Datasets)	Mostafavi S et al.[64]

**AD**	Transcriptomics	Diagnosis	Myelination	· Neuroinflammation pathways are significantly upregulated in AD.<br>· Genes for myelination and lipid metabolism are downregulated in AD.<br>· Novel treatments for AD can focus on promoting myelin repair.	Y (Three Independent Datasets)	Shouneng Peng et al.[65]
**AD**	Proteomics	Treatment	STAT3, YES1 and FYN	· STAT3, YES1, and FYN reduce neuroinflammation, TAU phosphorylation, and endogenous production of amyloid-42.<br>· Drugs targeting the cytokine transducer STAT3 and the Src family tyrosine kinases, YES1 and FYN, rescued molecular phenotypes relevant to AD pathogenesis.<br>· STAT3, YES1, and FYN can be used as drug targets for the treatment.	Y (Three Independent Datasets)	Jackson A et al.[66]
**AD**	Proteomics	Treatment	Insulin signaling and mitochondrial electron transport chain	· Changes in hippocampal protein expression profiles in APP/PS1 and E4 knockout mice.<br>· Different expression proteins in both mouse models, participate in insulin signaling and the mitochondrial electron transport chain.<br>· Preserving mitochondrial function and boosting insulin signaling could aid in improving cognitive function for AD patients.	N	He K et al.[67]
**AD**	Proteomics	Diagnosis /Treatment	The phosphorylation levels of GSK3β and Ppp3ca, GSK3β, Ppp3ca	· The phosphorylation levels of GSK3β and Ppp3ca are closely associated with mitochondrial biogenesis.<br>· Low-dose oral copper treatment changes the phosphorylation of key hippocampal proteins involved in mitochondrial, synaptic and axonal integrity.<br>· The phosphorylation levels of GSK3β and Ppp3ca are potential diagnostic and therapeutic targets.	N	Chen C et al.[68]

**AD** (autosomal dominant)	Proteomics	Diagnosis	GFAP, NPTX2, PEA15, SMOC1, SMOC2, TNFRSF1B	· Six-protein prediction model (GFAP, NPTX2, PEA15, SMOC1, SMOC2, TNFRSF1B) with excellent predictive performance (AUC>0.9)<br>· Six early biomarkers far exceed the warning time window of traditional markers	Y (Three Independent Datasets)	Shen Y et al.[69]
**AD**	Metabolomics	Diagnosis	Sphingolipids	· Identified sphingolipids map to AD-related pathways (TAU phosphorylation, amyloid metabolism, calcium homeostasis, acetylcholine biosynthesis, apoptosis AD is associated with dysregulation of transmethylation and polyamine pathways.<br>· Sphingolipids as early AD biomarkers.	Y (Independent Cohort)	Varma VR et al.[70]
**AD**	Metabolomics	Diagnosis	PKM2	· PKM is an important glycolytic enzyme associated with AD pathology.<br>· Induced neurons iNs from AD patients express cancer-associated PKM2.<br>· PKM2 promotes Warburg effect-like glycolytic reprogramming in old neurons.<br>· PKM2 specifically interacts with and enhances the transcription factors *STAT3* and *HIF1α*, promoting AD-induced neurons' neuronal fate loss.<br>· PKM2 is a potential diagnostic target for AD.	N	Traxler L et al.[71]
**AD**	ScRNA-seq	Diagnosis	Myelination-related gene (*LINGO1*)	· Myelination plays a pivotal role in the pathophysiology of AD.<br>· The myelination-related gene *LINGO1* is perturbed in neurons and glial cells in patients with AD.	Y (Two Independent Datasets	Mathys H et al.[73]

**AD**	SnRNA-seq	Diagnosis	*APOE*	· The transcription factor EB serves as a principal regulator of lysosomal function, modulating multiple disease-associated genes in specific astrocyte subpopulations in AD.<br>· The risk gene *APOE* exhibits upregulated expression in specific microglia and astrocytes in AD, correlating with the severity of TAU pathology.<br>· *APOE* as a risk gene for AD has been confirmed.	N	Grubman A et al.[74]
**AD**	Spatial transcriptomics	Diagnosis	*OLIG*	· Early plaque-proximal dysregulation of *OLIG*/myelination gene co-expression networks in AD.<br>· Multicellular 57-PIG networks emerge, enriched for complement activation, oxidative stress, lysosomal dysfunction, and neuroinflammatory pathways.<br>· *OLIG* can be used as a marker for future AD diagnosis to lay the foundation for AD diagnosis.	Y (Mouse Model)	Wei-Ting Chen et al.[75]
**AD**	Spatial transcriptomics	Diagnosis	*SPARC, CALB2, DIRAS2*, and *KRT17*	· 10× Visium + co-immunofluorescence of AD markers delineated gene expression architecture in human middle temporal gyrus.<br>· Cortex-specific layer-enriched DEGs, including novel candidates *SPARC, CALB2, DIRAS2,* and *KRT17*, exhibiting pronounced alterations.<br>· These genes demonstrate significant potential as diagnostic targets for AD.	Y (Independent cohort)	Shuo Chen et al.[76]

**AD**	ScRNA-seq, Spatial transcriptomics	Treatment	Inhibitor of *PTPRG* or *VIRMA*	· *PTPRG+* microglia subpopulation induces neuronal *VIRMA* via intercellular signaling.<br>· Neuronal *PTPRG* binding to *VIRMA* enhances RNA stability; upregulated *VIRMA* increases PRKN m6A, reduces its RNA stability, causing mitophagy-driven neuronal death and AD progression.<br>· *PTPRG/VIRMA* inhibitors show their impacts on mitochondrial function and neuronal survival, offering potential therapies for AD.	Y (Mouse Model)	Donghua Zou et al.[77]
**AD**	SnRNA-seq, Spatial transcriptomics	Treatment	Lipid	· Specific microglial and astrocytic subtypes linked to Aβ, TAU, and lipid pathways were identified, revealing new therapeutic targets for AD.<br>· Lipid-associated microglia drive Aβ-TAU interplay, while reactive astrocytes exacerbate TAU-mediated inflammation and cognitive decline.<br>· Altered glial communities and divergent aging trajectories directly promote AD through multicellular dysregulation, enabling early intervention strategies.	N	Green, G S et al.[78]
**AD**	Spatial transcriptomics, Epigenomes	Diagnosis	*SPI1/PU.1*	· *PU.1* contributes to inter-individual differences in microglial regulatory networks.<br>· Reduced *PU.1* binding disrupts chromatin stability, supporting its functional role in AD.<br>· *SPI1/PU.1* identified as key regulator of microglial gene expression and AD risk.	N	Kosoy, R et al.[79]

**AD**	Spatial transcriptomics, Epigenomes	Diagnosis	*SPI1*, *ELF2*, *RUNX1*	· AD risk loci are highly enriched in microglial enhancers and TF-binding sites (*SPI1*, *ELF2*, *RUNX1*).<br>· Glial cells show accessible regulatory changes in late AD, while neurons exhibit alterations in early stages.<br>· Suggests epigenomic erosion as a hallmark of late AD and a potential indicator of disease progression.	N	Xiong, X et al.[80]
**AD**	Spatial transcriptomics, Epigenomes	Diagnosis	SST^+ level, $Pvalb^+/Vip^+$ level	· An early stage characterized by slow pathological accumulation, inflammatory microglia, reactive astrocytes, loss of SST^+ inhibitory neurons, and remyelination by oligodendrocyte precursor cells.<br>· A late stage marked by exponential pathological increase and loss of both excitatory neurons and inhibitory ($Pvalb^+/Vip^+$) neuronal subtypes.	N	Gabitto, M I et al.[81]
**AD** (autosomal dominant)	SnRNA-seq, Transcriptomics	Diagnosis	*LRP1*, *FKBP1B*, *PSEN1*	· In autosomal dominant AD, autophagy and chaperone genes show marked upregulation, with spatially resolved transcriptomics confirming specific activation of chaperone-mediated autophagy.<br>· In autosomal dominant AD cases, astrocytic *LRP1* and *FKBP1B* upregulation alongside neuronal *PSEN1* downregulation may collectively represent an intrinsic protective mechanism.	Y (Independent Cohort)	Almeida, M C et al.[82]
**AD**	Proteomics, Transcriptomics	Diagnosis	*MAPK*/metabolic module, matrix body module	· AD-related modules include *MAPK* signaling/metabolism and matrixsome modules.<br>· Matrixsome module is affected by *APOE ε4* allele.<br>· *MAPK*/metabolism module links to cognitive decline rate.<br>· Disease modules are potential AD targets/biomarkers.	N	ECB et al.[84]

**AD**	Proteomics, Transcriptomics	Diagnosis	*FBP1, FBP2, RHOH, JPH2, ERAP2, SCLT1*, and *MBP*	· *FBP1, FBP2, RHOH, JPH2, ERAp2, and SCLT1* are upregulated in *APOE4* cases compared to average expression in the normal brain.<br>· *MBP* is one of the top candidate genes enhancing the relevance of myelination in AD.<br>· Biomarkers show consistent protein profiles in plasma and brain.	Y (Two Independent Datasets)	Madrid L et al.[85]
**AD**	Genomics, Transcriptomics, Proteomics, Metabolomics	Diagnosis	*ABCA1, CPT1A, Adiponectin* and *NGAL*	· Short-chain acylcarnitines/amino acids and medium/long-chain acylcarnitines are closely correlated with the severity of AD.<br>· Two genes (*ABCA1 and CPT1A*) and two proteins (Adiponectin and *NGAL*) participate in the regulation of acylcarnitines and amino acids in AD.<br>· *ABCA1, CPT1A*, Adiponectin, and *NGAL* may be AD diagnostic biomarkers.	Y (Two Independent Datasets)	Horgusluoglu E et al.[86]
**AD**	Proteomics, Transcriptomics	Diagnosis	*IVD, CYFIP1 and ADD2*	· Significantly higher *IVD* protein abundance in AD patients.<br>· *CYFIP1 and ADD2* are significantly downregulated in AD patients.<br>· *IVD, CYF0IP1, and ADD2* combine to diagnose AD.	N	San Segundo et al.[87]
**AD**	Genomics, Metabolomics	Diagnosis	*CSTD, CTSB, CTSD, and GM2A*	· *CSTD* has been validated as a marker in previous CSF and plasma samples.<br>· AD progression is clearly accompanied by increased fold changes in these lysosomal proteins.<br>· Lysosomal proteins *CTSB, CTSD, and GM2A* are significantly increased as markers in CSF samples from AD patients.	N	Wang H et al.[88]

**AD**	Genomics, Proteomics	Diagnosis	*PBXIP1*	· *PBXIP1*-encoded protein shows significant association with all three AD neuropathological features.<br>· *PBXIP1* is associated with AD through its role in astrocytes and hippocampal neurons and the mTOR pathway.<br>· *PBXIP1* is associated with neuropathology and cognitive function.	Y (Two Independent Datasets)	Jingyun Zhang et al.[89]
**AD**	Genomics, Transcriptomics, Proteomics	Diagnosis	*H3K27ac*	· Differentially acetylated peaks are enriched in disease-related biological pathways, including those associated with Aβ and TAU pathology progression.<br>· Highly significant enrichment of AD risk variants in the *H3K27ac* peak region of the inner olfactory cortex, including *CR1, GPR22, KMO, PIM3, PSEN1*, and *RGCC.*<br>· *H3K27ac* can serve as a diagnostic target for AD.	N	Marzi SJ et al.[90]
**AD**	Transcriptomics, Proteomics, Epigenomics	Diagnosis	*H3K27ac, H3K9ac*	· RNA-seq analysis reveals upregulation of histone acetyltransferases in *H3K27ac* and *H3K9ac*.<br>· Genome-wide increases in *H3K27ac* and *H3K9ac* exacerbate Aβ42-driven neurodegeneration.<br>· Proteomic screening singles out *H3K27ac* and *H3K9ac* as major AD-specific enrichments.<br>· *H3K27ac* and *H3K9ac* affect disease pathways through dysregulated transcription and chromatin gene feedback loops.	Y (Two Independent Datasets, Drosophila Model)	Raffaella Nativio et al.[91]

**AD**	Proteomics, Metabolomics, Lipidomics	Diagnosis	Protein 14-3-3 zeta/delta, clusterin, interleukin-15, and transgelin-2	· Enrichment pathway analysis reveals overexpression of hemostatic, immune response, and extracellular matrix signaling pathways associated with AD.<br>· Protein 14-3-3 zeta/delta, clusterin, interleukin-15, and transgelin-2 improve AD prediction.	N	Clark C et al.[92]
**AD**	Proteomics, Metabolomics, Lipidomics	Diagnosis	GABA synthesis, arginine biosynthesis, and alanine, aspartate, glutamate, and arginine pathways	· Gender-dependent effects are seen on the pathways of significant enrichment, including those of GABA synthesis, arginine biosynthesis, and alanine, aspartate, glutamate, and arginine metabolism.<br>· Lysophospholipid and amino acid metabolism are involved in the AD brain.	Y (Mouse Model)	Abigail Strefeler et al.[93]
**AD**	Genomics, Transcriptomics	Treatment	*TRPV1*	· *TRPV1* activation rescues memory deficits and neuronal loss in *APOE4* high-fat diet-fed mice.<br>· Neuronal loss increases in *APOE4* high-fat diet mice, rescued by *TRPV1* activation in the capsaicin group.<br>· *TRPV1* is a treatment option for AD disease.	Y (Mouse Model)	Chenfei Wang et al.[94]
**AD** (late-onset)	Genomics, Transcriptomics	Treatment	*ATP6V1A*	· *ATP6V1A* has been identified as a key regulator of the top neuronal subnetwork, which is the most dysregulated in late-onset AD.<br>· *ATP6V1A* can be used as a therapeutic target.<br>· NCH-51 ameliorates neuronal damage caused by *ATP6V1A* deficiency in a Drosophila model.	Y (Drosophila Model)	Wang M et al.[95]

AlzGPS: Alzheimer's disease genome-wide positioning systems platform; CSF: cerebrospinal fluid; PKM2: pyruvate kinase M2; *MBP*: Myeloid basic protein encoding gene.

Supplementary Table S2: Application of Multi-omics and High-spatial-resolution Omics Technologies in PD.

Disease	Omics Type	Purpose	Biomarker	Relevance to PD Pathogenesis	Experiment Validation	Reference
**PD**	Genomics	Diagnosis	*ZNF184, IL1R2, LRRK2, ITPKB,* and *PARK16*	· Alleles of *LRRK2* and *IL1R2* confer a higher risk of developing PD.<br>· The genotype models of *ZNF184, PARK16,* and *ITPKB* are significantly associated with PD.<br>· Most of these genes are involved in autophagy and lysosomal function-related pathways.	N	Gao T et al.[102]
**PD**	Genomics	Diagnosis	*HLA, LRRK2, MAPT, TRIM10,* and *SETD1A*	· *HLA, LRRK2, MAPT, TRIM10,* and *SETD1A* are high-risk genes associated with PD.<br>· Significantly associated loci linked to PD are found in the *HLA* and *MAPT* gene loci.	N	Witoelar A et al.[103]
**PD**	Transcriptomics	Diagnosis	*SSR1*	· *SSR1* is found to be upregulated in PD patients.<br>· *SSR1* expression is negatively correlated with dopaminergic neuron survival.<br>· The upregulation of *SSR1* expression in peripheral blood precedes the abnormal behavior of the animals.<br>· The *SSR1*-based RF classifier has an AUC value of 0.91 and can be used as a diagnostic marker.	N	Zhang W et al.[104]

**PD**	Proteomics	Diagnosis	OMD, CD44、VGF, PRL, MAN2B1, and LRRK2	· ML identifies that OMD, CD44, VGF, PRL, and MAN2B1 show significant changes in PD patients and are significantly correlated with PD clinical scores.<br>· The enhanced neuroinflammatory characteristics in LRRK2 gene carriers are strongly correlated with PD.<br>· OMD, CD44, VGF, PRL, MAN2B1, and LRRK2 can be used as biomarkers for PD.	N	Karayel, MM et al.[105]
**PD**	Metabolomics	Diagnosis	Lipid metabolism related to carnitine shuttle, sphingolipid metabolism, and arachidonic acid metabolism	· Alterations in lipid metabolism related to carnitine shuttle, sphingolipid metabolism, arachidonic acid metabolism, and fatty acid biosynthesis are detected.<br>· Carnitine shuttling is the most important pathway associated with unmedicated PD patients by sebum.	N	Sinclair E et al.[106]
**PD**	Metabolomics	Diagnosis	Short-chain fatty acids, butyric acid	· Low levels of short-chain fatty acids are significantly associated with cognitive decline in PD patients.<br>· Decreased butyric acid levels are associated with poorer posture and gait disorder scores.<br>· Short-chain fatty acids and butyric acid serve as a potential diagnostic target.	N	Tan AH et al.[107]
**PD**	Metabolomics	Diagnosis	Proline	· Energy and lipid metabolism are overexpressed in PD.<br>· 139 metabolites, including proline, have notable changes in carnitine shuttle, vitamin E metabolism, lipid-related, glycerol phospholipids, sphingolipids, and fatty acids pathways.<br>· Proline and 139 other metabolites are considered particularly predictive of PD status.	Y (Independent Cohort)	Pereira, P.A.B et al.[108]

**PD**	Metabolomics	Diagnosis	Phenylacetic acid, phenylacetylglutamine, histidine, uric acid, and imidazoleacetic acid	· 45 metabolic markers in PD patients show high diagnostic power in early stages (AUC=0.92).<br>· Metabolites like phenylacetic acid, phenylacetylglutamine, histidine, uric acid, and imidazoleacetic acid show upregulated urine levels in PD, linked to neuro disorders.	N	Cai Z et al.[109]
**PD**	Metabolomics	Diagnosis	BCAA metabolism, glycine derivatives, steroid hormone biosynthesis, tryptophan, and phenylalanine metabolism	· 18 differential metabolites in urine have been identified as biomarkers for PD.<br>· Differential metabolites alter metabolic pathways associated with BCAA metabolism, glycine derivatives, steroid hormone biosynthesis, tryptophan metabolism, and phenylalanine metabolism.	N	Cai Z et al.[110]
**PD**	SnRNA-seq	Diagnosis	*IL1B*, *GPNMB*, and *HSP90AA1*	· A neuron cluster characterized by *CADPS2* overexpression and low tyrosine hydroxylase levels is identified in PD.<br>· Astrocytes and microglia in PD show specific proliferation and gene dysregulation linked to unfolded protein response and cytokine signaling.<br>· Microglia show a pro-inflammatory state with high *IL1B*, *GPNMB*, and *HSP90AA1*, suggesting their diagnostic potential.	N	Semra Smajić et al.[111]
**PD**	ScRNA-seq	Treatment	*HSP90* inhibitors	· Neurons and glial cells in PD exhibit dysfunction, immune dysregulation, and impaired protein folding.<br>· Administration of *HSP90* inhibitors accelerates the degradation of inflammasomes, reducing inflammatory responses and alleviating neurodegeneration.	Y (Mouse Model)	Gabriel GE et al.[112]

**PD**	Single-cell genomics, Spatial transcriptomics	Diagnosis	*TP53, NR2F2*	· The AGTR1-marked SNpc ventral subtype is highly PD-susceptible, showing *TP53/NR2F2* target gene upregulation.<br>· *TP53/NR2F2*-regulated pathways are key to PD-related neuronal death.<br>· *TP53/NR2F2* target gene upregulation indicates diagnostic biomarker potential.	Y (Macaque Model)	Tushar Kamath et al.[113]
**PD**	ScRNA-seq, Proteomics	Diagnosis	*SYN2*	· Negative correlation between α-synuclein pathology and chaperone protein expression in excitatory neurons in PD, along with weakened neuron-astrocyte interaction and aggravated neuroinflammation.<br>· *SYN2* enrichment in PD brain regions suggests significant increase in synaptic signaling at both RNA and protein levels.<br>· *SYN2* as a potential diagnostic biomarker for PD	Y (Independent Cohort)	Biqing Zhu et al.[114]
**PD**	Proteomics, Transcriptomics	Diagnosis	*GPNMB, CD38,* and *DGKQ*	· *GPNMB* and *CD38* show significant causal effects in PD, with evidence from quantitative trait locus analysis and fine mapping.<br>· *GPNMB, CD38,* and *DGKQ* proteins are associated with PD risk.	Y (Three Independent Datasets)	Guxiaojing et al.[115]
**PD**	Three Proteomics	Diagnosis	DDC	· DDC, SUMF1, DPP7, ENPEP, WFDC2, and hundreds of proteins are upregulated in the CSF, blood, or urine of PD patients.<br>· DDC levels are linked to symptom severity in PD patients.<br>· DDC can serve as a target for accurate PD diagnosis.	Y (Seven Independent Datasets)	Rutledge J et al.[116]

**PD**	Transcriptomics, Metabolomics	Treatment	The relaxin signaling pathway, adhesion patch, and PI3K-Akt signaling pathway	· BHD reduces PD symptoms, impacting metabolic pathways, including the relaxin signaling pathway, adhesion patch, and *PI3K-Akt* signaling pathway.<br>· BHD promotes the survival of dopaminergic neurons in PD mice, leading to improved motor performance.	Y (Mouse Model)	Hujun et al.[117]
**PD**	Genomics, Metabolomics	Diagnosis/ Treatment	*CircSV2b*	· Detect 33 deregulated circular RNAs in the PD mouse model vs wild-type controls.<br>· *CircSV2b* overexpression via the ceRNA-Akt1 axis mitigates oxidative stress in PD.<br>· *CircSV2b* is a potential Parkinson's diagnostic and curative biomarker.	Y (Mouse Model)	Cheng Qc et al.[118]

BCAA: branched chain amino acid; SNpc: substantia nigra pars compacta; DDC: Dopamine decarboxylase; ML: machine learning; *LRRK2*: leucine-rich repeat kinase 2; SUMF1: sulfatase-modifying factor 1; DPP7: dipeptidyl peptidase 2/7; *SSR1*: signal sequence receptor subunit 1; ENPEP: glutamyl aminopeptidase; WFDC2: WAP four-disulfide core domain 2; BHD: Buyang Huanwu Decoction.

Supplementary Table S3: Application of Multi-omics and High-spatial-resolution Omics Technologies in Epilepsy.

Disease	Omics Type	Purpose	Biomarker	Relevance to Epilepsy Pathogenesis	Experiment Validation	Reference
**Epilepsy**	Transcriptomics	Diagnosis	*P38MAPK, JAK-STAT, PI3K*, and mTOR signal pathway	· The*P38MAPK, JAK-STAT,* and *PI3K* consistently exhibit high expression and along with stable regulation of mTOR signaling pathways in epilepsy patients.<br>· Differential genes engage in signal cascades, ECM remodeling, cell motility, apoptosis, and immune responses linked to seizures.	N	Oswaldo K Okamoto et al.[122]
**Epilepsy** (temporal lobe)	Transcriptomics	Diagnosis	*Tlr2, Lgals3, Serpine 1* and *Stat3* et al.	· Several hub genes identified in TLE, such as *Tlr2, Lgals3, Serpine1*, and *Stat3*, et al., positively correlate with seizure frequency.<br>· Activation and phagocytic activity of microglia/macrophages have changed during the epileptic occurrence process of TLE.<br>· *Tlr2, Lgals3, Serpine 1,* and *Stat3* can serve as markers for TLE.	N	QingLan Chen et al.[123]
**Epilepsy**	Transcriptomics	Diagnosis	*GABAergic*	· FBTCS+ patients exhibit more widespread bilateral cortical and subcortical morphological alterations compared to FBTCS− patients.<br>· Excitatory and inhibitory neurons are affected in FBTCS−, while only excitatory neurons are significantly altered in FBTCS+.<br>· Excitatory/inhibitory imbalance and *GABAergic* dysfunction may underlie FBTCS susceptibility.	N	Lin, Q et al.[124]

**Epilepsy**	Transcriptomics	Diagnosis	*NMDAR*	· Analyses identified upregulation of the *NMDAR* signaling pathway as a key mechanism underlying both the autism-like behaviors and the observed anti-epileptic phenotypes.<br>· Impaired *GABAergic* function and enhanced *NMDAR* activity disrupt excitatory-inhibitory balance, promoting autism-epilepsy comorbidity.<br>· Dysfunctional *GABAergic* signaling and elevated *NMDA* activity contribute to co-occurrence of autism and epilepsy.	Y (Mouse Model)	Fan, C et al.[125]
**Epilepsy** (temporal lobe)	Transcriptomics	Diagnosis	*RBFOX1*	· TLE shows synaptic network reorganization, reduced connectivity, lower clustering, longer pathways, mainly in temporolimbic and frontoparietal regions.<br>· Identified 183 downregulated synaptic genes: *RBFOX1* and other *GABAergic* genes are central.<br>· Coordinated downregulation of risk genes may drive synaptic dysfunction and epilepsy in TLE, suggesting new treatment targets.	Y (Two Independent Datasets)	Li, R et al.[126]
**Epilepsy**	Proteomics	Diagnosis	GFAP	· GFAP is consistently downregulated in brain tissue with high spike frequencies and exhibits a strong negative correlation with spike frequency.<br>· Reactive astrocytes, such as GFAP, protect the neocortex from epileptic discharges rather than induce them.<br>· Epilepsy severity is closely linked to decreased GFAP (astrocyte marker) levels.	N	Gal Keren-Aviram et al.[127]

**Epilepsy**	Proteomics	Diagnosis/ Treatment	ADPRC, LPAR3, calreticulin, UCH-L1, SNAP-25, and transgelin-3	· A total of 144 differentially expressed proteins, such as ADPRC, LPAR3, calreticulin, UCH-L1, SNAP-25, and transgelin-3, are identified in the epileptic hippocampal regions.<br>· Most differentially expressed proteins are associated with Ca2+ homeostasis.<br>· Inhibiting calcium influx alleviates seizures triggered by excessive brain Ca^{2+} rise in epilepsy.	N	Leila Sadeghi et al.[128]
**Epilepsy**	Proteomics	Diagnosis	Calcineurin	· Tutin induces epilepsy by activating calcium-modulating phosphatase and produces significant neurological damage.<br>· Calcineurin is a target of tutin, and that tutin activates Calcineurin, leading to seizures.	Y (Mouse Model)	Shi-Shan Yu et al.[129]
**Epilepsy**	Metabolomics	Diagnosis	N-acetyl glycoprotein, lactate, creatine, glycine, lipid, and citrate	· Serum N-acetyl glycoprotein, lactate, creatine, glycine, and lipid levels are elevated decreased levels of citrate in epileptic children, while the level of citrate is reduced.<br>· The aforementioned metabolic substances are potential diagnostic targets for epilepsy.	N	Łukasz Boguszewicz et al.[130]
**Epilepsy (mesial temporal lobe)**	Metabolomics	Diagnosis	GABA	· GABA is significantly increased in the epileptogenic zone of KA-MTLE mice.<br>· GABA is a specific biomarker of the epileptogenic zone in MTLE.	Y (Mouse Model)	Hamelin, S et al.[131]
**Epilepsy**	SnRNA-seq	Diagnosis	*Sst* and *Pvalb*	· Major transcriptomic alterations occur in principal neurons (*L5-6_Fezf2, L2-3_Cux2*) and *GABAergic* interneurons (*Sst, Pvalb*).<br>· Profound dysregulation in glutamate signaling, characterized by robust upregulation of glutamate receptor genes, notably within *Sst/Pvalb* subtypes.<br>· *Sst/Pvalb* interneurons represent potential diagnostic targets and are fundamental to early epileptogenesis.	N	Ulrich Pfisterer et al.[132]

**Epilepsy** (post-traumatic)	ScRNA-seq	Diagnosis	*XIST*	· Hereditary epilepsy shows higher oligodendrocyte/astrocyte counts, lower microglia/neuron counts vs PTE.<br>· *IL-17* signaling in microglia/astrocytes can be a PTE target/biomarker.<br>· *XIST*, upregulated in PTE, drives inflammation/fibrosis, useful for diagnosis and mechanism study.	N	Fang Wen et al.[133]
**Epilepsy** (temporal lobe)	ScRNA-seq, SnRNA-seq, Spatial transcriptomics	Diagnosis	*SPP1, Trem2, Tle4* and *Sipa1l3*	· The differentially up-regulated genes in TLE patients are predominantly expressed in glial cells, while the down-regulated genes are mainly expressed in neurons.<br>· *SPP1* and *Trem2* are up-regulated in glial cells, whereas *Tle4* and *Sipa1l3* are down-regulated in these cells.	N	Quanlei Liu et al.[134]
**Epilepsy**	Genomics, Transcriptomics	Diagnosis	*Sestrin 3*	· *Sestrin 3* is a key regulator in the pro-convulsant gene network in the hippocampus of human epilepsy.<br>· *Sestrin 3* positively regulates modules in macrophages, microglia, and neurons.<br>· *Sestrin 3* holds potential as a diagnostic means for epilepsy.	Y (Mouse Model)	Johnson, M.R. et al.[135]
**Epilepsy**	Proteomics, Transcriptomics	Diagnosis	*STAT3, ErbB,* and *MAPK8*	· The TGF-β pathway is associated with cardiac function in the hearts of epileptic animals.<br>· *STAT3, ErbB,* and *MAPK8* are key regulators of cardiac alterations in epilepsy that contribute to seizure-mediated cardiac damage.	N	Sharma, S et al.[136]

**Epilepsy**	Proteomics, Metabolomics	Diagnosis	GSTM1, ALDH2	· Within the somatosensory cortex module, GSTM1 is identified as a protein hub and elevated expression levels.<br>· In the thalamus module, ALDH2 is pinpointed as a protein hub.<br>· The metabolic pathway enriched by the differences is lysine degradation.<br>· GSTM1 and ALDH2 are identified as markers for seizure-related modules in epilepsy.	N	Harutyunyan, A et al.[137]
**Epilepsy**	Genomics, Metabolomics	Diagnosis	Lactate, creatine, phosphocreatine, and choline	· Lactate is significantly reduced, while creatine, phosphocreatine, and choline are significantly increased.<br>· Lactate is involved in G protein-coupled receptor signaling and angiogenic pathways, and shows upregulation of ubiquitination-related genes.	N	Wu, H.C. et al.[138]
**Epilepsy**	Proteomics, Transcriptomics	Treatment	*miR-10a-5p, miR-21a-5p* and *miR-142a-5p*	· *miR-10a-5p, miR-21a-5p,* and *miR-142a-5p* are identified as key transcripts.<br>· These microRNA transcripts are primarily associated with the TGF-β pathway signaling.<br>· The combination of anti-miR (*miR-10a-5p, miR-21a-5p, miR-142a-5p*) exhibits protective effects against acute and spontaneous seizures.	Y (Mouse Model)	Venø, M.T. et al.[139]

PTE: post-traumatic epilepsy; TLE: temporal lobe epilepsy; MTLE: mesial temporal lobe epilepsy; NMDA: N-methyl-D-aspartate; GABA: γ-aminobutyric acid; *GFAP*: glial fibrillary acidic protein; KA-MTLE: kainic acid into mesiotemporal lobe epilepsy mice; TGF-β, transforming growth factor β; ADPRC: ADP-ribosyl cyclase; FBTCS: focal to bilateral tonic–clonic seizures; LRRK2: leucine-rich repeat kinase 2; LPAR3: lysophosphatidic acid receptor 3; UCH-L1: ubiquitin carboxyl-terminal hydrolase L1; GSTM1: glutathione s-transferase M1; SNAP-25: synaptosome-associated protein 25.

Supplementary Table S4: Application of Multi-omics and High-spatial-resolution Omics Technologies in MS.

Disease	Omics Type	Purpose	Biomarker	Relevance to MS Pathogenesis	Experiment Validation	Reference
**MS**	Genomics	Diagnosis	*STAT3, IL7*	· Inhibitory neurons in the CNS are key contributors to MS susceptibility.<br>· Genes such as *STAT3* and *IL7* disrupt immune pathways specifically in inhibitory neurons.<br>· Targeted interventions for CNS pathways—such as neuronal and glial functions, including *STAT3* and *IL7*.	Y (Three Independent Datasets)	Philip De Jager et al.[146]
**MS**	Proteomics	Diagnosis	CXCL13, LTA, FCN2, ICAM3, LY9, SLAMF7, TYMP, CHI3L1, FYB1, TNFRSF1B, and NFL	· Lower levels of *NFL* in CSF show predictive potential for disease activity (AUC=0.77).<br>· An 11-protein panel in CSF has a high AUC for prediction, including CXCL13, LTA, FCN2, ICAM3, LY9, SLAMF7, TYMP, CHI3L1, FYB1, TNFRSF1B, and NFL (AUC=0.9).<br>· All the above proteins can be markers for MS.	N	Mika Gustafsson et al.[147]
**MS**	Metabolomics	Diagnosis	DRD2	· DRD2 exacerbates the disease by promoting inflammation and reducing the abundance of Lactobacillus species in the microbiome.<br>· Lactobacillus-derived N2-acetyl-L-lysine inhibits microglial activation, combating neurodegeneration.<br>· Intestinal epithelial DRD2, serving as a biomarker, can modulate the gut microbiome in MS.	N	Hairong Peng et al.[148]

**MS** (relapsing-remitting)	Metabolomics	Diagnosis/ Treatment	Glycolysis	· Identified four perturbed metabolic pathways, including structural/signaling lipids and energy, in the serum of patients with MS.<br>· Glycolysis is the common upstream feeding of these altered metabolic pathways.<br>· Targeting glycolysis in experimental autoimmune encephalomyelitis ameliorated the disease pathology by impeding immune cell effector function.	Y (Mouse Model)	Insha Zahoor et al.[149]
**MS**	ScRNA-seq	Diagnosis	*TFH*	· Myeloid dendritic cells and regulatory T cells are enriched in the CSF of patients with MS.<br>· The independent increase in clusters of TFH cells drives the known expansion of B-lineage cells in the CSF in MS.<br>· TFH cells promote the infiltration of B cells into the central nervous system, exacerbating MS disease.	Y (Mouse Model)	David Schafflick et al.[150]
**MS**	ScRNA-seq, Spatial transcriptomics	Diagnosis	*SERPINA3*	· Astrocytes can be classified into three types: homeostatic, intermediate, and disease-associated types.<br>· In patients with DA-Astro, the expression level of *SERPINA3* is significantly elevated.<br>· *SERPINA3* expression may constitute a glial cell survival response to resolve inflammation and prevent apoptosis during both initial and late resolution phases.	N	Petra Kukanja et al.[151]

**MS**	ScRNA-seq, Spatial transcriptomics	Diagnosis	*MAFB*	· The expression of pro-inflammatory molecules in oligodendrocytes near axonal damage is elevated in MS patients.<br>· *MAFB* mediates intercellular communication via complement factors and apolipoproteins.<br>· The inflammatory transcription factor *MAFB* serves as a biomarker for MS lesions.	N	Maria L Elkjaer et al.[152]
**MS (systemic)**	ScRNA-seq, Spatial transcriptomics, Spatial proteomics	Diagnosis/ Treatment	*POSTN/SCARA5*, *CXCR4*	· A dynamic spatial interaction network is established between fibroblasts and macrophages via the *ACKR3-CXCL12-CXCR4* signaling axis, playing a central role in driving fibrosis progression.<br>· Treatment with the *CXCR4* inhibitor AMD3100 significantly alleviates fibrosis in skin and lung tissues.<br>· The significantly elevated *POSTN/SCARA5* ratio in MS can serve as a predictive diagnostic biomarker.	Y (Mouse Model)	Zhijian Li et al.[153]
**MS**	Proteomics, Transcriptomics	Diagnosis	*GPR37L1, SIRPA, FGFR3, CADM3*, and *TYRO3*	· Neurological candidate molecules, including *GPR37L1, SIRPA, FGFR3, CADM3*, and TYRO3, are highly expressed in the CNS of MS.<br>· These genes are associated with early neuronal degeneration and dysfunctional trophic/anti-inflammatory intercellular communication.<br>· *GPR37L1, SIRPA, FGFR3, CADM3, and TYRO3* can be used as a diagnostic method for MS.	N	Max Kaufmann et al.[154]

**MS**	Proteomics, Transcriptomics	Diagnosis	24 iron death-related genes (*CHMP5, SLC38A1, PML*, etc.)	· High iron death scores at the margins of active lesions correlate with phagocytic activation.<br>· Elevated iron death scores in cortical neurons are associated with neurological diseases.<br>· A blood-based model of 24 iron death-related genes is a prognostic marker for diagnosing MS, including *CHMP5, SLC38A1, PML*, etc.	N	Tao Wu et al[155]
**MS**	Proteomics, Metabolomics	Diagnosis	LAMP1, FCG2A, and HPSE	· *HPSE* is positively correlated with many MS-related metabolites, including L-tyrosine, sphingosine 1-phosphate, sphingosine 1-phosphate, and L-tryptophan.<br>· The proteins LAMP1, FCG2A, and HPSE exhibit potential utility as specific biomarkers for MS.	N	Fan Yang et al.[156]
**MS**	Proteomics, Metabolomics	Diagnosis	Equine uric acid, sphingolipids	· Anti-inflammatory molecules and sphingolipids are reduced by metabolomics in MS patients.<br>· Low levels of equine uric acid in a severe subgroup of MS.<br>· Sphingolipids and equine uric acid facilitate the future development of biomarkers and targeted therapeutic interventions for MS.	N	Qinming Zhou et al.[157]

NFL: neurofilament light chain; DRD2: Dopamine Receptor D2; CNS: central nervous system; HPSE: heparinase; TFH: T follicular helper.

Supplementary Table S5: Application of Multi-omics and High-spatial-resolution Omics Technologies in Stroke.

Disease	Omics Type	Purpose	Biomarker	Relevance to Stroke Pathogenesis	Experiment Validation	Reference
**Stroke** (ischemic)	Proteomics	Diagnosis	NSF, RhoGDI1, and RabGDI	· Circulating NSF, RhoGDI1, and RabGDI are upregulated in patients with IS.<br>· These proteins trigger neuronal depolarization and calcium surge, activating death pathways in stroke.	Y (Independent Cohort)	Eloy Cuadrado et al.[160]
**Stroke** (ischemic)	Proteomics	Diagnosis	CMPK, CKB	· Circulating levels of CKB and CMPK are higher in patients with ischemic stroke than in controls during the acute phase.<br>· CKB plays a crucial role in energy transduction and homeostasis.<br>· CMPK is released in large amounts and participates in mechanisms that counteract cell disruption and neuronal cell death.	N	Alba Simats et al.[161]
**Stroke** (ischemic)	Proteomics	Diagnosis	SAHH2	· SAHH2 plays a significant role in the coordinated inhibition of $Ca^{2}+$ ion transporters.<br>· Increased expression of SAHH2 in neurons from the infarcted area is probably because of ischemia-triggered Ca2+ mobilization.	N	Teresa García-Berrocoso et al.[162]
**Stroke**	Transcriptomics	Diagnosis	LncRNA (*MEG3, H19,* and *MALAT1*)	· LncRNAs, such as *MEG3, H19,* and *MALAT1*, in blood cells between patients with stroke and healthy controls show differences.<br>· Differential genes modulate neuronal survival/apoptosis targets, impacting p53-mediated apoptosis in stroke.	N	Cheryl Dykstra Aiello et al.[163-167]

**Stroke**	Transcriptomics	Diagnosis	Extracellular microRNA	· Decreased levels of extracellular *miR-32-3p, miR-106b-5p, miR-423-5p, miR-451a, miR-1246, miR-1299, miR-3149* and *miR-4739*, and increased levels of extracellular *miR-224-3p, miR-377-5p, miR-518b, miR-532-5p* and *miR-1913* associate with stroke.<br>· These genes affect multiple pathways such as apoptosis, oxidation, angiogenesis, and neurogenesis in IS.	N	Ceren Eyileten et al.[168]
**Stroke**	Transcriptomics	Diagnosis	*IFN-I*	· Aged brains show marked upregulation of *IFN-I* signaling following ischemic injury.<br>· Aging leads to downregulation of genetic programs essential for axonal and synaptic integrity after stroke.<br>· *IFN-I* may serve as a diagnostic biomarker for stroke.	N	Androvic, P et al.[169]
**Stroke** (cardioembolic)	Metabolomics	Diagnosis	Valine, Leucine, Isoleucine	· The expression levels of BCAA, including valine, leucine, and isoleucine, are decreased in patients with cardioembolic stroke.<br>· Lower BCAA levels are also associated with poor neurological outcomes.	N	W Taylor Kimberly et al.[170]
**Stroke**	Metabolomics	Diagnosis	Total free fatty acid	· Plasma concentration of total free fatty acids is higher in patients with cardioembolic stroke than in patients with non-cardioembolic stroke.<br>· Elevated free fatty acid levels are significantly associated with cardioembolic stroke, suggesting their potential as a diagnostic target.	N	Jeong Yoon Choi et al.[171]

**Stroke**	ScRNA-seq	Treatment	Microglia and macrophages	· Aging jeopardizes the repair and regeneration of the cerebrovascular system and proteins after stroke.<br>· After stroke, microglia and macrophages may affect angiogenesis and oligodendrogenesis via paracrine mechanisms, impeding stroke recovery.<br>· Transplanting microglia and macrophages from the brains of young mice into the cerebral cortex of aged stroke-affected mice partially restores angiogenesis and oligodendrogenesis.<br>· Microglia and macrophages serve as effective targets for promoting stroke recovery	N	Chenghao Jin et al.[172]
**Stroke** (ischemic)	ScRNA-seq, Spatial transcriptomics	Treatment	*LILRB4*	· Stroke brains have up-regulated *LILRB4* and ischemia-linked microglial cluster 3.<br>· *LILRB4* knockout worsens ischemic brain injury via CD8+ T cell recruitment; overexpression offers neuroprotection.<br>· Targeting *LILRB4* and its downstream pathways represents an effective therapeutic strategy for ischemic stroke.	N	Yilin Ma et al.[173]
**Stroke** (brainstem)	SnRNA-seq, ScRNA-seq	Treatment	Myo1e	· Oligodendrocyte loss leads to neurological deficits following brainstem stroke.<br>· *OLG8* has an innate neuroprotective effect in brainstem stroke.<br>· Myo1e aids *OLG8* migration to the peri-infarct area in brainstem stroke.<br>· Myo1e overexpression in *OLG8* oligodendrocytes boosts brainstem stroke recovery.	N	Shaojun Li et al.[174]

**Stroke** (intracerebral hemorrhage)	ScRNA-seq, Spatial transcriptomics	Diagnosis	*SPP1*, *Lyz2*	· *SPP1/Lyz2* show high expression levels, and lymphocytes with high expression interact with myeloid cells in the late stage of stroke.<br>· During the acute phase of intracerebral hemorrhage, Lgmn+Macro-T cells and microglia interact via the *SPP1-cd44* pathway.<br>· *SPP1* and *Lyz2* are potential diagnostic targets for the acute phase of intracerebral hemorrhage.	N	Lingui Gu et al.[175]
**Stroke**	ScRNA-seq, Spatial transcriptomics	Treatment	Lipocalin-2	· Ferroptosis is the primary programmed cell death process post-hemorrhagic stroke, mainly affecting mature oligodendrocytes.<br>· A specific interaction between lipocalin-2-positive microglia and oligodendrocytes, mediated by the CSF1 receptor pathway, induces ferroptosis in oligodendrocytes and subsequent neurological deficits.<br>· Early therapeutic intervention by inhibiting *LCN2* expression may alleviate ferroptosis-induced oligodendrocyte damage and related neurological deficits.	N	Lingui Gu et al[176]
**Stroke**	ScRNA-seq, Spatial transcriptomics	Treatment	*LGALS9*	· Galectin (*LGAL*) signaling is enhanced in microglia and macrophages of ischemic mice.<br>· *LGALS9* treatment promotes oligodendrocyte remyelination and improves stroke recovery in mice.<br>· *LGALS9* can serve as a therapeutic approach to ameliorate stroke.	N	Bing Han et al.[177]

**Stroke (subarachnoid hemorrhage)**	ScRNA-seq, Spatial transcriptomics	Diagnosis	*THBS1, S100A6*	· *THBS1* and *S100A6* are closely associated with the prognosis of SAH, with their expression significantly increasing following the hemorrhage.<br>· The *THBS1-CD47* pair regulates cell apoptosis, and blocking their interaction may represent a new therapeutic approach for SAH.<br>· *THBS1* and *S100A6* serve as diagnostic biomarkers for stroke.	N	Xiaoyu Wang et al.[178]
**Stroke**	Genomics, Spatial transcriptomics	Diagnosis	*MMP-9*	· Plaque rupture occurs predominantly in proximal and most stenotic areas.<br>· Identified *MMP-9* as a key gene causally linked to rupture risk.<br>· Supports targeted intervention against *MMP-9* for precise stroke treatment.	N	Sun, J et al.[179]
**Stroke**	ScRNA-seq, Spatial transcriptomics	Diagnosis	*APOE, FABP5*	· Distinct astrocyte states were identified post-stroke, influenced by both time and proximity to the ischemic lesion.<br>· Proximal astrocytes exhibited functional divergence in lipid transport, characterized by elevated expression of *APOE* and *FABP5* after cortical ischemic stroke.<br>· *APOE* and *FABP5* hold promise as diagnostic biomarkers for stroke.	N	Scott, EY et al.[180]
**Stroke** (cardioembolic)	Proteomics, Transcriptomics	Diagnosis	*ICA1L, CAND2*, and *ALDH2*	· Reduced *ICA1L, CAND2,* and *ALDH2* may impair excitatory synaptic signaling, contributing to cardioembolic stroke pathogenesis.<br>· *ICA1L, CAND2,* and *ALDH2* are potential biomarkers for lacunar stroke.	Y (Three Independent Datasets)	Zhang C et al.[181]

**Stroke** (ischemic)	Genomics, Metabolomics	Treatment	Gut flora and metabolic disturbances	· ZHTC modulates the abundance of specific bacterial groups and 23 metabolic differences for IS, including arginine, L-lysine, and L-methionine.<br>· ZHTC improves intestinal barrier integrity by increasing the expression levels of tight junction proteins<br>· ZHTC meliorates IS by modulating gut flora and metabolic disturbances.	Y (Rat Model)	Wang R et al.[182]
**Stroke** (ischemic)	Proteomics, Transcriptomics	Treatment	*PI3K-Akt, MAPK*, and cAMP signaling pathways	· *YQTL* reduces infarct volume percentage and improves neurological function in cerebral ischemia-reperfusion injury mice.<br>· Network pharmacology and multi-omics studies reveal 15 components that regulate 82 targets and 19 pathways.<br>· *YQTL* protects against cerebral ischemia-reperfusion injury through *PI3K-Akt, MAPK*, and cAMP signaling pathways.	Y (Mouse Model)	Yuan Y et al.[183]

lncRNAs: long non-coding RNAs; IS: Ischemic Stroke; BCAA: branched-chain amino acid; *LGAL*: Galectin; *CSF1*: colony-stimulating factor 1; *SAH*: subarachnoid hemorrhage; ZHTC: Zhilong Huoxue Tongyu capsule; YQTL: Yiqi Tongluo granule; *ALDH2*: aldehyde dehydrogenase 2.

Supplementary Table S6: Application of Multi-omics and High-spatial-resolution Omics Technologies in Hydrocephalus.

Disease	Omics Type	Purpose	Biomarker	Relevance to Hydrocephalus Pathogenesis	Experiment Validation	Reference
**Hydrocephalus** (communicating)	Genomics	Diagnosis	*TRIM71*, *SMARCC1*, *PIK3CA*, *PTEN*, *MTOR*, *FOXJ1*, *FMN2*, *PTCH1*, and *FXYD2*	· *TRIM71* and *SMARCC1* exhibit genome-wide significant enrichment of de novo mutations, which may be genuine risk factors for CH.<br>· *PIK3CA*, *PTEN*, *MTOR*, *FOXJ1*, *FMN2*, *PTCH1*, and *FXYD2* are newly identified high-confidence sporadic CH genes.<br>· *TRIM71* and other genes, reducing neural cell proliferation to cause hydrocephalus, can be a diagnostic marker.	N	Sheng Chih Jin et al.[191]
**Hydrocephalus** (communicating)	Proteomics	Diagnosis	KLK6	· Expression of KLK6 is significantly up-regulated in CH patients.<br>· KLK6 is involved in CH development and may provide a new target for CH diagnosis.	Y (Rat Model)	Lei Yuan et al.[192]
**Hydrocephalus** (idiopathic normal pressure)	Proteomics	Diagnosis	QPCT, RBP4	· 39 proteins exhibit a significant increase, while 285 proteins show a significant decrease in CSF of iNPH.<br>· Elevated proteins mainly relate to myeloid leukocyte migration and extracellular matrix organization; reduced ones are linked to axon and synaptic development.<br>· QPCT and RBP4 have been identified as potential protein biomarkers in iNPH for predicting shunt outcomes.	N	Yuqi Ying et al.[193]

**Hydrocephalus** (idiopathic normal pressure)	Proteomics	Diagnosis	PTPRQ	· PTPRQ concentrations in CSF are significantly higher in iNPH patients than in AD patients.<br>· PTPRQ concentration in the CSF of non-responders to shunt operation tended to be relatively lower compared with that in the responders.<br>· PTPRQ is a candidate biomarker to distinguish iNPH from AD.	N	Yuki Nagata et al.[194]
**Hydrocephalus** (idiopathic normal pressure**)**	Metabolomics	Diagnosis	Glyceric acid, N-acetyl neuraminic acid, serine, and 2-hydroxybutyric acid	· Elevated glyceric acid and N-acetyl neuraminic acid, and reduced serine and 2-hydroxybutyric acid in AD CSF distinguish it from iNPH.<br>· Serine, glyceric acid, Neu5Ac, and 2-hydroxybutyrate combine as a diagnostic iNPH biomarker.	N	Yuki Nagata et al.[195]
**Hydrocephalus** (normal pressure)	Metabolomics	Treatment	Neu5Ac	· CSF Neu5Ac levels are low in NPH patients.<br>· Boosting brain Neu5Ac inhibits astrocyte activation.<br>· Brain Neu5Ac elevation reduces periventricular demyelination and improves hydrocephalus.<br>· Enhanced brain Neu5Ac improves neurological outcomes in NPH, suggesting a potential treatment.	Y (Mouse Model)	Zhangyang Wang et al.[196]
**Hydrocephalus** (tumor-associated)	SnRNA-seq, Spatial transcriptomics	Diagnosis/Treatment	*CPMCs*	· Ventricular cell atlas reveals *CPMC* expansion in TAH mice.<br>· *CPMCs* compromise ependymal ciliary integrity via tryptase-PAR2-FOXJ1 signaling, triggering pathological CSF hypersecretion that underlies hydrocephalus pathogenesis.<br>· Brain barrier-penetrating trypsin-like inhibitor BMS-262084 effectively inhibits TAH progression in vivo and attenuates mast cell-induced epithelial cilia damage.	N	Yiye Li et al.[197]

**Hydrocephalus**	Genomics, scRNA-seq	Diagnosis	*MAEL*	· scRNA-seq data from the cortical plate and germinal matrix revealed robust *MAEL* expression within neurogenic niches.<br>· Reduced *MAEL* levels may induce genomic structural alterations, thereby impairing cortical development, volume, and function.<br>· Decreased *MAEL* expression is associated with the pathogenesis of hydrocephalus.	Y (Independent Cohort)	Hale, AT et al.[198]
**Hydrocephalus** (communicating)	Genomics, Proteomics, Transcriptomics	Diagnosis	*MAEL*	· PrediXcan analysis in 10 neuro tissues and whole blood shows a correlation between reduced *MAEL* gene expression in the brain and hydrocephalus ($p < 0.05$).<br>· Reduced *MAEL* expression increases susceptibility to hydrocephalus.<br>· *MAEL* is a diagnostic biomarker for hydrocephalus.	Y (Mouse Model)	Andrew T Hale et al.[199]
**Hydrocephalus** (post-hemorrhagic)	Proteomics, Metabolomics	Diagnosis/Treatment	CSPG4	· CSPG4 positively correlates with ventricular size and the incidence of periventricular leukomalacia.<br>· Silencing of CSPG4 can inhibit ferroptosis, cell adhesion functions, and intracellular Ca2+ flux.<br>· CSPG4 has been identified as a CSF biomarker and effective therapeutic target.	Y (Mouse Model)	Juncao Chen et al.[200]

NPH: normal pressure hydrocephalus; iNPH: idiopathic normal pressure hydrocephalus; CH: communicating hydrocephalus; *Neu5Ac*: N-acetylneuraminic acid; TAH: tumor-associated hydrocephalus; *CPMCs*: choroid plexus mast cells; MAEL: maelstrom spermatogenic transposon silencer; KLK6, kallikrein-6; *QPCT*: glutaminyl-peptide cyclotransferase; RBP4: retinol-binding protein 4; PTPRQ: Q-type protein tyrosine phosphatase receptor; CSPG4: chondroitin sulfate proteoglycan 4.

Figure1

Click here to access/download;Figure;Figure1-Metabolomics_type.pdf

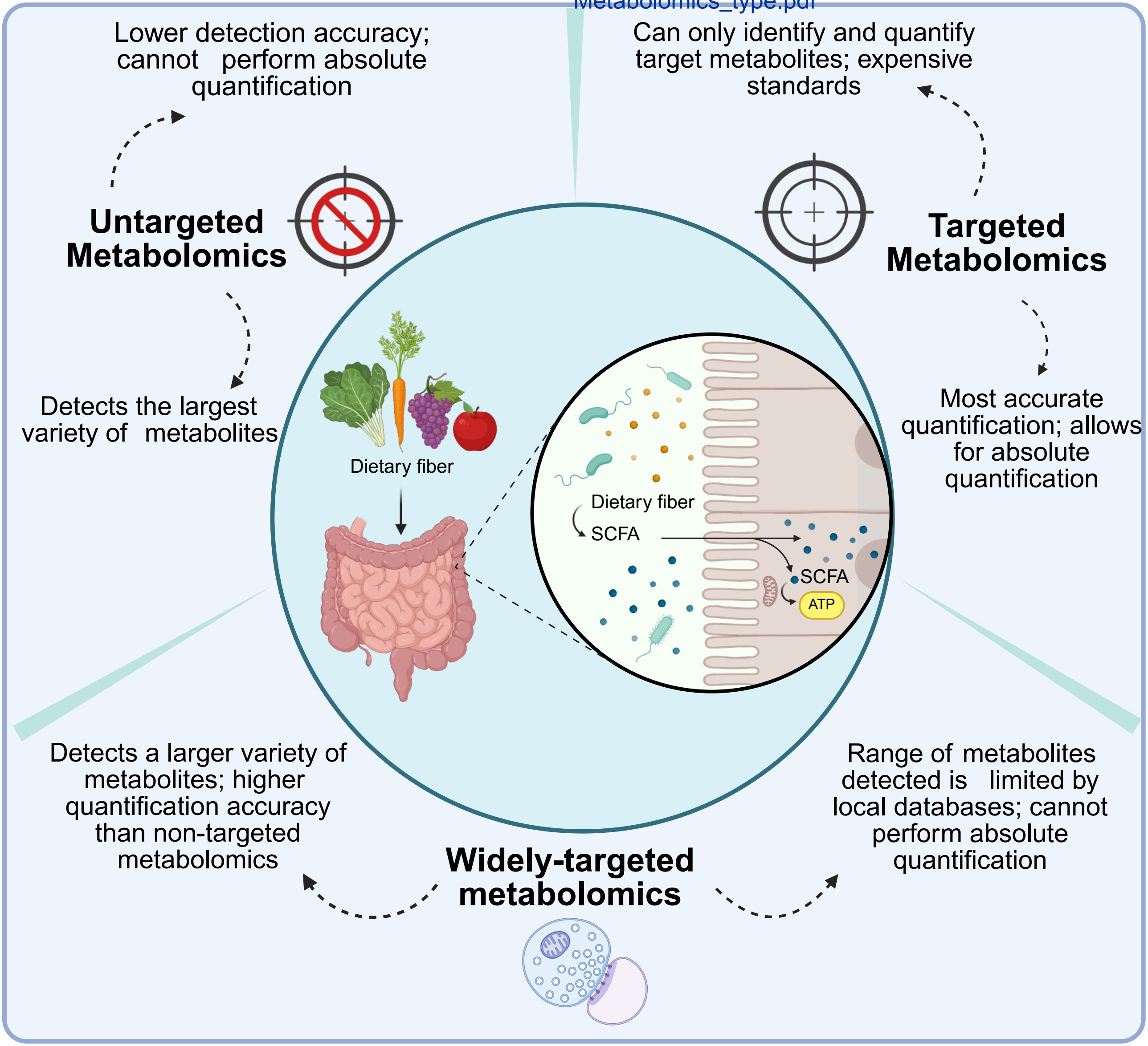

Figure2

Click here to access/download;Figure;Figure2-High-resolution_omics.pdf

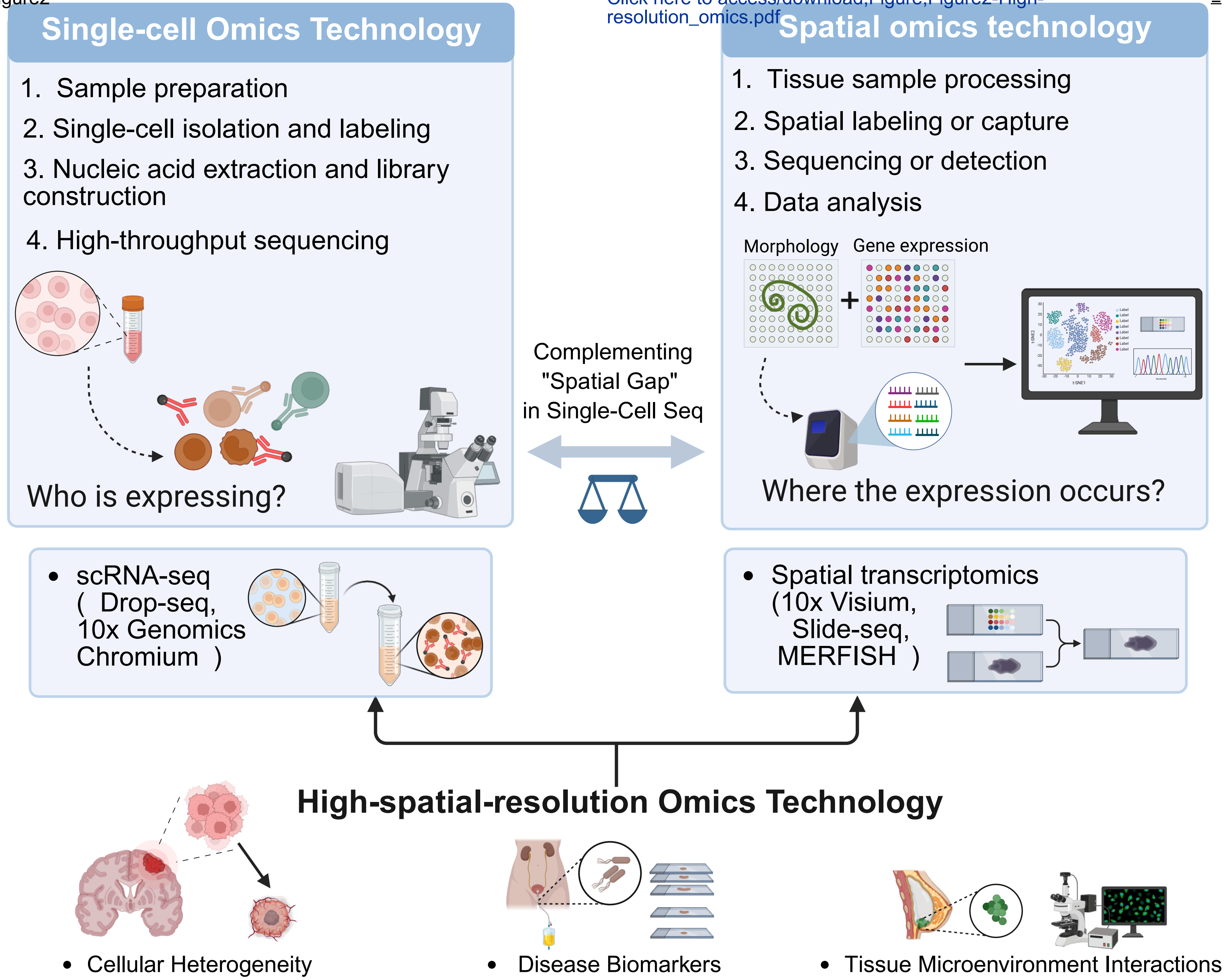

Figure3

Click here to access/download;Figure;Figure3-Muti_omics_in_nervous_system_diseases.pdf

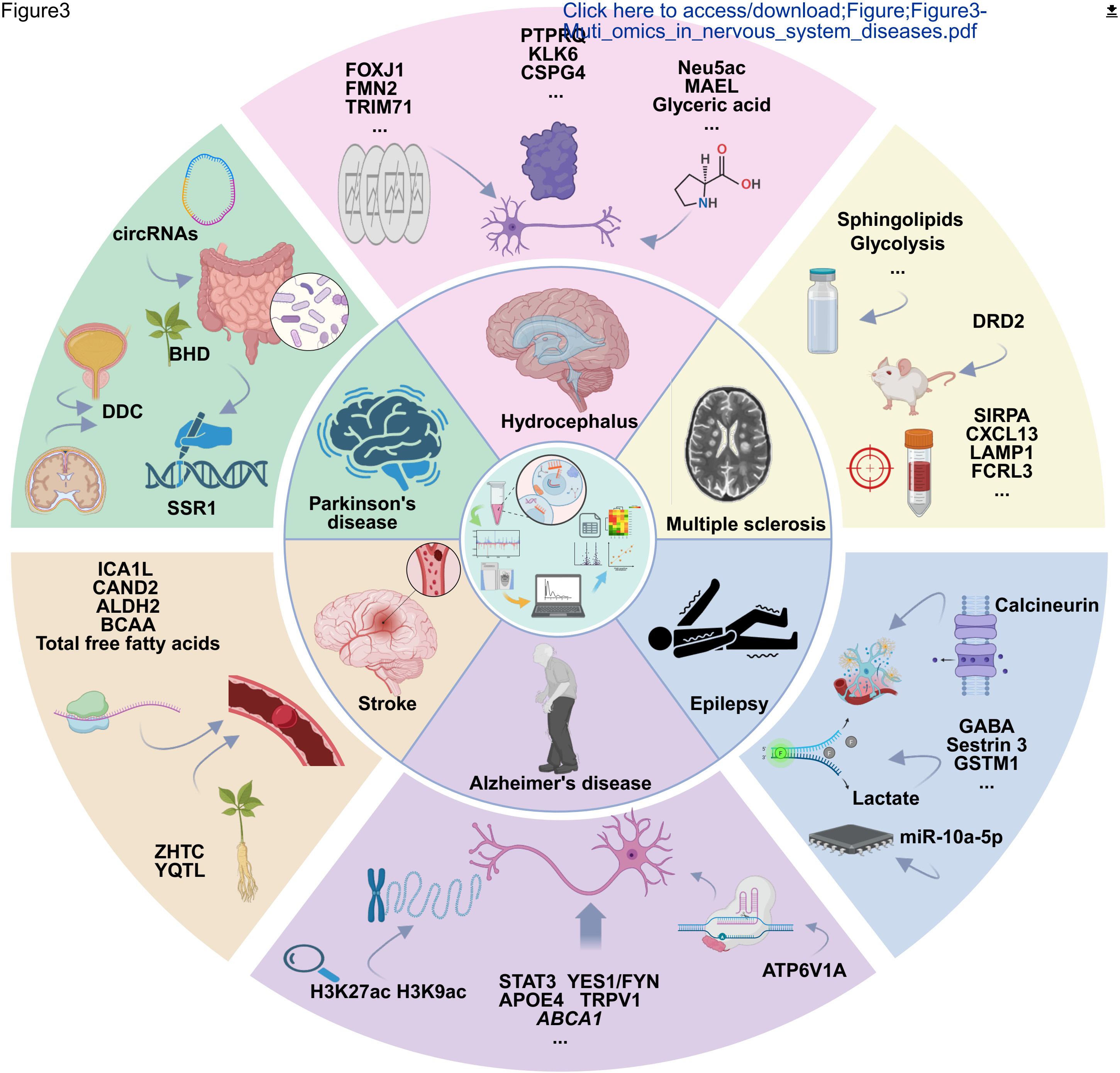

Click here to access/download;Figure;Figure4-AD_mechanism.pdf

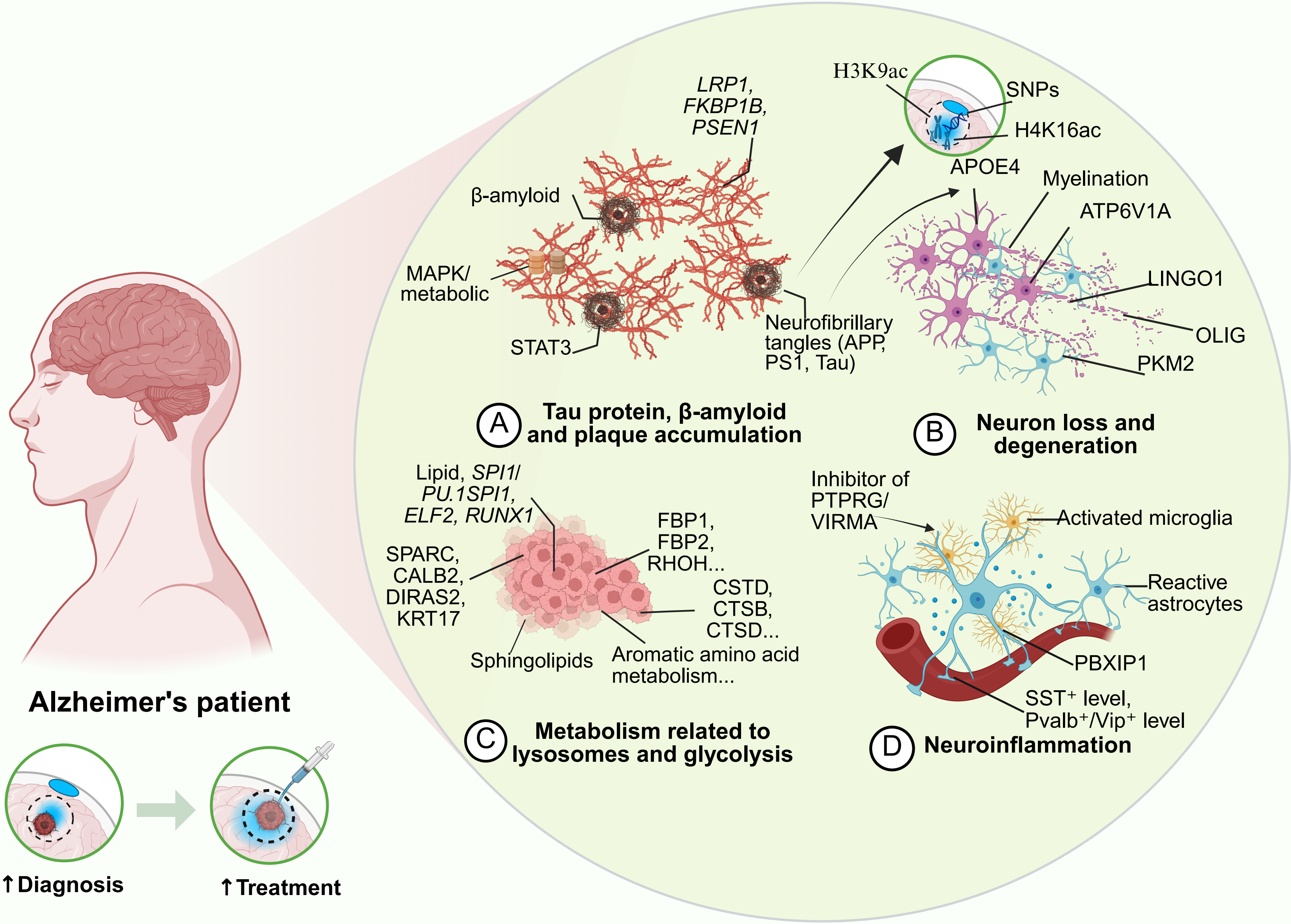

Click here to access/download;Figure;Figure5-PD_mechanism.pdf

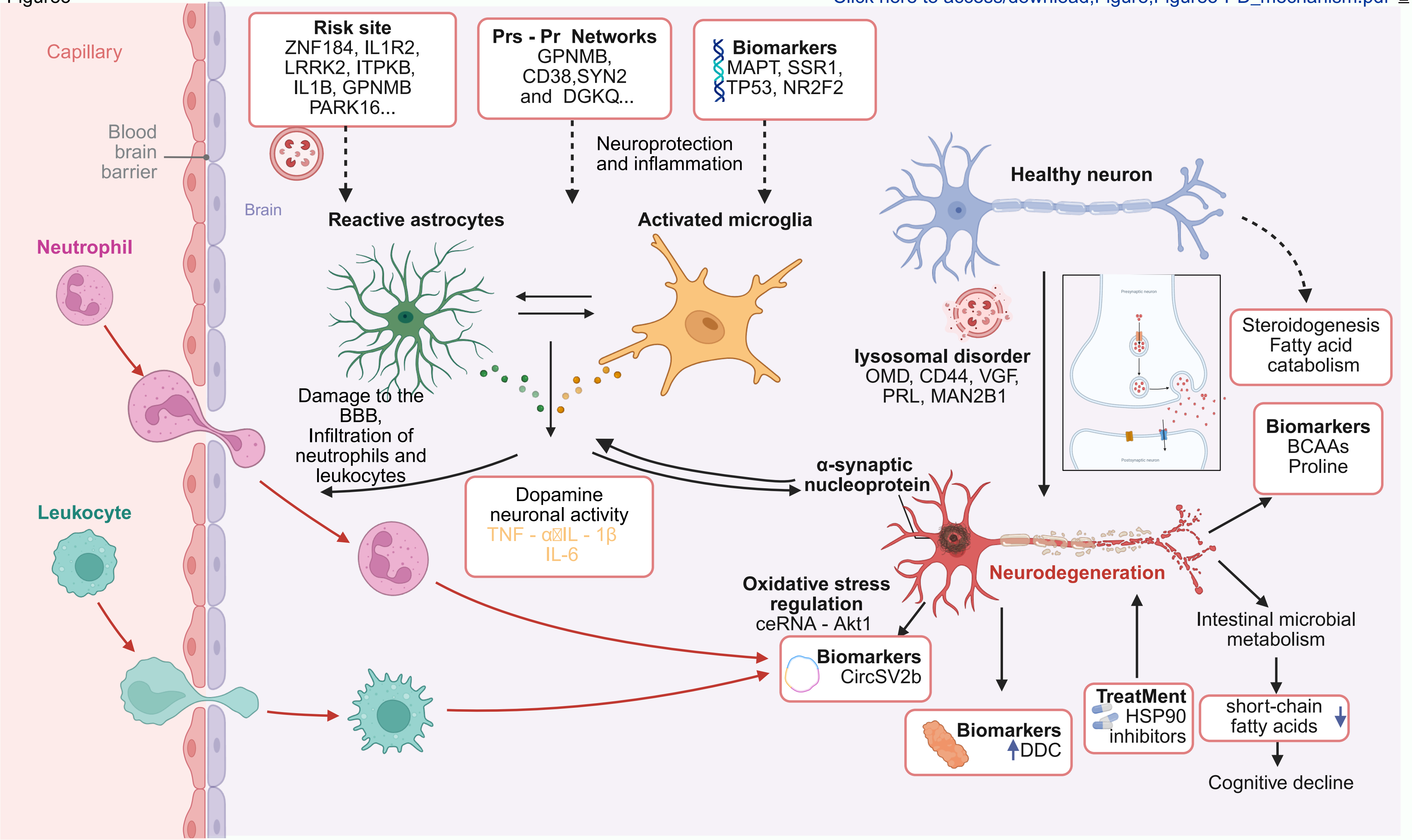

Personal Cover Click here to access/download;Personal Cover;Cover_Letter.docx

Xiuyun Liu, Professor
Dean of School of Pharmaceutical Sciences and Technology
Tianjin University
Tianjin, China, 300072
Email: xiuyun_liu@tju.edu.cn
Tel: +86 13820594672

August 30th, 2025

Dear Editor-in-Chief,

We wish to submit an original review article entitled "Multi-Omics and High-Spatial-Resolution Omics: Deciphering Complexity in Neurological Disorders" for consideration in GigaScience.

Neurological diseases, which have become an increasingly significant global health challenge, are characterized by structural and functional abnormalities in the central and peripheral nervous systems, triggered by multiple factors such as genetic mutations, metabolic disturbances, and immune dysfunctions, and exhibit highly complex pathogenesis involving disruptions at various molecular levels, ranging from genetic variations and transcriptional alterations to protein dysfunctions and metabolic imbalances. The advent of high-throughput technologies, such as next-generation sequencing and mass spectrometry, has accelerated the development of several single-omics disciplines. These include genomics, which reveals the genetic basis of diseases by analyzing gene variations; transcriptomics, which explores dynamic gene regulatory networks through RNA expression profiling; proteomics, which systematically identifies protein composition and post-translational modifications using mass spectrometry; and metabolomics, which investigates metabolic responses under both physiological and pathological conditions through comprehensive metabolite profiling. However, single-omics technologies provide only fragmented insights, each focusing on a specific molecular dimension, and fail to capture the intricate and dynamic interactions between molecular layers, which are critical for understanding the complexity of diseases. In contrast, basic-omics and high-spatial-resolution (single-cell and spatial) integration technologies, which combine data from genomics, transcriptomics, proteomics, metabolomics, single-cell, and spatial omics, offer a more comprehensive framework for decoding the complexities of neurological diseases by enabling a deeper understanding of the molecular networks and pathophysiological mechanisms underlying neurodegenerative disorders and other neurological conditions. This review deeply examines the core principles of single-omics technologies and their applications in neurological disease research, highlighting multi-omics and high-spatial-resolution integration's technological advancements, biological significance, and clinical translation potential while addressing challenges like data integration complexities, standardization issues, and high computational demands in multi-omics approaches. Studies suggest multi-omics technologies hold substantial potential for transforming precision diagnostics, biomarker discovery, therapeutic target identification, and patient stratification in neurological diseases, while also offering innovative opportunities for cross-disciplinary fields like synthetic biology beyond advancing personalized treatment strategies.

We believe that this work aligns with the scope of the prestigious journal, GigaScience, given the journal's focus on the intersection of multiple omics technologies and integrative biological approaches. We confirm that this work is original and has not been published elsewhere, nor is currently under consideration for publication elsewhere. We have no conflicts of interest to disclose.

Thank you very much for considering our submission.

Sincerely,
Xiuyun

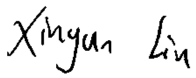

Xiuyun Liu, Professor
Dean of School of Pharmaceutical Sciences and Technology
Tianjin University
Tianjin, China, 300072
Email: xiuyun_liu@tju.edu.cn
Tel: +86 1382059467

Click here to access/download;Graphical Abstract;Graphical_Abstract.docx

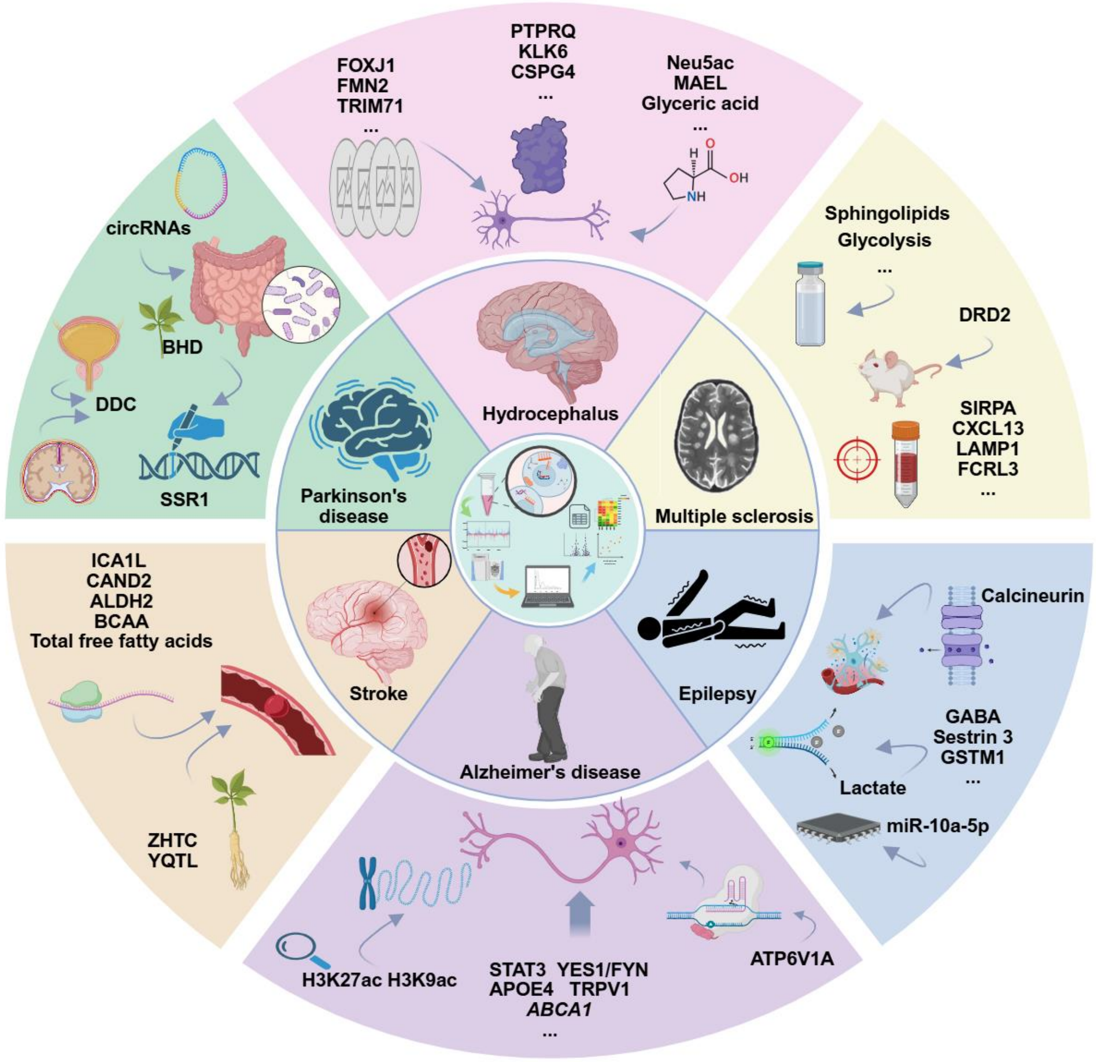

Applications of multi-omics and high-spatial-resolution omics technologies in the diagnosis and treatment of brain diseases in the field of neurology. By integrating high-throughput omics technologies, including four basic omics, single-cell, and spatial omics technologies, it is possible to dissect the complex pathogenic mechanisms of neurological disorders comprehensively. This multi-omics approach spans multiple levels, from genetic variations to metabolic changes, and reveals the interactions between these levels, providing an unprecedented perspective for in-depth disease understanding. In the study of diseases such as AD, PD, stroke, epilepsy, MS, and hydrocephalus, the application of these cutting-edge technologies has greatly facilitated the discovery of key biomarkers and significantly deepened our understanding of the molecular mechanisms of disease pathogenesis.

Dear editors and all the reviewers,

Thank you very much for your comments and feedback. We do appreciate your valuable time reviewing this manuscript, as well as your consideration of our work. We have carefully read through the comments on our manuscript and would like to express our sincere gratitude for the insightful and helpful comments. We have thoroughly revised our manuscript and we hope the revised version meets the requirements of the journal for publication.

In summary, we have revised the opening section of each disease to enhance reader engagement, structuring the content around the core challenges of the disease and how multi-omics technologies address these issues and drive advancements. We have also supplemented the conclusion section of each disease with specific descriptions of current challenges and future research directions—through a review of each disease, we discuss the limitations of existing technologies and the key priorities for overcoming disease-related hurdles. Additionally, we have labeled the experimental verifiability of each disease in all tables throughout the manuscript, specifying the validation type (e.g., human-based, animal model-based, or validated using multiple independent datasets). Furthermore, we have integrated the latest multi-omics dataset findings for each disease in a bid to render the review more comprehensive and up-to-date. Please find our detailed responses to your comments below. Finally, we have also conducted a comprehensive review of all gene and protein names throughout the manuscript to ensure their compliance with relevant nomenclature standards.

Once again, thank you for your time and consideration. We look forward to your feedback on the revised manuscript.

Sincerely,

Xiuyun Liu

Authors' Responses to Reviewers:

Reviewer #1:

Minor concerns:

1. It would be helpful to clearly distinguish between findings that remain at the omics-data level and those that have been experimentally validated. Omics analyses often reveal correlations rather than causality. Among the cases cited, which rely solely on data-driven evidence and which are supported by experimental validation should be explicitly specified.

Response 1. Thank you for your suggestion. We fully agree with your point that clearly distinguishing between omics-data-level findings and experimentally validated results is crucial for enhancing the rigor and clarity of our research. Following the reviewer's suggestion, an extra column has been added to the summary table for each disease, detailing whether the experiment was validated and specifying the validation type, including independent cohort-based validation, animal model-based validation, and validation using multiple independent datasets. **Please refer to Table for Supplementary Table S1-S6, Page 71-106.**

2. The current structure places heavy emphasis on uniform technical descriptions, with each section organized primarily around methodological perspectives. This makes the review somewhat monotonous. A more engaging approach would be to start each section by posing a key scientific question in the context of brain disorders, discuss the challenges in addressing it without omics, and then explain how omics technologies have advanced understanding of the problem. Such a structure would improve the narrative and reader engagement.

Response 2. We totally agree with the review and thank you for your suggestion. Furthermore, the framework you proposed, which involves opening each section with key scientific questions in the context of brain disorders, discussing the challenges inherent in addressing such questions without omics technologies, and subsequently

elaborating on how omics approaches have advanced our understanding of these issues, provides a clear and invaluable direction for optimizing the narrative logic of our work and enhancing reader engagement. We have added and modified the corresponding content before describing the histological techniques for each disease section.

Please refer to Line 175-193, Paragraph 2-3, Page 12.

The field of AD intervention has long been confronted with two major challenges: first, the pronounced delay in early diagnosis—owing to its insidious onset, most patients are identified only at moderate to severe stages when pathological progression has become largely irreversible[59], resulting in a critical missed window for intervention; second, the incomplete understanding of its pathogenetic mechanisms—although core pathological features such as amyloid plaques and tau neurofibrillary tangles have been identified[52,54], the molecular regulatory networks underlying subsequent neuronal injury, neuroinflammation, and gut microbiome dysbiosis remain poorly elucidated[55]. Prior to the emergence of multi-omics technologies, these challenges were compounded by methodological limitations. Conventional hypothesis-driven approaches largely focused on isolated pathways such as Aβ deposition or tau phosphorylation[54]. While valuable for elucidating specific pathological components, such strategies failed to capture system-level interactions among gene expression, protein networks, and metabolic alterations. This constrained the discovery of early specific biomarkers and hindered the development of integrated therapeutic strategies targeting multiple pathological processes.

The advent of multi-omics approaches has provided a pivotal toolkit to overcome these limitations[60]. This global perspective transcends the constraints of single-pathway investigations and marks a shift from compartmentalized pathological observation towards systematic mechanistic decoding. It provides a robust foundation for the identification of early intervention targets and the development of precision therapeutics, heralding a new era in AD research and clinical management.

Please refer to Line 397-408, Paragraph 3, Page 21-22.

A central unresolved question in PD research is how to accurately define disease subtypes and identify early molecular events prior to overt neurodegeneration. Without multi-omics approaches, studies were often limited to clinical phenotypes and single molecular layers, failing to capture the systemic interactions among genetic variants, transcriptional dysregulation, protein misfolding, and metabolic dysfunction. This narrow view hindered the discovery of predictive biomarkers and subtype-specific therapeutic targets, particularly at pre-symptomatic stages. The application of integrated genomics, transcriptomics, proteomics, and metabolomics has transformed this paradigm. These technologies enable high-resolution mapping of endophenotypes and dynamic pathways, revealing novel biomarkers such as GPNMB and DDC, and unmasking critical mechanisms like neuroinflammation, oxidative stress, and mitochondrial impairment. Multi-omics thus provides a holistic, mechanistic framework for early diagnosis, patient stratification, and targeted interventions in PD.

Please refer to Line 519-526, Paragraph 2, Page 27.

A fundamental challenge in epilepsy research is how to decipher the complex, multi-dimensional interactions among genetic predisposition, neuronal excitability, inflammatory processes, and metabolic dysregulation that collectively contribute to seizure generation and progression. Prior to the advent of multi-omics technologies, research was often siloed into isolated domains—electrophysiology, candidate genes, or histopathology—which provided fragmentary insights but failed to capture the systemic and dynamic nature of epileptogenesis. This limitation hindered the identification of convergent pathways and translatable biomarkers, especially across different epilepsy subtypes and developmental stages.

Please refer to Line 652-662, Paragraph 1, Page 33.

A major challenge in MS research is understanding how immune, inflammatory, and degenerative processes interact across disease stages. Traditional methods offered limited insight into system-wide changes, hindering biomarker discovery and targeted

therapy development. The complex and heterogeneous mechanisms of MS remain incompletely understood, impeding targeted therapeutic development. Multi-omics technologies offer a comprehensive approach to uncover molecular underpinnings and identify novel biomarkers and therapeutic targets.Multi-omics approaches have revolutionized MS research by integrating spatial, single-cell, and molecular data. These technologies reveal key cell subtypes, lesion-specific biomarkers, and critical pathways such as APOE-TREM2 and CXCL12-CXCR4. These advances are paving the way for improved stratification mechanisms and precision medicine interventions in MS.

Please refer to Line 756-763, Paragraph 2, Page 38.

A major challenge in stroke research is grasping the spatiotemporal dynamics of molecular and cellular responses to acute injury and recovery. Before multi-omics, studies were confined to single pathways or cell types, missing system-wide interactions across key networks and hindering the discovery of reliable biomarkers and neuroprotective strategies, especially early post-stroke. Multi-omics have revolutionized this by enabling high-resolution, integrated profiling of transcriptional, proteomic, and metabolic changes in a cell- and region-specific way. This has revealed key mechanisms like microglia-endothelial crosstalk and immune-metabolic axes (e.g., SPP1-CD44), and identified novel biomarkers and targets (e.g., LILRB4, LGALS9) for stroke treatment.

Please refer to Line 878-884, Paragraph 2, Page 43.

Multi-omics technologies integrate genomic, proteomic, and metabolomic data to systematically dissect molecular networks underlying hydrocephalus, enabling the discovery of novel biomarkers such as CSPG4 to improve early diagnosis and dynamic monitoring of disease progression. These approaches further uncover central pathophysiological mechanisms—including choroid plexus inflammation and ciliary dysfunction—and identify potential therapeutic targets within dysregulated signaling pathways (e.g., TGF-β, VEGF) and metabolic perturbations, thereby providing new

avenues for developing precision interventions.

3. While the review provides a good summary of past progress, it pays relatively little attention to open questions in the field and potential avenues for future solutions. Beyond summarizing existing achievements, a strong review should also highlight unresolved challenges (both in neurological disease research and in the development and application of omics technologies) and discuss how emerging omics strategies might address them. This aspect should be strengthened.

Response 3. We are deeply grateful for your perceptive comment. In the revised manuscript, we have added a dedicated section at the end of each disease chapter—including stroke, epilepsy, AD, and others—outlining the major unresolved challenges and future directions for omics research. In addition, we describe in detail the common set of challenges facing neurological disorders and the future direction of multi-omics technology in the Discussion section.

Please refer to Line 368-376, Paragraph 4, Page 20.

Despite advancements in multi-omics technologies, AD research remains heavily reliant on classic biomarkers—Aβ and tau, which continue to dominate both mechanistic and clinical frameworks. While omics approaches have identified numerous candidate biomarkers and pathways, few have been rigorously validated in large, diverse cohorts or translated into clinically usable tools. A major persistent challenge is the fragmented integration of multi-omics data: genomic, proteomic, and metabolomic layers are often studied in isolation rather than as interconnected networks. This lack of systematic integration limits our ability to uncover actionable biological mechanisms or subtype-specific signatures, hindering the development of personalized diagnostic and therapeutic strategies.

Please refer to Line 492-504, Paragraph 2, Page 26.

Despite advances in multi-omics research, the field of PD continues to grapple with

three fundamental challenges: clinical heterogeneity (such as differential treatment responses between tremor-dominant and postural instability/gait difficulty subtypes), poorly understood mechanisms of α-synuclein propagation (particularly via proposed gut-to-brain pathways through the vagus nerve), and a persistent lack of disease-modifying therapies. Future studies should prioritize deeply phenotyped longitudinal cohorts—incorporating real-time motor monitoring via wearable sensors and comprehensive autonomic function assessments—alongside the integration of single-cell sequencing and spatial transcriptomics to elucidate region-specific neurodegeneration in the substantia nigra. The development of advanced computational models (e.g., multimodal data fusion and Mendelian randomization) will be essential to infer causal pathways from high-dimensional data. By focusing on prodromal cohorts and currently underrepresented populations, researchers can systematically unravel neuroimmune-metabolic cross-talk, ultimately enabling the discovery of early biomarkers, patient stratification, and personalized therapeutic strategies.

Please refer to Line 628-638, Paragraph 2, Page 32.

The pathogenesis of epilepsy is highly complex, involving multilayered biological processes. Different types of epilepsy may exhibit distinct etiologies and mechanisms, complicating both research and therapeutic development. Furthermore, significant clinical heterogeneity among patients adds another layer of difficulty to studying the disease. Multi-omics studies in epilepsy generate vast amounts of data across genomic, transcriptomic, epigenomic, and other molecular levels. These insights extend beyond pathogenesis to offer novel diagnostic biomarkers—like miR-10a-5p and ALDH2—and reveal new therapeutic targets, exemplifying how multi-omics bridges mechanistic discovery and precision medicine in epilepsy. Integrating and analyzing these datasets require high-performance computing resources as well as systematic and statistically robust analytical frameworks and mathematical models. A major ongoing challenge lies in the development of efficient computational strategies to process and interpret these complex, high-dimensional data.

Please refer to Line 729-740 Paragraph 3, Page 36-37.

Multi-omics technologies have systematically uncovered key mechanisms in MS, including dysregulated $CD4^{+}$ T cell–B cell crosstalk, impaired TREM2-DAP12 signaling in microglia, and disrupted differentiation of oligodendrocyte precursor cells. While potential therapeutic targets such as SEMA4D and CXCL13 have been identified, their functional roles in remyelination and immune infiltration require further validation through in vivo gene editing and organoid models. Critical challenges remain in predicting the transition from relapsing-remitting to secondary progressive MS, deciphering compartmentalized inflammation within chronic lesions, and elucidating mechanisms of resistance to B cell-targeted therapies. Future efforts should prioritize longitudinal multi-omic profiling of well-annotated conversion-phase cohorts, integration of digital pathology with real-time wearable sensor data, and application of artificial intelligence—such as graph neural networks and multimodal learning—to uncover clinically actionable biomarkers and enable precision medicine interventions.

Please refer to Line 849-859, Paragraph 2, Page 42.

While multi-omics technologies hold promise for advancing precision medicine in stroke, addressing critical challenges—including pronounced disease heterogeneity, complex repair mechanisms, and difficulties in translating omic findings into clinical applications—remains essential to fully realize their potential. It is crucial to validate biomarkers and therapeutic targets across diverse stroke populations and to develop targeted delivery systems for neuroprotective agents. However, current stroke multi-omics datasets remain limited in scale and diversity, and integrating multimodal data is complicated by missing values, batch effects, and lack of standardization. Future research should prioritize longitudinal multi-omics profiling to capture dynamic disease progression, incorporate single-cell and spatial technologies to resolve cell- and region-specific responses, and leverage artificial intelligence for integrated analysis of multimodal data.

Please refer to Line 943-951, Paragraph 2, Page 46.

However, multi-omics studies in hydrocephalus face considerable challenges due to the relative difficulty in obtaining CSF samples, which are often limited in volume. Furthermore, the low abundance of proteins and metabolites in CSF necessitates highly sensitive detection technologies. Preanalytical variables during sample collection, processing, and storage can also significantly affect data accuracy and reproducibility. Critically, the scarcity of these precious samples has resulted in a notable paucity of high-quality omics datasets in hydrocephalus, making existing data exceptionally valuable. Overcoming these barriers will require intensified research efforts, standardized protocols, and enhanced support from funding agencies and policymakers to facilitate larger-scale sample acquisition and multi-institutional collaboration.

Reviewer #2

Major concerns:

1. Overall, this manuscript is thorough. However, to enhance its impact and ensure the review remains current and informative, the authors are encouraged to incorporate more up-to-date and comprehensive datasets in their analysis. Several recent published AD snucRNAseq datasets, e.g., PMIDs: 31042697 and 39198642, and the PsychAD project should be reviewed and appropriately cited.

Response 1. We sincerely thank the reviewer for this helpful suggestion. We have now incorporated and discussed these recommended snucRNA-seq datasets, including the PsychAD project, to enhance the timeliness and comprehensiveness of our review. **Please refer to Line 252-254, Paragraph 2, Page 15.**

The experiment also revealed gender differences that exist in AD, including transcriptional response differences and the overrepresentation of females in AD-associated cell subpopulations.

Please refer to Line 275-282, Paragraph 3, Page 16.

Using single-nucleus RNA sequencing (snRNA-seq) and spatial transcriptomic data, one study constructed a comprehensive brain cell atlas comprising 1.65 million single-nucleus transcriptomes from 437 aged individuals. It identified glial and neuronal subpopulations associated with AD, and employed causal modeling to highlight lipid-associated microglial subsets—which drive Aβ pathology or mediate Aβ-TAU crosstalk—alongside TAU-responsive astrocytic populations. These findings provide novel cellular insights into AD pathogenesis and suggest promising therapeutic targets. Furthermore, the identification of distinct brain aging trajectories may facilitate early diagnosis and personalized treatment strategies[78].

2. **In addition, for epigenomic datasets, including recent ATAC-seq datasets (e.g., PMIDs 35931864 and 37774680), should also be considered, as they offer important insights into chromatin accessibility in the AD brain.**

Response 2. We greatly appreciate this insightful comment. Following the reviewer's recommendation, we have included discussion of recent ATAC-seq datasets reflecting chromatin accessibility changes in AD.

Please refer to Line 283-296, Paragraph 4, Page 16-17.

Recent multi-omics studies on human primary microglia have elucidated how genetic regulation of chromatin accessibility influences transcriptional programs. Integrative transcriptomic and epigenomic analyses nominated SPI1/PU.1 as a key transcriptional regulator of microglial gene expression and AD risk, with reduced PU.1 binding destabilizing chromatin accessibility. These findings reinforce the central role of SPI1/PU.1 in microglial dysfunction in AD, while also identifying additional contributing transcription factors[79]. Through single-cell epigenomics and transcriptomics analysis of the prefrontal cortex, the study by Chen et al. constructed a cell-type-resolved regulome atlas of the human brain, revealing significant enrichment of AD risk loci in microglial enhancers and binding sites of transcription factors including SPI1, ELF2, and RUNX1. Previous research identified 9,628 cell-type-specific ATAC-QTLs and integrated these with peak-to-gene linkages to decipher variant-regulatory circuits in AD. Furthermore, the experiment uncovered differential accessibility of regulatory modules in glial cells during late-stage AD and in neurons at early stages, with advanced AD exhibiting global epigenomic dysregulation, suggesting widespread epigenomic erosion and loss of cellular identity[80].

3. Multiome datasets that integrate transcriptomic and epigenomic layers (e.g., PMID 39402379IF: 20.0 Q1) are particularly relevant and would strengthen the manuscript's discussion of integrative analyses.

Response 3. We thank the reviewer for highlighting the importance of multiomic integration. Accordingly, we have added content related to multiome datasets (such as the cited study) that simultaneously profile transcriptomic and epigenomic layers,

strengthening our discussion of integrated omics analyses.

Please refer to Line 297-303, Paragraph 2, Page 17.

One recent study employed single-cell epigenomics and spatial genomics to characterize AD cell populations, defining two disease stages: an early phase featuring slow pathological buildup with inflammatory microglia, reactive astrocytes, loss of SST^{+} inhibitory neurons, and remyelination; and a late phase marked by exponential pathology increase and loss of excitatory and inhibitory (Pvalb^{+}/Vip^{+}) neurons. These findings were validated in independent AD cohorts, and the systematic application of multi-omics technologies coupled with quantitative neuropathology enables effective modeling of disease progression across the spectrum of AD severity[81].

4. This suggestion is not limited to AD; for each neurological disorder reviewed, the authors should aim to incorporate the most recent omics datasets and studies to reflect the current state of the field.

Response 4. Thank you for your suggestion regarding the dataset. We have systematically updated the omics datasets and incorporated the most recent literature across all neurological disorders covered in this review, including AD, PD, stroke, MS, epilepsy, and hydrocephalus. These additions ensure a more comprehensive and up-to-date representation of the latest research advances. We have also supplemented the relevant figures and tables accordingly to provide a thorough and systematic overview of the most current progress in each disease area.

Please refer to Line 304-310, Paragraph 3, Page 17.

Through snRNA-seq combined with spatially resolved transcriptomics, Maria et al. demonstrated that compared to sporadic AD, autosomal dominant AD cases exhibit pronounced upregulation of autophagy-related and chaperone genes. Spatial transcriptomic analysis further confirmed the specific activation of chaperone-mediated autophagy pathways in PSEN1-E280A carriers. In autosomal dominant cases, they observed astrocytic overexpression of LRP1, elevated FKBP1B expression, and reduced neuronal PSEN1 levels, which may collectively underlie a

protective mechanism against AD pathology[82].

Please refer to Line 533-556, Paragraph 2, Page 28-29.

By integrating analyses of gray matter volume with transcriptomic data from the human brain atlas, one study revealed that patients with focal to bilateral tonic–clonic seizures (FBTCS+) exhibit more extensive bilateral cortical and subcortical morphological alterations compared to the non-FBTCS group (FBTCS−). Both groups were involved in neurodevelopmental and synaptic-related pathways. Cell-type mapping demonstrated that both excitatory and inhibitory neurons were affected in the FBTCS− group, whereas only excitatory neurons showed significant changes in the FBTCS+ group, suggesting that excitatory/inhibitory imbalance and abnormalities in the GABAergic system may constitute a key mechanism underlying FBTCS[124]. Through the establishment of a GABRA4 knockout (GABRA4$^{-/-}$) mouse model, the study found that the mice exhibit core behavioral phenotypes resembling high-functioning autism, including social deficits, enhanced spatial memory, and reduced susceptibility to seizures. Subsequent hippocampal transcriptomic and interactome analyses revealed that the NMDAR signaling pathway plays a central regulatory role in mediating these autism-like behaviors and anti-epileptic phenotypes, indicating a functional convergence between the GABAergic system and NMDA receptor pathways in the pathogenesis of autism. Gabra4 knockout mice exhibited significantly decreased susceptibility to pentylenetetrazol-induced seizures, indicating a protective effect against epileptic activity[125]. By integrating [18F]SynVesT-1 PET synaptic imaging and transcriptomic data, research revealed reorganization in the synaptic density similarity network in TLE, characterized by reduced connectivity, decreased clustering, and increased path length, particularly within temporolimbic and frontoparietal circuits. These network alterations correlated spatially with TLE risk gene expression and identified 183 downregulated synaptic transmission-related genes, including core GABAergic genes such as RBFOX1. The findings suggest that coordinated downregulation of risk genes may contribute to synaptic network dysfunction and epileptogenesis in TLE, offering new directions for molecular and

genetic network-targeted therapies[126].

Please refer to Line 657-664, Paragraph 2, Page 33-34.

A study involving over 20,000 MS patients has definitively established cells of the central nervous system—particularly inhibitory neurons—as pivotal cellular contributors to MS susceptibility. Specific risk variants, such as those influencing the STAT3 and IL7 genes, were found to affect gene expression exclusively within inhibitory neurons, revealing that cell-intrinsic dysregulation of immune-related pathways within neurons directly contributes to MS pathogenesis. These findings imply that future preventive and therapeutic strategies must incorporate interventions targeting CNS-specific pathways, including neuronal and glial functions, moving beyond solely immunosuppressive approaches[146].

Please refer to Line 765-773, Paragraph 3, Page 38-39.

Comprehensive RNA-sequencing analysis revealed that transcriptional responses to ischemic stroke occur in a similar direction but with markedly different magnitude in aged compared to young mouse brains. Specifically, aged mice exhibited downregulation of genetic programs associated with axonal and synaptic integrity, coupled with significant upregulation of the type I interferon (IFN-I) signaling pathway. This enhanced IFN-I response was primarily driven by microglia and oligodendrocytes. The increased vulnerability to stroke in aged brains was largely attributable to excessive activation of pro-inflammatory pathways rather than a failure of neurooprotective mechanisms[169].

Please refer to Line 816-827, Paragraph 2, Page 441.

A study utilizing spatial transcriptomics and GWAS compared different regions along the hemodynamic direction of human carotid artery plaques and found that plaque rupture primarily occurs in the proximal and most stenotic areas. Key genes such as MMP-9 were identified to have a causal relationship with rupture risk. The findings provide a theoretical basis for precise interventions targeting MMP-9 and other

relevant targets in stroke patients[179]. A recent study integrating high-resolution omics technologies—including single-cell transcriptomics, proteomics, and spatial transcriptomics—revealed that proximal astrocytes exhibit divergence in lipid handling, with enriched expression of APOE and FABP5 following cortical ischemic stroke. The data reported here demonstrate the acquisition of distinct astrocyte states post-stroke, governed by both temporal and spatial proximity to the lesion, while demonstrating the utility of a high-content single-cell tool termed tDISCO, which identified both the spatial boundaries and molecular profiles defining these distinct populations[180].

Please refer to Line 910-917, Paragraph 2, Page 45.

By integrating genomic and scRNA-seq approaches, a study systematically characterizes the evolutionary, temporal, and spatial expression profiles of maelstrom spermatogenic transposon silencer (MAEL) in the developing human brain, and functionally validates its expression in human hydrocephalic brain tissue. scRNA-seq analyses of the cortical plate and germinal matrix regions identified robust MAEL expression within neurogenic niches, wherein reduced MAEL expression may induce large-scale genomic rearrangements that disrupt cortical development, volume, and function. These findings suggest that diminished MAEL expression may contribute to the pathogenesis of hydrocephalus via multiple etiological pathways[198].

Minor comments

1. Gene names should be italicized throughout the manuscript to adhere to standard formatting conventions.

Response 1. Thank you for pointing out the issue related to genetic nomenclature. We have carefully revised the formatting of all gene and protein symbols in the manuscript to meet the standard nomenclature requirements, including the proper italicization of gene symbols. All corresponding changes are clearly marked in the original document. **Please refer to the highlighted parts throughout the manuscript for specific revisions.**
