# Supplementary material for: Multi-omics and high-spatial-resolution omics: deciphering complexity in neurological disorders
Source: Gigascience. 2025 Dec 5;14:giaf137. doi: 10.1093/gigascience/giaf137 (PMC12723665; doi:10.1093/gigascience/giaf137)
Supplement: giaf137_Authors_Response_To_Reviewer_Comments_Original_Submission [file giaf137_authors_response_to_reviewer_comments_original_submission.pdf]

Author's Response To Reviewer Comments

Dear editors and all the reviewers,
Thank you very much for your comments and feedback. We do appreciate your valuable time reviewing this manuscript, as well as your consideration of our work. We have carefully read through the comments on our manuscript and would like to express our sincere gratitude for the insightful and helpful comments. We have thoroughly revised our manuscript and we hope the revised version meets the requirements of the journal for publication.
In summary, we have revised the opening section of each disease to enhance reader engagement, structuring the content around the core challenges of the disease and how multi-omics technologies address these issues and drive advancements. We have also supplemented the conclusion section of each disease with specific descriptions of current challenges and future research directions—through a review of each disease, we discuss the limitations of existing technologies and the key priorities for overcoming disease-related hurdles. Additionally, we have labeled the experimental verifiability of each disease in all tables throughout the manuscript, specifying the validation type (e.g., human-based, animal model-based, or validated using multiple independent datasets). Furthermore, we have integrated the latest multi-omics dataset findings for each disease in a bid to render the review more comprehensive and up-to-date. Please find our detailed responses to your comments below. Finally, we have also conducted a comprehensive review of all gene and protein names throughout the manuscript to ensure their compliance with relevant nomenclature standards.
Once again, thank you for your time and consideration. We look forward to your feedback on the revised manuscript.

Sincerely,
Xiuyun Liu

Authors' Responses to Reviewers:
Reviewer #1:
Minor concerns:
1. It would be helpful to clearly distinguish between findings that remain at the omics-data level and those that have been experimentally validated. Omics analyses often reveal correlations rather than causality. Among the cases cited, which rely solely on data-driven evidence and which are supported by experimental validation should be explicitly specified.

Response 1. Thank you for your suggestion. We fully agree with your point that clearly distinguishing between omics-data-level findings and experimentally validated results is crucial for enhancing the rigor and clarity of our research. Following the reviewer's suggestion, an extra column has been added to the summary table for each disease, detailing whether the experiment was validated and specifying the validation type, including independent cohort-based validation, animal model-based validation, and validation using multiple independent datasets. Please refer to Table for Supplementary Table S1-S6, Page 71-106.

2. The current structure places heavy emphasis on uniform technical descriptions, with each section organized primarily around methodological perspectives. This makes the review somewhat monotonous. A more engaging approach would be to start each section by posing a key scientific question in the context of brain disorders, discuss the challenges in addressing it without omics, and then explain how omics technologies have advanced understanding of the problem. Such a structure would improve the narrative and reader engagement.

Response 2. We totally agree with the review and thank you for your suggestion.
Furthermore, the framework you proposed, which involves opening each section with key scientific questions in the context of brain disorders, discussing the challenges inherent in addressing such questions without omics technologies, and subsequently elaborating on how omics approaches have advanced our understanding of these issues, provides a clear and invaluable direction for optimizing the narrative logic of our work and enhancing reader engagement. We have added and modified the corresponding content

before describing the histological techniques for each disease section.
Please refer to Line 175-193, Paragraph 2-3, Page 12.
The field of AD intervention has long been confronted with two major challenges: first, the pronounced delay in early diagnosis—owing to its insidious onset, most patients are identified only at moderate to severe stages when pathological progression has become largely irreversible[59], resulting in a critical missed window for intervention; second, the incomplete understanding of its pathogenetic mechanisms—although core pathological features such as amyloid plaques and tau neurofibrillary tangles have been identified[52,54], the molecular regulatory networks underlying subsequent neuronal injury, neuroinflammation, and gut microbiome dysbiosis remain poorly elucidated[55]. Prior to the emergence of multi-omics technologies, these challenges were compounded by methodological limitations. Conventional hypothesis-driven approaches largely focused on isolated pathways such as Aβ deposition or tau phosphorylation[54]. While valuable for elucidating specific pathological components, such strategies failed to capture system-level interactions among gene expression, protein networks, and metabolic alterations. This constrained the discovery of early specific biomarkers and hindered the development of integrated therapeutic strategies targeting multiple pathological processes.
The advent of multi-omics approaches has provided a pivotal toolkit to overcome these limitations[60]. This global perspective transcends the constraints of single-pathway investigations and marks a shift from compartmentalized pathological observation towards systematic mechanistic decoding. It provides a robust foundation for the identification of early intervention targets and the development of precision therapeutics, heralding a new era in AD research and clinical management.

Please refer to Line 397-408, Paragraph 3, Page 21-22.
A central unresolved question in PD research is how to accurately define disease subtypes and identify early molecular events prior to overt neurodegeneration. Without multi-omics approaches, studies were often limited to clinical phenotypes and single molecular layers, failing to capture the systemic interactions among genetic variants, transcriptional dysregulation, protein misfolding, and metabolic dysfunction. This narrow view hindered the discovery of predictive biomarkers and subtype-specific therapeutic targets, particularly at pre-symptomatic stages. The application of integrated genomics, transcriptomics, proteomics, and metabolomics has transformed this paradigm. These technologies enable high-resolution mapping of endophenotypes and dynamic pathways, revealing novel biomarkers such as GPNMB and DDC, and unmasking critical mechanisms like neuroinflammation, oxidative stress, and mitochondrial impairment. Multi-omics thus provides a holistic, mechanistic framework for early diagnosis, patient stratification, and targeted interventions in PD.

Please refer to Line 519-526, Paragraph 2, Page 27.
A fundamental challenge in epilepsy research is how to decipher the complex, multi-dimensional interactions among genetic predisposition, neuronal excitability, inflammatory processes, and metabolic dysregulation that collectively contribute to seizure generation and progression. Prior to the advent of multi-omics technologies, research was often siloed into isolated domains—electrophysiology, candidate genes, or histopathology—which provided fragmentary insights but failed to capture the systemic and dynamic nature of epileptogenesis. This limitation hindered the identification of convergent pathways and translatable biomarkers, especially across different epilepsy subtypes and developmental stages.

Please refer to Line 652-662, Paragraph 1, Page 33.
A major challenge in MS research is understanding how immune, inflammatory, and degenerative processes interact across disease stages. Traditional methods offered limited insight into system-wide changes, hindering biomarker discovery and targeted therapy development. The complex and heterogeneous mechanisms of MS remain incompletely understood, impeding targeted therapeutic development. Multi-omics technologies offer a comprehensive approach to uncover molecular underpinnings and identify novel biomarkers and therapeutic targets.Multi-omics approaches have revolutionized MS research by integrating spatial, single-cell, and molecular data. These technologies reveal key cell subtypes, lesion-specific biomarkers, and critical pathways such as APOE-TREM2 and CXCL12-CXCR4. These advances are paving the way for improved stratification mechanisms and precision medicine interventions in MS.

Please refer to Line 756-763, Paragraph 2, Page 38.
A major challenge in stroke research is grasping the spatiotemporal dynamics of molecular and cellular

responses to acute injury and recovery. Before multi-omics, studies were confined to single pathways or cell types, missing system-wide interactions across key networks and hindering the discovery of reliable biomarkers and neuroprotective strategies, especially early post-stroke. Multi-omics have revolutionized this by enabling high-resolution, integrated profiling of transcriptional, proteomic, and metabolic changes in a cell- and region-specific way. This has revealed key mechanisms like microglia-endothelial crosstalk and immune-metabolic axes (e.g., SPP1-CD44), and identified novel biomarkers and targets (e.g., LILRB4, LGALS9) for stroke treatment.

Please refer to Line 878-884, Paragraph 2, Page 43.
Multi-omics technologies integrate genomic, proteomic, and metabolomic data to systematically dissect molecular networks underlying hydrocephalus, enabling the discovery of novel biomarkers such as CSPG4 to improve early diagnosis and dynamic monitoring of disease progression. These approaches further uncover central pathophysiological mechanisms—including choroid plexus inflammation and ciliary dysfunction—and identify potential therapeutic targets within dysregulated signaling pathways (e.g., TGF-β, VEGF) and metabolic perturbations, thereby providing new avenues for developing precision interventions.

3. While the review provides a good summary of past progress, it pays relatively little attention to open questions in the field and potential avenues for future solutions. Beyond summarizing existing achievements, a strong review should also highlight unresolved challenges (both in neurological disease research and in the development and application of omics technologies) and discuss how emerging omics strategies might address them. This aspect should be strengthened.

Response 3. We are deeply grateful for your perceptive comment. In the revised manuscript, we have added a dedicated section at the end of each disease chapter—including stroke, epilepsy, AD, and others—outlining the major unresolved challenges and future directions for omics research. In addition, we describe in detail the common set of challenges facing neurological disorders and the future direction of multi-omics technology in the Discussion section.
Please refer to Line 368-376, Paragraph 4, Page 20.
Despite advancements in multi-omics technologies, AD research remains heavily reliant on classic biomarkers—Aβ and tau, which continue to dominate both mechanistic and clinical frameworks. While omics approaches have identified numerous candidate biomarkers and pathways, few have been rigorously validated in large, diverse cohorts or translated into clinically usable tools. A major persistent challenge is the fragmented integration of multi-omics data: genomic, proteomic, and metabolomic layers are often studied in isolation rather than as interconnected networks. This lack of systematic integration limits our ability to uncover actionable biological mechanisms or subtype-specific signatures, hindering the development of personalized diagnostic and therapeutic strategies.

Please refer to Line 492-504, Paragraph 2, Page 26.
Despite advances in multi-omics research, the field of PD continues to grapple with three fundamental challenges: clinical heterogeneity (such as differential treatment responses between tremor-dominant and postural instability/gait difficulty subtypes), poorly understood mechanisms of α-synuclein propagation (particularly via proposed gut-to-brain pathways through the vagus nerve), and a persistent lack of disease-modifying therapies. Future studies should prioritize deeply phenotyped longitudinal cohorts—incorporating real-time motor monitoring via wearable sensors and comprehensive autonomic function assessments—alongside the integration of single-cell sequencing and spatial transcriptomics to elucidate region-specific neurodegeneration in the substantia nigra. The development of advanced computational models (e.g., multimodal data fusion and Mendelian randomization) will be essential to infer causal pathways from high-dimensional data. By focusing on prodromal cohorts and currently underrepresented populations, researchers can systematically unravel neuroimmune-metabolic cross-talk, ultimately enabling the discovery of early biomarkers, patient stratification, and personalized therapeutic strategies.

Please refer to Line 628-638, Paragraph 2, Page 32.
The pathogenesis of epilepsy is highly complex, involving multilayered biological processes. Different types of epilepsy may exhibit distinct etiologies and mechanisms, complicating both research and therapeutic development. Furthermore, significant clinical heterogeneity among patients adds another layer of difficulty to studying the disease. Multi-omics studies in epilepsy generate vast amounts of data across genomic,

transcriptomic, epigenomic, and other molecular levels. These insights extend beyond pathogenesis to offer novel diagnostic biomarkers—like miR-10a-5p and ALDH2—and reveal new therapeutic targets, exemplifying how multi-omics bridges mechanistic discovery and precision medicine in epilepsy. Integrating and analyzing these datasets require high-performance computing resources as well as systematic and statistically robust analytical frameworks and mathematical models. A major ongoing challenge lies in the development of efficient computational strategies to process and interpret these complex, high-dimensional data.

Please refer to Line 729-740 Paragraph 3, Page 36-37.
Multi-omics technologies have systematically uncovered key mechanisms in MS, including dysregulated $CD4^+$ T cell–B cell crosstalk, impaired TREM2-DAP12 signaling in microglia, and disrupted differentiation of oligodendrocyte precursor cells. While potential therapeutic targets such as SEMA4D and CXCL13 have been identified, their functional roles in remyelination and immune infiltration require further validation through in vivo gene editing and organoid models. Critical challenges remain in predicting the transition from relapsing-remitting to secondary progressive MS, deciphering compartmentalized inflammation within chronic lesions, and elucidating mechanisms of resistance to B cell-targeted therapies. Future efforts should prioritize longitudinal multi-omic profiling of well-annotated conversion-phase cohorts, integration of digital pathology with real-time wearable sensor data, and application of artificial intelligence—such as graph neural networks and multimodal learning—to uncover clinically actionable biomarkers and enable precision medicine interventions.

Please refer to Line 849-859, Paragraph 2, Page 42.
While multi-omics technologies hold promise for advancing precision medicine in stroke, addressing critical challenges—including pronounced disease heterogeneity, complex repair mechanisms, and difficulties in translating omic findings into clinical applications—remains essential to fully realize their potential. It is crucial to validate biomarkers and therapeutic targets across diverse stroke populations and to develop targeted delivery systems for neuroprotective agents. However, current stroke multi-omics datasets remain limited in scale and diversity, and integrating multimodal data is complicated by missing values, batch effects, and lack of standardization. Future research should prioritize longitudinal multi-omics profiling to capture dynamic disease progression, incorporate single-cell and spatial technologies to resolve cell- and region-specific responses, and leverage artificial intelligence for integrated analysis of multimodal data.

Please refer to Line 943-951, Paragraph 2, Page 46.
However, multi-omics studies in hydrocephalus face considerable challenges due to the relative difficulty in obtaining CSF samples, which are often limited in volume. Furthermore, the low abundance of proteins and metabolites in CSF necessitates highly sensitive detection technologies. Preanalytical variables during sample collection, processing, and storage can also significantly affect data accuracy and reproducibility. Critically, the scarcity of these precious samples has resulted in a notable paucity of high-quality omics datasets in hydrocephalus, making existing data exceptionally valuable. Overcoming these barriers will require intensified research efforts, standardized protocols, and enhanced support from funding agencies and policymakers to facilitate larger-scale sample acquisition and multi-institutional collaboration.

Reviewer #2
Major concerns:
1. Overall, this manuscript is thorough. However, to enhance its impact and ensure the review remains current and informative, the authors are encouraged to incorporate more up-to-date and comprehensive datasets in their analysis. Several recent published AD snucRNAseq datasets, e.g., PMIDs: 31042697 and 39198642, and the PsychAD project should be reviewed and appropriately cited.

Response 1. We sincerely thank the reviewer for this helpful suggestion. We have now incorporated and discussed these recommended snucRNA-seq datasets, including the PsychAD project, to enhance the timeliness and comprehensiveness of our review.
Please refer to Line 252-254, Paragraph 2, Page 15.
The experiment also revealed gender differences that exist in AD, including transcriptional response differences and the overrepresentation of females in AD-associated cell subpopulations.

Please refer to Line 275-282, Paragraph 3, Page 16.
Using single-nucleus RNA sequencing (snRNA-seq) and spatial transcriptomic data, one study constructed a comprehensive brain cell atlas comprising 1.65 million single-nucleus transcriptomes from 437 aged individuals. It identified glial and neuronal subpopulations associated with AD, and employed causal modeling to highlight lipid-associated microglial subsets—which drive Aβ pathology or mediate Aβ-TAU crosstalk—alongside TAU-responsive astrocytic populations. These findings provide novel cellular insights into AD pathogenesis and suggest promising therapeutic targets. Furthermore, the identification of distinct brain aging trajectories may facilitate early diagnosis and personalized treatment strategies[78].

2. In addition, for epigenomic datasets, including recent ATAC-seq datasets (e.g., PMIDs 35931864 and 37774680), should also be considered, as they offer important insights into chromatin accessibility in the AD brain.

Response 2. We greatly appreciate this insightful comment. Following the reviewer's recommendation, we have included discussion of recent ATAC-seq datasets reflecting chromatin accessibility changes in AD.
Please refer to Line 283-296, Paragraph 4, Page 16-17.
Recent multi-omics studies on human primary microglia have elucidated how genetic regulation of chromatin accessibility influences transcriptional programs. Integrative transcriptomic and epigenomic analyses nominated SPI1/PU.1 as a key transcriptional regulator of microglial gene expression and AD risk, with reduced PU.1 binding destabilizing chromatin accessibility. These findings reinforce the central role of SPI1/PU.1 in microglial dysfunction in AD, while also identifying additional contributing transcription factors[79]. Through single-cell epigenomics and transcriptomics analysis of the prefrontal cortex, the study by Chen et al. constructed a cell-type-resolved regulome atlas of the human brain, revealing significant enrichment of AD risk loci in microglial enhancers and binding sites of transcription factors including SPI1, ELF2, and RUNX1. Previous research identified 9,628 cell-type-specific ATAC-QTLs and integrated these with peak-to-gene linkages to decipher variant-regulatory circuits in AD. Furthermore, the experiment uncovered differential accessibility of regulatory modules in glial cells during late-stage AD and in neurons at early stages, with advanced AD exhibiting global epigenomic dysregulation, suggesting widespread epigenomic erosion and loss of cellular identity[80].

3. Multiome datasets that integrate transcriptomic and epigenomic layers (e.g., PMID 39402379IF: 20.0 Q1) are particularly relevant and would strengthen the manuscript's discussion of integrative analyses.

Response 3. We thank the reviewer for highlighting the importance of multiomic integration. Accordingly, we have added content related to multiome datasets (such as the cited study) that simultaneously profile transcriptomic and epigenomic layers, strengthening our discussion of integrated omics analyses.
Please refer to Line 297-303, Paragraph 2, Page 17.
One recent study employed single-cell epigenomics and spatial genomics to characterize AD cell populations, defining two disease stages: an early phase featuring slow pathological buildup with inflammatory microglia, reactive astrocytes, loss of SST^+ inhibitory neurons, and remyelination; and a late phase marked by exponential pathology increase and loss of excitatory and inhibitory ($Pvalb^+$/Vip^+) neurons. These findings were validated in independent AD cohorts, and the systematic application of multi-omics technologies coupled with quantitative neuropathology enables effective modeling of disease progression across the spectrum of AD severity[81].

4. This suggestion is not limited to AD; for each neurological disorder reviewed, the authors should aim to incorporate the most recent omics datasets and studies to reflect the current state of the field.
Response 4. Thank you for your suggestion regarding the dataset. We have systematically updated the omics datasets and incorporated the most recent literature across all neurological disorders covered in this review, including AD, PD, stroke, MS, epilepsy, and hydrocephalus. These additions ensure a more comprehensive and up-to-date representation of the latest research advances. We have also supplemented the relevant figures and tables accordingly to provide a thorough and systematic overview of the most current progress in each disease area.
Please refer to Line 304-310, Paragraph 3, Page 17.
Through snRNA-seq combined with spatially resolved transcriptomics, Maria et al. demonstrated that compared to sporadic AD, autosomal dominant AD cases exhibit pronounced upregulation of autophagy-related and chaperone genes. Spatial transcriptomic analysis further confirmed the specific activation of

chaperone-mediated autophagy pathways in PSEN1-E280A carriers. In autosomal dominant cases, they observed astrocytic overexpression of LRP1, elevated FKBP1B expression, and reduced neuronal PSEN1 levels, which may collectively underlie a protective mechanism against AD pathology[82].

Please refer to Line 533-556, Paragraph 2, Page 28-29.
By integrating analyses of gray matter volume with transcriptomic data from the human brain atlas, one study revealed that patients with focal to bilateral tonic–clonic seizures (FBTCS+) exhibit more extensive bilateral cortical and subcortical morphological alterations compared to the non-FBTCS group (FBTCS−). Both groups were involved in neurodevelopmental and synaptic-related pathways. Cell-type mapping demonstrated that both excitatory and inhibitory neurons were affected in the FBTCS− group, whereas only excitatory neurons showed significant changes in the FBTCS+ group, suggesting that excitatory/inhibitory imbalance and abnormalities in the GABAergic system may constitute a key mechanism underlying FBTCS[124]. Through the establishment of a GABRA4 knockout (GABRA4$^{-/-}$) mouse model, the study found that the mice exhibit core behavioral phenotypes resembling high-functioning autism, including social deficits, enhanced spatial memory, and reduced susceptibility to seizures. Subsequent hippocampal transcriptomic and interactome analyses revealed that the NMDAR signaling pathway plays a central regulatory role in mediating these autism-like behaviors and anti-epileptic phenotypes, indicating a functional convergence between the GABAergic system and NMDA receptor pathways in the pathogenesis of autism. Gabra4 knockout mice exhibited significantly decreased susceptibility to pentylenetetrazol-induced seizures, indicating a protective effect against epileptic activity[125]. By integrating [18F]SynVesT-1 PET synaptic imaging and transcriptomic data, research revealed reorganization in the synaptic density similarity network in TLE, characterized by reduced connectivity, decreased clustering, and increased path length, particularly within temporolimbic and frontoparietal circuits. These network alterations correlated spatially with TLE risk gene expression and identified 183 downregulated synaptic transmission-related genes, including core GABAergic genes such as RBFOX1. The findings suggest that coordinated downregulation of risk genes may contribute to synaptic network dysfunction and epileptogenesis in TLE, offering new directions for molecular and genetic network-targeted therapies[126].

Please refer to Line 657-664, Paragraph 2, Page 33-34.
A study involving over 20,000 MS patients has definitively established cells of the central nervous system—particularly inhibitory neurons—as pivotal cellular contributors to MS susceptibility. Specific risk variants, such as those influencing the STAT3 and IL7 genes, were found to affect gene expression exclusively within inhibitory neurons, revealing that cell-intrinsic dysregulation of immune-related pathways within neurons directly contributes to MS pathogenesis. These findings imply that future preventive and therapeutic strategies must incorporate interventions targeting CNS-specific pathways, including neuronal and glial functions, moving beyond solely immunosuppressive approaches[146].

Please refer to Line 765-773, Paragraph 3, Page 38-39.
Comprehensive RNA-sequencing analysis revealed that transcriptional responses to ischemic stroke occur in a similar direction but with markedly different magnitude in aged compared to young mouse brains. Specifically, aged mice exhibited downregulation of genetic programs associated with axonal and synaptic integrity, coupled with significant upregulation of the type I interferon (IFN-I) signaling pathway. This enhanced IFN-I response was primarily driven by microglia and oligodendrocytes. The increased vulnerability to stroke in aged brains was largely attributable to excessive activation of pro-inflammatory pathways rather than a failure of neurooprotective mechanisms[169].

Please refer to Line 816-827, Paragraph 2, Page 441.
A study utilizing spatial transcriptomics and GWAS compared different regions along the hemodynamic direction of human carotid artery plaques and found that plaque rupture primarily occurs in the proximal and most stenotic areas. Key genes such as MMP-9 were identified to have a causal relationship with rupture risk. The findings provide a theoretical basis for precise interventions targeting MMP-9 and other relevant targets in stroke patients[179]. A recent study integrating high-resolution omics technologies—including single-cell transcriptomics, proteomics, and spatial transcriptomics—revealed that proximal astrocytes exhibit divergence in lipid handling, with enriched expression of APOE and FABP5 following cortical ischemic stroke. The data reported here demonstrate the acquisition of distinct astrocyte states post-stroke, governed by both temporal and spatial proximity to the lesion, while demonstrating the utility

of a high-content single-cell tool termed tDISCO, which identified both the spatial boundaries and molecular profiles defining these distinct populations[180].

Please refer to Line 910-917, Paragraph 2, Page 45.
By integrating genomic and scRNA-seq approaches, a study systematically characterizes the evolutionary, temporal, and spatial expression profiles of maelstrom spermatogenic transposon silencer (MAEL) in the developing human brain, and functionally validates its expression in human hydrocephalic brain tissue. scRNA-seq analyses of the cortical plate and germinal matrix regions identified robust MAEL expression within neurogenic niches, wherein reduced MAEL expression may induce large-scale genomic rearrangements that disrupt cortical development, volume, and function. These findings suggest that diminished MAEL expression may contribute to the pathogenesis of hydrocephalus via multiple etiological pathways[198].

Minor comments
1. Gene names should be italicized throughout the manuscript to adhere to standard formatting conventions.

Response 1. Thank you for pointing out the issue related to genetic nomenclature. We have carefully revised the formatting of all gene and protein symbols in the manuscript to meet the standard nomenclature requirements, including the proper italicization of gene symbols. All corresponding changes are clearly marked in the original document. Please refer to the highlighted parts throughout the manuscript for specific revisions.

Editorial Manager®

Cookie Preference Center

We use cookies which are necessary to make our site work. We may also use additional cookies to analyze, improve and personalize our content and your digital experience. For more information, see our Cookie Policy.

You may choose not to allow some types of cookies. However, blocking some types may impact your experience of our site and the services we are able to offer. See the different category headings below to find out more or change your settings.

Allow all

Manage Consent Preferences

Strictly Necessary Cookies

Always active

These cookies are necessary for the website to function and cannot be switched off in our systems. They are usually only set in response to actions made by you which amount to a request for services, such as setting your privacy preferences, logging in or filling in forms. You can set your browser to block or alert you about these cookies, but some parts of the site will not then work.

Functional Cookies

These cookies enable the website to provide enhanced functionality and personalisation. They may be set by us or by third party providers whose services we have added to our pages. If you do not allow these cookies then some or all of these services may not function properly.

Performance Cookies

These cookies allow us to count visits and traffic sources so we can measure and improve the performance of our site. They help us to know which pages are the most and least popular and see how visitors move around the site.

Targeting Cookies

These cookies may be set through our site by our advertising partners. They may be used by those companies to build a profile of your interests and show you relevant adverts on other sites. If you do not allow these cookies, you will experience less targeted advertising.

Cookie List

Clear

Apply Cancel

Consent Leg.Interest

Confirm my choices

Powered by onetrust
