# Supplementary material for: Multi-omics and high-spatial-resolution omics: deciphering complexity in neurological disorders
Source: Gigascience. 2025 Dec 5;14:giaf137. doi: 10.1093/gigascience/giaf137 (PMC12723665; doi:10.1093/gigascience/giaf137)
Supplement: giaf137_Authors_Response_To_Reviewer_Comments_Revision_1 [file giaf137_authors_response_to_reviewer_comments_revision_1.pdf]

Author's Response To Reviewer Comments

Dear editors and all the reviewers,
Thank you very much for your comments and feedback. We do appreciate your valuable time reviewing this manuscript, as well as your consideration of our work. We have carefully read through the comments on our manuscript and would like to express our sincere gratitude for the insightful and helpful comments. We have thoroughly revised our manuscript, and we hope the revised version meets the requirements of the journal for publication.
In summary, we conducted a comprehensive revision of the entire manuscript following the reviewer's suggestion, especially in the results section and the discussion section, to precisely reflect our own thoughts and ideas. All the modifications were highlighted in yellow. Please refer to:
Once again, thank you for your time and consideration. We look forward to your feedback on the revised manuscript.

Sincerely,
Xiuyun Liu

Editorial Manager®

Cookie Preference Center

We use cookies which are necessary to make our site work. We may also use additional cookies to analyze, improve and personalize our content and your digital experience. For more information, see our Cookie Policy.

You may choose not to allow some types of cookies. However, blocking some types may impact your experience of our site and the services we are able to offer. See the different category headings below to find out more or change your settings.

Allow all

Manage Consent Preferences

Strictly Necessary Cookies

Always active

These cookies are necessary for the website to function and cannot be switched off in our systems. They are usually only set in response to actions made by you which amount to a request for services, such as setting your privacy preferences, logging in or

filling in forms. You can set your browser to block or alert you about these cookies, but some parts of the site will not then work.

Functional Cookies

These cookies enable the website to provide enhanced functionality and personalisation. They may be set by us or by third party providers whose services we have added to our pages. If you do not allow these cookies then some or all of these services may not function properly.

Performance Cookies

These cookies allow us to count visits and traffic sources so we can measure and improve the performance of our site. They help us to know which pages are the most and least popular and see how visitors move around the site.

Targeting Cookies

These cookies may be set through our site by our advertising partners. They may be used by those companies to build a profile of your interests and show you relevant adverts on other sites. If you do not allow these cookies, you will experience less targeted advertising.

Cookie List

Clear

Apply Cancel
Consent Leg.Interest

Confirm my choices

Powered by onetrust
