# Supplementary material for: Multi-omics and high-spatial-resolution omics: deciphering complexity in neurological disorders
Source: Gigascience. 2025 Dec 5;14:giaf137. doi: 10.1093/gigascience/giaf137 (PMC12723665; doi:10.1093/gigascience/giaf137)
Supplement: giaf137_Reviewer_1_Report_Original_Submission — Chuan Xu -- 9/15/2025 [file giaf137_reviewer_1_report_original_submission.pdf]

Reviewer Report

Title: Multi-Omics and High-Spatial-Resolution Omics: Deciphering Complexity in Neurological Disorders

Version: Original Submission **Date:** 9/15/2025

Reviewer name: Chuan Xu

Reviewer Comments to Author:

Unlike psychiatric disorders, neurological diseases are often accompanied by organic alterations. Therefore, the acquisition and analysis of omics data from disease tissues can yield valuable insights in the study of these diseases. This article provides a timely and systematic review, summarizing applications of various omics approaches across six types of neurological disorders. I find it to be a solid contribution, but I have a few minor suggestions:It would be helpful to clearly distinguish between findings that remain at the omics-data level and those that have been experimentally validated. Omics analyses often reveal correlations rather than causality. Among the cases cited, which rely solely on data-driven evidence and which are supported by experimental validation should be explicitly specified.The current structure places heavy emphasis on uniform technical descriptions, with each section organized primarily around methodological perspectives. This makes the review somewhat monotonous. A more engaging approach would be to start each section by posing a key scientific question in the context of brain disorders, discuss the challenges in addressing it without omics, and then explain how omics technologies have advanced understanding of the problem. Such a structure would improve the narrative and reader engagement.While the review provides a good summary of past progress, it pays relatively little attention to open questions in the field and potential avenues for future solutions. Beyond summarizing existing achievements, a strong review should also highlight unresolved challenges (both in neurological disease research and in the development and application of omics technologies) and discuss how emerging omics strategies might address them. This aspect should be strengthened.

Level of Interest

Please indicate how interesting you found the manuscript: Choose an item.

Quality of Written English

Please indicate the quality of language in the manuscript: Choose an item.

Declaration of Competing Interests

Please complete a declaration of competing interests, considering the following questions:

- Have you in the past five years received reimbursements, fees, funding, or salary from an organisation that may in any way gain or lose financially from the publication of this manuscript, either now or in the future?
- Do you hold any stocks or shares in an organisation that may in any way gain or lose financially from the publication of this manuscript, either now or in the future?
- Do you hold or are you currently applying for any patents relating to the content of the manuscript?
- Have you received reimbursements, fees, funding, or salary from an organization that holds or has applied for patents relating to the content of the manuscript?
- Do you have any other financial competing interests?
- Do you have any non-financial competing interests in relation to this paper?

If you can answer no to all of the above, write 'I declare that I have no competing interests' below. If your reply is yes to any, please give details below.

I declare that I have no competing interests

I agree to the open peer review policy of the journal. I understand that my name will be included on my report to the authors and, if the manuscript is accepted for publication, my named report including any attachments I upload will be posted on the website along with the authors' responses. I agree for my report to be made available under an Open Access Creative Commons CC-BY license (http://creativecommons.org/licenses/by/4.0/). I understand that any comments which I do not wish to be included in my named report can be included as confidential comments to the editors, which will not be published.

Choose an item.

To further support our reviewers, we have joined with Publons, where you can gain additional credit to further highlight your hard work (see: https://publons.com/journal/530/gigascience). On publication of this paper, your review will be automatically added to Publons, you can then choose whether or not to claim your Publons credit. I understand this statement.

Yes Choose an item.
