# Supplementary material for: Multi-omics and high-spatial-resolution omics: deciphering complexity in neurological disorders
Source: Gigascience. 2025 Dec 5;14:giaf137. doi: 10.1093/gigascience/giaf137 (PMC12723665; doi:10.1093/gigascience/giaf137)
Supplement: giaf137_Reviewer_2_Report_Original_Submission — Lu Zeng -- 9/15/2025 [file giaf137_reviewer_2_report_original_submission.pdf]

Reviewer Report

Title: Multi-Omics and High-Spatial-Resolution Omics: Deciphering Complexity in Neurological Disorders

Version: Original Submission **Date:** 9/15/2025

Reviewer name: Lu Zeng

Reviewer Comments to Author:

This review provides a comprehensive analysis of the recent advancements in multi- and high-spatial-resolution omics, with a focus on their applications in precision diagnostics, biomarker discovery, and therapeutic target identification in brain diseases.Overall, this manuscript is thorough. However, to enhance its impact and ensure the review remains current and informative, the authors are encouraged to incorporate more up-to-date and comprehensive datasets in their analysis.Major concernsSeveral recent published AD snucRNAseq datasets, e.g., PMIDs: 31042697 and 39198642, and the PsychAD project should be reviewed and appropriately cited.In addition, for epigenomic datasets, including recent ATAC-seq datasets (e.g., PMIDs 35931864 and 37774680), should also be considered, as they offer important insights into chromatin accessibility in the AD brain.Multiome datasets that integrate transcriptomic and epigenomic layers (e.g., PMID 39402379) are particularly relevant and would strengthen the manuscript's discussion of integrative analyses.This suggestion is not limited to AD; for each neurological disorder reviewed, the authors should aim to incorporate the most recent omics datasets and studies to reflect the current state of the field.Minor commentsGene names should be italicized throughout the manuscript to adhere to standard formatting conventions.

Level of Interest

Please indicate how interesting you found the manuscript: Choose an item.

Quality of Written English

Please indicate the quality of language in the manuscript: Choose an item.

Declaration of Competing Interests

Please complete a declaration of competing interests, considering the following questions:

- Have you in the past five years received reimbursements, fees, funding, or salary from an organisation that may in any way gain or lose financially from the publication of this manuscript, either now or in the future?
- Do you hold any stocks or shares in an organisation that may in any way gain or lose financially from the publication of this manuscript, either now or in the future?

- Do you hold or are you currently applying for any patents relating to the content of the manuscript?
- Have you received reimbursements, fees, funding, or salary from an organization that holds or has applied for patents relating to the content of the manuscript?
- Do you have any other financial competing interests?
- Do you have any non-financial competing interests in relation to this paper?

If you can answer no to all of the above, write 'I declare that I have no competing interests' below. If your reply is yes to any, please give details below.

I declare that I have no competing interests

I agree to the open peer review policy of the journal. I understand that my name will be included on my report to the authors and, if the manuscript is accepted for publication, my named report including any attachments I upload will be posted on the website along with the authors' responses. I agree for my report to be made available under an Open Access Creative Commons CC-BY license (http://creativecommons.org/licenses/by/4.0/). I understand that any comments which I do not wish to be included in my named report can be included as confidential comments to the editors, which will not be published.

Choose an item.

To further support our reviewers, we have joined with Publons, where you can gain additional credit to further highlight your hard work (see: https://publons.com/journal/530/gigascience). On publication of this paper, your review will be automatically added to Publons, you can then choose whether or not to claim your Publons credit. I understand this statement.

Yes Choose an item.
